# The *Tara* Oceans voyage reveals global diversity and distribution patterns of marine planktonic ciliates

**DOI:** 10.1038/srep33555

**Published:** 2016-09-16

**Authors:** Anna Gimmler, Ralf Korn, Colomban de Vargas, Stéphane Audic, Thorsten Stoeck

**Affiliations:** 1 University of Kaiserslautern, Ecology Group, D-67663 Kaiserslautern, Germany; 2 University of Kaiserslautern, Financial Mathematics Group, D-67663 Kaiserslautern, Germany; 3 Fraunhofer Institute for Industrial Mathematics ITWM, D-67663 Kaiserslautern, Germany; 4 CNRS, UMR 7144, Station Biologique de Roscoff, Place Georges Teissier, F-29680 Roscoff, France; 5 Sorbonne Universités, UPMC Univ Paris 06, UMR 7144, Station Biologique de Roscoff, Place Georges Teissier, F-29680 Roscoff, France

## Abstract

Illumina reads of the SSU-rDNA-V9 region obtained from the circumglobal *Tara* Oceans expedition allow the investigation of protistan plankton diversity patterns on a global scale. We analyzed 6,137,350 V9-amplicons from ocean surface waters and the deep chlorophyll maximum, which were taxonomically assigned to the phylum Ciliophora. For open ocean samples global planktonic ciliate diversity is relatively low (ca. 1,300 observed and predicted ciliate OTUs). We found that 17% of all detected ciliate OTUs occurred in all oceanic regions under study. On average, local ciliate OTU richness represented 27% of the global ciliate OTU richness, indicating that a large proportion of ciliates is widely distributed. Yet, more than half of these OTUs shared <90% sequence similarity with reference sequences of described ciliates. While alpha-diversity measures (richness and exp(Shannon H)) are hardly affected by contemporary environmental conditions, species (OTU) turnover and community similarity (β-diversity) across taxonomic groups showed strong correlation to environmental parameters. Logistic regression models predicted significant correlations between the occurrence of specific ciliate genera and individual nutrients, the oceanic carbonate system and temperature. Planktonic ciliates displayed distinct vertical distributions relative to chlorophyll a. In contrast, the *Tara* Oceans dataset did not reveal any evidence that latitude is structuring ciliate communities.

Recent results from the circumglobal *Tara* Oceans expedition impressively demonstrated that heterotrophic protists are even more diverse (and at the same time much less understood) than prokaryotes in the planktonic ocean ecosystem^1^. One such group of heterotrophic plankton is within the alveolates, namely the ciliates. Due to their relatively large size among the protists, ciliates are well studied since more than two centuries and thus, have a model character for protists^2^. Ciliates are morphologically very diverse and may reach abundances of up to tens of thousands of individuals in a litre of water^2^. In the marine food web they play a major role by grazing on pico- and nanoplankton while serving as prey for metazoans, thus acting as mediators of energy transfer to higher trophic levels^3^. Through a top-down control of specific bacterial taxon groups^4^, they furthermore contribute to major biogeochemical cycles.

Despite this knowledge accumulated over more than 200 years, some fundamental questions in ciliate ecology are subjects of an on-going controversial debate. This includes for example the local and global diversity of ciliates, spatial distribution patterns, and also the environmental factors governing these patterns. While for example some researchers find that global ciliate diversity is relatively low and local diversity covers a very high proportion of global diversity^5^,^6^, others find an extremely high global diversity and that the proportion of the global species pool found locally is only moderate^7^,^8^,^9^,^10^,^11^,^12^. Regarding latitudinal diversity gradients for ciliates such as for larger animals and plants^13^, one camp rejects the existence thereof^6^,^14^, while the other provides evidence thereof^15^. Information on environmental factors structuring ciliate plankton communities in the open ocean is hardly available in the current literature. The latter is due to a lack of adequate studies analysing the spatial distribution of ciliate diversity patterns in the context of environmental variables. Likewise, the afore mentioned controversies may partly find their explanation in a lack of adequate studies. Until recently, large-scale biodiversity studies in the sunlit ocean have focused primarily on larger multicellular organisms or prokaryotes. However, protistan plankton communities in general received relatively little attention. Thus far, the taxonomic and ecological diversity (including spatial patterns) of protistan plankton communities was studied on relatively small spatial and taxonomic scales using microscopy^16^, analyses of ribosomal RNA (18S rDNA) clone libraries^17^ or high-throughput sequencing of taxonomic marker gene fragments (environmental metabarcoding)^18^. This also applies to ciliates in specific. Most surveys of planktonic ciliate communities occurred on a small local scale^19^, in marginal seas^16^ or coastal waters^20^. Therefore, our current knowledge of marine planktonic ciliate diversity and distribution resembles more or less a patchwork of regionally restricted studies with the vast majority of global oceanic ciliate diversity being presumably unknown^11^,^12^.

Open ocean samples collected during the circumglobal *Tara* Oceans expedition (2009–2012) now provides the opportunity to analyse protistan plankton diversity on an unparalleled scale^21^. In our study, we analyzed >6 million ciliate DNA sequences (V9 region of the SSU rDNA as taxonomic marker gene^1^) collected from nearly all oceanic regions (surface waters and deep chlorophyll maximum) to reveal global ciliate diversity patterns. In specific, we used this global ocean ciliate plankton dataset to test the following hypotheses: (i) Ciliate diversity is a function of environmental variables such as temperature, salinity, oxygen, nutrients and the oceanic carbonate system; (ii) Ciliate diversity is a function of depth (surface waters versus deep chlorophyll maximum); (iii) Ciliate diversity is a function of latitude; (iv) Global marine ciliate plankton diversity is high; and (v) Local diversity covers a small/high proportion of global diversity. Finally, we tested the hypothesis that the extent of ciliate diversity in open ocean waters is still far from being fully appreciated.

## Methods

### Dataset

**Figure 1:**
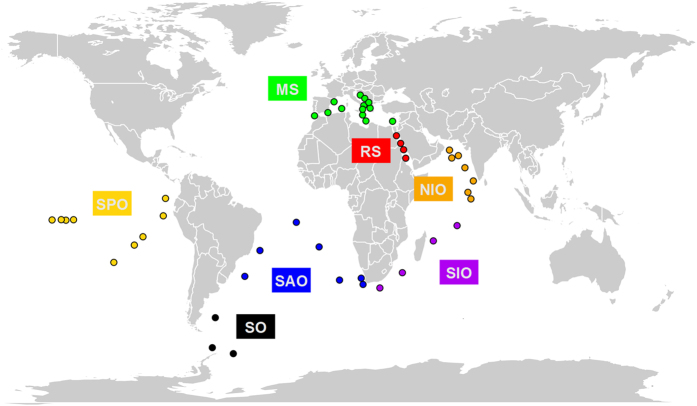
Geographic locations of sampling stations. Planktonic ciliate communities from surface waters and deep chlorophyll maximum layer have been sampled in seven oceanic regions. MS: Mediterranean Sea, RS: Red Sea, NIO: North Indian Ocean, SIO: South Indian Ocean, SAO: South Atlantic Ocean, SO: Southern Ocean, SPO: South Pacific Ocean. This map was created with the R package ‘maps’ v. 2. 3-6 (http://CRAN.R-project.org/package=maps)^68^.

The genomic and environmental data used in this study were collected during the circumglobal *Tara* Oceans expedition^21^ (2009–2012). Plankton samples were collected from surface waters (SRF) and deep chlorophyll maximum (DCM) at 47 stations in seven oceanic regions (Fig. 1, Supplementary Table S1).

Detailed sampling strategy and methodology are described by Pesant and colleagues^22^. In short, plankton samples (at least 0.1 m^3^ seawater) were collected on polycarbonate membranes. Samples were stored frozen until DNA extraction. Detailed information about DNA extraction, PCR amplification and Illumina sequencing of V9 rDNA metabarcodes, as well as subsequent sequence data cleaning and taxonomic assignment can be found in de Vargas *et al*.^1^. In short, DNA was extracted from each polycarbonate membrane and the hypervariable V9 region of the 18S rDNA was then PCR amplified using the universal eukaryotic primers 1389F (5′-TTGTACACACCGCCC-3′) and 1510R (5′-CCTTCYGCAGGTTCACCTAC-3′)^23^. Purified PCR products were Illumina-sequenced using a Genome Analyser IIx system (Illumina, San Diego, CA, USA). Subsequent sequence data cleaning included quality checking (Phred score filtering, elimination of reads without correct primers, and removal of chimeras) and conservative filtering (removal of metabarcodes present in less than three reads and two distinct samples). *Tara* Oceans metabarcodes were taxonomically assigned using the V9 PR^2^ database^24^. V9 ciliate sequence data used in this study are available from the Dryad Digital Repository (http://dx.doi.org/10.5061/dryad.c3n62).

Ciliate V9 rDNA reads with a minimum length of 100 base pairs were clustered into operational taxonomic units (OTUs) at a 97% sequence similarity threshold using a modified script of usearch quality filter (usearch_qf)^25^ and QIIME v. 1.7.0^26^. Details on the modified script can be found in Oikonomou *et al*.^27^. For final taxonomic assignment of the OTUs the representative read (longest) of each OTU was subjected to BLAST analyses^28^ against GenBank’s nr nucleotide database release 200 in JAguc^29^. JAguc employs BLASTn searches with algorithm parameters adjusted for short reads (*-m 7 -r 5 -q -4 -G 8 -E 6 -b 50*). Singletons and doubletons OTUs were excluded from the final dataset to reduce bias based on erroneous reads. The resulting OTU abundance table (Supplementary Table S2) contained 1,274 OTUs and 6,137,350 V9 rDNA sequences.

### Statistical analyses

All statistical analyses were conducted and visualized in R Studio (v. 2.15.1, http://r-projekt.org). Rarefaction analyses were performed in R using the ‘vegan’ package^30^. To predict ciliate OTU richness, we calculated the abundance-based coverage estimator (ACE) and the incidence-based coverage estimator (ICE) using the ‘fossil’ package^31^ in R.

Alpha diversity for each sample was assessed by the exponential of the Shannon entropy H (exp(H), effective number of species^32^) using the ‘vegan’ package^30^ in R. Prior to calculating the effective number of species, the OTU abundance table was randomly subsampled to the lowest number of reads present in any sample. Additionally, the ciliate OTU richness (number of OTUs) of each sample was calculated.

To investigate differences in ciliate OTU compositions between samples the OTU abundance table was transformed to presence-absence data and the binary Jaccard (J_incidence_) index was calculated. Jaccard distances were then analyzed by non-metric multidimensional scaling (NMDS) using the ‘vegan’ package in R^30^. Environmental parameters were fitted to the ordination using the function ‘envfit’ of the ‘vegan’ R package. The fit (R^2^) of each variable to the ordination was assessed with a Monte-Carlo analysis of 10000 permutations.

In general, all data was tested for normal distribution before deciding which statistical test to use (parametric or nonparametric). The Two-Sample t-test was used to test for significant differences in ciliate OTU richness and exp(H) between surface and DCM samples (global dataset). For individual sample comparisons, we used the One-Sample t-test.

Spearman’s rank coefficients were calculated in R to relate ciliate diversity and environmental data (Supplementary Table S1). Multiple logistic regression analyses^33^ in R modelled the relationship between environmental parameters and presence-absence of ciliate genera. Since the complete set of environmental parameters could not be obtained at all sampling sites, we had to run the logistic regression on two independent sets of environmental parameters. The first dataset included temperature, salinity, oxygen and nutrients (NO_2_^−^, NO_3_^−^, PO_4_^3−^, Si(OH)_4_) of 42 surface samples^34^ (Supplementary Table S1). The second dataset included measures of the oceanic carbonate system (pH, CO_2_, pCO_2_, HCO_3_, CO_3_, total alkalinity, total carbon) of 23 surface samples^35^ (Supplementary Table S1). For taxonomic assignment of ciliate OTUs on genus level, we applied a 95% sequence similarity threshold to reference sequences^36^. To define if a genus was present in a sample we used a threshold of 5% of the maximum read abundance of each genus. Only genera, which were present in at least three samples, were used for regression analyses. We defined null models (no relationship between occurrence of ciliate genus and environmental parameters) and full models (relationship between occurrence of ciliate genus and all environmental parameters) for each ciliate genus and then used a stepwise procedure to build the final model based on the Akaike Information Criterion (AIC). The final model included the environmental parameters, which are influencing the occurrence of a ciliate genus. In addition to the overall percentage of correct predictions we also analyzed the sensitivity (proportion of correctly predicted presences) and the specificity (proportion of correctly predicted absences).

### Identification of novel ciliate sequence diversity

To screen for genetically divergent ciliate OTUs we used the Swarm-network approach^37^. First, ciliate reads of the *Tara* Oceans dataset were dereplicated using the custom script as recommended on the Swarm homepage (https://github.com/torognes/swarm). Then, unique reads were clustered using Swarm v1.2.5^38^ with d = 1. From the resulting swarms, the “seed reads” (i.e. the most abundant read of each swarm) of the 200 most abundant swarms were BLASTed^28^ against GenBank’s nr nucleotide database (rel. 200) to identify their taxonomic affiliation. Only swarms assigned to ciliates with a sequence similarity of at least 80% to reference sequences were included into the network analyses. The seed reads were then aligned in Seaview^39^ and pairwise sequence similarities between each pair of seed reads were computed with PairAlign^17^. These pairwise sequence similarities were used to build a network with the ‘igraph’ R package^40^. In the network two nodes (each representing a swarm) were connected by an edge if they shared a pairwise sequence similarity of at least 90%. The resulting network was visualized in Gephi v.0.8.2-beta^41^ according to the swarms’ taxonomic affiliation and BLAST hit value.

## Results

### Local and global distribution of marine planktonic ciliates

The final ciliate dataset consisted of 6,137,350 high quality V9 SSU rDNA reads, which clustered into 1,274 distinct OTUs called at 97% sequence similarity threshold. The number of reads obtained from the different oceanic regions varied between 207,644 reads (Red Sea) and 2,105,449 reads (Mediterranean Sea), whereas the number of OTUs ranged from 401 in the Southern Ocean to 1,024 in the South Pacific Ocean. For surface waters (SRF) we obtained 3,864,389 V9 reads and for the deep chlorophyll maximum (DCM) 2,272,961 reads. These reads clustered into 1,159 and 1,176 OTUs, respectively. Rarefaction analyses indicated near-saturated sampling for most sampled regions (Supplementary Fig. S1) and depths (Supplementary Fig. S2).

**Figure 2:**
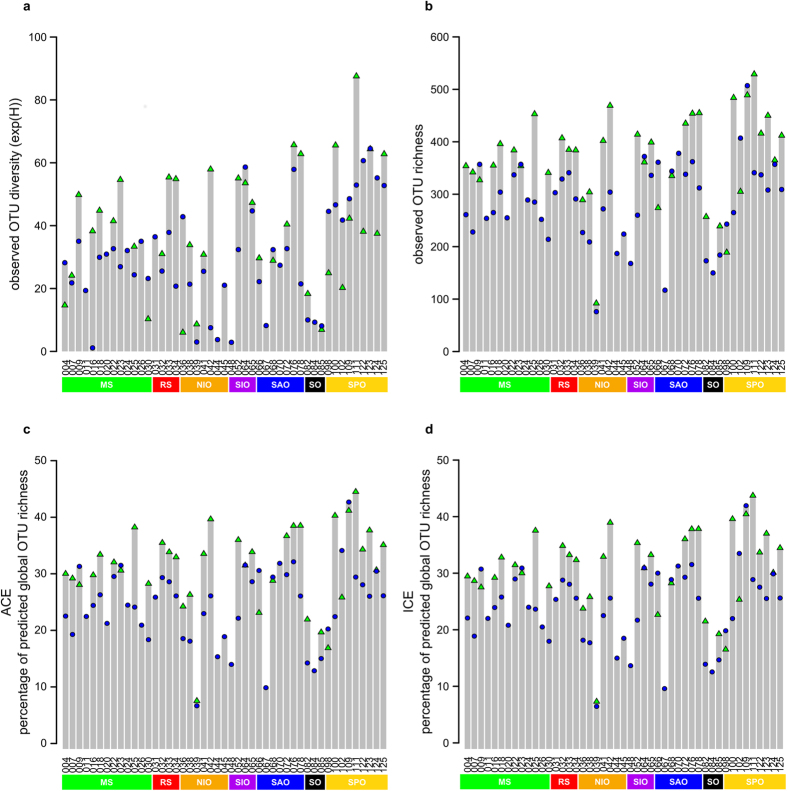
Observed and predicted alpha diversity of ciliate OTUs in the *Tara* Oceans dataset. Observed alpha diversity was assessed by calculating exp(H) (**a**) and ciliate OTU richness (**b**) for 47 surface water samples (blue circles) and 36 DCM samples (green triangles). Local ciliate OTU richness as predicted by ACE (**c**) and ICE (**d**) was compared to the global predicted ciliate OTU richness.

Observed local ciliate OTU alpha diversity in surface waters (SRF) and also in the deep chlorophyll maximum (DCM) varied notably among sites within and also between oceanic regions (Fig. 2a,b). For example, in the South Indian Ocean, exp(H) ranged from 2.9 (station 48) to 58.6 (station 64) in surface waters. The polar waters of the Southern Ocean harboured an exceptionally low mean ciliate OTU diversity (SRF: exp(H) = 9.1 ± 0.9 standard deviation (s.d.), OTU richness 169 ± 17 s.d.; DCM: exp(H) = 12.6 ± 8.1 s.d.; OTU richness 248 ± 13 s.d.), while the South Pacific Ocean was characterized by highest ciliate OTU diversity (SRF: exp(H) = 52 ± 7.5 s.d., OTU richness 342 ± 79 s.d.; DCM: exp(H) = 49.3 ± 22 s.d., OTU richness 404 ± 106 s.d.).

Accordingly, sampling sites in the South Pacific Ocean covered up to 43% (site 111) of the predicted OTU richness of the global ciliate diversity (ACE and ICE = 1,274 OTUs, corroborating with the total number of observed OTUs) while polar waters of the Southern Ocean harboured only a smaller proportion of the global ciliate OTU diversity (site 84, 22%; Fig. 2c,d). On average the predicted local ciliate OTU richness covered 27% (±8% s.d.) of the predicted global ciliate OTU richness (for both ACE and ICE).

**Figure 3:**
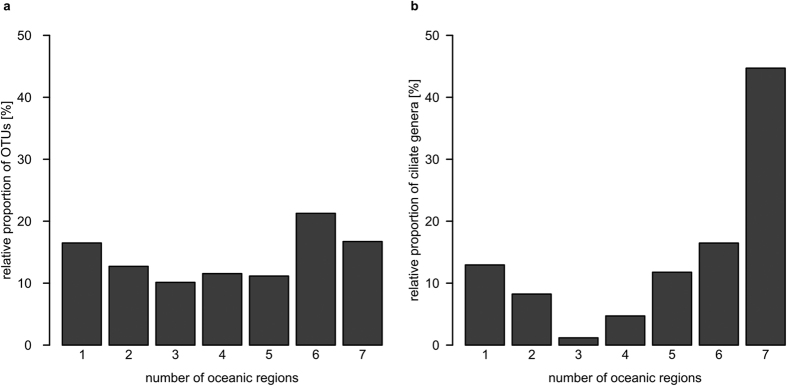
Occurrences of ciliate OTUs and genera in oceanic regions. Proportion of OTUs (**a**) and ciliate genera (**b**) which occurred in a certain number of oceanic regions. Only OTUs which had a sequence similarity of at least 95% to reference sequences were assigned to a genus.

We found that 16% of all OTUs were restricted to only one single oceanic region, and 17% occurred in all oceanic regions (at least in one sample of each region) (Fig. 3a). Among these latter OTUs most occurred at even abundances in all oceanic regions, whereas only few occurred predominantly in only a single oceanic region with high abundances. In other words, low-abundant cosmopolitan OTUs tended to be low-abundant in all oceanic regions and high-abundant cosmopolitan OTUs tended to be high-abundant in all oceanic regions. When assigning taxonomic identities (genera) to OTUs, 13% of all detected ciliate genera were restricted to only one single oceanic region (Fig. 3b), whereas 45% of all ciliate genera occurred in all oceanic regions (at least in one sample of each region).

### Taxonomic composition of ciliate communities

**Figure 4:**
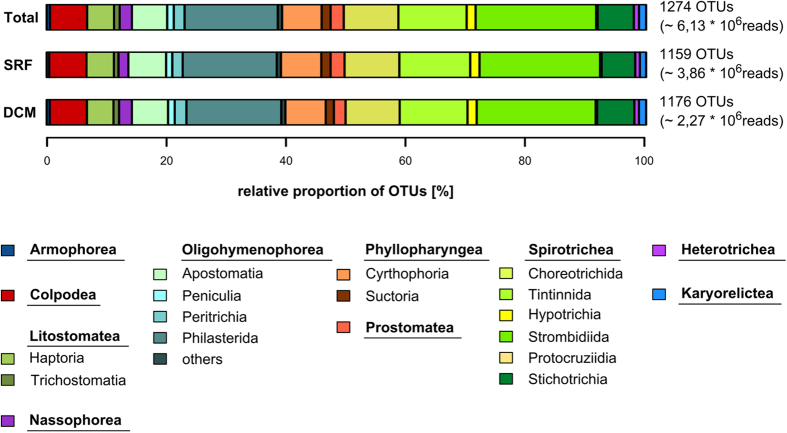
Taxonomic composition of ciliate communities. Proportion of ciliate OTUs detected in the whole dataset (Total), in all surface water samples (SRF) and in all DCM samples (DCM). Numbers on the right side of the bars indicate the number of OTUs and reads. Plagiopylea (1 OTU) were not considered in the diagram. Four ciliate classes (Litostomatea, Oligohymenophorea, Phyllopharyngea, and Spirotrichea) were divided into subgroups. Category “others” in class Oligohymenophorea represents taxonomic groups within the Oligohymenophorea which consisted of less than five OTUs. These were Astomatida, Hymenostomatida, Pleuronematida and Thigmotrichida.

Ciliate OTUs were assigned to eleven (out of 12) different ciliate classes (Fig. 4), originating from 112 families and 191 genera. Cariacotrichea was the only class not detected, while both Armophorea and Plagiopylea were represented with only few OTUs. Most OTUs belonged to the class Spirotrichea (48.4% of all OTUs, n = 616), followed by Oligohymenophorea (25%, 319 OTUs). Within the Spirotrichea, Strombidiida (256 OTUs), and Choreotrichia (Tintinnida, 145 OTUs; Choreotrichida, 116 OTUs) were the most diverse. Within the Oligohymenophorea, Philasterida (199 OTUs) accounted for most of the observed diversity. The third most diverse ciliate class was Phyllopharyngea (8.1%, 103 OTUs), followed by Colpodea (6.2%, 79 OTUs), Litostomatea (5.2%, 69 OTUs), Prostomatea (2.2%, 28 OTUs) and Nassophorea (2%, 26 OTUs). Heterotrichea and Karyorelictea accounted for 0.9% respectively 1.2% of all OTUs. On the class-level, the ciliate community composition varied only moderately across oceanic regions and depths (Supplementary Fig. S3). With only one exception, all observed classes occurred in the SRF and DCM of each oceanic region. The exception refers to Karyorelictea, which were not detected in the SRF of the Southern Ocean. Also Colpodea and Nassophorea were notably less diverse in the Southern Ocean compared to all other oceanic regions.

For a higher taxonomic resolution, we investigated diversity patterns for the five most diverse ciliate classes (Colpodea, Litostomatea, Oligohymenophorea, Phyllopharyngea and Spirotrichea) at the genus-level. Using this higher taxonomic resolution revealed more distinct differences in the structure of ciliate community composition among oceanic regions and depths (Supplementary Figs S4–S8). For statistical community analyses, we subsequently worked with molecular OTUs rather than Linnean taxonomy to further increase resolution.

### Effects of environmental variables, latitude and depth on marine ciliate plankton diversity

Statistical analyses were conducted to reveal if ciliate plankton diversity is a function of environmental variables, latitude and depth (SRF vs. DCM). Ciliate OTU richness correlated significantly only with chlorophyll *a* (Spearman’s rank coefficient: 0.367; p < 0.001, Table 1). None of the other measured and analyzed environmental parameters, nor latitude correlated significantly with ciliate OTU richness. Similarly, none of these parameters correlated significantly with ciliate diversity (exp(H)).

However, ciliate community composition emerged as a function of several environmental variables. Fitting environmental parameters into non-metric multidimensional scaling (NMDS) analyses based on community Jaccard distances (J_incidence_) (Supplementary Fig. S9) identified temperature, salinity, oxygen, nitrate, phosphate and silicate as significant explanatory variables (Table 2).

To model the effect of environmental variables on individual ciliate genera we used multiple logistic regression analyses. The occurrence of 44 ciliate genera were related to different combinations of nutrients, temperature, salinity and oxygen (Supplementary Table S3). The final model of each genus contained between one and four of these environmental parameters. The parameters most often included in models were oxygen, temperature, salinity and nitrite. The averaged sensitivity was 41%, while the specificity averaged 89%. The sensitivity was usually low when the genus was present in less than ten samples. The overall percentage of correct predictions was on a high level for all genera and averaged 80%. The occurrences of 20 ciliate genera were related to the oceanic carbonate system (Supplementary Table S4). The final model of each genus contained between one and three parameters for this dataset, with the most important parameter being pH value, followed by total alkalinity and total carbon. Also for the carbonate system, specificity (82%) was higher than sensitivity (49%). The overall percentage of correct predictions was 74% for the oceanic carbonate system data.

**Figure 5:**
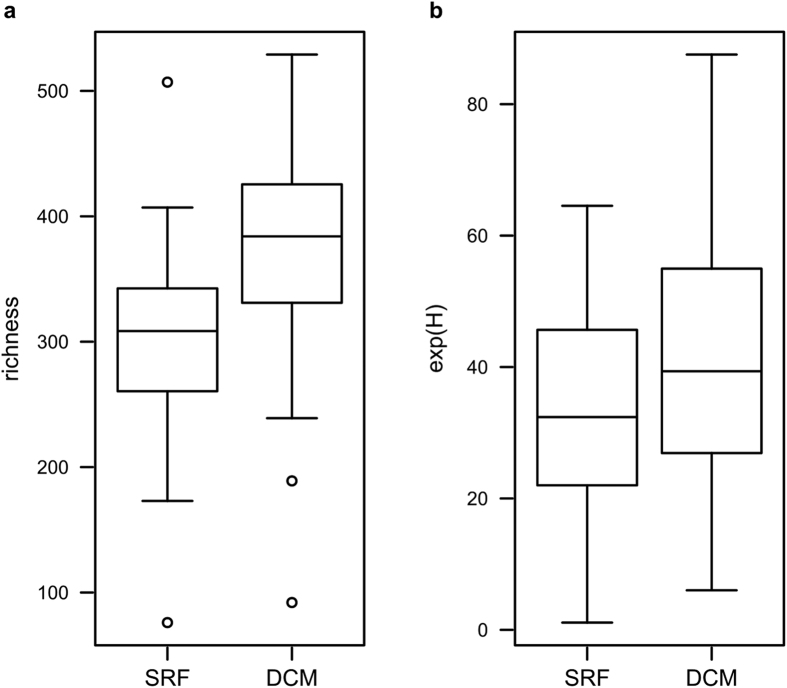
Comparison of diversity in surface and DCM communities. Ciliate OTU richness (**a**) and ciliate OTU diversity (exp(H), **b**) of surface water and DCM samples were depicted as box-whisker-plots. Only 36 sampling stations, of which surface waters and DCM samples were both available, were included in the analysis.

Ciliate communities in surface waters were notably different from ciliate communities in the deep chlorophyll maximum. J_incidence_ distances among ciliate communities of the DCM were significantly smaller when compared to J_incidence_ distances between ciliate communities from the surface waters and the DCM (p < 0.001). This result was also mirrored in significant differences (Two-sample t-test: p < 0.001) between observed ciliate OTU richness in surface waters and the DCM (Fig. 5a). While the mean OTU richness of surface samples was 299 OTUs (±76 OTUs s.d.), this value was 369 OTUs (±88 OTUs s.d.) for the DCM, despite of a higher sequence sample size in the corresponding surface water samples (n sequence reads = 3,327,419) compared to the DCM (n sequence reads = 2,272,961). Also, the average effective number of species (exp(H), Fig. 5b) was lower in the surface water samples (33.21 ± 16.85 s.d.) compared to the DCM (40.1 ± 19.55 s.d.), but insignificant in a Two-sample t-test (p = 0.1163). Considering all individual sample pairs between surface and DCM samples in a One-sample t-test, differences between DCM and surface water samples were significant for both exp(H) (p = 0.0396) and observed ciliate OTU richness (p < 0.0001).

### Uncharted genetic diversity and novel diversity hotspots of planktonic marine ciliates

**Figure 6:**
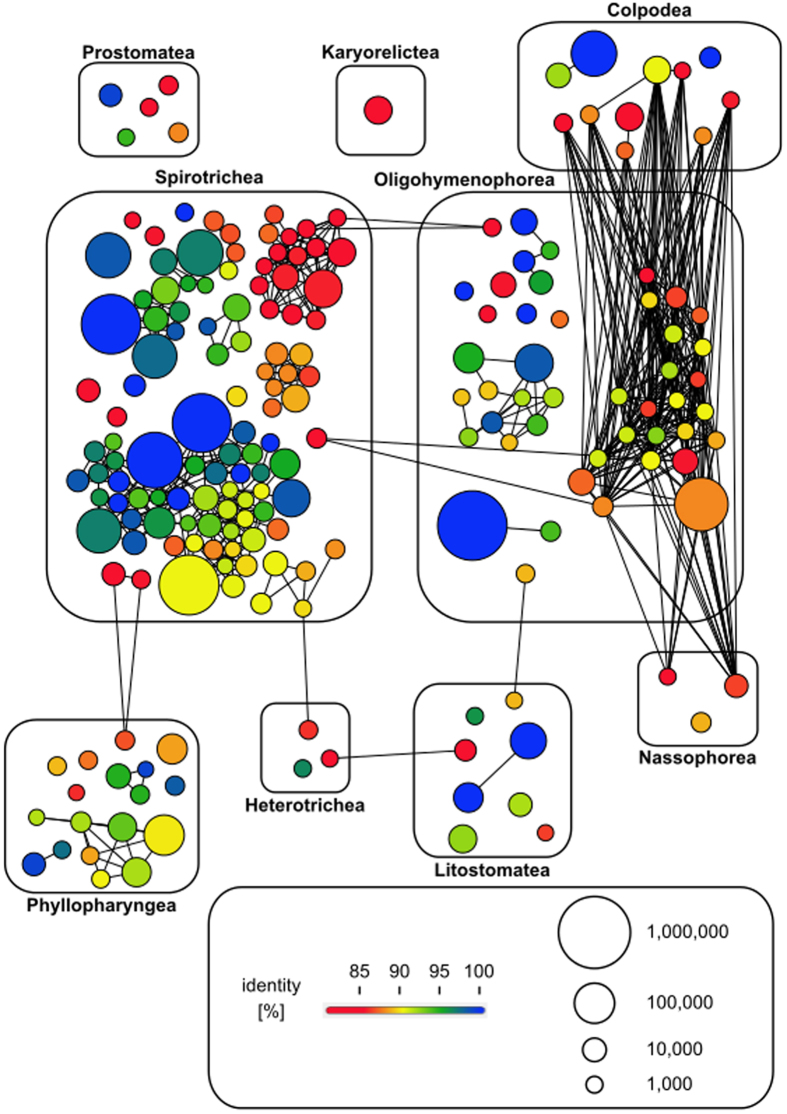
Novel ciliate genetic diversity network of 200 most abundant swarms pooled over depth and oceanic region. Each node in the network represents one swarm. Two nodes are connected by an edge if they share a sequence similarity of at least 90%. The size of a node indicates the number of reads in a swarm. Nodes are colored according to the percentage of identity of the swarms to the closest reference sequences in GenBank. Minimum identity is 80% (red).

In the network of the 200 most abundant swarms nearly all ciliate classes were represented (Fig. 6). Even among the most abundant OTUs, we detected numerous OTUs with poor identity to reference sequences of described ciliates. We found that 82.5% (n = 165) of the 200 most abundant swarms were less than 97% similar to reference sequences. And nearly half of these swarms (45.5%, n = 91) were even less than 90% similar to reference sequences. This evidences a high degree of uncharted genetic diversity in planktonic ciliates. Of the 91 swarms that had <90% similarity to reference sequences, 43 branched within the class Spirotrichea and 21 within the class Oligohymenophorea. Interestingly, these two classes also included the most swarms with the highest similarities to reference sequences. Ten Spirotrichean and five Oligohymenophorean swarms were strictly identical to reference sequences. The lowest similarity exhibited a swarm of the class Litostomatea with only 80.55% similarity to a reference sequence. The mean sequence similarity of all 200 most abundant swarms to reference sequences was 91% (±5.4% s.d.).

To reveal geographic hotspots of novel ciliate diversity, the relative sample-specific proportion of OTUs with <90% and <95% sequence similarity to reference sequences was plotted (Supplementary Fig. S10). The proportion of uncharted ciliate diversity was lowest in the Southern Ocean. None of the remaining six oceanic regions emerged as an extraordinary hotspot of uncharted ciliate diversity. Instead, all regions had a similarly high proportion of ciliate OTUs, which are poorly known. On average, 75% of all OTUs from the seven oceanic regions under study had less than 95% sequence similarity to reference sequences. And half of all OTUs from each sampling site shared even <90% sequence similarity with reference sequences of described species (average proportion of OTUs: 50.3%).

## Discussion

Of the twelve described ciliate classes, only Cariacotrichea were not recovered by the *Tara* Oceans expedition. This class is only known from deep anoxic waters of the Cariaco Basin^42^. Therefore, it is not surprising that this deep-sea clade was not found in the sunlit ocean waters sampled by the *Tara* Oceans expedition. Together, Spirotrichea and Oligohymenophorea accounted for 73% of the total observed ciliate diversity. Both classes are the most diverse morphotype assemblages in the phylum Ciliophora^2^. The class Oligohymenophorea includes a conspicuously high proportion of symbionts and parasites with a variety of different marine hosts^2^. The diversity, abundance and ecological role of symbionts and parasites in the plankton seem to be largely underestimated and deserve more appreciation^1^. Even though mass mortalities of fishes due to ciliate infections are well recognized^2^, the ecological impact of symbiotic and parasitic lifestyles of ciliates on marine plankton communities is largely unknown. Further noteworthy was the discovery of 79 OTUs of the class Colpodea in open ocean waters. Traditionally, colpodeans are considered as terrestrial and freshwater ciliates^2^. However, large-scale sequencing studies of coastal^43^ regions revised our picture of colpodeans as purely terrestrial and freshwater organisms. The same seems to be true for nassophoreans which are known to be more diverse in freshwater and soil ecosystems than in marine habitats^2^.

More than half of all ciliate OTUs shared less than 90% sequence similarity to reference sequences of described ciliates. This number increases up to 75%, when we consider sequences that share <95% sequence similarity to described species. Because 95% sequence similarity is a reasonable proxy to delineate ciliate species in the V9 region^36^, these data corroborate well with previous estimates on uncharted ciliate diversity: Considerations of Foissner and colleagues^11^ based on morphospecies estimated that >80% of ciliate morphospecies are still undescribed. In the past 20 years, only ca. 200 marine ciliate species have been discovered and described^11^. Considering the vanishing expertise in ciliate taxonomy and the speed of novel ciliate species descriptions, it is reasonable to assume that the next decades, if not even centuries, will not succeed to reveal the extant ciliate morphotypes diversity in our oceans. An alternative to traditional taxonomy may be a lineage specific barcoding, which may at least provide a repository for ciliate diversity, its distribution and basic ecological information^44^. Even though, many barcodes will probably never be accompanied by a formal taxonomic description. However, only recently Gimmler and Stoeck^37^ presented a streamlined approach to obtain at least basic morphological and also phylogenetic information from divergent short-tag sequence clades. DNA-barcoding in combination with this approach is probably our best chance for a full (or at least as full as it can get) global ciliate (protistan) diversity repository.

A correlation of ciliate OTU richness with chlorophyll *a* may partly be due to a mathematical artefact (autocorrelation), because pigmented ciliates (mixotrophs) may account for a remarkable proportion of the chlorophyll *a* crop in ocean water^16^. For example, up to 24% of the chlorophyll *a* biomass in Nordic seas was attributed to ciliates^45^. On the other hand, heterotrophic ciliates predominantly feed on pico- and nanophytoplankton and may consume up to 50% of the chlorophyll biomass in ocean waters^16^. Thus, waters with high chlorophyll content probably attract a higher diversity of predatory ciliates than waters with lower chlorophyll *a* content. For other environmental variables measured in this study, our analyses rejected the hypothesis that ciliate diversity (OTU richness and exp(H)) is a function of these parameters. However, while alpha diversity measures are hardly affected, ciliate species (OTU) turnover and community similarity (β-diversity) across taxonomic groups showed a strong correlation to contemporary environmental conditions. Community turnover is a result of the reaction of individual taxa to a (combination of) environmental variables as well as biotic interactions^46^. While the V9 amplicon dataset of the *Tara* Oceans project does not allow testing biotic interactions, logistic regression modelling enabled us to assess the effect of multiple environmental parameters to individual ciliate taxa (genera).

Logistic regression has often been used in ecological studies to model and predict the occurrence and distribution of species^47^. Also for protistan plankton this approach was applied to predict blooms of the toxigenic diatom *Pseudonitzschia*^48^. In contrast to these studies, the logistic regression for planktonic ciliates in this study is based on V9 rDNA reads instead of abundances of cells or individuals. Since logistic regression requires presence-absence data, we applied a threshold to define the occurrence of a genus at a specific sampling site. This strategy avoided statistical bias based on different rDNA copy numbers in ciliates^18^. Our modelling approach achieved high percentages of correct predictions, which ranged from 64.29% to 95.24% for the dataset including nutrients, physical variables and hydrochemistry and from 56.52% to 86.96% for the dataset of the oceanic carbonate system. Nitrite, oxygen, salinity and temperature were identified as significant factors playing in concerto to contribute to ciliate community assembly. Only few studies have evaluated the effect of (mostly individual) environmental parameters on ciliate taxa. In a north Chinese ocean bay a strong correlation between the distribution of non-loricate ciliates and nutrients, including nitrate and nitrite was observed^49^. The significant effect of nitrite on ciliate community assembly that was also observed in our data could be related to the primary nitrite maximum, which forms at the base of the euphotic zone^50^ close to the DCM. Oxygen and salinity were identified as strong parameters structuring ciliate communities using metadata from different ecosystems^51^. For individual ciliate species isolated from different geographic locations significant differences in responses to temperature were observed^52^. For the carbonate system, pH, total alkalinity and total carbon mainly affected the patterns of ciliate genera. The pH-tolerance of ciliates is also poorly documented. One study on freshwater ciliate cultures demonstrated an influence of pH on their geographic distribution^53^. Ocean regime shifts, including nutrient regimes, changes in sea surface temperature evolution and spread of oxygen-depleted zones and ocean acidification are a result of environmental change, intensifying in the coming decades^54^. Thus, shifts in ciliate diversity and distributions patterns are expected in the global oceans. Validated models as well as many more data that allow a focused and detailed modelling may help to predict the nature of plankton shifts in light of environmental change. Furthermore, biotic interactions will have to be included for a reliable predictive modelling. This is essential if we want to assess ecosystem effects caused by environmental change including shifts in plankton regimes.

Even though it is well known that water column stratification caused by e.g. oxyclines, thermoclines or haloclines foster the evolution of different ciliate communities throughout the water column^17^,^55^, not many studies have analyzed whether ocean surface waters and the deep chlorophyll maximum (DCM), often times located only few metres below the surface, select for different ciliate communities. In a microscopy-based study of a single sample site in the north-western Mediterranean Sea, Dolan and colleagues^16^ reported an increase of ciliate biomass with different functional guilds present in the DCM compared to surface waters. Likewise, in a recent molecular diversity study Forster *et al*.^20^ found notably different ciliate communities and predicted a higher ciliate OTU richness in the DCM of European coastal waters compared to surface waters. Commonly, ciliates are considered as herbivores. However, at second sight, ciliate communities are composed of variable proportions of more or less distinct trophic guilds^16^. While some species are strict heterotrophs feeding on pico- and nanophytoplankton, others are mixotrophic species which, in addition to phagotrophic feeding, sequester and exploit chloroplasts from ingested algae^2^. Grazing impacts of planktonic ciliates in the DCM could reach as much as 50% of the primary production^16^. Together with these previous studies, the *Tara* Oceans dataset revealed that also in the (unstratified) upper ocean water column different types of planktonic ciliates display different vertical distributions, relative to chlorophyll *a*, in a system with a well-defined DCM.

Community properties cannot be understood without biogeographical context. However, while some controversially discussed issues in biogeography are difficult (if at all) to test (for example Baas-Beckings^56^ famous “everything is everywhere–but the environment selects” paradigm–one can probably never prove that something is not present), other concepts in biogeography provide a well testable framework.

One important and well-testable aspect in geographic structuring of all living organisms is the relation of diversity (species richness) and latitudes^13^. We did not find a decline of ciliate plankton diversity with latitude (latitudinal diversity gradient–LDG) in the *Tara* Oceans dataset as known for most larger animals and plants^13^. Likewise, species turnover (community similarity, beta diversity) were not identified as a function of latitude. This finding corroborates well with previous assumptions that protists in general and ciliates in specific are less influenced by large-scale spatial processes than animals and plants^5^,^57^. Accordingly, in a meta-analysis of data collected from various literature sources Hillebrand and Azovsky^14^ found no LDG for protists compared to larger animals and plants. On the other hand, others^7^,^15^,^58^ reported pronounced LDGs at least for a specific evolutionary lineage of ciliates (marine planktonic tintinnids). Thus, the question whether planktonic ciliates exhibit LDGs or not is currently unresolved. Even though the data obtained from the circumglobal *Tara* Oceans voyage do not support an LDG for marine planktonic ciliates, this finding is not conclusive. Inference of LDGs from metadata obtained from various publications (such as in Hillebrand and Azovsky^14^) may be equally critical as using data from the *Tara* Oceans project: (i) Compiling (meta)data from inconsistent habitat types (e.g. habitats with strong variation of environmental variables such as pH or salinity), which may overlay and obscure spatial (latitudinal) patterns; (ii) non-uniform protocols to collect and process samples and different protocols to precisely quantify diversity may artificially introduce differences in sample sets from different studies; (iii) lumping together data collected from different seasons may make comparison of data from different studies or samples highly biased^59^. Therefore, testing LDGs in ciliate plankton solidly requires a well-designed sampling strategy. Until solid data from such a targeted sampling are available, LDGs for marine ciliate plankton will remain largely speculative and elusive (for an exception see Dolan *et al*.^58^).

Even though we found no evidence for ciliate LDGs in the *Tara* Oceans dataset, ciliate communities from the polar regions of the Southern Ocean differed notably in terms of richness and community composition from ciliate communities in all other investigated oceanic regions. Previous studies on individual ciliate morphotypes reported relatively low species richness in the Southern Ocean^60^, which is isolated from other oceans by the Polar Front. Associated with the Polar Front is the Antarctic Circumpolar Current, which is the fastest oceanic current in the world^61^. The Polar Front and the Antarctic Circumpolar Current represent a distinctive barrier for the exchange of water from north to south due to a significant change in temperature of the ocean surface water^61^. This barrier thus can hamper the dispersal of organisms in the southernmost part of the world’s oceans.

Other well-testable hypotheses in microbial biogeography address the extent and proportion of local versus global diversity (richness). Fenchel and colleagues^5^ exhaustively sampled ciliate morphospecies in the sediments of a lake (Priest Pot, United Kingdom) and a marine shallow-water bay (Nivå Bay, Denmark) and found that in these two limnic and marine sampling sites about 8% of all named free-living ciliates were present. One could argue that this number is an overestimation of the local proportion of global diversity, because numerous taxa escape microscopy detection for several reasons discussed in detail previously^62^. And if this is the case, the number of globally unseen ciliate morphospecies would most certainly be much higher than at individual sampling sites. However, also in a more sensitive molecular diversity survey, which also distinguishes cryptic species and finds very low abundant species, Santoferra *et al*.^63^ found at a single sampling site (Long Island Sound) over 5% of the global diversity of all extant Oligotrichia and Choreotrichia. In conclusion, these two studies support the idea that many ciliate species are widely distributed in space and time. However, like the microscopy approach, also the analysis presented in Santoferra *et al*.^63^ is not unbiased. The data obtained from Long Island Sound samples can only be compared to the molecular data available in public database (as a benchmark for the global diversity of Oligotrichea and Choreotrichea). But as shown recently, we are far away from having the full extent of marine ciliate diversity recorded and deposited in public databases. In first place, these databases comprise the genetic signatures of the most common, cultivable and morphological recognisable ciliate species. A different approach is to simply collect data (ciliate diversity) from the global oceans rather than relying on database entries (which do not cover as much diversity as found during the circumglobal *Tara* Oceans expedition^1^. Even if not all *Tara*-obtained ciliate OTUs can be assigned a species name, these OTUs can be compared among each other to assess the local diversity coverage of the global marine ciliate plankton diversity. Following this approach, it would be too stringent to require for an OTU to be present at every station to be considered as a cosmopolitan (even if sampling saturation profiles are close to saturation, individual low-abundant taxa can still escape detection in individual samples). Therefore, we defined that an OTU had to occur in at least one sample of each oceanic region to be considered as cosmopolitan. We found that 17% of all detected ciliate OTUs occurred in all oceanic regions under study and that, on average, local ciliate OTU richness represented 27% (±8% s.d.) of the global ciliate OTU richness. For all protistan taxon groups analyzed in the *Tara* Oceans dataset, the local richness covered, on average, 9.7% (±4% s.d.) of the global richness^1^. Thus, regardless of the taxonomic resolution (morphospecies or molecular OTUs), for open ocean samples it seems that global planktonic ciliate diversity is low (ca. 1,300 predicted ciliate OTUs for surface waters and the DCM together) with a relatively large proportion occurring in most local oceanic regions. We identified ciliates, which were assumed to have restricted geographic distribution based on microscopy observations, such as the tintinnid *Laackmanniella* (austral biogeography^15^) and the oligotrich ciliate *Spirotontonia* (northern hemisphere^12^) as cosmopolitans. The cosmopolitan ciliate genera showed high average abundances. Although number of reads do not reflect number of individual cells due to different SSU rDNA gene copy numbers^18^, the positive correlation between average abundance and spatial distribution indicates that cosmopolitan ciliates have higher local abundances than ciliates with a restricted distribution. At the same time, the abundances of most cosmopolitan ciliate OTUs were evenly distributed among different oceanic regions which may reflect organisms that are adapted to a broad range of environmental conditions. But the few cosmopolitan OTUs, which showed locally restricted maxima of abundances in single oceanic regions, indicate that some cosmopolitan ciliate taxa may switch from a rare to an abundant status and vice versa depending on their environmental surroundings. Similar findings are known from bacteria^64^ and match previous reports on ciliates^65^

However, our findings do not imply that global ciliate diversity in general is low as predicted earlier (only ca. 3000 free-living ciliate species^66^). Open ocean waters are merely one of many habitats for ciliates. For example, let alone ciliate OTU richness in coastal marine sediments may run up to several thousands, with little overlap to planktonic ciliates from the same sampling coordinates^20^. An additional several thousands of ciliates not found in the marine realm or freshwater habitats thrive in soils^8^. More species are added by specific habitats supporting rich and unique ciliate communities such as bromeliad tanks^67^ or extreme environments^17^,^18^,^55^. Thus, according to the tenet “the environment selects”^56^, we therefore suggest that global free-living ciliate diversity may indeed reach tens of thousands as predicted by Foissner *et al*.^11^ with open ocean waters however representing a low-diversity habitat.

## Additional Information

**How to cite this article**: Gimmler, A. *et al*. The *Tara* Oceans voyage reveals global diversity and distribution patterns of marine planktonic ciliates. *Sci. Rep.* **6**, 33555; doi: 10.1038/srep33555 (2016).

## Supplementary Material

Supplementary Information

Supplementary Information

## Figures and Tables

**Table 1:** Spearman’s rank correlation coefficients between ciliate OTU diversity (richness and exp(H)) and environmental parameters.

Habitat	Parameter	Richness		Exp(H)	
		r	p	r	p
a) SRF + DCM	Temperature (n = 77)	0.012	0.9142	0.128	0.2654
	Salinity (n = 75)	0.122	0.2979	0.077	0.5091
	Oxygen (n = 71)	−0.100	0.4081	−0.173	0.1487
	NO_2_^−^ (n = 74)	0.168	0.1524	0.122	0.2996
	NO_3_^−^ (n = 74)	−0.095	0.4223	−0.047	0.6882
	PO_4_^3−^ (n = 74)	−0.150	0.2028	−0.061	0.6081
	Si(OH)_4_ (n = 74)	−0.076	0.5172	−0.175	0.1366
	Chl *a* (n = 78)	0.367	0.0009	0.202	0.0761
	Latitude (SRF) (n = 47)	−0.027	0.8561	−0.086	0.5658
	Latitude (DCM)(n = 36)	−0.027	0.8762	−0.099	0.5643
b) SRF	pH (n = 23)	−0.018	0.9358	−0.340	0.1124
	Total alkalinity (n = 23)	−0.340	0.1130	−0.264	0.2229
	Total carbon (n = 23)	−0.326	0.1294	−0.323	0.1327
	CO_2_ (n = 23)	−0.011	0.9607	−0.155	0.4780
	pCO_2_ (n = 23)	−0.071	0.7486	0.302	0.1606
	HCO_3_ (n = 23)	−0.182	0.4063	−0.376	0.0775
	CO_3_ (n = 23)	−0.245	0.2596	0.079	0.7194

Correlations (r) were calculated for SRF and DCM data together (a) and concerning oceanic carbonate data only for SRF data (b). Number of samples is listed behind each parameter. P-values < 0.05 are considered as significant.

**Table 2:** Environmental factors fitted into NMDS analysis.

Habitat	Parameter	NMDS1	NMDS2	R^2^	Pr(>r)
a) SRF + DCM	Temperature	0.5741	0.8188	0.75	0.0001
	Salinity	0.9722	0.2342	0.11	0.0168
	Oxygen	−0.6602	−0.7511	0.63	0.0001
	NO_2_^−^	−0.3366	−0.9417	0.02	0.3243
	NO_3_^−^	−0.7845	−0.6202	0.44	0.0001
	PO_4_^3−^	−0.8309	−0.5564	0.40	0.0001
	Si(OH)_4_	−0.7332	−0.6801	0.40	0.0010
	Chl *a*	−0.7366	−0.6763	0.10	0.0522
b) SRF	pH	0.0002	−1.0000	0.00	0.9738
	Total alkalinity	0.0000	−1.0000	0.15	0.2071
	Total carbon	0.0000	−1.0000	0.04	0.6766
	CO_2_	0.0000	1.0000	0.01	0.9161
	pCO_2_	−0.0001	1.0000	0.01	0.9440
	HCO_3_	0.0003	−1.0000	0.02	0.8120
	CO_3_	−0.0001	−1.0000	0.13	0.2164

Columns NDMS1 and NMDS2 give direction cosines of the vectors and R^2^ gives the squared correlation coefficient. P-values for each factor are given in the last column and are considered as significant effect if P < 0.05.
