# Supplementary material for: The Tara Oceans voyage reveals global diversity and distribution patterns of marine planktonic ciliates
Source: Sci Rep. 2016 Sep 16;6:33555. doi: 10.1038/srep33555 (PMC5025661; doi:10.1038/srep33555)
Supplement: Supplementary Information [file srep33555-s1.pdf]

Supplementary Information S1: The *Tara* Oceans voyage reveals global diversity and distribution patterns of marine planktonic ciliates

Anna Gimmler[1], Ralf Korn[2,3], Colomban de Vargas[4,5], Stéphane Audic[4,5], Thorsten Stoeck[1]*

[1]University of Kaiserslautern, Ecology Group, D-67663 Kaiserslautern, Germany

[2]University of Kaiserslautern, Financial Mathematics Group, D-67663 Kaiserslautern, Germany

[3]Fraunhofer Institute for Industrial Mathematics ITWM, D-67663 Kaiserslautern, Germany

[4]CNRS, UMR 7144, Station Biologique de Roscoff, Place Georges Teissier, F-29680 Roscoff, France

[5]Sorbonne Universités, UPMC Univ Paris 06, UMR 7144, Station Biologique de Roscoff, Place Georges Teissier, F-29680 Roscoff, France

* correspondence:

email: stoeck@rhrk.uni-kl.de

phone: +49-631-2052502

fax: +49-631-2052496

station	environmental feature	oceanic region	longitude	latitude	salinity	temperature [°C]	oxygen [µmol/kg]	NO_2^- [µmol/l]	PO_4^{3-} [µmol/l]
004	surface water layer	Mediterranean Sea	-6.553	36.563	36.629607	20.336888	NA	NA	NA
004	deep chlorophyll maximum layer	Mediterranean Sea	-6.553	36.563	36.556443	16.240096	NA	NA	NA
007	surface water layer	Mediterranean Sea	1.948	37.031	37.4911	23.82415	NA	0	0.06
007	deep chlorophyll maximum layer	Mediterranean Sea	1.948	37.031	NA	NA	NA	0	0.01
009	surface water layer	Mediterranean Sea	5.82	39.112	37.8053	24.5021	NA	0.01	0.02
009	deep chlorophyll maximum layer	Mediterranean Sea	5.82	39.112	37.8232	16.143033	NA	0.02	0.02
011	surface water layer	Mediterranean Sea	2.798	41.666	NA	NA	NA	NA	NA
016	surface water layer	Mediterranean Sea	15.454	37.398	38.133342	20.659508	210.106333	0.02	0.04
016	deep chlorophyll maximum layer	Mediterranean Sea	15.454	37.398	38.262447	18.242387	220.830542	0.01	0.04
018	surface water layer	Mediterranean Sea	14.288	35.756	37.89245	21.4384	207.791417	0.018	0.026
018	deep chlorophyll maximum layer	Mediterranean Sea	14.288	35.756	37.89241	18.357443	237.8893	0.025	0.013
020	surface water layer	Mediterranean Sea	14.973	34.451	38.378467	21.471742	197.550333	0.01	0.01
022	surface water layer	Mediterranean Sea	17.4	39.729	37.759667	17.040533	220.927083	0.07	0.02
022	deep chlorophyll maximum layer	Mediterranean Sea	17.4	39.729	38.116167	17.251367	220.987333	0.02	0.01
023	surface water layer	Mediterranean Sea	17.729	42.176	38.187633	17.635625	220.000333	0.01	0.01
023	deep chlorophyll maximum layer	Mediterranean Sea	17.729	42.176	38.395417	15.696917	224.266917	0.01	0.01
024	surface water layer	Mediterranean Sea	17.956	42.457	38.114217	18.141667	218.536917	0.01	0.01
025	surface water layer	Mediterranean Sea	19.421	39.333	38.182917	18.341542	218.016833	0	0.01
025	deep chlorophyll maximum layer	Mediterranean Sea	19.421	39.333	38.475705	15.153159	229.547682	0.07	0.07
026	surface water layer	Mediterranean Sea	20.188	38.431	38.356667	19.16765	212.903417	0.01	0.01
030	surface water layer	Mediterranean Sea	32.789	33.929	39.425058	20.452725	207.567167	0	0
030	deep chlorophyll maximum layer	Mediterranean Sea	32.789	33.929	39.255952	18.966866	218.132538	0.01	0
031	surface water layer	Red Sea	34.819	27.151	39.981867	25.060908	188.763583	0.014	0.024
032	surface water layer	Red Sea	37.254	23.391	39.74725	25.823117	188.416583	0.014	0.018
032	deep chlorophyll maximum layer	Red Sea	37.254	23.391	40.196433	26.0308	179.968	0.01	0.025
033	surface water layer	Red Sea	38.218	22.057	38.937417	27.32825	182.812167	0	0.053
033	deep chlorophyll maximum layer	Red Sea	38.218	22.057	NA	NA	NA	NA	NA
034	surface water layer	Red Sea	39.884	18.445	38.646858	27.59735	184.061167	0.02	0.19
034	deep chlorophyll maximum layer	Red Sea	39.884	18.445	38.916583	27.575658	175.384083	0.27	0.09
036	surface water layer	North Indian Ocean	63.524	20.824	36.530058	25.5802	NA	0.05	0.37
036	deep chlorophyll maximum layer	North Indian Ocean	63.524	20.824	36.549267	25.370083	211.606583	0.51	0.51
038	surface water layer	North Indian Ocean	64.576	19.017	36.617917	26.23015	199.9355	0.01	0.32
038	deep chlorophyll maximum layer	North Indian Ocean	64.576	19.017	NA	25.488758	154.699917	0.7	0.42
039	surface water layer	North Indian Ocean	66.463	18.647	36.285367	26.816392	193.3535	0.01	0.23
039	deep chlorophyll maximum layer	North Indian Ocean	66.463	18.647	36.332317	26.806383	192.95875	0.02	0.26
041	surface water layer	North Indian Ocean	70.011	14.582	36.024883	29.08805	187.42925	0	0.14
041	deep chlorophyll maximum layer	North Indian Ocean	70.011	14.582	36.496162	27.104162	148.328125	0.07	0.34
042	surface water layer	North Indian Ocean	73.919	5.992	34.56155	29.978933	189.25475	0	0.08
042	deep chlorophyll maximum layer	North Indian Ocean	73.919	5.992	35.103505	27.704835	132.77885	0.15	0.34
044	surface water layer	North Indian Ocean	71.52	2.806	35.039842	30.692617	188.152333	0	0.11
045	surface water layer	North Indian Ocean	71.71	0.941	35.091133	30.495017	185.243833	0.02	1.94
048	surface water layer	South Indian Ocean	66.32	-9.408	34.181417	29.844617	187.598417	0.01	0.08
052	surface water layer	South Indian Ocean	53.508	-17.023	34.566667	27.867083	191.68	0	0.12
052	deep chlorophyll maximum layer	South Indian Ocean	53.508	-17.023	34.910755	24.87709	192.55925	0.03	0.18
064	surface water layer	South Indian Ocean	37.929	-29.508	35.334717	22.164483	210.00775	0	0.08
064	deep chlorophyll maximum layer	South Indian Ocean	37.929	-29.508	35.335545	22.237532	207.446227	0	0.08
065	surface water layer	South Indian Ocean	26.334	-35.226	35.44666	21.81569	207.0439	NA	NA
065	deep chlorophyll maximum layer	South Indian Ocean	26.334	-35.226	NA	21.809275	206.43725	NA	NA
066	surface water layer	South Atlantic Ocean	18.016	-34.905	35.323317	15.032708	238.916333	0.3	0.34
066	deep chlorophyll maximum layer	South Atlantic Ocean	18.016	-34.905	35.3315	15.01455	240.4135	0.26	0.37
067	surface water layer	South Atlantic Ocean	17.206	-32.292	34.845292	12.833708	249.351417	0.171	1.016
068	surface water layer	South Atlantic Ocean	4.62	-31.039	35.68685	16.83115	231.914833	0.25	0.229
068	deep chlorophyll maximum layer	South Atlantic Ocean	4.62	-31.039	35.687803	16.780772	231.683361	0.285	0.227
070	surface water layer	South Atlantic Ocean	-3.413	-20.229	36.376225	19.775817	215.650833	0.054	0.364
072	surface water layer	South Atlantic Ocean	-18.006	-8.691	36.42324	25.0249	199.1195	0.003	0.104
072	deep chlorophyll maximum layer	South Atlantic Ocean	-18.006	-8.691	36.603343	24.092317	194.428431	0.011	0.143
076	surface water layer	South Atlantic Ocean	-35.231	-21.029	37.101367	23.3484	206.194333	0.001	0.056
076	deep chlorophyll maximum layer	South Atlantic Ocean	-35.231	-21.029	36.724683	21.613933	203.814833	0.015	0.038
078	surface water layer	South Atlantic Ocean	-43.323	-30.158	36.32425	19.853083	221.47475	0	0
078	deep chlorophyll maximum layer	South Atlantic Ocean	-43.323	-30.158	36.27495	19.287683	217.140667	0.053	0
082	surface water layer	Southern Ocean	-58.012	-47.165	34.048642	7.321	305.012167	0.151	1.301
082	deep chlorophyll maximum layer	Southern Ocean	-58.012	-47.165	34.057594	6.971806	306.211529	0.135	1.416
084	surface water layer	Southern Ocean	-60.471	-60.395	33.71972	1.84374	338.2982	0.266	1.723
085	surface water layer	Southern Ocean	-49.503	-62.176	34.35388	0.67084	343.4373	0.107	2.111
085	deep chlorophyll maximum layer	Southern Ocean	-49.503	-62.176	34.319478	-0.784051	325.402987	0.06	2.309
098	surface water layer	South Pacific Ocean	-110.992	-26.261	36.402683	25.14755	200.538167	0	0.2
098	deep chlorophyll maximum layer	South Pacific Ocean	-110.992	-26.261	35.845669	20.137131	210.5781	0.02	0.1
100	surface water layer	South Pacific Ocean	-96.283	-13.162	35.835267	25.249967	200.233917	0.14	0.68
100	deep chlorophyll maximum layer	South Pacific Ocean	-96.283	-13.162	35.544655	20.63862	216.790338	0.16	0.78
102	surface water layer	South Pacific Ocean	-85.27	-5.218	34.748175	24.941942	205.992583	0.32	1
102	deep chlorophyll maximum layer	South Pacific Ocean	-85.27	-5.218	34.900284	19.557984	103.916711	1.2	1.86
109	surface water layer	South Pacific Ocean	-84.545	1.8	33.3676	27.6163	198.635833	0.04	0.27
109	deep chlorophyll maximum layer	South Pacific Ocean	-84.545	1.8	34.344808	26.525285	203.127654	0.11	0.5
111	surface water layer	South Pacific Ocean	-100.662	-16.932	35.97144	22.76754	208.8654	0.04	0.5
111	deep chlorophyll maximum layer	South Pacific Ocean	-100.662	-16.932	35.687249	19.864927	211.54555	0.2	0.44
122	surface water layer	South Pacific Ocean	-139.338	-8.969	35.3662	26.54279	186.2407	0.12	0.57
122	deep chlorophyll maximum layer	South Pacific Ocean	-139.338	-8.969	36.097575	24.67165	179.916875	0.1	0.59
123	surface water layer	South Pacific Ocean	-140.304	-8.878	35.359483	26.574917	189.758333	0.14	0.53
123	deep chlorophyll maximum layer	South Pacific Ocean	-140.304	-8.878	NA	NA	NA	NA	NA
124	surface water layer	South Pacific Ocean	-140.588	-8.999	35.396729	26.516693	190.724	0.16	0.63
124	deep chlorophyll maximum layer	South Pacific Ocean	-140.588	-8.999	NA	NA	NA	NA	NA
125	surface water layer	South Pacific Ocean	-142.61	-8.89	35.427733	26.778917	187.302833	0.19	0.56
125	deep chlorophyll maximum layer	South Pacific Ocean	-142.61	-8.89	NA	NA	NA	NA	NA

NO_3^- [µmol/l]	$Si(OH)_4$ [µmol/l]	Chl *a* [mg/m^3]	pH	Total alkalinity [umol/kg]	Total carbon [umol/kg]	CO2 [umol/kg]	pCO2 [uatm]	HCO3 [umol/kg]	CO3 [umol/kg]
NA	NA	0.098404	NA	NA	NA	NA	NA	NA	NA
NA	NA	0.880307	NA	NA	NA	NA	NA	NA	NA
0.03	0.52	0.0812	NA	NA	NA	NA	NA	NA	NA
0.03	0.5	0.553274	NA	NA	NA	NA	NA	NA	NA
0.03	0.75	-0.00745	NA	NA	NA	NA	NA	NA	NA
0.08	0.6	0.434367	NA	NA	NA	NA	NA	NA	NA
NA	NA	NA	NA	NA	NA	NA	NA	NA	NA
0.02	0.67	0.102723	NA	NA	NA	NA	NA	NA	NA
0.03	1.34	0.154014	NA	NA	NA	NA	NA	NA	NA
0.12	0.56	0.048898	NA	NA	NA	NA	NA	NA	NA
0.02	0.62	0.132494	NA	NA	NA	NA	NA	NA	NA
0.02	0.54	0.048936	NA	NA	NA	NA	NA	NA	NA
0.21	2.03	0.180911	NA	NA	NA	NA	NA	NA	NA
0	1.48	0.228614	NA	NA	NA	NA	NA	NA	NA
0.04	1.38	0.172965	NA	NA	NA	NA	NA	NA	NA
0.05	1.05	0.236208	NA	NA	NA	NA	NA	NA	NA
0.09	2.18	0.151466	NA	NA	NA	NA	NA	NA	NA
0.03	1.05	0.121845	8.137	2599.35	2250	11.461	344.759	1991.36	247.179
0.15	1.82	0.201016	NA	NA	NA	NA	NA	NA	NA
0.01	1.03	0.121845	8.126	2604.03	2253.6	11.585	355.129	1993.82	248.195
0.05	0.82	0.044622	NA	NA	NA	NA	NA	NA	NA
0.02	0.67	0.141478	NA	NA	NA	NA	NA	NA	NA
0.03	0.82	0.048898	8.087	2486.7	2099.7	10.15	367.834	1821.916	267.633
0	0.87	0	7.845	2462.1	2221.3	19.34	717.155	2025.859	176.102
0.01	0.85	0.224444	NA	NA	NA	NA	NA	NA	NA
0	1.15	0.163228	7.807	2455.35	2229.6	20.838	794.899	2040.889	167.873
NA	NA	NA	NA	NA	NA	NA	NA	NA	NA
0.03	3.91	0.186697	NA	NA	NA	NA	NA	NA	NA
0.64	1.35	0.181406	NA	NA	NA	NA	NA	NA	NA
0.13	1.2	0.129697	8.054	2392.47	2057.07	10.96	397.012	1810.586	235.525
2.08	1.79	0.353707	NA	NA	NA	NA	NA	NA	NA
0.06	1.18	0.161808	NA	NA	NA	NA	NA	NA	NA
1.86	1.63	0.604752	NA	NA	NA	NA	NA	NA	NA
0.03	1.44	0.099089	NA	NA	NA	NA	NA	NA	NA
0.09	1.28	0.17638	NA	NA	NA	NA	NA	NA	NA
0.09	1.47	0.020173	NA	NA	NA	NA	NA	NA	NA
0.49	1.38	0.47554	NA	NA	NA	NA	NA	NA	NA
0.03	2.21	0.006724	NA	NA	NA	NA	NA	NA	NA
1.39	3.2	0.388348	NA	NA	NA	NA	NA	NA	NA
0.05	2.31	0.004111	NA	NA	NA	NA	NA	NA	NA
28.64	15.14	0.026898	NA	NA	NA	NA	NA	NA	NA
0.06	2.12	0	NA	NA	NA	NA	NA	NA	NA
0	2.67	0.176565	7.612	2296.7	2184.4	33.462	1266.083	2050.987	99.951
0.47	3.22	0.394226	NA	NA	NA	NA	NA	NA	NA
0	1.77	0.162993	8.104	2318.27	2001.33	10.334	340.035	1770.109	220.887
0.02	1.75	0.214573	NA	NA	NA	NA	NA	NA	NA
NA	NA	0.215292	NA	NA	NA	NA	NA	NA	NA
NA	NA	0.272648	NA	NA	NA	NA	NA	NA	NA
3.34	2.74	0.250054	8.107	2329.27	2072	12.733	342.387	1878.852	180.415
3.23	2.47	0.424363	NA	NA	NA	NA	NA	NA	NA
7.091	13.88	1.551493	8.026	2319.83	2123.8	17.005	425.605	1964.878	141.917
1.303	2.601	0.201517	8.087	2347.5	2081	12.728	363.35	1880.916	187.357
1.08	2.436	0.42458	NA	NA	NA	NA	NA	NA	NA
0.986	1.362	0.324315	8.058	2394.6	2112.5	12.879	399.039	1900.241	199.38
0.018	0.868	0.050608	8.06	2395.9	2063.4	11.002	391.89	1818.711	233.687
0.044	1.319	0.263939	NA	NA	NA	NA	NA	NA	NA
0	0.814	0.033622	8.085	2441.8	2099.9	10.872	372.546	1849.043	239.985
0	0.712	0.150865	NA	NA	NA	NA	NA	NA	NA
0.017	0.481	0.053173	8.101	2390.37	2082.2	11.348	352.603	1855.008	215.843
0.104	0.319	0.226052	NA	NA	NA	NA	NA	NA	NA
18.15	1.824	0.310844	NA	NA	NA	NA	NA	NA	NA
19.07	3.078	1.01772	NA	NA	NA	NA	NA	NA	NA
25.3	16.55	0.109555	NA	NA	NA	NA	NA	NA	NA
28.5	79.52	0.065273	NA	NA	NA	NA	NA	NA	NA
31	81.82	0.540091	NA	NA	NA	NA	NA	NA	NA
0.05	0.38	0.013449	8.031	2405.1	2086.9	11.888	427.187	1849.937	225.075
0.05	0.35	0.176523	NA	NA	NA	NA	NA	NA	NA
6.2	1.2	0.287892	NA	NA	NA	NA	NA	NA	NA
5.7	1.9	0.366887	NA	NA	NA	NA	NA	NA	NA
12.6	5	0.238224	7.905	2297.07	2072.5	16.564	585.366	1892.003	163.934
24.4	10.5	0.728939	NA	NA	NA	NA	NA	NA	NA
0.9	1.7	0.28167	8.022	2206.87	1914.87	10.944	409.792	1698.697	205.228
4.6	3.2	0.737378	NA	NA	NA	NA	NA	NA	NA
2.68	1	0.203347	8.05	2384.87	2084.5	12.1	405.64	1860.032	212.368
0.93	0.9	0.378549	NA	NA	NA	NA	NA	NA	NA
5.46	2.19	0.166512	7.471	2305.97	2248.63	48.967	1801.446	2127.058	72.606
2.8	1.8	0.295808	NA	NA	NA	NA	NA	NA	NA
4.9	2.22	0.319103	8.012	2334.4	2030.85	11.921	439.082	1804.203	214.726
NA	NA	NA	NA	NA	NA	NA	NA	NA	NA
6.18	2.52	0.286662	7.871	2323.8	2100.4	17.6	646.697	1918.596	164.204
NA	NA	NA	NA	NA	NA	NA	NA	NA	NA
3.73	1.8	0.233673	8.015	2340.87	2032.93	11.794	436.549	1803.37	217.766
NA	NA	NA	NA	NA	NA	NA	NA	NA	NA
