# Supplementary material for: The Tara Oceans voyage reveals global diversity and distribution patterns of marine planktonic ciliates
Source: Sci Rep. 2016 Sep 16;6:33555. doi: 10.1038/srep33555 (PMC5025661; doi:10.1038/srep33555)
Supplement: Supplementary Information [file srep33555-s2.pdf]

Supplementary Information S2: The *Tara* Oceans voyage reveals global diversity and distribution patterns of marine planktonic ciliates

Anna Gimmler[1], Ralf Korn[2,3], Colomban de Vargas[4,5], Stéphane Audic[4,5], Thorsten Stoeck[1]*

[1]University of Kaiserslautern, Ecology Group, D-67663 Kaiserslautern, Germany

[2]University of Kaiserslautern, Financial Mathematics Group, D-67663 Kaiserslautern, Germany

[3]Fraunhofer Institute for Industrial Mathematics ITWM, D-67663 Kaiserslautern, Germany

[4]CNRS, UMR 7144, Station Biologique de Roscoff, Place Georges Teissier, F-29680 Roscoff, France

[5]Sorbonne Universités, UPMC Univ Paris 06, UMR 7144, Station Biologique de Roscoff, Place Georges Teissier, F-29680 Roscoff, France

* correspondence:

email: stoeck@rhrk.uni-kl.de

phone: +49-631-2052502

fax: +49-631-2052496

#OTU ID	representative sequence	total number of reads	4.SUR	4.DCM
1889	GTCGCTCCTACCGATTGAATGATCCG	4	0	0
595	GTCGCTCCTACCGATTTCGAGTACTTT	102	0	0
635	GTCGCTCCTACCGATTTCGAGTGCTTT	166	0	0
717	GTCGCTCCTACCGATTTCGAGTGCTTT	70	0	0
1334	GTCGCTCCTACCGATTGAGTGTTCTG	18	3	0
1599	GTCGCTCCTACCGATTTCGAGTGCTTT	12	0	0
2132	GTCGCTCCTACCGATTTCGAGTGCTCT	3	0	0
350	GTCGCTGCTACCGATTGAGTGATCCG	757	0	2
648	GTCGCTGCTACCGATTGAGTGGTCCG	248	0	16
334	GTCGCTCCTACCGATTTTGAGTGATCC	492	0	2
694	GTCGCTGCTACCGATTGAGTGGTCCG	73	0	0
1579	GTCGCTGCTACCGATTGGGTGTTCCG	11	0	0
1831	GTCGCTGCTACCGATTGAGTGGTCCG	7	0	0
583	GTCGCTGCTACCGATTGAGTGATCCG	107	0	0
911	GTCGCTCCTACCGATTGAGTGGTCCG	51	0	4
958	GTCGCTCCTACCGATTGAGTGATCCG	25	0	0
324	GTCGCTGCTACCGATTGAGTGGTCCG	2981	9	14
35	GTCGCTGCTACCGATTGAGTGGTCCG	21471	128	17
85	GTCGCTCCTACCGATTTTGAGTGGTCC	5465	4	0
439	GTCGCTCCTACCGATTTTGAGTGATCC	277	0	0
474	GTCGCTCCTACCGATTTTGAATGGTCC	183	0	0
914	GTCGCTCCTACCGATTTCGAGTGATTC	29	0	0
994	GTCGCTGCTACCGATTGAGTGGTCCG	44	0	0
1122	GTCGCTCCTACCGATTTTGAGTGATCC	16	0	0
1169	GTCGCTGCTACCGATTGAGTGATCCG	14	0	0
1467	GTCGCTCCTACCGATTTTGAGTGGTCC	7	0	0
1548	GTCGCTCCTACCGATTTTGAGTGGTCC	8	0	0
1681	GTCGCTCCTACCGATTTTGAGTGATCC	5	0	0
1767	GTCGCTCCTACCGATTTTGAGTGATCC	4	0	0
2008	GTCGCTGCTACCGATTGAGTGGTCCG	33	0	0
1362	GTCGCTACTACCGATTGAGTGGTCCG	51	0	0
1766	GTCGCTCCTACCGATTTTGAGTGATCC	4	0	0
299	GTCGCTGCTACCGATTGAGTGGTCCG	781	21	26
473	GTCGCTGCTACCGATTGAGTGATCCG	184	0	0
781	GTCGCTGCTACCGATTGAGTGATCCG	47	0	0
888	GTCGCTCCTACCGATTTGAGTGATCCC	32	0	0
4	GTCGCTGCTACCGATTTTGAGTGGTCC	176901	15	5
32	GTCGCTGCTACCGATTGAGTGATCAG	25263	13	0
37	GTCGCTGCTACCGATTTTGAGTGGTCC	13695	7	0
145	GTCGCTGCTACCGATTGAGTGGTCCG	2369	0	0
167	GTCGCTGCTACCGATTGAGAGATCCG	1672	0	0
169	GTCGCTGCTACCGATTGAGTGGTCCG	1576	3	5
178	GTCGCTGCTACCGATTGAGTGATCCG	1460	0	0
187	GTCGCTGCTACCGATTGAGTGATCCG	3575	1	0
199	GTCGCTGCTACCGATTGAGTGTTGAG	1342	2	27
287	GTCGCTGCTACCGATTGAGTGATCCG	718	0	0
296	GTCGCTGCTACCGATTGAGTGTTCCG	1334	5	29

342	GTCGCTGCTACCGATTGAGTGGTCCG	683	0	0
408	GTCGCTGCTACCGATTGAGTGATCCG	433	0	0
418	GTCGCTGCTACCGATTGAGTATTCCG[illegible]	348	0	0
428	GTCGCTGCTACCGATTGAGTGTTCCG[illegible]	986	2	15
459	GTCGCTGCTACCGATTGAGTGGTCCG	279	0	4
478	GTCGCTGCTACCGATTGAGTGTTCCG[illegible]	178	0	0
516	GTCGCTGCTACCGATTGAGTGTTCCG[illegible]	202	7	1
521	GTCGCTGCTACCGATTGAGTGATACG	334	0	0
543	GTCGCTGCTACCGATTTTGAGTGATC[illegible]	129	0	0
552	GTCGCTGCTACCGATTGAGTGATTAG	124	0	0
567	GTCGCTGCTACCGATTGAGTGTTCCG[illegible]	254	0	13
616	GTCGCTGCTACCGATTGAGTGATCCG	169	0	0
768	GTCGCTGCTACCGATTGAGTGTTCCG[illegible]	58	0	0
809	GTCGCTGCTACCGATTGAGTGTTCCG[illegible]	104	0	0
868	GTCGCTGCTACCGATTGAGTGTTCCG[illegible]	94	0	1
905	GTCGCTGCTACCGATTGAGTGATCCG	39	0	0
949	GTCGCTGCTACCGATTGAGTGATCCG	35	0	0
966	GTCGCTGCTACCGATTGAGTGTGCCG	39	0	0
991	GTCGCTGCTACCGATTTTGAGTGATT[illegible]	23	0	0
1015	GTCGCTGCTACCGATTGAGTGTTCCG[illegible]	25	0	0
1172	GTCGCTGCTACCGATTGAGTGTTCCG[illegible]	14	0	0
1259	GTCGCTCCTACCGATTGAGTGATCCG[illegible]	11	0	0
1355	GTCGCACCTACCGATTTTGAGTGGTC[illegible]	17	0	0
1415	GTCGCTACTACTGATTGGATGATCCG[illegible]	17	0	0
1421	GTTGCTGCTACCGATTGAGAGTTCCG[illegible]	8	0	0
1479	GTCGCTGCTACCGATTGAGTGATCCG	7	0	0
1560	GTCGCTGCTACCGATTGAGTGATACG	18	0	0
1667	GTCGCTGCTACCGATTGAGGGTTCCG	8	0	0
1949	GTCGCTGCTACCGATTGTGAGTGGTC	9	0	0
481	GTCGCTGCTACCGATTGAGTGATCCG	270	0	0
622	GTCGCTGCTACCGATTGAGTGATCCG	152	0	0
881	GTCGCTGCTACCGATTGAGTGATCCG	75	0	0
1144	GTCGCTGCTACCGATTGAGTGATCCG	15	0	0
1549	GTCGCTCCTACCGATTTTGAGTGGTC[illegible]	6	0	0
1645	GTCGCTCCTACCGATTGAGTGGTCCG[illegible]	5	0	0
1022	GTCGCTCCTACCGATTTTGAGTGATC[illegible]	21	0	0
1135	GTCGCTCCTACCGATTTTGAGTGATC[illegible]	41	0	0
1316	GTCGCTGCTACCGATTGAGTGGTCCG	10	0	0
1550	GTCGCTCCTACCGATTTTGAGTGGTC[illegible]	6	0	0
25	GTCGCTCCTACCGATCGAGTGTTTAG[illegible]	25804	2	0
26	GTCGCTCCTACCGATCGAGTGTTTAG[illegible]	18947	1653	115
39	GTCGCTACTACCGATTGAATGGTTTA[illegible]	13520	14	3
235	GTCGCTCCTACCGATCGAGTGATTAG[illegible]	1214	0	0
388	GTCGCTCCTACCGATCGAGTGTTTAG[illegible]	308	0	1
514	GTCGCTCCTACCGATCGAGTGATTAG[illegible]	149	0	0
715	GTCGCTCCTACCGATTTCGAGTGATT[illegible]	64	0	0
860	GTCGCTCCTACCGATCGAGTGTTTAG[illegible]	36	0	0
1072	GTCGCTCCTACCGATTGAGTGATCCG[illegible]	25	3	0

1525	GTCGCTCCTACCGATTTCGAGTGTCTA	7	0	0
1537	GTCGCTCCTACCGATCGAGTGATTAG	7	0	0
1538	GTCGCTCCTACCGATCGAGTGTTTAG	8	1	0
1695	GTCGCTCCTACCGATCGAGTGTTTAG	129	0	0
1739	GTCGCTCCTACCGATGAATGTTCCGA	5	0	0
13	GTCGCTCCTACCGATCGAGTGTTTAG	74486	7	8
531	GTCGCTCCTACCGATTTCGAGTGATC	464	0	0
977	GTCGTTACTACCGATTGGTGTGCAGG	25	0	0
1252	GTCGCTACTACCGATTGAGTGCCAGG	22	0	0
1455	GTCGCTACTACCGATTGAATGGTTTA	18	0	0
1526	GTCGCTCTTACCGATTGAGTAATTCTA	7	0	0
1742	GTCGCTGCTACCGATTGGATGGTTCT	5	0	0
1744	GTCGCTCCTACCGATCGAGTGTTTAG	5	0	0
850	GTCGCTCCTACCGATTTCGGGTACTCT	37	0	0
171	GTCGCTCCTACCGATTTTGAGTGATC	1528	0	0
200	GTCGCTCGTACCGATTTGAGTGATCC	1345	50	0
468	GTCGCTGCTACCGATTGAGTGTTCCG	199	1	5
681	GTCGCTCCTACCGATTTTGAGTGATC	141	0	0
786	GTCGCTCCTACCGATTGAGTGATCTG	59	1	0
214	GTCGCTCCTACCGATACCGGGTGATC	1712	889	16
1335	GTCGCTCCTACCGATACCGGGTGATC	10	0	0
1388	GTCGCTCCTACCGATTTCGGGTGATC	9	0	0
208	GTCGCTCCTACCGATTTCGAGTGATTA	1259	1	0
736	GTCGCTCCTACCGATTTCGTGTGATCA	324	0	1
1249	GTCGCTCCTACCGATTTCGAGTGATC	32	0	0
398	GTCGCTCCTACCGATTTCGAGTGGTC	551	0	0
448	GTCGCTCCTACCGATTTTGAGTGATC	218	0	1
530	GTCGCTCCTACCGATTTCGAGTGGTC	486	0	0
574	GTCGCTCCTACCGATTTTGAGTGATC	281	0	0
927	GTCGCTCCTACCGACTTGAGTGGTCC	28	0	0
1504	GTCGCTCCTACCGATTTCGGGTGATC	9	0	0
2147	GTCGCTCCTACCGATACCGGGTGATC	3	0	0
419	GTCGCTCCTACCGATTTCGAGTGACC	266	2	3
617	GTCGCTCCTACCGATTTCGAGTGATC	105	0	0
780	GTCGCTCCTACCGATTTCGAGTGATC	268	0	5
1155	GTCGCTCCTACCGATTGCGAGTGATC	300	0	0
288	GTCGCTCCTACCGATACCGGGTGATC	737	50	4
670	GTCGCTCCTACCGATTGGGTGAAATG	126	0	0
1438	GTCGCTCCTACCTTGTGTGAAATGGT	8	0	0
652	GTCGCTCCTACCGATTTCGAGTGATTA	121	0	0
413	GTCGCTCCTACCGATATAGGTGATCC	260	0	133
710	GTCGCTCCTACCGATTTCGAGTGATC	65	0	0
1006	GTCGCTCCTACCGATTTCGGGTACTTT	50	0	0
580	GTCGCTCCTACCGATACCGGGTGTTG	109	0	0
49	GTCGCTCCTACCGATACCGGGTGTTA	10846	0	4
365	GTCGCTCCTACCGATACCGGGTGATG	346	0	0
880	GTCGCTCCTACCGATTTCGAGTGTTG	65	0	0
1246	GTCGCTGCTACCGATACCGGGTGATG	12	0	0

725	GTCGCTCCTACCGATTGAGTGCTTTG	78	0	0
1884	GTCGCTCCTACCGATTTCGAGTGGTT	5	0	0
111	GTCGCTGCTACCGATTGGGTGATCCA	3418	87	47
497	GTCGCTCCTACCGATTTCGAGTGGTT	460	0	0
818	GTCGCTGCTACCGATACCGGGTGATG	60	0	0
1105	GTCGCTCCTACCGATTTCGAGTGCTTT	17	0	0
1654	GTCGCTGCTACCGATTGAGTGATCCA	5	0	0
2139	GTCGCTCCTACCGATTTCGAGTGGTT	5	0	0
1605	GTCGCTCCTACCGATTTCGAGTGGTC	8	0	0
327	GTCGCTCCTACCGATATCGGGTGATA	461	0	0
634	GTCGCTCCTACCGATTTCGGGTGATC	237	0	5
1887	GTCGCTCCTACCGATTTCGAGTGGTC	40	0	0
56	GTCGCTGCTACCGATTGAGTGATCCG	8868	6	26
104	GTCGCTGCTACCGATTTTGAGTGATT	3740	1	0
211	GTCGCTGCTACCGATGAGTGATCCGG	1380	11	89
320	GTCGCTGCTACCGATGAGTGATCCGG	470	0	0
374	GTCGCTGCTACCGATGAGTGATCCGG	379	0	7
386	GTCGCTGCTACCGATGAGTGATCCGG	390	5	4
522	GTCGCTGCTACCGATTTTGAGTGATT	139	0	0
788	GTCGCTGCTACCGATTGAGTGATCCG	85	0	0
979	GTCGCTTCTACCGATTGAGTGATCCG	124	3	0
1196	ATCGCTGCTACCGATTGAGTGATCCG	20	0	0
1269	GTCGCTGCTACCGATTGATTGATCCG	11	0	0
1313	GTCGCTGCTACCGATGAGTGATCCGG	20	0	5
1660	GTCGCTGCTACCGATTGAGTGATCCG	19	3	0
1822	GTCGCTGCTACCTATTGAGTGATCCG	4	0	0
2024	GTCGCTGTTACCGATTGAGTGATCCG	3	0	0
549	GTCGCTCCTACCGATTTTGAGTGATC	173	0	0
676	GTCGCTGCTACCGATTGAGTGATCCG	74	0	0
932	GTCGCTCCTACCGATTGAGTGGTCCG	39	0	0
660	GTCGCTCCTACCGATTTGAGTGATAC	82	0	0
346	GTCGCTGCTACCGATTGAGTGGTCCG	736	0	0
444	GTCGCTCCTACCGATTTCGAGTGATC	220	0	0
502	GTCGCTGCTACCGATTGAGTGTTCCG	153	0	22
926	GTCGCTGCTACCGATTGAGTGGTCCG	28	0	0
963	GTCGCTGCTACCGATTGAGTGATCCG	25	0	0
1463	GTCGCTCCTACCGATTTCGAGTGATC	7	0	0
812	GTCGCTCCTACCGATTGAGTGATCAG	42	0	0
354	GTCGCTGCTACCGATTTCGAGTGGTC	386	0	0
535	GTCGCTGCTACCGATTTCGAGTGGTC	175	2	0
1571	GTCGCTGCTACCGATTTCGAGTGGTC	6	0	0
1849	GTCGCTGCTACCGATTTCGAGTGTTC	4	0	0
20	GTCGCTGCTACCGATTTCGAGTGGTC	35767	22	1429
134	GTCGCTGCTACCGATTTCGAGTGGTC	3115	0	0
174	GTCGCTGCTACCGATTTCGAGTGGTC	2554	2	27
195	GTCGCTGCTACCGATTTCGAGTGGTT	1300	0	0
226	GTCGCTGCTACCGATTTCGAGTGGTC	1810	1	4
267	GTCGCTGCTACCGATTTCGAGTGGTC	758	0	0

435	GTCGCTGCTACCGATTTCGAGTGGTC(	236	0	0
559	GTCGCTGCTACCGATTTCGAGTGGTCT	161	0	0
565	GTCGCTGCTACCGATTTCGAGTGATC(	130	1	0
573	GTCGCTGCTACCGATTTCGAGTGTTC(	117	0	0
575	GTCGCTGCTACCGATTTCGAGTGGTC(	113	0	0
620	GTCGCTGCTACCGATTTCGAGTGATC(	93	0	0
682	GTCGCTGCTACCGATTTCGAGTGGTC(	85	0	0
698	GTCGCTGCTACCGATTTCGAGTGGTC(	68	0	0
766	GTCGCTGCTACCGATTTCGAGTGGTC(	52	0	0
870	GTCGCTGCTACCGATTTCGAGTGGTC(	35	0	0
997	GTCGCTGCTACCGATTTCGAGTGGTC(	34	0	0
1097	GTCGCTGCTACCGATTTCGAGTGGTC(	17	0	0
1237	GTCGCTGCTACCGATTTCGAGTGCTC(	12	0	0
1323	GTCGCTGCTACCGATTTCGAGTGGTC(	78	0	0
1423	GTCGCTGCTACCGATTTCGAGTGGTC(	14	0	0
1430	GTCGCTCCTACCGATTGAGTGATCCG(	8	0	0
1584	GTCGCTGCTACCGATTTCGAGTGGTC(	6	0	0
1588	GTCGCTGCTACCGATTTCGAGTGGTC(	16	0	0
2097	GTCGCTGCTACCGATTTCGAGTGGTCT	23	0	0
191	GTCGCTACTACCGATTTCGAGTGGTC(	1432	0	0
297	GTCGCTGCTACCGATTTCGAGTGGTC(	584	0	0
644	GTCGCTGCTACCGATTGAGTGTTCCG(	97	0	0
970	GTCGCTGCATCCGATTTCGAGTGGTC(	25	0	0
14	GTCGCTGCTACCGATTTCGAGTGGTC(	92238	24	32
194	GTCGCTGCTACCGATTTCGAGTGCTC(	1302	0	0
609	GTCGCTGCTACCGATTTCGAGTGGTC(	101	0	0
654	GTCGCTGCTACCGATTTCGAGTGGTC(	81	0	0
839	GTCACTGCTACCGATTTCGAGTGGTC(	297	2	1
1203	GTCGCTGCTACCGATTTCGAGTGTTC(	17	0	0
1276	GTCGCTACTACCGATTTCGAGTGGTC(	51	0	0
1393	GTCGCTGCTACCGATTTCGAGTGGTC(	9	0	0
1498	GTCGCTACTACCGATTGCGAGTGGTC	17	0	0
1693	GTCGCTCCTACCGATTGAGTGATCCG(	9	0	0
2069	GTCGCTGCTACCGATTTCGAGTGGTC(	9	0	0
2247	GTCGCTGCTACCGATTTCGAGTGGTC(	9	0	0
73	GTCGCTGCTACCGATTTCGAGTGGTC(	6856	0	0
107	GTCGCTGCTACCGATTTCGAGTGGTC(	9239	3	0
253	GTCGCTGCCACCGATTTCGAGTGGTC(	1973	1	0
277	GTCGCTGCTACCGATTTCGAGTGGTC(	882	0	0
331	GTCGCTGCTACCGATTTCGAGTGGTC(	986	0	0
355	GTCGCTGCTACCGATTTCGAGTGGTC(	383	0	0
359	GTCGCTGCTACCGATTTCGAGTGTTC(	683	0	0
361	GTCGCTGCTACCGATTTCGAGTGTTC(	365	0	0
392	GTCGCTGCTACCGATTTCGAGTGTTC(	298	0	0
485	GTCGCTGCTACCGATTTCGAGTGATC(	172	0	0
528	GTCGCTGCTACCGATTTCGAGTGTTC(	252	0	4
539	GTCGCTGCTACCGATTTCGAGTGGTC(	156	0	0
553	GTCGCTGCTACCGATTTCGAGTGGTC(	124	0	0

582	GTCGCTGCTACCGATTTCGAGTGGTC	133	0	0
586	GTCGCTGCTACCGATTTCGAGTGTTC	133	0	0
588	GTCGCTGCTACCGATTTCGAGTGGTC	149	0	0
604	GTCGCTGCTACCGATTTCGAGTGGTC	273	0	0
713	GTCGCTGCTACCGATTTCGAGTGTTC	64	0	0
769	GTCGCTGCTACCGATTTCGAGTGATC	51	0	0
777	GTCGCTGCTACCGATTTCGAGTTGTC	48	0	0
885	GTCGCTGCTACCGATTTCGAGTGGTC	63	0	0
1084	GTCGCTGCTACCGATTTCGAGTGGTC	44	0	0
1177	GTCGCTGCTACCGATTTCGAGTGGTC	49	0	0
1273	GTCGCTGCTACCGATTTCGAGTGGTC	11	0	0
1374	GTCGCTGCTACCGATTTCGAGTGGTC	27	0	0
1424	GTCGCTGCTACCGATTTCGAGTGTTC	8	0	0
1495	GTCGCTGCTACCGATTTCGAGTGGTC	7	0	0
1572	GTCGCTGCTACCGATTTCGAGTGTTC	10	0	0
1583	GTCGCTGCTACCGATTTCGAGTGGTC	6	0	0
1682	GTCGCTGCTACCGATTTCGAGTGGTC	12	0	0
799	GTCGCTCCTACCGATTTCGAGTTACT	44	0	0
1231	GTCGCTCCTACCGATTTCGAGTTACA	12	0	0
241	GTCGCTCCTACCGATTGAGTGGTTCG	956	0	0
703	GTCGCTCCTACCGATTGAGTGGTTCG	66	0	0
114	GTCGCTTGTAGTAACGAATGGTCTGG	3324	2	0
1669	GTCGCTGCTACCGATTGAGTGTTCCG	7	0	0
74	GTCGCTCCTACCGATTGAGTGATCCG	17779	0	38
380	GTCGCTCCTACCGATTGAGTGATCCG	326	0	0
697	GTCGCTCCTACCGATTGAGTGATCCG	239	0	0
1035	GTCGCTGCTACCGATTGAGTGATCCG	104	3	0
1142	GTCGCTGCTACCGATTGAGTGATCCG	20	0	0
1644	GTCGCTCCTACCGATTGAGTGATCCG	19	0	0
1806	GTCGCTCCTACCGATTGAGTGATCCG	10	0	0
1808	GTCGCTCCTACCGATTGAGTGATCCG	4	0	0
2048	GTCGCTCCTACCGATCGAGTGGTCCG	3	0	0
479	GTCGCTGCTACCGATTGAGTGTTCCG	223	0	0
512	GTCGCTGCTACCGATTGAGTGTTCCG	239	0	4
983	GTCGCTCCTACCGATCGAGTGGTCCG	37	0	0
1300	GTCGCTCCTACCGATTTCGAGTGGTC	10	0	0
684	GTCGCTATTACCGATTGGGTGTATAG	73	0	0
416	GTCGCTCCTACCGATTTCGAGTGCTCT	516	0	0
524	GTCGCTCCTACCGATTTCGAGTGTTCT	162	0	0
599	GTCGCTCCTACCGATTTCGAGTGCTAT	115	1	0
937	GTCGCTCCTACCGATTTCGAGTGCTCT	33	0	0
1004	GTCGCTCCTACCGATTTCGAGTGGTCT	32	0	3
1183	GTCGCTCCTACCGATTTCGAGTGTTAT	18	0	0
1330	GTCGCTCCTACCGATTTCGAGTGCTCT	14	0	0
1440	GTCGCTCCTACCGATTTCGAGTGCTCT	12	0	0
1709	GTCGCTCCTACCGATTTTGAGTGATCT	5	0	0
403	GTCGCTCCTACCGATTTCGAGTGAGC	276	0	0
726	GTCGCTCCTACCGATTTCGAGTGCTTT	64	0	0

849	GTCGCTCCTACCGATTTCGAGTGAGCT	85	0	0
1065	GTCGCTCCTACCGATTGAGTGTTGTGC	38	0	0
157	GTCGCTTTTACCGATTGAGTGCTCAGC	1830	0	0
47	GTCGCTTTTACCGATGAGTGTTCAGGT	10598	22	1
70	GTCGCTTTTACCGATTGAGTGTTCAGC	7492	5	5
251	GTCGCTTTTACCGATTGAGTGTTCAGC	921	0	0
28	GTCGCTGTTACCGATTGAGTGTTAAGC	18312	246	15
744	GTCGCTATTGCCGATTGAGTGCTCAGC	85	0	0
784	GTCGCTGTTACCGATTGAGTGTTAAGC	52	0	0
1210	GTCGCTATTACCGATTGAGTGTATAGC	13	0	0
1286	GTCGCTATTACCGATTGAGTGCTAAGC	11	0	0
7	GTCGCTGCTACCGATTGAGTGATCCG	277320	2027	1683
63	GTCGCTGCTACCGATTGAGTGATCCG	7666	8	94
69	GTCGCTGCTACCGATTGAGTGATCCG	11753	40	37
89	GTCGCTGCTACCGATTGAGTGATCCG	5757	6	38
90	GTCGCTGCTACCGATTGAGTGATCCG	10139	12	193
120	GTCGCTGCTACCGATTGAGTGATCCG	7028	19	59
129	GTCGCTGCTACCGATTGAGTGGTCCG	2728	6	35
142	GTCGCTGCTACCGATTGAGTGATCCAC	4602	0	4
146	GTCGCTGCTACCGATTGAGTGATCCG	2204	5	4
177	GTCGCTGCTACCGATTGAGTGGTCCG	1688	0	3
189	GTCGCTGCTACCGATTGAGTGGTCCG	2886	2	0
197	GTCGCTGCTACCGATTGAGAGGTCCG	2612	18	17
224	GTCGCTGCTACCGATTGAGTGGTCCG	1033	0	12
240	GTCGCTGCTACCGATTGAGAGGTCCG	1045	1	0
248	GTCGCTGCTACCGATTGAGAGATCCG	1113	0	0
254	GTCGCTGCTACCGATTGAGTTGTCCGC	910	0	0
266	GTCGCTGCTACCGATTGAGTGGTCCG	1066	0	0
268	GTCGCTGCTACCGATTGAGTGGTCCG	735	0	4
280	GTCGCTGCTACCGATTGAGTGTTCCGC	928	6	16
291	GTCGCTGCTACCGATTGAGTGATCCG	1025	0	5
301	GTCGCTGCTACCGATTGAGTGTTCCGC	538	0	50
303	GTCGCTGCTACCGATTTCGAGTGGTCC	571	0	0
318	GTCGCTGCTACCGATTTCGAGTGGTCC	503	1	23
339	GTCGCTGCTACCGATTGAGTGGTCCG	492	0	0
341	GTCGCTGCTACCGATTGAGTGATCCG	416	4	0
351	GTCGCTGCTACCGATTGAGTGATCCG	7012	109	34
360	GTCGCTGCTACCGATTGAGTGGTCAG	381	0	0
368	GTCGCTGCTACCGATTGAGTGTTCCGC	416	0	0
377	GTCGCTGCTACCGATTGAGTGATCCG	629	8	7
381	GTCGCTGCTACCGATTGAGTGGTCCG	391	0	0
409	GTCGCTGCTACCGATTGAGTGGTCCG	271	0	0
423	GTCGCTGCTACCGATTGAGTGGTCCG	813	1	0
443	GTCGCTGCTACCGATTGAGTGATCCG	396	0	0
445	GTCGCTGCTACCGATTGAGAGATCCG	222	0	0
462	GTCGCTGCTACCGATTGAGTGGTCCG	201	0	0
465	GTCGCTGCTACCGATTGAGTATTCCGC	286	0	0
482	GTCGCTGCTACCGATTGAGAGGTCCG	175	0	0

484	GTCGCTGCTACCGATTGAGTGGTCCG	265	0	0
496	GTCGCTGCTACCGATTGAGTGATCCG	384	0	2
498	GTCGCTGCTACCGATTGAGTGGTCCG	195	1	0
515	GTCGCTGCTACCGATTGAGTGGTCCG	145	1	0
566	GTCGCTGCTACCGATTGAGTGGTCCG	140	0	2
571	GTCGCTGCTACCGATTTCGAGTGTTC	115	0	0
585	GTCGCTGCTACCGATTGAGTGGTCCG	106	0	0
625	GTCGCTGCTACCGATTGAGTGATCCG	196	0	0
628	GTCGCTGCTACCGATTGAGTGATCCG	181	0	0
629	GTCGCTGCTACCGATTGAGTGTTCCG	91	0	0
643	GTCGCTGCTACCGATTGAGTGTTCAG	85	0	0
687	GTCGCTGCTACCGATTGAGTGGTCCG	72	0	0
695	GTCGCTGCTACCGATTGAGTGATCCG	80	0	0
712	GTCGCTGCTACCGATTGAGTGGTCCG	122	0	0
732	GTCGCTGCTACCGATTGAGTGGTCCG	94	0	11
742	GTCGCTGCTACCGATTGAGTATTCCG	166	0	0
782	GTCGCTGCTACCGATTGAGTGGTCCG	47	3	0
803	GTCGCTGCTACCGATTGAGTGATCCG	93	0	2
816	GTCGCGGCTACCGATTGAGTGATCCG	625	0	0
821	GTCGCTGCTACCGATTGAGTGGTCCG	45	0	0
822	GTCGCTGCTACCGATTGAGTGGTCCG	40	0	0
866	GTCGCTGCTACCGATTGAGTGATCCG	571	2	0
883	GTCGCTGCTACCGATTGAGTGGTCCG	33	0	0
935	GTCGCTGCTACCGATTGAGAGGTCCG	28	0	0
950	GTCGCTGCTACCGATTGAGTGTTCCG	34	0	0
962	GTCGCTGCTACCGATTGAGTGATCCG	80	1	0
965	GTCGCTGCTACCGATTGAGTGATCCG	25	0	0
993	GTCGCTGCTACCGATTTCGAGTGATT	23	0	0
995	GTCGCTGCTACCGATTGAGTGTTCCG	28	1	6
1016	GTCGCTGCTACCGATTGAGTGTTCCG	22	0	1
1024	GTCGCTGCTACCGATTGAGTGATCCG	218	0	0
1053	GTCGCTGCTACCGATTGTGTGATCCG	189	2	1
1054	GTCGCTGCTACCGATTGAGTGATCCG	230	1	0
1079	GTCGCTGCTACCGATTGAGTGATACG	349	5	1
1118	GTCGCTGCTACCGATTGAGTGGTCCG	77	0	0
1121	GTCGCTGCTACCGATGAGTGATCCGG	133	0	0
1170	GTCGCTTCTACCGATTGAGTGATCCG	221	3	1
1171	GTCGCTGCTACCGATTGAGTGGTCCG	14	0	0
1173	GTCGCTGCTACCGATTGAGTGTTCCG	18	0	0
1227	GTCGCTGCTACCGATTGAGTGATCCG	61	0	0
1228	GTCGCTGCTACCGATTGAGTGATCCG	40	0	0
1230	GTTGCTGCTACCGATTGAGTGATCCG	12	0	0
1308	GTCGCTGCTACCGATTGAGTGGTCCG	10	0	0
1314	GTCGCTGCTACCGATTAGTGATCCGG	34	0	1
1315	GTCGCTGCTACCGATTGAGTGGTCCG	20	0	0
1363	GTCGCTGCTACCGATTGAGTGTTCCG	14	0	0
1416	ATCGCTGCTACCGATTGAGTGATCTG	8	0	0
1417	GTCGCTGCTACCGATTGAGTGTTCCG	12	0	0

1481	GTCGCTGCTACCGATTGAGTGATCCG	73	0	0
1484	GTCGCTGCTACCGATTGAGTGATCGG	47	0	0
1490	GTCGCTGCTACCGATTGAGAGATCCG	7	0	0
1564	GTCGCACCTACCGATTGAATGGTCCG	18	0	0
1565	GTCGCGGCTACCGAGTGAGTGATCC	43	1	1
1569	GTCGCTGCTACCGATTGAGTGATCCG	19	0	0
1570	GTCGCTGCTACCGATTGAGTGTGACG	6	0	0
1573	GTCGCTGCTACCGATTGACTGATCCG	50	0	0
1659	GTCGCTGCTACCAATTGAGTGATCCG	9	2	0
1661	GTCGCTGCTACCGATTGAGTGATCCG	225	4	1
1663	GTCGCTGCTACCGATTGAGTGATCCG	7	0	0
1664	GTCGCTGCTACCGATTGAGTGATCCT	9	0	0
1665	GTCGCTGCTACCGATTGAGTGGTCCG	5	0	0
1674	GTCGCTGCTACCGATGAGTGATCCGG	20	0	0
1797	GTCGCTGCTACCGATTGAGTGATCCG	4	4	0
1798	GTCGCTGCTACCGATTGAGTGGTCCG	4	0	0
1812	GTCGCTGCTACAGATTGAGTGATCCG	8	0	0
1813	GTCGCTGCTACCGATTGAGTGATCCG	13	0	0
1815	GTCGCTGCTACCGATTGAGTGATCCG	12	1	0
1817	GTCGCTGCTACCGATTGAGTGGTCCG	8	0	0
1818	GTCGCTGCTACCGATTGAGTGGTCCG	5	0	0
1819	GTCGCTGCTACCGATTGAGTGTTCCG	4	0	0
1826	GTCGCTACTACCGATTGAATGATCCG	13	0	1
1836	GTCGCTGCTACCGATTAAGTGGTCCG	4	0	0
2014	GACGCTGCTACCGATTGAGTGATCCG	27	0	0
2020	GTCGCTGCTACCGATTGAGTGATCCG	6	0	0
2041	GTCGCTGCTACCGATGAGTGATCCGG	8	0	0
2078	GTCGCTACTACCGATCGAGTGGTCAG	7	0	0
2241	GTCGCTGCTACCGATTGAGAGGTCCG	11	0	0
236	GTCGCTGCTACCGATTGAGTGGTCCG	1210	2	16
306	GTCGCTGCTACCGATTGAGTGGTCCG	1140	7	149
356	GTCGCTGCTACCGATTGAGTGGTCCG	386	1	2
1080	GTCGCTACTACCGATTGAGTGGAACG	18	0	0
1666	GTCGCTGCTACCGATTGAGTGGTCCG	5	0	0
1820	GTCGCTGCTACCGATTGGGTGTTCCG	4	0	0
179	GTCGCTGCTACCGATTGAGTGGTCCG	2288	0	0
182	GTCGCTGCTACCGATTGAGTGGTCCG	3912	0	3
193	GTCGCTGCTACCGATTGAGTGGTCCG	3462	0	1
228	GTCGCTGCTACCGATTGGGTGCTCAG	1922	2	49
494	GTCGCTGCTACCGATTGAGTGGTCCG	388	0	4
1272	GTCGCTGCTACCGATTGAGTGATCCG	20	0	0
1369	GTCGCTGCTACCGATTGAGTGGTCCG	42	0	0
154	GTCGCTACTACCGATCGAGTGGTCAG	1913	3	0
907	GTCGCTACTACCGATTGAGTGGTCCG	36	0	0
952	GTCGCTACTACCGATCGAGTGGTCAG	49	0	0
1842	GTCGCTACTACCGATCGAGTGGTCAG	21	0	0
2018	GTCGCTGCTACCGATTGAGTGATCCG	3	0	0
274	GTCGCTCCTACCGATTTCGAGTGGTA	717	0	0

286	GTCGCTGCTACCGATTGAGTGGTCCG	1539	2	41
347	GTCGCTGCTACCGATTGAGTGTTCCG	588	1	0
475	GTCGCTCCTACCGATTTCGAGTGATTC	380	0	9
829	GTCGCTGCTACCGATTGAGTGGTCCG	527	0	1
906	GTCGCTGCTACCGATTGAGTGGTCCG	75	0	0
1816	GTCGCTGCTACCGATTGAGGGGTCCG	7	0	0
33	GTCGCTGCTACCGATTGAGTGATCCG	19942	81	1
188	GTCGCTGCTACCGATTGAGTGTTCCG	1472	10	5
205	GTCGCTGCTACCGATTGAGTGATCCG	5130	11	80
290	GTCGCTGCTACCGATTGAGTGGTCCG	1928	5	19
307	GTCGCTGCTACCGATTGAGTGTTCCG	724	0	0
387	GTCGCTGCTACCGATTGAGTGGTCCG	410	1	0
414	GTCGCTGCTACCGATTGAGTGGTCCG	282	0	0
677	GTCGCTCCTACCGATTTCGAGAGGTC	74	0	0
1056	GTCGCTGCTACCGATGAGTGATGCGG	19	0	0
1257	GTCGCTCCTACCGATTTCGAGTGATC	11	0	0
1568	GTCCCTGCTACCGATTGAGTGATCCG	12	0	1
1638	GTCGCTCCTACCGATTTCGAGTGATTC	5	0	0
80	GTCGCTGCTACCGATTGAGAGGTCCG	7174	4	0
476	GTCGCTGCTACCGATTGAGTGTTCCG	180	0	29
527	GTCGCTGCTACCGATTGAGTGGTCCG	137	0	0
590	GTCGCTGCTACCGATTGAGTATTCCG	296	0	0
669	GTCGCTGCTACCGATTGAGTGTTCCG	120	0	4
749	GTCGCTGCTACCGATTGAGTATTGCG	64	0	0
867	GTCGCTGCTACCGATTGAGTGCAAAG	35	0	0
1364	GTCGCTGCTACCGATTGAGTGTTGAG	9	0	0
510	GTCGCTCCTACCGATTTGAGTGATCC	334	0	0
533	GTCGCTCCTACCGATTTCGAGTGATC	134	0	0
954	GTCGCTCCTACCGATCGAGTGGTCCG	26	0	0
1001	GTCGCTCCTACCGATCGAGTGGTCCG	23	0	0
1373	GTCGCTCCTACCGATTTTGAGTGATC	9	0	0
1585	GTCGCTCCTACCGATTTCGAGTGATTC	6	0	0
330	GTCGCTCCTACCGATTTCGAGTGATC	472	0	0
383	GTCGCTCCTACCGATTTTGAGTGGTTT	319	0	0
457	GTCGCTCCTACCGATTTCGAGTGATC	237	0	1
1691	GTCGCTCCTACCGATTTCGAGTGATC	5	0	0
139	GTCGCTCCTACCGATTTCGAGTGTTC	2556	0	0
1038	GTCGCTCCTACCGATTTCGAGTGTTC	20	0	0
1394	GTCGCTTCTACCGATTCGATTTGTGTA	9	0	0
1582	GTCGCTCCTACCGATTTCGAGTGGTC	15	0	0
708	GTCGCTCCTACCGATTTCGAGTGATC	161	1	0
916	GTCGCTCCTACCGATTTCGAGTGATC	29	0	0
395	GTCGCTGCTACCGATTGAGTGTTCCG	590	4	16
472	GTCGCTGCTACCGATTGAGTGTTCCG	222	0	10
1036	GTCGCTGCTACCGATTGAGTGTTCCG	20	0	0
791	GTCGCTCCTACCGATCGAGTGGTCCG	46	0	0
1180	GTCGCTCATACCGATTTCGAGTCATTT	14	0	0
1320	GTCGCTCCTACCGATTTCGAGTGATC	17	0	0

294	GTCGCTCCTACCGATTTCGAGTGGTCC	1106	0	0
968	GTCGCTCCTACCGATTTCGAGTGGTCC	25	0	0
0	GTCGCTCCTACCGATTTCGAGTGGTCC	1228483	0	3
101	GTCGCTCCTACCGATTTCGAGTGGTCC	3986	0	0
805	GTCACATCATGAAAGCCGGTTTCGAG	43	0	0
1062	GTCGCTCCTACCGATTGAGTGATCCG	25	0	0
1101	GTCGCTCCTACCGATTGAGTGGTCCG	17	0	0
1590	GTCGCTCCTACCGATTGAGTGCTCAG	6	0	0
1757	GTCGCTACTACCGATTGAACGTTTAG	11	0	0
840	GTCGCTCCTACCGATCGAGTGGTCCG	38	0	0
500	GTCGCTCCTACCGATTTCGAGTGCTCT	390	0	0
534	GTCGCTCCTACCGATTTCGAATTATCC	143	3	5
683	GTCGCTCCTACCGATTTCGAATTATCC	73	0	0
1205	GTCGCTCCTACCGATCGAGTGGTCCG	13	0	0
1277	GTCGCTCCTACCGATTTCGAGTGGTCC	11	0	0
1503	GTCGCTACTACCGATTTCGAGTGGTCC	7	0	0
1864	GTCGCTCCTACCGATTTCGAGTGGTCC	4	0	0
2045	GTCGCTTCTACCGATTGAGAGGTCCG	5	0	0
638	GTCGCTCCTACCGATTTCGAGTGATTC	86	0	0
407	GTCGCTCCTACCGATTTCGAGTGATCC	282	0	0
1883	GTCGCACCTACCGATTGAATGGTCCG	4	0	0
163	GTCGCTCCTACCGATTGAGTGATCCG	1699	0	0
824	GTCGCTCCTACCGATTGAGTGATCCG	40	0	0
244	GTCGCTCCTACCGATCGAGTGGTCCG	938	0	7
412	GTCGCTCCTACCGATCGAGTGGTCCG	445	0	5
1098	GTCGCTCCTACCGATCGAGTGGTCCG	26	0	0
1426	GTCGCTCCTACCGATTTGAGTGGTTCC	8	0	0
18	GTCGCTCCTACCGATTTTGAGTGTTCC	40371	0	77
204	GTCGCTCCTACCGATTTTGAGTGATCC	1189	0	0
1026	GTCGCTCCTACCGATTTTGAGTGTTCC	106	0	0
1278	GTCGCTCCTACCGATTTTGAGTGATCC	11	0	0
138	GTCGCTCCTACCGATCGAGTGGTCCG	2322	0	3
846	GTCGCTCCTACCGATTGAGTGGTCCG	52	0	0
1029	GTCGCTCCTACCGATTGAGTGTTCCG	21	0	0
1148	GTCGCTCCTACCGATTGAGTGATCCG	15	0	0
371	GTCGCTCCTACCGATTGAGTGATCCG	338	0	0
778	GTCGCTCCTACCGATTGAGTGATCCG	64	0	0
1856	GTCGCTGCTACCGATTGGATGGTTCT	4	0	0
2085	GTCGCTCCTACCGATTGAATGATCCG	3	0	0
2092	GTCGTTACTACCGATTGGTGTGCAGG	3	0	0
132	GTCGCTCCTACCGATTGAGTGGTTCG	5592	135	64
311	GTCGCTCCTACCGATTGAGTGGGTCG	698	0	0
1427	GTCGCTACTACCGATCGAGTGGTCAG	28	0	0
15	GTCGCTCCTACCGATTTTGAGTGTTCC	75938	173	142
21	GTCGCTCCTACCGATTTTGAGTGATCC	36728	7	5
24	GTCGCTCCTACCGATTTTGAGTGATCC	32572	1303	31
45	GTCGCTCCTACCGATTTTGAGTGATCC	20472	15	34
67	GTCGCTCCTACCGATTTTGAGTGATCC	8995	812	19

149	GTCGCTCCTACCGATTTTGAGTGATCC	2165	0	0
184	GTCGCTCCTACCGATTTTGAGTGATCC	4496	157	33
217	GTCGCTCCTACCGATTTTGAGTGATCC	1098	0	10
256	GTCGCTCCTACCGATTTTGAGTGATCC	820	0	2
321	GTCGCTCCTACCGATTTTGAGTGATCC	767	0	0
322	GTCGCTCCTACCGATTTTGAGTGATCC	725	0	0
325	GTCGCTCCTACCGATTTTGAGTGATCC	460	2	0
328	GTCGCTCCTACCGATTTTGAGTGATCC	660	0	18
335	GTCGCTCCTACCGATTTTGAGTGATCC	823	0	0
343	GTCGCTCCTACCGATTTTGAGTGTTCC	1018	0	0
378	ATCGCTCCTACCGATTTTGAGTGATCC	695	1	4
385	GTCGCTCCTACCGATTTTGAGTGATCC	375	0	0
410	GTCGCTCCTACCGATTTTGAGTGATCC	503	0	0
415	GTCGCTCCTACCGATTTTGAGTGATCC	261	0	0
429	GTCGCTCCTACCGATTTGAGGGATCC	249	0	0
432	GTCGCTCCTACCGATTTTGAGTGATCC	286	2	0
450	GTCGCTCCTACCGATTTGAGTGATCCC	229	0	1
469	GTCGCTCCTACCGATTTTGAGTGATCC	1110	16	1
504	GTCGCTCCTACCGATTTTGAGTGATCC	154	0	0
513	GTCGCTCCTACCGATTTTGAGTGATCC	188	0	2
523	GTCGCTCCTACCGATTTTGAGTGATCC	138	0	0
551	GTCGCTCCTACCGATTTTGAGTGATCC	287	1	0
611	GTCGCTCCTACCGATTTTGAGTGATCC	97	0	0
655	GTCGCTCCTACCGATTTTGAGTGATCC	81	0	0
679	GTCGCTCCTACCGATTTTGAGTGATCC	74	0	0
707	GTCGCTCCTACCGATTTTGAGTGATCC	179	0	0
750	GTCGCTCCTACCGATTTTGAGTGATCC	105	0	0
754	GTCGCTCCTACCGATTTTGAGTGATCC	55	0	0
789	GTCGCTCCTACCGATTTTGAGTGGTCC	46	0	0
830	GTCGCTCCTACCGATTTTGAGTGATCC	39	0	0
837	GTCGCTCCTACCGATTTTGAGTGATCC	45	0	0
844	GTCGCTCCTACCGATTTTGAGTGATCC	37	0	0
845	GTCGCTCCTACCGATTTGAGTGATCCC	51	0	0
879	GTCGCTCCTACCGATTTTGAGTGATCC	48	0	0
889	GTCGCTCCTACCGATTTTGAGTGATCC	32	0	0
917	GTCGCTCCTACCGATTTTGAGTGATCC	97	0	0
928	GTCGCTCCTACCGATTTTGAGTGATCC	43	0	4
982	GTCGCTCCTACCGATTGAGTGATCCG	44	7	0
996	GTCGCTCCTACCGATTTTGAGTGATCC	23	0	0
1000	GTCGCTCCTACCGATTTGAGTGATCCC	23	0	0
1058	GTCGCTCCTACCGATTTTGAGTGATCC	19	0	0
1095	GTCGCTCCTACCGATTTTGAGTGATCC	39	0	0
1096	GTCGCTCCTACCGATTTTGAGTGATCC	27	0	0
1145	GTCGCTCCTACCGATTTTGAGTGATCC	15	0	0
1176	GTCGCTCCTACCGATTTTGAGTGATCC	14	0	0
1202	GTCGCTCCTACCGATTTTGAGTGATCC	15	0	0
1234	GTCGCTCCTACCGATTTTGAGTGATCC	21	0	0
1321	GTCGCTCCTACCGATTTTGAGTGATCC	10	0	0

1493	GTCGCTCCTACCGATTTTGGGTGATC(	24	0	0
1684	GTCGCTCCTACCGATTTTGAGTGATC(	9	3	0
1685	GTCGCTCCTACCGATTTTGAGTGATTA	5	0	0
1686	GTCGCTCCTACCGATTTTGAGTGGTC(	8	0	0
75	GTCGCTCCTACCGATTGAGTGATCCG(	9222	1	0
242	GTCGCTCCTACCGATTTTGAGTGATC(	1196	1	0
333	GTCGCTCCTACCGATTGAGTGATCCG(	432	0	0
50	GTCGCTCCTACCGATTGAGTGATCCG(	15335	33	30
1807	GTCGCTGCTACCGATTGAGTGATCCG	85	0	0
764	GTCGCTCCTACCGATTCGAGTGCCTC(	56	2	0
890	GTCGCTCCTACCGATTGAGTGGTTGG	43	0	0
119	GCCGCTTCTACCGATTCGAGTGGTTC(	3069	0	0
326	GCCGCTTCTACCGATTCGAGTGGTTC(	468	0	0
1885	GCCGCTTCTACCGATTCGAGTGGTTC(	4	0	0
143	GCCGCTCCCATCGATTGAGTGAGTCG	2350	0	0
36	GTCGCTCCTACCGATTGAGTGATTCG(	13297	0	0
125	GTCGCTCCTACCGATTGAGTGATTCG(	2817	0	0
207	GTCGCTCCTACCGATTGAGTGATTCG(	1173	0	0
623	GTCGCTCCTACCGATTGAGTGATTCG(	92	0	0
661	GTCGCTCCTACCGATTGAGTGATTCG(	174	0	0
743	GTCGCTCCTACCGATTCGAGTGGTTC(	58	0	0
770	GTCGCTCCTACCGATTGAGTGATTCG(	63	0	1
847	GTCGCTCCTACCGATTGAGTGATTCG(	41	0	0
1241	GTCGCTCCTACCGATTGAGTGATTCG(	12	0	0
1501	GTCGCTCCTACCGATTGAGTGATTCG(	7	0	0
1512	GCCGCTTCTACCGATTGAGTGGTTCG(	7	0	0
1862	GTCGCTCCTACCGATTCGAGTGATTC(	4	0	0
1870	GTCGCTCCTACCGATTTCGAGTGGTC(	4	0	0
1933	GTCGCTCCTACCGATTTTGAGTTATTA	3	0	0
168	GTCGCTGCTACCGATTGGGTGTTCCG(	1770	0	7
310	GTCGCTGCTACCGATTGGGTGTTCCG(	527	0	10
317	GTCGCTCCTACCGATTGAGGGATTCG	467	1	16
390	GTCGCTCCTACCGATTGAGGGATTCG	304	0	4
564	GTCGCTCCTACCGATTGAGGGATTCG	118	0	0
858	GTCGCTCCTACCGATCGAGTTGGTAT(	47	0	0
293	GTCGCTCCTACCGATTCGAGTGATCC(	1166	0	1
64	GTCGCTCCTACCGATCGAGTGATCCG(	8453	16	27
135	GTCGCTCCTACCGATTGAATGGTTCG(	2944	0	32
159	GTCGCTCCTACCGATTGAGGGATTCG	1832	0	224
1060	GTCGCTCCTACCGATCGAGTGGTCCG	19	0	0
1671	GTCGCTCCTACCGATCGAGTGATCCG(	14	0	0
1810	GTCGCACCTACCGATTGAATGGTCCG	7	0	0
115	GTCGCTCCTACCGATTGAATGATTCG(	3249	0	0
173	GTCGCTCCTACCGATTGAATGGTTCG(	1450	0	41
357	GTCGCTCCTACCGATTGAATGGTTCG(	382	0	0
541	GTCGCTCCTACCGATTGAATGGTTCG(	140	0	0
653	GTCGCTCCTACCGATTTTGAGTGCTGT	139	0	0
855	GTCGCTCCTACCGATTTTGAGTGCTGT	79	0	0

947	GTCGCTCCTACCGATTGAATGGTTCG(	26	0	0
992	GTCGCTCCTACCGATTTCGAGTGATC(	23	0	0
1081	GTCGCTCCTACCGATTGAATGGTTCG(	18	0	0
1554	GTCGCTCCTACCGATTGAATGGTTCG(	6	0	0
1823	GTCGCTACTACCGATTGAATGGCTTA(	4	0	0
980	GTCGCTGCTACCGATTGAGTGATCCG	24	0	0
532	GTCGCTGCTACCGATTGAGTGATCCG	323	0	0
1119	GTCGCTGCTACCGATTGAGTGTTCCG(	23	0	0
1352	GTCGCTCCTACCGATTTTGAGTGATC(	11	0	0
71	GTCGCTCCTACCGATTTCGAGTGGTC(	6476	0	0
315	GTCGCTCCTACCGATTTCGAGTGGTC(	479	3	0
696	GTCGCTCCTACCGATTTCGAGTGGTC(	69	0	0
11	GTCGCTAGTACCGATTTCGAGTGGTC(	86458	0	13
19	GTCGCTAGTACCGATTTCGAGTGGTC(	35723	0	1
82	GTCGCTCCTACCGATTTCGAGTGATC(	6327	19	11
192	GTCGCTCCTACCGATTTCGAGTGGTC(	1301	0	11
206	GTCGCTCCTACCGATTTCGAGTGGTC(	1193	0	0
223	GTCGCTCCTACCGATTTCGAGTGGTC(	1050	0	0
255	GTCGCTCCTACCGATTTCGAGTGGTT(	1244	0	15
257	GTCGCTCCTACCGATTTCGAGTGGTC(	803	0	0
278	GTCGCTAGTACCGATTTCGAGTGGTC(	694	1	48
302	GTCGCTCCTACCGATTTCGAGTGGTC(	523	0	0
308	GTCGCTCCTACCGATTTCGAGTGATC(	654	2	13
363	GTCGCTCCTACCGATTTCGAGTGGTC(	349	0	15
364	GTCGCTCCTACCGATTTCGAGTGGTT(	354	0	57
384	GTCGCTCCTACCGATTTCGAGTGGTC(	494	0	0
451	GTCGCTCCTACCGATTTCGAGTGGTC(	601	9	0
520	GTCGCTCCTACCGATTTCGAGTGGTC(	209	0	16
526	GTCGCTCCTACCGATTTCGAGTGGTC(	165	0	0
594	GTCGCTCCTACCGATTTCGAGTGGTC(	200	0	2
610	GTCGCTCCTACCGATTTCGAGTGATC(	149	0	0
787	GTCGCTCCTACCGATTTCGAGTGATC(	64	0	0
878	GTCGCTAGTACCGATTTCGAATGGTC(	113	0	0
948	GTCGCTCCTACCGATTTCGAGTGATT(	29	0	0
961	GTCGCTAGTACCGATTTCGAGTGGTC(	63	0	0
1116	GTCGCTCCTACCGATTTCGAGTGATC(	25	0	0
1178	GTCGCTCCTACCGATTTCGAGTGGTC(	14	0	0
1224	GTCGCTCCTACCGATTTCGAGTGGTC(	12	0	0
1370	GTCGCTGCTACCGATTGGATGGTTCT(	9	0	0
1408	GTCGCTACTACCGATTTCGAGTGATC(	48	0	0
1411	GTCGCTCCTACCGATTTCGAGTGGTC(	8	0	0
1486	GTCGCTCCTACCGATTGAATGATCCG(	7	0	0
1559	GTCGCTCCTACCGATTTCGCGTGGTCT	6	0	0
1575	GTCGCTCGTACCGATTTCGAGTGGTC(	15	0	0
1672	GTCGCTCCTACCGATTGAGTGATCCG(	5	0	0
1801	GTCGCTAGTACCGATTGCGAGTGGTC	7	0	0
1802	GTCGCTAGTACCGATTTCGAGTGGTC(	4	0	0
1828	GTCGCACCTACCGATTGAATGGTCCG	4	0	0

1977	GTCGCTCCTACCGATTTCGAATGGTC(	3	0	0
81	GTCGCTCCTACCGATTTCGAGTGGTC(	16476	22	93
153	GTCGCTCCTACCGATTTCGGGTGATT(	4485	20	6
218	GTCGCTCCTACCGATTTCGGGTGATT(	2703	114	3
300	GTCGCTCCTACCGATTTCGAGTGGTC(	561	0	18
313	GTCGCTCCTACCGATTGAGTGGTTTG(	658	0	0
394	GTCGCTCCTACCGATTTCGGGTGATT(	627	5	1
405	GTCGCTCCTACCGATTTCGAGTGGTC(	276	0	0
441	GTCGCTCCTACCGATTTCGAGTGGTC(	224	0	0
453	GTCGCTCCTACCGATTTCGGGTGATT(	978	0	0
471	GTCGCTCCTACCGATTTCGAGTGGTC(	185	0	0
477	GTCGCTCCTACCGATTTCGGGTGATT(	276	0	17
507	GTCGCTCCTACCGATTTCGGGTGATT(	149	4	0
562	GTCGCTCCTACCGATTTCGAGTGATT(	209	0	0
728	GTCGCTCCTACCGATTTCGAGTGGTC(	222	0	0
755	GTCGCTCCTACCGATTTCGAGTGATC(	68	0	0
756	GTCGCTCCTACCGATTTCGAGTGATC(	56	0	3
757	GTCGCTCCTACCGATTTCGGGTGATT(	157	0	20
762	GTCGCTCCTACCGATTTCGAGTGGTC(	53	0	2
771	GTCGCTCCTACCGATTTCGGGTGATT(	100	0	0
835	GTCGCTCCTACCGATTTCGAGTGGTC(	226	0	2
843	GTCGCTCCTACCGATTTCGAGTGATC(	41	0	0
856	GTCGCTCCTACCGATTTCGAGTGGTC(	39	0	0
1032	GTCGCTCCTACCGATTTCGAGTGGTC(	33	0	2
1318	GTCGCTCCTACCGATTTCGGGTGATT(	10	0	0
1356	GTCGCTCCTACCGATTTCGAGTGATC(	9	0	0
1372	GTCGCTCCTACCGATTTCGGGTGATT(	9	0	0
1401	GTCGCTCCTACCGATTTCGAGTGGTC(	8	0	0
1403	GTCGCTCCTACCGATTTCGGGTGATT(	8	0	0
1558	GTCGCTCCTACCGATTTCGAGTGGTT(	6	0	0
1581	GTCGCTCCTACCGATTTCGGGTGATT(	44	1	0
1787	GTCGCTCCTACCGATTTCGAGTGATC(	4	0	0
2	CGTCGCTCCTACCGATTTCGAGTGAT(	378699	0	94
6	GTCGCTCCTACCGATTTCGAGTGATC(	147720	35	98
40	GTCGCTCCTACCGATTTCGAGTGATC(	12962	58	96
78	GTCGCTCCTACCGATTTCGAGTGGTC(	6224	0	63
99	GTCGCTCCTACCGATTTCGAGTGGTC(	4177	7	12
198	GTCGCTCCTACCGATTTCGAGTGGTC(	1370	1	0
216	GTCGCTCCTACCGATTTCGAGTGATC(	2597	13	17
222	GTCGCTCCTACCGATTTCGAGTGATC(	3360	0	13
231	GTCGCTCCTACCGATTTCGAGTGGTC(	1088	0	0
246	GTCGCTCCTACCGATTTCGAGTGGTC(	2245	11	4
258	GTCGCTCCTACCGATTTCGAGTGATC(	824	0	0
279	GTCGCTCCTACCGATTTCGAGTGATC(	1012	2	0
340	GTCGCTCCTACCGATTTCGAGTGGTC(	426	1	0
352	GTCGCTCCTACCGATTTCGAGTGGTC(	705	59	0
659	GTCGCTCCTACCGATTTCGAGTGGTC(	153	0	0
864	GTCGCTCCTACCGATTTCGAGTGATC(	120	0	0

1034	GTCGCTCCTACCGATTTCGAGTGGTC(	33	0	0
1093	GTCGCACCTACCGATTGAATGGTCCG	17	0	0
1117	GTCGCTCCTACCGATTTCGAGTGGTC(	16	0	0
1167	GTCGCTCCTACCGATTTCGAGTGATC(	14	0	0
1195	GTTGCTCCTACAAATTTTGAGTGATC(	13	0	0
1226	GTCGCTCCTACCGATTTCGAGTGATTT	12	0	0
1232	GTCGCTCCTACTGATTGAGTGGTCCG(	21	0	0
1270	GTCGCACCTACCGATTGAATGGTCCG	11	0	0
1407	GTCGCTCCTACCGATTTCGAGTGATC(	36	0	0
1409	GTCGCTCCTACCGATTTCGAGTGATC(	63	0	0
1472	GTCGCTCCTACCGATTGCGAGTGATC(	49	0	0
1474	GTCGCTACTACCGATTTCGAGTGGTC(	46	0	0
1475	GTCGCTCCTACCGATTTCGAGTGGTC(	83	0	0
1477	GTCGCTCCTACCGATTTCGAGTGGTC(	7	0	0
1649	GTCGCTCCTACCGATTTCGAGTGGTC(	15	0	0
1803	GTCGCTCCGACCGATTTCGAGTGGTC(	6	0	0
1979	GTCGCTCCTACCGATTTCGAGTGATC(	3	0	0
2004	GTCGCTAGTACCGATTTCGAGTGGTC(	3	0	0
915	GTCGCTCCTACCGATTTCGAGTGATC(	83	0	0
1354	GTCGCTCCTACCGATTTCGAGTGATC(	9	0	0
1555	GTCGCTCCTACCGATTGCGAGTGATC(	31	0	0
1969	GTCGCTCCTACCGATTGCGAGTGATC(	18	0	0
401	GTCGCTCCTACCGATTTCGAGTGGTC(	280	0	0
452	GTCGCTCCTACCGATTTCGAGTGGTC(	218	4	0
554	GTCGCTCCTACCGATTTCGAGTGGTC(	206	0	0
912	GTCGCTCCTACCGATTTCGAGTGGTC(	29	0	0
1112	GTCGCTCCTACCGATTTCGAGTGGTC(	30	0	0
1298	GTCGCTCCTACCGATTTCGAGTGGTC(	17	0	0
1351	GTCGCTCCTACCGATTTCGAGTGGTC(	15	0	0
1264	GTCGCTCCTACCGATTGCGAGTGGTC(	746	0	29
828	GTCGCTCCTACCGATTTCGAGTGGTC(	195	0	3
1261	GTCGCTCCTACCGATTGCGAGTGGTC(	63	0	8
1366	GTCGCTCCTACCGATTTAGAGTGGTC(	248	0	2
1476	GTCGCTCCTACCGATTTCGAGTGGTC(	255	0	2
1651	GTCGCTCCTACCGATTTCGAGTGGTC(	28	0	2
1788	GTCGCTCCTACCGATTGCGAGTGGTC(	10	0	1
1791	GTCGCTCCTACCGATTTCGAGTGGTC(	7	0	0
1796	GCCGCTCCTACCGATTTCGAGTGGTC(	12	0	1
1967	GTCGCTCCTACCGATGTCGAGTGGTC(	9	0	0
1983	GTCGCTCCTACCGATTTCGAGTGGTC(	33	0	1
51	GTCGCTACTTCCGATTTCGGGTGATT(	9744	0	0
271	GTCGCTACTACCGATTTCGAGTGGTC(	721	0	15
425	GTCGCTACTTCCGATTTCGAGTGGTC(	249	0	0
632	GTCGCTCCTACCGATTTCGAGTGGTC(	205	0	7
686	GTCGCTACTACCGATTTCGAGTGGTC(	92	0	0
1544	GTCGCTACTACCGATTTCGAGTGGTT(	6	0	0
1999	GTCGCTTCTACCGATTTCGAGTGGTC(	6	0	0
2006	GTCGCTCCGACCGATTTCGAGTGGTC(	7	0	0

3	GTCGCTCCTACCGATTTCGAGTGGTC(	357722	38	14446
93	GTCGCTACTACCGATTTCGAGTGGTC(	5259	3	103
124	GTCGCTCCTACCGATTGAATGGTTCG(	2837	0	1
210	GTCGCTCCTACCGATTTCGAGTGGTC(	1171	0	0
461	GTCGCTCCTACCGATTTCGAGTGGTC(	711	0	2
493	GTCGCTACTACCGATTTCGAGTGGTC(	243	0	0
1302	GTCGCTCCTACCGATTTCGAGTGATC(	13	0	0
1631	GTCGCTACTACCGATTGAACGTTTTA(	11	0	0
1646	GTCGCTCCTACCGATTTCGAGTGGTC(	36	0	2
1781	GTCGCTCCTACCGATTGCGAGTGGTC(	28	0	3
1782	GTCGCTCCTACCGATTGCGAGTGGTC(	11	0	0
1974	GTCGCTCCTACCGATTTAGAGTGGTC(	18	0	1
436	GTCGCTACTACCGATTTCGAGTGGTC(	6059	0	185
109	GTCGCTCCTACCGATTTCGAGTGGTC(	3485	0	0
57	GTCGCTATTTCCGATTTCGAGTGGTC(	8377	6	2
1023	GTCGCTCCTACCGATTTCGAGTGGTC(	21	0	0
1256	GTCGCTACTACCGATTTCGAGTGGTC(	11	0	0
1304	GTCGCTCCTACCGATTTCGAGTGGTC(	10	1	0
1410	GTCGCTCCTACCGATTTCGAGTGGTC(	8	0	0
130	GTCGCTATTTCCGATTTCGAGTGGTC(	4723	2	0
619	GTCGCTCCTACCGATTGCGAGTGGTC(	451	0	0
752	GTCGCTCCTACCGATTTCGAGTGGTC(	55	0	0
1656	GTCGCTATTTCCGATTTCGAGTGGTC(	31	0	0
1972	GTCGCTCCTACCGATTGCGAGTGGTC(	45	0	0
46	GTCGCTCCTACCGATTTCGAGTGGTC(	19368	69	76
857	GTCGCTACTACCGATTTCGAGTGGTC(	36	0	0
1367	GTCGCTCCTACCGATTGAGTGATCCG(	9	0	0
1	GTCGCTCCTACCGATTTCGAGTGGTC(	458880	4870	32
54	GTCGCTCCTACCGATTTCGAGTGGTC(	8830	0	29
79	GTCGCTCCTACCGATTTCGAGTGGTC(	17395	14	284
110	GTCGCTCCTACCGATTTCGAGTGGTC(	4868	2	22
113	GTCGCTCCTACCGATTTCGAGTGGTC(	3820	1	14
147	GTCGCTCCTACCGATTTCGAGTGGTC(	4881	2	5
220	GTCGCTCCTACCGATTTCGAGTGGTC(	1173	0	0
245	GTCGCTCCTACCGATTTCGAGTGGTC(	2238	95	30
261	GTCGCTCCTACCGATTTCGAGTGGTC(	1024	0	2
336	GTCGCTCCTACCGATTTCGAGTGGTC(	886	0	0
466	GTCGCTCCTACCGATTTCGAGTGGTC(	197	0	14
613	GTCGCTCCTACCGATTTCGAGTGATC(	281	4	0
720	GTCGCTCCTACCGATTTCGAGTGGTC(	62	0	0
902	GTCGCTCCTACCGATTTCGAGTGGTC(	30	0	0
1650	GTCGCTCCTACCGATTTCGAGTGGTC(	5	0	0
1780	GTCGCTCCTACCGATTGCGAGTGGGC	12	0	0
30	GTCGCTCCTACCGATTTCGAGTGGTC(	19186	387	0
58	GTCGCTCCTACCGATTTCGAGTGGTC(	16335	61	0
219	GTCGCTCCTACCGATTTCGAGTGGTC(	1245	4	6
264	GTCGCTCCTACCGATTTCGAGTGGTC(	744	0	0
281	GTCGCTCCTACCGATTGAGTGGTTCG(	682	0	0

1136	GTCGCTCCTACCGATTTCGAGTGGTC(	28	0	0
1199	GTCGCTCCTACCGATTTCGAGTGGTC(	61	10	0
1262	GTCGCTCCTACCGATTTCGAGTGGTC(	116	19	0
1358	GTCGCTCCTACCGATTTCGAGTGGTC(	185	43	0
1632	GTCGCTACTACCGATTGAACGTTTTA(	20	8	0
1653	GTCGCTCCTAGCGATTTCGAGTGGTC(	5	0	0
2046	GTCGCTACTACCGATTGAACGTTTTA(	6	1	0
292	GTCGCTCCTACCGATTTCGAGTGGTC(	1062	160	0
624	GTCGCTCCTACCGATTTCGAGTGGTC(	161	3	0
96	GTCGCTCCTACCGATTTCGAGTGGTC(	7831	2	0
433	GTCGCTACTACCGATTTCGAGTGGTC(	241	0	0
508	GTCGCTACTACCGATTTCGAGTGGTC(	285	0	1
959	GTCGCTACTACCGATTTCGAGTGGTC(	42	0	0
1222	GTCGCTCCTACCGATTTCGAGTGGTC(	70	0	0
1639	GTCGCTCCTACCGATTTCGAGTGGTC(	11	0	0
8	GTCGCTACTACCGATTGAACGTTTTA(	135653	984	679
23	GTCGCTACTACCGATTTCGAGTGGTC(	26890	46	2636
86	GTCGCTACTACCGATTTCGAGTGGTC(	6337	16	0
589	GTCGCTACTTCCGATTTCGGGTGATT(	131	0	0
823	ATCGCTACTACCGATTTGATGTGGTC(	40	0	0
934	GTCGCTACTACCGATTTCGAGTGGTC(	228	0	6
1225	GTCGCTACTACCGATTTCGAGTGGTC(	42	1	0
1271	GTCGCTACTACCGATTCCGAGTGGTC(	38	1	0
1305	GTCGCTACTACCGATTTCGAGTGGTC(	29	0	0
1310	GTCGCTACTACCGATTTCGAGTGGTC(	759	3	18
1414	GTCGCTACTACCGATTTCGAGTGGTC(	412	7	0
1679	GTCGCTACTACCGATTGCGAGTGGTC	98	0	4
1833	GTCGCACCTACCGATTGAGTGTTCCG(	7	0	0
83	GTCGCTCCTACCGATTTCGGGTGGTC(	6252	93	240
88	GTCGCTCCTACCGATTTCGGGTGGTC(	5045	109	672
103	GTCGCTCCTACCGATTTCGGGTGGTC(	7859	73	1
112	GTCGCTCCTACCGATTTCGGGTGGTC(	3481	1	3
164	GTCGCTCCTACCGATTTCGGGTGGTC(	3404	11	16
489	GTCGCTCCTACCGATTTCGGGTGGTC(	166	0	0
739	GTCGCTCCTACCGATTTCGGGTGGTC(	58	0	0
794	GTCGCTCCTACCGATTTCGGGTGGTC(	45	0	0
1466	GTCGCTCCTACCGATTTCGGGTGGCC(	12	0	0
1764	GTCGCTCCTACCGATTTCGGGTGGCC(	17	0	0
12	GTCGCTCCTACCGATTTCGAGTGGTC(	144110	1340	22436
65	GTCGCTCCTACCGATTTCGAGTGGTC(	14780	147	2676
66	GTCGCTCCTACCGATTTCGAGTGGTC(	13273	0	2238
91	GTCGCTCCTACCGATTTCGAGTGGTC(	4915	0	1
128	GTCGCTCCTACCGATTTCGAGTGCTC(	3676	9	254
561	GTCGCTACTACCGATTTCGAGTGGTC(	120	0	0
615	GTCGCTCCTACCGATTTCGAGTGGTC(	95	0	0
776	GTCGCTCCTACCGATTTCGAGTGGTC(	150	1	17
1074	GTCGCTCCTACCGATTTCGAGTGGTC(	133	0	1
1165	GTCGCTCCTACCGATTTCGAGTGGTC(	52	0	31

1166	GTCGCTCCTACCGATTGAGTGATCCGC	25	0	11
1223	GTCGCTCCTACCGATTTCGAGTGGTCC	184	0	0
1404	GTCGCTGCTAACGATTTGGAGTGGTC	8	0	0
1545	GTCGCTCCTACCGATTTCGAGTGGTCC	19	0	0
1546	GTCGCTCCTACCGATTTCGAGTGGTCC	20	0	5
1641	GTAACTCCTACCGATTTCAAGTGGTCC	5	0	0
1761	GTCGCTCCTACCGATTTCGAGTGGTCC	32	0	8
1762	GTCGCTCCTACCGATTTCGAGTGGTCC	43	1	6
1769	GTTGCTCCTACCGATTTTGAGTGGTCC	4	0	0
1771	GTCGCTCCTACCGATTTCGAGTGGTCC	53	1	12
1943	GTCGCTCCTACCGATTGCGAGTGGTC	12	0	3
247	GTCGCTCCTACCGATTTCGAGTGGTCC	903	0	0
499	GTCGCTCCTACCGATTTCGAGTGGTCC	158	0	0
1013	GTCGCTCCTACCGATTTCGAGTGGTCC	38	0	0
180	GTCGCTCCTACCGATTTCGAGTGGTCC	1398	212	138
186	GTCGCTCCTACCGATTTCGAGTGGTCC	1371	2	0
229	GTCGCTCCTACCGATTTCGAGTGGTCC	1004	2	43
269	GTCGCTCCTACCGATTTCGAGTGGTCC	757	0	0
319	GTCGCTCCTACCGATTTCGAGTGGTCC	1082	0	1
338	GTCGCTCCTACCGATTGAGTGGTTCGC	425	0	0
597	GTCGCTCCTACCGATTTCGAGTGGTCC	101	0	0
658	GTCGCTCCTACCGATTTCGAGTGGTTC	79	0	0
738	GTCGCTCCTACCGATTTCGAGTGGTCC	58	0	0
945	GTCGCTCCTACCGATTTCGAGTGGTTC	26	0	0
1162	GTCGCTCCTACCGATTTCGAGTGGTCC	14	0	0
17	GTCGCTCCTACCGATTTCGAGTGGTCC	42110	5	153
53	GTCGCTCCTACCGATTTCGAGTGGTCC	9024	40	107
426	GTCGCTCCTACCGATTTCGAGTGGTCC	374	0	0
587	GTCGCTCCTACCGATTGAGTGGTTCGC	213	0	0
810	GTCGCTCCTACCGATTTCGAGTGGTCC	93	0	0
123	GTCGCTCCTACCGATTTCGAGTGGTCA	6365	0	6
238	GTCGCTCCTACCAATTTCGAGTGGCTC	1947	0	1
689	GTCGCTCCTACCAATTTCGAGCGGCTC	75	0	0
841	GTCGCTCCTACCGATTTCGAGTGATCA	38	0	0
1005	GTCGCTCCTACCGATTTCGAGTGGTTA	48	0	0
1747	GTCGCTCCTACCAATTTCGAGCGGCTC	4	0	0
1886	GTCGCTCCTACCGATTTCGAGTGGTCA	4	0	0
2106	GTCGCTACTACCAATTTCGAGTGGCTT	3	0	0
938	GTCGCTACTACCGATTGAACGTTTTAC	27	0	0
1125	GTCGCTCCTACCGATTGAGTGACCCGA	17	0	0
1711	GTCGCTACTACCGATTGAACGTTTTAC	10	0	0
1861	GTCGCTCCTACCAATAGGGTGACTTGC	4	0	0
72	GTCGCTCCTACCGATTTCGAGTGATCC	6743	0	44
106	GTCGCTCCTACCGATTTCGAGTGATCC	4702	1	0
323	GTCGCTCCTACCGATTTCGGGTGATTC	455	120	2
896	GTCGCTCCTACCGATTTCGAGTGATCC	31	0	0
924	GTCGCTCCTACCGATTTCGAGTGATCC	28	0	0
1175	GTCGCTCCTACCAATTTCGAGTGGCTC	23	0	0

1580	GTCGCTCCTACCAATTTCGAGTGGCTC	6	0	0
2050	GTCGCTCCTACCGATTGAGTGGTTCG	3	0	0
16	GTCGCTACTACCGATCGAGTGGTCAG	82356	71	153
27	GTCGCTACTACCGATTTCGAGTGGTC	31935	28	7807
31	GTCGCTACTACCGATCGAGTGGTCAG	23102	38	225
61	GTCGCTACTACCGATTGAGTGGTCAG	10706	0	2
68	GTCGCTACTACCGATTGAGTGGTCAG	8467	48	103
77	GTCGCTACTACCGATTGAGTGGTCAG	14710	39	40
92	GTCGCTACTACCGATTGAGTGGTCAG	5206	15	4
108	GTCGCTATTTCCGATTTCGAGTGGTCC	4047	500	5
117	GTCGCTACTACCGATTTCGAGTGGTC	3321	0	0
131	GTCGCTACTACCGATTGAGTGATCCG	3779	1	11
150	GTCGCTACTACCGATCGAGTGGTCAG	4063	0	1
162	GTCGCTACTACCGATCGAGTGGTCAG	3142	0	0
166	GTCGCTACTACCGATCGAGTGGTCAG	9212	15	52
172	GTCGCTACTACCGATTGAGTGGTCAG	2246	0	26
175	GTCGCTACTACCGATTGAGTGGTCAG	1575	0	0
183	GTCGCTACTACCGATCGAGTGGTCAG	2290	5	23
209	GTCGCTACTACCGATCGAGTGGTCAG	4785	21	0
213	GTCGCTACTACCGATTGAGTGGCCAG	1120	0	9
230	GTCGCTACTACCGATTGAGTGGTCAG	1002	0	1
233	GTCGCTACTACCGATCGAGTGGCTAG	1068	0	0
249	GTCGCTACTACCGATCGAGTGGTCAG	2006	0	87
250	GTCGCTACTACCGATCGAGTGGTCAG	1556	0	0
273	GTCGCTACTACCGATCGAGTGGTCAG	710	0	0
282	GTCGCTACTACCGATCGAGTGGTCAG	2919	5	0
289	GTCGCTACTACCGATCGAGTGGTCAG	1200	0	0
295	GTCGCTACTACCGATTGAGTGGTCAG	588	0	0
305	GTCGCTACTACCGATTGAGTGGTCAG	525	0	0
344	GTCGCTACTACCGATCGAGTGGTCAG	486	1	294
353	GTCGCTACTACCGATCGAGTGGTCAG	388	0	0
389	GTCGCTACTACCGATTGAGTGGTCAG	430	0	0
440	GTCGCTACTACCGATCGAGTGGTCAG	223	0	0
447	GTCGCTACTACCGATTGAGTGGTCAG	493	0	1
483	GTCGCTACTACCGATTGAGTGGTCAG	494	7	26
486	GTCGCTACTACCGATTGAGTGGTCAG	201	0	0
547	GTCGCTACTACCGATCGAGTGGTCAG	325	0	0
569	GTCGCTACTACCGATCGAATGGTCAG	116	0	0
576	GTCGCTACTACCGATCGAGTGGTCAG	113	0	0
591	GTCGCTACTACCGATTGAGTGGTCAG	103	0	0
630	GTCGCTACTACCGATTGAGTGGTCAG	92	0	0
631	GTCGCTACTACCGATTGAGTGGTCAG	88	0	0
646	GTCGCTACTACCGATCGAGTGGTCAG	176	0	1
668	GTCGCTACTACCGATTGAGTGGTCAG	77	0	0
706	GTCGCTACTACCGATTGAGTGGTCAG	65	0	0
721	GTCGCTACTACCGATTGAGTGGTCAG	155	1	0
724	GTCGCTACTACCGATCGAGTGGTCAG	61	0	0
733	GTCGCTACTACCGATCGAGTGGTCAG	59	0	0

737	GTCGCTATTTCCGATTTCGAGTGGTC(	58	0	0
753	GTCGCTACTACCGATTGAATGGTCAG	68	0	2
758	GTCGCTACTACCGATCGAGTGGTCAG	132	0	0
796	GTCGCTACTACCGATCGAGTGGTCAG	64	0	0
804	GTCGCTACTACCGATTGAGTGGTCAG	43	0	0
817	GTCGCTACTACCGATCGAGTGGTCAG	54	0	0
838	GTCGCTACTACCGATGAATGGTCAGG	38	0	0
884	GTCGCTACTACCGATCGAGTGGTCAG	33	0	0
919	GTCGCTACTACCGATCGAGTGGCCAG	189	1	1
964	GTCGCTACTACCGATCGAATGGTCAG	29	0	0
967	GTCGCTACTACCGATTGAATGGTCAG	25	0	0
990	GTCGCTACTACCGATTGAGTAGGTAG	23	0	0
1025	GTCGCTACTACCGATTGAGTGGTCAG	39	0	0
1057	GTCGCTACTACCGATCGAGTGGTCAG	36	0	0
1085	GTCGCTACTACCGATCGAGTGATCCG	23	0	0
1094	GTCGCTACTACCGATCGAGTGATCAG	32	0	0
1114	GTCGCTACTACCGATCGAGTGGTCAG	40	0	0
1120	GTCGCTACTACCGATTGAGTGGTCAG	32	0	0
1201	GTCGCTACTACCGATCGAATGGTCAG	24	0	0
1235	GTCGCTACTACCGATCGAGTGGTCAG	12	0	0
1236	GTCGCTACTACCGATCGAGTGGTCAG	14	0	0
1312	GTCGCTACTACCGATCGAATGGTCAG	10	0	0
1317	GTCGCTACTACCGATCGAGTGATCAG	15	0	0
1378	GTCGCTACTACCGATCGAGTGATCAG	17	0	0
1380	GTCGCTACTACCGATCGAGTGTTAAG	9	0	0
1384	GTCGCTACTACCGATCGAGTGGTCAG	15	0	0
1418	GTCGCTACTACCGATTGAGTGGTCAG	14	0	0
1429	GTCGCTACTACCGATCGAGTGGTCAG	15	0	0
1492	GTCGCTACTACCGATCGAGTGGTCAG	24	0	1
1553	GTCGCTACTACCGATTGAATGGTTCA(	12	1	0
1566	GTCGCTACTACCGATCGAATGGTCAG	6	0	0
1680	GTCGCTACTACCGATCGAGTGGTCAG	5	0	0
1683	GTCGCTACTACCGATCGAGTGATCTG(	5	0	0
1768	GTCGCTACTACCGATGTCGAGTGGTC.	4	0	0
1843	GTCGCTACTACCGATTGAATGGTCAG	26	0	1
1848	GTCGCTACTACCGATCGAGTGGTCAG	7	0	0
1953	GTCGCTACTACCGATTGGGTGATCAG	3	0	0
2062	GTCGCGAATACCGAGCGAGTGGTCA(	3	0	0
2067	GTCACTACTACCGATCGAGTGGTCAG	9	0	0
2068	GTCGCTACTACCGATCGAGTGGTCAG	11	0	0
2070	GTCGCTACTACCGATTGAGTGGTCAG	23	0	0
2073	GTCGCTACTACCGATTGAATGGCTAA(	5	0	0
2082	GTCGCTACTACCGATCGAGCGGTCAG	6	0	0
2083	GTCGCTACTACCGATCGAGTGGTAAG	3	0	0
41	GTCGCTCCTACCGATTTCGAGTGGTC(	18311	71	15
201	GTCGCTCCTACCGATTTCGAGTGGTC(	1873	0	18
456	GTCGCTCCTACCGATCGAGTGATCCG.	207	0	0
831	GTCGCTCCTACCGATTGAGTGATCCG(	71	2	0

887	GTCGCTCCTACCGATTTCGAGTGGTC	32	0	0
1133	GTCGCTCCTACCGATTGAGTGATCCG	15	0	6
1973	GTCGCTCCTACCGATTTCGAGTGGCC	6	0	0
165	GTCGCTCCTACCGATTTCGAGTGGTC	2445	0	15
458	GTCGCTCCTACCGATTTCGAGTGGTC	216	0	0
505	GTCGCTCCTACCGATTTGAGTGGTCC	149	1	0
802	GTCGCTCCTACCGATTTCGAGTGGTC	43	0	0
1078	GTCGCTCCTACCGATTTCGAGTGGTC	18	0	0
5	GTCGCTCCTACCGATTTCGAGTGGTC	162306	16	5172
9	GTCGCTCCTACCGATTTCGAGTGGTC	418737	942	15610
29	GTCGCTACTACCGATTTCGAGTGGTC	24891	1	339
34	GTCGCTACTACCGATTTCGAGTGGTC	15056	54	0
48	GTCGCTCCTACCGATTTCGAGTGGTC	21748	109	1037
76	GTCGCTCCTACCGATTTCGAGTGGTC	6692	34	128
97	GTCGCTCCTACCGATTTCGAGTGGTC	4400	0	153
122	GTCGCTCCTACCGATTTCGAGTGGTC	3867	5	99
152	GTCGCTACTACCGATTTCGAGTGGTC	2267	4	3
212	GTCGCTCCTACCGATTTCGAGTGGTC	1423	3	34
221	GTCGCTCCTACCGATTTCGAGTGGTC	1074	0	14
237	GTCGCTCCTACCGATTTCGAGTGGTC	1320	0	45
262	GTCGCTCCTACCGATTTCGAGTGGTC	1463	36	75
362	GTCGCTCCTACCGATTTCGAGTGGTC	979	2	75
379	GTCGCTCCTACCGATTTCGAGTGGTC	541	0	3
417	GTCGCTCCTACCGATTTCGAGTGGTC	262	166	3
540	GTCGCTCCTACCGATTTCGAGTGGTC	268	0	0
557	GTCGCTCCTACCGATTTCGAGTGGTC	484	0	2
601	GTCGCTCCTACCGATTTCGAGTGGTC	182	1	10
627	GTCGCTCCTACCGATTTCGAGTGGTC	1622	2	52
636	GTCGCTCCTACCGATTGCGAGTGGTC	1005	0	89
663	GTCGCTCCTACCGATTTCGAGTGGTC	78	0	1
667	GTCGCTCCTACCGATTTCGAGTGGTC	216	1	2
671	GTCGCTCCTACCGATTTCGAGTGGTC	112	5	0
699	GTCGCTCCTACCGATTTCGAGTGGTC	569	2	29
793	GTCGCTCCTACCGATTTCGAGTGGTC	211	1	7
795	GTCGCTCCTACCGACTTGAGTGGTCC	45	0	0
814	GTCGCTCCTACCGATTTCGAGTGGTC	41	0	0
826	GTCGCTCCTACCGATTTCGAGTGGTC	131	0	1
832	GTCGCTCCTACCGATTTCGAGTGGTC	230	0	24
833	GTCGCTCCTACCGATTTCGAGTGGTC	48	0	8
834	GTCGCTCCTACCGATTTCGAGTGGTC	98	0	5
895	GTCGCTCCTACCGATTTCGAGTGGTC	39	0	0
903	GTCGCTCCTACCGATTTCGAGTGGTC	44	9	0
946	GTCGCTCCTACCGATTTCGAGTGGTC	64	0	0
978	GTCGCTCCTACCGATTTCGAGTGGTC	24	7	0
1021	GTCGCTCCTACCGATTTCGAGTGGTC	85	0	1
1033	GTCGCTCCTACCGATTTCGAGTGGTC	26	0	0
1050	GTCGCTCCTACCGATTTCGAGTGGTC	109	0	2
1073	GTCGCTCCTACCGATTGAGTGGTCCG	18	0	0

1075	GTCGCTCCTACCGATTTCGAGTGGTC(	18	0	0
1091	GTCGCTCCTACCGATTTCGAGTGGTC(	1118	6	37
1134	GTCGCTCCTACCGATTTCGAGTGGTC(	37	0	0
1137	GTCGCTCCTACCGATTTCGATTGGTCC	23	0	0
1140	GTCGCTCCTACCGATTTCGAGTGGTC(	15	0	0
1163	GTCGCTCCTACCGATTTCGAGTGGTC(	187	0	23
1164	ATCGCTCCTACCGATTTCAAGTGGTCT	17	0	0
1191	GTCGCTCCTACCGATTTCGAGTGGTC(	1155	6	43
1194	GTCGCTCCTACCGATTTCGAGTGATC(	39	0	0
1221	GTCGCTCCTACCGATTTCGAGTGGTC(	25	0	0
1294	GTCGCTCCTACCGATTTCGAGTGGCG(	44	0	0
1296	GTCGCTCCTACCGATTTCGAGTGGTC(	115	0	0
1299	GTCGCTCCTACCGATTTCGAGTGGTC(	23	0	0
1350	GTCGCTCCTACCGATTTCGAGTGGTC(	214	0	7
1398	GTCGCTCCTACCGATTTCGAGTGGTC(	8	0	0
1399	GTCTCTCCTACCGATTTCGAGTGGTCC	177	1	1
1402	GTCGCTCCTACCGATTTCGAGTGGTC(	16	0	0
1405	GTCGCTCCTACCGATTGAATGATCCG(	8	0	0
1460	GTCGCTCCTACCGATTTCGAGTGGTC(	92	0	3
1465	GTCGCTCCTACCGATTTCGAGTGGTC(	36	0	0
1468	GTCGCACCTACCGATTGAATGGTCCG	11	0	0
1469	GTCGCTCCTACCGATTTCGAGTGGTC(	7	0	0
1478	GTCGCTCCTACCGATTTCGAGTGGTC(	56	1	0
1543	GTCGCTCCTACCGATTTCGAGTGGTC(	88	0	18
1557	GTCGCTCCTACCGATTTCGAGTGGTC(	19	0	0
1561	GTCGCTGCTACCGATTGGATGGTTCT(	6	0	0
1576	GTCGCTCCTACCGATTGAGTGATCCG(	8	0	1
1636	GTCGCTCCTACCGATTGAGTGGTCCG(	5	0	0
1643	GTCGCTGCTACCGATTGGATGGTTCT(	5	0	0
1749	GTCGCTCCTACCGATTGCGAGTGGTC(	36	0	2
1750	GTCGCTCCTACCGATTTCGAGTGGTC/	11	0	1
1751	GTCGCTCCTACCGATTTTCGAGTGGT(	15	0	1
1752	GTCGCTCCTACCGATTTCGAGTGGTC(	15	0	0
1760	GTCGCTCCTAACAATTTTCAATGGTCC	4	0	0
1765	GTCGCTCCTACCGATTTGAGTGGTCC(	4	0	0
1772	GTCGCTCCTACCGATTGAATGATCCG(	4	0	0
1774	GTCGCTCCTACCGATTTCGATTGGTCT	4	1	3
1786	GTCGCACCTACCGATTGAATGGTCCG	4	0	0
1917	GTCGCTCCTACCGATTTCGAGTGGTC(	9	0	1
1918	GTCGCTCCTACCGATTGCGAGTGGTC(	6	0	1
1921	GTCGCTCCTACCGATTTCGAGTGATC(	6	0	0
1922	GTCGCTCCTACCGATTTCGAGTGGGC/	21	0	5
1924	GTCGCTCCTACCGATTTCGAGTGGTC(	78	0	3
1927	GTCGCTCCTACCGATTTCGAGTGGTC(	9	0	1
1930	GTCGCTCCTACCGATTTCGAGTGGTC(	12	0	0
1931	GTCGCTCCTACCGATTTCGAGTGGTC(	18	1	2
1940	TTCGCTCCTACCGATTTCGAGTGGTCC	3	0	0
1947	ATCGCTCCTACCGATTTCGAGTGGTC(	18	0	0

1260	GTCGCTCCTACCGATTGAGTGGTCCG	11	0	0
43	GTCGCTCCTACCGATTTCGAGTGGTCC	33330	273	585
84	GTCGCTCCTACCGATTTCGAGTGGTCC	15012	14	176
100	GTCGCTCCTACCGATTTCGAGTGGTCC	4377	26	107
121	GTCGCTCCTACCGATTTCGAGTGGTCC	2982	2	0
137	GTCGCTATTTCCGATTTCGAGTGGTCC	3260	18	37
156	GTCGCTATTTCCGATTTCGAGTGGTCC	2111	0	93
285	GTCGCTTATACCGATTTCGAGTGGTCC	1062	13	15
314	GTCGCTACTACCGATTTCGAGTGGTCC	476	9	0
329	GTCGCTCCTACCGATTTCGAGTGGTCC	6005	7	119
411	GTCGCTACTACCGATTTCGAGTGGTCC	327	0	32
424	GTCGCTCCTACCGATTTCGAGTGGTCC	433	0	46
467	GTCGCTCCTACCGATTTCGAGTGGTCC	261	0	0
618	GTCGCTCCTACCGATTTCGAGTGGTCC	93	0	0
693	GTCGCTCCTACCGATTTCGAGTGGTCC	75	0	1
716	GTCGCTCCTACCGATTTCGAGTGGTCC	63	0	0
808	GTCGCTCCTACCGAGTTCGAGTGGTCC	2679	5	12
861	GTCGCTCCTACCGATTTCGAGTGGTCC	243	0	12
944	GTCGCTCCTACCGATTTCGAGTGGTCC	61	0	2
1092	GTCGCTCCTACCGATTTCGAGTGTTCC	59	1	0
1192	GTCGCTCCTACCGATTTCGAGTGGTCC	199	0	1
1255	GTCGCTCCTACCGATTTCGAGTGGTCC	273	1	2
1295	GTCGCTCCTACCGATTTCGAGTGGTCC	129	0	10
1297	GTCGCTCCTACCGATTTCGAGTGGTCC	20	0	0
1353	GTCGCTCCAACCGATTTCAATTGGACC	9	0	0
1461	GTCGCTCCTACCGATTTCGAGTGGTCC	78	0	1
1542	GTCGCTCCTACCGATTTCGAGTGGTCC	6	0	0
1634	GTCGCTCCTACCGATTTCGAGTGGTCC	73	0	1
1635	GTCGCTCCTACCGATTTCGAGTGGTCC	28	0	0
1754	GTCGCTCCTACCGATTTCGAGTGGTCC	28	0	0
1759	GTCGCTACTACCGATTTCGAGTGGTCA	11	0	0
1763	GTCGCTCCTACCGATTTCGAGTGTCCT	4	0	0
1775	GTCGCTGCTACCGATTGGATGGTTCTC	4	0	0
1925	GTCGCTCCTACCGATTTCGAGTGGTCC	41	0	0
1926	GTCGCTCCTACCGATTTCGAGTGGTCC	26	0	0
1963	GTCGCTGCTACCGATTGGATGGTTCTC	3	0	0
10	GTCGCTACTACCGATTTCGAGTGGTCA	171655	1835	1390
155	GTCGCTACTACCGATTTCGAGTGGTCC	2046	0	55
202	GTCGCTACTACCGATTTCGAGTGGTCA	1207	0	0
203	GTCGCTACTACCGATTTCGAGTGGTCA	1192	0	0
312	GTCGCTATTTCCGATTTCGAGTGGTCC	487	0	4
367	GTCGCTACTACCGATCGAATGGTCAG	345	0	0
421	GTCGCTATTTCCGATTTCGAGTGGTCC	252	0	0
422	GTCGCTACTACCGATTTCGAGTGGTCC	315	0	0
544	GTCGCTACTACCGATTGAATGGTTCAC	131	0	0
642	GTCGCTACTACCGATTGAACGTTTTAC	99	1	0
1012	GTCGCTACTACCGATTGCGAGTGGTC	111	2	0
1052	GTCGCTACTACCGATTTCGAGTGGTCA	71	0	46

1138	GTCGCTACTACCGATTTCGAATGGTC	67	1	0
1174	GTCGCTACTACCGATCGAGTGGTCAG	21	0	0
1258	GTCGCTACTACCGATTTCGAGTGGTC	225	0	112
1266	GTCGCTACTACCGATTGAATGGCTTA	11	0	0
1267	GTCGCTACTACCGATTGATGTGCAGG	11	0	0
1301	GTCGCTACTACCGATTTGGAGGGGTC	34	1	0
1413	GTCGTTACTACCGATTGGTGTGCAGG	22	0	0
1471	GTCGCTCCTACCGATTGAGTGATCCG	11	0	0
1480	GTCGCTACTACCGATTGAATAACAGG	7	0	0
1640	GTCGCTAGTACCGATTTCGAGTGGTC	17	0	0
1770	GTCGCTACTACCGATTTCGAGTGGTC	62	0	0
1830	GTCGCTACTACCGATCGAGTGATCAG	4	0	0
1942	GTCGCTACTACCGATTGCGAGTGGTC	6	0	0
1954	GTCGCTACTACCGATTTCGAGTGGTC	57	0	28
1956	GTCGCTACTACCGATTTCGAGTGGTC	12	0	0
1980	GTCGCACCTACCGATTGAATGGTCCG	3	0	0
2001	GTCGCTACTACCGATTGAATGGTTTA	3	0	0
370	GTCGCTCCTACCGATTTCGAGTGATC	342	0	9
614	GTCGCTCCTACCGATTTCGAGTGATG	96	0	0
645	GTCGCTCCTACCGATTTTGAGTGATC	89	0	0
748	GTCGCTCCTACCGATTTCGAGTGATC	56	0	0
225	GTCGCTACTACCGATTGAGTGATCCG	1270	0	0
548	GTCGCTCCTACCGATTTCGAGTGATC	166	0	0
731	GTCGCTACTACCGATTGAGTGATCCG	69	0	0
1633	GTCGCTCCTACCGATTTCGAGTGATC	5	0	0
1113	GTCGCTCCTACCGATTTCGAGTGGTC	16	0	0
98	GTCGCTCCTACCGATTTCGAGTGATT	4061	0	0
1115	GTCGCTCCTACCGATTTCGAGTGATC	16	0	0
140	GTCGCTCCTACCGATTTCGAGTGATT	2334	106	9
372	GTCGCTCCTACCGATTTCGAGTGGTC	457	25	7
572	GTCGCTCCTACCGATTTCGGGTGATT	144	1	0
904	GTCGCTCCTACCGATTTCGAGTGATC	30	0	0
1642	GTCGCTCCTACCGATTTCGAATGTTC	5	0	0
59	GTCGCTCCTACCGATTTCGAGTGGTC	10903	70	4
1789	GTCGCTACTACCGATTTCGAGTGGTC	45	0	0
144	GTCGCTCCTACCGATTTCGAGTGGTC	2226	81	5
399	GTCGCTCCTACCGATTTCGAGTGATC	305	0	0
455	GTCGCTCCTACCGATTTCGAGTGGTC	207	0	0
637	GTCGCTCCTACCGATTTCGAGTGGTC	107	0	0
711	GTCGCTCCTACCGATTTCGAGTGGTC	67	0	0
746	GTCGCTCCTACCGATTTCGAGTGGTC	57	0	0
1412	GTCGCTCCTACCGATTTCGAGTGTTC	21	0	0
118	GTCGCTCCTACCGATTTCGGGTGATT	4318	15	3
700	GTCGCTCCTACCGATTTCGAGTGATC	222	0	0
176	GTCGCTCCTACCGATTGCGAGTGGTC	1506	1	0
1652	GTCGCTCCTACCGATTTCGGGTGATT	8	0	0
87	GTCGCTCCTACCGATTTCGGGTGATT	4931	0	0
239	GTCGCTCCTACCGATTTCGAGTGGTC	2228	0	28

1779	GTCGCTCCTACCGATGAGGGATCCGG	4	0	0
38	GTCGCTCCTACCGATTGAATACATTG(	13270	123	5
160	GTCGCTCCTACCGATTTCGAGTGATC(	3843	5	25
442	GTCGCTCCTACCGATTTCGGGTGATT(	221	0	40
641	GTCGCTCCTACCGATTTCGGGTGATT(	85	0	9
865	GTCGCTCCTACCGATTTCGGGTGATT(	49	0	0
1139	GTCGCTCCTACCGATTTCGAGTGATC(	29	0	0
1422	GTCGCTCCTACCGATTTCGGGTGATT(	8	0	0
1306	GTCGCTCCTACCGATTTCGAGTGATC(	10	0	0
52	GTCGCTCCTACCGATTGAGTGGTTCG(	9596	136	101
55	GTCGCTCCTACCGATTTCGAGTGATC(	9011	1036	23
196	GTCGCTCCTACCGATTTCGAGTGATC(	1273	0	0
501	GTCGCTCCTACCGATTTCGAGTGATC(	153	0	0
506	GTCGCTCCTACCGATTTCGAGTGATC(	163	0	15
719	GTCGCTCCTACCGATTTCGAGTGATC(	62	0	0
740	GTCGCTCCTACCGATTTCGAGTGATC(	58	0	0
747	GTCGCTCCTACCGATTTCGAGTGATC(	56	0	0
763	GTCGCTCCTACCGATTTCGAGTGATC(	53	0	1
862	GTCGCTCCTACCGATTTCGAGTGATC(	35	0	0
875	GTCGCTCCTACCGATTTCGAGTGATC(	34	0	0
933	GTCGCTCCTACCGATTTCGAGTGATC(	36	0	0
1263	GTCGCTCCTACCGATTTCGAGTGATC(	11	0	0
1265	GTCGCTCCTACCGATTTCGAGTGGTC(	11	0	0
1406	GTCGCTCCTACCGATTTCAAGTGATC(	8	0	0
1473	GTCGCTCCTACCGATTTCGAGTGATC(	7	0	0
1637	GTCGCTCCTACCGATTTCGAGTGATC(	5	0	0
1785	GTCGCTCCTACCGATTTCGAGTGATC(	8	0	0
1978	GTCGCTCCTACCGATTTCGAGTGATC(	12	0	0
1784	GTCGCTCCTACCGATTTCGAGTGATC(	4	0	0
348	GTCGCTCCTACCGATTCGAGTGATCC(	426	0	0
1491	GTCGCTCCTACCGATTCGAGTGATCC(	10	0	0
621	GTCGCTCCTACCGATTTCGAGTGATT(	131	0	3
960	GTCGCTCCTACCGATTTCGAGTGATT(	25	0	0
1400	GTCGCTCCTACCGATTTCGAGTGATT(	8	0	0
148	GTCGCTCCTACCGATTTCGAGTGATC(	2131	0	4
227	GTCGCTCCTACCGATTTCGAGTGGTC(	1028	0	0
877	GTCGCTCCTACCGACTTGAGTGGTCC(	34	0	0
1689	GTCGCTCCTACCGATTGAGTGGTTTG(	42	0	0
95	GTCGCTCCTACCGATTTCGAGTGTTC(	4154	0	1
151	GTCGCTCCTACCGATTTCGAGTGATC(	2031	0	0
284	GTCGCTCCTACCGATTTCGAGTGATC(	1116	0	2
393	GTCGCTCCTACCGATTTCGAGTGATC(	345	0	0
761	GTCGCTCCTACCGATTTCGAGTGATC(	53	0	0
775	GTCGCTCCTACCGATTTCGAGTGATC(	94	0	0
863	GTCGCTCCTACCGATTTCGAGTGATC(	35	0	0
876	GTCGCTCCTACCGATTTCGAGTGATC(	34	0	1
1076	GTCGCTCCTACCGATTTCGAGTGATC(	18	0	0
1464	GTCGCCCCTACCGATTCCGAGTGATC(	12	0	0

345	GTCGCTCCTACCGATGAGTGATCCGG	425	0	13
1732	GTCGCTCCTACCGATTGAGTGATTCGC	5	0	0
2259	GTCGCACCTACCGATTGAATGGTCCG	3	0	0
190	GTCGCTCCTACCGATTTCGAGTGGTCA	1440	0	0
1102	GTCGCTACTACCGATTGAACGTTTTAG	29	0	0
1854	GTCGCTCCTACCGATTTCGAGTGATGA	4	0	0
185	GTCGCTCCTACCGATTGGGTGATGCG	2957	0	362
633	GTCGCTCCTACCGATTGGATGATGCG	88	0	0
735	GTCGCTCCTACCGATTGGATGATGCG	113	0	1
1432	GTCGCTCCTACCGATTGGGTGATGCG	8	0	0
1502	GTCGCTCCTACCGATTTCGAGTGATTA	21	0	0
1387	GTCGCTCCTACCGATTGAGTGGTCCGC	18	0	0
650	GTCGCTCCTACCGATTTCGAGTGTGCA	178	0	0
1911	GTCGCACCTACCGATTGAGTGATCCG	4	0	0
60	GTCGCTCCTACCGATTTCGAGTGATCA	22059	13	334
760	GTCGCTCCTACCGATTTCGAGTGACTA	66	0	0
892	GTCGCTCCTACCGATTTCGAGTGATCA	32	0	1
901	GTCGCTCCTACCGATTGAGTGTTCCGC	36	0	0
1069	GTCGCTCCTACCGATTTCGAGTGTGCA	39	0	2
1106	GTCGCTCCTACCGATTTCGAGTGATTA	17	0	0
1519	GTCGCTCCTACCGATTGAGTGATCCGC	7	0	0
1614	GTCGCTCCTACCGATTGGATGTGCTGC	6	0	0
1720	GTCGCTCCTACCGATTTCGAGTGATCA	98	0	0
1721	GTCGCTCCTACCGATTTCGAGTGATCA	10	0	0
2160	GTCGCTCCTACCGATTTCGAGTGATCA	6	0	0
2267	GTCGCTCCTACCGATTTCGAGTGATCA	48	0	1
1287	GTCGCTCCTACCGATTTCGAGTGTACA	15	0	0

7.SUR	7.DCM	9.SUR	9.DCM	11.SUR	16.SUR	16.DCM
0	0	0	0	0	0	0
0	0	0	0	0	1	0
0	0	0	0	0	0	0
0	0	0	0	0	0	0
0	0	0	0	0	0	0
0	0	0	0	0	0	0
0	0	0	0	0	0	0
0	1	0	0	0	0	0
0	0	6	53	0	0	0
0	0	0	0	0	0	3
0	0	67	0	0	0	0
0	1	0	1	0	0	0
0	0	0	0	0	0	0
0	0	0	0	0	0	0
0	0	0	2	0	0	4
0	0	0	0	0	0	0
0	0	5	11	0	1	43
115	473	16	45	57	19	85
1	0	3	0	0	0	0
0	0	1	1	0	1	2
0	0	0	0	0	0	0
0	0	0	0	0	0	0
0	0	0	0	0	0	1
0	0	2	0	0	0	0
0	0	0	0	0	0	0
0	0	0	0	0	0	0
0	0	0	0	0	0	0
0	0	0	0	0	0	0
0	0	0	0	0	0	0
0	0	1	0	0	0	0
0	0	0	0	0	0	0
1	0	0	0	0	0	0
0	72	1	5	0	0	9
0	0	0	0	0	0	0
0	0	0	0	0	0	0
0	0	0	0	0	0	0
6	660	336	0	0	46	0
80	12	8068	37	1	162	3
8	5	0	0	0	2	0
0	0	0	0	0	0	9
0	52	11	91	1	6	37
3	1034	0	1	2	0	0
2	0	0	0	0	3	0
0	31	0	1	2	4	3
0	199	1	21	6	1	81
0	0	0	0	0	0	11
0	310	2	12	0	0	14

0	4	3	3	0	1	13
0	0	2	7	0	0	2
0	0	1	0	0	1	0
0	44	1	7	0	0	7
0	1	0	5	0	0	10
0	0	0	0	0	0	0
0	0	1	10	0	0	4
0	0	0	0	0	0	0
0	0	0	0	1	10	1
0	0	0	0	0	0	0
0	3	0	0	0	1	0
0	1	0	0	0	0	0
0	2	0	0	0	0	0
0	0	2	20	0	0	1
0	0	0	2	2	0	5
0	0	0	0	0	0	0
0	0	0	0	0	0	0
0	1	0	0	0	0	0
0	0	0	0	0	0	0
0	0	0	0	0	0	0
0	0	0	1	0	0	0
0	0	0	0	0	0	0
0	0	0	0	0	0	0
0	0	0	0	0	0	0
0	0	0	0	0	0	0
0	0	0	0	0	0	0
0	0	0	0	0	0	0
0	0	0	0	0	0	0
0	0	0	0	0	0	0
0	0	0	0	0	0	0
0	0	0	0	1	35	2
0	0	0	0	0	0	0
0	0	0	0	0	0	0
0	0	0	0	0	0	0
0	0	0	0	0	0	0
0	0	0	0	0	0	0
1	0	3	1	1	0	0
0	0	0	0	0	0	0
0	0	0	0	0	0	0
0	0	64	1	0	11	0
137	927	244	76	694	768	81
2	1	420	6	1	4	1
0	0	0	0	0	3	0
0	2	0	1	0	16	0
0	0	0	0	0	0	0
0	0	0	0	0	0	0
0	0	0	2	0	0	0
0	2	0	0	1	1	0

0	0	0	0	0	0	0
0	0	0	0	2	0	0
0	0	0	0	0	0	0
0	0	6	0	0	0	0
0	0	0	0	0	0	0
0	1	92	22	2	0	0
0	0	0	0	0	0	0
0	0	0	0	0	0	0
0	0	0	0	0	0	0
0	0	0	0	0	0	0
0	0	0	0	0	0	0
0	0	0	0	0	0	0
0	0	0	0	0	0	0
0	0	0	0	0	0	0
0	0	0	0	0	0	0
0	4	0	0	0	0	0
0	15	0	3	0	0	0
0	0	0	0	0	0	0
0	0	0	0	0	0	0
3	25	138	0	0	0	0
0	0	0	0	0	0	0
0	0	0	0	0	0	0
0	0	35	416	0	0	3
0	1	0	3	0	1	0
0	0	0	3	0	0	0
0	13	0	2	2	4	6
0	0	0	0	0	0	0
2	1	0	0	0	1	0
0	0	0	0	1	2	9
0	0	0	0	0	0	0
0	0	0	0	0	0	1
0	0	0	0	0	0	0
0	0	0	0	0	0	0
0	0	0	0	0	0	0
0	0	0	3	0	0	2
0	0	4	0	0	0	0
0	0	242	0	0	3	0
0	0	0	0	0	0	0
0	0	0	0	0	0	0
0	0	0	1	0	0	0
0	2	0	10	0	0	0
0	0	0	1	0	0	0
0	0	0	0	0	0	0
0	0	0	0	0	0	0
0	0	0	0	0	0	0
0	0	0	0	0	0	0
0	0	0	0	0	0	0
0	2	0	1	0	0	0

0	0	0	2	0	0	0
0	0	0	0	0	0	0
0	8	8	123	2	1	49
0	0	0	0	0	1	0
0	0	8	0	0	0	0
0	0	0	0	0	0	0
0	0	0	0	0	0	0
0	0	0	0	0	0	0
0	0	0	0	0	0	0
0	0	0	0	0	4	0
0	1	1	5	2	0	46
0	0	0	0	0	0	6
0	9	243	16	0	1	11
0	0	0	0	0	0	0
0	96	1	13	7	0	57
0	0	0	0	0	1	0
0	0	0	1	0	0	4
0	42	0	8	0	0	19
0	0	0	0	0	0	0
0	0	0	1	0	0	1
1	2	30	2	0	0	0
0	0	0	0	0	0	0
0	0	0	0	0	0	0
0	0	0	0	0	0	1
0	0	2	0	0	0	1
0	0	0	0	0	0	0
0	0	0	0	0	0	0
0	0	7	0	9	0	0
0	0	0	0	0	0	0
0	0	1	2	0	0	0
0	0	0	0	0	0	0
0	0	0	0	0	0	2
12	23	12	0	0	0	0
0	7	0	3	2	0	9
0	0	0	1	0	0	0
0	0	0	0	0	0	3
0	0	0	0	0	0	0
0	0	0	0	0	0	0
0	0	0	0	0	1	0
0	0	0	0	0	0	1
0	0	0	0	0	0	0
0	0	0	0	0	0	0
1937	5835	537	3	3030	0	3
0	3	37	0	0	0	10
0	31	2	61	0	1	0
0	0	0	0	0	0	0
0	0	3	32	0	0	0
0	0	0	81	0	0	0

0	0	0	1	0	0	0
0	0	0	0	0	0	0
81	7	0	0	0	0	0
0	0	0	0	0	0	0
0	0	0	0	0	0	0
0	1	0	0	0	0	0
0	0	0	0	0	8	0
0	0	0	0	0	0	0
0	0	0	0	0	0	0
0	0	0	0	0	0	0
0	2	0	0	0	0	0
0	0	0	0	0	0	0
0	0	0	0	0	0	0
0	0	3	1	0	0	0
0	0	0	0	0	0	0
0	0	0	0	0	0	0
0	0	0	0	0	0	0
0	0	0	0	0	0	0
0	0	0	0	0	0	0
0	3	0	0	0	0	1
3	0	5	0	0	0	0
0	4	2	5	1	0	5
0	0	0	0	0	0	0
80	10	907	0	442	8	2
3	2	0	1	2	0	0
0	0	0	0	0	0	0
0	0	0	0	0	0	0
0	0	10	0	3	0	0
0	0	0	0	0	0	0
0	0	0	0	4	0	0
0	0	0	0	0	0	0
0	0	0	0	0	0	0
0	4	0	0	1	0	0
0	0	0	0	0	0	0
0	0	0	0	0	0	0
0	0	148	0	0	0	0
9	5	42	22	4	1	58
1	5	6	1	0	0	7
91	0	0	0	6	0	9
0	0	0	0	0	0	0
0	0	0	0	1	0	8
0	0	0	34	0	0	0
0	0	0	0	0	0	0
0	0	0	0	0	0	0
0	0	0	0	0	0	0
0	0	0	2	0	0	0
0	0	0	1	0	0	0
0	0	0	0	0	0	0

0	0	0	1	0	0	0
0	0	0	0	0	0	0
0	0	0	0	1	0	0
0	0	12	0	0	0	0
0	0	0	0	0	0	0
0	0	0	0	0	0	0
0	0	0	0	0	0	0
1	0	0	0	0	0	0
0	0	0	0	0	0	0
0	0	0	0	0	0	0
0	0	0	0	0	0	0
0	0	0	0	0	0	0
1	0	0	0	0	0	0
0	0	0	0	0	0	0
0	0	2	0	0	0	0
0	0	0	0	0	0	0
0	0	0	0	0	0	0
0	0	0	0	0	0	0
0	0	0	0	0	0	0
0	1	118	0	28	3	0
0	0	0	0	0	0	0
6	0	1	0	0	71	1
0	0	0	0	0	0	0
29	341	254	121	13	18	189
0	0	0	0	1	7	0
0	0	0	0	1	0	0
0	3	1	0	0	4	3
0	0	0	0	0	1	0
0	0	0	0	0	0	0
0	0	0	0	0	0	0
0	0	0	0	0	0	0
0	0	0	0	0	0	0
0	1	0	3	0	1	6
0	0	0	2	0	0	1
0	0	0	0	0	0	3
0	0	0	0	0	0	0
0	0	0	0	0	0	0
0	0	0	0	0	0	0
0	0	0	0	0	1	0
0	16	0	2	1	1	6
0	0	0	0	0	0	0
0	0	0	0	0	0	0
0	3	0	1	0	0	4
0	0	0	0	0	0	0
0	0	0	0	0	0	0
0	0	0	0	0	0	0
0	0	0	7	0	0	1
0	0	0	0	0	0	0

0	0	0	2	0	0	0
0	0	0	0	0	0	4
0	0	117	0	0	0	0
47	323	91	2	4	0	0
0	0	0	0	0	0	0
0	0	0	0	0	0	0
699	536	3815	15	552	1	4
0	0	0	0	0	0	0
0	0	1	0	0	0	0
0	0	0	0	0	0	0
0	0	0	0	0	0	0
2097	4097	6243	1964	573	6008	3566
0	124	11	138	0	19	52
50	5	388	24	17	3	330
0	18	3	0	1	5	17
1	619	8	91	9	17	122
30	601	273	55	42	4	271
1	162	3	41	16	2	75
0	0	1	0	1	714	2
0	1	9	66	0	0	0
0	0	0	0	0	1	4
0	10	0	2	0	19	0
0	800	3	29	0	0	0
0	0	0	0	0	0	0
0	0	0	5	0	1	0
0	0	0	0	0	5	0
0	0	0	0	0	0	0
0	0	0	2	0	0	12
0	0	0	0	6	0	71
0	73	4	44	0	0	3
0	385	0	4	0	3	9
0	17	0	8	1	0	19
0	0	0	0	0	0	0
0	22	0	0	0	0	6
0	0	0	0	0	0	0
4	0	0	0	0	0	0
156	133	458	30	35	193	72
0	0	0	0	0	0	0
0	0	0	1	0	40	0
0	15	2	22	0	0	3
0	0	0	0	0	0	0
0	0	0	0	0	0	0
0	0	0	4	0	0	5
0	0	0	0	0	0	0
0	0	0	0	0	1	0
0	0	0	0	0	0	0
0	0	0	0	0	0	3
0	0	0	0	0	1	0

0	0	1	0	0	99	0
2	6	15	0	0	5	0
0	0	0	0	0	0	0
19	0	12	0	0	0	0
0	0	0	0	0	0	0
0	0	0	0	0	0	0
0	0	0	0	0	0	1
0	0	1	0	0	0	0
0	0	0	0	0	0	0
0	0	0	0	0	0	0
0	0	0	0	0	0	0
0	0	0	1	0	0	0
1	0	0	4	0	1	0
0	0	0	0	1	1	11
0	7	0	0	0	0	0
0	0	0	0	0	0	0
0	0	0	0	0	0	0
0	0	0	4	0	0	0
0	1	0	0	0	44	0
0	0	1	0	0	0	0
0	0	0	0	0	0	0
0	2	5	4	0	10	8
0	0	0	0	0	0	0
0	0	0	0	0	0	0
0	0	1	0	0	0	0
0	3	0	0	0	0	0
0	0	0	0	0	0	0
1	0	5	0	0	2	0
0	0	0	2	0	0	0
0	3	0	0	0	0	1
0	0	0	0	0	2	0
4	3	13	4	0	4	2
3	5	23	1	1	1	9
9	8	22	3	1	5	2
0	0	0	0	0	0	0
0	0	0	0	1	0	1
0	1	10	0	1	0	13
0	0	0	0	0	0	0
0	0	0	0	0	0	1
0	0	0	0	0	1	1
0	1	0	0	0	0	0
0	5	0	1	0	0	2
0	0	0	0	0	0	0
0	0	0	1	0	0	2
0	0	0	0	0	0	0
0	0	0	0	0	0	0
0	0	0	0	0	0	0
0	0	0	0	0	0	0

0	0	1	0	0	1	1
2	1	5	0	0	0	0
0	0	0	0	0	0	0
0	0	0	0	0	1	0
0	1	0	0	0	0	0
0	1	1	0	0	0	0
0	0	0	0	0	0	0
2	3	8	0	0	1	0
0	0	0	0	0	0	0
6	5	15	0	3	8	4
0	0	0	0	0	0	0
0	0	0	0	0	0	0
0	0	0	0	0	0	0
0	0	4	0	0	0	0
0	0	0	0	0	0	0
0	0	0	0	0	0	0
0	0	0	0	0	0	1
0	0	0	0	0	0	0
0	0	1	0	0	3	0
0	0	0	0	1	0	0
0	0	0	0	0	0	0
0	0	0	0	0	0	0
0	0	0	0	0	2	0
0	0	0	0	0	0	0
1	0	0	0	0	0	1
0	0	1	0	0	0	0
0	0	0	0	0	0	0
3	0	0	0	0	0	0
0	0	0	0	0	0	0
0	0	0	0	0	0	0
0	0	2	6	0	0	0
0	0	10	70	0	0	22
0	0	0	0	0	0	0
0	0	0	0	0	0	0
0	0	0	0	0	0	0
0	0	0	7	1	3	2
0	0	1	14	0	1	7
0	0	0	0	1	0	12
0	3	0	12	0	4	5
0	0	0	0	0	35	0
0	0	0	0	0	0	0
0	0	0	0	0	0	0
0	0	1	20	0	0	18
0	0	0	0	0	0	0
0	0	0	0	0	0	0
0	0	0	0	0	1	0
0	0	0	0	0	0	0
18	0	3	0	4	10	0

0	52	0	18	2	0	9
0	0	0	3	1	2	6
0	0	0	0	0	2	0
9	31	1	2	1	0	3
0	0	0	0	0	0	0
0	0	0	0	0	0	0
112	7	733	3	0	82	0
0	2	2	12	1	0	3
1	173	92	11	1	22	33
0	5	0	26	1	2	2
0	0	0	4	0	0	2
0	4	0	4	2	0	26
0	0	0	0	0	0	0
0	0	0	0	0	0	0
0	0	0	0	0	0	0
0	0	0	0	0	2	0
0	0	0	0	0	0	0
0	0	0	0	0	0	0
0	3	31	34	1	5	13
0	9	0	5	2	0	29
0	0	0	0	0	0	0
0	0	1	2	0	0	0
0	2	0	5	1	0	9
0	0	0	0	0	0	0
0	0	0	0	0	0	0
0	0	0	0	0	0	0
4	79	1	8	0	0	9
0	0	0	0	0	13	0
0	0	0	0	0	0	0
0	0	1	0	0	0	1
0	0	0	0	0	0	0
0	0	0	0	0	0	0
204	183	6	0	2	0	0
0	0	0	0	0	0	0
0	0	0	0	0	2	0
0	0	0	0	0	0	0
0	0	132	0	0	0	0
0	0	0	0	16	0	0
0	0	0	0	0	0	0
0	0	0	0	0	0	0
0	0	0	0	0	3	0
0	0	0	0	0	0	0
0	88	6	27	0	0	8
0	49	0	0	0	0	2
0	0	0	0	0	0	0
0	0	0	0	0	0	0
0	0	0	0	0	0	10
0	0	0	0	0	0	0

6	24	4	1	8	0	0
0	0	0	0	0	0	0
0	4	188	8	104	1190668	2914
0	0	0	9	0	0	0
0	0	0	0	0	43	0
0	0	0	0	0	1	0
0	0	0	0	0	0	0
0	0	0	0	0	0	0
0	0	0	0	0	2	1
4	3	8	0	0	0	0
0	0	0	0	0	0	0
24	2	0	0	0	0	0
0	0	0	0	0	0	0
0	0	0	0	0	0	1
0	0	0	0	0	0	0
0	0	0	0	0	0	0
0	0	0	0	0	0	0
0	0	0	0	0	0	0
0	0	0	0	2	0	9
9	4	0	0	0	1	0
0	0	0	0	0	0	0
0	5	0	0	0	0	0
0	0	0	0	0	0	0
0	5	1	18	1	0	17
1	22	2	23	0	0	2
0	1	0	0	0	0	0
0	0	0	0	0	0	0
0	0	0	0	0	0	0
0	15	0	0	0	0	0
2	0	1	0	0	0	1
0	0	0	0	0	0	0
0	21	3	8	0	0	0
0	0	0	0	0	30	0
0	0	0	0	0	0	0
0	0	0	0	0	0	0
0	0	0	0	0	0	0
2	0	2	0	0	0	1
0	0	0	0	0	0	0
0	0	0	0	0	0	0
0	0	0	0	0	0	0
184	160	121	17	30	3	65
0	0	0	0	0	0	0
0	4	0	0	0	0	7
991	864	4048	324	902	875	2537
0	1	8	3	0	331	24
548	49	644	8	78	65	66
20	9	390	16	84	353	66
2	43	15	7	6	148	101

1	1	16	0	0	1	0
6	92	11	74	5	15	9
4	24	17	9	0	0	11
9	0	0	1	0	1	0
0	1	0	1	0	0	6
0	0	0	0	0	1	0
128	0	3	2	0	0	3
0	1	0	1	0	0	24
2	0	1	2	0	39	2
4	0	0	0	0	0	0
0	2	117	3	5	0	33
0	8	1	1	0	1	0
0	1	271	0	0	0	0
0	2	1	0	0	0	0
0	0	0	0	0	0	2
9	15	13	0	0	0	5
0	9	0	4	2	0	20
0	20	3	4	6	1	86
0	0	0	0	0	0	0
5	21	4	15	0	0	6
58	0	0	1	0	0	3
6	0	1	0	1	0	1
0	0	0	0	0	0	0
0	5	0	0	0	0	0
0	0	0	9	0	0	1
2	1	4	0	0	0	0
0	0	0	0	2	3	0
0	0	0	0	0	0	1
0	0	0	4	0	0	0
0	0	0	0	0	0	4
0	0	0	0	0	0	0
0	0	0	0	0	0	0
0	0	0	0	0	0	0
0	0	2	1	0	0	1
0	0	0	0	0	0	1
0	0	0	0	0	0	0
0	4	0	0	0	0	1
4	0	0	0	0	0	0
0	0	0	0	0	0	0
0	0	0	0	0	0	0
0	0	0	0	0	0	0
0	1	1	0	0	1	1
0	0	0	0	0	3	0
0	0	0	0	0	0	0
0	0	0	0	0	0	0
0	0	0	1	0	0	0
1	1	0	0	0	0	0
0	0	0	0	0	0	0

0	0	0	0	0	0	0
0	0	0	0	0	0	0
0	0	1	0	0	0	0
0	1	0	0	0	0	0
0	0	0	0	0	0	0
30	35	11	0	3	2	0
0	0	0	0	0	0	0
6	439	15	95	14	151	200
0	0	0	0	0	1	3
0	0	0	0	3	0	0
0	0	0	0	0	0	0
0	0	0	0	0	0	0
0	0	0	0	0	0	0
0	0	0	0	0	0	0
0	0	0	0	0	0	0
0	0	0	0	0	0	0
0	0	0	0	0	0	0
0	0	0	0	0	0	0
0	0	0	0	0	0	0
0	0	0	0	0	0	0
0	0	0	0	0	0	0
0	0	0	0	0	0	0
0	0	0	0	0	0	0
0	0	0	0	0	0	0
0	0	0	0	0	0	0
0	0	0	0	0	0	0
0	0	0	0	0	0	0
0	0	0	0	0	0	0
0	0	0	0	0	0	0
0	0	0	1	0	0	0
6	2	2	1	0	0	0
0	9	14	4	2	17	2
0	7	0	0	0	0	0
0	0	0	0	0	0	0
3	2	0	0	0	0	0
0	35	0	4	3	2	1
107	8	170	1	14	2	6
1	12	2	2	70	118	2
0	49	1	20	1	34	4
0	0	0	0	0	0	0
0	0	0	0	0	0	0
0	0	0	0	0	0	0
0	0	0	0	0	0	0
0	6	0	2	0	0	0
0	4	0	0	0	7	0
0	2	0	1	0	0	0
0	0	0	0	0	0	0
0	0	0	0	0	0	0

0	1	0	0	0	0	0
0	0	0	0	0	0	0
0	0	0	0	0	0	0
0	5	0	1	0	0	0
0	0	0	0	0	0	0
0	0	0	8	0	0	6
0	1	6	0	0	0	0
0	0	0	0	0	0	0
0	0	0	0	0	0	0
0	0	0	0	0	48	0
145	0	93	0	2	25	0
0	1	3	0	0	0	0
0	8	221	2	0	0	0
0	0	222	0	0	0	0
3151	37	12	0	8	0	22
17	97	1	13	0	3	5
0	0	0	0	0	0	0
0	0	0	0	0	0	0
0	7	2	6	1	0	16
0	0	0	0	0	2	0
1	21	0	0	0	0	16
0	7	0	1	0	0	0
0	16	4	40	4	0	46
60	29	5	1	0	0	2
0	3	2	7	0	0	8
0	0	81	0	0	2	0
16	14	3	0	1	25	1
0	16	1	7	0	0	0
0	0	0	4	0	0	0
0	0	13	1	1	0	26
0	0	0	0	0	0	0
0	0	0	0	0	0	2
0	0	0	0	0	0	0
0	0	0	0	0	0	0
0	0	0	0	0	0	0
0	0	0	0	0	0	0
0	0	0	0	0	0	0
0	0	0	0	0	0	0
0	0	0	0	0	0	0
2	0	1	3	0	0	0
0	0	0	7	0	0	0
0	0	0	0	0	0	0
0	0	0	0	0	0	0
0	0	1	0	0	0	0
0	0	0	0	0	0	0
0	0	0	0	0	0	0
0	0	0	0	0	0	0
0	0	0	0	0	0	0

0	0	0	0	0	0	0
103	24	292	64	23	63	239
20	11	2	4	2	32	52
0	11	2	2	1	1	10
81	18	21	22	24	11	0
0	0	0	0	0	0	0
2	8	89	3	1	29	3
69	2	39	4	1	59	0
0	0	0	0	0	27	0
0	1	1	8	4	0	76
0	0	1	24	0	0	3
0	0	1	8	2	0	60
3	0	9	3	1	19	0
0	1	2	6	0	0	3
4	6	2	1	2	0	0
0	0	0	0	0	0	0
0	0	0	0	0	0	0
0	5	1	2	0	0	0
0	0	0	0	1	0	10
0	1	0	0	0	0	0
0	1	14	0	0	0	1
0	0	0	0	0	0	0
0	0	0	0	0	0	0
1	0	0	0	0	0	0
0	0	0	0	0	0	0
0	0	1	0	0	0	0
0	0	0	0	0	0	0
0	0	0	0	0	0	0
0	0	0	0	0	0	0
0	0	0	0	0	0	0
0	0	0	0	0	0	0
0	0	0	0	0	0	0
0	0	142	4	1	0	46
23	68	94	64	30	14	63
505	6	1237	0	1540	113	2
0	22	0	0	0	1	7
0	0	0	3	0	1	0
1	3	45	0	4	0	0
0	7	10	1	8	3	14
24	35	12	17	6	1	32
1	0	126	2	1	0	2
39	23	84	33	21	98	42
0	0	0	0	0	0	0
0	0	0	7	0	3	15
6	3	4	0	0	0	0
51	105	2	0	0	1	9
0	0	1	4	0	0	3
0	0	0	0	0	0	0

1	0	1	0	0	0	0
0	0	0	0	0	0	0
0	0	0	0	0	0	0
0	0	0	0	0	0	0
0	0	0	0	0	0	0
0	0	0	0	0	0	0
0	0	0	0	0	0	0
0	0	0	0	0	0	0
0	0	0	0	0	0	0
0	0	0	0	0	0	0
0	0	0	0	0	0	0
0	0	1	0	0	0	0
0	0	0	0	0	0	0
0	0	0	0	0	0	0
0	0	0	0	0	0	0
0	0	0	0	0	0	0
0	0	0	0	0	0	0
0	0	0	0	0	0	0
0	0	0	0	0	0	0
0	0	0	0	0	0	0
0	0	0	0	0	0	0
0	0	0	0	0	0	0
0	0	0	0	0	5	0
0	0	0	0	0	0	0
0	0	80	0	0	0	0
0	0	0	0	0	0	0
2	1	0	0	0	0	0
0	0	0	0	0	0	0
0	0	0	0	0	0	0
0	0	2	0	0	0	1
0	16	1	0	3	0	0
0	0	0	0	0	0	0
1	134	0	0	1	0	1
1	147	1	0	1	0	0
0	0	0	0	0	0	0
1	3	0	0	0	0	0
0	0	0	0	0	0	0
0	4	0	0	0	0	0
0	0	0	0	0	0	0
0	0	1	0	0	0	0
0	0	112	0	0	0	0
0	0	0	0	0	0	0
0	0	0	0	0	0	0
0	6	0	0	0	0	0
0	0	0	0	0	0	0
0	0	0	0	0	0	0
0	0	0	0	0	0	0
0	0	0	1	0	0	0

79	8588	669	20	891	120	399
52	25	5	14	5	71	39
0	0	0	0	0	0	0
0	0	0	0	5	0	0
0	1	0	3	0	0	0
0	0	0	0	0	0	0
0	0	0	0	0	0	0
0	2	0	0	0	0	0
0	0	0	0	1	0	0
0	0	0	0	0	0	0
0	0	0	0	0	0	0
0	0	0	0	0	0	0
0	353	11	0	13	2	9
0	0	0	0	0	0	0
0	0	2	0	0	2	0
0	0	0	0	0	0	0
0	0	0	0	0	0	0
0	0	0	0	0	0	0
0	0	0	0	0	0	0
0	0	146	0	31	29	0
0	0	0	0	0	0	0
0	0	0	0	0	0	0
0	0	4	0	0	0	0
0	0	0	0	0	0	0
37	57	134	1	16	3	58
0	0	0	0	0	0	0
0	0	0	0	0	0	0
13	107	0	0	5	0	0
1	0	0	1	1	0	10
8	22	16	16	5	70	113
8	1	2	2	9	3	3
1	0	0	0	0	0	0
14	0	3	2	3	81	33
0	0	0	0	1	0	0
0	9	6	1	12	5	4
0	0	0	0	0	1	4
3	1	0	0	1	0	0
0	0	0	1	1	0	12
0	0	0	0	0	0	0
0	0	0	0	0	0	0
0	0	0	0	0	0	0
0	0	0	0	0	0	0
0	0	0	0	0	0	0
0	8	418	0	2	0	0
29	39	0	9	5	3	8
0	0	0	0	0	0	17
0	0	0	0	0	1	0
0	0	0	0	0	0	0

0	0	0	0	0	0	0
0	0	0	0	0	0	0
0	1	1	0	0	0	0
0	1	0	0	0	0	0
0	0	0	0	0	0	0
0	0	0	0	0	0	0
0	0	0	0	0	0	0
0	5	0	0	0	0	0
0	1	0	0	0	0	0
0	0	0	0	5	2	0
0	0	0	0	0	0	0
0	0	0	0	5	0	0
0	0	0	0	0	0	0
0	0	0	0	0	0	0
0	0	0	0	0	0	0
115	192	151	9	4043	47	25
0	2	20	2	45	0	89
7	4	0	1	10	0	0
0	0	0	0	0	0	0
0	0	0	0	0	0	0
1	0	0	0	5	0	0
0	0	0	0	1	0	0
0	0	0	0	0	0	0
0	0	0	0	0	0	0
0	1	7	0	16	0	0
1	9	0	0	1	1	0
0	0	0	0	2	0	0
0	0	0	0	0	0	0
431	210	33	1	83	13	0
0	12	161	9	16	41	45
407	291	84	0	24	0	0
0	5	0	1	8	0	39
90	32	100	25	9	60	45
0	0	0	0	0	0	0
0	0	0	0	0	0	0
0	0	0	0	0	0	0
0	0	0	0	0	0	0
0	0	1	0	0	0	0
1477	1254	1335	10	421	81	493
407	1225	307	0	80	0	118
0	0	1	1	6	0	38
0	0	0	0	0	1	1
12	16	160	0	21	0	15
0	0	0	0	0	0	0
0	0	0	0	0	0	0
1	1	1	0	0	0	1
0	0	0	0	0	0	0
0	0	0	0	0	0	0

2	1	0	0	0	0	0
0	0	0	0	0	0	0
0	0	0	0	3	0	0
0	0	0	0	0	0	0
1	0	0	0	0	0	0
0	0	0	0	0	0	0
2	0	2	0	0	0	0
1	0	1	0	0	0	0
0	0	0	0	0	0	0
0	1	0	0	0	0	1
0	0	1	0	0	0	0
19	18	0	0	75	0	5
0	0	0	0	0	0	0
0	0	0	0	0	0	0
0	0	0	0	3	47	13
0	0	0	0	0	0	0
0	43	0	0	0	0	2
57	109	2	0	0	0	2
0	0	0	0	3	0	0
0	0	0	0	0	0	0
0	0	5	0	0	0	0
0	0	0	0	0	1	0
0	1	0	0	0	0	11
0	0	0	0	0	0	0
0	0	0	0	0	0	0
3	1	208	4	787	38	24
73	23	134	0	23	0	14
0	0	1	0	5	0	1
0	22	0	1	0	21	0
0	0	0	2	3	0	1
0	15	9	80	14	3	417
6	0	0	0	0	1	1
0	0	0	0	0	0	0
0	0	0	0	0	0	0
0	0	1	0	0	0	0
0	0	0	0	0	0	0
0	0	0	0	0	0	0
0	0	0	0	0	0	0
0	0	0	0	0	0	0
0	0	0	0	0	0	0
0	0	0	0	0	0	0
0	0	0	0	0	0	0
0	149	3	23	0	0	8
109	365	55	0	3	0	18
0	0	16	0	0	0	0
0	0	0	0	0	0	0
0	0	0	0	0	0	0
0	0	0	0	0	0	0

0	0	0	0	0	0	0
0	0	0	0	0	0	0
2346	13296	446	146	936	200	671
3	19	67	0	6	9	12
1	132	337	279	50	54	512
0	0	3	0	0	2	0
86	298	9	59	13	34	313
0	105	153	82	12	19	281
0	41	7	6	1	0	58
105	61	129	29	35	37	8
0	0	0	0	0	17	0
0	3	0	0	0	0	19
1	4	26	39	5	1	53
0	0	0	0	0	8	0
11	1210	74	19	6	132	210
0	9	3	11	4	6	33
0	0	49	1	0	1	0
0	0	0	12	0	0	4
1	18	10	28	4	0	103
0	85	0	3	1	0	12
15	147	1	4	0	0	11
1	12	0	16	0	0	0
3	0	7	34	0	7	94
0	0	0	3	2	1	72
0	1	3	13	0	0	1
67	35	5	42	1	33	40
0	0	0	1	0	2	8
0	0	0	0	0	25	0
0	4	127	0	0	1	0
0	0	0	0	0	1	0
0	0	0	0	0	1	0
0	0	1	3	0	0	0
0	0	0	0	0	0	0
0	4	0	0	0	0	0
0	1	0	1	0	1	0
0	0	0	0	0	1	0
0	1	0	5	0	0	8
0	0	0	2	0	0	0
0	0	4	33	0	0	2
0	0	0	0	0	0	0
0	0	86	0	0	0	0
0	0	0	0	0	0	0
0	1	8	38	0	0	3
0	0	0	0	0	0	0
0	0	0	0	0	0	0
0	0	0	0	0	0	0
0	0	0	0	0	0	0
0	0	0	0	0	0	0

0	0	0	0	0	0	0
0	0	2	8	0	0	2
0	0	0	0	1	0	12
0	0	0	0	0	0	0
0	0	0	0	0	0	0
0	0	0	0	0	1	0
0	0	0	0	0	0	0
0	0	0	0	0	0	0
1	3	1	0	0	0	0
0	0	0	0	0	0	0
0	0	0	0	0	1	0
0	0	0	0	0	0	0
0	0	0	0	0	0	0
0	0	0	1	0	0	1
0	0	0	0	0	0	0
0	0	0	0	0	0	0
0	0	0	10	0	0	0
0	0	0	0	0	0	0
0	0	0	7	0	0	1
0	0	0	0	0	0	0
0	0	1	0	0	0	0
0	0	0	0	0	0	0
0	0	0	0	0	0	0
0	0	0	0	0	0	0
0	0	0	0	0	0	0
0	0	0	3	0	0	0
0	0	0	0	0	0	0
0	0	0	0	0	0	0
0	0	1	2	0	0	0
0	0	0	0	0	0	0
0	0	0	0	0	0	0
0	0	0	0	0	0	0
0	0	0	0	0	0	0
0	0	0	0	0	0	0
0	0	0	0	0	0	0
0	0	0	0	0	0	0
0	0	0	0	0	0	0
0	0	0	0	0	0	0
0	0	0	0	0	0	0
0	0	0	0	0	0	0
0	1	0	0	0	0	0
0	0	0	0	0	0	0
0	0	0	0	0	0	0
0	0	0	0	0	0	0
14	5	64	12	16	25	30
0	0	0	1	2	0	50
0	0	0	0	0	0	0
0	0	0	0	0	0	0

0	0	0	0	0	0	0
0	0	0	0	0	0	0
0	0	0	0	0	0	0
44	16	5	17	10	2	53
0	0	3	0	0	0	0
13	7	1	0	3	0	0
0	0	0	0	0	0	0
0	0	0	0	0	0	0
7636	485	4589	284	3336	350	521
2211	2014	4414	1048	1411	1132	3704
9	99	2	16	0	6	33
5	11	28	0	0	56	0
59	124	235	82	40	133	303
7	45	29	22	35	60	60
0	1	1	4	1	0	49
2	5	9	3	0	0	4
0	0	313	1	3	17	0
28	6	40	11	16	10	15
135	354	48	0	6	12	0
0	69	1	14	5	1	30
39	3	69	16	2	2	15
0	5	8	43	9	36	15
0	0	0	0	1	0	6
15	2	2	0	0	0	0
0	0	2	10	1	0	10
1	1	2	1	2	0	3
1	0	0	0	0	0	3
21	11	49	6	11	4	18
0	0	6	1	0	0	4
0	0	0	0	0	0	0
0	3	2	16	0	1	6
0	0	0	0	0	0	0
6	3	1	0	0	1	7
1	1	2	0	0	1	1
0	0	0	0	0	0	0
0	0	0	0	0	0	0
1	3	3	0	1	0	1
6	1	6	7	5	0	6
0	0	0	0	0	0	0
0	0	0	0	0	0	1
0	0	0	0	0	0	0
1	0	0	0	3	0	0
0	0	0	0	0	1	0
0	0	0	0	0	0	0
0	0	3	0	0	0	0
0	0	4	0	1	1	0
1	0	1	1	1	0	0
0	0	0	0	0	0	0

0	0	0	0	0	0	0
0	2	3	3	0	4	14
5	6	0	0	4	0	0
2	0	0	0	0	0	1
0	0	0	0	0	0	0
0	0	0	1	0	0	0
0	0	0	0	0	0	0
24	8	33	6	4	7	8
6	0	0	0	0	0	0
1	0	0	0	0	0	0
0	0	1	1	0	0	0
0	1	1	2	0	0	0
0	0	1	0	0	1	0
0	0	1	0	0	0	2
0	0	0	0	0	0	0
3	4	7	0	0	0	4
0	0	0	0	0	0	3
0	0	0	0	0	0	0
3	1	4	0	0	0	0
0	0	0	1	0	0	0
0	0	0	0	0	0	0
0	0	0	0	0	0	0
0	1	0	0	0	0	2
0	0	0	0	0	0	2
0	0	0	0	0	1	0
0	0	0	0	0	0	0
0	0	0	0	0	0	0
0	0	0	0	0	0	0
0	0	0	0	0	0	0
0	1	1	0	0	0	0
0	0	2	0	0	0	0
0	0	0	0	0	0	1
0	0	1	0	0	0	0
0	0	0	0	0	0	0
0	0	0	0	0	0	0
0	0	0	0	0	0	0
0	0	0	0	0	0	0
0	0	0	0	0	0	0
0	0	0	0	0	0	0
0	0	0	0	0	0	0
0	0	0	0	0	0	0
0	0	0	0	0	0	0
3	0	4	2	1	1	1
0	0	0	0	0	0	0
0	0	0	0	0	0	0
0	0	0	0	0	0	0
0	0	0	0	0	0	0
0	0	0	0	0	0	0

0	0	0	0	0	0	0
627	283	716	220	187	310	667
74	28	132	29	25	214	103
19	25	103	6	14	21	27
0	4	50	0	9	32	0
24	9	38	11	4	13	117
21	1	43	20	18	0	35
5	29	4	1	7	1	1
0	9	0	0	3	0	7
26	63	232	41	47	17	101
0	1	1	7	0	0	21
1	1	3	5	3	0	2
2	0	2	0	2	0	5
0	0	0	0	2	0	1
0	3	7	1	0	1	1
0	0	0	0	0	0	0
17	41	89	10	32	11	39
2	0	0	0	0	0	1
0	0	0	0	0	4	3
4	1	1	0	0	1	0
2	1	0	0	2	0	2
0	3	11	0	1	2	5
0	1	0	0	1	0	0
0	0	0	0	0	0	0
0	0	0	0	0	0	0
3	1	0	1	0	0	1
0	0	0	0	0	0	0
4	1	2	1	2	0	0
2	0	0	0	0	0	0
0	0	0	0	0	0	1
0	0	0	0	0	0	0
0	0	0	0	0	0	0
0	0	0	0	0	0	0
1	0	1	0	2	0	0
0	0	0	0	0	0	0
0	0	0	0	0	0	0
80	81	714	105	37	249	74
5	0	42	1	4	1	93
2	0	0	0	0	3	0
0	1	0	0	0	0	0
0	6	0	4	0	1	0
0	0	0	0	0	1	2
2	0	4	0	26	0	0
0	0	0	0	0	0	0
0	0	0	0	0	0	0
0	0	0	0	0	0	0
0	0	0	0	0	0	0
0	0	0	0	0	0	0

0	0	1	0	0	0	0
0	0	0	0	0	0	0
0	0	0	0	0	0	0
0	0	0	0	0	0	0
0	0	0	0	0	0	0
0	0	0	0	0	0	0
0	0	0	0	0	0	0
0	0	0	0	0	0	0
0	0	0	0	0	0	0
0	0	0	0	0	0	0
0	0	1	0	0	0	0
0	0	0	0	0	0	0
0	0	0	0	0	0	0
0	0	0	0	0	0	0
0	0	0	0	0	0	0
0	0	0	0	0	0	0
0	0	0	0	0	0	0
1	0	13	0	0	0	0
0	0	0	0	0	0	0
0	0	0	0	0	0	0
0	0	0	0	0	0	0
0	0	0	0	9	0	157
0	0	0	0	0	0	6
0	0	0	0	0	0	0
0	0	0	0	0	0	0
0	0	0	0	0	0	0
0	0	0	0	0	0	0
0	0	0	0	0	0	0
12	154	8	3	24	45	0
19	3	30	27	12	0	25
11	33	20	1	0	0	3
0	0	0	0	0	0	0
0	0	0	0	0	0	0
77	17	171	1	53	161	20
1	0	0	0	1	0	0
9	0	0	0	14	0	2
0	0	0	0	0	0	2
0	0	0	0	0	0	0
0	0	0	0	0	0	1
0	0	0	0	0	0	0
0	0	0	0	0	0	0
0	0	0	0	0	1	0
72	3	203	1	6	13	0
0	0	0	0	1	0	35
9	76	2	21	1	38	52
0	0	0	0	0	0	0
0	0	0	0	0	0	0
0	0	76	9	0	1	13

0	0	0	0	0	0	0
75	10	416	20	13	114	131
0	6	4	0	3	242	36
0	0	3	35	1	21	23
0	0	0	7	0	0	46
0	0	0	2	0	0	0
0	0	0	0	0	0	0
0	0	0	0	0	0	1
0	0	0	0	0	0	0
13	285	161	51	19	56	127
322	93	209	8	23	40	40
0	0	0	0	0	0	6
0	0	1	0	0	0	0
0	0	3	14	0	0	17
0	0	0	0	0	0	0
0	0	0	0	0	0	0
0	0	0	0	0	0	0
0	0	0	0	0	0	0
0	0	0	0	0	0	0
0	0	0	0	0	0	0
0	0	0	0	0	0	0
0	0	0	0	0	0	0
0	0	0	0	0	0	0
0	0	0	0	0	0	0
0	0	0	0	0	0	0
0	0	0	0	0	0	0
0	0	0	0	0	0	0
0	0	0	0	0	0	0
0	0	0	0	0	0	0
0	0	0	0	3	49	26
0	0	0	0	0	0	0
0	0	0	0	0	0	2
7	16	0	0	0	0	0
0	0	0	0	0	0	0
1	6	4	0	0	6	2
0	0	0	0	0	0	0
0	1	0	0	1	0	0
0	0	1	0	0	0	0
0	0	0	0	0	0	0
0	0	0	0	0	0	0
10	3	2	0	1	0	16
0	0	0	4	0	0	7
0	0	0	0	0	0	0
0	0	1	22	0	0	0
0	0	0	0	0	0	0
0	0	0	0	0	0	4
0	0	0	0	0	0	0
0	0	0	0	0	0	0

0	0	3	41	0	1	1
0	0	0	0	0	0	0
0	0	0	0	0	0	0
0	0	18	193	1	0	29
0	1	0	0	0	0	0
0	0	0	0	0	0	0
11	9	3	14	4	1	89
0	0	1	3	0	0	9
0	0	0	7	0	0	0
0	0	0	0	0	0	0
0	0	0	0	0	0	0
0	0	0	0	0	0	0
0	5	1	2	0	1	3
1	0	0	0	3	0	0
0	7	162	763	29	10	508
0	0	0	2	0	0	0
0	0	0	0	0	0	0
0	0	0	0	0	0	0
0	3	1	4	0	0	0
0	0	0	0	0	0	0
0	0	0	0	0	0	0
0	0	0	0	0	0	0
0	0	0	0	0	0	0
0	0	0	0	0	0	0
0	0	0	0	0	0	0
0	0	1	1	0	1	0
0	0	0	1	0	0	0

18.SUR	18.DCM	20.SUR	22.SUR	22.DCM	23.SUR	23.DCM
0	0	0	0	0	0	0
0	0	0	0	0	0	0
0	0	0	0	0	0	0
0	0	0	0	0	1	0
0	0	0	0	0	0	0
0	0	0	0	0	0	0
0	0	0	0	0	0	0
0	0	0	0	1	0	0
0	0	0	0	0	1	0
0	0	0	1	0	1	21
0	1	0	0	0	0	0
0	0	0	0	2	0	0
0	0	0	0	0	0	0
0	0	0	0	0	0	0
0	0	0	0	1	1	7
0	0	6	0	0	0	0
0	148	26	6	15	0	7
219	46	521	120	470	350	182
0	0	0	0	0	1	0
0	4	0	0	0	1	0
0	0	0	0	0	0	0
0	0	0	0	0	0	0
0	0	0	0	0	0	0
0	0	0	0	0	0	0
0	0	0	0	0	0	0
0	0	0	0	0	7	0
0	0	0	0	0	0	0
0	0	0	0	0	0	0
0	0	0	0	0	0	0
0	0	0	0	0	0	2
0	3	0	0	0	0	0
3	0	0	0	0	0	0
0	6	0	8	39	0	45
0	0	0	0	0	0	0
0	0	0	0	0	0	0
0	0	0	0	0	0	0
0	0	0	0	0	0	1
872	129	988	2	9	67	24
0	0	0	0	0	0	0
0	11	0	0	2	0	0
0	16	0	1	3	4	2
3	0	0	5	14	16	15
0	0	0	0	0	0	0
8	17	2	52	249	0	1
2	56	0	5	104	11	28
0	95	0	2	1	0	0
0	21	0	0	28	1	45

0	173	11	1	24	0	30
1	51	0	0	10	0	0
0	0	0	0	0	0	0
0	10	0	0	2	0	19
0	24	0	1	11	0	17
0	0	0	0	0	0	0
0	4	0	0	2	0	1
0	0	0	0	0	0	0
0	0	0	0	0	0	0
0	0	0	0	0	0	0
0	0	0	0	0	0	0
0	0	0	0	0	0	0
0	0	0	0	0	0	0
0	2	0	0	0	0	0
0	0	0	0	0	0	3
0	0	0	0	0	0	0
0	0	0	0	0	0	0
0	0	0	0	0	0	0
0	0	0	0	4	0	0
0	0	0	0	0	0	0
0	0	0	0	0	0	0
0	0	0	0	0	0	0
0	0	0	0	0	0	0
0	0	0	0	0	0	0
0	0	0	0	0	0	0
0	0	0	0	0	0	0
0	0	0	0	0	0	0
0	0	0	0	0	0	0
0	0	0	0	0	0	0
7	34	90	0	1	1	0
1	3	0	0	0	0	0
0	0	0	0	0	0	3
0	0	0	0	0	0	0
0	0	0	0	0	0	0
0	0	0	0	0	0	0
0	0	0	0	0	0	0
0	1	1	0	0	0	0
0	0	0	0	0	0	0
0	2	0	0	0	0	0
0	0	0	0	0	0	1
133	90	86	1441	782	1048	276
0	1	0	0	1	0	36
0	0	0	0	0	0	0
0	0	0	0	0	0	1
0	0	0	0	0	0	0
0	0	0	0	0	0	0
0	0	0	0	0	0	0
0	0	0	0	0	2	1

0	0	0	0	0	0	0
0	0	0	0	0	0	0
0	0	0	0	0	0	0
0	0	0	0	0	0	0
0	0	0	0	0	0	0
0	0	0	18	12	16	3
0	0	0	0	0	0	0
0	0	0	0	0	0	0
0	0	0	0	0	0	0
0	0	0	0	0	0	0
0	0	0	0	0	0	0
0	0	0	0	0	0	0
0	0	0	0	0	0	0
0	0	0	0	0	0	0
0	0	0	0	0	0	0
0	0	1	0	0	0	0
0	3	0	0	0	0	1
0	0	1	0	0	0	0
0	0	0	0	0	0	0
9	0	2	21	21	10	0
0	0	0	0	0	0	0
0	0	0	0	0	0	0
0	4	0	0	0	0	9
0	3	0	0	0	0	2
0	0	0	0	0	0	0
0	0	0	0	0	0	0
6	1	0	3	0	0	17
0	2	0	9	5	1	0
3	1	0	12	1	5	1
2	1	2	0	1	2	0
0	0	0	0	0	0	0
0	0	0	0	0	0	0
0	0	0	0	0	0	0
0	0	0	0	0	0	0
0	2	0	0	3	0	0
0	0	0	0	0	0	0
0	0	0	0	0	0	0
0	0	0	0	0	0	0
0	0	0	0	0	0	0
0	3	0	0	0	0	0
0	0	0	0	0	0	0
0	0	0	0	3	0	2
0	0	0	0	0	0	0
0	0	0	0	0	0	0
0	0	0	0	0	0	1
0	0	0	0	0	0	0
0	0	0	0	0	0	1
0	3	0	1	0	0	0

0	1	0	0	0	0	1
0	0	0	0	0	0	0
0	0	0	1	0	0	2
0	0	0	0	0	0	0
0	0	3	0	0	0	0
0	0	0	0	0	0	0
0	0	0	0	5	0	0
0	0	0	0	0	0	0
0	0	0	0	0	0	0
0	0	0	3	6	3	1
0	2	0	1	1	0	2
0	3	0	0	0	0	0
76	216	38	25	279	20	48
0	0	0	0	0	0	0
3	31	58	2	69	1	69
0	0	0	0	0	0	0
1	7	0	0	10	0	17
0	4	0	0	17	0	4
0	0	0	2	6	22	0
1	0	1	0	1	3	1
1	1	0	0	0	2	0
0	0	0	0	0	0	0
0	0	0	0	0	0	0
0	0	0	0	0	0	0
0	0	0	0	0	0	0
0	0	0	0	0	0	0
0	0	0	0	0	0	0
0	0	0	0	0	0	0
0	0	0	0	0	0	0
2	0	0	0	5	3	1
0	0	0	0	0	0	0
0	14	0	1	9	16	0
0	0	0	0	0	0	0
0	6	0	1	5	0	11
0	3	0	0	0	0	0
0	8	0	0	0	0	0
0	0	7	0	0	0	0
0	0	0	0	0	0	0
0	0	1	0	0	0	0
0	1	0	0	0	0	2
0	0	0	0	0	0	0
0	0	0	0	0	0	0
2	15	15	3721	1582	473	80
0	27	28	32	4	2	65
10	105	0	32	23	23	35
0	0	0	0	0	0	0
1	4	0	11	3	0	35
14	171	23	49	35	2	49

0	0	2	0	0	0	0
0	0	0	0	0	1	2
0	0	0	0	0	0	0
0	0	0	0	0	0	1
0	0	0	0	0	0	0
0	14	3	0	0	0	0
0	0	0	0	0	0	0
0	7	0	0	0	0	0
0	0	0	0	0	0	0
0	0	0	0	0	0	0
0	0	2	0	1	0	0
0	2	0	1	4	2	0
0	0	0	0	0	0	0
0	0	0	0	0	0	0
0	0	0	1	0	0	1
0	0	0	0	0	0	0
0	0	0	0	0	0	0
0	0	0	1	0	0	0
0	0	0	0	0	0	0
1	26	0	3	2	0	1
1	36	10	41	59	0	34
0	2	0	0	3	0	9
0	0	0	0	0	0	0
5	22	5	1418	127	2	128
1	2	0	0	0	1	0
0	0	1	0	0	0	0
0	0	0	0	0	0	0
0	0	0	15	1	0	0
1	0	0	0	12	0	0
0	0	0	0	1	0	0
0	0	0	0	0	0	0
0	0	0	0	0	0	0
0	0	0	1	3	0	0
0	0	0	0	0	0	0
0	0	0	0	0	0	0
0	0	0	1	71	0	0
21	12	7	98	0	1	34
0	0	0	2	0	0	0
32	0	8	6	19	0	3
0	4	11	0	0	0	0
0	0	0	0	0	0	0
0	72	0	77	0	0	36
0	0	0	0	0	0	0
0	0	0	0	0	0	0
0	0	0	0	0	0	0
0	0	0	1	10	0	2
0	0	0	6	0	0	7
0	0	3	7	6	0	4

0	0	0	0	0	0	1
0	0	0	0	0	0	0
0	0	0	0	0	0	0
0	0	0	0	0	0	0
0	0	0	0	0	0	0
0	0	0	0	0	0	0
0	0	0	0	0	0	0
0	0	0	0	0	0	2
1	1	0	0	0	0	3
0	0	0	2	0	0	2
0	0	0	0	0	0	0
0	0	0	0	0	0	4
0	0	0	0	0	0	0
0	0	0	0	0	0	0
0	0	0	0	0	0	0
0	0	0	0	0	0	0
0	0	0	0	0	0	0
0	0	0	0	0	0	0
0	0	0	0	0	6	0
0	0	0	4	84	84	0
0	0	0	0	0	0	0
1	1	0	2	0	0	49
0	0	0	0	0	0	0
41	484	483	8	244	18	86
0	0	0	0	0	1	0
1	2	5	0	1	0	0
1	2	0	1	1	1	2
0	0	0	0	0	0	0
0	0	0	0	0	0	0
0	0	0	0	0	0	0
0	0	0	0	0	0	0
0	0	0	0	0	0	0
0	10	0	0	0	0	1
0	32	0	0	3	0	0
1	2	2	0	0	0	2
0	0	0	0	0	0	0
0	0	0	0	0	0	0
0	0	0	0	0	0	0
0	2	0	0	0	0	0
0	0	0	0	0	0	1
0	0	0	0	0	0	0
0	0	0	0	0	0	0
0	3	0	0	0	1	1
0	0	0	0	0	0	0
0	0	0	0	0	0	0
0	0	0	0	0	0	0
0	5	0	0	0	0	0
0	0	0	0	0	0	0

0	0	0	0	0	0	0
0	0	0	0	0	0	0
0	0	0	0	0	0	0
0	4	0	26	70	12	18
0	1	0	0	0	0	0
0	0	0	0	0	0	0
1	34	3	771	436	677	152
0	0	0	0	0	0	0
0	0	0	0	0	0	0
0	0	0	0	0	0	0
0	0	0	0	0	0	0
2357	3424	3942	1035	6204	3478	2162
0	16	1	1	0	1	40
39	102	46	5	34	19	127
1	16	2	0	4	0	5
4	17	11	140	1173	138	125
42	141	124	14	90	48	33
6	61	15	29	120	46	174
163	24	478	1	30	120	0
1	2	0	0	2	0	0
0	40	1	0	0	1	5
0	0	0	0	0	0	0
2	0	0	0	0	0	1
0	0	0	0	0	0	0
0	0	0	0	0	0	0
0	0	0	0	0	0	0
0	0	0	0	0	0	0
0	3	0	1	0	0	18
31	130	68	2	10	2	14
0	6	0	0	0	1	3
0	5	0	0	0	0	3
0	0	0	12	63	40	74
0	0	0	0	0	0	0
0	0	0	21	45	67	25
0	0	0	0	0	0	0
1	54	54	0	0	3	0
71	109	66	37	196	132	69
0	0	0	0	0	0	0
0	0	0	0	0	0	0
0	4	0	7	29	58	239
0	0	0	0	0	0	0
0	0	1	0	0	0	0
2	22	46	0	1	2	2
0	0	0	0	0	0	0
0	0	0	0	0	0	0
0	0	0	0	0	0	2
0	0	0	0	0	0	1
0	0	0	0	0	0	0

0	0	0	0	0	0	0
0	3	1	1	1	3	0
0	0	0	0	0	0	0
5	0	0	0	0	0	0
0	5	0	0	0	0	0
0	0	0	0	0	0	0
0	0	0	0	0	0	0
0	0	0	0	0	0	0
0	7	0	0	0	0	0
0	0	0	0	0	0	0
0	0	0	0	0	0	0
0	1	0	0	3	0	0
0	0	0	0	0	0	0
0	15	0	0	4	0	0
0	0	0	0	0	0	0
0	0	0	1	0	0	0
0	0	0	0	0	0	0
0	0	0	0	0	0	0
12	0	27	1	1	11	0
0	0	0	0	0	0	0
0	0	0	0	0	0	0
9	14	13	1	4	14	4
0	0	0	0	0	0	0
0	0	0	0	0	0	0
0	0	0	0	2	0	0
1	2	2	0	0	1	2
0	0	0	0	0	0	0
0	0	0	0	0	0	0
0	0	0	0	0	0	0
0	0	0	1	3	1	3
0	0	0	0	0	0	0
2	2	0	1	4	4	2
1	7	0	0	1	2	0
6	3	3	3	10	3	1
0	0	0	0	0	0	0
0	5	0	0	0	0	0
0	4	2	0	1	0	4
0	0	0	0	0	0	0
0	0	0	0	0	0	0
2	0	1	0	1	1	0
0	0	0	0	0	0	1
0	1	0	0	0	0	1
0	0	0	0	0	0	0
0	3	0	0	0	0	1
0	0	0	0	0	0	0
0	0	0	0	0	0	0
0	0	0	0	0	0	0
0	0	0	0	0	0	0

2	2	0	0	2	0	0
1	0	0	0	2	0	0
0	0	0	0	0	0	0
0	1	0	0	0	0	0
0	2	0	0	0	0	0
0	0	0	0	0	1	0
0	0	0	0	0	0	0
0	0	0	0	0	0	0
0	0	0	1	0	0	0
3	3	4	2	8	2	4
0	0	0	0	0	0	0
0	0	0	0	0	1	0
0	0	0	0	0	0	0
0	0	0	0	0	0	0
0	0	0	0	0	0	0
0	0	0	0	0	0	0
0	0	1	0	0	0	0
0	0	0	0	0	1	0
0	0	0	0	0	1	0
0	1	0	0	0	0	0
0	0	0	0	0	0	0
0	0	0	0	0	0	0
0	0	0	0	1	0	0
0	3	0	0	0	0	0
0	2	0	0	0	1	0
0	0	0	0	0	0	0
0	2	0	0	0	0	0
0	0	0	0	0	0	0
0	0	0	0	0	0	0
1	72	0	6	73	0	2
0	0	0	0	0	0	0
0	40	0	0	3	0	1
0	0	0	0	0	0	0
0	0	0	0	0	0	0
0	0	0	0	0	0	0
0	18	0	0	4	1	3
0	159	8	1	0	0	19
1	172	0	0	1	0	1
0	0	0	0	5	0	0
0	5	0	0	1	0	2
0	0	0	0	0	0	0
0	1	0	0	0	0	0
0	242	6	0	1	3	0
0	0	0	0	0	0	0
0	0	0	0	0	0	0
0	0	0	0	0	0	0
0	0	0	0	0	0	0
2	0	0	0	0	0	0

0	19	8	1	80	32	17
0	0	0	0	0	0	0
0	6	0	0	2	0	7
7	3	6	0	4	7	2
0	0	0	0	0	0	0
0	0	0	0	0	0	0
18	8	1	62	200	91	1
0	19	0	0	0	0	6
6	82	17	0	23	4	64
3	89	56	6	119	0	27
0	17	0	0	7	0	4
0	9	0	0	10	1	44
0	0	0	0	0	0	0
0	6	0	0	0	0	0
0	0	0	0	0	0	0
0	0	0	0	0	0	0
0	0	0	0	0	0	2
0	0	0	0	0	0	0
0	38	16	2	3	28	4
0	6	0	1	11	0	23
0	0	0	0	0	0	0
0	0	0	0	0	0	0
0	1	0	0	0	0	6
0	0	0	0	0	0	0
0	0	0	0	0	0	0
0	0	0	0	0	0	0
0	4	8	1	12	7	2
0	0	0	0	0	0	0
0	0	0	0	0	0	0
1	0	0	1	0	0	2
0	0	0	0	0	0	0
0	0	0	0	0	0	0
5	0	0	0	0	0	0
5	0	0	0	0	0	0
0	0	0	0	3	0	0
0	0	0	0	0	0	0
0	0	0	0	0	0	0
0	0	0	0	0	0	0
0	0	0	0	0	0	0
0	0	0	2	0	0	0
1	0	0	0	0	0	0
0	0	0	0	0	0	0
0	9	0	0	7	2	50
0	11	0	1	0	1	11
0	0	0	0	0	0	0
12	9	6	0	0	7	0
0	0	0	1	1	0	2
0	0	0	0	0	0	0

0	0	0	0	6	0	0
10	12	3	0	0	0	0
4930	1740	19	48	49	34	35
0	0	0	0	0	0	0
0	0	0	0	0	0	0
5	0	0	0	1	1	0
0	0	0	0	0	0	0
0	0	0	0	0	0	0
0	0	0	0	0	0	0
0	0	0	0	2	1	3
0	0	0	0	0	0	0
1	0	0	0	0	1	0
0	0	0	0	0	0	0
0	0	0	0	0	0	0
0	0	0	0	0	0	0
0	0	0	0	0	0	0
0	0	0	0	0	0	0
0	0	0	0	0	0	0
0	0	0	0	6	0	0
4	2	0	0	0	0	0
0	0	0	0	0	0	0
0	0	0	0	0	0	0
0	0	0	0	0	0	0
0	11	0	0	10	2	21
0	1	0	3	3	0	1
0	1	0	0	1	0	0
0	0	0	0	0	0	0
0	0	0	0	0	0	14
0	0	18	0	0	0	0
0	0	0	0	0	0	0
0	0	0	0	0	0	0
0	0	0	0	0	0	1
0	0	0	0	0	0	0
0	0	0	0	0	0	0
0	0	0	0	0	0	0
0	0	0	0	0	0	0
0	0	1	0	1	1	0
0	0	0	0	0	0	0
0	0	0	0	0	0	0
0	0	0	0	0	0	0
26	25	23	119	145	158	91
0	0	0	0	0	0	0
0	0	0	0	0	0	0
502	1125	372	562	2013	1309	2024
55	39	91	7	119	46	29
570	880	2154	4546	4490	861	128
283	511	775	254	658	376	131
46	64	30	44	216	195	104

6	7	1	0	2	0	0
17	36	79	24	50	370	31
8	0	0	8	59	4	46
3	6	20	0	4	4	31
3	9	47	1	10	5	4
0	3	0	0	1	2	0
2	1	0	0	11	5	39
8	8	0	6	31	64	0
0	9	12	7	6	4	3
1	0	0	3	1	1	0
5	19	4	1	10	0	4
0	3	3	1	18	3	32
1	1	0	0	13	7	0
1	0	1	10	55	21	29
4	5	18	0	4	0	0
3	3	0	2	3	1	18
1	0	0	2	17	17	17
1	13	0	4	47	1	38
0	0	0	0	0	0	0
1	7	3	4	2	13	4
5	5	7	1	2	0	1
1	2	0	7	6	3	95
0	0	0	0	0	0	0
0	0	0	0	0	0	0
0	0	4	0	0	0	2
0	1	0	0	0	3	0
0	4	2	0	1	1	0
0	0	0	3	10	0	4
0	0	0	0	0	0	0
0	1	0	0	0	0	0
0	0	0	0	0	0	0
0	0	0	0	0	0	0
0	0	0	0	0	1	0
0	2	0	0	0	0	0
0	5	0	0	18	0	0
3	0	0	0	2	1	0
0	14	0	0	10	0	0
4	1	2	5	3	3	0
0	0	0	0	0	0	0
0	0	0	0	0	0	0
0	0	0	0	0	0	0
0	0	0	0	0	0	0
0	0	0	0	2	0	8
0	0	0	0	0	0	0
0	0	0	0	0	2	0
0	0	0	0	0	0	0
0	0	4	0	1	1	0
0	0	0	0	0	0	0

0	3	0	0	0	0	1
0	0	1	0	0	0	0
0	0	0	0	0	0	0
0	0	0	0	0	0	0
0	0	1	0	0	0	0
7	0	10	1	7	21	4
0	0	0	0	0	0	0
13	226	61	27	504	56	87
1	1	0	0	0	1	1
8	1	0	8	2	0	0
0	0	19	5	0	0	0
0	0	0	0	0	0	0
0	0	0	0	1	0	5
0	0	0	0	0	0	0
0	0	0	0	0	0	1
0	0	0	0	0	0	3
0	0	0	0	0	0	0
0	0	0	0	0	0	0
0	0	0	0	0	0	0
0	1	0	0	0	0	0
0	0	0	0	0	0	0
0	0	0	0	0	0	0
0	0	0	0	0	0	0
0	0	2	0	1	0	0
0	0	0	0	0	0	0
0	0	0	0	0	0	0
1	0	0	0	0	0	0
0	0	0	0	0	0	0
0	0	0	0	0	0	0
0	0	0	0	0	0	0
4	0	12	0	7	0	13
11	0	0	8	15	25	1
0	0	0	5	0	0	0
0	0	0	0	0	0	0
0	0	0	0	0	0	0
6	104	30	325	218	25	25
122	244	156	177	56	139	11
16	0	0	242	2	611	1
0	0	0	197	9	234	8
0	0	0	0	0	0	0
0	2	0	1	2	0	0
0	0	0	0	0	0	0
0	0	0	1	0	0	0
0	0	0	2	0	1	0
0	0	2	0	0	0	0
0	0	0	0	0	0	0
0	0	0	0	0	0	0
0	0	0	0	3	0	0

0	0	0	0	0	0	0
0	0	0	0	0	0	0
0	0	0	0	0	0	0
0	0	0	0	0	0	0
0	0	0	0	0	0	0
0	4	0	0	0	0	0
0	0	0	0	0	0	0
0	0	0	2	0	0	0
0	0	0	0	0	0	0
0	0	8	9	0	1	0
164	3	3	3	0	2	0
0	0	0	0	1	3	0
0	0	0	325	25	2	1
0	0	0	0	0	0	0
142	18	28	568	720	457	16
6	11	23	1	7	1	5
0	0	0	0	0	0	0
0	0	0	382	143	11	0
2	19	0	1	28	1	80
0	0	0	0	0	0	0
0	0	0	48	57	5	13
0	0	1	0	0	0	0
0	33	1	1	10	5	35
9	0	1	1	1	2	1
0	0	0	0	0	0	5
4	0	0	0	2	0	0
15	1	16	1	8	9	3
0	0	0	0	0	0	4
0	0	1	0	0	0	0
5	1	0	0	3	1	16
0	0	0	0	0	0	0
0	0	0	0	0	0	0
0	0	0	0	0	0	0
0	0	0	4	12	0	0
0	0	0	0	0	0	0
0	1	0	1	0	1	0
0	0	0	0	0	0	0
0	0	0	0	0	0	0
0	0	0	0	0	0	0
2	4	1	0	3	1	1
0	0	0	0	0	0	0
0	0	0	0	0	0	0
0	0	0	0	0	0	0
0	0	0	0	0	0	0
0	0	0	0	0	0	0
0	0	0	0	0	0	0
0	0	0	0	0	0	0
0	0	0	0	0	0	0

0	0	0	0	0	0	0
167	125	150	230	238	476	117
37	11	13	33	99	39	2
38	25	31	0	4	11	0
16	0	0	42	24	65	3
0	0	0	0	0	0	0
50	1	0	11	6	11	1
15	3	0	12	4	20	0
38	2	57	10	3	24	14
0	23	0	0	12	2	6
1	1	2	0	0	0	3
0	6	0	0	0	1	44
18	0	0	0	0	10	0
0	0	0	2	3	5	35
3	0	0	6	5	7	2
0	5	0	0	0	0	0
0	0	0	0	0	0	0
0	0	0	0	0	0	8
0	1	0	4	13	4	5
0	0	0	0	0	0	0
2	4	1	3	1	6	2
0	0	0	0	0	0	0
0	0	1	0	0	0	0
0	1	0	0	0	0	2
0	0	0	0	0	0	0
0	0	0	0	0	0	0
0	0	0	0	0	0	0
0	0	0	0	0	0	0
0	0	0	0	0	0	0
0	0	0	0	0	0	0
2	0	0	0	0	0	0
0	0	0	0	0	0	0
0	7	0	196	733	14	186
42	58	14	30	66	52	34
101	66	7	200	659	387	1
168	7	11	9	31	27	0
3	0	0	41	8	5	0
0	0	0	1	0	0	0
12	97	42	19	68	42	39
18	18	45	114	164	122	133
45	111	18	6	10	8	32
31	5	3	85	113	199	64
0	8	0	0	0	0	0
1	2	0	0	0	3	9
1	0	0	12	10	0	2
6	0	0	0	0	0	2
0	0	5	3	1	0	0
0	1	0	2	0	0	1

0	0	0	0	0	0	1
0	0	0	0	0	0	0
0	0	11	0	0	0	0
0	0	0	0	0	0	0
0	0	0	0	0	0	0
0	0	0	0	0	0	0
0	0	0	0	0	0	0
0	0	0	0	0	0	0
0	0	0	0	0	0	0
0	0	0	0	0	0	0
0	0	0	0	0	0	0
0	0	0	0	0	0	0
0	0	0	0	0	0	0
0	0	0	1	1	0	5
0	0	0	0	2	4	0
0	0	0	0	0	0	0
0	0	0	0	0	0	0
0	0	0	0	0	0	0
0	0	0	0	0	0	0
0	0	0	0	0	0	0
0	0	0	0	0	0	0
0	0	0	0	0	0	0
0	0	0	0	0	0	0
0	0	0	0	0	0	0
0	0	0	0	0	0	0
0	0	0	0	0	0	0
0	0	0	0	0	0	3
0	5	0	0	0	0	0
0	0	0	0	0	0	0
15	8	4	1	5	8	0
4	3	0	3	0	5	0
0	0	0	0	0	0	0
1	3	0	6	7	4	0
4	3	0	7	7	1	0
0	0	0	0	0	1	0
0	1	0	0	0	0	0
0	0	0	0	0	0	0
0	0	0	0	0	0	0
0	0	0	0	0	0	0
0	1	0	5	4	1	0
0	0	0	0	0	0	0
5	5	4	0	0	0	0
0	0	0	0	0	0	0
2	6	2	0	2	1	0
0	0	0	14	6	0	0
0	0	0	0	0	0	0
0	0	0	0	0	0	0
0	0	0	0	0	0	0

3492	6190	3054	2416	3248	9283	349
29	46	15	51	188	31	2
0	0	0	0	0	0	0
0	0	0	0	0	0	0
1	0	0	0	0	0	0
0	3	1	0	1	1	0
0	0	0	0	0	0	0
0	0	0	0	0	0	0
0	0	0	0	0	1	0
0	2	0	0	0	0	0
0	0	0	0	0	0	0
0	0	0	0	0	0	0
41	61	7	65	64	72	3
0	133	0	184	44	41	25
0	0	0	0	0	0	0
0	0	0	0	0	0	0
0	0	0	0	0	0	0
0	0	0	0	0	0	0
0	0	0	0	6	0	0
98	126	28	90	84	415	1
2	4	0	1	0	0	0
0	0	0	0	0	0	0
0	0	0	0	0	5	0
0	1	2	0	0	0	0
48	53	11	107	220	44	29
0	0	0	0	0	0	0
0	0	0	0	0	0	0
0	3	0	86	8	509	518
6	25	19	1	4	1	1
381	46	277	52	113	186	30
0	5	4	52	15	128	11
0	0	0	0	0	1	1
153	17	28	16	6	17	1
0	0	4	1	0	2	0
57	6	8	19	14	46	2
23	4	4	1	0	0	0
10	51	8	22	34	3	0
0	0	0	0	2	0	11
0	0	0	0	0	0	0
0	1	0	0	0	0	0
0	0	0	0	0	0	0
0	0	0	0	0	0	0
0	0	0	0	0	0	0
53	33	0	2	1	53	1
72	151	149	342	334	18	45
2	4	0	31	54	21	27
0	0	1	0	0	0	0
11	0	0	0	0	0	0

0	0	0	0	0	0	0
0	0	0	0	0	0	0
0	0	0	0	0	1	0
0	0	0	0	0	6	0
0	0	0	0	0	0	0
1	0	0	0	0	0	0
0	0	0	1	0	0	0
0	0	0	3	0	1	0
4	0	0	1	0	3	0
1	79	3	3	6	7	0
0	0	0	0	0	0	0
0	0	1	3	3	7	0
0	0	0	0	0	0	0
0	0	0	0	0	0	0
0	0	0	0	0	0	0
155	449	2476	3803	4929	7031	216
34	657	195	48	208	3	49
0	3	0	103	57	7	0
0	0	1	0	0	0	0
0	0	0	0	0	0	0
1	0	0	13	7	16	4
0	0	0	4	0	0	0
0	0	0	0	0	0	0
0	0	0	0	0	0	0
1	3	0	17	31	63	3
0	0	0	2	1	2	0
2	0	0	2	2	6	0
0	0	1	1	3	1	0
15	29	5	136	48	600	8
66	102	47	93	117	115	7
65	85	8	422	226	203	1
71	67	16	70	81	40	64
50	9	17	18	32	75	17
0	0	0	0	0	0	0
0	0	0	0	0	0	0
0	0	0	0	0	0	0
0	0	0	0	0	0	0
0	0	0	0	0	0	0
582	972	214	3132	3535	1356	420
32	248	35	81	88	252	72
11	283	1	8	32	22	7
0	0	0	0	0	0	0
39	123	88	17	20	21	11
0	0	0	0	0	0	0
0	0	0	0	0	0	0
1	5	0	0	0	0	0
0	0	0	0	0	0	0
0	2	0	0	0	0	0

0	0	0	0	1	0	0
0	0	0	0	0	0	0
0	0	0	1	3	0	0
0	0	0	0	0	0	0
0	0	0	0	0	1	0
0	0	0	0	0	0	0
0	0	0	0	0	0	0
0	0	0	0	0	0	0
0	0	0	0	0	0	0
0	0	0	1	0	0	0
0	2	0	0	0	0	2
0	1	0	59	93	145	22
0	0	0	0	0	0	0
0	0	0	0	0	0	0
24	3	73	9	29	62	14
0	0	0	6	0	143	0
0	1	0	0	0	0	0
10	8	8	3	22	5	1
3	77	11	2	5	6	0
0	0	0	6	3	3	0
2	0	0	0	0	0	0
0	0	0	0	0	0	0
0	0	0	0	0	3	3
0	0	0	0	0	0	0
0	0	0	2	0	5	0
1815	859	546	2185	1099	4681	20
995	1262	443	21	25	23	91
5	13	3	8	1	15	0
0	0	0	34	34	6	0
4	2	0	2	1	5	0
3	707	87	2	12	7	38
10	14	0	0	1	2	0
0	0	0	0	0	0	0
0	0	0	0	0	0	0
0	0	0	0	0	0	0
0	0	0	0	0	0	0
0	0	0	0	0	0	0
0	0	0	0	0	0	0
0	0	0	0	0	0	0
0	0	0	0	0	0	0
0	0	0	0	0	0	0
0	0	0	0	0	0	0
0	3	0	29	17	1	19
5	0	0	46	89	31	0
1	0	0	0	7	1	0
0	0	0	0	0	0	0
0	0	0	0	0	0	0
0	0	0	0	0	3	0

0	0	0	0	0	0	0
0	0	0	0	0	0	0
192	477	427	309	1061	59	113
50	2	50	87	14	254	1
11	302	10	97	263	56	62
0	0	0	0	0	0	37
25	366	29	74	412	18	95
76	218	163	12	107	3	68
0	34	0	139	816	22	128
56	10	27	110	11	254	25
0	0	3	0	1	0	0
0	10	0	62	595	3	112
10	72	9	13	76	62	7
0	0	2	0	0	0	0
42	131	53	1	9	5	15
0	7	1	0	5	0	1
0	2	0	0	0	0	0
0	0	0	0	0	0	0
117	72	188	2	74	23	2
0	37	0	0	0	2	4
6	1	0	0	0	0	4
0	0	0	4	2	1	0
0	65	0	3	4	0	2
0	67	0	0	0	0	3
3	17	9	0	0	1	0
12	6	28	11	35	14	24
0	52	0	0	0	0	0
0	0	0	0	0	0	0
0	4	12	0	0	0	0
0	0	0	0	0	0	0
0	0	0	0	0	0	0
0	0	0	0	0	0	0
0	0	0	0	0	0	0
1	0	0	0	0	0	0
0	0	0	0	0	0	0
0	1	0	0	0	0	0
1	1	0	0	0	1	6
1	7	0	3	20	6	2
0	0	0	0	0	0	0
0	6	0	0	0	0	0
0	0	0	0	0	0	0
0	0	0	0	0	0	0
0	2	4	1	0	0	1
0	0	0	0	0	0	0
0	0	0	0	0	0	0
0	0	0	0	0	0	0
0	0	0	0	0	0	0
0	0	0	0	0	0	0

0	0	0	0	0	0	0
0	0	0	0	0	0	0
0	0	0	1	0	1	2
0	0	0	0	0	0	0
0	0	0	0	0	0	0
0	0	0	0	0	0	0
0	0	0	0	0	0	0
0	0	0	0	0	0	0
1	0	0	0	0	0	0
0	0	0	1	1	0	0
0	3	0	0	0	0	0
0	0	0	0	0	0	0
0	1	0	0	0	0	0
0	3	0	0	0	0	0
0	0	0	0	0	0	0
0	0	0	0	0	0	0
0	0	0	0	0	0	0
0	0	0	0	0	0	0
0	0	0	0	0	0	4
0	0	0	0	0	0	0
0	0	0	0	0	0	0
0	0	0	0	0	0	0
0	0	0	0	0	0	0
0	0	0	0	0	0	0
0	0	0	0	0	0	0
0	0	0	0	0	0	0
0	0	0	0	0	0	1
0	0	0	0	0	0	0
0	0	0	0	0	0	0
0	0	0	1	0	0	0
0	0	0	0	0	0	0
0	0	0	0	0	0	0
0	0	0	0	0	0	0
0	0	0	0	0	0	0
0	0	0	0	0	0	0
0	1	0	0	0	0	0
0	0	0	0	0	0	0
0	0	0	0	0	0	0
0	0	0	0	0	0	0
0	0	0	0	0	0	0
0	0	0	0	0	0	0
0	0	0	0	0	0	0
0	0	0	0	0	0	0
0	0	0	0	0	0	0
27	6	3	144	52	97	51
2	11	0	8	29	10	19
0	0	0	0	0	0	0
0	0	1	0	0	0	0

0	0	0	0	0	0	0
0	0	0	0	0	0	0
0	0	0	0	0	0	0
65	35	210	12	254	116	252
10	1	1	0	0	1	0
11	0	0	3	10	0	0
0	0	0	0	0	0	0
0	0	0	0	0	0	0
274	594	87	804	782	2408	368
4104	2292	2254	4555	5696	6899	2562
0	0	4	11	41	60	67
85	0	8	0	0	1	0
148	154	254	167	141	808	105
72	3	90	51	12	154	50
0	21	0	2	30	0	14
8	19	20	7	23	30	7
8	2	0	0	8	0	0
9	8	16	0	0	2	1
26	1	10	4	0	13	0
49	11	5	25	20	38	27
39	6	17	54	31	75	18
26	5	20	21	6	20	12
0	10	0	0	3	1	0
0	0	0	0	4	0	0
0	0	0	8	38	1	47
3	7	11	2	2	29	1
1	42	11	0	0	2	1
46	11	13	33	35	24	4
2	2	0	7	18	4	2
0	0	0	0	0	0	0
2	0	1	2	16	5	23
5	0	0	0	0	6	0
7	3	1	6	7	10	2
0	5	0	1	3	1	0
0	0	0	0	0	1	0
0	0	0	1	36	0	0
1	3	0	0	5	1	1
3	12	1	4	9	1	2
0	1	0	0	0	0	4
0	0	0	2	2	0	0
0	0	0	0	0	0	0
0	0	0	4	0	11	0
0	0	1	0	0	0	0
0	0	0	0	0	6	0
1	0	0	0	0	0	0
1	0	0	0	0	0	0
0	2	1	0	0	1	0
0	0	0	0	0	0	0

0	0	0	0	0	0	0
9	2	2	4	9	19	3
0	0	0	0	0	0	0
0	1	4	0	0	0	0
0	0	0	0	0	0	0
0	0	0	2	0	0	0
0	0	0	0	0	0	0
27	11	9	43	33	23	10
0	0	0	0	0	0	0
0	0	0	0	0	0	0
0	1	0	1	1	0	0
3	0	1	1	3	4	0
0	0	0	0	0	0	0
0	0	1	4	2	0	2
0	0	8	0	0	0	0
1	1	1	7	3	2	1
0	0	0	0	0	0	1
0	0	0	0	0	0	0
0	1	0	0	0	0	0
0	0	0	0	2	1	0
0	0	0	0	0	0	0
0	0	0	0	0	0	0
1	1	0	1	0	1	0
1	0	0	2	4	0	0
0	0	0	0	0	4	0
0	0	0	0	0	0	0
0	0	0	0	0	0	0
0	0	0	0	0	0	0
0	0	0	0	0	0	0
1	0	0	0	0	0	0
0	0	0	0	0	0	0
0	0	0	0	0	0	0
1	0	0	0	0	0	0
0	0	0	0	0	0	0
0	0	0	0	0	0	0
0	0	0	0	0	0	0
0	0	0	0	0	0	0
0	0	0	0	0	0	0
0	0	0	0	0	0	0
0	0	0	0	0	0	0
0	0	0	0	0	0	0
0	0	0	0	0	0	0
6	1	2	4	3	0	0
0	0	0	0	1	0	1
0	0	0	0	1	0	0
0	1	0	0	0	0	0
0	0	0	0	0	0	0
0	0	0	1	0	0	0

0	0	0	0	0	0	0
995	189	814	465	304	1217	484
676	257	117	97	89	154	63
58	17	89	59	20	155	44
228	1	40	0	0	0	0
107	40	291	24	26	134	23
47	10	89	255	10	67	19
43	0	8	3	6	5	1
17	0	25	0	0	0	6
91	32	116	29	64	193	93
2	2	5	0	0	0	1
2	6	0	1	1	2	5
1	0	4	6	1	5	1
5	2	5	2	0	7	0
0	0	0	2	3	5	1
0	0	0	0	0	0	0
32	21	21	14	15	24	16
4	1	0	0	7	2	2
1	1	3	1	0	0	3
1	0	0	3	0	0	2
2	0	0	3	6	3	1
4	1	2	0	0	5	0
11	1	0	3	2	8	2
0	0	0	0	0	0	0
0	0	0	0	7	2	0
1	0	0	0	1	3	0
0	0	0	0	0	0	0
0	1	0	1	1	1	0
2	0	0	1	0	0	0
2	1	0	1	0	5	0
1	0	0	0	0	0	0
0	0	0	0	0	0	0
0	0	0	0	0	0	0
0	0	1	0	1	2	0
0	0	0	0	0	1	0
0	0	0	0	0	0	0
1004	38	3745	1398	172	742	37
23	18	26	1	19	18	51
0	0	0	0	0	0	0
0	0	0	0	0	0	0
1	0	1	0	0	0	0
0	6	0	0	0	0	0
0	0	0	9	0	5	0
0	0	0	0	0	0	0
0	0	0	0	0	0	0
1	0	0	0	0	0	0
1	0	0	0	0	0	0
0	0	0	0	0	0	0

0	0	0	3	0	1	0
0	0	0	0	0	0	0
0	0	1	0	0	0	0
0	0	0	0	0	0	0
0	0	0	0	0	0	0
0	0	0	0	0	0	0
0	0	0	0	0	0	0
0	0	2	0	0	0	0
0	0	0	0	0	0	0
0	0	0	0	0	0	0
0	0	0	1	0	0	0
0	0	0	0	0	0	0
0	0	0	0	0	0	0
0	0	0	0	0	1	0
0	0	0	0	0	0	0
0	0	0	0	0	0	0
0	0	0	0	0	0	0
0	0	0	0	0	0	0
0	0	0	0	0	0	0
0	0	0	0	0	0	0
0	0	0	0	0	0	0
0	61	0	47	472	2	30
0	7	0	2	46	2	50
0	0	0	0	0	0	0
0	0	0	0	0	0	0
0	0	0	0	0	0	0
0	0	0	0	0	0	0
0	0	0	0	0	0	0
7	0	2	1	0	0	0
1	0	0	0	3	5	0
1	0	0	1	10	2	1
0	0	0	0	0	0	0
0	0	0	0	0	0	0
158	22	144	12	6	21	12
0	0	0	0	0	0	2
31	1	11	29	35	97	0
1	6	0	62	113	2	0
0	0	0	0	0	0	0
0	0	0	52	1	5	1
0	0	0	35	15	1	0
0	0	0	0	0	0	0
0	0	0	1	1	0	0
22	2	0	52	15	116	13
2	2	0	2	7	1	0
9	63	13	1	10	9	35
0	2	0	0	0	0	0
0	0	0	0	0	0	0
55	25	21	7	25	8	45

0	0	0	0	0	0	0
264	23	55	45	99	180	74
539	90	270	82	59	129	18
0	0	0	1	3	3	7
0	6	0	2	1	0	0
0	0	0	0	0	0	0
0	0	0	0	0	0	0
0	0	0	0	0	0	0
0	0	0	0	0	0	0
135	6	250	39	112	115	77
160	31	74	68	94	71	39
11	68	188	11	39	1	49
0	97	0	0	0	0	0
0	0	0	0	0	2	7
0	0	0	0	0	0	0
0	0	0	0	0	0	0
0	0	0	0	0	0	0
0	7	0	0	0	0	0
0	0	0	0	0	0	0
0	0	0	0	0	0	0
0	0	0	0	0	0	0
0	0	0	0	0	0	0
0	0	0	0	0	0	0
0	0	0	0	0	0	0
0	0	0	0	0	0	0
0	0	0	0	0	0	0
0	0	0	0	0	0	0
0	0	0	0	0	0	0
0	0	0	0	0	0	0
0	0	0	0	0	0	0
0	0	0	0	0	0	0
3	0	11	1	6	1	0
0	0	0	1	0	1	0
0	0	0	0	0	0	0
1	0	0	0	20	0	4
8	1	0	0	6	0	0
1	0	9	0	0	2	0
0	0	1	0	0	1	0
0	0	0	0	0	0	0
0	0	0	0	0	0	0
0	4	0	1	78	1	64
0	3	0	0	7	4	13
0	0	0	0	0	0	0
0	0	0	0	0	0	6
0	0	0	0	0	0	0
4	5	0	0	0	0	3
0	0	0	0	0	0	0
0	0	0	0	0	0	0

1	3	0	0	0	1	2
0	0	0	0	0	0	0
0	0	0	0	0	0	0
0	10	0	0	0	0	9
0	0	0	0	1	0	0
0	0	0	0	0	0	0
1	157	10	4	45	0	5
0	0	0	1	0	0	1
0	0	0	0	6	0	0
0	0	0	0	0	0	0
0	0	0	0	0	0	0
0	0	0	0	0	0	0
0	6	0	0	1	0	0
0	0	0	0	0	0	0
0	1227	0	4	11	20	516
0	0	0	0	0	0	0
0	1	0	0	0	0	0
0	0	0	0	0	0	0
0	0	0	0	0	0	3
0	0	0	0	0	0	0
0	0	0	0	0	0	0
0	0	0	0	0	0	0
0	1	0	0	0	0	0
0	0	0	0	0	0	0
0	0	0	0	0	0	0
0	0	0	0	0	0	0
0	0	0	0	0	0	0

24.SUR	25.SUR	25.DCM	26.SUR	30.SUR	30.DCM	31.SUR
0	0	0	0	0	0	0
0	0	0	0	0	0	0
0	0	0	0	0	0	0
0	4	0	0	0	0	0
0	0	0	0	0	0	0
0	0	0	0	0	0	0
0	0	0	0	0	0	0
0	0	1	0	0	4	5
0	0	0	2	0	0	0
0	0	0	0	0	1	0
0	0	0	0	0	0	0
0	0	0	0	0	0	0
0	0	0	0	0	0	0
1	0	0	0	0	0	0
0	1	4	0	0	0	0
0	0	0	0	1	0	0
0	0	133	1	1	1	4
349	420	104	704	71	50	260
1	0	0	18	0	0	0
0	0	3	0	0	5	0
0	0	0	0	0	2	0
0	0	0	0	0	0	0
1	0	0	0	0	0	0
0	0	0	0	0	0	0
0	0	0	0	0	0	0
0	0	0	0	0	0	0
0	0	0	0	0	0	0
0	0	0	0	1	0	0
0	0	1	0	0	0	0
0	0	2	1	0	1	0
0	0	1	0	0	0	0
0	0	0	0	0	0	0
0	0	14	0	0	0	0
0	0	0	0	0	0	0
0	0	0	0	0	0	0
0	0	0	0	0	0	0
2	0	0	0	2	0	0
127	429	87	264	147	177	646
1	1	0	0	0	0	0
0	0	25	0	0	2233	0
0	0	43	0	0	0	0
0	2	12	0	0	0	0
0	0	0	0	0	0	18
31	0	9	73	0	0	58
0	16	28	0	0	23	0
0	0	10	0	0	0	0
0	0	40	0	0	0	17

0	0	50	0	3	31	0
0	0	6	0	0	32	0
0	0	0	0	0	0	0
0	1	56	0	0	0	0
0	0	9	0	0	2	0
0	0	0	0	0	0	0
0	0	5	0	0	0	0
0	0	0	0	0	0	3
0	0	0	0	0	0	0
0	0	0	0	0	0	0
0	0	0	0	0	0	0
0	0	0	0	0	0	0
0	0	0	0	0	0	0
0	0	0	17	0	2	0
0	0	7	0	1	0	0
0	0	0	0	0	0	0
0	0	0	0	0	0	1
0	0	0	0	0	0	0
19	0	0	0	0	0	0
0	0	0	0	0	0	0
0	0	0	0	0	0	0
0	0	0	0	0	0	0
0	0	0	0	0	0	0
0	0	0	0	0	0	0
0	0	0	0	0	0	0
0	0	0	0	0	0	0
0	0	0	0	0	0	0
0	0	0	0	0	0	0
0	0	0	0	0	0	0
17	0	0	10	2	2	0
0	0	0	0	0	0	0
0	0	0	0	0	1	0
0	0	0	0	0	0	0
0	0	0	0	0	0	0
0	0	0	0	0	0	0
0	0	0	0	1	0	0
0	0	0	0	2	5	0
0	0	0	0	0	0	0
0	0	0	0	0	0	0
14	0	4	2	359	405	406
4471	426	279	624	22	48	92
5	0	4	0	3	1	1804
0	0	4	1	0	0	0
0	0	0	0	0	0	2
0	0	0	0	0	0	0
0	0	0	0	0	0	0
0	0	0	0	0	0	0
1	3	2	0	0	0	1

0	0	0	0	0	0	0
0	0	0	0	0	0	0
0	0	0	0	0	0	0
0	0	0	0	0	0	17
0	0	0	0	0	0	0
18	3	2	4	0	12	4
2	0	0	0	0	0	0
0	0	0	0	0	0	0
0	0	0	0	0	0	0
0	0	0	0	0	0	0
0	0	0	0	0	0	0
0	0	0	0	0	0	0
0	0	0	0	0	0	0
0	0	0	0	0	0	0
1	0	0	0	0	0	0
0	0	0	0	0	0	0
0	0	3	0	0	2	0
0	0	0	0	0	0	0
0	0	0	0	0	0	0
2	0	1	0	3	2	0
0	0	0	0	0	0	0
0	0	0	0	0	0	0
0	0	614	8	0	0	0
0	0	5	0	0	1	0
0	0	3	0	0	0	0
11	1	0	0	51	29	0
0	0	8	44	0	4	0
0	5	4	0	0	3	2
0	0	0	0	0	15	1
0	1	0	5	0	0	0
0	0	0	0	0	0	0
0	0	0	0	0	0	0
0	0	1	0	0	0	0
0	0	0	0	0	0	0
0	0	7	0	0	2	0
0	0	1	0	0	0	0
0	0	0	0	0	0	25
0	0	0	0	0	0	0
0	0	0	0	0	0	0
0	0	2	0	0	0	0
0	0	0	0	0	0	0
0	0	5	4	0	0	0
0	0	2	0	0	0	0
0	0	0	0	2	106	0
0	0	0	0	0	3	0
0	0	0	0	0	0	0
0	0	2	0	0	0	0
0	0	0	0	0	0	0

0	0	0	0	0	1	0
0	0	0	0	0	0	0
0	0	316	0	0	0	10
0	0	0	46	0	0	0
0	0	0	0	0	0	0
0	0	0	0	0	0	0
0	0	0	0	0	0	0
0	0	0	0	0	0	0
0	0	0	0	0	0	0
0	0	0	0	0	0	28
0	1	9	0	0	2	3
0	0	6	0	0	3	0
33	5	20	47	0	32	11
0	0	0	0	0	0	0
0	2	111	0	0	97	0
0	0	0	0	0	0	0
0	0	16	0	0	8	0
0	0	10	0	0	5	0
6	16	0	0	5	0	18
14	2	4	27	1	3	2
1	2	0	2	0	0	1
0	0	0	0	0	0	0
0	0	0	0	0	0	0
0	0	14	0	0	0	0
0	0	0	0	0	0	0
0	0	0	0	0	0	0
0	0	0	0	0	0	0
0	0	0	0	0	0	0
0	0	0	0	0	0	0
0	0	0	0	1	1	0
0	0	0	0	0	0	1
8	2	1	0	0	6	3
0	0	0	0	0	0	8
0	0	19	0	0	0	0
0	0	4	0	0	0	0
0	0	0	0	0	0	0
0	0	0	0	0	0	0
0	0	0	0	0	0	0
0	0	0	0	0	0	0
13	0	0	0	0	0	0
0	0	0	0	0	0	0
0	0	0	0	0	0	0
631	249	76	504	0	7	85
3	53	32	10	1	3	0
18	64	100	3	12	4	0
0	0	0	0	0	0	0
0	1	22	0	0	0	0
7	50	62	0	1	1	0

0	0	0	0	0	0	0
0	0	0	0	0	0	6
0	0	0	0	0	0	0
0	0	0	0	0	0	0
0	0	6	0	0	0	0
0	0	0	0	0	0	0
0	0	0	0	0	0	0
0	0	0	3	0	0	0
0	0	0	0	0	0	8
0	0	0	0	0	0	0
1	0	17	0	0	0	0
0	0	0	0	0	0	0
0	0	0	0	0	0	11
0	0	0	0	0	0	1
0	0	1	0	0	0	0
0	0	0	0	0	0	0
0	0	0	0	0	0	0
0	0	0	0	0	0	0
0	0	0	0	0	0	0
0	196	10	0	0	1	10
0	9	6	0	0	0	0
0	0	13	0	0	0	0
0	0	0	0	0	0	0
13	10	29	5	12	63	599
0	0	1	0	0	0	3
0	0	0	0	0	0	0
0	0	0	0	0	0	1
0	1	1	0	0	0	0
0	0	0	0	0	0	0
0	0	0	0	0	2	0
0	0	0	0	0	0	0
0	0	0	0	0	0	0
0	0	0	0	0	0	0
0	0	0	0	0	0	0
0	0	0	0	0	0	0
0	0	0	0	0	0	0
6	21	1	3	0	18	104
0	1	0	0	0	0	2
0	2	2	25	0	0	19
0	0	0	0	0	0	4
0	2	1	0	0	0	0
0	3	54	0	0	12	0
0	0	0	0	0	0	0
0	0	0	0	0	0	0
0	0	0	0	0	0	1
0	0	0	0	0	0	0
0	0	12	0	0	0	0
0	0	12	0	0	0	0

0	0	1	0	0	0	0
0	0	0	0	0	0	0
0	0	0	0	0	0	1
1	0	0	0	0	0	0
0	0	0	0	0	0	0
0	0	0	0	0	0	0
0	0	0	0	0	0	0
0	0	0	0	0	0	0
0	0	0	0	0	0	0
0	0	0	0	0	0	0
0	0	0	0	0	0	0
0	0	0	0	0	0	0
0	0	0	0	0	0	0
0	0	0	0	0	0	0
0	0	0	0	0	0	0
0	0	0	0	0	0	0
0	0	0	0	0	0	0
0	0	0	0	0	0	1
3	1	0	0	1	1	0
11	10	0	149	0	0	0
0	0	0	0	0	0	0
0	0	0	0	2	1	0
0	0	0	0	0	0	0
23	17	335	1	4	893	94
0	0	0	0	0	0	1
0	0	0	0	0	6	4
0	0	4	0	0	2	6
0	0	0	0	0	0	0
0	0	0	0	0	0	1
0	0	2	0	0	0	0
0	0	0	0	0	0	0
0	0	0	0	0	0	0
0	0	31	0	0	8	0
0	0	0	27	0	16	0
0	0	18	0	0	0	0
0	0	0	0	0	0	0
0	0	0	0	0	0	0
0	0	0	0	0	0	0
0	0	0	0	0	0	0
0	0	1	0	0	0	1
0	0	0	0	0	1	0
0	0	0	0	0	0	0
0	0	0	0	0	1	0
0	0	0	0	0	0	0
0	0	0	0	0	0	0
0	0	0	0	0	0	0
0	0	14	0	0	1	2
0	0	0	0	0	0	0

0	0	7	0	0	0	0
0	0	1	0	0	0	0
0	0	0	0	0	0	0
8	77	18	5	0	0	1
0	0	0	0	0	7388	0
0	0	0	0	0	8	0
109	97	253	259	13	42	430
0	0	0	0	0	0	0
0	0	0	0	0	0	3
0	0	0	0	0	0	0
0	0	0	0	0	0	0
6130	2382	3481	7422	451	1051	4091
2	0	574	1	0	0	250
4	0	391	0	0	36	619
8	2	10	4	0	0	122
108	85	88	174	1	15	9
68	13	93	38	4	23	442
59	15	58	99	1	84	20
473	385	18	531	80	115	54
0	1	121	1	0	0	0
0	0	4	1	0	0	0
0	0	2	1	0	121	1
0	0	0	0	0	0	0
0	0	0	0	0	0	0
0	0	0	0	0	0	0
0	0	0	0	0	0	0
0	0	0	0	0	0	0
0	0	10	0	0	0	0
0	5	254	0	9	36	0
0	0	16	0	0	0	1
0	0	2	0	0	0	0
0	15	7	0	0	0	0
0	0	0	0	0	0	0
60	6	3	29	0	0	0
0	0	0	0	0	0	0
0	1	0	0	26	42	0
163	85	51	125	7	14	81
0	0	0	0	0	0	0
0	0	2	0	0	0	0
15	2	15	0	0	0	0
0	0	0	0	0	0	0
0	0	0	0	0	0	11
0	9	10	0	2	4	7
2	0	0	0	0	0	0
0	0	0	0	0	0	0
22	11	4	0	0	0	0
0	0	6	0	0	0	1
1	0	0	0	0	0	0

0	0	0	1	0	0	0
6	0	1	0	0	0	5
0	0	0	0	0	0	0
0	0	0	0	3	0	0
0	0	0	0	0	3	0
0	0	0	0	0	0	0
0	0	12	0	0	0	0
0	0	0	0	0	0	6
0	0	0	0	0	2	0
0	0	4	0	0	0	0
0	0	0	0	0	0	0
0	0	20	0	0	0	0
0	0	44	0	0	0	0
0	0	11	2	0	0	0
1	0	0	0	0	0	0
0	0	0	0	0	0	0
0	0	0	0	0	0	0
0	0	1	0	0	0	0
19	25	1	46	4	4	0
0	0	0	0	0	0	0
0	0	0	0	0	0	0
42	9	10	6	4	3	5
0	0	0	0	0	0	0
0	0	0	0	0	0	0
0	0	0	0	0	0	0
0	1	0	1	0	0	3
0	0	0	0	0	0	0
0	0	0	0	0	0	0
0	0	0	0	0	0	0
0	0	0	0	0	0	0
0	0	0	0	0	0	0
5	1	3	5	0	0	2
1	0	1	2	0	1	5
15	1	1	10	0	1	6
0	0	0	0	0	0	0
0	0	0	0	0	0	0
2	0	9	0	0	1	9
0	0	0	0	0	0	0
0	0	1	0	0	0	0
0	6	2	2	3	0	7
0	0	0	0	0	0	0
0	0	2	0	0	0	0
0	0	0	0	0	0	0
0	0	2	0	0	2	0
0	0	0	0	0	0	0
0	0	3	0	0	0	0
0	0	0	0	0	0	0
0	0	0	0	0	0	0

5	0	0	1	0	0	0
3	0	0	0	0	0	1
0	0	0	0	0	0	0
0	0	2	0	0	0	1
0	2	0	0	0	0	3
0	0	0	0	0	1	1
0	0	0	0	0	0	0
1	0	0	0	0	0	1
0	0	0	0	1	0	0
7	3	1	3	0	1	2
0	0	0	0	0	0	0
0	0	0	0	0	0	0
0	0	0	0	0	0	0
0	0	0	1	0	2	0
0	0	0	0	0	0	0
0	0	0	0	0	0	0
0	0	0	0	0	0	0
0	0	0	0	0	0	0
1	1	0	0	0	0	0
0	0	0	0	0	0	0
0	0	0	0	0	0	0
0	0	0	0	0	0	0
0	0	0	0	0	0	0
0	0	1	0	0	0	0
3	0	0	0	0	0	0
0	0	0	0	0	0	0
0	0	0	0	0	5	0
0	0	0	0	0	1	0
0	0	0	0	0	0	0
0	0	0	0	0	0	1
0	0	0	0	0	0	0
0	0	14	0	0	0	0
0	0	0	0	0	0	0
0	0	0	0	0	0	0
0	0	0	0	0	0	0
0	0	10	0	0	0	26
0	0	5	0	0	17	2
17	0	11	1	1	25	0
2	0	47	1	0	0	0
0	1	7	0	0	0	0
0	0	0	0	0	0	0
0	0	1	0	0	0	0
5	2	5	0	3	41	9
0	0	0	0	0	0	0
0	0	0	0	0	0	0
0	0	0	0	0	0	0
0	0	0	0	0	0	0
0	0	0	0	0	0	2

48	0	1	25	0	18	2
1	0	72	1	0	0	0
0	0	15	1	0	0	0
1	9	7	3	1	5	3
0	0	3	0	0	0	0
0	0	0	0	0	0	0
98	70	47	85	0	0	132
0	0	40	0	0	0	0
32	4	70	20	0	43	32
4	1	71	0	4	3	1
0	0	4	0	0	2	0
0	0	51	0	0	3	0
1	0	0	0	2	0	0
0	0	0	0	0	0	0
0	0	0	0	0	0	0
0	0	0	0	0	0	0
0	0	3	0	0	0	0
0	0	0	0	0	0	0
3	0	74	3	8	128	134
0	1	34	0	0	6	0
0	0	0	0	0	0	0
0	0	0	0	0	0	0
0	0	10	0	0	0	0
0	0	0	0	0	0	0
0	0	0	0	0	0	0
0	0	0	0	0	0	0
0	8	7	24	0	0	2
0	0	0	0	0	0	0
0	0	0	0	0	0	0
1	0	5	1	0	0	0
0	0	0	0	1	0	0
0	0	1	0	0	0	0
0	0	0	0	0	0	0
0	0	0	1	0	0	0
0	0	0	0	0	0	0
0	0	0	0	0	0	0
0	0	0	0	0	0	0
0	0	0	0	0	0	0
0	0	0	0	0	0	0
0	0	0	0	0	0	1
0	0	0	0	0	0	3
0	0	0	0	0	0	0
0	0	22	0	0	2	0
0	5	10	43	0	5	0
0	0	0	0	0	0	0
0	4	2	0	1	0	0
0	0	0	0	0	0	0
0	0	0	0	0	0	0

0	0	0	0	0	0	0
0	0	0	0	0	0	0
5	34	195	346	31	14	1
0	0	0	0	0	0	0
0	0	0	0	0	0	0
0	0	0	0	0	0	0
0	0	0	0	0	0	0
0	0	0	0	0	0	0
0	0	0	0	0	0	0
0	0	0	0	0	0	0
2	0	0	0	0	0	0
0	6	1	0	0	0	0
0	0	0	0	0	0	0
0	0	10	0	0	0	0
0	0	0	0	0	0	0
0	0	0	0	0	0	0
0	0	0	0	0	0	0
0	0	0	0	0	0	0
0	0	0	0	0	0	0
0	0	0	0	0	0	22
0	0	0	0	0	0	0
0	0	0	0	0	0	0
0	0	0	0	0	0	0
0	2	50	0	0	0	0
0	0	38	0	0	0	2
0	0	3	0	0	1	1
0	0	0	0	0	0	0
0	0	0	0	0	0	0
0	1	0	0	9	1088	0
0	2	1	0	2	0	6
0	0	1	0	0	0	0
0	0	0	0	0	0	410
7	0	0	15	0	0	0
0	0	0	0	0	0	0
0	0	0	0	0	0	0
0	0	0	0	0	0	0
1	0	0	0	0	0	0
0	0	0	0	0	0	0
0	0	0	0	0	0	0
0	0	0	0	0	0	0
177	104	43	174	12	43	39
0	0	0	0	0	0	0
0	0	1	0	0	0	0
921	367	792	1073	672	2651	582
0	6	16	59	1	11	28
383	108	534	226	387	80	20
930	20	108	458	37	58	10
45	14	95	37	7	13	10

8	0	0	30	16	0	3
38	2	1	1	83	78	12
0	3	18	0	4	31	4
5	1	0	29	11	10	9
1	0	0	0	93	17	4
0	0	5	0	0	271	0
1	0	0	0	0	3	0
9	2	5	1	0	5	1
9	0	1	0	0	0	3
2	0	1	1	8	5	0
0	1	25	16	1	5	0
16	0	4	0	1	0	1
28	4	0	1	1	1	0
0	3	1	0	0	2	0
0	0	4	0	0	12	0
6	0	1	0	1	5	7
13	7	14	12	3	5	0
2	7	64	0	0	24	2
0	0	0	0	0	0	0
0	3	11	0	0	3	1
0	1	2	0	0	0	0
5	2	0	0	1	6	2
0	0	0	0	0	0	0
0	0	0	0	0	0	0
0	0	17	0	0	1	0
0	0	0	0	0	0	1
0	0	1	0	0	2	0
2	0	0	0	0	0	0
0	0	0	0	0	2	0
0	0	3	0	0	1	0
0	0	0	0	0	0	0
0	0	0	0	0	0	0
1	0	0	0	0	0	0
0	0	0	0	0	0	0
0	0	0	0	0	0	0
0	0	0	0	2	1	1
0	0	1	0	0	0	0
0	0	0	0	0	0	0
0	0	0	0	0	0	0
0	0	0	0	0	0	0
0	0	0	0	9	3	2
1	1	0	0	0	0	0
0	0	0	0	0	0	0
0	0	0	0	0	0	0
10	0	0	0	0	0	0
0	0	0	0	0	0	0
0	1	0	0	3	3	0
0	0	0	0	0	0	0

1	0	0	0	0	0	1
0	0	0	0	0	0	0
0	0	0	0	0	0	0
0	0	0	0	0	0	2
0	0	0	0	21	9104	0
30	4	0	18	42	21	13
0	0	0	0	0	0	0
31	27	142	52	0	35	106
4	1	3	3	0	0	8
5	0	0	1	4	3	0
0	0	4	0	0	3	0
0	0	0	0	0	0	0
0	0	0	0	0	0	2
0	0	0	0	0	0	0
0	0	1	0	0	0	0
0	0	0	0	0	0	0
0	0	0	0	0	0	0
0	0	0	0	0	0	0
0	0	0	0	0	0	0
2	0	0	0	0	0	0
0	0	0	0	0	0	0
0	0	0	0	0	0	0
0	0	0	0	0	0	0
0	2	0	0	0	0	0
0	0	0	0	0	0	0
0	0	0	0	0	0	0
0	0	0	0	0	0	0
0	0	0	0	0	0	0
0	0	0	0	0	0	0
0	0	0	0	0	0	0
0	5	8	0	0	1	1
79	37	1	42	0	0	0
0	0	0	0	0	0	0
0	0	0	0	0	0	0
0	1	0	0	0	0	0
0	2	33	6	1	15	0
99	5	37	30	152	37	14
739	183	2	263	0	0	40
442	17	0	23	0	0	0
0	0	0	0	0	0	4
0	0	0	0	0	2	0
0	0	0	0	0	0	0
0	0	0	0	0	0	0
2	1	0	0	0	0	0
0	0	0	0	0	0	8
0	0	0	0	0	0	0
0	0	0	0	0	0	4
0	0	0	0	0	0	0

0	0	0	0	0	0	0
0	0	0	0	0	0	0
0	0	0	0	0	0	0
0	0	0	0	0	0	0
0	0	0	0	0	0	0
0	0	5	0	0	0	0
0	1	0	0	0	0	11
0	0	2	0	0	0	0
0	0	0	0	0	0	1
1	0	1	0	0	0	383
7	9	0	4	10	0	0
6	10	0	14	0	0	0
32	0	1	0	0	3	0
0	0	4	0	0	4	0
46	23	34	30	12	7	1
0	0	0	0	0	3	1
0	0	0	0	1	0	0
43	0	0	7	0	0	81
0	0	70	0	0	4	0
0	0	0	0	0	0	85
0	0	1	0	0	0	0
0	0	0	0	0	0	0
0	2	30	7	0	11	5
0	0	0	0	0	0	4
0	0	11	0	0	0	0
0	0	0	0	0	0	2
2	4	3	18	11	7	4
0	0	15	0	0	0	0
0	0	1	0	0	0	1
0	2	3	0	0	7	5
0	0	0	0	0	0	0
0	0	0	0	0	0	0
0	0	0	0	0	0	0
0	0	0	0	0	0	0
0	0	0	0	0	0	0
0	0	1	0	0	1	0
0	0	0	0	0	0	0
0	0	0	0	0	0	0
0	0	0	0	0	0	0
0	1	0	0	0	0	0
0	0	0	0	0	0	0
0	0	0	0	0	0	0
0	0	0	0	0	0	0
0	0	0	0	0	0	0
0	0	0	0	0	0	0
0	0	0	0	0	0	0
0	0	0	0	0	0	0
0	0	0	0	0	0	0

0	0	0	0	0	0	0
163	329	354	355	169	213	107
43	49	14	64	4	13	36
15	18	3	15	26	19	55
15	21	7	66	0	0	1
0	0	0	0	0	0	0
6	6	0	3	2	0	0
10	6	4	1	0	0	0
1	11	2	0	11	1	0
0	2	23	0	0	1	12
0	0	2	0	0	0	0
0	0	26	0	0	2	0
4	45	1	25	3	1	0
0	2	4	0	0	0	0
9	3	2	1	0	0	0
0	0	45	0	0	0	0
0	0	0	0	0	0	0
0	0	5	0	0	0	0
0	5	2	0	0	0	0
0	0	0	0	0	0	1
1	4	4	3	15	9	2
0	0	0	0	0	0	0
1	0	0	0	0	1	0
9	0	0	0	0	0	0
0	0	0	0	0	0	0
0	0	0	0	0	0	0
0	0	0	0	0	0	0
0	0	0	0	0	0	0
0	0	0	0	0	0	0
0	0	0	0	0	0	0
0	0	0	0	0	0	0
0	0	0	0	0	0	0
4	0	87	12	0	3	0
73	33	100	26	0	17	16
1084	196	133	529	36	233	87
62	2	131	81	0	10	9
12	0	1	0	1	0	44
0	0	0	6	0	0	43
42	3	116	12	15	390	5
53	21	363	49	7	138	2
5	8	15	6	19	6	0
190	35	7	75	4	0	20
0	0	0	0	9	3	0
0	1	43	0	2	9	0
0	0	0	0	0	0	0
0	0	0	0	0	2	5
0	0	12	0	0	0	0
0	0	0	0	0	1	0

0	0	0	0	0	0	0
0	0	0	0	0	0	0
0	0	0	0	0	0	0
0	0	0	0	0	0	0
0	0	0	0	0	0	0
0	0	0	0	0	0	0
0	0	0	0	0	0	0
0	0	0	0	0	0	0
0	0	0	0	0	0	0
0	0	0	0	0	0	0
0	0	0	0	0	0	0
0	0	0	0	0	0	0
0	0	0	0	0	0	0
0	0	0	0	0	0	0
1	1	1	0	0	0	0
0	0	0	0	0	0	0
0	0	0	0	0	0	0
0	0	0	0	0	0	0
0	0	0	0	0	0	0
0	0	0	0	0	0	1
0	0	0	0	0	0	0
0	0	0	0	0	0	0
0	0	0	0	0	0	0
0	0	0	0	0	0	0
0	0	0	0	0	0	0
0	0	0	0	0	0	0
0	0	0	0	0	0	0
0	0	3	0	0	0	0
0	0	0	0	0	0	0
3	6	46	0	6	19	3
0	1	10	0	5	2	5
0	0	2	0	0	0	0
0	0	21	1	0	1	0
2	0	30	1	1	4	0
0	0	1	0	0	0	0
0	0	0	0	0	0	0
0	0	0	0	0	0	0
0	0	0	0	0	0	0
0	0	0	0	0	0	0
0	0	1	0	0	0	0
0	0	0	0	0	0	0
0	0	0	0	3	15	0
0	0	0	0	0	0	0
1	0	0	0	5	3	4
0	0	0	0	0	0	0
0	0	0	0	0	0	0
0	0	0	0	0	0	0
0	0	0	0	0	0	0

1143	2940	11102	673	4254	4201	4276
5	23	189	16	17	12	41
0	0	0	0	0	0	0
0	0	0	0	0	0	0
0	0	2	0	0	0	0
0	1	8	0	3	2	0
0	0	0	0	0	0	0
0	0	3	0	0	1	0
0	0	1	0	0	0	0
0	0	0	0	0	2	0
0	0	0	0	0	0	0
0	0	0	0	2	5	0
17	29	274	3	149	43	435
1	0	212	0	0	13	0
0	0	0	0	0	0	144
0	0	0	0	0	0	0
0	0	0	0	0	0	0
0	0	0	0	0	0	0
2	0	0	0	0	0	0
115	288	172	33	221	16	2
0	0	0	0	0	0	0
0	0	0	0	0	0	0
1	0	0	0	0	0	0
0	0	0	0	0	0	0
12	57	555	41	10	65	114
0	0	0	0	0	0	9
0	0	0	0	0	0	0
641	10	6	4	96	12	3
0	2	1	116	5	4	77
193	347	208	525	81	33	124
232	6	59	137	2	9	78
0	0	67	0	0	126	137
15	52	19	189	39	7	24
3	0	0	1	17	23	0
65	48	19	259	1	0	0
5	8	2	2	3	0	1
3	12	43	9	0	1	1
0	1	5	0	0	0	0
0	0	0	0	1	0	0
0	0	0	0	0	0	0
0	0	0	0	0	0	0
0	0	0	0	0	0	0
0	0	0	0	0	0	0
56	0	0	3	7	1	283
3	10	120	20	15	4	115
0	26	53	27	2	63	0
0	0	0	0	0	0	2
0	0	0	0	0	0	110

0	0	0	0	0	0	0
3	0	0	0	0	0	1
1	0	0	0	0	0	0
4	0	0	0	1	0	0
0	0	0	0	0	0	0
0	0	0	0	0	0	0
0	0	0	0	0	0	0
0	0	0	0	38	73	0
0	0	0	0	36	3	0
2	3	310	5	8	95	1
0	0	0	0	0	0	0
3	14	15	7	2	0	0
0	0	0	0	0	3	0
0	0	0	0	0	0	0
0	0	0	0	0	0	0
3381	7570	10214	3266	1086	192	313
4	169	5192	32	5	122	1512
5	11	28	6	0	1	2578
0	0	2	0	0	0	6
0	0	0	0	0	0	1
5	8	10	0	1	0	0
0	0	0	0	0	0	0
0	1	1	0	0	0	0
0	0	0	0	0	0	2
25	10	29	2	2	0	0
5	3	5	3	0	0	2
5	4	3	1	0	0	0
0	0	0	0	0	0	0
196	268	421	65	19	0	0
66	66	174	80	48	11	6
39	43	155	20	273	21	2
4	60	778	8	71	44	0
19	33	21	51	13	13	59
0	0	0	0	0	0	0
0	0	0	0	0	0	0
0	0	0	0	0	0	0
1	0	0	0	0	0	1
0	0	0	0	0	0	0
379	436	5426	542	476	6429	374
16	36	1696	74	107	854	4
4	10	1518	13	17	103	0
0	0	18	0	0	267	0
10	30	546	12	74	277	0
0	0	0	0	0	0	0
0	0	0	0	0	0	0
0	0	63	0	2	9	0
0	0	14	1	0	1	0
0	0	2	0	0	2	0

0	0	0	0	0	0	0
0	0	0	0	0	0	0
0	1	0	0	0	0	0
0	0	0	0	0	0	0
0	0	1	0	0	2	0
0	0	0	0	0	0	0
0	0	6	0	2	2	0
0	0	6	0	2	13	0
0	0	0	0	0	0	0
0	0	1	0	1	4	0
0	0	0	0	0	2	0
191	42	143	66	0	0	0
0	0	0	0	0	0	0
0	0	0	0	0	0	0
17	57	32	100	53	36	1
362	8	3	10	0	0	0
0	0	21	0	0	0	0
0	0	0	0	0	0	1
0	3	5	1	1	1	0
2	0	0	6	0	0	64
0	0	0	0	1	0	0
0	0	0	0	0	0	0
0	0	2	0	0	0	0
0	0	0	0	0	0	0
7	0	0	0	0	0	0
1409	3532	5081	551	772	146	416
6	68	284	9	1665	213	0
3	10	15	0	0	61	0
0	0	0	0	0	0	0
0	3	11	1	1	0	0
0	5	559	33	74	1446	0
0	13	4	4	1	0	0
0	0	0	0	0	0	0
0	0	0	0	0	2	0
0	0	0	0	0	0	0
0	0	0	0	0	0	0
0	0	0	0	0	0	0
0	0	0	0	0	3	0
0	0	0	0	0	0	0
0	0	0	0	0	0	0
0	0	0	0	0	4	0
0	0	0	0	0	0	0
0	0	59	0	0	0	0
6	5	1	9	0	0	0
0	0	0	0	0	0	14
0	0	0	0	0	0	0
0	0	0	0	0	0	0
0	0	0	0	0	0	0

0	0	0	0	0	0	0
0	0	0	0	0	0	0
113	26	3522	178	21	120	1766
329	319	7	205	10	0	23
76	18	533	89	0	10	254
2	0	0	1	0	0	0
48	41	99	30	1	8	153
4	0	309	21	5	180	15
0	3	233	15	0	6	10
201	111	12	77	27	8	13
0	0	0	0	0	0	1
0	0	8	0	1	1720	0
47	7	65	35	7	46	220
3	0	0	0	0	0	64
43	1	52	14	3	6	243
3	0	46	0	0	19	0
0	0	3	0	0	0	70
0	0	3	0	0	0	26
14	6	52	46	17	510	26
0	1	42	0	0	0	87
0	0	5	0	0	0	0
0	0	0	0	0	0	0
1	0	83	0	0	3	1
0	0	10	0	0	0	4
7	0	4	19	0	10	26
38	6	69	22	5	9	44
0	0	22	0	0	12	51
0	0	0	339	0	0	0
0	0	0	0	1	6	0
0	0	11	0	0	0	0
0	0	0	0	0	0	0
0	0	56	0	0	0	0
0	0	0	0	0	0	0
0	0	0	0	0	0	0
0	0	18	0	0	0	0
0	0	1	0	0	0	0
0	0	17	0	0	20	0
10	2	0	0	0	0	1
0	0	0	0	0	0	0
0	0	4	0	0	0	0
0	0	0	0	0	0	0
0	0	0	0	0	0	0
0	0	3	0	0	2	2
0	0	0	0	0	0	0
0	0	0	0	0	0	0
0	0	0	23	0	0	0
0	0	0	0	0	0	0
0	0	0	0	0	0	0

0	0	0	0	0	0	6
0	0	6	0	0	0	0
0	0	0	0	0	0	0
0	0	3	0	0	0	3
0	0	0	0	0	0	0
0	0	0	0	0	0	0
0	0	0	0	0	0	0
0	0	0	0	0	0	0
0	0	0	0	0	0	1
0	0	0	0	0	0	0
0	0	0	0	0	0	0
0	0	0	0	0	0	0
0	0	0	0	0	0	0
0	0	0	0	0	0	1
0	0	0	0	0	0	0
0	0	0	0	0	0	0
0	0	0	0	0	0	0
0	0	0	0	0	0	0
0	0	3	0	0	0	0
0	0	0	0	0	0	0
0	0	0	0	0	0	0
0	0	0	0	0	0	0
0	0	0	0	0	0	0
0	0	0	0	0	0	0
0	0	0	0	0	0	0
0	0	0	0	0	0	0
0	0	4	0	0	0	0
0	0	0	0	0	0	0
0	0	0	0	0	0	0
0	0	0	0	0	0	0
0	0	0	0	0	0	0
0	0	0	0	0	0	0
0	0	0	0	0	0	0
0	0	0	0	0	0	0
0	0	0	0	0	0	0
0	0	0	0	0	5	0
0	0	0	0	0	0	0
0	0	0	0	0	0	0
0	0	0	0	0	0	1
0	0	0	0	0	0	0
0	0	0	0	0	0	0
0	0	0	0	0	0	0
3	0	0	0	0	0	0
0	0	0	0	0	0	0
340	49	19	4	18	21	9
31	10	22	4	3	2	209
0	0	0	0	0	0	0
0	0	0	0	0	0	0

0	0	0	0	0	0	0
0	0	0	0	0	0	0
1	1	0	1	0	0	0
94	70	111	168	10	35	3
2	0	0	0	11	0	0
1	2	0	7	0	0	2
0	0	0	0	0	0	0
0	0	0	0	0	0	5
2275	1444	1145	2460	102	703	213
2599	3398	5966	2539	532	60670	1382
9	0	60	15	0	0	11
0	10	0	4	0	0	19
403	375	133	826	132	66	110
88	94	21	135	2	5	7
0	0	175	5	1	3405	0
8	6	16	9	0	62	3
3	0	0	0	0	0	58
0	5	9	18	0	7	0
1	0	0	4	1	5	0
12	53	56	5	4	90	3
22	188	128	73	1	15	1
0	19	32	16	2	41	5
1	4	8	7	3	94	20
4	0	7	0	0	0	0
0	1	5	7	0	0	10
14	18	36	12	12	61	12
1	0	17	4	1	6	0
11	26	20	12	4	498	14
1	5	21	1	0	773	0
0	0	0	0	0	0	0
1	1	7	1	0	0	0
0	2	0	0	0	0	0
3	4	8	2	2	33	3
1	2	5	2	0	22	1
5	0	0	7	0	0	0
0	0	0	0	0	0	0
0	0	1	0	0	3	0
0	0	8	0	0	50	6
0	0	29	0	0	0	0
0	1	0	0	0	18	0
0	0	0	0	0	0	0
0	2	0	0	0	0	2
0	0	0	0	0	0	5
2	3	0	6	0	0	0
0	0	0	0	1	67	0
0	0	0	0	0	0	0
0	1	0	0	0	7	0
0	0	0	0	0	0	0

0	0	0	0	0	0	0
8	11	42	2	1	149	9
0	0	0	0	0	0	0
0	4	0	0	0	0	0
0	0	0	0	0	0	0
0	0	4	0	0	19	1
0	0	0	0	0	0	0
10	23	9	9	2	281	14
0	1	0	0	0	0	0
0	0	0	1	0	0	0
0	0	1	0	0	3	0
1	2	2	1	0	21	3
0	1	0	0	0	0	0
1	1	1	0	0	3	0
0	0	0	0	0	0	0
5	3	4	4	1	40	1
0	0	0	0	0	0	0
0	0	0	0	0	0	0
0	2	1	0	0	32	2
0	0	0	0	0	3	0
0	0	0	0	0	0	0
0	0	0	0	0	0	0
0	1	3	0	0	0	1
0	1	11	0	0	38	0
1	0	0	1	0	0	0
0	0	0	0	0	0	0
0	0	0	0	0	0	0
0	0	0	0	0	0	2
0	0	0	0	0	0	0
1	0	1	0	0	0	0
0	0	0	0	0	7	0
0	0	2	0	0	3	0
0	0	0	0	0	1	0
0	0	0	0	0	0	0
0	0	0	0	0	0	0
0	0	0	0	0	0	0
0	0	0	0	0	0	0
0	0	0	0	0	0	0
0	0	0	0	0	0	0
0	0	0	0	0	0	0
0	0	0	0	0	0	0
0	0	1	0	0	9	0
0	2	1	0	0	14	0
0	0	0	0	0	5	0
0	0	0	0	0	7	0
0	0	0	0	0	8	0
0	0	0	0	0	0	0
0	0	0	0	0	0	0

0	0	0	0	0	0	0
280	792	444	502	172	350	382
54	97	606	49	24	7490	72
88	150	15	145	21	24	108
3	1	0	0	33	2	22
10	31	5	45	20	40	9
39	64	16	29	3	23	14
3	2	0	5	0	0	0
3	0	7	0	1	3	1
31	40	62	30	5	156	4
1	1	3	0	0	0	0
0	1	50	0	0	21	1
1	4	1	0	0	2	0
5	32	1	7	11	9	0
0	2	1	0	0	0	0
0	0	0	0	0	0	0
14	7	10	12	5	10	0
0	1	12	1	0	78	2
0	1	1	0	0	9	0
1	0	0	1	0	1	0
0	0	5	0	0	59	0
1	2	1	2	0	0	0
1	5	6	0	0	0	2
0	0	1	0	0	3	0
0	0	0	0	0	0	0
1	0	0	0	0	0	0
0	0	0	0	0	0	0
0	0	0	0	0	0	0
0	0	1	0	0	0	0
0	0	2	0	0	1	1
0	0	0	0	0	0	0
0	0	0	0	0	0	0
0	0	0	0	0	0	0
0	0	0	0	0	0	0
0	0	0	0	0	1	0
0	0	0	0	0	0	0
3156	913	29	977	392	90	33
0	9	9	56	4	23	2
0	0	0	0	0	0	0
0	0	0	0	0	0	11
0	0	0	0	0	0	0
1	0	0	0	0	0	0
0	1	0	5	0	0	2
0	0	0	0	0	0	0
0	0	0	0	0	0	0
0	0	0	0	0	0	0
1	0	0	1	0	0	0
0	2	0	3	0	0	0

0	0	0	0	1	0	0
0	0	0	0	0	0	0
0	5	1	1	0	0	0
0	0	0	0	0	0	0
0	0	0	0	0	0	0
0	0	0	0	0	0	0
0	0	0	0	0	0	0
0	0	1	0	0	0	0
0	0	0	0	0	0	0
0	0	0	0	0	0	0
4	0	0	0	0	0	0
0	0	0	0	0	0	0
0	0	0	0	0	0	0
0	1	0	0	0	0	0
0	0	0	0	0	0	0
0	0	0	0	0	0	0
0	0	0	0	0	0	0
0	0	6	0	0	3	8
0	0	0	0	0	0	1
0	0	0	0	0	0	0
0	0	0	0	0	0	0
0	1	58	0	0	0	0
0	0	9	0	0	0	0
0	0	0	0	0	0	0
0	0	0	0	0	0	0
0	0	0	0	0	0	0
0	0	0	0	0	0	0
0	0	0	0	0	0	0
0	0	0	27	0	0	0
4	39	17	140	1	4	0
0	0	0	30	0	3	0
0	0	0	0	0	0	0
0	0	0	0	0	0	0
27	43	7	92	18	10	110
0	0	0	0	0	0	0
142	111	18	342	2	2	0
0	21	8	18	3	5	2
0	0	0	0	0	0	0
25	1	1	0	0	0	0
1	0	0	0	0	0	0
0	0	0	0	0	0	0
8	4	2	4	0	0	0
257	107	5	256	1	0	16
0	1	0	0	0	0	0
1	4	1	23	0	14	0
0	0	0	0	0	3	0
0	0	0	0	0	0	0
0	5	1	9	6	10	2

0	0	0	0	0	0	0
85	39	6	189	299	27	117
119	326	59	285	54	86	6
6	0	0	0	0	0	0
0	0	7	0	0	0	0
0	0	0	0	0	0	0
0	0	0	0	0	0	0
0	0	0	0	0	0	0
0	0	0	0	0	0	0
112	38	4	48	40	17	55
78	23	23	107	5	188	66
0	0	470	0	1	7	0
0	0	0	0	0	1	0
0	0	70	0	0	0	0
0	0	0	0	0	0	0
0	0	0	0	0	0	0
0	0	0	0	0	0	0
0	0	0	0	0	6	0
0	0	0	0	0	0	0
0	0	0	0	0	0	0
0	0	6	0	0	0	0
0	0	0	0	0	0	0
0	0	0	0	0	0	0
0	0	0	0	0	0	0
0	0	0	0	0	0	0
0	0	4	0	0	0	0
0	0	0	0	0	0	0
0	0	0	0	0	0	0
0	0	0	0	0	0	0
0	0	0	0	0	0	0
0	0	0	0	0	0	0
0	8	0	0	0	5	0
0	0	0	0	0	0	0
0	0	0	0	0	0	0
0	0	4	0	0	0	0
0	2	0	0	0	0	3
0	1	1	0	0	1	2
0	0	0	0	0	0	0
0	0	0	0	0	0	0
0	0	0	0	0	0	0
2	0	28	2	1	6	0
2	0	1	0	0	1	0
0	0	0	0	0	0	0
0	0	0	0	0	0	0
1	0	0	0	0	0	6
0	0	2	0	0	2	0
0	0	0	0	0	0	0
2	0	0	0	0	0	0

1	0	7	0	2	4	1
0	0	0	0	0	0	1
0	0	0	0	0	0	0
0	0	76	0	2	79	0
0	0	0	0	0	0	0
0	0	0	0	0	0	0
2	0	421	1	1	96	7
0	0	17	0	0	0	0
0	0	44	0	0	0	0
0	0	0	0	0	0	0
0	0	0	0	0	0	0
0	0	0	0	0	0	0
0	0	37	0	0	2	1
0	0	0	0	0	0	0
1	0	1433	10	2	47	2
0	0	3	0	0	0	0
0	0	0	0	0	0	0
0	0	0	0	0	0	0
0	0	4	0	0	0	0
0	0	0	0	0	0	0
0	0	0	0	0	0	0
0	0	0	0	0	0	0
0	0	1	0	0	0	0
0	0	0	0	0	0	0
0	0	3	0	0	0	0
0	0	0	0	0	0	0
0	0	0	0	0	0	0

32.SUR	32.DCM	33.SUR	33.DCM	34.SUR	34.DCM	36.SUR
0	0	0	0	0	0	0
0	0	0	0	0	10	0
0	0	0	2	0	9	0
1	3	0	1	1	14	0
0	0	6	0	0	0	0
0	0	0	0	0	1	0
0	0	0	0	0	0	0
0	10	7	1	9	167	0
0	0	0	7	0	1	0
0	0	1	0	0	0	0
0	0	0	0	0	0	0
0	5	0	0	0	0	0
0	0	0	0	1	3	0
0	1	0	0	3	33	0
1	5	0	0	0	1	0
3	8	0	0	0	0	0
1	27	6	7	4	54	0
273	112	775	181	60	75	28
0	0	0	8	3948	182	75
0	1	0	1	0	16	6
0	0	0	0	0	0	0
0	2	0	0	0	0	0
0	0	0	0	0	0	0
0	0	0	0	0	0	0
0	0	0	0	0	1	0
0	0	0	0	0	0	0
0	0	0	0	0	0	0
0	0	0	0	0	0	0
0	0	0	0	0	0	0
1	0	0	1	0	1	0
0	0	0	0	0	0	1
0	0	0	0	0	0	0
1	15	0	12	2	11	0
1	3	0	0	0	12	0
0	2	0	1	0	4	0
0	0	0	0	0	0	0
0	838	5	7	23	4	16
647	46	1736	316	50	83	583
11	8	0	3	1	1	3
0	0	0	0	0	0	0
1	23	0	0	0	16	0
0	0	0	27	0	4	0
28	4	30	80	4	9	1
2	12	85	104	46	664	8
1	14	0	27	1	21	0
0	0	0	1	1	73	0
0	1	39	19	5	11	8

1	8	0	11	0	0	0
0	2	0	1	0	37	0
2	4	0	0	1	27	0
7	38	0	9	0	26	0
0	1	0	2	0	0	0
0	1	0	0	3	56	0
0	5	0	1	1	5	0
25	2	6	0	0	4	1
0	0	0	0	0	0	13
0	0	0	0	0	6	0
0	0	0	0	0	1	5
0	2	0	1	0	15	0
1	0	6	1	0	0	8
1	0	0	2	0	1	0
1	3	0	0	0	13	0
0	0	0	0	0	0	0
0	0	0	0	3	2	0
0	1	0	0	0	8	0
0	0	0	0	0	0	0
0	0	0	3	0	0	0
0	7	0	0	0	2	0
0	0	0	0	0	0	0
0	0	0	0	0	0	0
0	0	0	0	0	0	0
0	0	0	0	0	0	0
0	0	0	0	0	0	0
1	0	0	0	0	0	0
0	0	0	1	0	0	0
0	0	0	0	0	0	0
0	2	0	5	0	5	0
0	1	0	0	1	5	0
0	0	0	2	1	0	0
0	0	0	0	0	0	0
0	0	0	0	0	0	0
0	0	0	0	0	0	0
0	0	0	0	0	0	0
0	0	2	0	1	0	0
0	0	0	0	0	0	0
0	0	0	0	0	0	0
152	308	867	392	41	219	205
21	34	253	54	6	1	9
27	58	4529	182	144	8	572
0	9	0	0	2	44	0
0	0	8	1	0	0	0
0	0	0	0	0	2	0
0	0	0	0	0	0	0
0	0	0	0	0	10	0
0	0	2	0	0	0	0

0	3	0	0	0	3	0
0	0	0	0	0	0	0
0	0	0	0	0	0	0
0	0	38	3	2	0	6
0	0	0	0	0	0	0
0	1	4	0	11	6	0
0	0	0	0	0	0	0
0	0	0	0	0	0	0
0	0	0	0	0	0	0
0	0	0	0	0	0	0
0	0	0	0	0	0	0
0	0	0	0	0	0	0
0	0	0	0	0	0	0
0	0	0	0	0	0	0
0	0	0	0	0	0	0
0	0	0	0	0	0	1
2	6	0	17	0	2	0
0	1	0	0	0	0	0
0	0	0	0	0	0	0
5	2	0	7	0	0	0
0	0	0	0	0	0	0
0	0	0	0	0	4	0
1	9	0	0	0	4	0
0	3	0	13	0	4	0
0	0	0	0	0	0	0
1	0	0	1	2	4	13
3	0	2	0	0	0	0
1	4	17	0	0	6	11
0	0	1	2	0	0	0
0	0	0	0	0	0	0
0	0	0	0	0	0	0
0	0	0	0	0	0	0
0	0	0	0	0	0	0
0	1	0	0	0	4	0
1	22	0	1	1	17	0
2	15	0	6	2	24	0
0	5	45	0	10	10	0
0	2	0	0	0	4	0
0	0	0	0	0	0	0
3	17	0	0	0	4	0
0	0	0	0	0	0	0
0	0	0	0	0	1	0
9	0	0	0	0	1	0
0	0	0	0	1	0	0
0	0	0	5	0	0	0
0	0	0	0	0	0	0
0	1	0	0	0	2	0
0	0	0	0	0	0	0

3	2	0	0	1	7	0
0	0	0	0	0	0	0
66	6	41	26	2	1	38
0	0	0	1	4	0	0
0	0	2	0	1	1	6
0	0	0	0	0	0	0
0	0	0	0	0	0	0
0	0	0	0	0	0	0
0	0	0	0	0	0	0
0	0	57	0	0	0	0
0	4	4	2	0	1	0
0	1	0	0	0	3	0
13	67	18	104	89	583	0
0	0	0	0	0	0	0
1	1	0	36	0	18	0
3	20	1	10	1	60	0
0	3	0	23	0	0	0
4	4	0	9	0	8	0
1	0	32	0	0	0	0
0	0	4	0	0	0	0
0	0	1	9	1	2	3
0	0	0	0	0	0	0
0	0	0	0	0	0	0
0	0	0	0	0	0	0
0	0	0	0	0	1	0
0	0	0	0	0	1	0
0	0	0	0	0	0	0
0	0	0	0	0	0	0
0	0	0	0	0	0	0
0	0	2	0	0	0	0
0	0	2	2	0	0	0
0	0	1	0	3	0	0
0	0	22	0	1	1	0
0	0	0	0	0	0	0
0	0	0	2	0	2	0
0	0	0	0	0	0	0
0	0	0	0	0	0	0
0	0	0	0	0	0	7
0	0	0	0	0	0	0
0	0	0	0	0	0	0
0	0	0	0	0	0	0
0	0	0	0	0	0	0
0	2	52	69	20	23	90
0	43	0	0	0	6	5
0	2	0	0	4	0	2
0	0	0	0	0	0	0
1	0	0	0	2	0	0
0	1	0	0	0	0	0

0	0	0	0	0	0	0
0	1	3	0	2	28	0
0	0	0	0	0	0	0
0	0	0	0	0	0	0
0	0	0	0	0	0	0
0	3	0	0	0	0	0
0	0	0	0	0	0	0
0	0	0	0	0	0	5
0	0	4	8	0	0	0
0	0	0	0	0	0	0
0	0	0	0	0	0	0
0	0	0	0	0	0	0
0	0	1	0	0	0	0
0	0	1	2	0	0	0
0	0	0	0	0	0	0
0	0	0	0	0	0	0
0	0	0	0	0	0	0
0	0	0	0	0	0	1
0	0	0	0	0	1	0
0	31	0	6	0	0	0
1	0	0	0	0	0	0
3	5	3	7	0	0	0
0	0	0	0	0	0	0
9	582	244	530	36	84	55
0	4	2	2	6	1	31
0	0	0	0	0	0	0
0	2	0	0	15	63	0
0	0	0	3	0	0	0
0	0	0	0	0	0	0
0	2	1	2	0	0	0
4	0	4	0	0	1	0
0	0	0	0	0	0	0
0	0	0	0	0	0	0
0	0	0	0	0	0	0
0	0	0	0	0	0	0
0	0	0	3	0	0	0
5	13	66	490	28	24	29
0	2	2	1	3	1	1
1	1	19	0	0	2	4
1	1	2	0	7	2	25
0	0	0	0	0	0	0
0	0	0	0	0	0	0
0	0	0	0	0	0	0
0	0	0	0	0	0	0
0	0	0	0	0	0	0
0	1	0	0	1	0	0
0	0	0	0	0	0	0
0	0	0	0	0	0	0

0	2	0	0	0	6	0
0	0	0	0	0	0	0
0	0	3	3	0	0	0
0	1	0	0	0	0	0
0	0	0	0	0	0	0
0	0	0	0	0	0	0
0	0	0	0	0	0	0
0	0	0	0	0	0	0
0	0	0	0	0	0	0
0	0	0	0	0	0	0
0	0	0	0	0	0	0
0	0	0	0	0	0	0
0	0	0	0	0	0	0
0	0	0	0	0	0	0
0	0	0	0	1	0	0
0	0	0	0	0	0	0
0	0	0	0	0	0	0
0	0	1	1	0	0	0
0	0	0	0	0	0	0
20	0	0	0	0	0	0
0	0	0	0	0	0	20
0	1	0	0	2092	55	0
0	0	0	0	0	0	0
59	379	199	142	23	314	3
1	0	1	0	8	5	0
2	0	16	56	3	2	0
1	0	6	2	0	0	0
0	0	0	0	0	0	0
0	0	0	1	0	3	0
0	0	0	1	0	0	0
0	0	0	0	0	0	0
0	0	0	0	0	0	0
5	5	1	18	0	24	0
0	0	0	6	0	1	0
0	0	0	0	0	0	0
0	0	0	0	0	0	0
0	0	0	0	0	0	0
1	2	0	0	2	17	0
0	1	0	12	0	6	0
2	1	1	0	0	12	0
0	0	0	0	0	0	0
0	0	0	0	0	0	0
0	0	0	0	0	0	0
0	0	0	0	0	3	0
0	0	0	0	0	0	0
0	0	0	0	0	0	0
1	7	0	5	0	2	0
0	0	0	0	0	0	0

1	6	0	1	0	2	0
0	2	0	1	0	0	0
0	0	0	0	0	0	0
19	30	2	72	8	0	32
0	0	0	0	0	0	7
0	0	0	0	0	0	0
34	147	283	135	42	41	77
0	0	0	0	0	0	0
0	0	6	1	5	3	0
0	0	0	0	0	0	0
0	0	0	0	0	0	0
3533	1818	10673	2825	598	2804	2284
110	13	732	113	62	4	103
141	149	1604	185	106	297	2
119	99	354	36	25	192	10
26	37	26	57	14	68	54
49	9	960	112	20	35	18
5	2	39	22	2	5	0
20	35	165	18	15	65	0
11	44	8	1	4	116	3
3	25	0	26	2	62	0
0	10	5	74	8	32	22
0	0	0	0	1	0	0
2	14	0	28	0	41	0
0	0	0	40	1	0	0
1	0	0	1	0	0	0
0	0	0	1	0	0	0
0	0	0	56	0	0	0
0	0	0	0	0	0	0
3	25	4	35	0	20	0
0	0	0	0	0	7	0
0	0	0	0	0	0	0
0	0	0	0	0	0	4
0	0	0	0	0	1	0
0	0	0	0	0	0	0
3	0	0	0	0	0	0
107	22	110	97	6	27	114
0	18	0	0	0	0	0
0	4	0	0	0	47	0
0	0	0	6	0	0	0
1	5	0	0	0	8	0
39	2	44	46	1	2	0
61	5	39	39	4	14	2
0	1	0	0	0	41	0
0	0	0	0	0	0	0
0	0	2	0	4	9	0
2	28	0	2	0	27	0
0	0	0	0	0	0	0

0	0	0	0	3	0	0
5	3	9	2	0	20	0
0	0	0	0	0	0	0
9	0	2	0	0	0	0
0	0	0	12	0	0	0
0	0	0	0	0	0	0
0	0	0	0	0	0	0
2	4	4	11	1	26	0
0	0	0	0	2	20	0
0	0	0	0	0	0	0
0	0	0	0	0	0	0
0	0	0	0	0	0	0
0	0	0	0	0	0	0
0	0	0	0	1	0	0
0	4	0	0	0	3	0
0	0	0	0	0	0	0
0	0	0	2	0	0	0
0	2	0	0	0	13	20
29	0	1	13	0	0	0
0	0	0	0	1	2	0
0	0	0	2	0	1	0
4	4	21	5	4	3	6
0	0	0	0	0	0	0
0	0	0	0	0	0	0
0	0	0	0	0	4	0
0	1	2	1	0	0	1
0	0	0	0	0	0	0
0	0	0	0	0	0	0
0	0	0	1	0	3	0
0	0	0	0	0	0	0
0	1	0	7	0	1	3
8	2	4	2	0	0	5
1	6	19	3	3	10	1
1	0	3	2	0	0	6
0	1	0	1	0	0	0
3	1	0	5	0	4	0
1	1	26	2	2	4	0
0	0	0	0	0	0	0
0	0	0	2	0	1	0
1	0	10	2	1	2	0
0	0	7	0	0	3	0
0	0	0	0	0	0	0
0	0	0	0	0	0	0
0	0	1	0	0	0	0
0	0	0	0	0	4	0
0	0	0	0	0	0	0
0	0	0	0	0	0	0
1	3	0	0	1	1	0

0	0	0	1	0	1	0
2	0	1	0	0	0	0
0	0	0	0	0	0	0
1	0	2	0	0	0	0
0	2	2	0	0	1	1
0	0	0	0	0	0	0
0	0	0	0	0	0	0
2	0	1	1	0	1	0
0	0	1	0	0	0	0
2	0	0	4	1	0	3
0	0	0	0	0	0	0
0	0	1	0	0	0	0
0	0	0	0	0	5	0
0	1	0	0	0	0	0
0	0	0	0	0	0	0
0	0	0	0	0	0	0
0	0	1	0	0	0	0
0	0	0	0	0	0	0
0	0	0	0	0	0	0
0	1	0	0	0	0	0
0	0	0	0	0	0	0
0	0	0	0	0	0	0
0	0	0	0	0	0	0
0	0	0	0	0	0	0
0	0	0	0	0	0	0
0	0	0	0	0	0	0
0	0	0	0	0	0	0
0	1	0	0	0	0	0
0	0	0	0	0	0	0
0	43	0	5	16	73	0
0	0	0	36	0	44	0
0	1	0	9	0	2	0
0	0	0	0	0	0	0
0	0	0	0	0	0	0
0	0	0	0	0	0	0
4	18	67	109	11	46	0
3	37	8	61	0	7	0
0	18	0	17	0	85	0
5	33	0	59	1	53	0
0	0	0	7	0	8	0
0	0	0	0	0	0	0
0	4	0	1	0	0	0
17	29	16	2	0	0	15
0	0	0	0	1	0	0
1	9	0	0	0	0	5
0	0	1	0	0	0	0
0	0	0	0	0	0	0
0	0	2	0	2	0	0

7	9	3	13	1	11	0
1	3	0	4	0	10	0
1	9	0	5	1	5	0
4	10	8	21	1	5	0
0	1	0	1	0	0	0
0	0	0	0	0	0	0
351	53	273	9	79	236	54
0	11	0	5	3	42	0
27	94	67	124	7	132	0
0	12	1	51	1	22	0
0	34	1	13	1	103	0
0	0	0	0	0	0	0
2	9	0	0	0	9	0
0	0	0	0	0	0	0
0	1	0	0	0	2	0
0	0	0	0	0	0	0
0	0	0	0	0	0	0
0	0	0	0	0	0	0
49	352	351	342	58	477	0
0	0	0	1	0	0	0
0	0	0	0	0	0	0
0	1	0	1	0	4	0
0	0	0	4	0	1	0
0	0	0	0	0	0	0
0	0	0	0	0	0	0
0	0	0	0	0	0	0
3	0	0	0	0	0	5
0	0	0	0	0	0	0
0	0	0	0	0	0	0
0	0	0	0	0	0	0
0	0	0	0	0	0	0
0	0	0	0	0	0	0
0	0	0	0	0	0	9
0	1	0	0	0	4	0
0	0	0	0	0	0	0
0	0	0	0	0	0	0
0	0	0	4	0	14	0
0	0	0	0	0	0	0
0	0	0	0	0	0	0
0	0	4	1	0	0	0
0	0	0	1	1	3	0
0	0	0	0	0	0	0
2	4	0	6	0	18	0
0	3	0	2	0	0	0
0	0	0	0	0	0	0
0	0	0	0	0	0	0
0	0	0	0	0	0	0
0	0	0	0	0	0	0

0	0	0	0	0	0	0
0	0	0	0	0	0	0
5	23	4	4	7	1	1
1	0	0	0	0	0	0
0	0	0	0	0	0	0
0	0	0	0	0	0	0
0	0	0	0	0	0	0
0	0	0	0	0	0	0
0	0	0	0	0	0	0
0	0	0	0	0	0	0
0	15	0	1	0	11	0
0	0	0	0	0	98	0
0	0	0	0	0	42	5
0	0	0	0	0	0	0
0	0	0	0	0	0	0
0	0	0	0	0	0	0
0	0	0	0	0	0	0
0	0	0	0	0	0	0
0	0	0	2	0	0	0
1	3	38	0	2	29	0
0	0	0	0	0	0	0
0	17	0	0	0	0	0
0	0	0	0	0	0	0
0	0	0	0	0	0	0
3	29	4	1	1	4	8
0	1	0	0	0	0	0
0	1	0	0	0	0	0
0	0	0	0	0	0	0
0	0	0	0	0	0	0
0	1	17	1	1	0	0
0	0	0	0	0	0	0
40	17	579	10	12	0	109
0	0	0	0	0	0	0
0	0	0	0	0	0	0
0	0	0	0	0	0	0
0	0	0	0	0	0	0
0	0	0	0	0	0	0
0	0	0	0	0	0	0
0	0	0	0	0	0	0
0	0	0	0	0	0	0
30	27	71	34	3	9	14
0	0	0	0	0	0	0
0	0	0	0	0	0	0
341	50	1332	1184	97	826	239
1	3	54	43	2	198	3
107	482	49	9	5	115	329
37	181	27	104	27	310	15
5	7	31	38	12	60	19

0	0	16	8	0	2	489
5	0	17	23	1	22	97
4	1	3	1	0	3	7
0	1	10	0	0	2	0
0	2	3	2	0	0	0
0	1	3	6	1	1	6
0	0	0	0	0	0	0
6	1	2	0	0	3	3
0	2	7	53	0	0	0
0	2	0	0	0	0	0
6	9	4	3	0	4	0
0	0	5	2	1	8	16
0	0	0	0	0	0	26
1	0	1	4	0	5	0
1	1	0	1	0	0	0
3	0	11	0	1	1	0
0	0	0	0	0	4	3
8	5	4	25	0	19	27
0	0	0	0	1	0	6
0	0	0	0	0	0	3
1	0	1	0	0	0	0
1	0	1	0	0	0	0
0	0	0	0	0	0	0
0	0	0	0	0	0	0
0	0	0	1	0	0	0
1	4	0	0	0	4	4
1	2	0	0	1	0	0
13	3	1	0	0	0	0
0	1	0	3	0	0	0
1	8	0	0	0	0	8
0	0	0	4	1	0	0
1	5	0	0	2	19	0
0	0	0	0	0	0	0
0	0	2	3	0	0	0
0	1	0	0	0	3	0
0	1	7	1	0	0	0
0	0	0	0	0	0	0
0	0	0	0	0	0	0
0	0	1	0	0	0	3
0	0	0	0	0	0	0
0	0	1	0	0	2	0
0	0	0	0	0	0	0
0	0	0	0	0	0	0
0	0	0	0	0	0	0
0	0	0	0	0	0	0
0	0	0	0	0	0	0
0	0	0	0	0	0	0
0	0	0	0	0	0	0

2	0	0	0	0	0	0
0	0	0	0	0	0	0
0	0	0	0	0	0	0
0	0	0	0	0	0	0
0	0	9	2	0	3	2
7	6	34	33	0	0	17
0	0	0	0	0	0	0
101	98	322	335	44	328	2
1	1	16	0	0	3	1
0	0	0	0	0	0	5
0	0	0	0	0	0	0
0	0	0	0	0	0	0
0	0	0	0	2	0	8
0	0	0	0	0	0	0
0	0	0	0	0	0	1
0	0	0	0	0	0	0
0	0	0	0	0	0	0
0	1	0	0	0	0	0
0	0	0	0	0	0	0
0	8	0	0	0	0	0
0	0	0	0	0	0	0
0	0	0	0	0	0	0
0	0	0	0	0	0	0
0	0	0	0	0	0	0
0	0	0	0	0	0	0
0	0	0	0	0	0	0
0	0	0	0	0	0	0
0	0	0	0	0	0	0
0	0	0	0	0	0	0
0	0	0	0	0	0	0
3	1	5	1	2	1	3
0	0	0	0	0	0	15
0	0	0	0	0	0	0
0	0	0	0	0	0	0
0	0	0	0	0	0	0
0	9	0	5	0	0	0
751	425	30	6	8	11	25
0	0	125	0	0	0	0
0	1	0	0	1	0	4
3	0	4	0	0	0	8
0	1	0	0	0	0	0
0	0	0	0	0	0	0
0	0	0	0	0	0	0
0	0	0	0	0	0	0
0	0	31	0	1	0	1
0	0	0	0	0	0	0
0	0	5	0	81	44	0
0	0	0	0	0	5	0

0	0	0	0	0	0	0
0	0	0	0	0	0	16
0	0	0	0	0	0	0
0	0	0	0	0	0	0
0	0	0	0	0	0	0
0	0	0	0	0	0	0
0	1	26	1	4	1	0
0	0	0	1	0	0	0
0	0	0	0	0	3	0
2	1	950	18	2	0	0
0	0	1	0	0	0	0
0	0	0	0	0	0	0
0	0	0	0	0	0	0
0	0	0	1	0	0	0
4	1	1	55	0	0	28
0	0	2	1	0	2	18
0	0	0	0	0	0	0
0	0	191	2	2	0	23
6	9	0	8	7	4	0
7	0	254	4	5	4	0
0	0	0	1	0	1	1
0	2	8	5	0	6	12
7	2	17	4	0	2	0
0	0	6	9	0	1	9
0	0	2	2	5	0	0
0	1	9	3	6	32	11
1	0	18	11	1	2	9
0	0	0	3	1	1	0
0	0	3	0	0	0	0
0	0	18	1	0	0	0
0	0	0	0	0	0	0
0	0	0	0	0	0	0
0	0	0	0	0	0	0
0	0	0	0	0	0	0
0	0	0	0	0	0	0
0	1	0	0	0	0	0
0	0	0	0	0	0	0
0	0	0	0	0	0	0
0	0	0	0	0	0	0
0	0	0	1	0	0	0
0	0	0	0	0	0	0
0	0	0	0	0	0	0
0	0	0	0	0	0	0
0	0	0	0	0	0	0
0	0	0	0	0	0	0
0	0	0	0	0	0	0
0	0	0	0	0	0	0
0	0	0	0	0	0	0

0	0	0	0	0	0	0
36	27	341	26	65	33	39
6	2	94	21	20	6	115
17	4	121	5	5	4	9
0	0	3	0	0	0	26
0	0	0	0	1	1	0
6	2	1	1	1	4	7
0	0	0	0	0	0	2
0	0	0	0	0	0	0
36	4	43	13	5	2	0
0	7	0	1	0	3	0
0	0	0	3	0	0	0
0	0	0	0	0	0	0
1	0	2	0	0	0	13
3	2	0	0	2	0	6
0	0	0	0	0	0	0
0	0	0	0	0	4	0
0	0	0	0	0	0	0
0	0	0	0	0	0	0
1	0	6	0	2	1	0
1	0	0	1	0	0	0
0	0	0	2	0	0	0
0	0	0	1	0	0	0
1	0	1	1	0	0	0
0	0	0	0	1	3	0
0	0	0	0	0	0	0
0	0	0	0	0	0	0
0	0	0	0	0	0	0
0	1	0	0	0	0	0
0	0	0	0	0	0	0
0	0	2	2	0	0	2
0	0	0	0	0	0	0
0	0	0	1	8	0	6
7	6	66	11	13	1	19
4	5	71	2	7	5	0
0	0	30	13	8	7	13
0	3	99	0	29	0	73
0	0	117	0	0	0	28
36	37	29	9	6	12	6
16	19	5	8	1	18	18
0	0	0	1	0	0	0
2	2	64	2	9	0	11
0	0	0	0	0	0	0
5	1	0	6	2	1	0
0	0	0	0	2	0	26
0	0	22	2	0	0	48
0	0	0	0	0	0	27
0	0	0	1	0	1	0

0	0	0	0	0	0	0
0	0	0	0	0	0	0
0	0	0	0	0	0	0
0	0	0	0	0	0	0
0	0	0	0	0	0	0
0	0	0	0	0	0	0
0	0	0	0	0	0	0
0	0	0	0	0	0	0
0	0	0	0	0	0	0
0	0	0	0	0	0	0
0	0	0	0	0	0	0
1	0	0	1	0	0	0
0	0	0	0	0	0	0
0	0	0	0	0	0	0
0	0	0	0	0	0	0
0	0	0	0	0	0	0
0	0	0	0	0	0	0
0	0	0	0	0	0	0
0	5	0	0	1	3	0
0	1	0	0	1	0	0
0	0	0	0	0	0	0
0	0	0	0	0	0	0
0	1	3	0	1	1	0
0	0	0	14	0	0	0
0	0	0	0	4	25	0
0	0	0	0	0	0	0
0	0	0	0	0	0	0
0	1	0	0	0	0	0
0	0	0	0	0	0	0
2	53	2	0	1	5	0
0	0	4	0	1	7	0
2	1	0	0	0	0	0
0	8	0	0	0	2	0
0	7	0	0	0	2	0
2	0	1	0	0	2	0
0	0	0	0	0	1	0
0	0	0	0	0	0	0
0	0	0	0	0	0	0
0	0	0	0	0	0	0
0	0	0	0	0	1	0
0	0	0	0	0	0	0
0	47	1	0	3	20	0
0	0	0	0	0	0	0
22	3	2	0	0	0	0
0	0	0	0	0	0	0
0	0	0	0	0	0	0
0	0	0	0	0	0	0
0	0	0	0	0	0	0

5713	9305	3854	76	602	2224	23
52	110	65	9	20	57	7
0	0	0	0	0	0	0
25	18	0	0	0	0	0
1	2	0	0	0	1	0
0	0	0	0	0	2	0
0	0	0	0	0	0	0
0	1	0	0	0	0	0
0	0	0	0	0	0	0
0	0	0	0	0	0	0
0	0	0	0	0	0	0
0	0	0	0	0	0	0
464	121	394	2	33	62	0
0	419	0	0	0	4	1
36	16	142	56	28	22	0
0	0	0	0	0	0	0
0	3	0	0	0	0	0
0	0	0	0	0	0	0
0	0	0	0	0	0	0
24	159	2	1	4	0	0
9	8	0	0	6	13	0
0	0	0	0	0	0	0
0	0	0	0	0	0	0
1	2	0	0	2	0	0
141	162	136	26	52	66	157
0	0	27	0	0	0	0
0	0	0	0	0	0	0
1	4	2	1	0	1	0
18	1	184	11	9	3	0
361	33	255	29	20	5	48
16	8	185	2	12	2	147
3	54	140	35	360	273	0
2	0	56	2	7	6	0
256	46	1	1	2	0	0
0	0	2	3	2	0	0
1	1	3	0	1	0	0
9	9	0	0	2	0	28
0	0	0	0	0	0	0
0	0	0	0	0	0	0
0	0	0	0	0	0	0
0	0	0	0	0	0	0
0	0	0	0	0	0	0
0	0	0	0	0	0	0
1503	89	280	67	56	67	0
222	301	120	4	152	240	293
0	0	2	1	0	0	0
11	1	12	2	0	0	0
9	2	283	6	3	1	1

0	0	0	0	0	0	0
1	0	0	0	0	0	0
1	0	0	0	1	0	0
0	0	0	0	0	0	0
0	0	0	0	0	0	0
0	0	0	0	0	0	0
0	0	0	0	0	0	0
0	0	0	0	0	0	0
1	0	0	0	0	0	0
1	1026	0	5	0	68	0
0	3	0	0	0	4	0
6	3	0	0	0	1	0
0	24	0	0	0	0	0
0	0	0	0	0	0	0
0	0	0	0	0	0	0
7412	1421	392	70	215	289	0
3903	1813	1511	102	103	236	0
20	2	2493	1	4	6	0
5	0	10	1	2	0	0
0	0	0	0	0	0	0
0	0	1	0	0	0	0
0	0	0	0	0	0	0
0	0	0	0	0	0	0
0	0	3	0	0	1	0
0	0	0	0	0	0	0
0	0	1	1	0	0	0
0	0	1	0	0	0	0
0	0	0	0	0	0	0
433	206	2	5	0	0	1
46	31	13	2	7	5	697
715	375	5	3	0	13	81
0	32	0	1	2	23	1
21	3	158	12	6	7	101
0	0	0	0	0	0	0
0	1	0	0	0	0	0
0	0	0	0	0	0	0
0	0	6	0	0	0	0
0	0	2	0	0	0	0
318	1347	1011	94	45	118	99
1057	650	5	3	10	251	0
100	2110	0	0	2	46	0
1	1380	0	1	1	40	0
163	309	1	1	0	52	1
1	13	0	0	0	1	0
0	2	0	0	0	1	0
2	5	0	0	1	2	0
0	66	0	0	0	1	0
0	1	0	0	0	0	0

0	0	0	0	0	0	0
0	0	0	0	0	0	0
0	0	0	0	0	0	0
0	0	0	0	0	0	0
0	2	0	0	0	0	0
0	0	0	0	0	0	0
0	1	0	0	0	0	0
0	3	0	0	0	0	0
0	0	0	0	0	0	0
1	0	2	1	0	0	0
0	0	0	0	0	0	0
0	2	0	0	0	0	0
0	0	0	0	0	0	0
0	0	0	0	0	0	0
11	3	10	7	0	0	0
1	0	0	0	0	0	0
0	2	0	6	0	1	1
44	12	3	6	6	3	0
14	7	0	0	1	0	6
8	0	129	0	0	0	0
0	0	0	0	0	0	0
0	0	0	0	0	0	0
2	8	0	0	0	0	0
0	0	0	0	26	0	0
0	0	0	0	0	0	0
748	337	494	10	105	245	0
98	540	0	0	0	0	1
2	98	2	0	0	6	0
0	0	0	0	0	0	0
2	2	0	0	0	0	0
0	30	0	17	2	73	3
3	2	2	0	0	6	15
0	0	0	0	0	0	0
0	2	0	0	0	1	0
0	0	0	0	1	0	0
0	0	0	0	0	0	0
0	0	0	0	0	0	0
0	0	0	0	0	0	0
3	1	0	0	0	0	0
0	0	0	0	0	0	0
0	0	0	0	0	0	0
0	0	0	0	0	0	0
0	0	1	0	0	0	0
5	2	0	1	0	0	0
21	1	21	3	1	0	0
0	0	0	0	0	0	0
0	0	0	0	0	0	0
2	2	0	0	0	0	0

0	0	0	0	0	0	0
0	0	0	0	0	0	0
426	573	3489	454	144	170	383
534	23	32	6	107	4	5
96	91	483	266	31	43	249
0	0	0	7	2	7	0
29	54	287	59	10	7	153
2	3	28	100	9	91	0
29	19	23	113	2	1	2
29	1	27	11	20	0	166
0	0	0	2	5	0	198
0	1	0	5	1	50	0
7	17	466	26	5	1	226
2	2	130	12	25	7	0
119	68	499	137	56	29	4
0	1	0	8	3	0	0
1	2	186	134	2	33	11
1	1	41	2	0	0	3
35	22	34	30	13	2	24
5	12	205	28	2	0	4
0	0	0	3	0	0	0
0	0	0	0	1	0	1
22	112	0	12	1	19	0
2	6	4	4	4	1	0
2	5	48	3	1	7	39
13	39	76	6	1	1	154
14	24	87	67	7	29	0
0	0	0	1	0	0	0
1	11	0	0	1	41	0
0	0	0	0	0	0	0
0	0	0	4	5	0	0
0	0	0	3	0	0	0
0	0	0	0	0	0	0
0	0	0	0	0	1	0
0	0	0	0	0	0	0
0	0	0	1	0	1	0
3	5	1	0	0	1	0
1	0	0	0	0	2	0
0	0	0	0	0	0	0
0	0	0	0	0	0	0
0	0	0	0	0	0	0
0	0	0	0	0	0	0
5	1	0	0	0	0	0
0	0	0	0	0	0	0
0	0	0	0	0	0	0
0	0	0	0	2	2	0
0	0	0	0	0	0	0
0	14	0	0	0	10	0

0	0	10	0	1	0	0
1	0	0	0	0	0	0
6	6	1	0	0	0	3
0	1	3	0	0	0	0
0	0	0	0	0	2	0
0	0	0	0	0	0	0
0	0	0	0	0	0	0
0	0	0	0	0	0	0
0	0	0	0	0	0	0
0	0	0	0	0	0	0
0	0	0	0	0	3	0
0	0	0	0	0	0	0
0	0	0	0	0	0	0
0	1	0	0	1	5	0
0	0	0	0	0	0	0
0	0	0	0	0	0	0
0	2	0	0	0	0	0
0	0	0	0	0	0	0
0	0	0	0	0	0	0
0	0	0	0	0	0	0
0	0	0	0	0	0	0
0	0	0	0	0	0	0
0	0	0	0	0	0	0
0	0	0	0	0	0	0
0	0	0	0	0	0	0
0	0	0	0	0	0	0
0	0	0	0	0	0	0
0	0	1	0	0	0	1
0	0	0	0	0	0	0
0	0	0	0	0	0	0
0	0	0	0	0	0	0
0	0	0	0	0	0	0
0	0	0	0	0	0	0
0	0	0	0	0	0	0
0	0	0	0	0	0	0
0	0	0	0	0	0	0
0	0	0	0	0	0	0
0	0	0	0	0	0	0
0	0	2	0	0	0	0
0	0	0	0	0	0	0
0	0	0	0	0	0	0
0	0	0	0	0	0	0
0	0	0	0	0	0	0
0	0	0	0	0	0	0
107	25	34	2	13	7	4
8	8	440	22	1	0	4
0	0	0	0	1	0	0
0	0	0	0	0	0	0

0	0	0	0	0	0	0
0	0	0	0	0	0	0
0	0	0	0	0	0	0
4	30	6	6	0	0	2
0	0	0	0	0	0	0
0	0	1	0	2	2	0
0	0	0	0	0	0	0
0	0	13	0	0	0	0
2788	300	485	60	27	50	24
1213	671	3990	524	393	282	1379
2	5	11	2	0	0	0
117	1	29	2	1	0	0
258	31	283	35	53	26	153
27	2	37	12	6	3	8
0	6	0	0	0	3	0
2	7	7	2	1	0	2
7	1	127	5	49	19	0
13	3	1	4	6	1	2
2	0	0	0	0	0	15
3	1	18	0	2	0	0
5	7	2	2	0	0	0
1	8	6	0	1	6	0
47	1	60	0	5	1	4
0	0	0	7	0	0	0
4	8	37	1	0	0	0
1	3	34	1	1	0	0
1	2	2	0	0	0	0
5	6	41	5	15	2	7
0	2	1	2	0	1	0
0	0	0	0	0	0	0
0	0	0	0	0	0	2
7	1	0	0	0	0	0
0	1	4	0	0	0	0
0	3	4	1	0	0	1
0	0	0	0	0	0	1
0	0	0	0	0	0	0
1	3	3	2	0	5	1
0	0	10	0	3	1	0
0	0	0	0	0	0	0
0	0	0	0	0	0	4
0	0	0	1	0	0	0
0	0	2	0	1	0	1
0	0	7	0	1	1	0
0	0	0	0	0	0	0
0	0	0	1	0	0	0
0	0	0	0	0	0	0
0	0	0	0	0	0	1
0	0	0	0	0	0	0

0	0	0	0	0	0	0
6	2	10	2	0	0	0
0	0	0	0	0	0	0
0	0	1	0	0	0	0
0	0	0	0	0	0	0
0	0	0	0	0	0	0
0	0	0	4	0	0	0
7	7	46	6	9	1	13
6	0	0	0	0	0	0
0	0	0	0	0	0	0
0	0	0	0	0	0	0
0	1	1	0	0	0	4
0	0	0	0	0	0	0
0	0	0	0	0	0	1
0	0	0	0	0	0	0
0	1	2	2	0	1	1
0	0	0	0	0	3	0
0	0	0	0	0	0	0
0	0	2	0	0	0	2
0	0	0	1	0	0	0
0	0	0	0	0	0	0
0	1	5	0	0	0	0
0	0	1	0	0	0	1
0	0	0	0	0	0	0
0	0	0	0	0	0	0
0	0	0	0	0	0	0
0	0	0	0	0	0	0
0	0	3	0	0	0	0
0	0	0	0	0	0	0
0	1	0	0	0	0	0
0	0	0	0	0	0	0
0	0	0	0	0	0	0
0	0	0	0	0	0	0
0	0	0	0	0	0	0
0	0	0	0	0	0	0
0	0	0	0	0	0	0
0	0	0	0	0	0	0
0	0	0	0	0	0	0
0	0	0	0	0	0	0
0	0	0	0	0	0	0
0	0	0	0	0	0	0
0	0	0	0	0	0	0
0	2	2	0	0	0	0
0	0	0	0	0	0	0
0	0	0	0	0	0	0
0	0	0	0	0	0	0
0	0	0	0	0	0	0
0	0	0	0	0	0	0

0	0	0	0	0	0	0
452	75	966	129	121	38	544
26	14	145	23	14	14	34
27	5	278	11	13	2	51
88	4	83	8	32	1	0
64	4	24	14	15	2	146
11	1	62	6	4	0	107
0	0	0	1	3	0	9
13	0	2	0	0	1	8
24	28	27	6	3	12	31
1	1	0	3	1	0	0
4	12	2	1	0	1	0
0	0	0	0	0	0	2
0	0	0	0	0	0	0
0	0	1	3	0	0	0
0	0	0	0	1	0	0
11	2	13	4	0	0	19
0	1	4	1	0	0	0
2	1	2	0	1	0	0
1	0	0	0	0	0	2
1	0	2	1	0	0	0
2	0	0	1	0	0	1
1	0	6	2	0	0	1
0	3	0	0	0	1	0
0	0	0	0	0	0	0
0	0	3	1	0	1	2
0	0	0	1	0	0	0
1	0	0	0	0	0	0
1	0	0	0	0	0	0
0	0	0	0	0	0	0
0	0	0	0	0	0	0
0	0	0	0	0	0	0
0	0	0	0	0	0	0
0	0	0	0	0	0	0
0	0	1	0	0	0	0
0	0	0	0	0	0	0
397	22	60	97	23	4	1253
9	8	4	9	0	7	11
0	0	0	1	0	0	48
98	12	25	0	1	0	0
0	1	0	1	1	0	11
0	0	0	0	0	0	0
15	0	6	0	2	0	4
0	0	0	1	1	0	0
0	0	0	0	0	0	0
0	0	0	0	0	0	0
0	0	0	0	0	0	0
2	0	0	0	0	0	0

0	0	0	0	0	0	0
0	0	0	0	0	0	0
6	0	0	0	0	0	0
0	0	0	0	0	0	0
0	0	0	0	0	0	0
0	0	0	0	0	0	0
0	0	0	0	0	0	0
0	0	0	0	0	0	1
0	0	0	0	0	0	0
0	0	0	0	0	0	0
0	0	0	0	0	0	0
0	0	0	0	0	0	0
0	0	0	0	0	0	0
3	1	0	0	0	0	0
0	0	0	0	0	0	0
0	0	0	0	0	0	0
0	0	0	0	0	0	0
1	1	10	0	6	3	1
0	0	5	0	2	3	0
0	0	0	0	0	0	0
0	6	0	1	0	4	0
0	52	0	1	1	38	0
0	0	0	0	0	0	0
0	0	0	0	0	0	0
0	0	0	0	0	0	0
0	0	0	0	0	0	0
0	0	0	0	0	0	0
0	0	0	0	0	0	0
0	0	1	1	1	0	0
0	0	0	0	1	0	0
0	0	0	1	0	0	0
0	0	0	0	0	0	0
0	0	0	0	0	0	0
61	15	250	42	31	12	119
1	0	0	0	0	0	0
0	0	0	3	0	0	0
1	0	5	1	2	0	0
0	0	0	1	0	1	0
0	0	0	0	0	0	0
0	0	0	0	0	0	0
0	0	0	0	0	0	0
0	0	0	0	0	0	0
0	0	27	2	8	0	3
0	0	0	2	0	0	0
44	7	1	2	10	0	0
0	0	0	0	0	0	0
0	0	0	0	0	0	0
2	9	7	4	8	1	0

0	0	0	0	0	0	0
114	24	287	48	43	14	10
6	0	11	7	4	0	13
0	0	0	0	0	0	0
0	0	0	0	0	0	0
0	0	0	0	0	0	0
0	0	0	0	0	0	0
0	0	0	1	0	0	0
0	3	0	0	0	1	0
135	66	123	42	16	26	310
26	10	173	54	13	3	4
11	10	1	0	0	1	0
0	1	0	0	0	6	0
0	0	0	0	0	0	0
0	0	0	0	0	0	0
0	0	0	0	0	0	0
0	0	0	0	0	0	0
0	0	0	0	0	0	0
0	0	0	0	0	0	0
0	0	0	0	0	0	0
0	0	0	0	0	0	0
0	0	0	0	0	0	0
0	0	0	0	0	0	0
0	0	5	0	0	0	0
0	0	0	0	0	0	0
0	0	0	0	0	0	0
0	0	0	0	0	0	0
0	0	0	0	0	0	0
0	0	0	0	0	0	0
0	0	0	0	0	0	0
0	0	0	0	0	0	0
0	0	0	1	0	0	0
0	0	0	0	0	0	0
0	0	0	0	0	0	0
0	0	0	4	0	1	100
0	0	10	50	0	0	200
0	0	0	3	0	0	0
0	0	0	0	0	0	0
0	0	0	0	0	0	0
0	0	0	0	0	0	0
0	19	2	50	12	7	0
2	0	0	0	0	0	0
0	0	0	0	0	0	0
0	0	0	2	0	0	0
0	0	6	0	1	0	0
0	0	0	0	0	0	0
2	1	0	0	0	0	0
0	0	0	0	0	0	0

0	8	0	2	0	11	0
0	0	0	0	0	0	0
0	0	0	0	0	0	0
28	11	0	26	0	14	0
1	0	0	5	0	0	0
0	1	0	0	0	0	0
30	136	11	0	4	15	50
0	0	0	0	0	0	0
0	3	1	0	1	3	0
0	0	0	0	0	0	0
0	0	0	0	0	0	0
0	0	0	0	0	0	2
1	2	0	2	0	5	0
0	0	0	0	0	0	0
121	1017	0	297	84	818	0
3	5	0	0	0	0	0
0	1	0	4	0	3	0
1	0	0	0	0	0	0
1	4	0	0	0	1	0
0	0	0	0	0	0	0
0	0	0	0	0	0	0
0	0	0	0	0	0	0
1	8	0	1	0	10	0
0	0	0	0	0	0	0
0	0	0	0	0	0	0
0	1	0	1	0	0	0
0	0	0	0	0	0	0

36.DCM	38.SUR	38.DCM	39.SUR	39.DCM	41.SUR	41.DCM	
0	0	0	0	0	0	0	0
0	0	0	0	0	0	0	0
0	0	0	0	0	0	0	0
0	0	0	0	0	0	0	1
0	0	0	0	0	0	0	0
0	0	0	0	0	0	0	0
0	0	0	0	0	0	0	0
0	0	1	0	0	0	0	35
0	0	3	0	0	0	0	7
0	0	0	0	0	0	0	414
0	0	0	0	0	0	0	0
0	0	0	0	0	0	0	1
0	0	0	0	0	0	0	0
0	0	0	0	0	0	0	0
0	0	0	0	0	0	0	0
0	0	0	0	0	0	0	0
0	0	17	0	0	0	0	10
254	55	97	0	822	194	526	
2	5	0	0	532	0	2	
35	1	0	0	0	2	3	
0	0	0	0	0	0	0	
0	0	0	0	0	0	0	
0	0	0	0	0	0	0	
0	0	0	0	0	0	0	
0	0	0	0	0	0	0	
0	0	0	0	0	0	0	
0	0	0	0	0	6	0	
0	0	0	0	0	0	0	
0	0	0	0	0	3	0	
1	1	0	0	3	0	3	
0	0	5	0	0	0	0	
0	0	0	0	0	0	0	
0	0	31	0	0	0	8	
0	0	0	0	0	0	5	
0	0	0	0	0	0	16	
0	0	0	0	0	0	0	
2	29	23	1	0	2611	11885	
1022	30	85	0	0	654	226	
4	0	14	0	0	1115	517	
0	0	0	0	0	0	0	
0	0	0	0	0	0	0	
0	0	0	0	0	0	0	
34	6	56	0	0	1	7	
4	0	0	0	0	4	37	
2	0	2	0	0	1	4	
0	0	0	0	0	0	0	
0	0	5	0	0	2	8	

0	0	0	0	0	0	0
0	0	0	0	0	0	6
0	0	23	0	0	0	32
0	0	5	0	0	0	30
0	0	0	0	0	0	7
0	0	0	0	0	0	5
14	0	1	0	0	0	8
0	0	0	0	0	21	7
25	2	2	0	0	1	67
0	0	0	0	0	0	0
9	0	0	0	0	0	0
0	0	0	0	0	0	2
4	0	1	0	0	0	0
1	0	0	0	0	0	1
0	0	0	0	0	0	0
0	0	0	0	0	0	0
0	0	0	0	0	0	0
0	0	0	0	0	0	0
0	0	0	0	0	0	0
0	0	0	0	0	0	0
0	0	0	0	0	0	0
0	0	0	0	0	0	0
0	0	0	0	0	0	1
0	0	0	0	0	0	3
0	0	0	0	0	0	0
0	0	0	0	0	0	0
0	0	0	0	0	5	1
0	0	0	0	0	0	0
0	0	0	0	0	0	2
0	0	0	0	0	0	0
0	0	0	0	0	0	5
0	0	2	0	0	0	0
0	0	0	0	0	0	0
0	0	0	0	0	0	0
0	0	0	0	0	0	0
0	0	0	0	0	0	0
2	0	0	0	0	0	0
0	0	0	0	0	0	0
0	0	0	0	0	0	0
765	41	232	0	0	88	418
15	1	38	0	0	1	0
2683	16	8	0	0	300	2
0	1	65	1	0	0	0
2	0	3	0	0	0	5
0	2	0	1	0	0	0
0	0	0	0	0	0	0
0	0	0	0	0	0	0
3	0	0	0	0	1	0

0	0	0	0	0	0	0
0	0	0	0	0	0	0
0	0	0	0	0	0	0
24	0	0	0	0	4	0
0	0	0	0	0	0	0
0	0	8	0	0	0	1
0	0	0	0	0	0	0
0	0	0	0	0	0	0
0	0	0	0	0	0	0
0	0	0	0	0	0	0
0	0	0	0	0	0	0
0	0	0	0	0	0	0
0	0	0	0	0	0	0
0	0	0	0	0	0	0
0	0	0	0	0	0	0
911	19	75	0	0	108	0
0	0	0	0	0	0	0
0	0	0	0	0	8	2
0	0	0	0	0	0	0
0	0	0	0	0	28	4
0	0	0	0	0	0	0
0	0	0	0	0	1	0
0	0	0	0	0	0	2
0	0	0	0	0	0	0
0	0	0	1	0	0	0
13	0	0	0	0	1	0
0	0	2	0	0	1	2
4	0	2	0	0	7	2
0	0	0	0	0	0	0
0	0	0	0	0	0	0
0	0	0	0	0	0	1
0	0	0	0	0	0	0
0	0	0	0	0	0	0
0	0	0	0	0	0	0
0	0	0	0	0	0	0
0	0	1	0	0	0	0
1	0	0	0	0	0	17
0	0	0	0	0	0	0
0	0	0	0	0	0	0
0	16	1	0	0	0	0
0	0	0	0	0	0	0
0	0	0	0	0	0	0
0	0	0	0	0	0	0
0	0	0	0	0	0	0
0	0	34	0	0	0	0
0	0	0	0	0	1	0
0	0	0	0	0	0	0
0	0	0	0	0	0	0

0	0	0	0	0	0	0
0	0	1	0	0	0	0
67	7	29	0	0	6	56
0	0	74	0	0	0	0
2	0	0	0	0	0	15
0	0	0	0	0	0	0
0	0	0	0	0	0	0
0	0	0	0	0	0	0
0	0	0	0	0	0	0
0	0	55	0	0	0	0
3	0	6	0	0	0	11
0	0	0	0	0	0	0
16	0	2	0	1928	6	209
0	0	0	0	0	0	0
0	0	5	0	0	0	2
0	0	10	0	0	0	2
0	0	0	0	0	0	16
1	0	6	0	0	0	1
0	0	0	0	0	0	0
0	0	0	0	0	0	1
7	0	1	0	7	0	0
0	0	5	0	0	0	0
0	0	0	0	0	0	0
0	0	0	0	0	0	0
0	0	0	0	0	0	0
0	0	0	0	0	0	0
0	0	0	0	0	0	0
0	0	0	0	0	107	16
0	0	4	0	0	0	0
0	0	0	0	0	0	0
0	0	1	0	0	0	7
6	0	3	0	0	0	0
0	0	1	0	0	49	61
0	0	0	0	0	0	0
0	0	0	0	0	0	0
0	0	0	0	0	0	0
0	0	0	0	0	0	0
32	0	0	0	0	3	0
0	0	0	0	0	0	0
0	0	0	0	0	0	0
0	0	0	0	0	0	0
0	0	0	0	0	0	0
166	78	908	65	244	72	117
32	13	27	2	36	2	67
0	0	0	0	0	0	3
0	0	0	0	0	0	0
3	24	31	0	0	1	0
0	0	0	0	0	0	0

0	0	0	0	0	0	0
0	0	0	0	0	0	0
0	0	0	0	0	0	0
0	16	90	0	0	0	0
0	0	0	0	0	0	0
0	0	0	0	0	0	8
0	0	0	0	0	0	0
0	0	0	0	0	0	0
0	0	0	3	1	0	0
0	0	0	0	0	0	0
4	2	3	1	1	0	0
0	0	0	0	0	0	0
0	0	0	0	0	0	0
1	2	0	0	3	3	1
0	0	0	0	0	0	0
0	0	0	0	0	0	0
0	0	0	0	0	0	0
0	0	0	0	0	0	0
0	0	0	0	0	0	0
0	0	4	0	2	0	2
0	0	0	0	0	0	0
8	0	2	0	0	0	7
0	0	0	0	0	0	0
199	211	291	0	15	745	81
4	0	0	0	0	0	0
2	0	0	0	0	0	0
0	0	0	0	0	0	0
0	0	3	0	0	0	0
0	0	0	0	0	0	0
0	0	0	0	0	0	0
0	0	0	0	0	0	0
0	0	0	0	0	0	0
0	0	0	0	0	0	0
0	0	0	0	0	0	0
0	0	0	0	0	0	0
0	0	2	0	0	0	0
40	27	40	3	76	111	108
0	3	0	0	2	2	3
0	0	0	2	0	5	0
37	0	3	4	1	1	24
0	0	0	0	0	0	0
0	0	0	0	0	0	0
0	0	0	0	0	0	0
0	0	0	0	0	0	0
0	0	0	0	0	0	0
0	0	1	0	0	0	0
1	0	0	0	0	0	0
0	0	0	0	0	0	0

0	0	0	0	0	1	8
0	20	65	0	0	0	0
1	0	0	0	0	0	0
0	0	0	0	0	0	0
0	0	0	0	0	0	0
0	0	0	0	0	0	0
0	0	0	0	0	0	0
0	0	0	0	0	0	0
0	0	0	0	0	0	0
0	0	0	0	0	0	0
0	0	0	0	0	0	0
0	0	0	0	0	0	0
0	0	0	0	0	0	0
0	0	0	0	0	0	0
0	0	0	0	0	0	0
0	0	0	0	0	0	0
0	0	0	0	0	0	0
0	0	0	0	0	0	0
0	0	0	0	0	0	0
0	0	0	0	0	0	0
19	3	0	0	2	1	0
0	55	21	0	0	3	0
0	0	0	0	0	0	0
39	43	41	1	0	5	115
0	0	0	0	0	25	5
0	24	0	0	0	0	6
1	0	0	0	0	2	0
0	0	0	0	0	0	0
0	0	0	0	0	0	1
0	0	0	0	0	0	0
0	0	0	0	0	0	0
0	0	3	0	0	0	0
0	0	0	0	0	0	0
0	0	0	0	0	0	0
0	0	0	0	0	0	0
0	0	0	0	0	0	0
0	0	0	0	0	0	0
0	0	0	0	0	0	0
0	0	2	0	0	0	4
0	0	1	0	0	0	0
0	0	0	0	0	0	0
0	0	0	0	0	0	0
0	0	0	0	0	0	0
0	0	0	0	0	0	0
0	0	0	0	0	0	0
2	0	0	0	3	0	0
0	0	0	0	0	0	0
0	0	0	0	0	0	0

0	0	0	0	0	0	0
0	0	0	0	0	0	0
0	0	0	0	0	0	0
23	1	9	5698	58	319	32
0	0	75	0	0	0	2
0	0	2	0	0	0	0
455	38	218	15	19	23	90
0	0	0	41	35	0	0
2	0	0	16	8	0	3
0	0	13	0	0	0	0
0	2	0	0	1	0	0
8789	1307	2401	6	6321	4007	3033
64	34	0	0	0	9	7
4	70	106	0	0	1	264
5	0	44	1	0	1	283
300	12	85	0	0	30	135
84	11	78	0	1	10	132
0	1	0	0	0	0	14
0	1	29	0	0	0	72
0	0	52	0	0	0	317
0	0	0	1	0	0	79
27	0	0	0	0	6	92
0	235	715	0	0	0	24
0	0	0	0	0	0	9
0	0	0	0	0	0	0
0	0	0	0	0	0	0
0	299	583	0	0	0	0
0	0	0	0	0	0	0
0	0	0	0	0	0	0
7	0	1	0	0	0	19
0	0	0	0	0	0	10
0	0	0	0	0	0	0
2	0	0	0	0	0	0
0	0	0	0	0	0	0
0	184	281	0	0	0	0
0	0	0	0	0	0	0
245	41	62	1	438	44	32
0	0	0	0	0	0	0
0	0	0	0	0	0	0
0	0	0	0	0	0	3
0	0	0	0	0	0	0
0	0	0	0	0	0	10
30	0	0	0	0	5	22
0	0	0	0	0	0	0
0	0	0	0	0	0	0
0	2	1	0	0	0	0
0	0	0	0	0	0	14
0	0	0	0	0	0	0

0	0	0	0	0	0	0
8	1	1	0	8	0	1
0	0	0	0	0	0	0
0	0	0	0	0	0	1
0	0	0	0	0	0	0
0	0	0	0	0	0	0
0	0	0	0	0	0	2
2	0	0	0	0	0	0
0	0	0	0	0	0	4
0	0	0	0	0	0	0
0	0	0	0	0	0	0
0	0	0	0	0	0	0
1	0	0	0	1	0	0
0	0	0	1	0	0	0
0	0	0	0	0	0	3
0	0	0	0	0	0	0
0	0	0	0	0	0	0
0	0	0	0	0	0	0
0	0	0	0	0	12	5
0	0	0	0	0	0	0
0	0	0	0	0	0	0
37	1	25	0	0	4	22
0	0	0	0	0	0	0
0	0	0	0	0	0	0
0	0	0	0	0	0	0
5	0	1	0	0	2	0
0	0	0	0	0	0	0
0	0	0	0	0	0	0
2	0	0	0	0	0	0
0	0	0	0	0	0	0
3	0	5	0	0	0	4
9	0	0	0	4	0	0
2	2	2	0	3	1	1
15	7	4	0	18	0	0
0	0	0	0	0	0	0
1	0	0	0	0	0	2
1	0	0	0	0	0	4
0	0	0	0	0	0	0
0	0	0	0	0	0	0
0	0	1	0	0	0	2
4	0	1	0	0	0	0
0	0	0	0	0	0	0
0	0	0	0	0	0	0
0	0	0	0	0	0	0
0	0	0	0	0	0	0
0	0	0	0	0	0	0
0	0	0	0	0	0	0
0	0	0	1	0	0	0

2	0	6	0	0	4	2
4	1	0	0	3	0	0
0	0	0	0	0	0	0
0	0	0	0	0	0	0
4	0	0	0	0	1	0
3	0	0	0	0	0	0
0	0	0	0	0	0	0
1	0	0	0	0	0	0
0	0	0	0	0	0	0
7	5	0	0	10	0	2
0	0	0	0	0	0	0
0	0	0	0	0	0	0
0	0	0	0	0	0	0
0	0	0	0	0	0	0
0	0	0	0	0	0	0
0	0	0	0	0	0	0
0	0	0	0	0	0	0
5	0	0	0	0	1	0
0	0	0	0	0	0	0
0	0	0	0	0	0	0
0	0	0	0	0	0	0
0	0	0	0	0	0	0
0	0	0	0	0	3	0
0	0	0	0	0	0	0
1	1	1	0	2	2	0
0	0	0	0	0	0	1
0	0	0	0	0	0	0
0	0	0	0	0	0	0
0	4	6	0	0	0	0
0	0	0	0	1	0	92
0	0	0	0	0	0	39
7	0	9	0	0	1	32
0	0	0	0	0	0	0
0	0	0	0	0	0	0
0	0	0	0	0	0	0
0	0	16	0	0	0	59
0	0	0	0	0	0	119
0	1	0	7	1	0	36
0	0	1	0	0	0	30
0	0	0	0	0	0	0
0	0	0	0	0	0	0
0	0	0	0	0	0	1
58	0	0	0	0	8	0
0	0	0	0	0	0	1
1	0	1	0	0	1	0
0	0	0	0	0	0	0
0	0	0	0	0	0	0
0	0	0	0	0	0	0

0	0	12	0	0	0	21
0	0	0	1	0	0	0
0	0	0	0	0	0	0
18	6	0	0	28	0	21
0	0	0	0	0	0	6
0	0	0	0	0	0	0
5	58	2	0	0	1378	467
0	161	270	0	0	0	12
3	0	13	1	0	1	149
0	0	0	0	0	0	21
0	0	1	0	0	0	16
0	0	1	0	0	0	16
0	0	0	0	0	0	9
0	0	0	0	0	0	0
0	0	0	0	0	0	4
0	0	0	0	0	0	0
0	0	0	0	0	0	0
0	0	0	0	0	0	0
0	4	3	2	0	0	25
0	0	0	0	0	0	0
0	0	0	0	0	0	0
0	0	0	0	0	0	20
0	0	0	0	0	0	5
0	0	0	0	0	0	0
0	0	14	0	0	0	0
0	0	0	0	0	0	0
11	0	1	0	0	7	3
0	0	0	0	0	0	0
0	0	0	0	0	0	0
0	0	0	0	0	0	0
0	0	0	0	0	0	1
0	0	0	0	0	0	0
14	0	0	0	0	1	4
0	0	0	0	0	0	3
0	0	0	0	0	0	0
0	0	0	0	0	0	0
0	1	0	0	1	0	1
0	0	0	0	0	0	0
0	0	0	0	0	0	0
1	0	0	0	0	0	0
0	0	0	0	0	0	4
0	0	0	0	0	0	0
0	0	3	0	0	0	9
1	0	1	0	0	0	8
0	0	0	0	0	0	0
0	0	0	0	0	0	0
0	0	0	0	0	0	0
0	0	0	0	0	0	0

0	0	0	0	0	0	0
0	0	0	0	0	0	0
23	51	37	0	4	4	0
0	0	0	0	0	0	0
0	0	0	0	0	0	0
0	0	0	0	0	0	0
0	0	0	0	0	0	0
0	0	0	0	0	0	0
0	0	0	0	0	0	0
0	0	1	0	0	0	0
0	0	0	0	0	0	0
0	0	0	0	0	0	0
1	4	0	0	0	0	1
0	0	0	0	0	0	0
0	0	0	0	0	0	0
0	0	0	0	0	0	0
0	0	0	0	0	0	1
0	0	5	0	0	0	0
3	0	0	0	0	0	0
0	6	0	0	0	0	17
0	0	0	0	0	0	0
0	0	0	0	0	84	61
0	0	0	0	0	0	0
0	0	0	0	0	0	0
9	0	0	0	0	0	4
0	0	0	0	0	0	0
0	0	0	0	0	0	0
0	0	80	0	0	0	2
0	0	0	0	0	0	0
0	0	0	0	0	2	0
0	0	0	0	0	0	0
117	12	1	0	0	8	10
0	0	0	0	0	0	0
0	0	0	0	0	0	0
0	0	0	0	0	0	0
0	0	0	0	0	0	0
0	0	0	0	0	0	0
0	0	0	0	0	0	0
0	0	0	0	0	0	0
0	0	0	0	0	0	0
5	1	2	0	0	52	10
0	0	0	0	0	0	0
0	0	0	0	0	0	0
657	44	356	2	3	222	227
6	0	76	0	0	28	86
628	88	167	76	116	374	1382
31	38	67	20	121	308	369
17	14	6	3	0	43	39

882	13	80	0	0	111	9
236	17	15	7	0	53	16
8	0	0	0	0	5	27
0	0	0	0	0	0	1
0	4	0	0	0	0	1
4	0	0	0	0	26	8
1	0	0	0	0	0	1
6	0	4	1	0	8	10
2	0	1	0	0	0	0
0	0	0	0	0	0	10
3	0	1	0	0	1	11
14	0	0	0	0	4	5
69	0	1	0	0	9	0
0	0	0	0	0	0	0
0	0	0	0	0	0	1
4	0	0	0	0	0	1
11	0	7	0	0	2	0
2	0	4	0	0	2	10
3	0	0	0	0	0	0
2	0	0	0	0	0	0
0	0	0	0	0	1	2
0	0	2	0	0	0	0
0	0	0	0	0	0	0
0	0	0	0	0	0	0
0	0	0	0	0	0	0
2	0	1	0	0	1	8
0	0	0	0	0	0	4
0	0	0	0	0	1	0
0	0	0	0	0	0	0
1	3	2	0	2	0	1
0	0	0	0	0	0	0
0	0	0	0	0	0	0
0	15	0	0	0	0	1
0	0	0	0	0	0	0
0	0	0	0	0	0	0
0	0	0	0	0	0	0
0	0	0	0	0	0	0
0	0	0	0	1	3	3
0	0	0	0	0	0	0
0	0	0	0	0	0	0
0	0	0	0	0	0	0
0	0	0	0	0	0	4
0	0	0	0	0	0	0
0	0	0	0	0	0	0
0	0	0	0	0	0	0
0	0	0	0	0	0	0
0	0	0	0	0	0	0
0	0	0	0	0	0	0

0	0	0	0	0	0	0
0	0	0	0	0	0	0
0	0	0	0	0	0	0
0	0	0	0	0	0	0
0	35	30	0	0	0	2
28	4	0	1	0	5	7
0	0	0	0	0	0	0
15	1	110	0	0	9	223
0	0	2	0	0	0	1
1	3	1	0	1	7	0
0	0	0	0	0	0	6
0	2924	74	0	0	2	0
84	12	24	0	8	0	40
0	0	4	0	0	0	0
1	1424	161	0	0	0	4
0	0	69	0	0	0	0
0	0	0	0	0	0	0
1	0	0	0	0	0	0
0	0	0	0	0	0	0
0	0	0	0	0	0	0
0	0	0	0	0	0	0
0	38	1	0	0	0	0
0	0	0	0	1	1	2
0	0	0	0	0	0	0
0	0	0	0	0	0	0
0	0	0	0	0	0	0
0	0	0	0	0	0	0
0	0	0	0	0	0	0
0	0	0	0	0	0	0
0	0	0	0	0	0	0
5	2	0	0	0	39	5
70	2	1	0	0	5	0
0	0	0	0	0	0	0
0	0	0	0	0	0	0
0	0	0	0	0	0	0
13	1	4	0	0	0	50
15	4	1	0	0	603	99
0	0	0	0	0	0	0
29	0	4	0	0	3	0
0	0	0	0	0	0	0
0	0	0	0	0	0	0
0	0	0	0	0	0	0
0	0	0	0	0	0	0
0	0	0	0	0	0	0
0	0	0	0	0	0	0
0	0	0	0	0	0	0
0	0	0	0	0	0	0
0	0	0	0	0	0	24

0	0	0	0	0	0	0
4	0	0	0	0	3	0
0	0	0	0	0	0	0
0	0	0	0	0	0	0
0	0	0	0	0	0	0
0	0	0	0	0	0	0
0	0	0	0	0	0	17
0	0	0	0	0	0	0
0	0	0	0	0	0	0
0	0	0	0	0	0	0
0	0	0	0	0	0	0
0	0	0	0	0	0	0
0	0	9	0	0	0	12
0	0	0	0	0	0	0
24	3	21	0	0	3	4
75	1	4	0	0	4	0
0	0	0	0	0	0	0
5	81	11	0	0	2	0
0	0	0	0	0	1	1
0	1	4	0	0	0	9
0	4	14	0	0	0	0
13	0	0	0	0	0	0
1	0	1	0	0	1	3
12	2	1	0	0	0	0
0	0	0	0	0	0	3
14	2	0	0	0	23	25
2	1	0	0	1	0	1
0	0	1	0	0	0	2
0	0	0	0	0	0	2
0	0	0	0	0	0	0
0	0	0	0	0	0	0
0	0	0	0	0	0	0
0	0	0	0	0	0	0
0	0	0	0	0	0	0
0	0	0	0	0	0	0
0	0	0	0	0	0	1
0	0	0	0	0	0	0
0	0	0	0	0	0	0
0	0	0	0	0	0	0
0	0	0	0	0	0	0
0	0	0	0	0	0	0
0	0	0	0	0	0	0
0	3	0	0	0	0	0
0	0	0	0	0	0	0
0	0	0	0	0	0	0
0	0	0	0	0	0	0
0	0	0	0	0	0	0
0	0	0	0	0	0	0

0	0	0	0	0	0	0
46	18	39	1	1	17	32
124	35	28	0	2	28	31
10	9	19	0	0	17	9
5	1	1	0	0	1	0
0	0	0	0	0	0	0
34	1	3	0	0	3	3
0	1	0	0	0	0	0
0	0	0	0	0	0	0
0	2	7	0	0	5	3
0	0	0	0	0	0	0
0	0	0	0	0	0	0
0	0	0	0	0	0	0
16	0	0	0	0	0	0
1	0	0	0	0	6	3
0	0	0	0	0	0	0
0	0	0	0	0	0	2
0	0	0	0	0	0	0
0	0	0	0	0	0	0
0	0	0	0	0	0	0
0	0	0	0	0	0	1
0	0	0	0	0	0	0
0	0	0	0	0	0	0
0	0	0	0	0	0	1
0	0	1	0	0	0	0
0	0	0	0	0	0	0
0	0	0	0	0	0	0
0	0	0	0	0	0	0
0	0	0	0	0	0	0
0	0	0	0	0	0	0
6	1	3	0	0	0	0
0	0	0	0	0	0	0
0	46	1311	0	0	0	13
235	39	96	0	1	37	11
0	0	20	0	0	36	21
0	1	17	1	0	0	22
43	24	20	0	1	3	12
13	27	13	0	491	0	0
0	6	0	0	0	0	12
27	0	3	0	0	8	5
0	0	0	0	0	3	4
28	2	13	0	0	1	2
0	0	0	0	0	0	96
0	0	20	0	0	0	3
23	0	6	0	0	2	0
31	4	0	0	0	1	0
0	16	3	0	0	0	0
1	0	0	0	0	0	0

0	0	0	0	0	0	0
0	0	0	0	0	0	0
0	0	0	0	0	0	0
0	0	0	0	0	0	0
0	0	0	0	0	0	0
0	0	0	0	0	0	0
0	0	0	0	0	0	0
0	0	0	0	0	0	0
0	0	3	0	0	0	0
0	0	4	0	0	0	0
0	0	0	0	0	0	0
0	0	0	0	0	0	0
0	0	0	0	0	0	0
0	0	0	0	0	0	0
0	0	0	0	0	0	0
0	0	0	0	0	0	0
0	0	0	0	0	0	0
0	0	0	0	0	0	0
0	0	0	0	0	0	0
0	0	0	0	0	0	0
0	0	0	0	0	0	0
0	0	0	0	0	0	0
0	0	0	0	0	0	1
0	0	0	0	0	2	0
0	0	0	0	0	0	0
0	0	0	0	0	0	0
8	0	0	0	0	2	1
0	0	0	0	0	0	0
0	0	0	0	0	0	0
1	0	0	0	0	0	12
0	0	0	0	0	0	2
0	0	0	0	0	0	0
0	0	1	0	0	0	2
0	0	0	0	0	0	1
0	0	0	0	0	0	0
0	0	0	0	0	0	0
1	0	0	0	0	0	0
0	0	0	0	0	0	0
0	0	0	0	0	0	0
0	0	0	0	0	0	0
0	0	0	0	0	0	0
74	0	2	0	0	3	38
0	0	0	0	0	0	0
0	0	20	0	0	0	1
0	0	0	0	0	0	0
0	0	0	0	0	0	0
0	0	0	0	0	0	0
0	0	0	0	0	0	0

65	34	712	25	26	39	716
16	30	46	4	0	5	36
0	0	0	0	0	0	0
0	0	0	0	0	929	53
0	1	2	0	0	2	1
0	0	1	0	0	0	0
0	0	0	0	0	0	0
0	0	0	0	0	0	0
0	0	0	0	0	0	0
0	0	0	0	0	0	0
0	0	0	0	0	0	0
0	0	0	0	0	0	0
4	14	3	0	0	0	7
0	2	5	0	0	0	817
0	1	0	0	0	22	33
0	0	0	0	0	0	0
0	0	0	0	0	0	6
0	0	0	0	0	0	0
0	0	0	0	0	0	0
0	1	0	0	0	103	36
0	0	0	0	0	16	10
0	0	0	0	0	0	0
0	0	0	0	0	0	0
0	0	0	0	0	0	0
74	41	89	367	226	1089	208
0	0	0	0	0	0	0
0	0	0	0	0	0	0
0	64	1301	0	2	9522	354
0	1	4	0	1875	1	0
15	57	2	2	5	211	82
110	13	11	1	6	98	9
0	13	0	0	0	0	21
2	3	3	0	1	5	22
0	0	0	0	4	60	13
0	1	0	0	0	26	30
0	0	0	0	0	5	4
13	0	0	8	1	38	9
0	0	0	0	0	0	0
0	0	13	0	0	9	0
0	0	0	0	0	0	0
0	0	0	0	0	0	0
0	0	0	0	0	0	0
0	0	0	0	0	0	0
0	1	0	0	0	3967	264
127	84	92	140	89	607	306
0	0	0	0	0	0	0
0	0	0	0	0	47	6
0	1	0	0	0	12	0

0	0	0	0	0	3	0
0	0	0	0	0	31	1
1	0	0	0	0	44	1
0	0	0	0	0	61	3
0	0	0	0	0	6	2
0	0	0	0	0	0	0
0	0	0	0	0	0	0
0	0	0	0	0	0	0
0	0	0	0	0	4	2
0	0	1	1	0	0	72
0	0	0	0	0	0	3
0	0	0	0	0	1	1
0	0	0	0	0	0	5
0	0	0	0	0	0	0
0	0	0	0	0	0	0
2	141	66	263	140	2422	401
0	4	36	0	0	1	325
0	2	0	6	6	11	2
0	0	0	0	0	0	0
0	0	0	0	0	5	0
0	0	0	0	0	6	0
0	0	0	0	0	0	0
0	0	0	0	0	0	0
0	0	0	0	0	0	0
0	0	0	0	0	17	1
0	1	0	3	3	7	2
0	2	0	0	0	2	0
0	0	0	1	0	0	0
10	10	8	10	0	1016	63
211	169	39	54	24	173	59
30	0	2	218	62	142	129
3	0	0	3	2	21	43
181	16	1	0	3	17	14
0	0	0	0	0	124	9
0	0	0	0	0	31	8
0	0	0	0	0	41	3
0	0	0	0	0	0	0
0	0	0	0	0	0	0
108	92	351	38	34	10	175
1	1	106	24	17	1	88
1	0	17	0	0	0	213
0	0	0	0	0	0	264
1	0	13	0	1	2	99
0	0	0	0	0	0	0
0	0	0	0	0	0	0
0	0	1	0	0	0	0
0	0	0	0	0	0	2
0	0	0	0	0	0	0

0	0	0	0	0	0	0
0	0	0	0	0	0	0
0	0	0	0	0	0	0
0	0	0	0	0	0	0
0	0	0	0	0	0	2
0	0	0	0	0	0	0
0	0	2	0	0	0	0
0	0	0	0	0	0	0
0	0	0	0	0	0	0
0	0	0	0	0	0	0
0	0	0	0	0	0	0
0	0	0	0	0	0	0
0	0	0	0	0	0	0
0	0	0	0	0	0	0
0	0	0	0	0	0	0
0	0	0	0	0	0	0
6	2	1	0	0	0	6
0	0	0	4	4	196	26
0	0	0	1	0	43	6
0	0	0	0	0	0	0
0	0	0	0	0	0	1
0	0	0	0	0	0	0
0	0	0	0	0	0	0
0	0	0	0	0	0	0
0	0	0	0	0	0	0
2	0	0	31	23	438	87
8	1	0	0	0	0	0
1	0	0	1	0	2	3
0	0	0	0	0	0	0
0	0	3	0	0	0	1
0	0	0	0	0	0	0
2	2	0	0	0	191	18
0	0	0	0	0	0	1
0	0	0	0	0	0	2
0	0	0	0	0	0	0
0	3	0	0	0	0	0
0	0	0	0	0	0	0
0	0	0	0	0	0	0
0	0	0	0	0	0	0
0	1	16	0	0	0	0
0	0	0	0	0	0	0
0	0	0	0	0	0	0
0	0	0	0	0	1	0
0	0	5	0	0	0	0
0	0	1	0	0	4	0
0	0	0	0	0	0	0
0	0	0	0	0	0	0
0	0	0	0	0	0	0

0	0	0	0	0	0	0
0	0	0	0	0	0	0
475	149	201	5	11	511	391
22	176	0	0	5	220	92
364	194	132	2	4	365	207
15	3174	5043	1	0	0	38
328	39	82	0	0	83	107
13	12	104	2	0	3	192
10	0	5	0	0	1	15
295	3	4	1	5	70	33
322	7	7	1	0	34	48
0	0	114	0	0	0	53
498	7	30	0	0	43	4
6	18	28	0	0	1	30
43	7	80	5	4	28	19
7	0	5	0	0	0	14
73	0	7	0	0	15	36
22	3	48	0	0	3	79
63	0	26	0	1	12	62
0	3	8	0	0	0	2
0	0	0	0	0	0	4
1	0	0	0	0	0	0
0	1	5	0	0	0	36
0	0	18	0	0	0	0
38	0	11	0	0	8	1
362	5	33	0	0	46	6
0	0	0	1	0	0	5
0	0	135	0	1	0	0
0	0	0	0	0	0	7
0	0	0	0	0	0	0
0	0	0	0	0	0	8
0	0	0	0	0	0	0
0	0	0	0	0	0	0
0	1	19	0	0	0	0
0	0	34	0	0	0	52
0	0	0	0	0	0	0
0	0	0	0	0	0	6
0	0	5	0	0	0	2
2	0	1	0	0	3	0
0	0	0	0	0	0	0
0	0	0	0	0	0	0
0	0	0	0	0	0	0
1	0	0	0	0	3	2
0	1	0	0	0	0	5
0	0	0	0	0	0	0
0	0	15	0	0	0	0
0	0	0	0	0	0	0
0	0	0	0	0	0	0

2	1	3	2	0	1	0
0	0	0	0	0	0	0
3	0	0	0	0	0	0
0	0	0	0	0	0	0
0	0	0	0	0	0	0
0	0	0	0	0	0	0
0	0	0	0	0	0	0
0	0	0	0	0	0	0
0	0	1	0	0	0	2
0	0	0	0	0	0	0
0	0	0	0	0	0	0
0	0	0	0	0	0	0
0	0	0	0	0	0	0
0	0	0	0	0	0	0
0	0	0	0	0	0	0
0	0	0	0	0	0	0
0	0	0	0	0	0	1
0	0	0	0	0	0	0
0	0	0	0	0	0	0
0	0	0	0	0	0	0
0	0	0	0	0	0	0
0	0	0	0	0	0	0
0	0	0	0	0	0	0
0	0	0	0	0	0	0
0	0	0	0	0	0	0
0	0	0	0	0	0	0
0	0	0	0	0	0	0
2	0	0	0	0	0	1
0	0	0	0	0	0	0
0	0	0	0	0	0	0
0	0	0	0	0	0	0
0	0	0	0	0	0	0
0	0	0	0	0	0	0
0	0	0	0	0	0	0
0	0	0	0	0	0	2
0	0	0	0	0	0	0
0	0	0	0	0	0	0
0	0	0	0	0	0	0
0	0	0	0	0	0	0
0	0	0	0	0	0	0
0	0	1	0	0	0	0
0	0	0	0	0	0	0
0	0	0	0	0	0	0
0	0	0	0	0	0	0
9	2	1	0	0	34	9
0	0	0	0	0	0	2
0	0	0	0	0	0	0
0	0	1	0	0	0	0

0	0	0	0	0	0	0
0	0	0	0	0	0	6
0	0	0	0	0	0	0
42	0	3	0	0	2	8
0	0	0	0	0	2	0
1	0	2	0	0	6	7
0	0	0	0	0	0	0
0	0	0	0	0	0	0
52	8	80	0	397	17	73
2551	1116	732	2	150	741	559
0	16	3	0	0	0	5
0	8	17	0	0	46	15
200	75	12	0	1	113	84
20	10	3	0	0	3	4
0	0	57	0	0	1	0
7	2	1	0	0	0	2
0	0	0	0	0	184	26
0	2	1	0	0	7	10
64	0	1	0	0	9	2
1	1	2	0	0	1	3
0	0	0	0	0	8	2
8	0	1	0	0	0	7
2	0	9	0	0	4	11
0	0	0	0	0	0	2
0	0	0	0	0	0	0
0	1	2	0	0	0	7
4	0	0	0	0	0	3
13	2	12	0	0	1	2
2	0	6	0	0	0	0
0	0	0	0	0	0	0
0	1	1	0	0	0	0
0	0	0	0	0	0	0
0	2	0	0	0	0	1
4	1	0	0	0	0	0
22	0	1	0	0	5	0
0	0	0	0	0	0	0
1	0	1	0	0	0	0
1	1	1	0	0	5	0
0	0	0	0	0	0	0
0	0	0	0	0	0	0
0	0	0	0	0	0	0
7	1	0	0	0	0	0
0	0	1	0	0	0	1
0	0	0	0	0	0	0
0	0	1	0	0	0	0
0	0	0	0	0	0	0
1	1	0	0	0	0	0
0	0	0	0	0	0	0

0	0	0	0	0	1	1
3	1	0	0	0	0	0
0	0	0	0	0	0	0
0	0	0	0	0	0	0
0	0	0	0	0	0	0
0	0	0	0	0	0	0
0	0	0	0	0	0	0
14	4	5	0	0	5	2
0	0	0	0	0	0	0
0	0	0	0	0	0	0
1	0	1	0	0	1	0
1	0	0	0	0	0	0
0	0	0	0	0	0	0
0	0	0	0	0	0	0
0	0	0	0	0	0	0
2	0	2	0	0	1	2
0	0	0	0	0	0	2
0	0	0	0	0	0	0
2	0	0	0	0	1	0
0	0	0	0	0	0	0
0	0	0	0	0	0	0
0	0	0	0	0	0	0
1	1	0	0	0	0	0
0	0	0	0	0	0	1
0	0	0	0	0	0	0
0	0	0	0	0	0	0
0	0	0	0	0	0	0
0	0	0	0	0	0	0
0	0	0	0	0	0	0
0	0	0	0	0	0	0
0	0	0	0	0	0	0
0	0	0	0	0	0	0
0	0	0	0	0	0	0
0	0	0	0	0	0	0
0	0	0	0	0	0	3
0	0	0	0	0	0	0
0	0	0	0	0	0	0
0	0	0	0	0	0	0
0	0	0	0	0	0	0
0	0	0	0	0	0	0
0	0	0	0	0	0	0
0	0	0	0	0	0	0
1	1	0	0	0	0	0
0	0	0	0	0	0	0
0	0	0	0	0	0	0
2	0	1	0	0	0	0
0	0	0	0	0	0	0
0	0	0	0	0	0	0

0	0	0	0	0	0	0
877	47	37	2	1	348	163
47	49	83	0	0	18	22
54	4	2	0	0	17	5
0	24	29	0	0	31	19
268	34	14	0	0	71	77
65	2	0	2	0	46	3
17	20	4	0	0	6	2
7	2	0	0	0	0	11
49	6	15	0	0	31	29
0	0	0	0	0	0	4
4	0	0	0	0	1	5
5	0	0	0	0	5	1
0	0	0	0	0	0	0
4	0	0	0	0	3	1
0	0	0	0	0	0	0
36	16	7	0	0	14	6
2	0	1	0	0	2	0
0	0	0	0	0	0	0
2	0	0	0	0	1	0
0	0	2	0	0	0	0
5	0	1	0	0	0	2
0	0	0	0	0	1	0
0	0	0	0	0	0	0
0	0	0	0	0	0	0
7	0	1	0	0	1	0
0	0	0	0	0	0	0
1	0	0	0	0	0	0
0	0	0	0	0	0	0
0	0	0	0	0	0	0
4	0	0	0	0	0	0
0	0	0	0	0	0	1
0	0	0	0	0	0	0
0	0	0	0	0	0	0
1	0	0	0	0	0	0
0	0	0	0	0	0	0
58389	175	76	14	22	7273	201
10	0	3	0	0	3	1
14	1	0	7	4	91	16
0	0	0	0	0	2	0
24	0	1	0	0	7	11
0	0	0	0	0	0	0
12	0	0	0	0	32	0
1	0	0	0	0	0	0
110	0	0	0	0	16	0
0	0	0	0	0	1	0
6	0	0	0	0	0	0
0	0	0	0	0	0	0

0	0	0	0	0	0	0
0	0	0	0	0	0	0
0	4	0	0	0	1	0
0	0	0	0	0	0	0
0	0	0	0	0	0	0
26	0	1	0	0	3	0
0	0	0	0	0	0	0
4	0	0	0	0	1	0
0	0	0	0	0	0	0
0	0	0	0	0	0	0
0	0	0	0	0	3	0
0	0	0	0	0	0	0
0	0	0	0	0	0	0
0	1	0	0	0	0	0
1	0	0	0	1	0	0
0	0	0	0	0	0	0
0	0	0	0	0	0	0
1	0	0	0	0	8	4
0	0	0	0	0	0	67
0	0	0	0	0	0	0
0	0	0	0	0	0	0
0	19	0	1	0	0	3
0	0	0	0	0	0	26
0	0	0	0	0	0	0
0	0	0	0	0	0	0
0	0	13	0	0	0	3
0	0	0	0	0	0	2
0	0	0	0	0	0	0
0	0	0	0	0	16	0
0	0	0	0	0	2	2
0	0	0	0	0	0	0
0	0	0	0	0	0	0
0	0	0	0	0	0	0
131	146	29	0	2	75	22
0	2	0	0	0	0	1
0	0	0	0	0	0	0
0	0	0	0	0	0	0
0	0	0	0	0	0	0
0	0	0	0	0	0	0
0	0	0	0	0	0	0
0	0	0	0	0	0	0
0	0	0	0	0	0	0
3	3	3	1	0	0	0
0	0	0	0	0	0	0
0	2	0	0	0	1	2
0	0	0	0	0	0	0
0	0	9	0	0	0	0
3	0	5	0	0	5	4

0	0	0	0	0	0	0
5	23	26	0	2	71	52
6	7	11	0	1	11	3
0	0	0	0	0	0	0
0	0	0	0	0	0	0
0	0	0	0	0	0	0
0	0	0	0	0	0	0
0	0	0	0	0	0	0
0	0	0	0	0	0	0
325	7	86	0	0	89	68
1	0	4	0	0	50	27
0	0	1	0	0	0	5
0	0	0	0	0	0	0
0	0	0	0	0	0	0
0	0	0	0	0	0	0
0	0	0	0	0	0	0
0	0	0	1	0	0	0
0	0	0	0	0	0	0
0	0	0	0	0	0	0
0	0	0	0	0	0	0
0	0	8	0	0	0	0
0	0	0	0	0	0	0
0	0	0	0	0	0	0
0	0	0	0	0	0	0
0	0	0	0	0	0	0
0	0	0	0	0	0	0
0	0	0	0	0	0	0
0	0	0	0	0	0	0
0	0	0	0	0	0	0
0	316	3	0	0	0	0
0	10	0	0	0	0	0
15	0	3	0	0	2	3
0	0	0	0	0	0	0
0	0	0	0	0	0	0
1045	0	5	0	0	103	5
442	0	4	0	0	87	0
0	0	0	0	0	0	0
0	0	0	0	0	0	0
0	0	0	0	0	0	0
0	0	0	0	0	0	0
0	0	0	0	0	0	18
0	0	0	0	0	0	4
0	0	0	0	0	0	0
0	0	0	0	0	0	0
0	0	0	0	0	0	0
0	0	0	0	0	0	0
0	0	0	0	0	0	0
0	0	0	0	0	0	0

0	0	1	0	0	0	0
0	0	0	0	0	0	0
0	0	0	0	0	0	0
1	0	6	1	0	0	0
0	0	0	0	0	0	0
3	0	0	0	0	0	0
73	1	8	1	0	5	56
0	0	0	0	0	0	0
0	0	0	0	0	0	1
0	0	0	0	0	0	0
0	0	0	0	0	0	0
4	0	0	0	0	0	0
0	0	0	0	0	0	0
0	0	0	0	0	0	0
1	0	24	0	1	0	141
0	0	0	0	0	0	0
0	0	0	0	0	0	0
0	0	0	0	0	0	10
0	0	0	0	0	0	0
0	0	0	0	0	0	0
0	0	0	0	0	0	0
0	0	0	0	0	0	0
0	0	0	0	0	0	0
0	0	0	0	0	0	0
0	0	0	0	0	0	0
0	0	0	0	0	0	0
0	0	0	0	0	0	0

42.SUR	42.DCM	44.SUR	45.SUR	48.SUR	52.SUR	52.DCM
0	0	0	0	0	0	0
0	0	0	0	0	0	0
0	2	0	0	0	0	0
0	0	0	0	0	0	0
0	1	0	0	0	0	0
0	0	0	0	0	0	0
0	0	0	0	0	0	0
0	54	0	0	0	0	11
0	10	0	0	0	0	11
0	0	0	0	0	0	0
0	0	0	0	0	0	0
0	0	0	0	0	0	1
0	0	0	0	0	0	0
0	0	0	0	0	0	0
0	2	0	0	0	0	4
0	0	0	0	0	0	1
0	62	0	0	0	0	25
310	659	34	673	213	158	285
2	4	1	1	1	1	0
0	3	29	21	20	4	0
0	3	0	0	0	0	0
0	0	19	0	0	2	1
0	0	0	0	0	0	0
0	0	0	0	0	0	0
0	1	0	0	0	0	0
0	0	0	0	0	0	0
1	0	0	0	0	0	0
0	0	0	0	0	0	0
0	0	0	0	0	0	0
0	3	0	0	0	0	0
0	0	0	0	0	0	0
0	0	0	0	0	0	0
0	3	0	0	0	0	8
0	0	0	0	0	0	1
1	2	0	0	0	0	14
0	0	0	0	0	0	0
64878	1190	36261	4006	47187	2878	1208
362	411	121	853	403	534	154
614	155	395	197	306	148	209
0	0	0	0	0	0	0
0	13	0	0	0	0	20
0	0	2	0	0	0	4
2	49	0	0	0	2	5
0	54	0	0	0	7	9
0	8	0	0	0	0	10
0	11	0	0	0	0	10
0	20	0	0	0	1	9

0	0	0	0	0	0	0
0	17	0	0	0	0	11
3	19	0	0	0	0	7
1	33	0	0	1	0	41
0	7	0	0	0	0	11
0	19	0	0	0	0	1
0	13	0	0	0	0	10
6	1	6	56	11	1	0
1	0	0	0	0	1	1
0	0	0	0	0	0	0
0	0	0	0	0	0	0
0	2	0	0	0	0	1
0	3	0	0	0	0	0
0	0	0	0	0	0	8
0	18	0	0	0	0	1
0	0	0	0	0	0	0
0	0	0	0	0	0	0
0	0	0	0	0	0	0
0	0	0	0	0	0	0
0	0	0	0	0	0	0
0	0	0	0	0	0	0
0	0	3	1	7	0	0
3	0	10	0	2	0	1
6	1	2	1	4	0	0
0	0	0	0	0	0	0
0	0	0	0	0	0	0
0	0	0	3	0	0	0
0	0	0	0	0	0	0
6	0	0	0	1	0	0
0	3	0	0	0	0	3
1	1	0	0	0	0	0
0	2	0	0	0	0	9
0	0	0	0	0	0	3
0	0	0	0	0	0	0
0	0	0	1	0	0	0
0	0	0	0	0	0	20
0	0	9	4	0	0	0
0	1	0	0	0	0	0
0	0	0	0	0	0	0
17	1958	3	13	1	42	375
0	12	0	1	0	0	0
0	19	2	1	7	0	1
0	12	0	0	0	0	19
0	1	6	0	0	0	1
0	3	0	0	0	0	0
0	2	0	0	0	0	0
0	0	0	0	0	0	0
0	0	0	0	0	0	0

0	0	0	0	0	0	0
0	0	0	0	0	0	0
0	0	0	0	0	0	0
0	0	0	0	0	0	0
0	0	0	0	0	0	0
2	4	1	3	0	0	1
0	0	0	0	0	0	0
0	0	0	0	0	0	0
0	0	0	0	0	0	0
0	0	0	0	0	0	0
0	0	0	0	0	0	0
0	0	0	0	0	0	0
0	0	0	0	0	0	0
0	0	0	0	0	0	0
0	0	0	0	0	0	0
0	1	0	0	0	0	0
0	0	0	0	0	0	1
13	0	8	33	3	0	0
0	0	0	0	0	0	0
0	1	2	1	2	0	0
0	0	0	0	0	0	0
0	0	0	0	0	0	0
1	3	0	0	0	0	0
1	3	0	0	0	0	0
0	0	0	0	0	0	0
0	7	6	0	3	0	2
3	1	1	4	4	1	4
24	1	51	0	44	0	1
0	0	0	3	0	0	0
0	3	0	0	0	0	0
0	0	0	0	0	0	0
0	0	0	0	0	0	0
0	0	0	0	0	0	0
0	5	0	0	0	0	0
0	20	0	0	0	0	2
0	1	0	0	0	0	14
0	4	28	0	0	0	0
0	8	0	0	0	0	0
0	0	0	0	0	0	0
0	3	0	0	0	0	1
0	0	0	0	0	0	0
0	15	0	0	0	0	0
0	5	0	0	0	0	0
0	0	0	0	0	0	0
37	0	0	1	0	0	0
3	301	0	41	0	0	0
0	0	0	0	0	0	0
0	0	0	0	0	0	0

0	11	0	0	0	0	0
0	0	0	0	0	0	0
3	251	0	2	0	36	83
0	0	0	0	0	1	0
0	8	0	0	0	0	0
0	0	0	0	0	0	0
0	0	0	0	0	0	0
0	0	0	0	0	0	0
0	0	0	0	0	0	0
0	0	0	0	0	0	0
0	5	0	0	0	0	9
0	1	0	0	0	0	0
13	275	0	0	0	2	89
0	0	0	0	0	0	0
0	24	0	0	0	1	16
5	147	0	0	2	0	17
0	14	0	0	0	0	4
0	23	0	0	0	0	3
0	0	0	0	0	0	0
0	1	0	0	0	0	1
0	0	0	0	0	1	0
0	0	0	0	0	0	0
0	0	0	0	0	0	0
0	0	0	0	0	0	0
0	0	0	0	0	0	0
0	0	0	0	0	0	0
0	0	0	0	0	0	0
1	0	33	0	0	0	0
0	0	0	0	0	0	3
0	2	0	0	0	0	0
9	0	0	35	0	0	0
0	28	0	0	0	0	2
2	4	0	1	2	0	0
0	1	0	0	0	0	2
0	0	0	0	0	0	0
0	0	0	0	0	0	0
0	0	0	0	0	0	0
0	0	0	0	0	0	0
0	0	0	0	0	0	0
0	1	0	0	0	0	0
0	0	0	0	0	0	0
0	0	0	0	0	0	0
542	179	36	123	2	459	1770
5	863	0	0	0	1	20
0	4	0	0	0	0	16
0	0	0	0	0	0	0
0	2	0	2	0	4	14
0	0	0	0	0	0	0

0	0	0	0	0	0	0
0	0	0	0	0	0	0
0	0	0	0	2	11	0
0	0	0	0	0	0	0
0	0	0	0	0	0	0
1	0	0	0	0	0	3
1	0	0	0	0	0	0
0	0	0	0	0	0	0
0	0	0	0	0	0	0
0	0	2	0	0	0	0
0	0	0	0	0	0	0
0	0	0	0	0	0	0
0	0	0	0	0	0	0
0	0	0	2	0	0	0
0	0	0	0	0	0	0
0	0	0	0	0	0	0
0	0	0	0	0	0	0
0	0	0	0	0	0	0
0	0	1	3	0	1	0
0	12	0	0	0	0	0
0	0	0	0	0	2	9
0	2	0	0	0	0	0
0	0	0	0	0	0	0
316	669	301	1608	2	1717	1855
0	5	16	69	1	9	10
0	0	0	0	0	0	0
0	0	0	0	0	0	0
2	7	0	1	0	4	3
0	0	0	0	0	0	0
0	0	0	0	0	0	0
0	0	0	0	0	0	0
0	0	0	1	0	0	0
0	0	0	0	0	0	0
0	0	0	1	0	0	0
0	0	0	0	0	0	0
0	0	0	2	0	0	3
24	76	124	459	21	92	91
0	4	4	31	0	12	12
0	0	24	1	1	9	0
0	8	39	2	7	2	5
0	156	0	0	0	0	7
0	0	0	0	0	0	0
0	0	0	0	0	0	0
0	0	0	0	0	0	0
0	0	0	0	0	11	2
0	0	0	0	0	0	0
0	0	0	0	0	0	0
0	0	0	0	0	0	1

0	1	0	0	0	0	3
0	0	0	0	0	0	0
0	0	0	0	0	0	0
0	0	0	0	0	0	0
0	0	0	0	0	0	0
0	0	0	0	0	0	0
0	0	0	0	0	0	0
0	0	0	0	0	0	0
0	0	0	0	0	0	0
0	0	0	0	0	0	0
0	0	0	0	0	0	0
0	6	0	0	0	0	0
0	0	0	0	0	0	0
0	0	0	0	0	0	0
0	0	0	0	0	0	0
0	0	0	0	0	0	0
0	0	0	0	0	0	0
0	0	7	0	3	2	0
0	0	0	0	0	0	0
0	0	2	316	36	30	4
0	0	0	0	0	0	0
5	4	3	3	2	2	3
0	3	0	0	0	0	0
73	415	0	1	0	29	124
0	4	16	0	3	0	0
16	4	0	0	0	1	2
0	0	0	1	2	1	0
0	0	0	0	0	0	10
0	0	0	0	0	1	1
0	0	0	0	0	0	0
0	0	0	0	0	0	0
0	0	0	0	0	0	0
0	17	0	0	0	0	6
0	1	0	0	0	0	0
0	0	0	0	0	0	0
0	0	1	0	0	0	0
0	0	0	0	0	0	0
0	0	0	0	0	0	0
0	3	0	0	0	0	1
0	7	0	0	0	0	0
0	0	0	0	0	0	0
0	0	0	0	0	0	0
0	0	0	0	0	0	0
0	0	0	0	0	0	0
0	0	0	0	0	0	0
0	0	0	0	0	0	0
0	11	0	0	0	0	0
0	0	0	0	0	0	0

0	4	0	0	0	0	0
0	0	0	0	0	0	0
0	0	0	0	0	0	0
12	2	1	70	0	350	83
0	7	0	0	0	0	0
1	0	0	0	0	0	0
0	3	0	0	1	627	71
0	0	0	0	0	0	0
0	1	0	0	0	0	0
0	0	0	0	0	0	0
7	0	0	0	0	1	0
2481	5410	1779	9030	4757	1272	1772
1	22	1	1	0	155	63
6	892	0	0	0	46	207
10	1165	1	5	4	6	74
3	446	7	4	6	94	125
21	177	5	18	6	18	15
4	21	0	0	0	0	0
14	301	8	18	0	41	64
14	439	0	0	0	2	157
0	116	0	0	0	0	35
8	125	0	0	0	35	125
1	12	0	0	0	0	0
0	141	0	0	0	0	52
0	1	0	2	0	0	95
4	0	0	0	0	0	0
0	0	0	0	0	0	0
0	4	0	0	0	0	13
0	0	0	1	0	0	0
1	41	0	0	0	0	20
1	23	0	0	0	0	19
0	0	0	0	0	0	0
1	0	0	0	0	0	0
0	0	0	0	0	0	0
0	0	0	0	0	0	0
0	0	0	0	0	3	8
34	58	19	56	45	46	32
0	0	0	0	0	0	0
0	7	0	0	0	0	1
0	0	0	0	0	0	5
0	5	0	0	0	0	0
0	12	0	0	0	17	10
0	32	0	0	0	8	73
0	1	0	0	0	0	0
0	0	0	0	0	0	0
0	4	0	0	0	0	0
0	49	0	0	0	0	7
0	0	0	0	0	0	0

0	0	0	0	0	0	0
2	12	2	6	2	2	5
0	0	0	0	0	0	0
5	3	2	21	11	1	0
0	6	0	0	0	0	0
0	0	0	0	0	0	0
0	0	0	0	0	0	1
0	1	0	0	0	0	0
0	13	0	0	0	0	6
0	0	0	0	0	0	0
0	0	0	0	0	0	0
0	0	0	0	0	0	0
0	0	0	0	0	0	0
0	0	0	2	0	0	0
0	0	0	0	0	0	4
0	0	0	0	0	0	2
0	0	0	0	0	0	0
0	1	0	0	0	0	1
50	4	17	111	57	9	2
0	0	0	0	0	0	0
0	0	0	0	0	0	0
0	70	0	0	0	5	18
0	0	0	0	0	0	0
0	0	0	0	0	0	0
0	0	0	0	0	0	0
0	0	0	0	1	0	0
0	0	0	0	0	0	0
0	0	0	0	0	0	0
0	3	0	0	0	0	0
0	0	0	0	0	0	0
0	20	0	0	0	9	3
0	1	0	1	1	0	0
0	2	0	3	0	1	1
3	5	0	10	1	9	2
0	4	0	0	0	0	2
1	12	0	0	0	1	2
0	4	2	0	0	0	5
0	0	0	0	0	0	0
0	3	0	0	0	0	1
2	4	0	0	0	0	1
0	10	0	0	0	0	0
0	0	0	0	0	0	0
0	0	0	0	0	0	0
0	0	0	0	0	0	0
0	0	0	0	0	0	0
0	0	0	0	0	0	0
0	0	0	0	0	0	0
0	0	0	0	0	0	0

0	5	0	0	0	1	1
0	2	0	0	0	0	0
0	0	0	0	0	0	0
0	0	0	0	0	0	0
0	0	0	0	0	0	0
0	0	0	0	0	0	0
0	0	0	0	0	0	0
1	0	0	3	0	0	0
0	3	0	0	0	0	0
1	3	0	5	1	2	0
0	0	0	0	0	0	0
0	0	0	0	0	0	0
0	0	0	0	0	0	0
0	1	0	0	0	0	1
0	0	0	0	0	0	0
0	0	0	0	0	0	0
0	0	0	0	0	0	0
0	0	0	0	0	0	0
0	0	0	0	0	1	0
0	1	0	0	0	0	0
0	0	0	0	0	0	0
0	0	0	4	0	0	0
1	1	0	0	0	0	1
0	0	0	0	0	0	0
0	1	0	0	0	0	0
0	0	0	0	0	0	0
0	0	0	0	0	0	0
0	0	0	0	0	0	0
0	0	0	0	0	0	0
0	61	0	0	0	0	2
0	19	1	0	0	0	48
0	40	0	0	0	0	0
0	0	0	0	0	0	0
0	0	0	0	0	0	0
0	0	0	0	0	0	0
1	68	0	0	0	14	80
0	34	0	3	0	0	656
0	7	0	0	0	0	1
1	6	0	0	0	0	12
0	0	0	0	0	0	1
0	0	0	0	0	0	0
0	0	0	0	0	0	0
0	70	0	0	0	3	27
0	1	0	0	0	0	0
0	0	0	0	0	0	0
0	1	0	0	0	0	1
0	0	0	0	0	0	0
0	0	3	129	9	2	0

0	23	0	0	0	1	17
0	8	0	0	0	0	1
0	1	0	0	0	0	8
1	51	0	2	0	0	20
0	12	0	0	0	0	13
0	0	0	0	0	0	0
1333	143	1233	4431	431	37	34
0	39	0	0	0	0	50
1	275	0	3	0	4	204
0	107	0	0	0	4	15
2	63	0	0	0	0	7
0	0	0	0	0	0	5
2	74	0	0	0	0	62
0	0	0	0	0	0	0
0	1	0	0	0	0	0
0	0	0	0	0	0	0
0	0	0	0	0	0	0
0	0	0	0	0	0	0
4	421	2	1	0	0	240
0	0	0	0	0	0	0
0	1	0	0	0	0	0
0	19	0	0	0	0	6
0	1	0	0	0	0	2
0	0	0	0	0	0	0
2	0	0	0	0	0	0
0	0	0	0	0	0	0
7	3	11	27	0	2	1
0	0	0	0	0	0	0
0	0	0	0	0	0	0
1	0	0	0	0	0	0
4	0	0	0	0	0	0
0	0	0	0	0	0	0
0	0	0	0	0	0	1
5	0	0	3	0	0	3
0	0	0	0	0	0	0
0	0	0	0	0	4	0
28	0	0	2372	0	0	0
0	0	0	0	0	0	0
0	0	0	0	0	0	0
0	0	0	0	0	0	0
1	3	0	0	0	1	1
0	0	0	0	0	0	0
0	7	1	0	0	0	6
1	5	0	0	0	0	0
0	4	0	0	0	0	9
0	0	0	0	0	0	0
0	0	0	0	0	0	0
1	0	0	0	0	0	0

0	0	0	0	0	0	0
0	0	0	0	0	0	0
3	7	2	12	0	0	14
0	0	0	0	0	0	0
0	0	0	0	0	0	0
0	0	0	0	0	0	0
0	0	0	0	0	11	6
0	0	0	0	0	0	0
0	0	0	0	0	0	0
0	0	0	0	0	0	0
0	5	0	0	0	0	1
0	0	0	0	0	0	0
9	0	0	9	1	1	0
0	0	0	0	0	0	0
0	0	0	0	0	0	0
0	0	0	0	0	0	0
3	0	0	0	0	0	0
0	0	0	0	0	0	0
0	0	0	0	0	0	0
0	8	1	0	0	2	7
0	0	0	0	0	0	0
151	26	1290	19	34	0	0
0	0	0	0	0	0	0
0	1	0	0	0	0	0
0	20	0	0	0	1	1
0	1	0	0	0	0	3
0	0	0	0	0	0	0
240	0	0	0	0	0	1
7	4	0	10	0	0	1
1	3	3	14	31	0	0
0	0	0	0	0	0	0
2	80	0	0	0	7	3
0	0	0	0	0	0	0
0	0	0	0	0	0	0
0	0	0	0	0	0	0
0	0	0	0	0	0	0
0	0	0	0	0	0	0
0	0	0	0	0	0	0
0	0	0	0	0	0	0
0	0	0	0	0	0	0
27	35	19	232	54	6	19
0	0	1	1	0	0	0
0	0	1	1	0	1	0
449	1496	392	685	1045	90	205
17	80	100	287	5	14	14
206	42	5	4	23	20	180
296	241	126	19	8	28	201
69	80	46	29	65	21	24

0	3	0	0	0	0	4
15	16	11	64	54	10	9
48	5	30	74	9	14	6
10	7	3	20	14	0	0
1	3	0	0	0	0	5
0	71	0	0	0	0	1
1	3	0	0	3	0	0
5	6	39	0	0	0	3
2	1	42	26	10	3	2
51	12	78	12	1	0	0
17	31	22	21	7	0	8
3	83	0	1	0	1	2
2	3	0	1	0	0	1
0	18	0	0	0	0	3
0	6	5	33	0	1	3
6	2	11	18	21	2	1
1	0	0	0	0	0	0
30	40	18	1	2	0	3
0	0	0	0	0	0	0
3	3	2	2	1	1	2
5	2	5	1	1	5	2
6	2	0	0	0	0	0
0	0	0	0	0	0	0
0	0	0	0	0	0	0
0	0	0	0	0	0	0
8	5	7	3	0	0	1
2	0	0	0	0	0	3
0	4	0	0	0	0	4
0	0	0	0	0	0	0
0	0	3	0	0	0	0
0	0	0	0	0	0	0
0	0	0	0	0	0	0
0	0	0	0	0	0	0
0	0	0	2	2	0	0
0	0	0	0	0	0	0
0	0	0	0	0	0	0
0	0	0	0	0	0	0
0	0	0	0	0	0	0
1	0	0	0	0	0	1
0	0	0	0	0	0	0
0	0	0	0	0	0	0
0	1	0	0	0	0	0
0	0	0	0	0	0	0
0	0	0	0	0	0	0
0	0	0	0	0	0	0
0	0	0	0	0	0	0
0	0	0	0	0	0	0
0	0	0	0	0	0	0

2	0	0	0	0	0	1
0	0	0	0	0	0	0
0	0	0	0	0	0	1
0	0	0	0	0	0	0
0	0	0	0	0	0	0
15	0	160	44	7	2	2
0	0	0	0	0	0	0
54	739	5	5	0	72	270
0	3	0	0	0	0	1
0	0	0	0	0	0	0
1	0	0	0	0	0	0
7	0	0	1	0	0	0
1	12	0	0	0	4	0
0	0	0	0	0	0	0
1	1	0	0	0	0	0
211	0	0	0	0	0	0
0	1	0	0	0	0	0
0	0	0	0	0	0	0
0	0	0	0	0	0	0
0	0	0	0	0	0	0
0	0	0	0	0	0	0
0	0	0	5	0	0	0
0	0	0	0	0	0	0
0	0	0	0	0	0	0
0	0	0	0	0	0	0
0	0	0	0	0	0	0
0	0	0	0	0	0	0
0	0	0	0	0	0	0
0	0	0	0	0	0	0
0	0	0	0	0	0	0
8	6	9	7	1	17	3
0	0	0	0	0	0	0
0	0	0	0	0	0	0
0	0	0	0	0	0	0
0	0	0	0	0	0	0
0	10	0	0	0	0	1
159	33	0	8	13	80	7
2	0	0	0	0	0	0
0	0	0	0	0	0	0
0	0	0	0	0	0	0
0	0	0	0	0	0	0
0	0	0	0	0	0	0
0	0	0	0	0	0	0
0	0	0	0	0	0	0
0	0	0	0	0	0	0
0	0	0	0	0	0	0
0	0	3	0	0	0	0
0	0	0	0	0	0	0

0	0	0	0	0	0	0
0	0	0	0	0	0	0
0	0	0	0	0	0	0
0	0	0	0	0	0	0
0	0	0	0	0	0	0
0	0	0	0	0	0	0
28	21	1	15	22	0	0
0	0	0	0	0	0	0
0	0	0	0	0	0	7
0	0	19	1	0	0	0
0	0	0	0	0	0	0
0	0	0	0	0	0	0
12	0	0	4	0	0	1
0	15	0	2	0	0	0
27	6	1	0	79	0	0
0	1	0	0	0	0	3
0	0	0	0	0	0	0
0	0	0	0	0	0	0
0	19	0	0	0	2	8
2	5	4	9	0	2	2
0	0	0	0	0	0	0
0	18	0	0	0	1	2
0	33	0	0	0	0	2
2	0	0	0	2	0	2
0	9	0	0	0	0	0
2	7	0	0	0	2	3
0	7	0	0	1	1	3
0	2	0	0	0	0	7
0	5	3	0	0	0	0
0	0	0	0	0	1	0
0	0	0	0	0	0	0
0	0	0	0	0	0	0
0	0	0	0	0	0	0
0	0	0	0	0	0	0
0	0	0	0	0	0	0
0	0	0	0	0	0	0
0	0	0	0	0	0	0
0	3	0	0	0	0	0
0	0	0	0	0	0	0
1	0	0	0	0	0	0
0	0	0	0	0	0	0
0	0	0	0	0	0	0
0	0	0	0	0	0	0
0	0	0	0	0	0	0
0	0	0	0	0	0	0
0	0	0	0	0	0	0
0	0	0	0	0	0	0
0	0	0	0	0	0	0

0	0	0	0	0	0	0
49	43	69	41	11	47	26
39	23	59	64	13	43	13
21	27	20	42	20	14	6
0	0	0	0	0	6	3
0	0	0	0	0	0	0
7	6	0	0	8	0	0
0	0	1	0	3	2	0
0	0	0	0	0	0	0
10	14	33	14	36	2	6
1	5	0	0	0	3	1
0	0	0	0	0	0	6
0	0	0	0	0	0	0
0	2	0	2	0	0	0
0	7	0	0	0	1	0
0	1	0	0	0	0	1
1	4	0	0	0	0	8
0	6	0	0	0	0	3
0	0	0	0	0	0	0
0	0	0	0	0	0	1
3	0	1	0	0	0	2
0	0	0	0	0	0	1
0	0	0	0	0	0	0
0	0	0	0	0	0	0
0	0	0	0	0	0	0
0	0	0	0	0	0	0
0	0	0	0	0	0	0
0	0	0	7	0	0	0
0	0	0	0	0	0	0
0	0	0	0	0	0	0
0	0	0	0	0	1	0
0	0	0	0	0	0	0
3103	1	0	2	0	1	5
22	38	9	44	1	47	14
12	1	0	0	9	3	13
21	139	0	0	0	0	3
0	3	0	1	0	9	2
0	4	3	0	0	4	0
43	37	0	0	0	5	18
1	99	0	0	0	0	11
11	0	0	3	0	0	1
0	18	1	0	0	0	3
0	0	0	0	0	0	1
2	17	0	0	0	0	10
0	6	0	0	0	0	0
0	0	4	45	18	0	2
0	1	0	0	0	0	0
0	0	0	0	0	0	0

0	2	0	0	0	0	0
0	0	0	0	0	0	0
0	0	0	0	0	0	0
0	0	0	0	0	0	0
0	0	0	0	0	0	0
0	0	0	0	0	0	0
0	0	0	0	0	0	0
0	0	0	0	0	0	0
0	0	0	0	0	0	0
0	0	0	0	0	0	0
0	0	0	0	0	0	0
0	0	0	0	0	0	0
0	0	0	0	0	0	0
0	0	0	0	0	0	0
0	0	0	0	0	0	0
0	0	0	0	0	0	0
0	0	0	0	0	0	0
0	0	0	0	0	0	0
0	1	0	0	0	0	0
0	0	0	0	0	0	0
1	0	0	0	0	0	0
0	0	0	0	0	0	0
0	0	67	0	0	0	0
0	0	0	0	0	0	0
0	0	27	0	0	0	0
0	0	0	0	0	0	0
0	0	0	0	0	0	0
0	2	0	0	0	0	2
0	0	0	0	0	0	0
0	1	0	0	0	0	2
0	0	0	0	0	0	0
0	0	0	0	0	0	0
0	0	0	0	0	0	1
1	0	0	0	0	0	1
0	0	0	0	0	0	0
0	0	0	0	0	0	0
0	0	0	0	0	0	0
0	0	0	0	0	0	0
0	0	0	0	0	0	0
0	0	0	0	0	0	0
0	0	0	0	0	0	0
1	0	0	0	0	2	1
0	0	0	0	0	0	0
1	0	0	0	0	0	0
0	0	0	0	0	0	0
0	0	0	0	0	0	0
0	0	0	0	0	0	0
0	0	0	0	0	0	0

1691	106	31	159	21	80	911
29	73	0	0	1	8	14
0	2	0	0	0	0	0
89	1	3	39	0	0	0
13	0	0	1	0	0	0
0	0	0	0	0	0	0
0	0	0	0	0	0	0
0	0	0	0	0	0	0
0	0	0	0	0	0	0
1	0	0	1	0	0	0
0	0	0	0	0	0	0
0	0	0	0	0	0	0
4	1	0	0	0	2	6
0	0	0	0	0	0	70
42	4	0	0	0	4	1
0	0	0	0	0	0	0
0	0	0	0	0	0	2
0	0	0	0	0	0	0
0	0	0	0	0	0	0
135	2	0	0	0	6	1
294	0	0	2	0	0	1
0	0	0	0	0	0	0
2	0	0	0	0	0	0
24	0	0	0	0	0	0
1310	36	3	20	0	99	25
0	0	0	0	0	0	0
0	0	0	0	0	0	0
4014	13	0	53	23	254	1
0	4	0	0	0	3	4
262	26	169	444	281	56	20
54	2	70	237	95	4	6
0	5	0	0	0	4	10
19	15	28	3	6	5	3
213	1	0	4	0	19	4
142	16	12	141	51	12	1
31	2	22	21	17	3	1
197	0	2	0	2	1	0
0	0	0	0	0	0	0
0	0	0	0	0	0	0
0	0	0	0	0	0	0
0	0	0	0	0	0	0
0	0	0	0	0	0	0
0	0	0	0	0	0	0
2489	1	0	8	2	43	5
4815	18	9	150	22	100	19
0	0	0	0	0	0	1
34	6	33	15	2	13	7
0	0	24	64	0	3	0

9	0	0	0	0	0	0
6	0	0	0	0	1	0
21	0	0	0	1	0	0
24	0	0	0	0	3	0
2	0	0	0	0	0	0
0	0	0	0	0	0	0
0	0	0	0	0	0	0
0	0	0	0	0	0	0
15	0	1	1	7	0	0
0	4	2	2	0	3	264
3	11	0	0	0	0	3
9	0	0	0	0	1	0
0	0	0	0	0	0	1
0	0	0	0	0	0	0
0	0	0	0	0	0	0
6343	35	7	345	10	1037	138
416	0	0	10	0	114	35
42	0	0	1	0	34	3
0	5	0	5	1	2	0
1	0	0	0	0	2	0
6	0	0	0	0	0	0
2	0	0	0	0	3	0
1	0	0	0	0	0	0
0	0	0	0	0	3	0
61	0	0	0	0	2	2
5	0	0	0	0	10	0
10	0	0	0	0	0	0
0	0	0	0	0	0	0
825	2	1	6	0	70	0
84	5	3	5	5	14	4
383	8	2	120	5	24	11
24	17	0	0	0	10	11
33	24	11	129	19	44	18
28	0	4	1	0	0	0
0	0	10	7	0	0	0
0	0	0	1	0	0	0
0	0	0	0	0	0	0
0	0	0	0	0	0	0
48	582	9	6	29	57	276
37	517	2	0	8	9	137
6	96	0	0	0	23	71
2	63	0	0	0	0	115
157	4	0	0	1	1	49
0	19	0	0	0	0	10
0	2	0	1	0	0	0
0	0	0	0	0	1	1
0	0	0	0	0	0	0
0	1	0	0	0	0	1

0	0	0	0	0	0	0
0	0	0	0	0	0	0
0	0	0	0	0	0	0
0	0	0	0	0	0	0
0	1	0	0	0	0	0
0	0	0	0	0	0	0
0	0	0	0	0	0	0
0	0	0	0	0	0	0
0	0	0	0	0	0	0
0	0	0	0	0	0	1
0	0	0	0	0	0	0
0	0	0	0	0	0	0
0	0	0	0	0	0	0
0	0	0	0	0	0	0
0	29	0	0	0	0	6
0	0	1	0	0	0	0
1	12	0	0	0	1	1
180	0	5	9	0	10	1
118	11	0	0	5	10	4
0	4	0	24	0	0	0
17	0	4	9	3	1	0
0	0	0	0	0	0	0
0	0	0	0	0	0	12
0	0	0	0	0	0	0
0	0	0	0	0	0	0
335	16	1	2	0	1	0
1	3	0	0	0	0	5
0	0	1	0	0	0	0
0	0	0	0	0	0	0
0	6	0	0	0	0	0
0	21	0	0	0	0	170
2	2	10	10	0	0	1
0	0	0	0	0	0	0
0	0	0	0	0	0	0
0	0	0	0	0	0	0
0	0	0	0	0	0	0
0	0	0	0	0	0	0
0	0	0	0	0	0	0
0	0	0	2	0	0	0
0	0	0	0	0	0	0
0	0	0	0	0	0	0
0	0	0	0	0	0	0
0	0	0	0	0	0	0
0	2	0	0	1	0	0
7	7	17	15	1	0	0
0	0	0	0	0	0	0
0	0	0	0	0	0	0
0	0	0	0	0	0	0

0	0	0	0	0	0	0
0	0	0	0	0	0	0
720	1957	784	824	1116	460	644
483	0	126	146	50	64	16
104	453	260	1186	754	135	162
2	82	0	0	0	0	51
92	385	86	119	55	101	52
7	311	3	0	1	2	78
0	71	0	0	0	0	3
46	19	33	10	26	34	5
2	69	0	0	0	0	3
0	19	0	0	0	0	6
2	44	0	0	4	14	23
0	152	0	0	0	16	58
51	167	92	56	66	10	40
15	35	32	0	0	111	32
0	25	1	0	0	0	1
8	67	2	0	0	0	10
26	200	52	5	28	18	9
0	28	0	0	0	8	5
0	5	0	0	0	0	2
0	4	0	0	0	0	0
24	32	9	2	1	6	100
1	41	0	0	0	0	28
0	11	2	0	0	0	1
14	39	94	10	12	1	15
22	58	0	2	3	13	26
0	0	0	0	0	0	0
0	6	0	0	0	0	20
0	5	0	0	0	0	9
0	11	0	0	0	1	12
0	0	0	0	0	0	0
0	3	0	0	0	0	0
4	0	0	0	0	0	0
1	46	0	0	0	0	6
0	1	0	0	0	0	0
1	7	0	0	0	0	6
0	0	0	0	0	0	1
0	4	0	0	0	0	1
0	0	0	0	0	0	2
0	0	0	0	0	0	0
0	0	0	0	0	0	0
0	10	14	6	6	0	2
0	0	0	0	0	0	0
0	0	0	0	0	0	0
0	0	0	0	0	0	0
0	6	0	0	0	0	0
0	0	0	0	0	0	0

0	1	0	0	0	0	0
0	0	0	0	0	0	0
2	11	0	0	0	1	2
0	6	0	0	0	1	2
0	2	0	0	0	0	0
0	40	0	0	0	0	0
0	0	0	0	0	0	1
0	0	0	0	0	0	0
0	5	1	0	1	1	2
0	0	0	0	0	0	0
0	3	0	0	0	0	0
0	0	0	0	0	0	0
0	0	0	0	0	0	0
0	1	0	0	0	0	2
0	0	0	0	0	0	0
0	0	0	0	0	0	0
0	11	0	0	0	0	1
0	0	0	0	0	0	0
0	0	0	0	0	0	0
0	0	0	0	0	0	0
0	0	0	0	0	0	0
0	0	0	0	0	0	0
0	0	0	0	0	0	0
0	0	0	0	0	0	0
0	0	0	0	0	0	0
0	0	0	0	0	0	0
0	0	0	0	0	0	0
0	0	0	0	0	0	0
0	0	0	0	0	0	0
0	0	0	0	0	0	0
0	0	0	0	0	0	0
0	0	0	0	0	0	0
0	0	0	0	0	0	0
0	0	0	0	0	0	0
0	3	0	0	0	0	1
0	0	0	0	0	0	0
0	0	0	0	0	0	0
0	0	0	0	0	0	0
0	3	0	0	0	0	0
0	0	0	0	0	0	0
0	0	0	0	0	2	1
0	0	0	0	0	0	0
0	0	0	0	0	0	0
0	0	0	0	0	0	0
14	49	13	55	7	9	66
3	4	0	0	0	0	5
0	0	0	0	0	0	0
0	5	0	0	0	0	11

0	0	0	0	0	1	0
0	0	0	0	0	0	0
3	0	0	0	0	0	0
0	32	0	0	1	5	18
0	0	0	3	1	0	0
0	2	0	0	0	0	0
11	0	0	0	0	0	0
0	0	0	0	0	0	0
6	186	11	10	152	18	76
714	1767	279	702	238	1209	925
0	25	0	0	0	4	2
68	1	37	37	30	26	1
143	183	68	143	119	156	92
0	4	2	2	1	8	5
0	2	0	0	0	0	0
3	9	0	2	0	4	4
24	67	72	15	14	24	14
2	4	1	7	7	10	2
8	0	3	14	4	8	0
0	12	0	0	0	14	3
3	12	0	27	6	3	3
0	8	0	0	0	0	7
0	9	0	0	6	3	3
0	0	0	0	0	0	0
0	6	0	0	0	0	0
1	0	1	0	0	1	0
0	0	0	0	0	0	2
2	11	0	4	2	6	7
0	1	0	0	0	1	0
0	0	0	0	0	0	0
0	0	0	0	0	0	1
1	0	0	0	0	0	0
2	1	0	0	0	2	0
1	3	0	0	0	0	2
0	0	0	0	0	0	0
0	0	0	0	0	0	0
1	1	0	0	0	0	0
1	6	0	1	0	4	1
0	0	0	0	0	0	0
0	0	0	0	0	0	0
0	0	0	0	0	0	0
0	0	0	0	0	0	0
0	14	0	0	0	0	0
0	0	0	0	0	0	0
0	1	0	0	0	0	0
0	0	15	0	0	0	0
0	0	0	0	0	0	0
0	0	0	0	0	0	0

0	0	1	10	2	0	0
1	4	0	0	0	5	2
0	0	0	0	0	0	0
0	0	0	0	0	0	0
0	0	0	0	0	0	0
0	1	0	0	1	0	2
0	0	0	0	0	0	0
3	18	2	2	0	5	3
0	0	0	0	0	0	0
0	0	0	0	0	0	0
0	0	0	0	0	0	0
0	1	0	0	0	1	1
0	0	0	0	0	0	0
0	0	0	0	0	0	0
0	0	0	0	0	0	0
1	1	1	0	0	2	2
0	3	0	0	0	0	0
0	0	0	0	0	0	0
0	0	0	0	0	0	0
0	0	0	0	0	0	2
0	0	0	0	0	0	0
0	0	1	0	0	0	0
0	1	0	0	0	0	1
0	1	0	0	0	0	0
0	0	0	0	0	0	0
0	0	0	0	0	0	0
0	0	0	0	1	0	0
0	0	0	0	0	0	0
0	0	0	0	0	0	0
0	0	0	0	0	0	0
0	0	0	0	0	0	0
0	0	0	0	0	0	0
0	0	0	0	0	0	0
0	0	0	0	0	0	0
0	0	0	0	0	0	0
0	0	0	0	0	0	0
0	0	0	0	0	0	0
0	0	0	0	0	0	0
0	0	0	0	0	0	0
0	0	0	0	0	0	0
0	0	0	0	0	0	0
0	1	0	0	0	0	0
0	0	0	0	0	1	0
0	0	0	0	0	0	0
0	0	0	0	0	0	0
0	0	0	0	0	0	0
0	0	0	0	0	0	0
0	0	0	0	0	0	0

0	0	0	0	0	1	0
350	313	122	263	134	340	116
27	148	14	42	9	30	84
36	20	27	103	11	79	26
57	38	41	30	64	57	8
171	30	16	45	20	29	10
15	20	3	22	11	15	5
0	4	0	1	0	7	1
6	2	0	0	1	13	3
15	76	0	8	4	22	60
0	15	0	0	0	4	10
0	18	0	0	0	4	2
2	1	0	0	0	0	0
0	0	0	0	0	0	2
6	2	2	3	3	0	1
0	0	0	0	0	0	0
8	8	0	3	0	12	2
0	0	0	0	0	5	12
0	0	0	1	0	0	0
0	2	0	0	1	1	1
0	4	0	1	0	0	0
2	0	0	1	1	1	1
0	1	0	1	0	0	0
0	0	0	0	0	0	0
0	0	0	0	0	0	0
1	0	0	3	0	0	2
0	0	0	0	0	0	0
0	0	0	0	0	0	1
0	1	0	0	0	0	0
0	0	0	0	0	0	0
0	0	0	0	0	0	0
3	0	0	0	0	0	0
0	0	0	0	0	0	0
0	0	0	0	0	0	0
0	0	0	0	0	1	1
0	0	0	0	0	0	0
551	79	353	209	286	125	25
0	20	2	0	0	17	17
72	0	0	32	11	55	3
6	0	0	86	42	17	0
27	1	2	5	2	13	1
0	6	0	0	0	0	0
19	0	2	15	10	1	0
0	6	0	0	0	4	1
0	0	0	0	0	0	0
0	0	0	0	1	0	0
1	0	0	0	0	0	0
0	0	0	0	0	0	0

0	0	0	0	0	0	0
0	0	0	0	0	0	0
0	0	0	0	0	1	1
0	0	0	0	0	0	0
0	0	0	0	0	0	0
0	0	0	0	0	0	0
0	0	0	0	0	0	0
0	0	0	0	0	0	0
0	0	0	0	0	0	0
0	0	0	0	0	0	0
1	0	0	1	0	0	0
0	0	0	0	0	0	0
0	0	0	0	0	0	0
0	0	0	0	0	0	0
0	0	0	0	0	0	0
0	0	0	0	0	0	0
0	0	0	0	0	0	0
13	33	1	7	4	2	12
1	0	1	0	0	3	1
0	0	0	0	0	0	0
0	2	0	0	0	0	1
0	3	11	0	0	1	1
4	9	0	0	0	0	0
0	0	0	0	0	0	0
0	0	0	0	0	0	0
0	0	0	0	0	0	0
0	11	0	0	0	0	0
0	0	0	0	0	0	0
0	0	0	374	0	0	0
15	5	0	1	1	0	0
0	1	0	0	0	0	0
0	0	0	0	0	0	0
0	0	0	0	0	0	0
84	36	38	152	176	59	12
1	1	0	0	0	1	0
0	0	2	0	0	0	0
0	0	0	0	0	0	0
0	0	0	0	0	0	1
0	0	0	0	0	0	0
0	0	0	0	0	0	0
0	0	0	0	0	0	0
0	0	0	0	0	0	0
0	0	0	0	0	7	1
1	0	0	0	0	0	0
3	11	4	2	0	30	8
0	1	0	0	0	0	0
0	0	0	1	0	0	0
2	16	0	2	1	9	6

0	0	0	0	0	0	0
110	62	206	318	61	65	27
4	8	4	0	2	9	9
0	0	0	0	0	0	0
0	0	0	0	0	0	1
0	0	0	0	0	0	0
0	0	0	0	0	0	0
0	0	0	0	0	0	0
0	0	0	0	0	0	0
36	97	107	170	109	45	19
50	20	49	41	36	42	20
0	2	0	0	0	0	0
0	2	0	0	0	0	0
0	0	0	0	0	0	0
0	0	0	0	0	0	0
0	5	0	0	0	0	0
0	3	0	0	0	0	0
0	0	0	0	0	0	0
0	1	0	0	0	0	0
0	6	0	0	0	0	0
0	0	0	0	0	0	0
0	0	0	0	0	0	0
0	0	0	0	0	0	0
0	0	0	0	0	0	0
0	0	0	0	0	0	0
0	0	0	1	0	0	0
0	0	0	0	0	0	0
0	0	0	0	0	0	0
0	0	0	0	0	0	0
0	0	0	0	0	0	0
0	0	0	0	0	0	0
0	2	0	0	0	0	2
0	0	0	0	0	0	0
0	0	0	0	0	0	0
10	2	0	0	0	0	10
0	0	0	1	0	0	2
2	0	0	0	0	0	0
0	0	0	1	1	0	0
0	3	0	0	0	0	0
0	0	0	0	0	0	0
0	7	2	0	0	3	21
0	3	0	0	0	0	3
0	0	0	0	0	0	0
0	0	0	0	0	0	0
1	0	0	0	0	1	0
0	0	0	0	0	0	0
4	0	0	0	0	0	0
0	0	0	0	0	0	0

0	9	0	0	0	0	20
0	0	0	0	0	0	0
0	0	0	0	0	0	0
2	0	0	0	0	0	18
0	3	0	1	0	0	0
0	0	0	0	0	0	0
1	66	0	0	0	4	35
0	0	0	0	0	0	0
0	18	0	0	0	0	4
0	0	0	0	0	0	0
0	0	0	0	0	0	0
0	0	0	0	0	0	0
0	2	0	0	0	0	1
0	0	0	0	0	0	0
9	190	3	1	0	1	806
0	0	0	0	0	0	0
0	0	0	0	0	0	0
25	0	0	0	0	0	0
0	0	0	0	0	0	0
0	0	0	0	0	0	0
0	0	0	0	0	0	0
0	1	0	0	0	5	0
0	1	0	0	0	0	3
0	0	0	0	0	0	0
0	0	0	0	0	0	1
0	0	0	0	0	0	0
0	0	0	0	0	0	0

64.SUR	64.DCM	65.SUR	65.DCM	66.SUR	66.DCM	67.SUR
0	0	0	0	0	0	0
0	0	0	0	0	0	0
0	0	0	0	0	0	0
0	0	0	0	0	0	0
0	0	0	0	0	0	0
0	0	0	0	0	0	0
0	0	0	0	0	0	0
0	14	4	137	0	0	0
12	20	6	20	0	0	0
0	0	0	0	0	0	0
0	0	0	0	0	0	0
0	0	0	0	0	0	0
0	0	0	0	0	0	0
0	0	0	53	0	0	0
0	0	0	0	0	0	0
0	1	0	0	0	0	0
0	11	5	17	1	1	0
407	200	583	1957	239	189	4
0	0	0	0	0	1	0
0	0	0	0	0	0	0
0	4	1	0	0	0	0
0	0	0	0	0	0	0
0	0	0	0	0	0	0
0	0	0	0	0	0	0
0	0	0	0	0	0	0
0	0	0	0	0	0	0
0	0	0	0	0	0	0
0	0	0	0	0	0	0
0	0	0	0	0	0	0
0	0	1	1	0	0	0
0	0	1	0	0	0	0
0	0	0	0	0	0	0
0	0	0	21	0	0	0
6	0	36	15	0	0	0
0	0	0	0	0	0	0
0	0	0	0	0	0	0
84	39	396	59	24	62	0
74	126	208	709	11	26	0
65	12	2	25	0	0	4
0	0	0	1	0	0	0
10	31	16	52	33	58	0
0	0	0	0	39	35	0
3	11	41	146	0	0	0
668	7	88	142	2	0	0
1	2	15	48	21	72	0
0	0	1	0	0	0	0
3	2	1	22	25	8	24

4	1	12	15	0	0	0
3	2	40	38	0	0	0
0	0	0	4	0	0	0
0	4	17	28	0	0	0
0	3	0	5	0	0	0
0	0	0	0	0	0	0
0	0	0	0	0	0	0
7	0	5	2	0	0	0
0	0	0	0	0	0	0
0	0	0	0	0	0	0
3	7	0	2	62	40	0
0	0	0	0	0	0	0
0	0	0	0	0	0	0
0	0	0	0	1	0	0
0	0	0	2	0	0	0
0	0	0	0	0	0	0
0	0	0	0	0	0	0
0	0	0	0	0	0	0
0	0	0	0	0	0	0
0	0	0	0	0	0	0
0	0	0	0	0	0	0
0	0	0	0	0	0	0
0	0	0	0	0	0	0
0	0	0	0	0	0	0
0	0	0	0	0	0	0
0	0	0	0	0	0	0
1	0	0	1	0	0	0
0	0	0	0	0	0	0
0	0	0	0	0	0	0
2	1	0	16	0	0	0
5	2	1	10	0	0	0
4	1	14	9	1	0	0
0	0	0	5	0	0	0
0	0	0	0	0	0	0
0	0	0	0	0	0	0
0	0	0	0	0	0	0
0	0	0	0	0	0	0
0	0	0	0	0	0	0
0	0	0	0	0	0	0
1044	1354	1802	3884	4	41	0
89	84	222	263	617	117	190
24	16	542	916	9	2	2
0	1	1	27	1	1	2
0	0	1	7	0	0	0
0	0	0	0	0	0	0
0	0	0	0	0	0	0
0	0	0	0	0	0	0
0	0	0	1	0	0	0

0	0	0	0	0	0	0
0	0	0	0	0	0	0
0	0	0	0	0	0	0
0	0	2	15	0	0	0
0	0	0	0	0	0	0
1	0	6	79	21779	3970	504
0	0	0	0	105	12	9
0	0	0	0	0	0	0
0	0	0	0	0	0	0
0	0	0	0	2	0	0
0	0	0	0	0	0	0
0	0	0	0	0	0	0
0	0	0	0	4	0	0
0	0	0	0	0	0	0
0	0	0	0	0	0	0
0	0	0	0	0	0	0
0	0	0	7	1	0	0
0	0	0	0	0	0	0
0	0	0	0	0	0	0
241	24	13	49	0	0	0
0	0	0	0	0	0	0
0	0	0	0	0	0	0
0	1	1	8	3	0	0
0	3	1	2	0	9	0
0	0	0	0	0	0	0
0	0	0	0	12	0	0
1	0	9	10	0	0	0
0	0	0	0	24	0	0
8	0	0	22	0	0	0
0	0	0	0	3	4	0
0	0	0	0	0	0	0
0	0	0	0	0	0	0
0	3	0	0	0	0	0
0	0	0	0	0	0	0
1	0	0	0	1	0	0
2	3	2	1	1	0	0
0	0	0	0	0	0	0
0	0	0	0	0	0	0
0	0	0	0	0	0	0
0	0	1	0	0	3	0
11	0	0	0	74	5	0
0	0	0	0	0	0	0
0	0	0	0	0	0	0
0	0	0	0	0	0	0
181	6	0	0	9	0	0
0	0	0	0	0	0	0
0	0	0	0	0	0	0
0	0	0	0	1	0	0

0	0	3	0	0	0	0
0	0	0	0	0	0	0
4	9	16	15	35	46	0
0	5	0	0	0	0	0
2	1	0	4	0	0	0
0	0	0	0	0	0	0
0	0	0	0	0	0	0
0	0	0	0	0	0	0
0	0	0	0	0	0	0
0	0	40	154	0	0	0
0	0	0	0	1	0	0
0	0	0	0	0	0	0
199	52	174	643	1	44	0
25	0	0	0	0	28	3667
1	8	17	3	7	10	0
0	0	1	20	0	0	0
1	0	0	0	0	0	0
0	0	0	1	0	0	0
0	0	0	0	1	0	19
0	0	0	2	1	1	0
1	1	1	0	0	7	0
0	0	0	0	0	0	0
6	0	0	3	0	0	0
0	0	0	0	0	0	0
0	0	0	0	0	1	0
0	0	0	0	0	0	0
0	0	0	0	0	0	0
0	0	0	0	0	0	0
0	0	0	0	0	0	0
0	0	0	0	0	0	0
0	0	0	0	0	0	0
0	0	0	0	0	0	0
0	0	0	0	0	0	0
0	0	0	0	0	0	0
0	0	0	0	0	0	0
0	0	0	0	0	0	0
0	0	0	0	0	0	0
0	0	0	0	0	0	0
0	0	0	0	0	0	0
2	0	11	0	5	0	1
0	0	0	0	0	0	0
0	0	0	0	4	0	0
2	34	106	213	2	25	0
20	7	12	16	2	0	0
13	1	9	0	95	0	0
0	0	0	0	0	0	0
7	3	32	9	104	12	0
7	0	1	0	11	0	5

0	0	1	0	0	0	0
0	0	1	0	13	0	4
1	0	0	0	16	0	3
0	0	0	1	0	0	0
0	0	0	0	105	0	0
5	2	0	0	0	0	0
0	0	0	0	0	0	0
0	0	0	0	23	0	16
0	0	0	0	0	0	0
0	0	0	0	0	0	0
0	0	0	0	0	0	0
0	0	0	0	0	0	8
0	0	0	0	0	0	0
0	0	1	3	0	0	0
0	0	0	0	0	0	0
0	0	0	0	0	0	0
0	0	0	0	0	0	0
0	3	1	0	0	0	0
0	0	0	1	0	0	0
174	0	4	0	0	0	4
10	0	9	6	0	26	0
0	0	0	0	0	0	0
0	0	0	0	0	0	0
162	134	768	157	4	125	0
11	27	27	0	95	5	0
0	0	0	0	0	0	0
0	0	0	0	0	0	0
0	1	0	0	0	0	0
1	0	0	0	0	0	0
0	0	0	0	0	0	0
0	0	0	0	0	0	0
0	0	1	0	0	0	0
0	0	0	0	0	0	0
0	0	0	0	0	0	0
0	0	0	0	0	0	0
94	0	0	0	0	0	6458
54	42	63	150	0	0	1
7	3	37	54	0	0	5
0	0	0	0	0	0	37
0	3	9	14	5	0	0
0	0	0	0	0	0	0
0	0	6	0	50	0	0
0	0	0	0	0	0	0
0	0	0	0	0	0	0
0	0	0	0	0	0	0
0	0	0	0	1	0	0
0	0	0	0	4	0	0
0	0	0	0	0	0	0

0	2	0	0	0	0	0
0	0	0	0	0	0	0
0	0	0	1	0	0	0
3	0	7	0	0	0	0
0	0	0	0	0	0	0
0	0	0	0	0	0	0
0	0	0	0	0	0	0
0	0	0	0	1	0	0
0	1	1	0	0	0	0
0	0	0	0	0	0	0
0	0	0	0	0	0	0
0	0	0	0	0	0	0
0	0	0	0	0	0	3
0	0	0	0	0	0	0
0	0	0	0	0	0	0
0	0	0	0	0	0	0
1	0	0	0	0	0	4
0	0	0	0	0	0	0
0	0	0	0	0	0	0
0	0	0	0	0	0	0
0	0	0	0	0	0	0
13	0	0	306	0	1	2
0	0	0	0	0	0	0
39	195	208	661	495	360	0
1	0	4	0	0	0	0
0	3	3	6	0	2	0
1	2	4	2	0	1	0
0	0	0	0	0	0	0
0	0	0	4	0	0	0
0	0	0	0	0	0	0
0	0	0	0	0	0	4
0	0	0	0	0	0	0
0	0	0	1	0	0	0
0	0	0	0	0	0	0
0	0	0	2	1	0	0
0	0	0	0	0	0	0
0	0	0	0	0	0	0
0	0	0	2	0	0	0
0	0	0	0	0	0	0
0	0	0	1	0	0	0
0	0	0	0	0	0	0
0	0	0	0	0	0	0
0	0	0	2	0	0	0
0	0	0	0	0	0	0
0	0	0	0	0	0	0
0	0	0	0	0	0	0
0	0	0	0	0	0	0
0	1	0	0	0	0	0

0	0	0	0	0	0	0
0	0	0	11	0	0	0
0	0	0	0	0	0	0
176	319	135	2	0	0	0
0	0	0	0	0	0	0
0	1	0	0	0	0	0
900	265	575	53	3	0	0
0	0	0	0	0	0	0
0	0	0	0	0	0	0
0	0	0	0	0	0	0
0	0	0	0	0	0	0
3239	2359	3255	9790	5894	4605	16
83	95	128	387	80	98	0
253	198	305	786	95	95	2
63	93	162	605	13	7	0
203	133	135	314	870	548	0
57	21	51	101	238	223	0
226	90	36	537	4	0	0
15	6	0	8	0	0	0
4	7	15	72	7	1	0
0	81	40	18	0	0	0
13	17	7	218	0	0	0
1	3	0	2	46	47	0
21	59	41	125	0	0	0
0	3	3	34	0	0	0
0	10	0	0	0	0	0
0	0	0	0	0	0	0
0	0	1	0	0	0	0
0	0	0	0	0	0	0
0	1	2	8	1	5	0
0	1	9	26	0	0	0
0	0	0	0	98	90	0
0	0	0	0	0	0	0
0	0	0	0	30	17	3
0	0	0	0	0	0	0
4	6	1	7	0	0	0
38	27	56	115	89	95	0
0	0	0	0	0	0	0
0	0	0	0	0	1	0
0	1	1	14	15	6	0
0	0	0	8	0	0	0
2	0	3	0	0	0	0
0	1	1	20	0	0	0
0	0	0	0	0	0	0
0	0	0	0	0	0	0
0	0	1	57	8	0	0
0	0	1	19	0	0	0
0	0	0	0	0	0	0

0	5	0	0	0	0	0
1	0	1	8	1	1	0
0	1	2	0	0	0	0
0	0	0	0	0	0	0
0	1	0	0	0	0	0
0	0	0	0	55	0	1
0	2	1	8	1	0	0
3	5	2	23	0	0	0
0	0	7	0	4	0	0
0	0	0	0	0	1	0
0	1	0	0	0	0	0
0	0	0	0	0	0	0
0	0	0	1	6	3	0
0	0	0	0	0	0	0
0	0	0	0	4	0	0
0	0	0	0	0	0	0
0	0	0	0	0	0	0
0	0	1	3	1	0	0
0	0	0	14	0	0	0
0	0	0	0	0	0	0
0	0	0	0	0	0	0
4	3	1	11	3	1	0
0	0	0	0	0	0	0
0	0	0	0	0	0	0
0	0	0	0	0	0	0
0	3	2	1	1	0	0
0	0	0	0	0	0	0
0	0	0	0	0	0	0
0	0	0	0	0	0	0
0	1	0	0	0	0	0
18	9	13	8	0	0	0
1	0	2	5	3	2	0
7	6	8	22	2	0	0
2	1	5	6	3	5	0
0	1	0	0	0	0	0
0	2	0	1	0	0	0
1	1	0	1	2	2	0
0	0	0	0	0	0	0
0	0	0	0	0	0	0
1	0	0	1	0	0	0
0	0	2	0	3	1	0
0	0	0	0	0	0	0
0	0	0	0	0	0	0
0	0	0	0	3	2	0
0	0	0	0	0	0	0
0	0	0	0	0	0	0
0	0	0	0	0	0	0
0	0	0	0	0	0	0

0	0	0	1	2	0	0
0	0	0	1	0	0	0
0	0	0	0	0	0	0
0	0	0	3	0	0	0
1	1	0	0	0	2	0
0	0	0	3	2	0	0
0	0	0	0	0	0	0
0	1	0	1	0	1	0
0	0	0	1	0	0	0
3	2	2	3	3	2	0
0	0	0	0	0	0	0
0	0	0	2	0	0	0
0	0	0	0	0	0	0
0	0	0	0	0	0	0
0	0	0	0	0	0	0
0	0	0	0	0	0	0
0	0	0	0	0	1	0
0	1	0	0	2	1	0
1	0	0	0	0	0	0
0	0	0	0	0	0	0
0	0	0	0	0	0	0
0	0	0	0	0	0	0
0	0	0	0	0	0	0
0	0	0	0	0	0	0
0	0	0	1	0	1	0
0	0	0	1	0	0	0
0	0	0	0	0	0	0
0	1	0	0	0	0	0
0	0	0	0	0	1	0
10	39	97	107	0	0	0
0	3	15	180	7	4	0
0	0	0	0	1	1	0
0	0	0	0	0	0	0
0	0	0	0	0	0	0
0	0	0	0	0	0	0
4	52	38	184	0	0	0
2	36	21	37	0	0	0
0	14	20	1	0	0	0
0	1	0	8	1	0	0
0	0	0	0	6	0	0
0	0	0	0	0	0	0
0	0	0	0	0	0	0
5	3	3	2	0	0	0
0	1	0	0	0	0	0
0	3	0	11	0	0	0
3	0	0	0	0	0	0
0	0	0	0	0	0	0
306	3	0	0	0	0	0

0	3	2	30	17	8	0
0	1	0	0	0	0	0
0	0	0	3	0	0	0
2	6	16	30	0	0	0
0	0	0	0	0	0	0
0	0	0	0	0	0	0
426	44	73	354	2	0	4
0	2	0	16	26	18	0
1	29	83	208	35	35	2
2	3	5	15	1	0	0
0	0	0	7	1	0	0
2	20	31	50	2	7	0
0	0	2	0	0	0	0
0	0	0	0	0	0	0
0	0	0	0	0	0	0
0	0	0	0	0	0	0
0	1	0	0	0	0	0
0	0	0	0	2	0	0
101	119	150	904	0	2	0
0	0	0	7	0	0	0
0	0	40	0	0	0	0
0	1	0	3	0	0	0
0	0	0	0	0	0	0
0	0	0	0	0	0	0
0	0	0	0	0	0	0
0	0	0	0	0	0	0
1	2	0	1	0	1	0
0	0	0	0	0	0	0
0	0	0	0	0	0	0
2	0	0	0	0	0	0
0	0	0	0	0	0	0
0	0	0	0	0	0	0
0	0	0	0	0	0	0
0	4	0	0	0	0	0
0	0	1	0	16	0	0
0	0	0	0	0	0	0
0	0	0	0	0	0	0
0	0	0	0	0	0	0
0	0	0	0	0	0	0
0	0	0	0	0	0	0
3	0	8	4	0	0	0
0	0	0	0	0	0	0
0	1	1	20	13	3	0
0	1	6	0	11	5	0
0	0	0	0	0	0	0
0	0	0	0	0	0	0
0	0	0	0	0	0	0
0	0	0	0	0	0	0

0	1	1	0	9	0	1
0	0	0	0	0	0	0
12	7	7	70	4	13	25
0	0	0	0	0	0	0
0	0	0	0	0	0	0
0	0	0	0	0	0	0
0	0	0	0	0	0	0
0	0	0	0	0	0	0
0	0	0	0	0	0	0
0	0	0	0	3	0	0
0	0	0	0	1	0	0
0	0	0	0	0	0	0
0	0	0	0	0	0	0
0	0	0	0	1	0	0
0	0	0	0	0	0	0
0	0	0	0	0	0	0
0	0	0	0	0	0	0
0	0	0	0	0	0	0
2	0	0	3	0	0	0
8	13	32	19	0	0	0
0	0	0	0	0	0	0
0	7	0	0	1	0	0
0	0	0	0	0	0	0
0	0	1	0	12	11	9
9	4	0	4	9	5	0
0	1	0	0	0	0	0
0	0	0	0	0	0	0
0	15	0	0	0	26	0
0	0	0	0	0	0	0
3	0	1	2	0	0	0
0	0	0	0	0	0	0
16	6	2	19	0	0	0
0	0	0	0	0	0	0
0	0	0	0	0	0	0
0	0	0	0	0	0	0
0	0	0	0	0	0	0
0	0	0	0	1	0	0
0	0	0	0	0	0	0
0	0	0	0	0	0	0
0	0	0	0	0	0	0
189	76	131	298	58	31	2
0	0	0	0	0	0	0
1	0	0	1	0	0	0
2870	463	1246	1818	555	550	1
332	18	200	262	2	49	0
771	922	557	533	47	9	3
3598	682	192	6	1	41	3
2487	9	51	5	4	8	0

0	1	0	2	1	0	0
150	8	20	7	13	16	0
47	15	63	127	19	23	0
53	301	43	115	0	0	0
112	7	40	22	0	0	0
87	0	1	0	2	0	0
0	0	26	84	1	2	0
0	0	0	0	0	0	0
9	2	0	1	4	2	0
2	0	6	0	0	0	0
5	0	24	7	0	0	0
4	1	0	4	0	0	0
0	2	0	1	0	0	2
2	0	0	4	0	0	0
0	0	0	0	0	0	0
0	0	0	0	0	5	0
0	0	1	0	8	2	0
4	14	2	11	0	0	0
0	0	0	0	0	0	0
3	2	0	5	2	8	0
0	0	0	0	0	0	0
1	7	0	10	0	1	0
0	0	0	0	0	0	0
0	0	0	0	29	10	36
0	0	0	0	0	0	0
0	0	1	0	3	1	0
0	0	0	0	1	0	0
0	0	0	0	0	0	0
0	0	0	0	0	0	0
0	0	0	0	0	0	0
0	0	0	0	0	0	0
0	0	0	0	0	0	0
2	0	0	0	0	0	0
0	1	0	0	2	1	0
0	0	0	0	0	0	0
45	1	0	0	0	0	0
0	0	0	0	0	0	0
0	2	0	0	1	0	0
0	0	0	0	4	10	0
0	0	0	0	0	0	0
0	0	0	0	0	0	0
2	0	0	0	0	2	0
0	0	0	0	0	0	0
0	0	0	0	0	0	0
0	0	0	0	1	0	0
0	0	0	0	0	0	0
0	0	0	0	0	0	0
0	0	0	0	0	0	0

1	1	1	0	0	1	0
0	0	1	0	0	0	0
0	0	0	0	0	0	0
0	0	0	0	0	0	0
0	0	0	0	0	0	0
18	2	8	13	5	9	0
0	0	0	0	0	0	0
147	240	165	556	380	344	3
1	0	3	1	3	4	0
0	0	0	0	1	0	0
0	0	0	0	0	0	0
0	0	7	0	0	0	0
0	0	17	0	0	0	0
0	0	0	0	0	0	0
0	0	8	0	60	8	1
0	16	0	0	15	0	0
1	0	0	0	0	0	0
0	0	0	0	0	0	0
0	0	0	0	0	0	0
0	0	0	0	0	0	0
0	0	0	0	0	0	0
0	0	1	0	0	0	0
0	0	1	0	0	0	0
0	0	0	0	0	0	0
0	0	7	0	0	0	0
0	0	0	0	0	0	0
0	0	0	0	0	0	0
0	0	0	0	0	0	0
0	0	0	0	0	0	0
0	1	1	4	21	36	0
17	7	5	26	8	0	0
0	0	0	0	38	14	0
0	0	0	0	257	25	6
0	0	0	0	0	0	118
0	0	0	0	0	0	0
4	49	6	0	0	0	0
118	43	41	0	13	1	3
1	0	0	1	133	26	298
0	0	0	0	412	97	0
0	0	0	0	0	0	0
0	0	0	0	0	0	0
0	0	0	0	0	0	0
23	0	0	0	0	0	3211
4	0	0	0	638	84	667
0	0	0	0	2	0	0
0	0	3	0	0	0	0
0	0	0	0	0	0	0
0	2	0	0	0	0	0

0	0	0	0	23	2	0
0	0	0	0	0	0	0
0	0	0	0	0	0	15
0	0	0	0	0	0	0
0	0	0	0	0	0	0
0	0	0	0	0	0	0
5	6	3	76	0	0	0
0	0	0	0	0	0	0
0	0	0	0	0	0	0
0	0	0	0	0	0	0
0	0	0	0	0	0	0
0	0	0	0	0	0	0
0	2	1	277	0	12	12
0	0	0	0	247	108	17
24	1	6	1	1	14	0
76	8	0	29	2	2	0
0	0	2	0	0	0	0
0	0	0	0	0	0	0
40	11	2	22	49	27	0
1	1	1	1	0	0	0
8	0	5	39	10	2	270
106	18	10	54	0	0	0
2	0	0	4	7	1	0
60	12	4	27	3	1	0
8	0	0	3	7	3	0
12	12	10	5	0	0	0
21	3	2	21	4	0	0
37	1	0	8	2	2	0
0	0	1	2	0	0	0
1	0	0	0	9	4	0
0	0	0	0	0	0	0
1	0	0	0	0	0	0
0	0	0	0	0	0	0
0	0	0	0	1	0	0
0	0	0	0	0	0	0
0	0	0	1	0	0	0
0	0	0	0	0	0	0
0	0	0	2	0	0	0
0	0	0	0	0	0	0
0	0	2	5	0	1	0
0	0	0	0	0	0	0
0	0	0	0	0	0	0
0	0	0	0	0	0	0
0	0	0	0	0	0	0
0	0	0	0	0	0	0
0	0	0	0	0	0	0
0	0	0	0	0	0	0
0	0	0	0	0	0	0

0	0	0	0	0	0	0
273	51	13	139	63	42	0
141	22	30	156	9	1	0
24	7	7	31	5	1	0
8	0	0	4	4	5	11
0	0	0	0	0	0	0
9	0	0	1	72	49	0
0	0	0	0	0	0	0
0	0	0	0	0	0	0
65	7	1	25	7	0	0
4	3	0	32	0	0	0
0	0	0	1	0	0	0
0	0	0	0	0	0	0
0	0	0	0	0	0	0
0	0	0	5	0	0	0
0	0	0	2	0	1	0
0	0	0	1	0	1	0
0	0	0	0	55	24	0
0	0	0	0	0	1	0
1	0	0	0	0	0	0
7	4	0	1	22	8	0
0	1	3	3	0	0	0
4	3	0	0	1	0	0
0	0	0	0	0	0	0
0	0	0	0	0	0	0
0	0	0	0	0	0	0
0	0	0	0	0	0	0
0	0	0	0	0	0	0
0	0	0	0	0	0	0
0	0	0	1	0	0	0
8	0	0	3	0	0	0
0	0	0	0	0	0	0
43	259	15	88	155	23	0
101	21	9	243	699	278	3885
407	135	304	76	2189	253	0
83	37	11	171	6	59	0
9	0	1	71	0	0	0
2	0	6	24	0	0	0
33	31	31	34	2	0	0
33	31	29	49	20	13	0
13	7	2	0	0	0	0
56	11	6	54	32	18	0
0	2	3	2	0	0	0
10	8	4	124	53	59	0
0	0	3	26	6	1	0
0	2	0	0	0	8	0
0	0	0	8	0	1	0
3	0	0	1	0	0	0

0	0	0	0	0	0	0
0	0	0	0	0	0	0
0	0	0	0	0	0	0
0	0	0	0	0	0	0
0	0	0	0	0	0	0
0	0	0	0	0	0	0
0	0	0	0	0	0	0
0	0	0	0	0	0	0
0	0	0	0	0	0	0
0	1	0	0	0	0	0
0	1	0	0	0	0	0
1	1	0	1	1	0	0
0	0	0	0	0	0	48
0	0	0	0	0	0	0
0	0	0	0	0	0	0
0	0	0	0	0	0	0
0	0	0	0	0	0	0
0	0	0	0	0	0	0
0	0	0	0	0	0	0
0	0	0	0	0	0	0
0	0	0	0	0	0	0
0	0	0	0	0	0	0
0	0	0	0	0	0	0
0	0	0	0	0	0	0
0	0	0	0	0	0	0
0	0	0	0	29	0	0
0	0	0	1	0	0	0
0	0	0	0	0	0	0
0	0	0	0	0	0	0
21	18	34	2	26	0	1
5	3	9	0	13	0	0
1	1	2	0	1	0	0
1	2	5	0	3	0	0
0	1	7	0	0	0	0
0	0	1	0	0	0	0
0	1	0	0	1	0	0
0	0	0	0	0	0	0
0	0	1	0	1	0	0
2	0	1	0	0	0	0
1	0	1	0	2	0	0
0	0	0	0	321	97	0
29	30	12	0	7	0	0
1	0	0	0	0	0	248
4	1	0	0	1	0	0
0	0	0	0	0	0	72
0	0	0	0	0	0	0
0	0	0	0	0	0	0
0	0	0	0	0	1	0

2109	1916	3226	121	8267	88	1130
54	72	39	9	38	3	1
0	0	0	0	3	4	0
0	0	0	0	0	0	0
46	24	9	3	6	0	0
0	0	0	0	22	0	1
0	0	0	0	0	0	0
0	0	1	0	0	0	0
0	0	0	0	0	0	0
0	0	0	0	0	0	0
0	0	0	0	0	0	0
0	0	0	0	0	0	0
78	27	161	4	223	0	0
16	23	44	0	0	0	0
773	106	41	3	3	0	1
0	0	0	0	0	0	21
0	0	0	0	0	0	0
0	0	0	0	0	0	0
0	0	0	0	0	0	0
87	16	2	0	706	4	0
7	0	0	0	7	0	0
0	0	0	0	0	0	0
2	1	0	0	11	0	0
2	0	0	0	0	0	0
35	30	12	76	53	18	6
0	0	0	0	0	0	0
0	0	0	0	0	0	0
323	72	23	6	45	14	0
31	12	12	24	99	68	0
489	35	5	9	141	43	2
164	15	15	26	68	23	0
1	2	37	0	1613	11	0
66	10	2	2	53	11	0
3	4	0	0	0	0	0
35	3	5	5	0	0	0
8	0	0	0	2	0	0
1	0	0	0	22	0	0
0	1	0	5	52	21	0
0	0	0	0	0	0	0
0	0	0	0	0	0	0
0	0	0	0	0	0	0
0	0	0	0	0	0	0
0	0	0	0	0	0	0
30	11	15	0	15	23	0
263	93	18	0	547	5	0
19	5	0	3	69	23	0
8	7	0	1	0	0	0
0	0	0	0	0	0	0

0	0	0	0	0	0	0
0	0	0	0	0	0	0
0	0	0	0	0	0	0
3	1	0	0	0	0	0
0	0	0	0	0	0	0
0	0	0	0	0	0	0
0	1	0	0	0	0	0
2	0	0	0	0	0	0
0	0	0	0	0	6	0
59	66	190	2	19	0	0
1	2	0	0	0	0	0
2	3	3	0	0	0	0
0	0	0	0	0	0	0
0	0	0	0	0	0	0
0	0	0	0	0	0	0
1433	713	101	19	694	18	3
305	221	100	0	564	11	0
2	0	0	0	1	0	0
2	0	0	0	0	0	0
0	0	0	0	0	0	0
4	4	0	0	6	0	0
0	2	0	0	0	0	0
0	0	0	0	0	0	0
0	0	0	0	0	0	0
39	10	1	0	21	0	0
0	0	0	0	0	0	0
4	1	0	1	3	0	0
0	0	0	0	0	0	0
7	8	3	0	3	0	0
0	3	1	0	39	10	0
24	33	5	0	0	0	0
141	167	47	3	52	0	0
51	12	1	18	22	8	1
0	0	0	0	0	0	0
0	0	0	0	0	0	0
0	0	0	0	0	0	0
0	1	0	0	0	0	0
0	0	0	0	0	0	0
1916	426	252	220	11736	340	23931
61	29	103	0	20	5	21
87	95	263	3	511	30	0
15	11	16	36	5	0	0
82	71	73	1	9	0	0
0	0	0	3	0	0	0
0	0	0	0	14	1	0
0	0	0	1	0	0	0
0	0	1	0	0	0	0
2	0	7	0	1	0	0

0	0	0	0	0	0	6
0	0	0	0	0	0	0
0	0	0	0	0	0	0
0	0	0	1	0	0	0
1	0	0	0	0	0	0
0	0	0	0	0	0	0
0	0	0	0	0	0	0
0	0	0	0	0	0	0
0	0	0	0	0	0	0
3	0	0	0	3	1	1
0	0	0	0	0	0	0
0	0	21	0	0	0	1
0	0	0	0	0	0	0
0	0	0	0	0	0	0
45	18	0	2	25	1	0
9	1	0	0	17	15	0
49	17	21	40	4	5	0
0	0	0	0	2	0	0
0	0	0	0	0	0	0
0	0	0	0	0	0	1
0	0	0	2	0	0	0
0	0	0	0	0	0	0
0	0	0	0	0	0	0
0	0	0	0	0	0	0
0	0	0	0	0	0	0
207	129	43	16	1082	16	0
110	67	29	0	116	2	0
0	0	8	0	5	0	0
0	0	0	0	0	0	1
0	0	0	1	1	0	0
1	1	1	6	36	16	0
2	0	0	0	1	0	0
0	0	0	0	0	0	0
0	2	0	0	0	0	0
0	0	0	0	0	0	0
0	0	0	0	0	0	0
0	0	0	0	0	0	0
0	0	0	0	0	0	0
0	0	0	0	0	0	0
0	0	0	0	0	0	0
0	0	0	0	0	0	1
0	0	0	0	0	0	0
0	0	3	0	45	15	2
29	7	3	25	1	0	0
5	0	0	0	0	0	0
0	0	0	0	0	0	0
0	0	0	0	0	0	0
0	0	0	0	0	0	0

0	0	0	0	0	0	0
0	0	0	0	0	0	0
1729	445	346	1495	1062	923	1929
70	9	7	235	1141	219	0
558	363	360	596	640	376	0
56	21	78	354	35	120	0
352	292	98	386	298	196	129
437	83	259	712	935	207	0
349	5	11	67	28	59	0
26	3	10	68	10	6	0
40	24	36	76	0	0	0
3	0	17	72	0	0	0
11	16	10	100	37	31	0
35	31	28	59	0	19	0
150	150	103	186	174	109	0
86	36	107	206	118	77	0
129	22	21	138	2	3	0
4	10	77	41	180	88	0
138	68	40	46	2	2	0
7	2	24	30	12	4	0
37	2	29	51	430	193	0
0	0	0	0	99	57	173
18	43	14	42	32	16	0
34	14	5	19	5	8	1
4	8	2	12	2	0	0
12	19	18	14	11	15	0
25	8	12	11	2	0	0
0	3	0	0	0	0	0
0	0	0	5	0	0	0
11	13	3	14	0	0	0
6	12	8	18	6	1	0
94	0	9	11	2	0	0
1	0	0	0	5	0	0
4	1	26	27	9	6	0
1	3	7	22	2	2	0
1	0	9	30	6	12	0
0	0	0	1	0	0	0
5	5	4	0	0	0	0
0	0	0	5	0	0	0
0	0	0	0	0	0	0
0	0	0	0	0	0	0
0	0	0	0	0	0	0
0	0	0	0	0	0	0
1	0	0	10	0	0	0
1	1	1	0	0	1	0
0	2	0	0	1	0	0
0	0	0	0	0	0	0
0	0	0	0	0	0	0

7	0	0	2	0	0	0
0	0	0	0	0	0	0
0	0	0	1	1	0	0
0	0	0	0	0	0	0
0	0	0	0	0	0	0
0	1	0	0	0	0	0
0	0	0	0	0	0	0
0	1	0	0	0	0	0
2	1	0	5	2	1	1
0	0	0	0	0	0	0
0	0	0	0	0	0	0
0	0	0	0	0	0	0
0	0	0	1	0	0	0
0	0	0	0	0	0	0
0	0	0	0	0	0	0
0	0	0	0	0	0	0
0	0	0	0	0	0	0
0	1	0	0	0	0	0
0	0	0	0	0	0	0
0	0	0	0	0	0	0
0	0	0	0	0	0	0
0	0	0	0	0	0	0
0	0	0	0	0	0	0
0	0	0	0	0	0	0
0	0	0	0	0	0	0
0	0	0	0	0	0	0
0	0	6	0	1	0	0
0	0	0	0	0	0	0
0	0	0	0	0	0	0
0	0	0	0	0	0	0
0	0	0	0	0	0	0
0	0	0	0	0	0	0
0	0	0	0	0	0	0
0	0	0	0	0	0	0
0	0	0	0	0	0	0
0	0	0	0	0	0	0
0	0	0	0	0	0	0
0	0	0	0	0	0	0
0	0	0	0	0	0	0
0	0	0	0	0	0	0
1	0	1	1	0	0	0
0	0	0	1	0	0	0
0	0	0	0	0	0	0
0	0	0	0	0	0	0
33	18	10	75	342	129	2
155	21	3	61	18	8	1
0	0	0	0	0	0	0
0	0	0	0	0	0	0

0	0	0	0	0	0	0
0	0	0	0	0	0	0
0	0	0	0	0	0	0
38	26	10	168	31	12	1
0	0	0	0	0	0	0
10	5	3	7	0	0	0
0	0	0	0	0	0	0
0	0	0	0	0	0	0
448	89	31	754	1933	628	170
2262	613	229	3246	3841	1505	2554
24	7	10	122	277	81	4026
17	2	0	0	581	134	0
232	61	41	269	651	282	3
38	12	5	87	145	45	1506
0	1	0	0	0	1	0
13	5	1	10	13	4	14
15	7	0	1	0	0	0
25	3	0	14	33	4	0
16	1	1	20	0	0	0
123	12	0	41	34	8	0
2	1	1	2	47	24	0
7	12	10	124	38	15	0
18	10	1	12	0	0	1
0	0	0	0	0	0	0
0	0	0	0	3	2	0
2	0	1	6	3	2	0
3	1	0	5	4	1	0
10	3	0	69	21	7	3
2	0	0	2	4	2	0
0	0	0	0	0	0	77
1	1	0	0	0	4	0
0	0	0	0	0	0	0
4	1	0	6	10	1	3
0	0	0	1	2	3	1
0	0	0	0	0	0	0
0	0	0	0	0	0	0
0	1	0	1	1	2	0
0	0	0	0	0	0	0
0	0	0	0	3	0	0
0	0	0	0	0	0	2
0	0	0	3	0	0	0
0	0	0	0	0	0	0
0	0	0	5	0	0	0
0	0	0	0	0	0	0
0	1	0	1	0	0	0
0	0	0	0	0	0	0
0	0	0	0	0	0	3
0	0	0	0	0	0	0

0	0	0	0	0	0	0
2	2	1	3	10	0	0
0	0	0	0	0	0	0
0	0	0	0	0	0	0
0	0	0	0	0	0	0
0	0	0	4	1	1	0
0	0	0	0	0	0	0
11	2	1	24	25	7	1
0	0	0	0	0	0	0
1	0	0	0	1	0	0
2	0	0	0	0	0	2
0	0	0	1	2	0	3
0	0	0	0	0	0	0
1	0	0	2	0	0	1
0	0	0	0	0	0	0
0	0	0	2	5	1	2
0	0	0	3	0	0	0
0	0	0	0	0	0	0
1	0	0	2	1	0	0
0	0	0	0	0	2	0
0	0	0	0	0	0	0
0	0	0	0	0	0	0
1	0	0	2	3	1	0
0	1	0	0	0	0	0
0	0	0	0	1	0	2
0	0	0	0	0	0	0
0	0	0	0	0	0	0
0	0	0	0	0	0	0
0	0	0	0	0	0	0
0	0	0	1	1	0	1
0	0	0	0	0	0	0
0	0	0	0	0	0	0
0	0	0	0	0	0	0
0	0	0	0	0	0	0
0	0	0	0	0	0	0
0	0	0	0	0	0	0
0	0	0	0	0	0	0
0	0	0	0	0	0	0
0	0	0	1	1	0	0
0	0	0	0	0	0	0
0	0	0	0	0	0	0
0	0	0	0	0	0	0
1	0	1	0	3	1	0
0	0	0	0	0	0	0
0	0	0	0	0	0	0
0	0	0	0	0	0	0
0	0	0	0	0	0	0
0	0	0	0	0	1	7

0	0	0	0	0	0	0
397	93	42	602	378	187	0
49	22	11	83	82	28	8
49	12	2	34	48	14	0
32	4	3	2	0	0	0
18	11	5	43	1	4	0
16	8	3	32	6	3	0
25	2	10	26	15	8	0
9	1	1	10	0	0	0
71	19	6	163	27	8	0
6	2	2	20	7	2	0
6	6	1	10	2	3	0
0	0	0	0	0	0	5
0	0	0	0	0	0	0
1	1	0	2	0	1	0
0	0	0	0	40	21	0
16	6	4	41	5	3	0
1	0	1	1	0	1	0
0	0	0	1	0	0	0
1	1	0	1	0	0	0
2	2	0	2	0	1	0
2	0	0	3	1	0	1
0	0	0	4	5	2	1
0	0	0	0	0	0	0
0	0	0	0	0	0	0
1	0	0	1	1	0	0
0	0	0	0	0	0	0
0	0	0	0	1	0	0
0	0	0	0	0	0	0
5	0	0	1	0	1	0
1	0	0	0	0	0	0
0	0	0	0	0	0	0
0	0	0	0	0	0	0
1	0	0	0	0	0	0
0	0	0	0	0	0	0
0	0	0	0	0	0	0
412	34	41	644	154	71	2
18	12	27	129	4	0	1
49	3	2	10	0	0	0
0	0	0	0	0	0	0
2	3	0	35	0	0	0
0	0	0	1	0	0	0
0	0	0	2	0	0	0
1	0	3	48	0	0	0
0	0	0	0	0	0	0
0	1	0	0	0	0	0
0	0	0	0	0	0	0
0	0	0	0	8	1	0

0	0	0	2	1	0	0
0	0	0	0	0	0	0
0	0	0	0	23	3	0
0	0	0	0	0	0	0
0	0	0	0	0	0	0
0	0	0	0	0	0	0
0	0	0	0	0	0	0
0	0	0	0	0	0	0
0	0	0	0	0	0	0
0	0	0	0	0	0	0
0	0	0	1	0	0	0
0	0	0	0	0	0	0
0	0	0	0	0	0	0
0	0	0	1	8	0	0
0	0	0	0	0	0	0
0	0	0	0	0	0	0
0	0	0	0	0	0	0
4	3	11	31	2	0	0
0	0	0	0	0	0	0
0	0	0	0	0	0	0
0	0	0	2	0	0	0
0	0	2	0	0	0	0
0	0	0	0	0	0	0
0	0	3	0	0	0	0
0	0	0	0	0	0	0
0	0	0	0	0	0	0
0	0	0	0	0	0	0
0	0	0	0	0	0	0
0	0	0	1	192	50	29
0	0	0	1	1	0	0
25	0	0	0	0	0	0
0	0	0	0	0	0	0
0	0	0	0	0	0	0
213	19	10	39	40	24	1
3	1	0	0	0	0	0
3	1	0	0	19	14	0
1	0	0	0	3	0	0
142	10	5	9	0	0	0
0	0	0	0	0	0	0
0	0	0	0	0	0	14
0	0	0	0	1	0	0
0	0	0	0	0	0	0
53	0	0	4	203	99	0
0	0	0	0	0	0	0
20	0	0	3	18	12	0
0	0	0	0	0	0	0
0	0	0	0	0	0	0
35	22	11	152	55	11	0

0	0	0	0	0	0	0
294	31	28	192	16	20	0
72	7	12	40	52	16	0
0	0	0	0	11	2	0
1	0	0	0	0	2	0
1	1	0	0	0	4	0
0	0	0	0	0	0	0
0	0	0	0	0	0	0
0	0	0	0	0	0	0
145	58	47	242	166	67	1
119	21	11	74	26	13	1
35	31	100	0	8	0	0
0	0	0	1	0	0	0
0	0	0	0	7	3	0
0	0	0	0	0	0	0
0	0	0	0	0	0	0
0	0	1	5	0	0	0
0	0	0	0	0	0	0
0	0	0	0	3	0	0
0	0	0	6	0	0	0
0	0	0	0	0	0	0
0	0	0	0	0	0	0
0	0	0	0	8	3	0
0	0	0	0	0	0	0
0	0	0	0	0	0	0
0	0	0	0	0	0	0
0	0	0	0	0	0	0
0	0	0	0	0	0	0
0	0	0	0	0	0	0
0	0	6	0	0	0	0
0	0	0	0	0	0	0
19	1	2	1	0	0	0
0	0	0	0	0	0	0
0	0	0	0	0	0	0
4	10	1	0	11	32	0
0	0	0	0	0	9	0
0	0	0	0	0	0	0
12	0	0	0	0	0	0
0	0	0	0	0	0	0
0	0	0	0	0	0	0
9	20	11	38	0	0	0
7	3	2	4	0	0	0
0	0	0	0	0	0	0
0	0	0	0	0	0	0
0	0	0	0	0	0	0
0	0	0	0	2	0	0
0	0	0	0	0	0	0
0	0	0	0	0	0	0

0	1	0	0	0	0	0
0	0	0	0	0	0	0
0	0	0	0	0	0	0
1	1	0	0	0	0	0
0	0	0	0	0	0	1
0	0	0	0	0	0	0
84	127	171	17	16	5	0
0	0	0	0	2	0	0
0	0	0	2	2	0	0
0	0	0	0	0	0	0
0	1	0	0	0	0	0
0	0	0	0	0	0	0
0	0	0	0	0	0	0
0	0	0	0	0	0	0
74	224	67	509	12	8	0
0	0	0	0	0	0	0
0	0	0	0	0	0	0
0	0	0	0	0	0	0
0	0	0	0	0	0	0
0	0	0	0	0	0	0
0	0	0	0	0	0	0
0	0	0	0	0	0	0
1	3	0	1	0	0	0
0	0	0	0	0	0	0
0	1	0	0	0	0	0
1	0	0	0	0	0	0
0	0	0	0	0	0	0

68.SUR	68.DCM	70.SUR	72.SUR	72.DCM	76.SUR	76.DCM
0	0	0	0	0	0	0
0	0	0	0	1	0	0
0	0	0	0	0	0	0
0	0	0	0	0	0	2
0	0	0	0	0	1	0
0	0	0	0	0	0	0
0	0	0	0	0	0	0
0	1	0	0	37	1	2
0	0	1	0	6	0	2
0	0	0	0	0	0	0
1	0	0	0	0	0	0
0	0	0	0	0	0	0
0	0	0	0	0	0	0
0	0	0	0	0	0	0
0	0	0	0	0	0	1
0	0	0	0	0	0	0
2	1	2	11	2	5	156
63	424	133	272	259	415	110
0	5	1	1	2	6	0
0	0	0	6	0	2	23
0	0	0	2	9	7	16
0	0	0	0	0	0	0
0	0	0	0	0	1	3
0	3	0	0	0	0	0
0	0	0	0	0	0	0
0	0	0	0	0	0	0
0	0	0	0	0	0	0
0	0	0	0	0	4	0
0	0	0	0	0	0	0
0	0	0	0	2	0	1
0	0	0	0	0	0	0
0	0	0	0	0	0	0
0	17	2	0	37	0	4
0	0	0	0	3	0	0
0	0	0	0	4	0	0
0	0	0	0	0	0	0
5	11	0	1	1	7	0
22	8	2	648	45	220	56
181	41	0	5340	209	2223	98
0	0	0	1	0	0	7
4	10	12	0	39	3	341
4	61	4	0	6	3	0
2	0	4	19	24	0	1
1	6	15	1	11	0	127
3	69	29	4	26	0	22
0	0	0	0	32	0	224
1	31	4	0	21	0	26

0	11	3	0	17	0	7
0	1	4	1	16	0	15
0	0	2	1	4	0	0
0	0	0	0	63	0	15
0	0	5	0	10	1	9
0	1	0	0	0	0	0
0	0	0	0	5	0	1
0	0	0	4	1	0	0
0	0	0	0	0	2	0
0	0	0	0	0	0	12
3	20	7	3	0	0	0
0	0	0	0	0	0	30
0	0	1	0	0	0	0
2	6	1	5	2	1	2
0	0	0	0	3	0	2
0	0	0	0	0	0	0
0	0	0	0	0	0	0
3	0	0	0	0	0	0
0	0	0	0	0	0	0
0	0	0	0	0	0	0
0	0	0	0	0	0	0
0	0	0	0	0	0	0
0	0	0	0	0	0	0
0	0	0	0	0	0	0
0	1	0	0	0	0	0
0	0	0	0	0	0	0
0	0	0	0	0	0	0
0	0	0	0	0	0	0
0	0	0	0	0	0	0
0	0	0	0	15	0	5
0	0	0	0	0	0	10
1	0	0	4	0	3	0
0	0	0	0	0	0	0
0	0	0	0	0	0	0
0	0	0	3	1	0	0
0	0	0	0	0	0	0
0	0	2	0	0	0	0
0	0	0	0	0	0	7
0	0	0	0	0	0	0
5	14	23	5	1104	67	2633
1049	70	85	0	0	1	6
15	8	1	1	7	12	34
0	0	0	0	92	0	259
0	0	4	0	0	0	0
0	0	0	1	0	0	7
0	0	0	0	0	0	27
0	0	0	0	0	0	0
1	0	0	0	0	0	0

0	0	0	0	0	0	0
0	0	0	0	0	0	0
0	0	0	0	0	0	0
0	0	1	0	0	0	0
0	0	0	0	0	0	0
3	11	36	0	0	1	1
0	0	0	0	0	0	0
0	0	0	0	0	0	0
0	0	0	0	0	0	0
0	0	0	0	0	0	0
0	0	0	1	0	0	0
0	0	0	0	0	0	0
0	0	0	0	0	0	0
0	0	0	0	0	0	0
0	0	0	0	0	0	0
0	0	0	0	0	0	0
0	1	0	0	0	1	1
0	0	0	0	0	1	0
0	0	0	0	0	0	0
3	0	2	0	0	2	0
0	0	0	0	0	0	0
0	0	0	0	0	0	0
0	0	2	0	15	1	7
1	7	1	0	6	0	22
0	0	0	0	0	0	0
3	0	0	14	6	61	0
0	2	0	0	0	0	0
0	0	0	8	13	1	15
1	0	0	11	0	0	0
0	1	0	1	0	0	1
0	0	0	0	0	0	0
0	0	0	0	0	0	0
0	0	0	1	0	0	0
0	0	0	0	6	0	19
0	6	1	0	11	0	9
0	2	0	0	48	0	18
7	2	0	1	0	0	0
0	0	0	0	0	0	0
0	0	0	0	0	0	0
0	1	0	0	2	0	8
16	0	0	0	0	0	0
0	0	0	0	0	0	1
0	0	0	0	0	0	4
0	0	0	0	0	0	0
22	0	0	0	0	1	0
0	0	0	0	0	0	0
0	0	0	0	1	0	0
0	0	0	0	0	0	0

0	1	0	0	1	0	10
0	0	0	0	0	0	0
63	131	25	140	107	1	5
0	0	0	0	0	0	0
0	0	0	0	0	0	1
0	0	0	0	0	0	0
0	0	0	0	0	0	0
0	0	0	0	0	0	0
0	0	0	0	0	0	2
0	0	0	0	0	0	0
0	0	0	0	6	0	0
0	0	0	0	3	0	11
43	77	50	36	64	7	182
3	0	0	0	0	0	0
0	73	3	0	13	1	4
0	0	0	0	8	0	13
0	0	3	0	6	1	15
0	5	0	0	9	1	8
0	0	0	0	0	0	0
0	1	0	1	1	0	0
3	5	11	0	0	5	2
0	0	0	0	3	0	0
0	0	0	0	0	0	0
0	0	0	0	0	0	0
0	0	2	0	0	0	0
0	0	0	0	0	0	0
0	0	0	0	0	0	0
0	0	0	0	0	0	0
0	0	0	0	0	0	0
0	0	0	0	1	0	0
0	0	0	0	0	0	0
0	1	0	1	23	1	210
0	0	0	0	0	0	0
0	0	0	0	0	0	0
0	0	0	0	0	0	2
0	0	0	0	12	0	0
0	0	0	0	0	0	0
0	0	0	0	0	0	0
0	0	0	27	14	0	0
26	19	0	0	6	0	0
0	0	0	0	0	0	0
0	0	0	0	0	0	0
17	0	17	13	3	8	1
533	28	59	20	67	0	42
806	8	43	4	750	5	8
0	0	0	0	0	0	0
577	17	260	4	436	2	17
103	0	10	0	4	0	5

6	2	1	0	0	0	0
0	0	0	0	0	0	0
0	0	0	0	0	0	0
0	2	0	0	0	3	0
0	1	0	0	0	1	0
0	0	0	0	0	0	0
0	1	0	0	0	0	0
13	0	0	0	0	0	0
0	0	0	0	0	0	0
0	0	2	0	3	0	0
0	0	0	0	0	0	0
0	0	0	0	0	0	0
0	0	0	0	0	0	0
0	0	5	0	6	0	0
10	1	0	0	0	0	0
0	0	0	0	0	0	0
5	0	0	0	0	0	0
0	0	0	0	0	0	2
0	0	0	0	3	1	1
0	0	26	0	665	0	10
8	6	15	0	8	0	7
0	0	0	0	0	0	1
0	0	0	0	0	0	0
206	28	7529	133	11350	13	79
22	19	32	15	8	2	2
0	0	0	0	0	0	0
0	0	0	0	0	0	0
1	0	0	0	0	0	0
0	1	0	0	0	0	0
0	0	1	0	1	0	0
0	0	0	0	0	0	0
0	0	0	0	2	0	0
0	0	0	0	0	0	0
0	0	0	0	1	0	0
0	0	1	0	0	0	0
2	0	0	0	5	0	0
31	47	312	14	1138	25	592
0	1	89	5	466	4	16
1	0	14	0	0	0	0
0	0	2	39	5	1	1
1	0	0	0	7	0	0
326	4	0	0	0	3	0
0	0	0	0	0	0	0
0	0	0	0	0	0	0
0	0	0	0	0	0	0
16	1	35	1	57	10	0
122	1	1	0	0	0	0
70	7	0	0	1	0	4

15	1	1	0	6	0	0
1	0	0	0	0	0	0
0	0	0	0	0	0	0
0	0	0	0	0	0	0
0	0	0	0	0	0	0
0	0	0	0	0	0	0
0	0	0	0	0	0	0
0	0	0	0	2	0	0
0	0	2	0	0	0	1
29	0	0	0	2	0	0
0	0	0	0	0	0	0
0	0	0	0	1	0	0
0	0	0	0	0	0	0
7	0	0	0	0	0	0
0	0	0	0	0	0	0
0	0	0	0	0	0	0
0	0	0	0	1	0	0
0	0	0	0	0	0	0
0	0	0	0	0	0	0
0	0	0	0	0	0	0
0	0	0	0	0	0	0
1	0	0	7	4	9	1
0	0	0	0	0	0	0
19	412	85	67	971	107	578
0	0	0	0	0	0	0
0	8	2	1	5	5	5
3	6	15	4	1	1	0
0	0	0	0	0	0	0
0	0	0	0	1	0	0
0	1	0	0	0	0	0
0	0	0	0	0	0	0
0	0	0	0	0	0	0
0	0	0	0	3	1	1
0	0	0	0	3	1	3
0	0	0	0	1	0	0
0	0	0	0	0	0	0
0	0	0	0	0	0	0
0	0	0	0	0	0	11
0	0	0	0	0	0	0
0	0	1	2	4	0	0
0	0	0	0	0	0	5
0	0	0	0	0	0	0
0	0	0	0	0	0	0
0	0	0	0	0	0	0
0	0	0	0	2	0	0
0	0	0	0	0	0	0
0	0	0	0	0	0	1
1	0	0	0	0	0	0

0	0	0	0	0	0	0
0	0	0	0	0	0	1
3	0	0	3	0	0	0
5	0	2	48	8	14	3
0	0	0	0	0	1	0
0	0	0	0	0	0	0
109	1	630	202	452	91	48
0	0	0	0	0	0	0
0	0	0	0	0	0	0
0	0	0	0	0	0	0
0	0	0	0	0	0	0
4019	7599	14610	6768	6718	2017	2957
259	461	526	72	160	2	68
17	35	76	68	673	11	147
4	5	9	41	461	4	33
128	181	352	30	205	17	113
18	38	48	279	170	73	115
0	23	24	0	5	4	17
19	20	5	26	110	75	17
0	0	2	0	294	5	111
0	0	0	0	1	7	73
0	0	0	0	36	4	109
5	1	0	1	0	1	0
0	0	0	0	41	0	39
0	149	0	1	31	0	52
0	0	0	1	0	0	0
0	0	0	0	0	0	0
0	0	0	0	27	0	12
0	0	0	1	10	1	62
0	2	0	1	16	0	8
0	0	0	0	28	1	49
2	10	0	0	0	0	0
0	0	0	0	0	0	0
1	0	2	0	3	0	4
0	0	0	0	0	0	0
0	0	15	74	2	26	9
69	107	442	212	91	39	77
0	2	0	3	36	0	0
0	0	0	1	7	0	0
1	1	35	0	0	1	0
0	0	0	0	1	0	5
0	0	0	0	12	0	1
5	83	3	0	64	1	19
0	1	0	0	1	0	120
0	0	0	0	0	0	0
0	0	0	0	14	3	6
0	0	0	0	10	0	14
0	0	0	0	0	0	18

0	0	0	0	0	0	1
1	4	9	3	38	3	42
0	0	0	0	12	0	63
0	0	0	2	0	8	0
0	0	0	0	16	0	2
0	0	59	0	0	0	0
0	27	5	0	32	0	4
0	0	15	0	31	0	5
0	0	1	0	14	1	1
0	1	0	0	0	0	5
0	0	0	0	0	0	0
0	0	0	0	0	0	0
6	8	0	0	0	0	0
1	0	0	1	0	1	1
0	0	3	0	12	0	7
0	0	1	0	0	0	26
0	0	0	0	0	0	0
0	0	0	0	4	0	3
1	0	0	41	0	22	0
1	0	0	1	0	0	0
0	0	0	0	0	0	0
4	1	1	6	17	7	4
0	0	0	0	0	0	0
0	0	0	3	0	0	0
0	0	0	0	0	0	0
0	1	4	0	0	0	0
0	0	0	0	2	0	13
0	0	0	0	0	0	0
0	0	0	0	0	0	0
0	0	0	0	0	0	0
1	1	1	0	5	1	15
1	7	29	1	1	1	1
1	0	4	2	5	1	5
2	3	19	11	4	3	3
0	0	0	0	11	0	12
0	1	0	1	3	0	1
0	0	3	2	11	0	1
0	0	1	0	3	0	3
0	0	0	0	3	0	1
0	1	0	0	0	0	0
0	0	0	0	0	0	4
0	0	0	0	0	0	0
0	0	0	0	0	0	0
0	1	0	0	0	0	1
0	0	0	0	0	0	1
0	0	0	0	0	0	0
0	0	0	0	0	0	0
0	0	0	0	0	0	1

0	0	1	2	3	3	1
0	2	6	0	0	0	0
0	0	0	0	0	0	0
0	2	3	0	0	0	0
4	3	8	0	0	0	1
0	3	0	0	0	0	1
0	0	0	0	0	0	0
2	1	2	1	2	2	0
0	0	0	0	0	0	0
1	3	11	7	9	1	1
0	0	0	0	0	0	0
0	0	0	0	0	0	0
0	0	0	0	0	0	0
0	0	0	0	4	0	3
0	0	0	0	0	0	0
0	0	0	0	0	0	0
0	0	0	3	0	0	0
0	0	1	0	0	0	0
0	0	0	1	0	0	0
0	0	0	0	0	0	0
0	0	0	0	0	0	1
0	0	0	0	0	0	0
0	0	1	1	0	0	0
0	0	0	0	0	0	0
0	0	3	0	0	0	0
0	0	0	0	0	0	0
0	0	0	0	0	0	0
0	0	0	0	0	0	0
0	0	0	0	0	0	0
1	18	4	0	7	2	13
0	0	0	0	0	0	1
0	0	0	0	2	0	9
0	0	0	0	0	0	0
0	0	0	0	0	0	0
0	0	0	0	0	0	0
2	61	64	0	80	1	22
2	327	0	0	42	1	923
0	0	0	0	3	2	495
0	1	7	0	16	0	153
0	0	0	0	30	0	20
0	0	0	0	0	0	0
0	1	0	0	0	0	5
2	0	6	0	14	19	87
0	0	0	0	0	0	0
0	0	0	0	7	0	0
0	0	7	0	2	0	0
0	0	0	0	0	0	0
0	0	0	1	0	0	0

7	27	5	0	30	0	228
0	2	2	2	49	0	17
0	0	0	0	54	0	26
0	7	0	0	9	0	12
0	0	0	0	15	0	4
0	1	0	0	0	0	3
10	0	31	1811	176	46	6
19	94	26	2	58	3	14
5	11	33	4	182	2	237
0	10	20	1	14	1	112
0	8	2	0	34	1	105
0	0	0	0	22	2	8
0	0	0	0	27	0	2
0	0	0	0	0	0	0
0	0	0	0	0	0	0
0	0	0	0	0	0	0
0	0	0	0	0	0	1
0	0	0	0	0	0	0
3	36	4	2	372	8	543
0	0	2	0	0	1	0
0	0	0	0	0	0	50
0	0	0	3	0	0	6
0	0	0	0	0	0	0
0	0	0	0	0	0	0
0	7	0	0	0	0	1
0	0	0	0	0	0	0
0	2	1	3	6	1	0
0	0	0	0	0	0	0
0	0	0	0	0	0	0
0	0	1	0	0	0	0
0	0	0	0	0	3	0
0	0	0	0	0	0	0
0	0	0	0	0	1	0
0	0	0	0	0	0	13
0	0	5	0	0	0	0
0	0	0	0	0	0	0
2	0	0	0	0	0	0
0	0	0	0	0	0	0
0	0	0	0	0	0	0
0	0	0	0	0	0	0
0	0	0	0	0	0	0
0	0	0	0	0	0	0
0	0	4	1	13	0	12
0	2	0	0	3	0	3
0	0	0	0	5	0	0
0	0	0	0	0	0	0
0	0	0	0	0	0	0
0	0	0	0	0	0	0

0	0	0	0	0	0	0
0	0	0	0	0	0	0
316	28	497	247	60	816	3
0	0	0	0	0	50	0
0	0	0	0	0	0	0
0	0	0	0	0	2	0
0	0	0	0	0	0	0
0	0	0	0	0	0	0
0	0	0	0	0	0	0
0	0	0	4	2	2	0
0	0	0	0	1	0	16
0	0	0	0	0	0	0
0	0	0	0	0	0	0
0	0	0	0	0	0	0
0	0	0	0	0	0	0
0	0	0	0	0	0	0
0	0	0	0	0	0	0
0	0	0	0	0	0	0
0	3	33	0	0	0	0
0	0	4	0	4	0	0
0	0	0	0	0	0	0
0	0	0	0	0	0	0
0	0	0	0	0	5	0
5	12	53	0	0	0	0
9	19	73	0	3	0	12
0	0	1	0	0	0	1
0	0	0	0	0	0	0
4	10	0	0	0	0	0
0	0	0	0	0	0	0
0	3	0	1	0	0	0
0	0	0	0	0	0	0
1	0	0	0	1	0	0
0	0	0	0	0	0	0
0	0	0	0	0	0	0
0	0	0	15	0	0	0
0	0	0	0	0	0	0
0	2	0	1	0	0	0
0	0	0	0	0	0	0
0	0	0	0	0	0	0
0	0	0	0	0	0	0
528	168	125	141	24	27	14
0	0	0	1	0	0	0
0	1	3	0	1	0	0
1894	5486	748	407	685	589	863
1	23	26	637	55	3	4
37	12	32	84	43	47	173
31	33	18	45	108	98	177
1	61	13	981	9	9	71

1	0	0	6	12	10	0
6	1	3	29	11	63	13
2	1	4	3	0	10	4
0	0	13	6	2	1	0
5	6	0	0	0	14	4
0	0	0	0	0	0	58
0	0	5	0	0	0	0
0	6	0	0	31	13	4
4	8	6	1	1	7	0
0	1	0	0	0	365	14
2	1	1	18	8	33	27
4	0	0	0	0	6	1
9	0	0	5	0	7	0
1	5	0	0	0	1	2
0	0	1	11	5	0	0
0	3	1	23	1	6	0
0	9	3	2	1	1	1
0	0	0	5	52	8	7
0	0	0	0	0	4	1
0	7	0	0	0	0	0
0	2	0	2	0	1	0
0	0	2	0	3	2	1
0	0	0	0	0	0	0
0	0	0	0	0	0	0
0	0	0	0	0	0	0
2	1	0	6	2	3	2
0	0	0	0	0	2	4
0	0	0	0	0	0	0
0	0	0	0	0	0	1
0	0	0	0	0	0	0
0	0	0	0	0	0	13
0	0	0	0	0	0	2
0	0	0	1	0	0	0
0	1	0	1	0	4	0
0	0	0	0	0	0	4
0	0	0	0	0	7	0
0	0	0	0	7	0	0
0	0	0	0	0	0	0
0	0	0	0	0	0	0
0	0	0	0	0	0	0
0	0	0	0	0	1	0
0	0	0	0	0	0	0
0	0	0	1	0	0	0
0	0	0	0	0	0	0
0	0	0	0	0	0	0
0	0	0	0	1	0	0
0	0	0	0	0	1	0
0	0	0	0	0	0	0

0	0	0	0	0	0	1
0	0	0	0	0	0	0
0	0	0	0	0	0	0
0	0	0	0	0	0	0
0	0	0	0	0	5	0
2	7	1	15	3	32	3
0	0	0	0	0	0	0
76	786	91	77	478	10	404
0	1	0	1	3	0	0
0	0	0	0	0	0	0
0	0	0	0	0	0	0
0	34	0	1	0	0	0
1	0	42	14	129	0	1
0	0	0	0	0	0	0
9	642	1	0	0	1	2
0	0	0	0	0	0	0
1	0	0	0	0	0	0
8	0	0	3	247	0	0
0	0	0	0	0	0	0
0	0	0	1	0	0	0
0	0	0	0	0	0	0
0	0	0	0	0	0	0
0	0	0	0	0	0	0
0	0	0	0	0	0	0
0	0	0	0	0	0	0
0	0	0	0	0	0	0
0	0	0	0	0	0	0
0	0	0	0	0	0	0
0	0	0	0	0	0	0
2	11	5	0	2	0	2
3	1	1	45	1	4	0
0	0	0	0	0	0	0
0	0	0	0	0	0	0
0	0	0	0	0	0	0
0	0	0	2	0	1	0
0	0	0	0	0	0	0
3	0	6	707	54	218	24
0	15	0	0	0	0	0
0	0	0	0	0	2	0
0	0	0	0	0	0	0
0	0	0	0	0	0	0
0	0	0	1	0	0	0
0	0	0	0	0	0	0
0	0	0	0	0	0	0
0	0	0	0	0	0	0
0	0	0	0	0	0	0
0	0	0	0	1	0	0
0	0	11	0	0	0	0

0	0	0	0	0	0	0
0	0	0	0	0	0	0
0	0	0	0	0	0	0
0	0	0	0	0	0	0
0	0	0	0	0	0	0
0	0	0	0	0	0	0
0	0	4	1	18	1	4
0	0	0	0	0	0	0
0	0	0	0	0	0	0
0	0	0	0	0	0	0
0	0	2	0	0	1	0
0	0	0	0	0	0	0
5	24	0	4	0	0	0
7	0	0	0	1	0	0
1	97	68	24	41	181	29
26	30	23	0	0	14	0
0	0	0	0	0	0	0
0	0	0	0	0	0	0
137	24	258	11	14	0	3
0	0	0	32	23	2	1
0	0	0	0	0	0	0
35	17	31	0	1	1	0
1	3	3	0	19	2	95
4	1	14	0	2	3	0
1	1	127	0	0	0	0
1	1	0	22	4	0	1
117	103	19	0	0	8	0
4	10	1	0	1	0	0
0	1	1	0	2	0	0
9	15	20	0	8	2	0
0	0	0	0	0	0	0
0	0	0	0	0	0	0
0	0	0	0	0	0	0
0	0	0	0	0	0	0
0	0	0	0	0	0	0
0	0	0	0	0	0	9
0	0	0	0	0	0	0
0	0	0	0	4	0	0
0	0	0	0	0	0	0
0	0	1	0	1	0	0
0	0	0	0	0	0	0
0	0	0	0	0	0	0
0	0	0	0	3	0	0
0	0	0	0	0	0	0
0	0	0	0	0	0	0
0	0	0	0	0	0	0
0	0	0	0	0	0	0
0	0	0	0	0	0	0

0	0	0	0	0	0	0
224	603	655	314	114	362	6
23	9	197	301	46	35	4
13	3	9	30	18	22	6
16	2	0	0	1	0	0
0	0	0	0	0	455	13
5	3	27	0	0	13	0
0	0	0	0	0	0	0
8	0	10	0	0	0	0
9	3	8	13	0	13	0
6	30	1	0	14	0	2
31	25	8	0	0	0	0
0	0	0	0	0	2	0
0	0	0	3	0	1	0
3	1	0	23	0	0	0
0	0	0	0	1	0	2
0	0	0	0	0	0	0
9	5	1	0	0	0	0
4	0	0	0	0	0	0
0	0	0	2	0	39	3
39	12	7	3	4	1	0
0	0	0	0	1	0	4
0	0	21	0	2	0	0
0	0	0	0	0	0	0
0	0	0	0	0	0	2
0	0	0	0	0	0	0
0	0	0	0	0	0	9
0	0	0	0	0	0	0
0	0	0	0	0	0	5
0	0	0	0	0	3	0
0	0	2	3	0	1	0
0	0	0	0	0	0	0
72	87	42	4	4	0	2
202	132	764	77	16	76	4
26	4	179	370	104	88	67
42	131	402	278	418	31	47
0	0	0	0	2	1	0
1	0	0	1	0	0	0
38	33	20	17	102	31	74
33	73	11	10	112	19	299
2	1	0	23	24	19	3
10	2	179	1	2	49	0
0	0	0	0	0	2	2
32	55	11	0	54	6	36
3	1	147	0	0	5	0
0	0	3	5	12	4	0
0	23	0	0	0	0	1
0	1	0	0	0	0	10

0	0	0	0	9	0	0
0	0	0	0	0	0	0
0	0	0	0	0	0	0
0	0	0	0	0	0	0
0	0	0	0	0	0	0
0	0	0	0	0	0	4
0	0	0	0	0	0	0
0	0	0	0	0	0	0
0	0	0	0	0	0	0
0	0	0	0	0	0	0
0	0	0	0	0	0	0
0	0	0	0	0	10	2
0	0	0	0	0	0	0
0	0	0	0	0	0	0
2	0	1	0	0	0	0
0	0	0	0	0	0	0
0	0	0	0	0	0	0
0	0	0	0	0	0	0
0	0	0	0	8	0	17
0	0	0	0	0	0	0
0	0	0	0	0	0	0
0	0	0	0	0	0	0
0	0	0	0	0	0	0
0	0	0	0	0	0	0
3	0	1	0	0	0	0
0	0	0	0	0	0	0
0	0	0	0	0	0	0
0	0	0	0	0	0	1
0	0	0	0	0	0	0
5	0	39	31	8	4	14
2	0	7	0	1	0	1
1	0	0	0	0	0	0
0	0	2	2	0	0	0
0	0	3	0	0	0	0
0	0	2	1	2	0	0
0	0	0	0	0	0	0
0	0	0	0	0	0	0
0	0	0	0	0	0	0
0	0	0	0	0	0	0
0	0	1	0	0	0	0
1	0	0	0	0	1	0
1	0	33	0	0	38	13
0	0	0	0	0	0	0
0	0	0	1	1	1	2
0	0	0	0	0	0	0
0	0	0	0	6	0	0
0	0	0	0	0	0	0
0	0	0	0	0	0	0

767	323	11702	9796	2318	864	1388
13	0	216	204	158	73	5
0	0	0	0	0	0	0
0	0	0	9	0	0	0
5	0	60	5	37	1	0
0	0	10	1	0	0	0
0	0	0	0	0	0	0
0	0	0	1	0	0	0
0	0	3	1	0	0	0
0	1	0	1	0	0	0
0	0	0	0	0	0	0
0	0	0	0	0	0	0
14	0	128	74	30	5	4
3	0	68	0	0	2	55
0	1	0	104	15	273	142
0	0	0	0	0	0	0
0	0	0	0	0	0	0
0	0	0	0	0	0	0
0	0	0	0	0	0	0
63	0	41	287	15	305	212
0	0	0	5	0	0	0
0	0	0	0	0	0	0
0	0	0	0	0	1	0
0	0	0	1	0	0	0
21	4	423	58	54	30	9
0	0	0	0	0	0	0
0	0	0	0	0	0	0
19	0	6	1229	74	299	254
5104	27	557	17	30	1	3
341	46	293	770	128	302	27
110	1	49	191	23	80	7
0	7	6	13	1	12	2
110	1	15	1659	81	817	15
0	0	0	1	2	23	17
1	2	52	72	14	32	6
4	2	2	401	20	110	13
0	2	0	1	0	5	3
18	2	40	0	1	0	0
0	0	0	0	0	0	0
0	0	0	1	0	30	24
0	0	0	0	0	0	0
0	0	0	0	0	0	0
0	0	0	0	0	0	0
12	0	12	6571	476	341	263
13	1	40	571	67	125	80
738	4	14	0	0	6	0
0	0	0	109	0	11	0
0	0	0	2	0	0	0

0	0	0	0	0	0	0
0	0	0	4	0	1	0
0	0	0	14	0	1	0
0	0	0	14	1	1	2
0	0	0	1	0	0	0
0	0	0	0	0	0	0
0	0	0	0	0	0	0
253	4	0	105	0	1	0
1	0	0	0	0	1	0
1	2	19	0	233	21	62
7	40	0	0	1	1	39
7	0	6	4	0	1	32
0	0	0	0	0	0	0
0	0	0	0	0	0	0
0	0	0	0	0	0	0
4134	64	2036	5259	678	1238	782
471	4	1227	130	24	61	79
0	1	0	0	0	0	0
0	0	1	2	2	10	0
0	0	0	0	0	0	0
37	0	20	4	0	0	2
1	0	2	0	0	0	0
0	0	0	0	0	0	0
0	0	0	0	0	0	0
84	0	56	29	9	6	7
0	0	0	0	0	0	0
12	0	7	5	1	1	1
0	0	0	0	0	0	0
1	0	0	0	1	27	2
9	1	2	1	0	93	4
20	0	96	321	88	102	36
2	1	0	368	17	194	35
58	17	115	27	14	119	3
0	0	0	0	0	0	0
0	0	0	0	0	0	0
0	0	0	0	0	0	0
0	0	0	0	0	0	0
0	0	2	0	0	0	0
1048	489	1897	351	692	251	1127
16	247	16	0	86	9	345
131	64	405	1088	825	13	294
0	0	5	2	236	2	453
6	0	3	0	37	6	20
0	0	0	0	61	0	1
0	0	0	0	0	0	0
0	0	0	0	8	1	4
0	0	0	0	2	0	11
0	0	0	0	2	0	0

0	0	1	0	0	0	0
1	0	0	0	0	0	0
0	0	0	0	0	0	0
0	0	0	0	0	0	0
0	0	0	0	1	0	0
0	0	0	0	0	0	0
0	0	0	0	1	0	2
0	0	0	0	0	0	2
0	0	0	0	0	0	0
0	0	0	0	0	0	0
0	0	0	0	0	0	1
0	0	0	0	0	0	0
0	0	5	0	0	47	0
0	0	0	0	0	0	0
41	22	7	154	21	30	4
102	0	2	146	25	38	5
25	73	41	0	17	28	16
0	0	0	0	0	4	1
0	0	1	62	3	52	33
0	0	0	0	1	0	0
0	0	1	0	0	1	0
0	0	0	0	0	5	6
0	0	0	0	0	0	0
0	0	0	0	0	0	0
0	0	0	0	0	0	0
282	0	3981	304	62	114	29
19	0	366	0	2	239	155
1	0	15	3	2	1	0
0	0	0	0	0	0	0
0	0	9	1	5	0	0
1	4	10	4	199	22	747
0	0	0	0	4	0	10
0	0	0	0	0	0	0
0	0	0	0	5	0	1
0	0	0	0	0	0	0
0	0	0	0	0	0	0
0	0	0	0	0	0	0
0	0	0	0	0	0	0
0	0	0	0	0	0	1
0	0	0	0	0	0	0
0	0	0	0	0	0	1
0	0	0	0	0	0	0
13	16	1	0	0	1	0
0	2	0	0	0	17	1
5	0	0	0	0	9	0
0	0	5	0	2	0	1
0	2	0	0	0	14	0
0	0	0	0	0	0	1

0	0	0	0	0	0	0
0	0	0	0	0	0	0
283	938	2898	5474	1635	526	367
506	5	863	46	0	58	0
200	913	1155	765	715	256	152
14	47	110	7	124	1	193
196	104	78	153	237	190	14
46	824	496	4	428	12	508
62	163	78	0	18	13	93
101	9	17	16	0	50	0
0	8	0	1	0	5	1
0	32	12	0	13	1	115
70	79	18	102	391	32	13
1	0	0	320	20	55	63
135	604	339	43	282	148	56
10	44	48	6	28	1	9
5	10	3	0	13	1	39
171	895	217	0	61	0	0
1	9	379	605	197	372	28
6	149	26	0	6	0	2
0	0	1	0	0	1	15
1	7	0	4	0	0	0
0	42	230	9	216	17	41
4	20	32	45	121	4	69
4	15	13	72	0	61	3
19	38	38	57	133	138	67
0	2	9	43	67	26	129
0	0	0	0	0	0	0
2	3	1	0	36	2	68
0	43	0	0	24	0	0
0	2	43	0	7	0	0
2	0	0	0	54	0	26
0	9	5	0	0	0	0
1	77	213	0	21	0	19
0	13	2	0	15	0	1
0	2	1	0	31	0	29
0	3	1	0	0	0	10
1	0	6	2	0	3	0
0	0	0	0	4	0	0
0	0	0	0	7	0	4
3	0	0	0	0	0	0
0	0	0	0	0	0	2
16	8	0	12	1	0	3
0	0	0	0	19	0	0
0	1	0	0	1	0	11
0	0	0	0	0	0	0
0	0	0	0	0	0	0
0	0	0	0	0	0	17

0	0	0	0	0	0	0
0	0	0	0	3	0	3
1	0	1	17	9	18	0
0	0	0	0	0	0	0
0	0	0	0	0	0	28
0	0	0	0	0	0	3
0	6	0	0	0	0	31
0	0	0	0	0	0	0
0	1	42	20	21	0	0
0	0	0	0	0	3	0
0	0	0	0	4	0	0
0	0	0	0	0	0	0
0	1	0	0	0	0	0
0	0	0	0	0	0	0
0	0	0	0	0	0	0
0	0	0	0	0	0	0
0	0	0	0	6	0	1
0	0	0	0	0	0	0
1	2	0	0	0	0	3
0	0	0	0	0	0	0
1	0	0	0	0	0	0
0	0	0	0	0	0	2
0	0	0	0	0	0	0
0	0	0	0	0	0	0
0	0	0	0	0	0	0
0	1	0	0	0	0	0
0	0	0	0	0	0	1
0	0	0	0	1	0	0
0	1	2	3	1	0	4
0	0	0	0	0	0	0
0	0	0	0	0	0	1
0	0	0	0	0	0	0
0	0	0	0	0	0	0
0	0	0	0	0	0	0
0	0	6	2	1	0	0
0	0	0	1	0	0	0
0	0	0	0	0	0	0
0	0	3	0	0	0	0
0	0	0	0	0	0	0
0	0	0	1	0	0	0
0	0	4	1	1	0	0
0	0	0	0	0	0	0
0	1	0	0	2	0	0
0	0	0	0	0	0	0
841	72	22	1489	385	153	34
205	176	117	42	13	13	5
0	0	0	0	21	0	0
0	0	0	24	0	1	0

0	0	0	0	0	0	0
0	0	0	0	0	0	0
0	0	0	0	0	0	0
12	6	14	8	10	6	4
0	0	0	15	0	2	0
0	0	0	0	0	0	0
0	0	0	32	0	0	0
0	0	0	0	0	0	0
5752	254	706	705	371	167	19
10285	1789	9455	4897	1867	1442	2081
103	33	70	0	10	0	0
547	1	88	8	0	448	3
2507	402	1459	1823	158	507	56
298	9	73	8	6	10	4
1	0	299	0	5	1	13
27	4	49	12	10	4	15
7	0	46	27	14	7	0
150	17	116	32	4	25	0
0	0	0	0	0	1	0
57	3	195	2	11	2	34
22	2	52	62	25	22	5
59	31	26	0	2	2	11
0	0	22	8	3	2	0
1	0	0	4	0	3	0
0	2	12	6	0	4	0
18	28	8	1	9	4	10
11	5	4	3	5	2	2
25	6	40	22	12	3	2
7	2	5	1	1	0	3
0	0	0	0	0	0	0
9	0	7	5	0	0	0
60	3	0	3	0	3	0
0	2	8	5	4	1	6
8	0	6	6	1	0	13
0	0	0	0	0	0	0
0	0	0	0	0	0	0
3	1	1	2	3	0	6
2	13	2	0	0	0	3
0	0	0	0	0	0	0
0	0	1	0	0	0	2
0	0	0	0	0	0	4
0	0	0	0	0	0	0
0	0	0	0	0	0	0
0	0	0	0	0	0	0
1	0	2	0	0	0	1
0	0	0	0	0	0	0
1	0	0	2	0	0	0
0	0	0	0	0	0	0

0	0	0	0	0	0	0
43	11	21	2	4	0	3
0	0	0	0	0	0	0
1	1	0	0	0	2	0
0	0	0	0	0	0	0
0	0	2	2	1	0	0
0	0	0	0	3	0	0
26	11	19	15	16	1	2
0	0	0	0	0	0	0
0	0	0	0	0	0	0
3	1	1	0	0	2	2
3	5	10	1	4	0	0
0	0	0	0	0	0	0
3	1	1	0	1	0	2
0	0	0	0	0	0	0
3	0	5	5	3	1	0
0	0	0	0	0	0	0
0	0	0	0	0	0	0
4	0	2	3	0	0	2
0	0	2	0	0	0	0
0	0	0	0	0	0	0
0	0	0	0	0	0	0
1	2	5	2	1	0	0
0	0	2	0	0	0	0
2	0	1	1	0	1	0
0	0	0	0	0	0	0
0	0	0	3	0	1	0
0	0	0	0	0	0	0
0	0	0	0	0	0	0
0	0	1	1	0	0	2
0	0	0	0	0	0	1
0	0	0	0	1	0	3
0	0	0	0	0	1	0
1	0	0	0	0	0	0
0	0	1	0	0	0	0
0	0	0	0	0	0	0
0	0	0	0	0	0	0
0	0	0	0	0	0	0
0	0	0	0	0	0	0
0	0	0	0	0	0	0
0	0	0	0	0	0	0
0	0	0	0	0	0	0
0	1	0	1	3	1	0
0	0	1	0	0	0	0
0	0	0	0	0	0	1
0	0	0	0	0	0	0
0	0	0	0	0	0	0
2	0	4	0	0	0	0

0	0	0	0	0	7	3
1665	279	625	749	57	382	38
121	122	195	139	168	79	614
249	34	87	54	12	30	1
0	0	1	79	2	49	2
56	9	99	20	1	14	0
73	0	53	14	1	57	3
4	2	64	3	4	5	0
11	1	4	2	0	10	2
104	53	135	36	35	47	84
21	9	12	0	2	3	1
7	8	5	1	11	4	4
0	0	2	3	1	2	0
0	0	0	0	0	2	0
2	0	0	0	0	0	3
0	0	1	0	0	0	0
51	5	25	20	3	15	4
3	0	4	1	0	2	3
4	0	5	0	0	2	1
3	2	0	1	0	0	0
1	1	1	10	1	0	21
2	3	1	1	1	2	0
19	0	10	0	0	1	1
0	0	0	0	0	0	3
0	0	0	0	0	0	0
3	0	0	6	0	1	0
0	0	0	0	0	0	1
1	0	0	2	1	0	2
0	2	0	0	0	0	0
4	0	0	0	0	0	0
0	0	0	0	0	0	0
0	0	0	0	0	0	0
0	0	0	0	0	0	0
0	0	0	0	0	0	0
1	0	0	0	0	0	0
0	0	0	0	0	0	0
614	100	526	1692	17	1842	27
619	64	11	1	8	33	9
0	0	0	0	0	1	0
0	0	0	0	0	0	0
2	2	8	4	0	0	0
0	0	0	0	0	0	2
0	0	0	0	0	0	0
1	5	4	1	0	0	3
0	0	0	0	0	0	0
0	0	0	0	0	1	0
1	0	1	0	0	1	0
3	0	0	0	0	0	0

2	0	0	1	0	1	0
0	0	0	0	0	0	13
4	0	24	1	0	0	0
0	0	0	0	0	0	0
0	0	0	0	0	0	0
1	0	0	0	0	0	0
0	0	0	0	0	0	0
0	0	0	0	0	0	0
0	0	0	0	0	0	0
0	0	0	0	0	0	0
0	0	0	1	0	0	0
0	0	0	0	0	0	0
0	0	0	1	0	0	0
1	0	6	0	0	0	0
0	0	0	0	0	0	0
0	0	0	0	0	0	0
0	0	0	0	0	0	0
0	0	0	12	13	2	15
0	0	0	0	0	0	0
0	0	0	0	0	0	12
0	0	0	0	8	0	15
0	0	0	0	0	2	23
0	0	0	5	0	0	0
0	0	0	0	0	0	11
0	0	0	0	0	0	0
0	0	0	0	0	0	0
0	0	0	0	0	0	0
0	0	0	0	0	0	0
0	0	0	0	0	0	0
0	3	2	21	0	1	0
0	0	0	0	0	0	0
0	0	0	0	0	0	0
0	0	0	3	1	0	0
427	47	366	1285	109	378	20
0	0	0	2	1	1	0
129	7	418	3	0	3	2
1	1	0	3	8	1	0
0	0	0	0	0	0	0
0	0	0	13	2	0	1
0	0	0	0	0	0	0
0	0	0	0	0	0	0
0	0	0	0	0	0	0
634	6	656	84	4	9	1
2	0	7	0	0	0	3
26	0	26	126	18	49	11
0	0	0	0	0	0	2
0	0	0	0	0	0	0
52	22	92	11	13	26	1

0	0	0	0	0	0	0
25	9	92	304	26	227	8
239	22	156	134	44	38	3
58	6	0	0	0	0	0
0	0	0	0	0	0	0
2	17	0	0	0	13	0
0	0	0	0	0	0	0
0	0	0	0	0	0	0
0	0	0	0	0	0	0
106	51	199	240	117	80	67
26	4	84	1083	31	126	4
5	0	9	0	22	26	15
0	0	0	0	2	0	3
0	0	0	0	0	0	4
0	0	0	0	0	0	0
0	0	0	0	4	0	0
0	0	0	0	6	0	5
0	0	0	0	0	0	4
0	0	0	0	14	0	2
0	0	0	0	12	0	2
0	0	0	0	1	0	9
0	0	0	0	0	0	0
0	0	0	0	0	0	0
0	0	0	0	0	0	0
0	0	0	0	0	0	0
0	0	0	0	0	0	0
0	0	0	0	0	0	0
0	0	0	0	0	0	0
0	0	0	0	0	0	0
0	0	0	0	0	0	0
0	0	0	0	0	0	0
0	9	2	0	0	1	0
0	0	0	0	0	0	0
0	0	0	0	0	0	0
0	157	3	0	0	2	0
0	0	0	105	2	48	0
0	0	0	0	0	0	0
0	0	9	1	0	10	2
0	0	0	0	0	0	0
0	0	0	0	0	0	0
4	16	37	0	11	0	45
12	19	8	0	6	0	15
0	0	0	0	0	0	0
0	0	0	0	0	0	4
0	0	0	1	0	0	0
0	0	11	0	0	0	0
1	1	0	0	0	0	0
0	0	0	0	0	0	0

0	3	0	0	14	1	17
0	0	0	0	0	0	0
0	0	0	0	0	0	0
0	1	1	25	0	43	0
0	0	2	0	0	0	0
0	0	0	0	0	0	0
9	4	37	102	86	32	2
4	2	0	0	0	0	0
0	0	0	2	6	0	2
0	0	0	0	0	0	0
0	0	0	1	0	0	2
0	0	0	0	0	0	0
0	0	0	0	3	0	2
0	0	0	0	0	0	0
53	482	14	15	1570	5	1081
0	0	0	0	1	0	6
0	0	0	0	1	0	4
0	0	0	0	0	0	0
0	0	0	0	0	0	0
0	0	0	0	0	0	0
0	0	0	4	0	0	0
0	0	0	0	0	0	0
0	0	0	0	13	0	7
0	1	0	0	0	0	0
0	0	0	0	0	0	0
0	6	0	0	1	0	3
0	0	0	0	0	0	0

78.SUR	78.DCM	82.SUR	82.DCM	84.SUR	85.SUR	85.DCM
0	0	4	0	0	0	0
0	0	0	0	0	0	0
0	1	0	0	0	0	0
0	1	0	0	0	0	0
0	0	0	0	0	0	0
0	6	0	0	0	0	0
0	0	0	0	0	0	0
0	1	0	0	0	0	0
0	24	0	0	0	0	0
37	9	0	0	0	0	0
0	0	0	0	0	0	0
0	0	0	0	0	0	0
0	0	0	0	0	0	0
0	0	0	0	0	0	0
0	0	0	0	0	0	0
0	0	0	0	0	0	0
0	52	0	16	0	0	8
148	48	0	0	1	3	23
22	4	0	13	0	0	3
0	26	0	0	0	0	0
0	2	0	0	0	0	0
0	0	0	0	0	0	0
0	0	0	0	0	0	0
0	0	0	0	0	0	0
0	0	0	0	0	0	0
0	0	0	0	0	0	0
0	0	0	0	0	0	0
0	0	0	0	0	0	0
0	0	0	0	0	0	0
0	0	0	0	0	0	0
0	1	0	0	0	0	0
0	0	0	0	0	0	0
0	8	0	0	0	0	0
0	0	0	0	0	0	0
0	0	0	0	0	0	0
0	0	23	9	0	0	0
2	0	2	79	0	1	19
32	55	0	4	0	11	1
370	98	0	5	5	11	2
0	0	0	0	0	0	0
0	16	0	0	0	0	0
0	0	0	0	0	0	0
0	0	0	0	0	0	0
0	130	0	64	0	0	4
11	18	0	0	0	0	0
0	31	0	0	0	0	0
4	35	0	32	0	0	55

0	57	0	0	0	0	0
0	34	0	0	0	0	0
0	4	0	0	0	0	0
0	22	0	0	0	0	0
0	6	0	0	0	0	0
1	0	0	0	0	0	0
0	9	0	0	0	0	0
0	0	0	0	0	0	0
0	0	0	0	0	0	0
0	0	0	0	0	0	0
0	0	0	0	0	0	0
0	0	0	0	0	0	0
0	3	0	0	0	0	0
4	0	0	0	0	0	0
0	0	0	0	0	0	0
0	0	0	0	0	0	0
0	0	0	18	0	0	0
0	0	0	0	0	0	0
0	0	0	0	0	0	0
0	0	0	0	0	0	0
0	2	0	0	0	0	0
0	0	0	0	0	0	0
0	0	0	0	0	0	0
0	0	0	0	0	0	0
0	0	0	0	0	0	0
0	0	0	0	0	0	0
0	0	0	0	0	0	0
0	3	0	0	0	0	0
0	0	0	0	0	0	0
0	3	0	0	0	0	0
0	33	0	0	0	0	0
0	0	0	0	0	0	0
0	3	0	0	0	0	0
6	0	0	0	0	0	0
0	0	0	0	0	0	0
0	0	0	0	0	0	0
2	0	2	0	0	0	0
0	2	0	0	0	0	0
0	0	0	0	0	0	0
121	2053	1	116	0	0	20
46	176	65	62	1	2	0
15	1	0	20	1	0	28
2	37	0	0	0	0	0
0	0	0	0	0	0	0
0	1	0	0	0	0	0
0	0	0	0	0	0	0
0	0	0	0	0	0	0
0	0	0	0	0	0	0

0	0	0	0	0	0	0
0	0	0	0	2	0	3
0	6	0	0	0	0	1
1	0	0	0	1	0	0
0	0	0	0	0	0	0
2	0	43	348	2263	41071	3932
0	0	0	4	23	280	28
0	0	0	0	1	23	1
0	0	0	2	5	9	6
0	0	0	0	1	14	1
6	0	0	0	0	0	0
0	0	0	0	0	5	0
0	0	0	0	0	1	0
0	1	0	0	0	0	0
0	0	0	0	0	0	0
0	0	0	98	0	0	21
0	8	0	0	0	0	0
0	0	0	0	0	0	0
0	0	0	0	0	48	5
74	1	0	0	0	0	0
0	0	0	0	0	0	0
0	0	0	0	0	0	0
0	36	0	1	0	0	0
0	50	0	0	0	0	0
0	0	0	0	0	0	0
0	0	0	0	0	1	0
0	0	0	0	0	0	0
55	4	0	0	0	0	0
1	2	10	3	0	0	8
0	0	0	0	0	0	0
0	2	0	0	0	0	0
0	0	0	0	0	0	0
0	7	0	0	0	0	0
0	10	0	0	0	0	0
2	13	0	0	0	0	0
0	18	0	0	0	0	0
0	0	0	0	0	0	0
0	0	0	0	0	0	0
0	0	0	0	0	0	0
0	5	0	0	0	0	0
0	0	2	1	0	0	0
0	2	0	0	0	0	0
0	0	0	0	0	0	0
0	0	0	0	0	0	0
148	1	0	6	30	6758	3567
0	0	0	0	0	0	0
0	0	0	0	0	0	0
0	0	1	1	0	0	0

0	20	0	0	0	0	0
0	0	0	0	0	0	0
0	7	0	1	4	1	0
0	0	0	0	0	0	0
0	0	0	0	0	0	0
0	0	0	0	0	0	0
0	0	0	0	0	0	0
0	0	0	0	0	0	0
0	0	0	0	0	0	0
0	0	0	0	0	0	0
3	3	0	0	0	0	0
0	0	0	0	0	0	0
0	253	3	98	2	0	12
15	0	0	0	0	0	0
6	21	0	0	0	0	0
0	0	0	0	0	0	0
0	25	0	0	0	0	0
0	29	0	0	0	0	0
0	0	0	0	0	0	0
1	0	0	0	0	0	0
0	0	0	0	0	0	0
0	0	0	0	0	0	0
0	0	0	0	0	0	0
0	0	0	0	0	0	0
0	0	0	0	0	0	0
0	0	0	0	0	0	0
0	0	0	0	0	0	0
0	0	0	0	0	0	0
0	0	0	0	0	0	0
0	0	0	0	0	0	0
0	0	0	0	0	0	0
0	209	0	26	0	0	5
0	0	0	0	0	0	0
0	4	0	0	0	0	0
0	1	0	0	0	0	0
0	2	0	0	0	0	0
0	0	0	0	0	0	0
0	0	0	0	0	0	0
0	0	0	3	0	0	22
0	5	0	8	6	2	10
0	0	0	0	0	0	0
0	0	0	0	0	0	0
7	13	2	134	0	0	2
10	116	51	431	1	0	1
12	22	0	30	0	1	18
0	0	359	932	7	0	0
0	11	0	20	2	0	7
3	21	0	1	0	0	0

0	0	0	86	2	4	59
0	0	19	2	0	0	0
0	0	0	0	0	0	0
0	0	0	0	0	0	0
0	0	0	0	0	0	0
0	2	2	0	0	0	0
0	2	0	0	0	0	0
0	0	0	0	0	0	0
0	0	0	0	0	0	0
0	0	0	0	0	0	0
0	0	0	0	0	0	0
0	0	0	0	0	0	0
0	0	0	0	0	0	0
0	0	0	0	0	0	0
0	0	0	0	0	0	0
0	0	2	6	0	0	0
0	0	0	0	0	0	0
0	0	0	0	0	0	0
0	0	0	0	0	0	0
0	0	5	23	0	0	16
16	82	0	0	0	0	0
0	0	1	10	0	0	0
0	0	0	0	0	0	0
26	353	2	157	2	2	8
5	4	22	146	22	66	157
0	0	0	0	0	0	0
0	0	0	0	0	0	0
0	0	0	1	0	0	0
0	0	0	0	0	0	0
0	0	0	0	0	0	0
0	0	0	0	0	0	0
0	0	0	0	0	0	0
0	0	0	0	0	0	0
0	0	0	0	0	0	0
0	0	0	0	0	0	0
1	6	7	1	0	0	0
6	130	0	3	0	0	1
0	3	0	0	0	0	0
0	0	0	0	0	38	65
0	7	0	7	0	0	0
0	0	0	0	0	0	0
0	3	0	0	0	0	0
0	0	0	2	0	2	361
0	0	0	0	0	0	297
0	2	0	0	0	0	0
0	2	0	0	0	0	1
0	1	0	0	0	0	0
0	2	0	0	0	0	0

3	1	0	1	0	0	0
0	0	0	0	0	0	0
0	0	0	0	0	0	0
0	2	0	0	0	0	130
0	0	0	0	0	0	0
0	0	11	0	0	2	38
0	0	0	0	0	0	47
1	2	33	4	1	1	0
0	12	0	0	0	0	0
0	4	0	0	0	0	0
0	0	0	0	0	0	0
0	1	0	0	0	0	0
0	0	0	0	0	0	0
0	0	0	0	0	0	0
0	0	0	0	0	0	0
0	0	0	0	0	0	0
0	0	0	0	0	0	0
0	0	0	0	0	0	0
0	0	0	0	0	0	0
0	0	0	0	0	0	0
0	0	0	0	0	0	0
37	3	4	167	6	3	8
0	2	0	0	0	0	0
8	412	0	1	0	0	2
0	0	0	0	0	0	0
0	2	0	0	0	0	0
0	0	0	0	1	0	0
0	0	0	0	0	0	0
0	2	0	0	0	0	0
0	0	0	0	0	0	0
0	0	0	0	0	0	0
0	0	0	0	0	0	0
0	8	0	0	0	0	0
0	1	0	0	0	0	0
0	5	0	0	0	0	0
0	0	0	0	0	0	0
0	1	0	0	0	0	0
0	1	0	0	0	0	0
0	46	0	0	0	0	0
0	0	0	0	0	0	0
0	0	0	0	0	0	0
0	0	0	0	0	0	0
0	0	0	0	0	0	0
0	0	0	0	0	0	0
0	0	0	0	0	0	0
0	0	0	0	0	0	0
0	157	0	0	0	0	0
0	0	0	0	0	0	0

0	0	0	0	0	0	0
0	3	0	0	0	0	0
0	0	0	0	0	0	0
1	16	0	0	0	0	0
0	0	0	0	0	0	0
0	0	0	0	0	0	0
10	13	1	99	0	1	49
0	0	0	0	0	0	0
0	0	0	0	0	0	0
0	0	0	0	0	0	0
0	0	0	0	0	0	0
5662	3768	13	466	14	16	58
34	228	0	142	0	0	3
93	174	0	14	0	0	8
2	53	1	0	0	2	5
38	222	0	79	0	0	16
201	66	1	0	5	0	0
1	45	0	0	0	0	0
15	9	0	38	0	0	5
0	0	1	0	0	0	0
0	3	0	10	0	0	0
0	0	0	0	0	0	0
0	3	0	0	0	0	0
0	58	0	0	0	0	0
0	6	0	31	0	0	9
0	0	0	0	0	1	0
0	0	0	2	0	0	0
0	0	0	0	0	0	0
0	0	0	0	0	0	0
0	15	3	0	0	0	4
0	54	0	0	0	0	0
0	4	0	0	0	0	3
0	0	0	0	0	0	0
0	0	28	7	83	0	0
0	1	0	0	0	0	0
65	4	0	0	0	0	0
264	112	1	16	1	0	1
0	13	0	0	0	0	0
0	0	0	0	0	0	0
0	19	0	0	0	1	0
0	0	0	0	0	0	0
0	0	0	0	0	0	0
0	0	0	0	0	0	0
0	15	0	0	0	0	0
0	0	0	0	0	0	0
0	8	0	0	0	0	0
0	0	0	0	0	0	0
0	0	0	0	0	0	0

0	1	0	0	0	0	0
4	5	0	1	0	0	0
0	11	0	0	0	0	0
0	0	0	0	0	0	0
0	11	0	0	0	0	0
0	0	0	0	0	0	0
0	0	0	0	0	0	0
0	2	0	0	0	0	0
0	1	0	0	0	0	0
0	3	0	0	0	0	0
0	0	0	0	0	0	0
0	0	0	0	0	0	0
0	0	0	0	0	0	0
0	16	0	0	0	0	0
0	5	0	0	0	0	0
0	7	0	0	0	0	0
0	0	0	0	0	0	0
0	6	0	0	0	0	0
0	0	0	0	0	1	0
0	1	0	0	0	0	0
0	0	0	0	0	0	0
5	5	0	0	0	0	0
0	0	0	0	0	0	0
0	0	0	0	0	0	0
0	0	1	1	0	0	1
0	2	0	2	0	0	0
0	2	0	0	0	0	0
0	0	0	0	0	0	0
0	0	0	0	0	0	0
0	3	0	0	0	0	0
0	9	0	2	0	0	2
9	3	0	1	0	0	0
3	2	0	2	0	0	0
16	7	0	0	0	0	0
0	1	0	0	0	0	0
0	5	0	0	0	0	0
1	0	0	0	0	0	0
0	0	0	0	0	0	0
0	0	0	0	0	0	0
0	1	0	0	0	0	0
0	0	0	0	0	0	0
0	0	0	0	0	0	0
0	0	0	0	0	0	0
0	0	0	0	0	0	0
0	0	0	0	0	0	0
0	0	0	0	0	0	0
0	0	0	0	0	0	0
0	0	0	0	0	0	0

0	0	0	0	0	0	0
4	1	0	0	0	0	0
0	0	0	0	0	0	0
0	0	0	0	0	0	0
0	0	0	0	0	0	0
0	1	0	0	0	0	0
0	0	0	0	0	0	0
2	2	0	1	0	0	0
0	0	0	0	0	0	0
12	4	0	0	0	0	0
0	0	0	0	0	0	0
0	0	0	2	0	0	0
0	0	0	0	0	0	0
0	0	0	0	0	0	0
0	0	0	0	0	0	0
0	0	0	0	0	0	0
0	0	0	1	0	0	0
0	0	0	0	0	0	0
0	0	0	0	0	0	0
0	0	0	0	0	0	0
0	3	0	0	0	0	0
0	0	0	0	0	0	0
0	0	0	0	0	0	0
0	0	0	0	0	0	0
2	1	0	0	0	0	0
0	0	0	0	0	0	0
0	0	0	0	0	0	0
0	0	0	0	0	0	0
0	0	0	0	0	0	0
0	14	0	0	1	0	0
0	13	0	0	0	0	0
0	16	0	0	0	0	0
0	0	0	0	0	0	0
0	0	0	0	0	0	0
0	0	0	0	0	0	0
0	52	0	0	0	1	0
0	23	0	0	0	0	7
0	162	0	0	0	0	0
0	54	0	0	0	0	0
0	28	0	0	0	0	0
0	0	0	0	0	0	0
0	1	0	0	0	0	0
181	185	0	0	0	0	0
0	0	0	0	0	0	0
0	0	0	0	0	0	0
0	0	0	0	0	0	0
0	0	0	0	0	0	0
0	0	0	0	0	0	0

0	69	0	0	0	0	0
0	16	0	0	0	0	0
0	10	0	0	0	0	0
0	5	0	0	0	2	0
0	0	0	0	0	0	0
0	0	0	0	0	0	0
60	1	0	9	1	10	2
0	69	0	0	0	0	10
7	218	1	8	0	0	0
0	30	0	59	1	0	0
0	29	0	0	0	0	0
11	19	0	0	0	0	0
0	0	0	0	0	0	0
0	0	0	0	0	0	0
0	0	0	0	0	0	0
0	0	0	0	0	0	0
0	0	0	0	0	0	0
0	0	0	0	0	0	0
5	68	0	3	2	10	25
0	0	0	0	0	0	0
0	0	0	6	0	0	0
0	0	0	0	0	0	0
0	0	0	0	0	0	0
0	0	0	0	0	0	0
0	0	0	0	0	0	0
0	0	0	0	0	0	0
3	22	0	0	0	0	0
0	0	0	0	0	0	0
0	0	8	3	6	0	9
0	2	0	0	0	0	0
0	0	0	0	0	0	0
0	0	0	0	0	0	0
0	0	0	0	0	0	0
0	1	0	0	0	0	0
0	0	25	118	1	0	19
0	0	0	0	0	0	0
0	0	0	0	0	0	0
0	0	0	0	0	0	0
0	0	0	0	0	0	0
0	0	0	0	0	0	0
0	0	0	0	0	0	0
0	0	22	7	0	0	0
0	2	0	0	0	0	0
0	11	0	0	0	0	0
0	0	0	0	0	0	0
5	0	0	0	0	0	0
0	0	0	0	0	0	0
0	0	0	0	0	10	6

0	0	162	116	55	544	160
0	0	0	0	0	0	0
20963	727	10	48	3	2	10
3916	2	0	0	0	0	0
0	0	0	0	0	0	0
15	0	0	0	0	0	0
0	0	0	0	0	0	0
6	0	0	0	0	0	0
8	0	0	0	0	0	0
3	0	0	0	0	0	0
0	22	0	0	0	0	0
0	0	0	0	0	0	0
0	0	0	0	0	0	0
0	0	0	0	0	0	0
0	0	0	0	0	0	0
0	1	0	0	0	0	0
0	0	0	0	0	0	0
0	0	0	0	0	0	0
0	0	0	0	0	0	0
0	0	0	0	0	0	0
0	0	0	0	0	4	0
1	0	0	0	0	0	0
35	0	0	0	0	0	0
0	10	76	98	1	0	496
0	23	0	0	0	0	0
0	0	0	0	0	0	0
0	3	0	0	0	0	0
0	0	1	181	5	24127	15559
0	0	0	0	0	0	0
0	0	0	0	0	0	0
0	6	0	0	0	0	0
0	3	0	3	0	0	0
0	0	0	0	0	0	0
0	0	0	0	0	14	7
0	0	0	0	0	0	0
0	0	336	1	1	0	0
1	0	39	0	0	0	0
0	0	0	0	0	3	1
0	0	0	0	0	0	3
0	0	0	0	0	3	0
203	103	0	0	0	0	0
0	0	403	121	34	0	137
0	2	0	0	0	0	0
662	757	2360	690	39	162	301
22	15	21840	8067	930	10	1511
200	70	650	193	177	12	378
9	195	0	13	51	888	133
294	59	0	155	26	0	4

0	10	0	0	0	0	0
296	2	0	1	0	0	0
59	0	0	0	0	0	0
0	0	0	3	0	0	0
0	0	0	0	0	0	0
1	1	0	0	0	0	0
0	0	0	0	0	0	0
0	29	0	0	0	0	0
152	0	160	14	18	0	8
0	0	37	95	5	5	245
0	43	0	1	0	0	0
1	0	0	0	0	0	0
14	0	0	1	0	0	0
0	0	0	0	0	0	0
0	0	0	0	0	0	0
4	0	0	0	0	0	0
0	7	0	0	0	0	0
0	9	0	1	0	30	5
0	0	2	0	0	0	1
8	0	0	0	0	0	0
5	0	0	0	0	0	0
0	20	0	0	0	0	0
0	0	0	0	0	0	0
0	0	1	0	0	0	0
2	19	0	0	0	0	0
0	12	0	0	0	0	0
0	0	0	0	0	0	0
0	0	0	0	0	0	0
0	0	0	0	0	0	0
0	0	0	0	0	0	0
0	0	0	0	0	0	0
0	0	0	0	0	0	0
0	0	0	0	0	0	0
0	5	4	0	0	0	0
0	0	0	0	0	0	0
1	0	0	0	0	0	0
0	0	0	0	0	0	0
1	0	2	0	0	0	0
0	0	0	0	0	0	0
0	0	9	14	0	0	0
0	0	0	0	0	0	0
0	3	0	0	0	0	0
0	1	0	0	0	0	2
0	0	0	0	0	0	0
0	1	0	0	0	0	0
0	0	0	0	0	0	0
0	0	0	0	0	0	0
0	0	0	0	0	0	0

0	0	0	0	0	0	0
0	0	0	0	0	0	0
0	0	0	0	0	0	0
2	0	0	0	0	0	0
0	0	0	0	0	0	0
80	1	16	1	16	11	20
429	3	0	0	0	0	0
75	230	0	107	1	0	8
0	0	0	0	0	0	0
0	0	0	0	0	0	0
0	0	0	0	0	0	0
0	0	0	0	0	1	13
0	0	0	0	0	0	0
0	0	0	0	0	0	0
0	0	0	0	1	0	0
0	0	0	58	1	9836	3086
0	39	0	0	0	0	0
0	29	0	0	0	0	0
0	0	0	0	0	0	0
4	2	0	60	0	13	2
0	4	0	0	0	0	0
0	0	0	0	0	0	0
0	0	0	0	0	0	0
7	0	0	0	0	0	0
0	0	0	0	0	0	0
0	0	0	0	0	0	0
0	0	0	0	0	0	0
0	0	0	0	0	1	3
0	0	0	0	0	0	0
0	42	102	131	20	12	1330
14	0	0	2	0	0	0
0	0	0	0	0	0	0
0	0	0	0	0	0	0
0	0	0	0	0	0	0
2	0	0	0	0	0	0
1	21	0	0	0	0	0
14	7	0	34	0	0	1
1	0	0	0	0	0	3
0	0	0	0	0	0	0
0	0	0	0	0	0	0
0	0	0	0	0	0	0
0	0	0	0	0	0	0
7	0	0	7	0	0	0
0	0	0	0	0	0	0
0	0	0	0	0	0	0
0	0	1	3	0	64	66
0	0	0	0	0	0	0
0	0	0	0	0	0	0

0	0	0	0	0	0	0
0	0	0	0	0	0	0
0	0	3	0	0	0	0
0	0	0	0	0	0	0
0	0	1	0	0	3	0
0	0	0	0	0	0	0
0	0	0	0	0	0	0
0	1	0	0	0	0	0
0	0	0	0	0	0	0
0	0	0	24	0	0	2
0	0	0	0	0	0	0
0	0	0	0	0	0	0
2	0	1528	1127	12934	61788	7974
0	0	180	388	109	164	33886
18	1	0	2	0	2	0
9	3	0	0	0	0	0
0	0	229	545	60	0	352
0	0	0	0	0	0	0
2	16	0	0	0	0	0
0	0	0	0	0	0	0
0	0	0	0	0	0	0
1	2	0	0	0	0	0
0	7	0	0	0	0	0
15	0	0	0	0	0	0
0	13	0	0	0	0	0
0	0	0	0	0	0	0
8	10	0	0	0	0	0
0	0	0	0	0	0	0
0	3	0	0	0	0	0
1	0	0	0	0	0	0
0	0	17	14	3	3	111
0	8	0	0	0	0	0
0	0	4	3	25	76	5
0	0	0	0	0	0	3
0	0	0	0	8	49	5
0	1	0	0	0	0	0
0	0	0	0	0	0	0
0	3	0	0	0	0	0
0	0	0	0	0	8	1
0	0	0	0	0	0	0
0	0	0	0	0	0	0
0	0	0	0	0	6	1
0	0	0	0	0	0	0
0	0	0	0	0	0	14
0	0	0	0	0	0	5
0	0	0	0	0	4	3
0	0	0	0	1	3	0
0	0	0	0	0	4	0

0	0	0	0	0	0	0
483	11	0	0	0	4	0
694	25	0	0	0	0	0
828	4	0	42	0	0	6
0	0	0	0	0	0	0
2	0	0	0	0	0	0
47	0	7	8	1	0	0
2	0	0	0	0	0	0
0	5	0	0	0	0	0
91	2	0	1	0	0	0
3	12	0	0	0	0	0
9	0	0	0	0	0	0
0	0	1	0	0	0	0
0	0	3	7	80	0	0
10	0	0	0	0	0	0
0	1	0	0	0	0	0
0	0	0	0	0	0	0
0	0	0	0	0	0	0
1	0	0	0	0	0	0
0	0	0	0	0	0	0
5	0	0	0	0	0	0
0	0	0	0	0	0	0
0	0	0	0	0	0	0
0	0	0	0	0	0	0
0	3	0	0	0	0	0
0	0	0	0	0	0	0
0	0	0	0	0	0	0
0	0	0	0	0	0	0
0	0	0	0	0	0	0
1	0	0	0	0	0	0
1	0	0	0	0	0	0
0	0	0	0	0	0	0
0	248	1005	3340	50	245863	120917
129	4	6010	1310	4136	94708	31949
6	2	1	43	0	0	0
1	414	0	39	0	0	0
0	1	0	1	0	1	3
0	0	0	0	0	0	0
0	87	0	0	0	0	0
1	79	7	12	0	0	2
405	1	0	0	0	0	0
13	1	0	0	0	0	0
0	0	0	0	0	0	0
0	84	27	32	1	0	0
33	2	0	0	0	0	0
16	0	0	0	0	0	0
0	0	0	0	0	0	0
0	1	0	0	0	0	0

0	0	0	0	0	0	0
0	0	0	0	0	17	0
0	0	0	0	0	0	0
0	0	0	0	0	0	0
0	0	0	0	7	6	0
0	0	0	0	0	0	0
0	0	0	1	0	3	17
0	0	0	0	0	11	0
0	0	0	1	0	15	17
0	0	0	1	0	31	26
0	0	0	2	0	25	21
0	0	0	0	0	0	0
0	0	19	4	12	0	0
0	0	0	0	0	0	0
0	0	0	0	0	0	0
0	0	0	0	0	0	0
0	0	0	0	0	3	0
0	0	0	0	0	3	0
0	11	0	0	0	0	0
0	0	0	0	0	0	0
0	0	0	0	0	16	14
0	0	0	1	0	9	8
0	0	0	0	0	0	0
0	0	169	25	3	0	0
0	0	0	0	0	0	0
0	0	0	0	0	0	0
0	0	0	0	0	0	0
0	0	0	0	0	0	0
0	1	0	0	0	0	0
0	12	0	1	0	0	0
0	1	0	0	0	0	0
0	0	0	0	0	0	0
0	1	0	0	0	0	0
0	0	0	0	0	0	0
0	0	0	0	0	0	0
0	0	0	0	0	0	0
0	0	0	0	0	0	0
0	0	0	0	0	0	0
0	0	0	0	0	0	0
0	0	0	0	0	0	0
0	0	66	16	1	5627	3478
0	10	0	0	0	0	0
0	0	0	0	0	0	0
1	0	0	1	10	81	9
0	0	0	0	0	0	0
0	0	0	0	0	0	0
6	0	0	0	0	0	0
0	0	0	0	0	0	0

303	1835	62	508	36086	79049	18225
52	129	0	3	12	12	9
0	0	2	0	5	2	2786
0	0	0	0	0	0	0
4	5	0	10	1	9	436
1	4	139	21	4	3	0
0	0	0	0	0	10	3
0	0	0	0	0	2	0
0	0	0	0	7	15	2
0	0	0	0	4	8	5
0	0	0	0	1	8	1
0	0	0	0	4	5	1
0	28	0	2	1	0	0
0	163	0	0	0	0	8
1	6	0	0	0	0	0
0	0	0	0	0	0	0
0	0	0	0	0	0	0
0	0	0	0	0	0	0
0	0	0	0	0	0	0
2	5	0	0	0	0	0
0	0	0	0	0	0	0
0	0	0	0	0	0	0
0	1	0	0	0	0	0
0	0	0	0	0	0	0
43	38	1	5	38	75	45
0	0	0	0	0	0	0
0	0	0	0	0	0	0
335	11	8	9516	2161	118	419970
4	2	1	1	0	0	0
4762	101	1	0	0	0	0
198	15	1	45	0	1	4
0	1	0	2	0	0	0
127	3	0	9	0	4	1
0	0	0	0	0	0	0
213	6	0	0	0	0	0
144	1	0	45	0	0	0
40	8	0	0	0	0	0
0	9	0	0	0	0	0
0	0	0	4	1	1	247
0	0	0	0	0	0	0
0	0	0	1	0	0	29
0	0	0	1	0	0	4
0	0	0	1	0	0	11
46	5	0	9	2	2	255
849	42	0	33	5	2	1293
17	0	0	0	0	0	0
0	0	0	17	0	0	0
0	0	0	65	0	0	0

0	0	0	0	0	0	0
0	0	0	0	0	0	0
1	0	0	1	0	0	0
3	0	0	0	0	0	0
0	0	0	0	0	0	0
4	0	0	0	0	0	0
0	0	0	0	0	0	3
89	9	0	0	0	0	0
24	0	0	0	0	0	0
1	62	0	0	3030	5	1
0	99	0	0	0	0	0
12	84	1	0	0	0	0
0	1	0	0	0	0	0
0	0	0	0	70	0	0
0	0	0	0	11	0	0
20849	1157	1224	78	1	9	8
246	183	93	6	0	0	2
1	0	0	0	0	0	0
0	10	0	0	0	0	0
0	0	0	0	0	0	0
37	3	0	1	0	0	0
0	0	0	0	0	0	0
0	0	0	0	0	0	0
0	0	0	0	0	0	0
123	19	0	0	0	0	0
0	0	0	1	0	0	0
11	1	0	0	0	0	0
0	0	0	0	0	0	0
102	13	0	0	0	0	0
103	3	0	0	0	0	0
758	118	0	0	0	0	0
1	3	0	2	0	0	0
74	6	19	17	0	0	0
0	0	0	0	0	0	0
0	0	0	0	0	0	0
0	0	0	0	0	0	0
0	0	0	0	0	0	0
1	0	0	0	0	0	0
234	685	597	241	3977	12	292
30	217	0	6	0	1	7
11	252	0	0	0	0	1
48	100	0	27	0	0	3
106	54	0	2	0	0	1
0	0	0	0	0	0	0
0	2	0	0	0	0	0
2	2	0	0	0	0	0
0	3	0	0	0	0	0
0	0	0	0	0	0	0

0	0	3	0	0	0	0
0	0	0	0	170	1	9
0	0	0	0	0	0	0
0	0	0	0	16	0	2
0	1	0	0	0	0	0
0	0	0	0	0	0	0
0	2	0	0	0	0	0
1	1	0	0	0	0	0
0	0	0	0	0	0	0
0	0	1	0	0	0	0
0	0	0	0	0	0	0
0	0	0	0	0	0	0
106	0	0	0	0	0	0
0	0	0	1	0	13	23
17	11	0	0	0	0	0
455	6	0	0	0	2	0
9	18	0	0	0	0	1
0	0	0	0	0	0	0
19	8	0	0	0	0	1
0	0	0	0	0	0	0
8	2	0	0	0	0	0
0	0	0	0	0	0	0
0	0	0	0	0	0	0
0	0	0	0	0	0	0
0	0	0	0	0	0	0
5996	256	1	11	1	1	0
1241	155	0	0	0	0	1
12	31	0	0	0	0	0
0	0	0	0	0	0	0
4	1	0	0	0	0	0
0	136	0	3	0	0	0
55	2	2	4	530	0	397
0	0	0	0	0	0	0
0	0	0	0	0	0	0
0	2	0	0	0	0	1
0	0	0	0	0	0	0
0	0	0	0	0	0	0
0	0	0	0	0	0	0
2	0	0	0	0	0	0
0	0	0	0	0	0	0
2	0	0	0	0	0	0
0	0	0	0	0	0	0
0	1	331	504	159	209	5038
11	1	23	4	2	0	3433
112	0	0	0	0	0	0
0	1	0	0	0	0	0
1	0	0	0	0	0	0
0	0	0	0	1	0	0

6	0	0	0	0	0	0
0	0	0	0	0	0	0
598	1220	169	861	9	4	21
1585	59	8	3	1	1	4
406	206	0	15	0	1	0
0	248	0	5	0	1	7
172	127	8	58	0	0	0
13	690	0	15	0	0	58
2	19	0	68	0	1	12
70	0	0	0	0	0	0
0	65	0	36	0	0	3
0	129	0	0	0	0	0
9	15	0	2	0	0	0
0	2	0	0	0	0	0
180	384	0	0	0	0	1
0	38	0	0	0	0	0
3	349	0	0	0	0	0
2	6	0	0	0	0	0
77	40	0	26	1	0	0
0	205	0	0	0	0	0
7	4	5	1	0	0	0
0	1	238	69	204	2	85
3	29	0	0	0	0	0
0	159	1	0	0	0	1
13	27	0	0	0	0	0
25	45	0	0	0	0	0
1	94	0	0	0	0	0
0	0	0	0	0	0	0
0	8	0	0	0	0	0
0	6	0	0	0	0	0
0	0	0	0	0	0	0
0	9	0	0	0	0	0
0	199	0	0	0	0	0
0	16	0	0	0	0	0
0	7	0	0	0	0	1
0	12	0	0	0	0	0
0	0	0	0	0	0	0
0	0	0	0	0	0	0
0	1	0	0	0	0	0
0	0	0	0	0	0	0
0	0	0	0	0	0	0
0	0	0	0	0	0	0
0	0	0	0	0	0	0
0	0	0	0	0	0	0
0	46	0	0	0	0	0
0	0	0	0	0	0	1
0	0	0	0	0	0	0
0	0	0	0	0	0	0

0	0	0	0	0	0	0
0	1	0	0	0	0	0
0	4	0	0	0	0	0
0	0	0	0	0	0	0
0	0	0	0	0	0	0
0	3	0	0	0	0	0
0	0	0	0	0	0	0
0	0	0	0	0	0	0
0	1	0	3	0	0	1
0	0	0	0	0	0	0
2	0	0	0	0	0	0
0	0	0	1	0	0	0
0	0	0	0	0	0	0
0	0	0	0	0	0	0
0	0	0	0	0	0	0
0	0	0	0	0	0	0
0	0	0	0	0	0	0
0	0	0	0	0	0	0
0	0	0	0	0	0	0
0	0	0	0	0	0	0
0	0	0	0	0	0	0
0	4	0	0	0	0	0
0	0	0	0	0	0	0
0	0	0	0	0	0	0
0	0	0	0	0	0	0
0	0	0	0	0	0	0
0	0	0	0	0	0	0
0	0	0	0	0	0	0
0	0	0	0	0	0	0
0	0	0	0	0	0	0
0	3	0	0	0	0	0
0	0	0	0	0	0	0
0	0	0	0	0	0	0
0	0	0	0	0	0	0
0	0	0	0	0	0	0
0	0	0	0	0	0	0
0	0	0	0	0	0	0
0	0	0	0	0	0	0
0	0	0	0	0	0	0
0	0	1	0	0	0	0
0	0	0	0	0	0	0
0	0	0	0	0	0	0
0	0	0	0	0	0	0
0	0	0	0	0	0	0
2100	106	558	1591	4610	3157	160
36	28	0	0	0	0	0
2	0	0	0	0	0	0
0	0	0	0	0	0	0

0	0	0	0	0	0	0
0	0	0	0	0	0	0
0	0	0	0	0	0	0
7	7	12	11	4	24	121
157	2	0	0	0	0	0
0	0	0	0	0	0	0
0	0	0	0	0	0	0
0	0	0	0	0	0	0
847	128	2814	1254	2337	22645	75713
7241	2199	34913	16027	5775	54783	57069
1	0	2529	1704	1262	572	12202
36	0	2503	71	639	8469	405
1048	134	6	36	0	3	3
337	3	416	391	11	0	0
2	71	0	1	0	1	1
28	47	23	3	14	66	2911
147	10	0	0	0	0	0
40	33	14	22	0	0	0
8	2	0	0	0	0	0
15	7	0	0	0	0	1
0	3	42	31	1	0	0
5	23	1	2	1	3	1
1	1	0	0	0	4	1
35	0	2	0	0	0	0
0	0	0	0	0	0	0
1	9	7	3	1	9	21
4	3	0	0	0	0	0
13	6	34	39	2	8	55
1	0	0	0	0	1	2
0	0	0	0	0	0	0
12	1	10	1	0	31	3
15	0	0	0	0	0	0
7	2	5	10	12	48	237
2	7	2	1	0	11	26
3	0	0	0	0	0	0
0	0	4	0	0	0	0
1	13	4	0	5	6	25
0	0	0	0	0	0	0
0	0	0	0	0	0	0
0	0	0	0	0	0	0
0	4	0	0	0	0	0
0	0	0	0	0	0	0
0	0	0	0	0	0	0
0	0	0	0	0	0	0
0	0	0	0	0	0	2
0	0	0	0	0	0	0
2	0	6	2	0	53	11
0	0	0	0	0	0	0

0	0	0	0	0	0	0
9	3	3	0	0	527	21
0	0	0	0	1	20	1
0	0	0	1	0	0	0
0	0	0	0	0	0	0
0	1	9	46	14	28	2
0	1	0	0	0	0	0
6	12	21	20	1	0	6
1	0	5	1	2	10	7
0	0	0	0	0	6	15
0	0	0	3	0	3	10
0	1	1	4	1	1	11
0	0	0	1	0	12	7
0	1	0	2	0	3	158
0	0	0	0	0	0	0
0	0	6	3	1	1	8
0	0	0	0	0	0	0
0	0	0	0	0	2	6
0	0	1	1	0	0	13
1	0	0	0	0	0	9
0	0	0	0	0	11	0
0	0	0	0	0	0	0
1	0	0	0	0	0	0
0	0	0	0	0	0	0
0	0	0	0	0	0	0
0	0	1	0	0	0	5
1	0	0	0	0	0	0
0	0	0	0	0	0	0
0	0	0	0	0	4	1
1	1	0	1	0	1	16
0	0	0	0	0	0	0
0	0	0	0	0	0	0
0	0	0	0	0	0	10
3	0	0	0	0	0	0
0	0	0	0	0	0	0
0	0	0	0	0	0	4
0	0	0	0	0	0	0
0	0	0	1	3	0	0
0	0	0	0	0	2	4
0	0	0	0	0	2	3
0	0	0	0	0	6	0
0	0	0	0	0	0	0
0	0	0	1	0	0	0
0	0	0	0	0	0	0
0	0	0	0	0	0	0
0	0	0	0	0	0	2
0	0	0	0	0	0	0
0	1	0	1	0	0	0

0	0	0	0	0	0	0
1517	117	2732	939	28	4	20
40	311	54	56	23	66	225
132	6	129	71	3	0	0
383	0	0	0	0	0	0
70	12	0	0	0	0	0
24	8	62	43	0	0	0
114	0	0	0	0	0	0
9	5	0	0	0	0	0
50	66	1422	713	327	39	36
6	7	0	0	0	0	0
29	19	29	14	9	1	0
4	2	86	52	45	0	0
0	0	0	0	0	0	0
2	0	0	0	0	0	0
0	0	0	0	0	0	0
24	7	1065	355	202	45	22
9	4	0	19	0	0	4
2	2	4	1	0	0	0
1	0	8	4	0	0	0
0	3	1	1	0	1	18
1	0	119	32	15	7	4
1	0	1	0	0	0	0
0	8	0	0	0	0	0
0	0	0	0	0	0	0
4	0	0	0	0	0	0
0	0	0	0	0	0	0
0	1	23	8	4	0	0
0	0	7	6	0	1	0
0	1	0	1	0	0	0
0	0	4	0	0	1	0
0	0	0	0	0	0	0
0	0	3	0	1	0	0
1	0	15	4	6	0	1
0	0	10	5	1	1	1
0	0	3	0	0	0	0
17474	83	8720	2164	18992	24727	1249
35	45	0	0	0	0	0
0	0	0	0	0	0	0
0	4	0	0	0	0	0
13	4	0	0	0	0	0
0	0	0	0	0	0	0
0	0	0	1	0	0	0
0	34	0	0	0	0	0
4	0	0	0	0	0	0
3	0	15	2	73	0	0
16	0	7	3	31	35	3
5	0	0	0	0	0	0

3	0	5	0	15	27	1
0	0	0	0	0	0	0
36	0	0	0	0	0	0
0	0	2	0	0	9	0
0	0	0	3	8	0	0
0	0	0	0	0	0	0
0	0	0	3	7	12	0
1	0	0	0	0	0	0
0	0	0	0	5	2	0
0	0	0	0	6	11	0
7	0	8	1	15	16	0
0	0	0	0	0	0	0
0	0	2	0	3	0	0
4	0	0	0	0	2	0
1	1	3	0	1	4	0
0	0	0	0	0	3	0
0	0	0	0	0	3	0
0	1	0	0	0	0	0
0	0	0	0	0	0	0
0	1	0	0	0	0	0
0	5	0	0	0	0	0
0	0	0	0	0	0	0
0	0	0	0	0	0	0
0	1	0	0	0	0	0
0	0	0	0	0	0	0
0	0	0	0	0	0	0
0	0	427	213	496	17	2860
0	0	0	0	0	0	0
0	0	129	0	0	0	0
0	1	0	0	0	0	0
0	0	0	0	0	0	0
0	0	0	0	0	0	0
0	0	0	0	0	0	0
817	5	1	2	1	0	1
2	0	1	0	0	0	0
669	18	0	0	0	0	0
9	0	0	0	0	0	0
0	0	0	0	0	0	0
4	0	0	0	0	0	0
1	0	0	0	0	0	0
0	0	17	39	0	0	0
0	0	0	0	0	0	0
495	2	2	11	0	0	0
0	5	1	1	0	71	60
74	0	0	0	0	0	0
0	0	0	0	0	0	0
0	0	9	13	93	31	4737
90	11	12	3	0	0	0

0	0	0	0	0	0	0
2662	29	0	1	0	0	1
18	3	1	8	0	0	0
0	0	1	0	0	0	0
0	0	0	0	0	0	0
0	0	0	0	0	0	4
0	0	0	0	0	0	0
0	0	0	0	0	0	0
0	0	0	0	0	0	0
51	14	0	0	0	0	2
2197	15	0	0	0	0	0
65	24	0	0	0	0	0
0	9	0	0	0	0	0
0	11	0	0	0	0	0
62	0	0	0	0	0	0
0	1	0	0	0	0	0
0	4	0	0	0	0	0
0	0	0	0	0	0	0
0	10	0	0	0	0	0
0	0	0	0	0	0	0
0	0	0	0	0	0	0
0	0	0	0	0	6	5
0	0	0	0	0	0	0
0	0	0	0	0	0	0
0	0	0	0	0	6	1
0	0	0	0	0	0	0
0	0	0	0	0	5	3
0	0	0	0	0	6	6
0	0	0	0	4	0	0
0	0	0	0	0	0	0
0	0	0	0	0	0	0
0	0	0	0	0	0	0
0	0	0	0	0	0	0
0	0	0	0	0	0	0
0	13	4	45	0	0	2
0	0	0	9	0	0	0
0	0	0	0	0	0	0
0	0	0	0	0	0	0
0	0	0	4	19	0	4080
2028	1	0	0	0	0	0
0	27	0	0	0	0	0
9	4	0	0	0	0	46
0	0	0	0	0	0	0
0	17	0	0	0	0	0
0	0	0	0	0	0	0
0	0	0	0	0	0	0
0	0	0	0	0	0	0
0	0	0	0	0	0	0

0	1	0	0	0	0	0
0	0	0	0	0	0	0
0	0	0	0	0	0	0
0	46	0	0	0	0	0
0	0	2	0	0	0	0
0	0	0	0	0	0	0
5	18	9	37	98	0	6
0	18	0	0	0	0	0
0	0	0	0	0	0	0
0	0	0	0	0	0	0
0	1	0	0	0	0	0
0	0	0	0	0	0	0
0	7	0	0	0	0	0
0	0	0	0	0	0	0
31	2137	0	111	0	0	10
0	0	0	0	0	0	0
0	0	0	0	0	0	0
0	0	0	0	0	0	0
0	0	0	0	0	0	0
0	0	0	0	0	0	0
0	0	0	3	0	0	0
0	0	0	0	0	0	0
0	7	0	0	0	0	0
0	1	0	0	0	0	0
0	0	0	0	0	0	0
0	13	0	0	0	0	0
0	0	0	0	0	0	0

98.SUR	98.DCM	100.SUR	100.DCM	102.SUR	102.DCM	109.SUR
0	0	0	0	0	0	0
0	0	0	0	0	0	11
0	0	0	2	0	0	104
0	0	0	2	0	0	19
0	0	0	0	7	0	0
0	0	0	0	0	0	0
0	0	0	0	0	0	0
0	0	0	83	0	0	7
0	0	0	28	0	0	0
0	1	0	0	0	0	0
0	0	0	0	0	0	0
0	0	0	0	0	0	0
0	0	0	0	0	0	1
0	0	0	0	0	0	5
0	0	0	0	0	0	0
0	0	0	0	0	0	0
2	0	7	172	6	0	375
140	72	346	671	572	59	673
0	0	0	0	0	0	1
0	2	0	4	0	0	5
0	0	12	1	0	0	0
0	0	5	0	0	0	0
0	0	0	0	0	0	6
0	0	5	0	0	0	0
0	0	0	0	0	0	0
0	0	0	0	0	0	0
0	0	0	0	0	0	0
0	0	0	0	0	0	0
0	0	0	0	0	0	0
0	0	1	5	0	0	0
0	0	0	4	0	0	9
0	0	0	0	0	0	0
0	0	0	76	87	2	24
0	0	1	7	0	0	0
0	0	0	3	0	0	0
0	0	0	0	0	0	0
0	449	1	2	0	3	59
127	98	0	50	9	8	351
5	24	12	46	43	23	8
0	1	0	0	0	0	42
0	0	0	4	0	0	0
0	0	0	172	64	0	0
1	0	0	160	0	0	0
2	0	7	158	65	0	53
0	0	0	92	29	0	76
0	0	0	33	12	0	0
2	0	0	144	30	6	60

0	0	0	131	0	0	2
0	0	0	16	4	0	1
0	0	0	2	0	2	31
0	0	0	76	29	0	97
0	0	0	53	0	0	1
0	0	0	0	0	0	0
0	0	0	29	31	0	5
1	8	95	0	6	0	0
0	0	0	0	0	0	2
0	0	0	0	0	0	0
0	0	7	0	16	9	6
0	0	0	46	46	0	0
0	0	0	0	5	0	3
0	0	0	13	3	0	1
0	0	0	5	0	0	0
0	0	0	0	0	0	29
0	0	0	0	4	0	0
0	0	0	0	0	0	11
0	0	0	0	0	0	0
0	0	0	14	8	0	0
0	0	0	0	0	0	0
0	0	0	0	0	0	0
0	0	0	0	0	0	0
0	0	0	0	0	0	0
0	0	0	7	0	0	0
0	0	0	0	7	0	0
1	0	0	0	0	0	0
0	0	0	0	0	0	2
0	0	0	0	0	0	0
0	0	0	23	0	0	0
0	0	0	0	0	0	1
0	0	0	0	12	0	0
0	0	0	0	0	0	0
0	0	0	0	0	0	0
0	0	0	0	0	0	0
0	0	0	0	0	0	0
0	0	0	0	0	0	0
0	0	0	0	0	0	0
1	0	0	0	0	0	0
7	4	0	982	22	12	60
1	23	0	27	160	304	236
0	0	9	243	17	5	77
0	0	0	0	0	1	0
0	0	0	9	2	0	1
0	0	0	0	0	0	124
0	0	0	0	0	0	0
0	0	0	0	0	0	22
0	0	0	0	0	0	0

0	0	0	0	0	0	0
0	0	0	0	0	0	0
0	0	0	0	0	0	0
0	0	0	1	0	0	0
0	0	0	0	0	0	0
8	19	0	4	129	0	8
0	0	0	0	1	0	0
0	0	0	0	0	0	0
0	0	0	0	0	0	0
0	0	0	0	0	0	0
0	0	0	0	0	0	0
0	0	0	0	0	0	0
0	0	0	0	0	0	0
0	0	0	0	0	0	26
0	0	0	0	0	0	0
0	0	38	1	0	17	0
0	0	0	88	14	0	1
4	0	0	0	0	0	0
5	0	0	0	0	0	0
0	0	25	8	0	5	4
10	0	0	0	0	0	0
0	0	0	0	0	0	0
0	0	0	0	2	0	27
0	0	0	70	0	0	27
0	0	0	0	0	0	13
4	0	5	9	0	0	0
0	0	26	0	0	0	9
15	0	3	0	0	0	28
6	0	2	3	2	0	23
0	0	0	0	0	0	0
0	0	0	0	0	0	0
0	0	0	0	0	0	0
0	0	0	8	0	0	172
0	0	0	10	0	0	3
0	0	5	28	0	0	57
0	0	0	53	0	0	14
0	0	8	2	0	0	0
0	0	0	4	0	0	0
0	0	0	0	0	0	0
0	0	0	0	0	0	40
0	0	0	0	6	0	0
0	0	2	22	0	0	0
0	0	0	8	2	0	5
0	0	0	0	0	0	0
30	1	0	0	0	1	0
0	0	0	0	0	0	0
0	0	0	0	0	0	39
0	0	0	0	0	0	0

0	0	0	2	0	0	0
0	0	0	0	0	0	4
0	0	1	139	768	0	99
0	0	0	0	0	0	32
0	0	4	0	0	0	0
0	0	0	0	0	0	17
0	0	0	0	0	0	0
0	0	0	0	0	0	0
0	0	0	0	0	0	3
0	0	0	0	0	0	0
0	0	0	28	19	0	27
0	0	0	0	0	0	0
10	0	26	377	71	0	35
0	0	0	0	1	0	0
0	0	0	251	51	0	16
0	0	0	3	0	0	0
0	0	0	137	0	0	0
0	0	0	43	12	0	10
0	0	0	0	0	0	0
0	0	0	3	0	0	2
0	0	0	8	0	0	0
0	0	0	0	0	0	0
0	0	0	0	0	0	0
0	0	0	0	0	0	0
0	0	0	2	4	0	0
0	0	0	0	0	0	0
0	0	0	0	0	0	0
0	0	0	0	0	0	0
0	0	0	0	0	0	0
0	0	7	5	0	0	0
0	5	0	0	0	0	2
0	0	0	0	0	0	0
0	0	0	0	2	0	13
0	0	0	8	2	0	0
0	0	0	6	0	0	0
0	0	0	0	0	0	0
0	0	0	0	0	0	0
0	0	0	0	0	0	0
0	0	100	0	88	0	0
0	0	7	0	2	0	0
0	0	0	0	0	0	0
0	0	0	0	0	0	0
4	17	3	4	2902	3122	3098
0	5	3	1	3	1	12
5	4	0	3	0	1	6
2	0	0	0	0	0	0
1	0	1	0	0	1	7
2	4	0	0	0	0	1

0	0	0	0	29	8	2
0	0	0	0	0	0	0
0	0	0	0	4	4	0
0	0	0	0	0	2	0
0	0	0	0	0	0	0
0	0	0	10	0	0	12
0	0	0	0	0	0	0
0	0	0	0	0	0	0
0	0	0	0	1	0	3
0	0	0	0	0	0	0
0	0	0	0	0	0	0
0	0	0	0	0	0	0
0	0	0	0	0	0	0
0	0	0	2	3	21	10
0	0	0	0	0	0	0
0	0	0	0	0	0	0
0	0	0	0	0	0	0
0	0	0	1	2	0	0
0	0	0	0	0	7	2
0	0	0	0	2	0	4
0	0	0	2	0	0	0
0	0	0	1	3	0	0
0	0	0	0	1	24	0
9	30	11	117	9534	14855	8526
3	5	29	1	21	46	7
0	0	0	0	1	2	0
0	0	0	0	0	0	0
0	0	0	1	32	83	82
0	2	0	0	0	0	0
0	0	0	11	7	14	2
0	0	0	0	0	0	0
0	0	0	0	1	4	1
0	0	0	0	0	0	0
0	0	0	0	5	1	1
0	0	0	0	2	2	3
0	0	0	12	1	0	1
4	7	41	65	514	1435	468
1	1	14	4	247	145	52
0	0	12	10	11	1	9
0	0	8	2	115	54	12
0	0	0	7	24	3	18
0	0	2	0	0	0	0
0	0	0	0	0	0	0
1	0	0	0	0	0	0
0	0	0	0	36	40	56
0	0	1	0	37	0	3
0	0	0	0	0	0	0
0	1	0	0	0	0	0

0	0	0	0	0	0	1
0	0	0	0	0	1	7
0	0	32	2	9	94	1
1	0	0	1	1	0	1
0	0	0	0	64	0	0
0	0	0	0	0	0	0
1	0	0	0	0	0	0
0	0	0	0	0	1	4
0	0	0	0	0	0	3
0	0	0	0	0	0	1
0	0	0	0	0	0	2
0	3	0	0	0	0	7
0	0	0	0	0	0	2
0	0	0	0	0	0	0
0	0	0	0	0	0	2
0	0	0	0	0	0	0
0	0	0	1	0	0	0
1	0	25	3	0	0	0
0	0	0	0	0	0	0
0	36	0	0	0	0	1
0	0	0	0	1	20	0
1	27	3	27	1	5	5
0	0	0	0	0	0	0
34	0	1	428	59	4	99
0	0	1	0	0	0	0
0	0	0	13	0	0	0
0	0	0	1	1	0	0
0	0	0	0	0	0	0
0	0	0	2	2	0	0
0	0	0	3	0	0	0
0	0	0	0	0	0	0
0	0	0	0	0	0	0
0	0	0	15	6	0	4
0	0	0	46	4	0	0
0	0	0	0	0	0	0
0	0	0	0	0	0	4
0	0	0	0	0	65	0
0	0	0	4	0	0	226
0	0	0	57	0	0	17
0	1	0	0	2	0	17
0	0	0	0	0	0	8
0	0	0	0	0	0	27
0	0	0	0	0	0	0
0	0	0	0	0	0	0
0	0	0	3	0	0	2
0	0	0	0	0	0	0
0	0	0	23	0	0	0
0	0	0	0	0	0	54

0	0	0	4	0	0	7
0	0	0	1	0	0	1
0	41	0	0	1665	1	0
1	0	65	63	493	856	25
0	0	0	1	0	0	0
0	0	0	4	0	0	496
4	24	26	161	2	24	30
0	0	0	0	0	0	8
0	0	0	0	0	0	0
0	0	0	0	0	0	0
0	0	0	0	0	0	0
948	1012	863	5967	3948	362	4998
0	0	0	63	122	11	210
3	6	4	196	412	0	22
0	0	0	154	20	0	3
7	24	0	139	152	0	61
15	1	2	174	119	2	80
0	0	4	111	28	1	42
61	5	0	4	0	0	0
0	0	0	6	54	0	3
0	1	3	436	3	0	56
9	0	106	657	14	1	15
0	0	0	0	213	10	66
8	0	0	123	0	0	0
0	0	0	303	18	0	78
0	0	0	0	0	0	713
0	0	0	6	0	9	2
0	0	0	631	6	0	35
2	0	0	0	0	0	0
0	0	1	257	82	1	42
0	0	0	48	162	0	2
0	0	0	12	10	0	0
0	0	0	0	0	0	0
0	0	0	0	6	0	0
0	0	0	0	0	4	4
0	0	0	0	0	0	0
1	11	69	151	87	29	179
0	0	0	0	0	0	201
0	0	0	9	0	0	118
0	0	0	68	24	0	0
0	0	0	0	0	0	0
0	0	0	0	10	0	20
4	0	0	13	4	0	20
0	0	0	0	13	0	22
0	0	0	3	18	0	94
0	0	0	0	5	0	2
0	0	0	0	0	0	0
0	0	0	0	0	0	0

0	1	0	0	0	0	10
0	0	1	0	1	0	6
0	0	0	0	0	0	10
10	2	0	0	0	0	0
0	0	3	77	0	0	0
0	0	0	0	0	0	0
0	0	0	10	0	0	0
0	0	0	0	0	0	21
0	0	0	17	0	0	9
0	0	0	0	0	0	1
0	0	0	0	0	0	43
0	0	0	0	0	0	0
0	0	1	0	1	0	1
0	0	0	3	0	0	28
0	0	0	2	3	0	2
0	0	0	26	0	0	0
0	0	0	3	33	0	0
0	0	0	9	0	0	5
27	12	0	0	0	0	1
0	0	0	0	0	0	0
0	0	0	22	1	0	0
1	0	0	12	8	0	31
0	0	0	0	0	0	13
0	0	0	0	17	0	6
0	0	0	1	0	0	0
0	0	0	1	0	0	0
0	0	0	0	0	0	0
0	0	0	0	0	0	0
0	0	0	3	7	0	0
0	0	0	2	0	0	0
0	0	0	25	2	0	3
0	0	2	0	0	2	7
0	1	0	7	3	0	2
0	2	7	3	2	6	10
0	0	0	10	0	0	4
0	0	0	41	0	0	22
0	0	0	2	26	0	1
0	0	0	0	0	0	0
0	0	0	5	0	0	0
0	0	0	0	0	0	1
0	0	0	0	1	0	0
0	0	0	0	0	0	0
0	0	0	0	0	0	3
0	0	0	0	0	0	0
0	0	0	0	0	0	1
0	0	0	0	0	0	5
0	0	0	1	0	0	0
0	0	0	0	0	0	1

0	0	0	0	1	0	5
0	0	1	0	0	0	2
0	0	0	0	0	0	5
0	0	1	0	0	0	0
0	0	0	1	1	0	0
0	0	0	0	0	0	0
0	0	0	0	0	0	2
0	0	0	1	0	1	2
0	0	0	0	0	0	0
0	0	1	2	3	3	4
0	0	0	0	0	0	0
0	0	0	3	0	0	0
0	0	0	0	0	0	0
0	0	0	0	0	0	0
0	0	0	0	0	0	0
0	0	0	0	0	0	4
0	0	0	0	0	0	0
0	0	0	0	1	0	0
0	0	0	0	0	0	0
0	0	0	0	0	0	0
0	0	0	0	0	0	0
0	0	0	0	0	0	0
0	0	0	0	0	0	0
0	0	0	0	0	0	0
0	0	1	0	1	0	0
0	0	0	0	0	0	0
0	0	0	0	0	0	0
0	0	0	0	0	0	1
0	0	0	0	0	0	0
0	0	87	148	3	0	3
0	0	0	264	182	0	1
0	0	0	81	0	0	0
0	0	0	0	0	0	14
0	0	0	0	0	0	3
0	0	0	0	0	0	0
0	0	0	533	10	0	53
0	0	0	365	1	4	1
1	0	0	452	0	0	0
0	0	2	590	16	0	262
0	0	0	53	0	0	0
0	0	0	4	0	0	0
0	0	0	15	0	0	0
0	0	1	14	0	0	0
0	0	0	8	3	0	10
0	0	0	0	0	0	0
0	0	0	0	1	0	3
0	0	0	0	0	0	3
0	16	40	0	0	1	2

0	0	0	79	87	0	62
0	0	0	19	0	0	31
0	0	0	23	0	0	6
0	0	1	87	7	0	5
0	0	0	19	0	0	0
0	0	0	0	1	0	0
40	458	93	14	17	7	9
1	0	0	35	54	0	49
3	1	0	623	197	11	61
0	0	0	304	2	0	6
0	0	0	43	1	0	1
0	0	0	3	0	0	6
0	0	0	0	0	0	2
0	0	0	0	0	0	0
0	0	0	0	0	0	0
0	0	0	0	0	0	0
0	0	0	1	0	0	0
0	0	0	3	0	0	0
0	0	0	653	17	0	2
0	0	0	7	5	0	0
0	0	0	0	0	0	1
2	0	0	25	0	0	127
0	0	0	37	25	0	0
0	0	0	0	0	0	59
0	0	0	0	0	0	8
0	0	0	0	0	0	0
4	1	3	0	3	0	3
0	0	0	0	0	0	0
0	0	0	0	0	0	0
0	0	0	1	3	0	0
0	0	0	0	0	0	0
0	0	0	0	0	0	0
0	0	0	1	0	0	26
0	0	43	0	0	0	0
3	0	0	0	9	2	9
0	0	0	0	0	0	1
0	1	0	0	0	0	0
0	0	0	0	0	0	0
0	0	0	0	2	1	0
0	0	0	0	1	4	0
0	0	0	0	9	0	12
0	0	0	0	0	0	0
0	0	0	44	36	0	49
0	0	0	8	0	0	0
0	0	0	0	0	0	0
0	0	0	0	0	0	0
0	0	0	0	0	0	0
0	0	0	0	0	0	0

1	0	0	0	2	5	0
0	0	0	0	0	0	0
15	25	35	10	27	63	403
0	0	1	0	2	1	0
0	0	0	0	0	0	0
0	0	0	0	0	0	0
0	0	0	0	0	0	0
0	0	0	0	0	0	0
0	0	0	0	0	0	0
0	0	0	0	0	0	0
0	0	0	7	1	0	95
2	0	0	0	0	0	0
0	0	0	0	0	0	0
0	0	0	0	1	0	0
0	0	0	0	0	0	0
0	0	0	0	0	0	0
0	0	0	0	0	0	0
0	0	0	0	0	0	0
0	0	0	3	0	0	0
0	0	0	0	7	4	12
0	0	0	0	0	0	0
0	3	0	0	0	0	0
0	0	0	0	0	0	0
2	0	0	0	1	0	0
0	0	0	0	66	6	0
0	0	0	0	2	0	0
0	0	0	0	0	0	0
27	0	2	0	0	0	0
0	0	0	0	0	0	0
0	0	0	1	0	2	2
4	0	0	0	0	0	0
0	12	0	1	230	39	337
0	0	0	0	0	0	0
0	0	0	0	0	0	0
0	0	0	0	0	0	0
0	0	0	0	0	0	0
0	0	2	1	0	0	1
0	0	0	0	0	0	0
0	0	0	0	0	0	0
0	0	0	0	0	0	0
6	25	14	32	125	279	74
0	0	0	0	0	0	0
0	0	0	1	2	0	0
471	48	817	1311	1394	1439	2458
58	18	80	72	114	1	203
548	113	859	639	148	78	157
159	47	1999	1062	222	13	93
15	4	307	100	59	58	19

0	0	16	24	6	18	102
12	1	165	112	312	133	323
2	1	28	6	41	4	65
0	0	2	0	2	0	8
0	0	2	43	129	43	16
0	0	3	35	1	0	9
0	0	17	2	9	20	2
6	0	3	17	148	0	7
6	5	3	15	18	35	13
14	0	0	0	0	0	27
54	2	0	11	13	0	10
0	0	6	26	15	0	10
12	0	0	0	0	0	2
0	0	0	16	8	6	1
7	0	70	6	0	0	3
20	1	7	4	0	0	2
0	0	0	4	4	0	1
30	0	8	55	18	0	7
3	0	4	0	0	0	0
5	0	1	6	0	1	11
0	0	3	0	0	0	4
5	0	27	0	0	0	2
0	0	0	0	0	0	0
0	0	0	0	0	0	0
0	0	0	16	0	0	0
10	0	7	0	0	0	4
3	1	0	1	54	0	0
0	0	0	0	0	0	0
0	0	0	2	0	0	8
0	0	0	0	0	0	0
0	0	0	8	0	2	0
0	0	0	0	0	0	0
0	0	0	0	0	0	1
0	0	1	9	0	0	0
0	0	0	0	0	0	0
9	0	0	0	0	0	1
0	0	0	0	0	0	0
0	1	0	1	0	0	0
0	0	0	0	0	0	0
0	0	0	0	0	0	0
0	0	0	1	0	0	0
0	0	0	5	0	0	0
0	0	0	6	0	0	4
0	0	0	15	0	0	0
0	0	0	0	0	0	0
0	0	0	0	0	0	0
0	0	1	0	0	0	0
0	0	0	0	0	0	0

0	0	2	3	0	0	0
0	0	3	0	0	0	0
0	0	0	0	0	0	0
0	0	0	0	0	0	0
0	0	0	5	0	1	0
12	5	33	18	21	1	55
0	0	0	0	0	0	0
3	0	0	1661	279	0	1021
0	0	0	4	1	0	3
0	0	0	0	0	0	0
0	0	0	0	0	0	0
0	0	4	0	0	1	0
0	0	0	0	4	1	7
0	0	0	0	0	0	0
0	0	0	3	0	0	0
0	0	0	0	0	2	0
0	0	1	0	97	2660	16
0	0	1	0	0	11	30
0	0	0	0	0	0	0
0	0	4	0	0	1	0
0	0	5	0	0	49	0
0	0	0	2	0	0	11
0	0	0	0	0	0	0
0	0	0	0	0	0	0
0	0	0	0	0	0	0
0	0	0	0	0	0	7
0	0	0	0	0	0	0
0	0	0	0	0	0	0
0	0	0	3	0	0	0
7	0	0	1	0	0	14
0	1	11	2	4	0	6
0	0	0	0	33	1	0
0	0	0	0	0	0	0
0	0	0	0	0	0	0
0	0	0	0	0	0	0
1	1	1	23	78	0	0
93	53	43	66	2	22	11
0	0	0	0	0	0	0
1	0	0	0	4	1	5
0	0	0	0	0	0	0
0	0	0	0	0	0	0
0	0	0	0	0	0	0
0	0	0	0	0	0	0
1	0	0	0	0	1	0
0	0	0	0	0	0	5
0	0	0	0	0	0	0
0	0	0	0	0	0	0
0	0	0	1	0	0	0

0	0	0	0	0	0	0
0	0	0	0	0	0	0
0	0	0	0	0	0	0
0	0	0	0	0	0	0
0	0	0	0	0	0	0
0	0	0	0	1	0	0
6	3	0	0	0	0	2
0	0	0	1	0	0	5
0	0	0	0	0	0	0
0	0	0	2	0	0	0
0	0	0	0	0	0	0
0	31	0	0	0	0	0
65	5	0	8	0	0	13
362	5	0	0	0	0	0
0	0	7	36	9	0	6
0	0	4	11	63	13	4
4	0	0	0	0	0	0
0	0	0	0	0	0	62
0	0	2	7	52	18	5
0	1	2	2	0	1	19
0	0	0	0	98	14	6
0	0	8	3	4	12	5
0	0	0	6	59	0	3
0	0	0	0	13	0	2
0	0	0	1	0	0	2
0	0	3	5	2	0	12
0	0	5	3	6	19	7
0	0	1	6	0	0	0
0	0	0	9	0	0	0
0	0	0	19	0	4	0
1	0	0	0	0	0	0
0	0	0	41	0	0	0
0	0	0	0	0	0	0
0	0	0	2	7	0	0
1	0	0	0	0	0	0
0	0	0	5	2	0	0
0	0	0	0	0	0	0
0	0	0	0	0	0	0
0	0	0	0	0	0	0
0	0	0	3	0	0	3
0	0	0	0	0	0	0
0	0	0	0	0	0	0
0	0	0	0	0	0	0
0	0	0	0	0	0	0
0	0	0	0	0	0	0
0	0	0	0	0	0	0
0	0	0	0	0	0	0
0	0	0	0	0	0	0

0	0	0	0	0	0	0
4	3	187	42	455	457	199
2	5	94	9	65	453	56
9	2	59	9	9	77	64
0	0	0	0	4	2	0
23	0	149	6	0	0	0
0	0	1	3	26	4	4
0	0	0	0	0	0	6
0	0	0	0	0	0	0
2	2	1	0	0	65	30
0	0	0	2	0	0	0
0	0	0	26	0	0	0
0	0	0	0	0	0	0
1	0	3	1	2	3	0
1	0	46	0	12	1	1
0	0	0	0	0	0	0
0	0	0	10	17	0	0
0	0	0	0	0	0	1
0	0	0	0	0	0	0
4	0	2	3	0	0	5
0	1	1	1	3	0	3
0	0	0	13	0	0	1
0	0	0	4	0	0	0
0	0	0	2	1	0	0
0	0	0	0	0	0	0
0	0	0	0	0	0	1
0	0	0	0	0	0	0
0	1	0	0	0	0	0
0	0	0	0	0	0	0
0	0	0	1	0	0	0
0	0	1	0	1	3	1
0	0	0	0	0	0	0
70	4	0	44	298	26	2
247	18	3	17	43	205	34
5	1	237	33	1	5	0
0	4	0	8	96	1303	10
1	0	4	10	528	1891	61
0	0	3	0	102	400	6
7	3	223	174	46	25	7
4	1	43	223	28	3	23
5	0	1	0	0	0	0
0	0	88	2	15	41	16
201	32	184	52	0	0	0
6	1	18	33	17	0	5
0	0	14	0	54	1	3
5	2	27	24	0	1	7
0	0	0	0	1	18	4
0	0	0	11	0	0	2

0	0	0	0	0	0	0
0	0	0	0	0	0	0
0	0	0	0	0	0	0
0	0	0	0	0	0	0
0	0	0	0	0	0	0
0	0	0	0	0	0	0
0	0	0	0	0	0	0
0	0	0	0	0	0	0
0	0	0	0	0	0	0
0	0	0	0	0	0	0
0	0	0	0	0	0	0
0	0	2	0	2	23	0
0	0	0	0	0	0	0
0	0	0	0	0	0	0
0	0	0	0	1	0	2
0	0	0	3	0	0	0
0	0	0	0	0	0	0
0	0	0	0	0	0	0
0	0	0	0	0	0	0
0	0	0	0	0	0	0
0	0	0	0	0	0	0
0	0	0	0	0	0	0
0	0	0	0	0	0	0
0	0	0	0	0	0	1
0	0	0	0	0	0	0
0	0	0	0	0	0	0
0	0	0	0	3	0	4
0	0	0	1	0	0	0
0	0	0	9	0	0	0
2	15	1	6	57	171	5
0	2	0	0	10	47	11
0	1	0	0	7	34	1
0	0	0	0	4	20	5
0	0	0	0	0	4	5
0	0	0	0	5	8	0
0	0	0	0	0	0	1
0	0	0	0	2	3	1
0	0	0	0	1	4	0
0	0	0	0	0	6	0
0	0	0	0	4	7	1
22	1	0	0	0	0	0
0	0	2	38	51	0	78
0	0	0	0	0	0	0
0	1	0	1	0	0	0
0	0	0	0	0	0	0
0	0	0	0	0	0	0
0	0	0	0	0	0	0
0	0	0	0	1	0	0

461	1994	2030	4238	6923	26281	1647
0	1	35	12	86	42	31
32	0	0	0	0	0	0
0	0	0	0	0	0	0
2	0	0	0	1	2	0
2	0	0	0	0	7	0
0	0	0	0	0	0	0
0	0	0	0	0	0	0
0	0	0	1	0	0	0
0	0	0	0	0	0	0
0	0	1	0	0	0	0
0	0	0	0	0	0	0
0	9	0	6	449	1662	50
0	100	13	678	0	0	0
1	0	0	4	307	3976	11
0	0	0	0	0	0	0
0	0	0	0	0	0	0
0	0	0	0	9	0	0
0	0	0	0	0	0	0
21	5	292	34	3	4	25
1	0	7	4	0	6	1
0	0	0	0	5	50	0
0	0	1	1	0	0	0
0	0	0	1	0	2	0
4	5	15	52	260	714	109
0	0	0	0	0	0	0
0	0	0	7	0	0	0
665	65	0	16	0	33	5
0	0	67	3	10	92	11
91	31	1095	35	31	403	365
17	15	52	19	14	28	113
0	6	1	9	9	9	65
25	1	69	7	6	115	7
26	2	105	63	0	0	0
42	14	54	4	0	6	17
6	4	7	0	2	14	0
5	3	41	11	4	13	9
0	0	0	0	1	0	0
0	0	0	0	0	0	0
5	1	0	0	0	0	0
0	0	0	0	0	0	0
0	0	0	0	0	0	0
0	0	0	0	0	0	0
58	1	0	14	11	1	0
113	41	95	41	108	777	76
0	0	0	0	0	0	4
0	1	0	0	0	0	3
0	9	0	0	0	0	74

10	5	0	0	0	0	0
1	0	0	0	0	0	0
3	0	0	0	0	1	0
5	0	0	0	0	0	0
1	0	0	0	0	0	0
0	0	0	0	0	0	0
0	0	0	0	0	0	0
292	13	0	1	0	0	3
12	32	0	3	0	0	0
600	187	1	51	0	2	0
0	0	0	0	0	0	0
0	18	6	0	0	3	0
0	1	0	0	0	0	0
0	0	0	0	0	0	0
0	0	0	0	0	0	0
776	208	449	412	853	8789	324
113	116	18	72	153	12	5
0	0	14	9	51	528	10
0	1	5	0	0	0	0
0	0	0	0	0	11	1
0	0	0	0	1	4	0
0	0	0	0	2	18	1
0	0	0	1	1	18	0
0	0	1	0	0	12	0
0	0	3	3	1	8	1
0	0	4	3	25	201	2
0	0	0	0	0	0	0
0	0	0	0	0	0	0
16	7	8	11	21	66	11
1	1	0	7	546	17	30
6	17	34	54	449	129	8
4	5	0	0	109	320	2
0	16	16	6	46	110	359
0	0	0	0	0	0	0
0	1	0	0	0	0	0
0	0	0	0	0	0	0
0	0	0	0	1	0	1
0	0	0	0	0	0	6
16	99	358	875	3808	16407	239
5	132	5	48	3	23	7
1	53	1	40	0	1	0
0	32	1	37	0	0	0
19	66	2	100	3	7	1
0	0	0	0	0	0	0
0	0	0	0	0	0	0
0	1	0	1	0	0	0
0	1	0	0	0	0	0
0	0	0	0	0	0	0

0	0	0	0	0	0	0
0	0	0	0	0	0	0
0	0	0	0	0	0	0
0	0	0	0	0	0	0
0	0	0	0	0	0	0
0	0	0	0	0	5	0
0	0	0	0	0	0	0
0	0	0	0	0	0	0
0	0	0	0	0	0	0
0	0	0	2	0	4	1
0	0	0	0	0	0	0
0	0	0	0	0	0	0
0	0	0	0	0	0	0
1	0	0	0	0	0	0
5	0	0	0	0	0	0
7	0	3	0	0	0	0
8	0	0	17	85	243	5
3	0	0	0	0	3	0
35	9	46	12	17	118	2
0	4	0	0	0	0	0
0	1	0	0	0	0	2
0	0	0	0	0	0	0
0	0	0	0	0	0	0
0	0	0	0	0	0	0
0	0	0	0	0	0	0
1	14	147	285	123	11	75
5	80	14	30	71	0	2
0	0	1	2	3	0	0
0	0	0	0	6	41	47
0	0	0	3	8	0	0
0	0	0	240	2	0	9
5	6	16	14	4	0	3
0	0	0	0	2	1	0
0	0	0	0	0	0	0
0	0	0	0	0	0	12
0	0	0	0	0	0	0
0	0	0	0	0	0	3
0	0	0	0	0	0	0
0	0	0	1	3	0	0
0	0	0	0	0	0	0
0	0	0	0	0	0	0
0	0	0	0	0	0	0
41	0	0	0	2	0	0
35	1	156	23	57	27	39
0	12	0	0	0	3	11
0	0	0	13	0	0	0
7	0	1	0	0	0	0
3	0	0	0	0	0	0

0	0	0	0	0	0	0
0	0	0	0	0	3	0
104	69	588	387	1514	1923	1322
62	3	64	18	299	13258	233
26	156	121	304	595	6	644
0	2	0	94	68	92	25
2	12	18	15	20	12	135
2	16	0	441	1826	32	71
0	0	0	64	1998	1	1
8	0	68	6	87	291	237
0	0	1	1	46	78	347
0	0	0	43	361	0	0
10	0	7	80	96	114	105
0	0	0	16	0	0	85
6	7	10	205	184	36	56
0	0	1	1	30	1	19
1	0	0	28	13	0	1
0	0	0	47	110	1	1
50	0	56	17	22	3	46
1	0	0	12	2	0	3
0	0	0	0	0	0	0
1	0	0	0	42	0	0
39	0	0	87	1	1	4
12	0	0	36	0	3	13
2	0	8	10	19	0	14
7	2	18	60	287	102	13
5	0	0	2	0	0	0
0	1	0	0	0	0	5
0	0	0	0	0	0	0
2	3	0	6	5	0	0
0	0	0	23	0	0	0
0	0	0	8	8	0	0
0	0	0	0	0	0	0
0	0	0	18	4	0	0
0	0	0	41	65	0	9
0	0	0	0	0	0	8
0	0	0	14	15	0	5
4	0	0	0	0	0	0
0	0	0	0	0	0	0
0	0	0	9	0	0	1
0	0	0	3	0	0	0
0	0	0	0	0	0	0
16	2	0	1	0	0	0
0	0	0	0	0	0	0
0	0	0	1	0	0	0
0	0	0	2	0	0	16
0	0	0	0	0	0	0
0	0	0	0	0	0	0

0	0	0	0	4	7	9
0	0	0	4	0	0	3
0	0	2	0	0	1	0
5	0	0	0	0	0	1
0	0	0	0	0	0	0
0	0	0	0	0	0	0
0	0	0	0	0	0	0
0	0	0	0	0	0	1
0	0	2	2	15	19	2
0	0	0	0	0	0	0
0	0	0	0	0	0	0
0	0	0	0	0	0	4
0	0	0	0	0	0	4
0	0	0	0	0	0	2
0	0	0	0	0	0	0
0	0	0	2	0	0	19
0	0	0	3	0	0	0
0	0	0	0	0	0	0
0	0	0	3	0	0	0
0	0	0	0	0	0	0
0	0	0	0	0	0	6
0	0	0	0	0	0	0
0	0	0	0	0	0	5
0	0	0	0	0	0	17
0	0	0	0	0	0	3
0	0	0	1	0	0	2
0	0	0	0	0	0	0
0	0	0	0	0	0	1
0	0	0	0	0	0	0
0	0	0	0	0	10	0
0	0	0	0	0	0	0
0	0	0	0	0	0	0
0	0	0	0	0	0	4
0	0	0	0	0	4	0
0	0	1	0	0	3	0
0	0	0	0	0	0	0
0	0	0	0	0	0	3
0	0	0	0	0	0	0
0	0	0	0	0	0	0
0	0	0	0	0	0	1
0	0	1	0	3	2	1
0	0	0	0	0	0	0
0	0	0	0	0	0	0
0	0	0	0	0	0	3
5	1	16	9	11	109	19
0	0	0	5	6	1	0
0	0	0	0	0	0	0
0	0	0	0	0	0	0

0	0	0	0	0	0	0
0	0	0	0	0	0	0
0	0	0	0	0	0	0
3	0	14	28	7	2	6
5	0	0	0	0	0	0
0	0	0	0	8	4	5
0	0	0	0	0	0	0
0	0	0	0	0	0	0
591	10	67	269	295	265	313
871	108	647	1122	3373	4625	1508
109	1	0	3	132	9	38
3	4	1	0	0	134	30
61	17	124	48	431	707	369
3	0	4	3	125	824	160
0	1	0	17	0	0	0
32	0	2	16	11	18	1
3	1	13	3	4	21	30
0	0	1	1	22	4	15
5	0	85	47	13	73	26
0	0	4	12	11	7	1
12	5	2	5	28	0	3
1	0	2	12	45	1	4
8	0	12	0	2	54	0
2	1	0	0	0	0	2
0	0	0	4	7	0	0
0	0	0	6	23	2	2
0	0	1	1	2	6	1
1	0	5	9	38	27	5
0	0	0	0	1	1	3
0	0	0	0	0	0	0
0	0	6	0	3	3	0
0	0	0	0	0	0	0
3	0	1	2	2	3	0
0	1	0	8	3	2	0
0	0	0	0	0	0	0
0	0	0	0	0	0	0
1	0	1	1	0	1	0
0	0	1	0	0	1	0
0	0	0	0	0	0	1
0	0	0	1	0	0	1
0	0	0	26	0	0	0
0	0	0	0	0	0	0
0	0	0	0	0	0	0
0	0	0	0	0	0	0
0	0	0	0	0	0	0
0	0	0	0	0	0	0
0	0	0	0	0	0	0
0	0	0	0	0	0	0

0	3	0	0	0	0	0
0	1	0	1	4	12	22
0	0	0	0	0	0	0
0	0	0	0	0	0	3
0	0	0	2	1	12	0
0	0	0	1	2	3	2
0	0	0	9	0	0	0
0	0	8	7	36	8	6
0	0	0	0	0	0	0
0	0	0	0	0	0	0
0	0	0	1	0	0	0
0	0	0	0	1	1	0
0	0	0	0	0	0	0
0	0	0	2	3	0	0
0	0	0	0	0	0	0
0	0	2	2	3	1	0
0	0	0	0	0	0	0
0	0	0	0	0	0	0
0	0	0	0	3	0	0
0	0	0	6	1	0	0
0	0	0	0	0	0	0
0	0	0	0	0	0	0
0	0	0	0	5	3	1
0	0	0	0	1	0	0
0	0	1	0	0	1	2
0	0	0	0	0	0	0
0	0	0	0	0	0	0
0	0	0	0	0	0	0
0	0	0	0	0	0	0
0	0	0	0	0	0	0
0	0	0	0	0	0	0
0	0	0	1	0	0	0
0	0	0	0	0	0	0
0	0	0	0	0	0	0
0	0	0	0	0	0	0
0	0	0	0	0	0	0
0	0	0	0	0	0	0
0	0	0	0	0	0	0
0	0	0	0	0	0	0
0	0	0	0	0	0	0
0	0	0	0	0	0	0
0	0	0	0	0	0	0
0	0	0	0	2	0	0
0	0	0	0	0	0	0
0	0	0	0	0	0	0
0	0	0	0	1	0	0
0	0	0	0	0	0	0
0	0	0	0	0	0	1

0	0	0	0	0	0	0
92	27	125	194	553	1808	395
18	12	15	183	70	51	28
12	13	11	13	66	275	159
33	2	156	0	0	0	76
23	1	49	60	52	294	175
3	6	7	3	11	96	143
0	0	17	3	4	101	223
0	0	22	4	31	104	37
4	0	12	35	92	68	31
1	0	0	9	8	0	0
5	0	2	12	3	3	5
0	0	0	0	1	0	1
0	0	0	0	0	0	0
2	2	0	0	0	0	0
0	0	0	0	0	0	0
1	1	7	7	20	47	14
0	0	0	2	5	2	3
0	0	0	0	0	1	0
0	0	0	0	2	5	0
0	0	0	0	0	1	0
0	0	0	1	1	9	2
0	0	0	1	2	1	1
0	0	0	0	0	0	0
0	0	0	0	0	0	0
0	0	0	1	2	18	0
0	0	0	4	0	0	0
0	0	0	0	0	3	3
0	0	0	0	0	1	0
0	0	0	0	0	0	0
0	0	0	0	0	0	0
0	0	0	0	0	0	0
0	0	0	0	0	0	0
1	0	0	0	0	2	1
0	0	0	0	0	0	1
0	0	0	0	0	0	0
426	33	101	190	581	2215	275
0	1	92	15	16	4	14
2	1	454	0	0	0	22
0	5	0	0	3	879	0
0	0	16	9	14	147	15
0	0	0	0	0	0	0
0	1	0	6	0	11	51
0	0	1	0	20	146	13
0	0	0	0	0	1	0
0	0	0	0	0	0	0
0	0	0	0	0	0	1
1	0	0	0	0	0	0

0	0	0	0	0	2	0
0	0	0	0	0	0	2
1	0	0	0	0	0	0
0	0	0	0	0	0	0
0	0	0	0	0	0	0
0	0	0	0	0	1	0
0	0	0	0	0	0	0
0	0	0	0	1	0	0
0	0	0	0	0	0	0
0	0	0	0	0	0	0
0	0	0	0	1	1	0
0	0	0	0	0	0	1
0	0	0	0	0	0	0
0	0	0	0	0	0	0
0	0	0	0	0	0	0
0	0	0	0	0	0	0
0	0	0	0	0	0	0
2	2	2	0	0	0	3
0	0	5	4	0	0	0
0	0	0	0	0	0	26
0	0	0	8	0	0	0
0	0	0	35	2	0	0
0	0	0	0	0	0	0
0	0	0	8	0	0	0
0	0	0	0	0	0	0
0	0	0	0	0	0	0
35	0	0	0	0	0	0
0	0	0	0	0	0	0
0	37	0	14	5	3	0
0	0	2	2	0	0	0
0	0	0	0	0	0	0
0	0	0	0	0	0	0
0	0	0	0	1	0	0
88	20	225	26	34	675	97
0	0	0	0	0	11	4
0	0	0	0	0	0	2
0	0	0	1	3	3	1
14	0	1	0	0	1	0
0	0	0	0	0	0	0
0	0	0	0	0	0	0
0	0	0	0	0	0	0
0	0	0	0	0	0	0
0	1	21	0	1	174	32
0	0	0	7	0	0	0
8	0	70	1	4	143	14
0	0	0	0	0	0	0
38	0	0	0	0	0	0
1	0	7	15	185	561	9

0	0	0	0	0	0	1
14	24	66	28	39	206	250
2	0	7	5	0	0	6
0	0	0	0	0	0	0
1	0	2	0	0	0	0
0	0	0	0	0	0	0
0	0	0	0	0	0	2
0	0	0	5	0	0	0
0	0	0	0	0	0	0
3	14	247	40	332	497	744
14	1	32	14	138	32	57
0	0	0	0	0	2	0
0	0	0	8	0	0	0
0	0	0	2	4	0	1
0	0	0	0	0	0	0
0	0	0	2	0	0	0
0	0	0	0	0	0	0
0	0	0	11	0	0	2
0	0	0	0	0	0	0
0	0	0	0	0	0	0
0	0	0	9	0	1	0
0	0	0	0	0	0	0
0	0	0	0	0	0	0
0	0	1	0	0	0	0
0	0	0	0	0	0	0
0	0	0	0	0	0	0
0	0	0	0	0	0	0
0	0	0	0	0	0	0
0	0	0	0	0	0	0
0	0	0	0	0	0	0
0	0	0	0	0	0	0
0	0	2	5	20	0	0
0	0	0	0	0	0	0
0	0	0	0	0	0	2
0	0	10	40	193	6	31
0	0	0	11	1	0	0
1	0	0	0	0	0	0
3	0	0	0	0	0	0
47	0	0	0	0	0	0
0	0	0	1	0	0	0
0	1	19	63	64	2	14
0	1	0	45	25	0	0
0	0	0	0	0	0	0
0	0	0	27	0	0	0
0	0	0	0	0	0	0
0	0	0	0	0	0	0
0	0	0	7	0	0	0
0	1	0	0	0	0	0

0	0	0	39	3	0	5
0	0	0	0	0	0	4
0	0	0	0	0	0	0
5	0	8	147	0	0	9
0	0	0	0	1	2	0
0	0	0	0	0	0	0
2	3	12	19	96	14	3
0	0	0	0	30	0	0
0	0	0	5	0	0	0
0	0	2	0	0	0	0
0	0	0	0	0	0	5
0	0	0	0	0	0	0
0	0	0	13	2	0	0
0	0	0	0	0	0	0
2	0	0	1878	56	4	1456
0	0	0	1	0	0	8
0	0	0	12	1	0	0
0	0	0	0	0	0	0
0	0	0	0	2	0	0
0	0	0	0	0	0	0
0	0	0	0	0	0	0
0	0	0	0	0	0	0
0	0	0	8	0	0	8
0	0	0	7	0	0	1
0	0	0	0	0	0	0
0	0	0	12	0	0	2
0	0	0	0	0	0	0

109.DCM	111.SUR	111.DCM	122.SUR	122.DCM	123.SUR	123.DCM
0	0	0	0	0	0	0
0	0	6	0	14	0	12
8	0	17	0	0	0	15
2	0	3	0	3	0	4
0	0	0	0	0	0	0
0	0	0	0	0	0	2
0	0	3	0	0	0	0
34	1	43	0	0	0	37
1	0	1	0	0	0	1
1	0	0	0	0	0	0
0	0	0	0	0	0	0
0	0	0	0	0	0	0
2	0	0	0	0	0	0
1	0	0	0	0	0	0
0	0	0	4	3	0	6
0	0	1	0	0	0	0
608	4	40	18	240	0	52
115	364	238	60	186	330	55
2	26	56	1	8	0	0
2	0	0	6	0	6	7
0	23	22	12	46	1	2
0	0	0	0	0	0	0
9	0	0	0	0	0	0
0	0	6	0	0	0	0
0	0	0	0	8	0	4
0	0	0	0	0	0	0
0	0	0	0	0	0	1
0	0	0	0	0	0	0
0	0	0	0	0	0	0
0	0	0	0	1	0	0
25	0	0	0	0	0	1
0	0	0	0	0	0	0
90	0	27	0	26	0	4
3	42	42	0	0	0	0
0	0	0	0	0	0	0
0	0	0	0	0	0	0
5	9	39	21	133	0	11
48	0	7	156	33	417	3
21	187	35	121	55	93	203
4	1	32	0	0	0	0
3	16	200	6	141	0	181
30	2	4	0	0	0	0
0	1	24	2	104	38	0
30	26	69	33	73	22	4
2	0	14	3	30	0	9
0	1	95	4	68	0	0
16	2	45	3	49	0	8

0	1	45	0	0	0	6
3	1	13	0	52	0	4
2	0	9	0	13	0	30
26	1	49	7	126	0	10
0	0	14	1	40	0	4
0	1	44	2	22	0	0
3	0	17	0	0	0	5
8	0	0	3	0	31	3
0	0	0	0	0	0	0
0	0	0	0	0	0	0
5	12	1	10	0	2	1
0	0	23	0	0	0	0
0	0	1	0	17	0	0
0	0	5	0	0	0	0
0	0	8	1	7	0	0
10	0	0	0	0	0	0
1	0	0	0	0	0	0
3	0	2	0	7	0	0
0	0	0	0	0	0	0
0	0	0	0	0	0	0
0	0	0	0	0	0	0
0	0	0	0	0	0	0
0	0	0	0	0	0	0
0	0	0	0	0	0	0
0	0	0	0	0	0	0
0	0	0	0	0	0	0
0	0	0	0	1	3	0
0	0	1	0	0	0	0
0	0	0	0	0	0	0
0	2	6	0	8	0	0
0	1	10	0	14	0	4
0	0	6	0	0	0	0
0	0	4	0	0	0	0
0	0	0	0	0	0	0
0	0	0	0	0	0	0
0	0	0	0	0	0	0
1	0	0	0	0	0	1
0	0	0	0	0	0	0
0	0	0	0	3	0	0
8	84	786	55	411	35	568
40	2	38	0	0	0	0
16	145	442	6	0	0	0
0	0	41	0	55	0	103
0	0	0	0	0	214	2
6	0	0	0	0	0	0
0	0	2	0	0	0	26
2	0	0	0	0	0	0
0	0	0	0	0	0	0

0	0	0	0	0	0	0
0	0	0	0	0	0	0
0	0	0	0	0	0	0
0	1	4	0	0	3	0
0	0	0	5	0	0	0
2	0	0	0	0	0	0
0	0	0	0	0	0	0
0	0	0	0	0	0	0
0	0	0	0	0	0	0
0	0	0	0	0	0	0
0	0	0	0	0	0	0
0	0	0	0	0	0	0
0	0	0	0	0	0	0
10	0	0	0	0	0	0
0	0	0	19	0	9	5
0	0	0	0	0	0	0
0	0	13	0	3	0	0
0	0	0	0	0	28	39
0	0	0	0	0	0	0
8	0	0	0	0	1	6
0	0	0	0	0	0	0
0	0	0	0	3	0	0
1	0	11	4	21	0	3
6	0	58	0	8	0	8
0	0	0	0	0	0	4
2	9	8	29	131	6	9
1	19	12	11	0	0	0
18	15	3	0	0	19	0
20	32	13	12	45	0	0
0	0	0	0	0	0	0
0	0	5	0	0	0	0
0	0	3	0	0	0	0
39	0	30	0	0	0	0
1	0	19	2	14	0	6
4	0	16	0	3	0	8
11	0	48	0	0	0	4
0	0	0	1	0	170	59
0	0	13	6	58	0	3
0	0	0	1	7	0	0
3	0	1	0	0	0	2
0	0	0	0	0	0	0
0	1	4	0	0	0	0
0	1	9	0	0	0	4
0	0	0	0	0	0	0
0	0	0	0	0	0	0
0	0	0	0	0	0	0
7	0	3	0	0	0	2
0	0	0	0	0	2	0

0	1	4	0	0	0	0
0	0	0	0	0	0	0
14	13	168	16	118	4	21
290	0	0	0	0	0	0
0	0	2	0	0	0	0
0	0	0	0	0	0	0
0	0	0	0	0	0	0
5	0	0	0	0	0	0
0	0	0	0	0	0	2
0	0	0	0	0	40	10
2	5	8	1	8	0	0
0	0	2	0	0	0	1
26	53	215	190	727	95	54
0	0	0	0	0	0	0
1	0	20	3	22	0	0
0	0	0	14	57	0	2
0	4	33	4	15	0	0
18	25	18	7	22	1	4
0	0	0	0	0	0	0
0	0	0	1	2	0	0
0	0	0	0	0	0	0
0	0	0	0	0	0	0
0	0	0	0	0	0	0
0	0	0	0	0	0	0
0	0	0	0	2	0	0
0	0	0	0	0	0	0
0	0	3	0	0	0	0
0	0	0	0	0	0	0
0	0	0	0	0	0	0
0	0	1	3	0	0	0
0	0	0	0	0	0	18
0	0	0	0	0	0	6
6	0	0	0	0	0	0
0	0	38	0	0	0	1
0	0	2	0	4	0	1
0	0	0	0	0	0	0
0	0	0	0	0	0	0
0	0	0	0	0	0	0
15	43	0	0	2	0	63
1	15	21	0	0	0	0
1	0	0	0	1	0	0
0	0	0	0	0	0	0
677	10	4	1	1	43	24
5	1	65	2	29	0	25
58	1	27	0	5	0	7
0	0	0	0	0	0	0
17	0	19	5	3	5	6
0	0	1	0	0	0	2

0	0	0	0	0	0	4
0	0	0	0	0	1	24
0	0	0	0	0	0	0
2	0	0	0	0	0	0
0	0	0	0	0	0	0
6	0	6	0	8	0	0
0	0	0	6	3	4	39
0	0	0	0	0	0	0
0	0	0	2	9	1	6
0	0	0	0	2	0	3
0	0	0	0	0	0	0
0	0	0	0	0	0	0
0	0	0	0	0	0	0
5	1	0	0	0	0	0
0	0	0	0	0	0	0
0	0	0	0	0	0	0
0	0	0	0	1	0	0
0	0	1	0	0	1	1
1	0	0	0	0	0	0
0	0	0	0	7	1	131
0	0	10	5	56	0	23
0	0	0	0	0	0	0
0	0	0	0	0	0	0
4425	42	361	1516	3245	2030	4218
1	14	72	16	25	36	15
0	0	0	8	10	2	0
0	0	0	0	0	0	0
38	0	0	0	1	0	0
0	0	0	0	0	0	0
1	1	0	0	0	0	0
0	0	0	0	0	0	0
1	0	0	0	0	2	2
0	0	0	0	0	0	0
0	0	0	0	0	0	0
1	0	0	0	0	0	0
0	0	38	0	0	0	0
229	39	132	12	100	179	421
58	1	2	35	48	138	340
22	11	3	6	303	5	19
185	5	15	8	108	18	62
1	0	2	0	83	0	14
0	0	0	0	0	0	0
0	0	0	0	0	0	0
0	0	0	0	0	0	0
18	0	0	0	0	2	0
10	0	5	0	19	0	1
0	0	0	0	0	0	0
0	0	1	0	2	0	1

75	0	0	0	2	0	0
20	0	15	0	0	0	0
0	0	1	0	0	0	0
10	0	2	2	14	0	35
0	0	0	0	0	0	0
0	0	0	0	0	0	0
0	0	0	0	0	0	0
7	0	0	0	0	0	2
3	0	3	0	2	0	7
8	0	0	0	0	0	0
9	0	0	0	0	0	0
5	0	0	0	0	0	0
2	0	0	0	0	0	0
0	0	0	0	0	0	0
4	0	0	0	0	0	0
0	0	0	0	6	0	0
1	0	4	0	0	0	0
0	0	0	0	0	0	0
0	0	0	0	0	0	0
0	0	0	0	0	14	0
0	0	0	0	0	0	0
1	10	0	0	0	2	5
0	0	0	0	0	0	0
68	33	903	237	1038	60	862
1	0	12	6	0	182	11
0	1	8	0	1	1	3
2	0	3	0	0	0	1
0	0	5	0	0	2	0
0	0	0	0	0	0	0
0	0	0	0	2	0	0
0	0	0	0	0	0	0
0	0	0	0	0	0	0
2	0	25	0	14	0	4
0	1	20	7	48	0	0
0	0	0	0	0	0	0
5	0	0	0	0	0	0
0	0	0	1	0	0	6
24	0	1	5	49	0	140
3	1	0	0	0	1	2
0	0	3	0	14	0	6
4	0	0	1	4	0	2
2	0	0	0	0	0	0
0	0	0	0	0	0	2
0	0	7	0	0	0	4
0	0	0	0	5	0	0
0	0	0	0	0	0	0
0	0	34	0	0	0	0
5	0	0	0	0	0	0

3	0	11	0	9	0	15
0	0	0	0	5	0	6
0	0	0	0	0	0	0
85	194	153	49	74	5	2
0	0	0	0	0	0	0
15	244	42	15	3	52	4
2183	10	3	186	72	17	52
0	0	0	0	0	1	0
1	0	0	0	1	0	0
0	0	0	0	0	0	0
0	0	0	0	0	0	0
826	830	3731	652	4312	811	1140
26	27	52	337	216	227	3
7	5	362	18	208	55	36
2	2	135	58	190	38	38
7	3	197	95	258	203	56
12	5	59	20	140	8	39
18	45	25	23	14	9	70
0	0	24	3	27	2	15
0	0	0	0	17	3	18
12	3	192	4	105	0	56
18	23	88	47	236	12	46
65	3	269	0	0	0	17
0	3	49	14	41	0	25
23	0	43	0	16	0	70
288	0	3	0	0	0	0
0	0	0	0	0	0	0
26	2	124	4	71	0	6
0	0	16	0	0	0	0
19	4	40	2	32	0	7
2	1	70	3	0	0	3
0	0	3	0	0	0	0
0	1	98	448	6	3	0
1	0	1	2	2	0	1
7	0	0	0	0	0	3
0	0	0	0	0	0	0
40	61	126	15	72	29	42
4	0	0	0	3	0	8
4	0	13	0	0	0	77
2	0	0	0	0	0	0
0	0	2	23	222	0	6
2	10	2	5	0	0	0
4	37	32	0	5	3	5
2	0	41	0	0	0	20
93	1	12	0	0	0	0
0	0	0	0	14	0	0
0	0	12	1	16	0	6
0	0	0	0	0	0	78

69	0	2	0	0	0	16
4	3	9	3	38	0	12
2	0	1	5	8	0	34
0	15	7	6	0	0	0
0	1	1	0	0	0	0
0	0	0	0	0	0	0
0	0	0	0	0	0	0
5	0	0	2	16	1	0
34	0	21	0	15	0	0
0	3	73	0	0	0	0
30	0	0	0	0	0	0
0	0	47	0	0	0	0
0	0	0	0	0	0	0
0	0	18	0	0	0	2
1	0	4	0	0	0	6
0	1	7	0	18	0	43
0	0	6	0	0	0	0
6	0	9	0	0	0	0
0	0	0	0	0	0	0
0	0	0	0	8	0	0
0	0	14	0	0	0	0
11	0	2	1	20	0	2
20	0	0	0	0	0	0
2	0	0	0	0	0	0
0	0	3	0	13	0	0
0	0	0	0	0	0	1
0	1	7	0	0	0	0
0	0	0	0	0	15	0
0	0	0	0	0	0	0
0	0	3	0	0	0	0
0	0	5	2	0	12	0
2	1	4	0	1	1	0
0	1	3	0	2	1	0
1	5	2	0	3	0	1
4	1	3	1	12	0	6
6	0	1	0	1	0	1
0	0	4	0	21	1	2
0	0	0	0	0	0	0
0	0	0	0	0	0	0
0	0	0	0	0	0	0
0	0	0	0	0	0	2
0	0	0	0	0	0	0
7	0	0	0	0	0	0
0	2	9	0	0	0	0
2	0	0	0	7	0	5
4	2	0	0	0	0	0
0	1	6	0	0	0	0
1	0	0	0	0	0	2

2	0	0	0	9	0	0
0	0	1	0	0	0	0
2	0	0	0	0	0	0
0	0	0	0	0	0	0
0	0	0	0	0	0	0
0	0	1	0	0	0	0
0	0	0	0	0	0	0
0	2	0	0	0	0	0
0	0	0	0	0	0	0
1	3	2	0	3	0	2
0	0	1	0	0	0	0
0	0	0	0	0	0	0
0	0	0	0	0	0	0
0	0	0	0	1	0	0
0	0	0	0	0	0	0
0	0	0	0	0	0	0
0	0	0	0	0	0	0
0	0	0	0	0	0	0
0	0	0	0	0	0	0
0	0	0	0	3	0	1
1	0	0	0	0	0	0
0	0	0	0	0	0	0
0	0	0	0	0	0	1
0	0	0	0	0	0	0
0	0	0	0	0	0	0
0	0	0	0	0	0	0
0	0	1	0	0	0	0
0	0	0	0	0	0	0
0	0	0	0	0	0	0
5	1	16	4	57	0	3
0	0	10	0	4	0	0
0	0	1	0	0	0	2
4	0	0	0	0	0	0
2	0	0	0	0	0	0
0	0	1	0	0	0	3
10	5	179	49	71	14	27
0	17	165	0	80	0	271
0	0	65	11	109	0	20
37	0	124	9	132	0	56
3	8	15	0	36	0	20
0	0	11	0	0	0	5
0	0	0	1	2	0	1
0	58	56	29	405	1	140
4	0	2	0	0	0	1
2	0	1	4	0	0	0
1	0	0	0	0	0	0
0	0	0	0	0	0	0
0	1	0	0	0	110	51

50	0	82	22	117	0	31
23	0	57	2	21	0	38
17	0	4	2	29	0	23
4	0	5	0	12	0	3
0	0	0	0	0	0	0
1	0	0	0	1	0	0
46	39	50	816	450	1180	213
50	1	66	1	99	0	26
64	14	362	66	304	0	71
9	5	188	0	17	0	72
0	1	55	2	71	0	30
2	11	33	0	0	0	0
0	0	37	7	8	0	1
0	0	0	9	49	0	0
0	0	4	0	6	1	0
0	0	0	0	0	9	0
0	0	2	0	0	0	0
0	0	0	0	0	0	0
5	6	314	12	201	0	292
0	0	1	0	0	0	0
0	0	11	0	0	0	26
9	0	16	0	0	0	5
3	0	2	0	0	0	0
5	0	0	0	0	0	0
1	0	0	0	0	0	0
0	0	0	0	0	0	0
4	1	5	1	0	1	2
0	0	0	0	0	120	1
0	0	0	0	0	0	0
0	0	0	0	0	0	0
0	0	0	0	0	0	0
0	0	5	0	0	0	0
15	0	0	0	0	0	0
4	124	21	50	33	0	0
9	0	0	0	0	4	9
0	0	0	0	0	0	0
0	0	0	0	0	0	0
0	0	0	0	0	4	0
0	6	0	0	0	0	0
1	0	0	0	0	0	0
6	0	0	0	0	91	5
0	0	0	0	0	0	0
20	1	27	4	14	0	5
0	0	3	0	0	0	0
0	0	2	0	0	0	0
0	0	0	0	0	0	0
0	0	0	0	0	0	0
0	0	0	0	0	0	0

0	0	0	0	0	0	0
0	0	0	0	0	0	0
35	343	373	138	330	309	381
0	0	0	0	1	0	0
0	0	0	0	0	0	0
0	0	0	0	0	0	0
0	0	0	0	0	0	0
0	0	0	0	0	0	0
0	0	0	0	0	0	0
0	2	0	0	0	0	0
1	0	9	2	14	0	33
0	0	0	0	0	0	0
0	0	0	0	0	0	0
0	0	0	0	0	0	0
0	1	2	0	0	0	0
0	0	0	0	6	0	0
0	0	0	0	0	0	0
0	0	0	0	0	0	0
0	10	10	0	0	0	0
9	0	1	0	0	7	4
0	0	0	0	0	0	0
0	0	0	0	0	0	0
0	0	0	0	0	0	0
1	0	2	0	0	0	2
3	3	2	0	1	0	3
3	4	0	0	0	0	0
0	0	3	0	0	0	0
0	0	0	0	0	0	0
0	5	2	23	0	0	3
0	0	0	0	0	0	0
0	0	0	0	0	0	0
16	0	0	2	0	25	1
0	0	0	0	0	0	0
0	0	0	0	0	0	0
0	0	0	0	0	0	0
0	0	0	0	0	0	0
0	0	0	0	0	0	3
0	0	0	0	0	0	0
0	0	0	0	0	0	0
0	0	0	0	0	0	0
11	34	25	20	11	50	4
0	0	0	0	0	0	0
0	0	0	0	0	0	0
573	695	1051	519	290	760	503
30	41	7	114	37	5	29
522	664	366	305	476	42	22
197	615	380	182	548	39	126
46	343	63	242	74	416	36

25	86	84	10	2	9	1
93	206	100	59	17	60	193
9	20	9	12	1	3	1
3	12	0	14	11	5	0
2	26	16	9	29	0	6
2	22	15	15	21	3	0
2	18	0	32	0	11	0
10	19	38	0	3	0	16
6	4	9	0	5	4	0
8	0	0	0	0	1	0
3	1	8	0	3	1	1
5	15	15	3	0	4	2
0	0	4	3	0	0	0
0	13	15	14	6	4	1
1	41	0	0	0	0	0
4	4	5	3	0	3	0
0	0	11	0	0	2	0
4	35	43	19	112	1	46
8	0	0	0	0	67	5
3	0	0	0	0	1	0
0	0	9	0	0	0	0
1	4	0	42	0	0	0
0	0	0	0	0	0	0
0	0	0	0	0	0	0
0	0	2	0	0	0	0
0	2	2	0	8	4	22
1	0	1	1	0	0	3
0	6	3	0	0	0	0
0	0	3	0	0	0	17
1	0	0	0	0	0	0
0	0	0	0	0	0	15
0	0	2	0	0	0	2
23	1	0	0	0	0	0
0	0	0	0	1	1	2
0	0	0	0	0	0	0
0	0	0	7	1	1	1
0	0	0	0	0	0	0
0	0	0	0	0	0	0
0	1	0	0	0	0	0
0	0	0	0	0	0	0
0	0	0	0	0	0	0
0	2	0	0	0	3	2
0	0	0	0	0	0	0
0	0	0	0	0	0	0
0	0	0	0	0	0	0
0	0	0	2	9	0	0
0	4	0	0	0	0	0
0	0	0	0	9	0	0

0	0	0	2	0	1	0
0	0	0	0	0	1	0
0	0	0	3	0	0	0
0	0	0	3	0	0	0
0	0	0	0	0	0	0
7	9	4	25	24	66	1
0	0	0	0	0	0	0
113	5	302	238	540	142	251
0	0	1	1	1	0	0
0	0	0	0	0	0	0
0	0	0	0	0	0	1
0	0	0	0	0	0	0
3	0	0	0	2	0	38
0	0	0	0	0	0	0
1	0	0	0	0	0	0
0	0	0	0	0	0	0
1	0	0	0	0	0	0
8	0	0	0	0	29	525
0	0	0	8	0	76	4
0	0	0	0	0	75	0
0	0	0	0	0	0	0
0	0	0	0	0	0	0
0	0	0	0	0	35	1
0	0	0	0	0	0	0
0	0	0	0	0	0	0
0	0	0	0	0	0	0
0	0	0	0	0	3	0
0	0	0	0	0	0	0
0	0	0	0	0	0	0
8	1	5	0	0	0	3
2	40	23	16	10	6	0
3	0	0	0	0	0	0
0	0	0	0	0	0	0
0	0	0	0	0	0	0
0	18	18	0	0	0	0
1	7	7	0	14	0	0
88	164	52	944	372	395	32
0	0	0	0	0	0	0
4	0	0	0	0	0	0
0	0	0	0	0	0	0
0	0	0	0	6	0	0
0	0	0	3	0	1	0
0	0	0	0	0	0	0
0	0	0	0	0	0	0
9	0	0	0	0	100	1
0	0	0	0	0	0	0
0	0	0	0	0	0	0
0	1	3	0	0	29	0

0	0	0	0	0	0	0
0	0	0	0	0	0	0
0	0	0	0	0	0	0
0	0	0	0	0	0	0
0	0	0	0	0	0	0
0	0	0	0	0	0	0
2	0	4	1	10	0	11
0	0	10	0	0	0	1
0	0	0	0	0	0	0
0	0	0	16	0	925	44
0	0	0	0	0	1	0
0	0	0	0	0	0	0
4	0	0	2	0	0	0
0	0	0	0	0	0	0
2	29	24	13	16	8	6
13	0	0	5	0	184	3
0	0	0	0	0	0	0
3	0	0	0	0	0	0
8	11	12	0	14	25	3
21	4	2	28	1	43	4
10	0	0	0	0	0	0
13	10	4	0	0	50	0
59	8	8	2	8	0	7
0	0	0	0	0	1	0
5	2	0	0	0	6	1
8	0	0	0	0	146	13
5	2	0	5	0	0	0
1	13	13	0	0	3	6
5	2	10	11	2	26	0
0	0	1	0	0	0	0
0	0	0	0	0	0	0
0	0	0	0	3	0	3
0	0	0	0	0	0	0
0	0	0	0	0	0	0
0	0	0	0	0	0	0
0	0	0	0	0	0	0
0	0	0	0	0	1	0
0	0	0	0	0	0	0
0	0	0	0	0	0	0
4	0	1	0	0	1	0
0	0	0	0	0	0	0
0	0	0	0	0	0	0
0	0	0	0	0	0	0
0	0	0	0	0	0	0
0	0	0	0	0	0	0
0	0	0	0	0	0	0
0	0	0	0	0	0	0
0	0	0	0	0	0	0

0	0	0	0	0	0	0
196	70	97	115	15	680	121
37	10	13	72	6	119	49
21	36	41	168	70	84	2
3	0	0	0	0	0	0
0	8	0	0	0	0	0
0	4	3	0	2	3	0
9	0	0	0	0	0	0
0	0	0	0	0	0	0
18	3	6	9	0	25	6
0	3	5	0	4	0	6
0	0	0	0	0	0	0
0	0	0	0	0	0	0
0	3	2	0	0	0	0
5	6	13	0	0	0	0
4	0	1	0	0	0	2
2	0	0	0	3	0	0
0	0	0	12	0	0	0
0	0	0	0	0	0	0
5	1	1	6	3	0	0
5	1	2	0	6	0	0
0	0	1	0	0	0	4
0	0	0	0	0	0	0
0	0	3	0	2	3	2
0	0	0	0	0	0	0
6	0	0	0	0	0	0
0	0	0	0	0	0	0
0	0	0	0	0	0	0
0	0	0	0	0	0	2
0	0	0	0	0	0	0
0	0	0	0	0	0	0
4	0	0	0	0	0	0
3	0	8	0	0	0	0
16	31	18	5	0	201	15
20	132	77	3	0	32	19
60	0	41	36	72	206	137
84	1	2	10	0	170	8
10	4	5	0	0	0	0
16	22	52	22	42	6	30
56	103	103	27	68	28	23
0	16	5	0	0	18	10
30	0	4	2	0	38	1
0	123	74	0	21	1	4
9	2	13	2	44	3	39
7	4	3	0	3	1	0
15	13	58	6	0	12	3
5	0	1	0	15	0	0
8	0	3	0	11	0	28

0	3	7	0	0	0	9
0	0	0	0	0	0	0
0	0	5	0	0	0	0
0	1	6	0	7	0	0
0	0	0	0	0	0	0
0	0	0	0	0	0	2
0	0	0	0	0	0	0
0	0	0	0	0	0	0
0	0	0	0	0	0	0
0	0	0	0	0	0	0
0	0	0	0	0	0	0
0	0	0	0	0	0	0
0	0	0	0	0	0	0
0	0	0	0	0	0	0
0	0	0	0	0	0	0
2	0	0	0	0	0	0
0	0	0	0	0	0	0
0	0	0	0	0	0	0
0	0	7	0	4	0	15
0	0	0	0	0	0	3
0	0	0	0	0	0	0
0	0	0	0	0	0	0
0	0	0	0	0	179	16
0	0	0	0	0	0	0
0	0	0	0	0	57	9
0	0	0	0	0	0	0
0	0	0	4	0	0	0
0	0	0	0	0	0	0
0	0	0	0	0	5	0
1	3	6	3	14	0	3
0	0	0	0	1	0	2
0	0	0	0	0	0	0
0	2	1	0	3	0	0
0	5	2	0	4	0	0
0	0	0	0	0	0	0
0	0	0	0	0	0	0
0	0	0	0	0	0	0
0	0	0	0	0	0	0
0	0	0	0	0	0	0
0	0	0	0	1	0	0
0	0	0	0	0	0	0
45	33	35	1	4	0	0
0	0	0	0	0	0	0
0	1	0	0	0	0	0
0	0	0	0	0	0	0
0	0	0	0	0	0	0
0	0	0	0	0	0	0
3	0	1	0	0	0	0

273	1978	2246	2420	13183	253	1085
57	13	28	7	215	126	202
0	0	0	0	0	0	0
0	0	0	0	0	0	0
4	0	1	0	4	0	0
0	1	0	0	1	0	0
0	0	0	0	0	0	0
0	0	0	0	0	0	0
0	0	0	1	1	0	0
0	0	0	0	0	0	0
0	0	0	0	0	0	0
0	0	0	0	0	0	0
5	9	11	2	18	2	26
0	126	198	0	12	0	3
313	1	0	2	6	413	339
0	0	0	0	0	0	0
0	0	0	0	0	0	0
0	0	0	0	0	0	0
0	0	0	0	0	0	0
59	17	14	6	5	86	17
37	1	4	1	1	1	0
0	0	0	0	0	0	0
0	0	1	0	0	0	0
5	0	0	0	2	0	0
4909	26	13	135	49	202	51
0	0	0	0	0	0	0
2	0	0	0	0	0	0
682	19	17	112	45	56	4
0	23	30	51	17	10	21
390	37	44	184	29	105	67
114	0	4	72	31	99	16
500	63	36	1	2	0	2
10	27	39	98	12	37	24
0	6	8	82	145	2	3
27	89	67	117	3	10	1
2	0	4	8	2	5	3
153	11	5	10	12	0	0
0	0	0	0	0	0	0
1	0	0	0	0	0	0
0	0	0	0	0	0	0
0	0	0	0	0	0	0
0	0	0	0	0	0	0
0	0	0	0	0	0	0
51	85	3	184	32	109	86
1154	88	35	13	69	10	9
0	0	2	0	0	0	0
3	0	0	14	5	45	5
1	0	0	0	0	0	0

1	0	0	0	0	0	0
0	0	0	0	0	0	0
2	0	0	0	0	0	0
8	0	0	0	0	0	0
0	0	0	0	0	0	0
0	0	0	0	0	0	0
0	0	0	0	0	0	0
0	0	2	0	0	1	3
0	0	0	0	0	0	0
2	0	20	245	726	3	109
0	0	4	0	14	0	0
6	0	12	1	1	0	0
0	0	2	0	5	0	0
0	0	0	0	0	0	0
0	0	0	0	0	0	0
5743	354	195	1558	1777	293	58
2	101	210	710	990	404	148
69	6	3	53	78	9	2
0	0	0	28	2	7	0
4	0	0	10	1	1	0
5	1	1	2	2	0	0
1	1	0	1	2	0	0
4	0	0	5	3	2	0
1	0	1	1	2	0	0
6	3	3	2	9	1	0
30	3	1	20	27	8	0
1	0	1	1	0	0	0
0	0	0	0	0	0	0
52	20	14	28	10	29	4
257	19	13	30	9	16	9
419	49	44	115	85	8	7
73	15	5	64	59	33	4
190	23	19	50	7	42	3
0	0	0	0	0	0	0
0	0	0	0	0	0	0
0	0	0	0	0	0	0
0	0	0	0	0	1	0
0	0	0	1	0	1	0
1206	1626	1031	1017	2682	1002	1729
1	25	255	42	808	34	452
52	3	138	193	768	173	424
75	3	77	30	923	9	390
1	26	70	44	177	5	18
0	0	1	0	0	0	5
0	0	0	0	38	0	11
1	0	2	0	5	0	5
0	0	6	1	8	0	14
0	0	0	0	0	0	0

0	0	0	0	0	0	0
0	1	0	0	0	2	0
0	0	0	0	0	0	0
0	0	0	0	0	0	0
1	0	1	0	0	0	0
0	0	0	0	0	0	0
0	0	1	0	0	0	1
0	0	1	0	0	0	3
0	0	0	3	0	0	0
0	3	2	0	2	1	1
0	0	0	0	1	0	0
0	0	0	0	0	0	0
0	0	0	0	0	0	0
0	0	0	0	0	0	0
0	0	0	0	0	0	0
0	0	0	0	0	0	0
9	30	33	5	26	2	0
6	1	0	0	0	0	0
120	23	7	51	111	0	1
0	0	0	1	0	1	0
9	14	1	0	1	5	0
0	0	0	0	0	52	14
0	0	0	0	7	0	0
0	0	0	0	0	0	0
0	0	0	0	0	0	0
101	205	164	89	98	93	83
0	109	25	3	6	0	0
0	0	1	0	29	0	0
0	0	0	0	0	0	0
0	0	0	0	0	0	9
6	3	393	11	195	0	317
5	1	23	2	10	486	36
0	0	0	0	0	64	7
0	0	7	0	0	0	0
26	0	0	0	0	0	0
0	0	0	0	0	0	0
1	0	0	0	0	0	0
0	0	0	0	0	0	0
0	0	1	6	1	2	0
0	0	0	0	0	0	0
0	2	0	0	0	0	0
0	0	0	0	0	3	1
1	0	0	0	0	1	0
26	9	10	0	0	0	0
3	0	0	12	1	2	0
0	0	2	0	0	0	3
1	1	0	0	0	0	0
0	0	11	0	0	0	0

0	0	0	0	0	0	0
0	0	0	0	0	0	0
1308	1784	1093	1009	386	1720	439
45	38	28	84	4	133	5
293	166	218	390	318	1223	215
162	6	155	5	39	0	55
19	20	32	47	87	133	12
342	50	622	75	942	13	447
1	1	78	10	64	2	39
39	38	13	94	3	19	0
536	0	17	95	80	457	24
1	0	98	4	67	0	10
36	65	62	57	68	11	30
11	21	2	103	24	422	44
14	61	135	25	120	66	24
5	209	148	106	48	89	1
3	0	77	3	21	0	5
0	9	6	0	0	0	0
34	106	62	55	8	34	3
0	2	34	0	0	0	0
0	0	9	0	7	0	0
1	0	0	0	18	0	16
6	1	18	28	60	92	1
75	12	91	59	120	45	29
23	26	23	7	0	3	3
64	71	73	5	5	0	3
0	0	22	10	44	6	24
71	0	0	7	0	0	0
1	1	25	3	45	0	15
6	0	7	0	0	0	13
0	1	5	19	24	31	1
0	0	116	1	9	0	9
0	0	1	0	0	0	0
0	0	19	0	0	0	0
11	1	67	0	10	0	3
16	0	2	3	17	1	8
7	3	20	1	78	0	44
0	0	0	0	13	0	0
0	0	2	1	34	0	11
6	0	26	0	4	0	3
0	0	0	0	0	0	0
0	0	36	0	4	0	5
0	0	1	0	0	0	0
0	0	1	5	29	0	0
0	0	1	0	0	0	0
81	0	3	0	1	0	1
0	0	0	0	23	0	24
0	0	5	0	11	0	0

2	0	0	0	0	0	0
1	0	11	0	6	0	7
3	3	4	0	0	1	2
0	0	0	4	0	0	0
0	0	0	0	0	0	0
0	0	1	0	0	0	5
0	0	0	0	0	0	0
31	0	0	0	0	0	0
3	2	12	0	0	2	0
5	0	3	0	10	0	1
0	2	0	0	0	3	1
18	0	0	0	0	0	0
32	0	0	0	0	0	0
0	0	4	0	0	0	0
4	0	19	0	0	0	0
11	0	0	0	0	0	0
0	0	4	0	0	0	0
15	0	0	0	0	0	3
0	0	0	0	0	0	0
0	0	0	0	0	0	10
6	0	0	0	0	0	0
0	0	0	0	0	0	4
6	0	0	0	0	0	0
0	0	0	0	0	0	0
6	0	0	0	0	0	0
4	0	4	0	0	0	0
0	0	1	0	0	0	0
0	0	0	0	7	0	1
0	0	0	0	2	0	4
0	0	0	0	0	0	0
0	0	2	0	0	0	0
5	0	0	0	0	0	0
1	0	0	0	0	0	0
0	0	0	0	0	0	0
1	2	1	0	0	0	0
0	0	0	0	0	0	0
0	0	0	0	0	0	0
0	0	0	0	0	0	0
0	0	0	0	0	1	0
5	0	0	0	1	0	0
2	0	0	0	0	0	0
0	0	4	0	0	0	0
0	0	0	0	0	0	0
0	0	0	0	0	0	0
4	9	38	35	24	231	20
2	1	8	0	5	0	0
0	12	1	30	42	20	33
0	0	0	1	0	6	0

31	0	0	0	0	0	0
0	0	0	0	0	0	1
0	0	0	0	0	0	0
4	35	16	8	4	5	3
0	0	0	0	0	0	0
1	0	0	0	0	17	0
0	0	0	0	0	0	0
0	0	0	0	0	0	0
122	111	171	21	57	389	86
1718	1446	1238	1399	2957	3563	1549
57	10	2	27	1	90	12
7	7	1	1	0	121	16
61	150	155	158	34	212	37
18	1	1	14	0	231	5
9	0	2	0	7	0	27
25	3	7	14	5	10	9
22	38	59	52	3	188	85
4	0	0	9	3	211	5
0	7	3	0	0	0	0
39	6	8	0	13	4	19
7	3	6	6	0	1	2
4	9	16	1	5	2	1
0	4	7	15	1	4	0
0	0	0	0	0	0	0
0	8	5	1	0	2	0
2	0	1	0	2	3	8
1	0	0	1	0	0	0
18	4	5	2	5	7	2
3	0	1	1	5	1	1
0	0	0	0	0	0	0
2	1	0	1	0	9	0
0	1	0	0	0	0	0
2	1	1	4	7	2	5
0	0	0	1	20	0	5
0	0	0	0	0	0	0
0	0	0	0	0	0	0
3	0	1	2	1	0	0
3	0	0	3	5	0	2
0	0	2	0	0	0	0
56	0	0	1	0	0	0
0	0	0	0	0	0	1
0	0	0	0	0	0	0
0	1	22	0	0	0	0
0	0	0	0	0	0	0
1	0	0	0	0	0	0
0	0	0	0	0	4	0
0	0	0	0	0	2	0
0	0	0	6	8	0	0

0	0	0	0	0	0	0
10	0	2	0	4	4	2
0	0	0	0	0	0	0
2	0	0	0	0	0	0
0	0	0	0	0	0	0
1	1	3	1	0	5	0
0	0	0	0	0	0	0
6	6	4	5	4	5	3
0	0	0	0	0	0	0
0	0	0	0	0	0	0
0	0	0	0	1	0	1
1	1	1	0	0	2	0
0	0	0	0	0	0	0
1	1	0	0	1	1	0
0	0	0	0	0	0	0
2	1	1	0	1	0	1
0	0	0	0	0	0	0
0	0	0	0	0	0	0
1	0	1	0	0	0	0
0	2	1	0	0	0	1
0	0	0	0	0	0	0
0	0	0	0	0	0	0
1	0	2	0	1	2	0
0	0	0	0	2	1	1
0	0	0	0	0	0	0
0	0	0	0	0	0	0
0	0	0	0	0	1	0
0	0	0	0	0	0	0
0	0	0	0	0	0	0
1	0	0	0	0	0	0
0	0	0	0	0	0	0
0	0	0	0	2	0	1
1	0	0	0	0	0	0
0	0	0	0	0	0	0
0	0	0	0	0	0	0
0	0	0	0	0	0	0
0	0	0	0	0	0	0
0	0	0	0	0	0	0
0	0	0	0	0	0	0
0	0	0	0	0	0	0
0	0	0	0	0	0	0
0	0	0	0	1	0	4
1	1	1	0	0	1	2
0	0	0	0	0	0	0
1	0	1	0	1	0	0
0	0	0	0	0	0	0
0	3	0	0	0	0	0
0	0	0	0	0	0	0

0	0	0	0	0	0	0
105	135	117	346	88	378	34
59	21	98	35	113	38	80
26	36	31	58	15	200	13
11	81	74	90	5	221	12
33	16	25	4	6	2	0
30	35	15	15	3	4	0
22	5	0	0	0	59	4
5	5	8	4	1	11	4
30	12	44	19	48	11	47
0	1	5	41	11	6	1
0	6	10	1	3	0	4
0	1	0	1	0	0	1
0	0	0	0	0	0	0
0	0	0	0	1	0	0
0	0	0	0	0	0	0
3	4	6	4	4	8	1
5	0	1	2	3	1	0
0	0	1	0	0	0	0
1	0	0	0	1	0	0
0	0	1	0	4	0	14
0	0	0	1	0	1	0
0	0	1	4	0	1	2
0	0	1	0	0	0	0
0	0	0	0	0	0	0
0	0	0	0	0	0	1
0	0	0	0	0	0	0
5	0	0	1	0	0	0
0	1	0	1	0	0	0
0	0	0	0	0	0	0
0	0	0	0	0	0	0
0	0	0	0	0	0	0
0	0	0	0	0	0	0
0	1	0	0	0	0	0
0	0	0	0	0	0	0
0	0	0	0	0	0	0
59	113	65	293	5	91	1
7	15	17	131	44	38	7
4	5	4	155	27	30	0
0	0	0	0	0	0	0
7	3	5	53	0	10	0
0	0	0	1	144	0	90
8	0	0	0	0	0	0
12	3	4	0	0	1	0
0	0	0	0	0	0	0
0	0	0	0	0	0	0
0	0	0	0	0	0	0
0	0	0	0	0	0	0

0	0	0	0	0	0	0
3	0	0	0	0	0	0
0	0	0	0	0	0	0
0	0	0	0	0	0	0
0	0	0	0	0	0	0
0	0	0	0	0	0	0
0	0	0	0	0	0	0
0	0	0	0	0	0	0
0	0	0	0	0	0	0
0	0	0	0	0	0	0
0	0	0	0	0	0	0
3	0	0	0	0	0	0
0	0	0	0	0	0	0
0	0	0	0	0	0	0
0	0	0	0	0	0	0
0	0	0	0	0	0	0
0	0	0	0	0	0	0
0	5	7	33	11	0	2
0	0	0	0	0	3	0
12	0	0	0	0	0	25
0	0	0	0	0	0	0
0	2	72	6	53	1	103
0	0	0	0	0	0	0
0	0	0	0	0	0	0
0	0	0	0	0	0	5
0	0	0	0	0	0	0
0	0	0	0	0	0	0
0	0	0	0	0	0	0
0	0	0	1	0	596	28
7	1	5	6	0	0	0
0	0	0	0	0	0	0
0	0	0	0	12	0	8
0	0	0	0	0	0	0
39	40	32	113	16	779	263
0	0	0	0	0	2	0
0	0	0	0	0	0	0
2	1	3	2	0	0	0
0	6	16	0	0	0	0
0	0	0	0	0	0	0
0	0	0	0	0	0	0
0	0	0	0	0	0	0
0	0	0	0	0	0	0
11	10	9	77	0	28	2
0	0	11	0	0	0	0
20	2	4	12	11	63	11
0	0	0	0	0	0	0
0	0	0	0	0	0	0
6	6	12	11	6	20	13

0	0	0	0	0	0	0
114	26	33	198	36	1027	281
2	10	6	0	10	18	0
0	0	0	0	0	0	0
0	0	0	0	0	0	0
1	0	0	0	0	1	0
13	0	0	0	0	0	9
0	0	1	0	0	0	0
0	0	0	0	6	0	0
124	67	66	37	15	182	1
93	73	99	201	18	19	6
0	9	4	5	14	1	5
0	0	6	0	0	0	5
0	0	1	0	0	0	2
0	0	0	0	0	0	0
0	0	19	0	11	0	6
0	0	0	0	3	2	6
0	0	9	0	10	0	2
0	0	2	0	0	0	3
0	0	2	0	0	0	1
0	0	2	0	0	0	0
0	0	0	0	0	0	0
0	0	0	0	0	0	0
0	2	0	0	0	0	0
0	0	0	0	0	0	0
0	0	0	0	0	0	0
0	0	0	0	0	0	0
0	0	0	0	0	0	0
0	0	0	0	0	0	0
0	11	12	0	0	0	0
0	0	0	0	0	0	0
0	0	0	0	0	0	0
0	0	0	0	0	0	0
6	0	0	0	0	0	0
2	17	11	29	1	132	1
0	0	20	5	0	0	0
0	0	0	9	0	0	0
0	0	0	0	0	0	0
0	0	0	0	0	0	0
0	0	0	0	0	0	1
50	16	34	13	30	13	7
0	16	10	31	11	0	6
0	0	0	0	0	0	0
1	0	3	0	0	0	0
0	0	0	0	0	0	0
0	0	0	0	0	0	0
0	0	0	0	0	0	0
0	0	0	0	0	0	3

19	0	55	1	47	0	46
0	0	0	0	0	0	0
3	0	0	0	0	0	0
3	185	388	19	11	0	9
0	0	0	0	0	5	0
0	0	0	0	0	0	0
32	28	9	6	18	37	18
0	0	0	0	0	0	0
0	0	1	0	1	0	2
0	0	0	6	0	0	0
3	0	1	0	0	0	3
3	0	0	0	0	0	8
2	1	20	1	13	0	24
0	0	0	0	0	0	0
222	52	2448	11	154	0	400
1	0	5	0	1	0	5
0	0	0	0	0	0	1
0	0	0	0	0	0	0
0	0	3	0	0	0	5
0	0	0	0	13	0	4
0	0	0	0	0	0	0
0	0	0	0	0	0	0
1	0	21	0	0	0	2
0	0	0	0	0	0	0
0	0	1	0	0	0	0
2	0	2	0	0	0	0
0	0	2	0	4	0	6

124.SUR	124.DCM	125.SUR	125.DCM
0	0	0	0
0	39	0	8
0	6	0	0
0	6	0	2
0	0	0	0
0	3	0	0
0	0	0	0
0	24	11	11
0	7	0	2
0	0	0	0
0	2	0	2
0	0	0	0
0	0	0	0
0	0	0	10
0	0	0	0
3	0	1	0
4	408	3	114
726	165	557	59
0	3	518	0
12	2	4	10
13	5	0	0
0	0	0	0
0	0	0	23
0	0	0	0
0	0	0	0
0	0	0	0
0	0	0	0
0	0	0	0
0	0	0	0
0	0	0	0
0	0	0	0
0	0	0	0
7	8	0	4
7	0	0	0
0	0	0	0
0	0	0	0
5	586	30	631
887	51	100	0
195	124	56	28
0	0	0	0
1	142	0	13
0	0	0	0
365	160	13	0
7	3	160	19
0	12	0	32
0	0	0	2
2	12	0	9

0	0	0	4
0	0	0	0
0	45	0	79
1	45	0	15
0	15	0	0
0	21	0	1
0	4	0	0
0	0	7	3
0	0	0	0
0	0	0	106
3	0	3	0
0	0	0	0
0	2	0	0
0	0	0	0
0	9	0	0
0	0	0	0
0	0	0	6
0	3	0	0
0	0	0	0
0	0	0	0
0	0	0	2
0	0	0	0
0	0	0	0
0	0	0	0
0	0	0	0
0	0	0	0
0	0	1	0
0	0	0	1
0	0	0	0
0	2	0	5
0	4	0	1
0	0	0	0
0	0	0	0
0	0	0	0
0	0	0	0
0	0	0	0
0	0	0	0
0	0	0	0
0	0	0	0
125	220	287	441
0	0	10	2
8	6	26	0
2	295	0	133
16	1	0	1
0	0	0	2
0	0	0	7
0	0	0	0
0	0	0	0

0	0	0	1
0	0	0	0
0	0	0	0
0	0	0	0
0	0	0	0
0	0	0	0
0	0	0	0
0	0	0	0
0	0	0	0
0	0	0	0
0	0	0	0
0	0	0	0
0	0	0	0
0	0	0	0
1466	3	21	4
0	0	0	0
0	0	0	0
0	0	0	0
0	0	0	0
52	0	0	0
0	0	0	0
0	1	0	0
0	2	0	3
1	0	0	3
0	0	0	8
45	21	4	0
3	3	0	0
54	0	1	0
0	0	0	0
0	1	0	0
0	0	0	0
0	0	0	0
0	0	0	0
0	1	0	4
0	9	0	6
0	2	0	3
43	0	0	0
0	28	0	0
0	0	0	0
0	3	0	1
0	0	0	0
0	2	0	0
0	0	0	0
0	0	0	0
0	0	0	0
0	0	0	0
0	0	0	7
0	0	0	0

0	7	0	0
0	0	0	0
12	53	8	0
0	0	0	6
0	0	0	0
0	0	0	0
0	0	0	0
0	0	0	0
0	0	0	1
60	0	0	0
0	0	0	0
0	0	0	0
18	72	169	214
0	0	0	0
3	19	0	6
5	64	4	0
0	4	0	0
0	0	0	0
11	0	0	0
0	0	0	0
0	0	0	0
0	12	0	0
0	0	2	0
0	0	0	0
1	0	0	0
0	3	0	0
0	0	0	0
0	0	0	0
0	0	0	67
1	0	0	1
0	0	0	0
2	0	0	147
0	0	0	0
0	10	0	2
0	0	0	0
0	0	0	0
0	0	0	0
0	0	0	0
0	0	7	0
2	0	0	6
0	0	0	4
0	0	0	0
2	3	0	0
0	34	1	80
0	5	0	23
0	0	0	0
0	12	3	9
0	4	0	27

0	3	0	26
1	2	5	46
0	0	0	0
0	0	0	0
0	0	0	0
0	2	0	5
1	19	0	1
0	0	0	1
0	0	6	0
0	0	0	23
0	0	0	0
0	0	0	0
0	0	0	0
0	1	0	0
0	0	0	0
0	0	0	0
0	0	0	0
0	1	0	1
0	0	1	0
7	39	0	3
0	52	0	27
0	0	0	0
0	0	0	0
1109	3342	708	3429
49	11	18	70
43	0	0	32
0	0	0	0
0	0	0	0
0	0	0	0
0	1	0	0
0	0	0	0
0	0	0	2
0	0	0	0
0	0	0	0
0	0	0	0
0	0	0	0
66	171	21	309
33	23	4	28
10	0	2	12
17	21	31	42
0	44	0	4
0	1	0	0
0	0	0	0
0	0	0	0
0	1	0	3
0	0	1	29
0	0	0	0
0	1	0	1

0	1	0	0
0	4	0	0
0	0	0	0
0	0	0	50
0	0	0	0
0	0	0	0
0	0	0	0
0	0	0	1
0	0	0	4
0	0	0	1
0	0	0	0
0	0	0	0
0	0	0	0
0	0	0	0
0	0	0	1
0	0	0	0
0	0	0	0
0	0	0	0
0	0	0	0
5	0	0	0
0	0	0	0
2	3	271	3
1	1	0	0
262	2971	116	245
6	0	24	0
2	10	2	4
0	2	2	1
2	0	0	0
0	0	0	0
0	1	0	0
0	0	0	0
0	0	0	0
0	2	0	0
0	12	0	0
0	0	0	0
0	0	0	0
0	0	0	0
0	0	0	31
0	1	0	2
0	6	0	4
0	5	0	3
0	0	0	0
0	0	0	0
0	0	0	0
0	0	0	0
0	0	0	0
0	2	0	3
0	0	0	3

0	3	0	10
0	1	0	1
0	0	0	0
0	0	84	170
0	0	0	0
5	0	29	0
200	7	37	293
0	0	0	0
0	0	0	1
0	0	0	0
0	0	0	0
1982	4356	3452	519
517	104	97	2
153	483	311	26
273	266	291	28
368	102	162	32
142	176	22	1
24	38	9	12
3	16	3	11
3	177	1	22
0	66	2	107
24	293	98	68
0	0	0	1
22	57	2	6
0	21	0	8
0	0	0	86
0	0	0	8
0	0	0	2
0	0	0	0
2	24	0	0
0	71	0	19
0	0	0	0
4	4	0	0
0	2	0	1
0	0	0	8
0	0	0	0
64	81	20	21
0	5	0	88
1	36	0	47
0	0	0	0
0	90	0	15
2	1	6	0
9	13	0	16
0	0	0	116
0	0	0	0
0	11	0	11
2	15	0	50
0	0	0	77

0	7	0	49
0	18	0	27
0	8	0	37
0	0	0	0
0	0	0	0
0	0	0	0
0	0	0	0
1	0	5	1
0	1	0	1
0	0	0	0
0	0	0	11
0	0	0	0
1	0	0	0
0	0	0	2
0	0	12	0
0	15	0	19
0	0	0	0
0	2	0	0
0	0	0	0
0	0	0	30
0	0	0	0
0	7	2	3
0	0	0	0
0	0	0	0
0	6	0	1
1	0	0	30
0	0	0	0
0	0	0	0
0	0	0	0
0	0	0	0
17	1	4	4
0	1	0	0
8	4	2	0
2	4	3	3
0	3	0	0
0	5	1	2
5	2	19	0
0	0	0	7
0	0	0	0
0	1	0	2
0	0	0	0
0	0	0	0
0	0	0	0
0	3	0	0
0	0	0	0
0	0	0	0
0	0	0	0
0	0	0	0

0	4	0	1
1	0	0	0
0	0	0	0
0	0	1	0
0	0	0	0
0	0	0	0
0	0	0	4
0	0	0	0
0	0	0	0
1	1	0	0
0	3	0	3
0	0	0	0
0	0	0	0
0	1	0	1
0	0	0	0
0	0	0	0
0	0	0	0
0	0	0	0
1	0	0	0
0	0	0	0
0	0	0	0
0	0	0	0
0	0	0	0
0	0	0	0
0	1	0	0
0	0	0	3
0	0	0	0
0	0	0	0
0	0	0	0
1	19	0	84
0	105	0	0
0	6	0	3
0	0	0	0
0	0	0	0
0	0	0	0
57	94	52	5
0	78	5	339
0	295	0	1395
0	41	0	81
0	6	0	92
0	0	0	0
0	0	0	8
3	88	0	7
0	2	2	0
0	2	1	0
0	0	0	0
0	0	0	0
0	0	0	0

10	64	8	8
1	147	0	45
0	97	0	14
0	9	2	1
0	1	0	0
0	0	0	0
118	9	321	557
0	20	0	20
32	207	4	83
1	28	6	338
0	39	0	13
2	0	2	0
4	20	0	2
0	2	0	8
0	0	0	0
0	0	0	0
0	1	0	0
0	0	0	0
18	197	41	196
0	0	0	0
0	0	0	2
0	9	0	36
0	0	0	2
0	0	0	0
0	0	0	2
0	0	0	9
0	0	4	2
0	0	0	0
0	0	0	0
0	0	0	0
0	0	0	0
0	0	0	0
0	0	0	0
1	0	0	0
1	0	0	0
0	0	0	0
0	0	0	0
0	0	0	0
0	0	0	0
0	0	0	0
0	0	0	0
0	0	0	0
0	5	0	18
0	0	0	0
0	0	0	0
0	0	0	0
0	0	0	0
0	0	0	0

0	0	0	0
0	0	0	0
70	345	39	102
0	0	0	3
0	0	0	0
0	0	0	0
0	0	0	0
0	0	0	0
0	0	0	0
0	0	0	0
0	7	0	146
0	0	0	0
0	0	0	0
0	0	0	0
8	0	0	0
0	0	0	0
0	0	0	0
0	0	0	0
0	0	0	0
0	0	2	0
0	0	0	0
0	0	0	0
0	0	0	0
0	0	0	0
0	1	2	0
0	0	0	1
0	0	0	1
0	0	0	0
2	0	1	0
1	0	1	0
0	0	0	0
129	2	24	0
0	0	0	0
0	0	0	0
0	0	0	0
0	0	0	0
4	0	0	0
0	0	0	0
0	0	0	0
0	0	0	0
154	0	2	0
0	0	0	0
0	0	1	0
3032	198	1016	454
2	0	4	3
13	9	3	92
89	125	27	57
32	24	111	98

11	10	9	0
94	5	41	19
14	0	0	0
3	0	5	4
3	3	2	5
12	8	10	8
13	0	7	0
0	16	0	13
44	0	8	0
0	0	0	0
8	4	0	2
3	6	3	3
0	0	0	0
3	0	1	0
1	0	2	0
9	2	3	0
0	0	0	0
4	15	16	9
5	0	4	40
0	0	1	0
0	0	0	0
4	0	3	0
1	96	0	0
0	0	0	0
0	0	0	0
3	7	7	9
1	0	0	3
0	0	0	0
0	0	0	5
0	0	0	0
0	2	0	0
0	0	0	4
0	0	0	5
0	0	0	0
0	0	0	0
1	3	0	0
0	2	0	0
0	0	0	0
0	0	2	0
0	0	0	0
0	0	0	0
9	0	0	0
0	0	0	0
0	0	0	0
0	0	0	0
0	2	0	0
0	0	0	0
0	1	0	0

0	0	0	0
0	0	0	0
0	0	0	0
0	0	0	0
1	0	0	0
12	0	4	4
0	0	0	0
451	662	241	165
0	1	0	0
0	0	0	0
0	3	0	1
0	0	0	0
0	6	0	0
0	0	0	0
0	0	0	18
0	0	0	0
0	0	0	0
187	85	0	8
0	0	1	3
0	0	0	1
0	0	0	0
0	0	2	2
0	0	0	0
0	0	0	0
0	0	0	0
0	0	0	0
0	0	0	0
0	0	0	0
0	0	0	0
0	0	0	1
48	3	31	0
0	0	1	0
0	0	0	0
0	0	0	0
0	0	0	0
0	8	0	15
281	2	271	367
0	0	1	0
0	0	1	0
0	0	0	0
0	0	0	0
1	0	1	0
0	0	0	0
0	0	0	0
208	0	3	0
0	0	0	0
0	0	1	0
0	0	0	0

0	0	0	0
0	0	0	0
0	0	0	0
0	0	0	0
0	0	0	0
0	0	0	0
0	0	3	7
0	0	0	0
0	0	0	0
4002	14	3	0
1	0	0	0
0	0	0	0
0	0	0	0
0	0	0	0
12	0	12	17
402	5	49	7
0	0	0	0
0	0	1	0
117	1	33	3
204	1	9	0
0	0	0	0
54	0	8	0
8	4	0	0
2	0	8	0
18	0	33	9
0	0	2	4
1	0	2	0
14	0	10	0
69	1	0	0
0	0	1	0
0	0	0	0
0	5	0	1
0	0	0	0
0	0	0	0
0	0	0	0
0	0	0	0
13	0	0	0
0	0	0	0
0	0	0	0
0	4	0	1
0	1	0	0
0	0	0	0
0	0	0	0
0	0	0	0
0	0	0	0
0	0	0	0
0	0	0	0
0	0	0	0

3	0	0	0
4778	13	513	51
369	1	48	12
173	1	34	5
0	0	0	0
0	0	0	0
8	0	2	0
1	0	0	0
0	0	0	0
113	0	47	0
0	0	0	0
0	0	0	0
0	0	0	0
0	0	1	0
9	0	2	0
0	1	0	1
0	0	0	0
0	0	0	0
0	0	0	0
12	0	0	0
1	0	4	1
0	5	0	2
0	0	0	0
1	0	0	0
0	0	0	0
0	0	0	1
0	0	0	0
0	0	0	0
0	0	0	0
0	0	0	0
0	0	0	0
0	0	0	0
1	0	0	0
307	0	40	0
627	2	26	3
923	19	138	85
552	0	262	43
0	0	2	0
14	7	48	89
93	20	16	39
81	3	5	0
175	0	54	1
0	0	0	4
20	11	1	28
5	0	0	0
54	7	2	0
0	0	0	0
1	3	0	29

0	0	0	0
0	0	0	0
0	0	0	0
0	0	0	0
0	0	0	0
0	0	0	6
0	0	0	0
0	0	0	0
0	0	0	0
0	0	0	0
0	0	0	0
0	0	0	0
0	0	0	0
0	0	0	0
0	0	0	0
0	0	0	1
0	0	0	0
0	0	0	0
0	11	0	0
0	3	0	0
0	0	0	0
0	0	0	0
5	0	1	0
0	0	0	0
0	0	0	0
0	0	0	0
1	0	0	0
0	2	0	0
0	0	0	0
2	0	1	5
0	0	0	5
0	0	0	1
0	0	0	0
0	0	0	0
0	0	0	0
0	0	0	0
0	0	0	0
0	0	0	0
0	0	0	0
0	0	0	0
0	0	1	0
6	0	5	11
0	0	0	0
0	0	0	1
0	0	0	0
0	0	0	0
0	0	0	0
0	0	0	0

1792	78	671	1802
1542	29	102	65
0	0	0	0
0	0	0	0
0	3	0	0
1	0	0	2
0	0	0	0
0	0	0	0
0	0	0	0
0	0	0	0
0	0	0	0
0	0	0	0
20	0	14	44
0	0	0	2
770	2	96	104
0	0	0	0
0	0	0	0
0	0	0	0
0	0	0	0
11	1	4	3
1	0	0	1
0	0	0	0
0	0	0	0
0	0	0	0
5509	21	60	16
0	0	0	0
0	0	0	0
26	2	36	81
41	1	18	20
1281	12	138	35
1257	1	81	21
131	1	8	3
351	7	58	88
5	0	0	0
125	1	57	13
39	0	4	0
1	0	2	3
0	0	0	0
0	0	0	0
0	0	0	0
0	0	0	0
0	0	0	0
0	0	0	0
65	1	131	116
129	1	23	44
0	0	0	0
311	1	9	1
0	0	1	0

0	0	0	0
1	0	0	0
0	0	0	0
0	0	1	0
0	0	0	0
0	0	0	0
0	0	0	0
4	0	0	0
0	0	0	0
35	39	4	53
0	3	0	3
0	0	0	0
0	0	0	0
0	0	0	0
0	0	0	0
631	2	177	81
202	4	133	91
27	0	2	1
12	0	6	3
3	0	0	0
2	0	2	0
0	0	0	0
0	0	0	0
2	0	0	0
1	0	0	0
10	0	2	0
1	0	0	0
0	0	0	0
205	0	1	0
89	0	12	7
61	0	1	1
66	2	37	26
253	0	47	2
0	0	0	0
0	0	0	0
0	0	0	0
0	0	0	0
2	0	1	0
1877	239	172	1046
52	34	5	151
208	44	13	90
13	33	4	174
41	6	1	8
0	4	0	1
0	19	0	4
0	0	0	2
0	0	0	0
0	0	0	0

0	0	0	0
0	0	0	0
0	0	0	0
0	0	0	0
0	0	0	0
0	0	0	0
0	0	0	0
0	0	0	2
0	0	0	1
0	0	0	1
0	0	0	0
0	0	0	0
0	0	0	0
0	0	0	0
6	0	0	0
0	0	2	0
22	1	2	0
0	0	0	0
5	0	0	1
129	6	33	0
6	0	5	0
1	0	0	0
0	9	0	0
0	0	0	0
0	0	0	0
695	6	83	96
1	0	0	0
0	3	0	0
0	0	0	0
1	0	0	0
0	148	0	21
0	0	0	5
0	0	0	0
0	13	1	2
0	0	0	5
0	1	0	0
0	0	0	0
0	0	0	0
2	0	0	2
0	0	0	0
0	0	0	0
0	0	0	0
3	0	0	0
1	0	0	0
19	0	1	0
0	0	0	4
0	0	1	0
0	0	0	0

0	0	0	0
0	0	0	0
3642	226	1272	102
229	0	1079	120
1243	98	383	36
2	26	0	85
236	12	77	0
169	627	130	305
4	21	13	29
26	0	37	6
462	43	119	0
2	5	0	0
83	23	49	8
680	64	454	19
290	202	748	52
251	4	80	2
2	17	0	13
0	0	1	0
103	1	157	1
0	0	0	0
0	1	0	0
0	6	0	1
108	17	14	1
161	33	34	20
23	0	15	7
47	4	25	6
18	52	49	33
0	0	0	0
0	51	0	17
0	0	0	9
81	2	54	2
3	4	0	2
0	0	0	0
1	0	0	0
0	5	0	13
2	2	3	0
0	14	0	19
2	6	0	0
0	5	0	0
1	26	0	4
0	0	0	0
0	35	0	6
0	0	0	0
3	1	2	0
0	0	0	0
0	1	0	3
0	8	0	0
0	2	0	0

0	0	0	0
0	6	0	2
12	0	0	0
1	19	0	15
0	9	0	2
0	0	0	0
0	0	0	0
0	0	0	0
3	0	2	2
0	5	0	0
0	2	1	0
0	0	0	0
0	0	0	0
0	0	0	14
0	0	0	0
0	0	0	0
0	0	0	1
0	0	0	13
0	0	0	0
0	0	0	2
0	0	0	0
0	0	0	0
0	0	0	4
0	0	0	0
0	0	0	0
0	0	0	0
0	0	0	0
0	0	0	0
0	2	0	1
0	0	0	0
0	0	0	0
0	0	0	0
0	0	0	0
0	0	0	0
2	0	0	0
0	0	0	0
0	0	0	0
0	0	0	0
1	0	1	0
1	1	0	0
0	0	0	0
0	0	0	0
0	0	0	0
0	0	0	0
268	19	48	6
12	1	2	0
24	5	1	15
6	0	13	0

0	0	0	0
1	0	1	0
0	0	0	0
14	3	5	0
0	0	0	0
0	0	5	0
0	0	0	0
0	0	0	0
674	134	74	24
6787	619	2145	918
474	9	22	4
37	1	23	1
853	25	410	43
489	5	41	3
0	9	0	0
33	1	6	1
24	2	309	61
231	1	62	1
1	0	1	0
20	0	6	1
28	0	2	4
26	3	1	2
6	0	17	1
0	0	0	0
13	0	3	0
2	1	0	4
1	0	0	0
38	1	6	1
1	0	1	1
0	0	0	0
9	0	2	0
0	0	0	0
6	2	1	2
1	3	0	0
0	0	0	0
0	0	0	0
0	0	0	1
0	1	8	0
0	0	0	0
1	0	0	0
0	0	0	0
0	0	0	0
0	0	4	0
0	0	0	0
0	0	0	0
0	0	0	0
5	0	0	1
0	0	2	2

0	0	0	0
12	0	2	2
0	0	0	0
0	0	0	0
0	0	0	0
1	0	2	0
0	0	0	0
28	2	4	1
0	0	0	0
0	0	0	0
0	0	0	0
3	0	1	0
0	0	0	0
0	0	1	2
0	0	0	0
0	0	1	1
1	0	0	0
0	0	0	0
1	0	0	0
0	0	0	1
0	0	0	0
0	0	0	0
2	0	1	0
0	0	0	1
0	0	0	0
0	0	0	0
0	0	0	0
0	0	0	0
0	0	0	0
0	0	0	0
0	0	0	0
0	0	0	0
0	0	0	0
0	0	0	0
0	0	0	0
0	0	0	0
0	0	0	0
0	0	0	0
0	0	0	0
0	0	0	0
0	0	0	0
0	0	0	0
1	0	1	0
0	0	0	0
0	0	0	0
0	0	0	0
0	0	0	0
0	0	0	0

0	0	0	0
608	28	694	56
100	44	38	30
293	1	72	4
367	0	245	11
17	0	9	1
12	0	6	0
80	1	33	1
8	0	7	0
18	21	20	7
14	6	8	0
1	4	0	0
0	0	1	0
0	0	0	0
1	0	0	0
0	0	0	0
7	0	8	1
2	1	8	2
1	0	0	0
0	0	0	0
0	2	0	13
0	0	2	0
3	0	2	0
0	0	0	0
0	0	0	0
3	0	1	0
0	0	0	0
0	0	0	0
0	0	1	0
0	0	1	0
0	0	0	0
0	0	0	0
0	0	0	0
0	0	0	0
0	0	0	0
0	0	0	0
265	0	402	30
33	2	10	3
27	0	50	1
0	0	0	0
3	0	7	0
0	46	0	45
0	0	0	0
0	0	1	0
0	0	0	0
0	0	0	0
0	0	0	0
0	0	0	0

0	0	0	0
0	0	0	3
0	0	0	0
0	0	0	0
0	0	0	0
0	0	0	1
0	0	0	0
0	0	0	0
0	0	0	0
0	0	0	0
0	0	0	0
0	0	0	0
0	0	0	0
0	0	0	0
0	0	0	0
0	0	0	0
0	0	0	0
9	10	10	5
0	0	0	0
0	0	0	13
0	4	0	0
0	0	0	1
0	0	0	0
0	0	0	46
0	0	0	0
0	0	0	0
0	0	0	0
0	0	0	16
444	2	12	0
15	0	0	0
0	0	0	0
0	3	0	7
0	0	0	0
1495	8	323	77
6	0	0	0
0	0	1	0
6	0	4	0
0	0	0	0
0	0	0	0
0	0	0	0
0	0	0	0
0	0	0	0
398	1	53	1
0	0	0	0
141	0	92	17
0	0	0	0
0	0	0	0
271	3	46	7

0	0	0	3
2795	11	155	25
328	2	18	5
0	0	0	0
0	0	0	0
0	0	3	0
0	4	0	1
0	0	0	0
0	0	0	0
1560	21	110	10
824	9	81	21
6	5	0	0
0	10	0	1
0	0	0	0
0	0	0	0
0	8	0	2
13	7	0	0
0	0	0	1
0	0	0	0
0	5	0	0
0	0	0	0
0	0	0	0
0	0	0	0
0	0	0	0
0	0	0	0
0	0	0	0
0	0	0	0
0	0	0	0
0	0	0	0
0	0	0	0
0	0	0	0
0	0	1	0
0	0	0	0
0	0	0	0
31	2	0	4
2	0	0	0
0	0	0	0
0	0	0	0
0	0	0	0
0	0	0	0
52	10	122	26
1	1	0	9
0	0	0	53
0	0	0	11
14	0	2	2
0	0	0	0
0	0	2	0
0	0	0	6

0	15	0	26
0	0	0	0
0	0	0	0
5	2	2	5
4	0	0	0
0	0	0	0
17	9	20	10
0	0	0	0
0	1	0	0
0	0	0	0
0	1	0	3
0	0	0	1
1	4	0	13
0	0	0	0
1	112	0	247
0	10	0	15
0	0	0	3
0	0	0	0
0	6	0	0
0	0	0	0
0	0	0	0
0	0	0	0
0	0	0	0
0	0	0	0
0	0	0	0
0	0	0	0
0	0	0	2

Taxonomy
Eukaryota / Alveolata / Ciliophora / Intramacronucleata / Armophorea / Armophorida / Metopidae / Metopus
Eukaryota / Alveolata / Ciliophora / Intramacronucleata / Armophorea / Clevelandellida / Clevelandellidae / Cleve
Eukaryota / Alveolata / Ciliophora / Intramacronucleata / Armophorea / Clevelandellida / Clevelandellidae / Cleve
Eukaryota / Alveolata / Ciliophora / Intramacronucleata / Armophorea / Clevelandellida / Clevelandellidae / Cleve
Eukaryota / Alveolata / Ciliophora / Intramacronucleata / Armophorea / Clevelandellida / Clevelandellidae / Cleve
Eukaryota / Alveolata / Ciliophora / Intramacronucleata / Armophorea / Clevelandellida / Clevelandellidae / Cleve
Eukaryota / Alveolata / Ciliophora / Intramacronucleata / Armophorea / Clevelandellida / Clevelandellidae / Cleve
Eukaryota / Alveolata / Ciliophora / Intramacronucleata / Colpodea / Bryometopida / Bryometopidae / Bryometop
Eukaryota / Alveolata / Ciliophora / Intramacronucleata / Colpodea / Bryometopida / Bryometopidae / Bryometop
Eukaryota / Alveolata / Ciliophora / Intramacronucleata / Colpodea / Bryophryida / Bryophryidae / Bryophryoides
Eukaryota / Alveolata / Ciliophora / Intramacronucleata / Colpodea / Bryophryida / Bryophryidae / Bryophryoides
Eukaryota / Alveolata / Ciliophora / Intramacronucleata / Colpodea / Bryophryida / Bryophryidae / Bryophryoides
Eukaryota / Alveolata / Ciliophora / Intramacronucleata / Colpodea / Bryophryida / Bryophryidae / Bryophryoides
Eukaryota / Alveolata / Ciliophora / Intramacronucleata / Colpodea / Bursariomorphida / Bursariidae / Bursaria
Eukaryota / Alveolata / Ciliophora / Intramacronucleata / Colpodea / Bursariomorphida / Bursariidae / Bursaria
Eukaryota / Alveolata / Ciliophora / Intramacronucleata / Colpodea / Bursariomorphida / Bursariidae / Bursaria
Eukaryota / Alveolata / Ciliophora / Intramacronucleata / Colpodea / Colpodida / Colpodidae / Bresslaua
Eukaryota / Alveolata / Ciliophora / Intramacronucleata / Colpodea / Colpodida / Colpodidae / Colpoda
Eukaryota / Alveolata / Ciliophora / Intramacronucleata / Colpodea / Colpodida / Colpodidae / Colpoda
Eukaryota / Alveolata / Ciliophora / Intramacronucleata / Colpodea / Colpodida / Colpodidae / Colpoda
Eukaryota / Alveolata / Ciliophora / Intramacronucleata / Colpodea / Colpodida / Colpodidae / Colpoda
Eukaryota / Alveolata / Ciliophora / Intramacronucleata / Colpodea / Colpodida / Colpodidae / Colpoda
Eukaryota / Alveolata / Ciliophora / Intramacronucleata / Colpodea / Colpodida / Colpodidae / Colpoda
Eukaryota / Alveolata / Ciliophora / Intramacronucleata / Colpodea / Colpodida / Colpodidae / Colpoda
Eukaryota / Alveolata / Ciliophora / Intramacronucleata / Colpodea / Colpodida / Colpodidae / Colpoda
Eukaryota / Alveolata / Ciliophora / Intramacronucleata / Colpodea / Colpodida / Colpodidae / Colpoda
Eukaryota / Alveolata / Ciliophora / Intramacronucleata / Colpodea / Colpodida / Colpodidae / Colpoda
Eukaryota / Alveolata / Ciliophora / Intramacronucleata / Colpodea / Colpodida / Colpodidae / Colpoda
Eukaryota / Alveolata / Ciliophora / Intramacronucleata / Colpodea / Colpodida / Colpodidae / Colpoda
Eukaryota / Alveolata / Ciliophora / Intramacronucleata / Colpodea / Colpodida / Colpodidae / Colpoda
Eukaryota / Alveolata / Ciliophora / Intramacronucleata / Colpodea / Colpodida / Hausmanniellidae / Bresslauides
Eukaryota / Alveolata / Ciliophora / Intramacronucleata / Colpodea / Colpodida / Hausmanniellidae / Bresslauides
Eukaryota / Alveolata / Ciliophora / Intramacronucleata / Colpodea / Colpodida / Marynidae / Maryna
Eukaryota / Alveolata / Ciliophora / Intramacronucleata / Colpodea / Colpodida / Marynidae / Maryna
Eukaryota / Alveolata / Ciliophora / Intramacronucleata / Colpodea / Colpodida / Marynidae / Maryna
Eukaryota / Alveolata / Ciliophora / Intramacronucleata / Colpodea / Colpodida / Marynidae / Maryna
Eukaryota / Alveolata / Ciliophora / Intramacronucleata / Colpodea / Cyrtolophosidida / Cyrtolophosididae / Aristo
Eukaryota / Alveolata / Ciliophora / Intramacronucleata / Colpodea / Cyrtolophosidida / Cyrtolophosididae / Aristo
Eukaryota / Alveolata / Ciliophora / Intramacronucleata / Colpodea / Cyrtolophosidida / Cyrtolophosididae / Aristo
Eukaryota / Alveolata / Ciliophora / Intramacronucleata / Colpodea / Cyrtolophosidida / Cyrtolophosididae / Aristo
Eukaryota / Alveolata / Ciliophora / Intramacronucleata / Colpodea / Cyrtolophosidida / Cyrtolophosididae / Aristo
Eukaryota / Alveolata / Ciliophora / Intramacronucleata / Colpodea / Cyrtolophosidida / Cyrtolophosididae / Aristo
Eukaryota / Alveolata / Ciliophora / Intramacronucleata / Colpodea / Cyrtolophosidida / Cyrtolophosididae / Aristo
Eukaryota / Alveolata / Ciliophora / Intramacronucleata / Colpodea / Cyrtolophosidida / Cyrtolophosididae / Aristo
Eukaryota / Alveolata / Ciliophora / Intramacronucleata / Colpodea / Cyrtolophosidida / Cyrtolophosididae / Aristo
Eukaryota / Alveolata / Ciliophora / Intramacronucleata / Colpodea / Cyrtolophosidida / Cyrtolophosididae / Aristo
Eukaryota / Alveolata / Ciliophora / Intramacronucleata / Colpodea / Cyrtolophosidida / Cyrtolophosididae / Aristo

Eukaryota / Alveolata / Ciliophora / Intramacronucleata / Colpodea / Cyrtolophosidida / Cyrtolophosididae / Aristo
Eukaryota / Alveolata / Ciliophora / Intramacronucleata / Colpodea / Cyrtolophosidida / Cyrtolophosididae / Aristo
Eukaryota / Alveolata / Ciliophora / Intramacronucleata / Colpodea / Cyrtolophosidida / Cyrtolophosididae / Aristo
Eukaryota / Alveolata / Ciliophora / Intramacronucleata / Colpodea / Cyrtolophosidida / Cyrtolophosididae / Aristo
Eukaryota / Alveolata / Ciliophora / Intramacronucleata / Colpodea / Cyrtolophosidida / Cyrtolophosididae / Aristo
Eukaryota / Alveolata / Ciliophora / Intramacronucleata / Colpodea / Cyrtolophosidida / Cyrtolophosididae / Aristo
Eukaryota / Alveolata / Ciliophora / Intramacronucleata / Colpodea / Cyrtolophosidida / Cyrtolophosididae / Aristo
Eukaryota / Alveolata / Ciliophora / Intramacronucleata / Colpodea / Cyrtolophosidida / Cyrtolophosididae / Aristo
Eukaryota / Alveolata / Ciliophora / Intramacronucleata / Colpodea / Cyrtolophosidida / Cyrtolophosididae / Aristo
Eukaryota / Alveolata / Ciliophora / Intramacronucleata / Colpodea / Cyrtolophosidida / Cyrtolophosididae / Aristo
Eukaryota / Alveolata / Ciliophora / Intramacronucleata / Colpodea / Cyrtolophosidida / Cyrtolophosididae / Aristo
Eukaryota / Alveolata / Ciliophora / Intramacronucleata / Colpodea / Cyrtolophosidida / Cyrtolophosididae / Aristo
Eukaryota / Alveolata / Ciliophora / Intramacronucleata / Colpodea / Cyrtolophosidida / Cyrtolophosididae / Aristo
Eukaryota / Alveolata / Ciliophora / Intramacronucleata / Colpodea / Cyrtolophosidida / Cyrtolophosididae / Aristo
Eukaryota / Alveolata / Ciliophora / Intramacronucleata / Colpodea / Cyrtolophosidida / Cyrtolophosididae / Aristo
Eukaryota / Alveolata / Ciliophora / Intramacronucleata / Colpodea / Cyrtolophosidida / Cyrtolophosididae / Aristo
Eukaryota / Alveolata / Ciliophora / Intramacronucleata / Colpodea / Cyrtolophosidida / Cyrtolophosididae / Aristo
Eukaryota / Alveolata / Ciliophora / Intramacronucleata / Colpodea / Cyrtolophosidida / Cyrtolophosididae / Aristo
Eukaryota / Alveolata / Ciliophora / Intramacronucleata / Colpodea / Cyrtolophosidida / Cyrtolophosididae / Aristo
Eukaryota / Alveolata / Ciliophora / Intramacronucleata / Colpodea / Cyrtolophosidida / Cyrtolophosididae / Aristo
Eukaryota / Alveolata / Ciliophora / Intramacronucleata / Colpodea / Cyrtolophosidida / Cyrtolophosididae / Aristo
Eukaryota / Alveolata / Ciliophora / Intramacronucleata / Colpodea / Cyrtolophosidida / Cyrtolophosididae / Aristo
Eukaryota / Alveolata / Ciliophora / Intramacronucleata / Colpodea / Cyrtolophosidida / Cyrtolophosididae / Aristo
Eukaryota / Alveolata / Ciliophora / Intramacronucleata / Colpodea / Cyrtolophosidida / Cyrtolophosididae / Aristo
Eukaryota / Alveolata / Ciliophora / Intramacronucleata / Colpodea / Cyrtolophosidida / Cyrtolophosididae / Aristo
Eukaryota / Alveolata / Ciliophora / Intramacronucleata / Colpodea / Cyrtolophosidida / Cyrtolophosididae / Aristo
Eukaryota / Alveolata / Ciliophora / Intramacronucleata / Colpodea / Cyrtolophosidida / Cyrtolophosididae / Aristo
Eukaryota / Alveolata / Ciliophora / Intramacronucleata / Colpodea / Cyrtolophosidida / Cyrtolophosididae / Aristo
Eukaryota / Alveolata / Ciliophora / Intramacronucleata / Colpodea / Cyrtolophosidida / Cyrtolophosididae / Aristo
Eukaryota / Alveolata / Ciliophora / Intramacronucleata / Colpodea / Cyrtolophosidida / Cyrtolophosididae / Cyrto
Eukaryota / Alveolata / Ciliophora / Intramacronucleata / Colpodea / Cyrtolophosidida / Cyrtolophosididae / Cyrto
Eukaryota / Alveolata / Ciliophora / Intramacronucleata / Colpodea / Cyrtolophosidida / Cyrtolophosididae / Cyrto
Eukaryota / Alveolata / Ciliophora / Intramacronucleata / Colpodea / Cyrtolophosidida / Cyrtolophosididae / Pseu
Eukaryota / Alveolata / Ciliophora / Intramacronucleata / Colpodea / Cyrtolophosidida / Platyophryidae / Platyoph
Eukaryota / Alveolata / Ciliophora / Intramacronucleata / Colpodea / Cyrtolophosidida / Woodruffiidae / Kuklikop
Eukaryota / Alveolata / Ciliophora / Intramacronucleata / Colpodea / Grossglockneriida / Grossglockneriidae / Pse
Eukaryota / Alveolata / Ciliophora / Intramacronucleata / Colpodea / Grossglockneriida / Grossglockneriidae / Pse
Eukaryota / Alveolata / Ciliophora / Intramacronucleata / Colpodea / Sorogenida / Sorogenidae / Sorogena
Eukaryota / Alveolata / Ciliophora / Intramacronucleata / Colpodea / Sorogenida / Sorogenidae / Sorogena
Eukaryota / Alveolata / Ciliophora / Intramacronucleata / Litostomatea / Haptoria / Cyclotrichida / Mesodiniidae /
Eukaryota / Alveolata / Ciliophora / Intramacronucleata / Litostomatea / Haptoria / Cyclotrichida / Mesodiniidae /
Eukaryota / Alveolata / Ciliophora / Intramacronucleata / Litostomatea / Haptoria / Cyclotrichida / Mesodiniidae /
Eukaryota / Alveolata / Ciliophora / Intramacronucleata / Litostomatea / Haptoria / Cyclotrichida / Mesodiniidae /
Eukaryota / Alveolata / Ciliophora / Intramacronucleata / Litostomatea / Haptoria / Cyclotrichida / Mesodiniidae /
Eukaryota / Alveolata / Ciliophora / Intramacronucleata / Litostomatea / Haptoria / Cyclotrichida / Mesodiniidae /
Eukaryota / Alveolata / Ciliophora / Intramacronucleata / Litostomatea / Haptoria / Cyclotrichida / Mesodiniidae /
Eukaryota / Alveolata / Ciliophora / Intramacronucleata / Litostomatea / Haptoria / Cyclotrichida / Mesodiniidae /
Eukaryota / Alveolata / Ciliophora / Intramacronucleata / Litostomatea / Haptoria / Cyclotrichida / Mesodiniidae /

Eukaryota / Alveolata / Ciliophora / Intramacronucleata / Litostomatea / Haptoria / Cyclotrichida / Mesodiniidae /
Eukaryota / Alveolata / Ciliophora / Intramacronucleata / Litostomatea / Haptoria / Cyclotrichida / Mesodiniidae /
Eukaryota / Alveolata / Ciliophora / Intramacronucleata / Litostomatea / Haptoria / Cyclotrichida / Mesodiniidae /
Eukaryota / Alveolata / Ciliophora / Intramacronucleata / Litostomatea / Haptoria / Cyclotrichida / Mesodiniidae /
Eukaryota / Alveolata / Ciliophora / Intramacronucleata / Litostomatea / Haptoria / Cyclotrichida / Mesodiniidae /
Eukaryota / Alveolata / Ciliophora / Intramacronucleata / Litostomatea / Haptoria / Cyclotrichida / Mesodiniidae /
Eukaryota / Alveolata / Ciliophora / Intramacronucleata / Litostomatea / Haptoria / Cyclotrichida / Mesodiniidae /
Eukaryota / Alveolata / Ciliophora / Intramacronucleata / Litostomatea / Haptoria / Cyclotrichida / Mesodiniidae /
Eukaryota / Alveolata / Ciliophora / Intramacronucleata / Litostomatea / Haptoria / Cyclotrichida / Mesodiniidae /
Eukaryota / Alveolata / Ciliophora / Intramacronucleata / Litostomatea / Haptoria / Cyclotrichida / Mesodiniidae /
Eukaryota / Alveolata / Ciliophora / Intramacronucleata / Litostomatea / Haptoria / Cyclotrichida / Mesodiniidae /
Eukaryota / Alveolata / Ciliophora / Intramacronucleata / Litostomatea / Haptoria / Cyclotrichida / Mesodiniidae /
Eukaryota / Alveolata / Ciliophora / Intramacronucleata / Litostomatea / Haptoria / Cyclotrichida / Mesodiniidae /
Eukaryota / Alveolata / Ciliophora / Intramacronucleata / Litostomatea / Haptoria / Haptorida / Acropisthiidae / F
Eukaryota / Alveolata / Ciliophora / Intramacronucleata / Litostomatea / Haptoria / Haptorida / Didiniidae / Cyclot
Eukaryota / Alveolata / Ciliophora / Intramacronucleata / Litostomatea / Haptoria / Haptorida / Didiniidae / Cyclot
Eukaryota / Alveolata / Ciliophora / Intramacronucleata / Litostomatea / Haptoria / Haptorida / Didiniidae / Cyclot
Eukaryota / Alveolata / Ciliophora / Intramacronucleata / Litostomatea / Haptoria / Haptorida / Didiniidae / Cyclot
Eukaryota / Alveolata / Ciliophora / Intramacronucleata / Litostomatea / Haptoria / Haptorida / Didiniidae / Cyclot
Eukaryota / Alveolata / Ciliophora / Intramacronucleata / Litostomatea / Haptoria / Haptorida / Didiniidae / Didini
Eukaryota / Alveolata / Ciliophora / Intramacronucleata / Litostomatea / Haptoria / Haptorida / Didiniidae / Didini
Eukaryota / Alveolata / Ciliophora / Intramacronucleata / Litostomatea / Haptoria / Haptorida / Didiniidae / Monc
Eukaryota / Alveolata / Ciliophora / Intramacronucleata / Litostomatea / Haptoria / Haptorida / Enchelyidae / Enc
Eukaryota / Alveolata / Ciliophora / Intramacronucleata / Litostomatea / Haptoria / Haptorida / Enchelyidae / Enc
Eukaryota / Alveolata / Ciliophora / Intramacronucleata / Litostomatea / Haptoria / Haptorida / Enchelyidae / Enc
Eukaryota / Alveolata / Ciliophora / Intramacronucleata / Litostomatea / Haptoria / Haptorida / Paraspathidiidae
Eukaryota / Alveolata / Ciliophora / Intramacronucleata / Litostomatea / Haptoria / Haptorida / Paraspathidiidae
Eukaryota / Alveolata / Ciliophora / Intramacronucleata / Litostomatea / Haptoria / Haptorida / Paraspathidiidae
Eukaryota / Alveolata / Ciliophora / Intramacronucleata / Litostomatea / Haptoria / Haptorida / Paraspathidiidae
Eukaryota / Alveolata / Ciliophora / Intramacronucleata / Litostomatea / Haptoria / Haptorida / Paraspathidiidae
Eukaryota / Alveolata / Ciliophora / Intramacronucleata / Litostomatea / Haptoria / Haptorida / Spathidiidae / Arc
Eukaryota / Alveolata / Ciliophora / Intramacronucleata / Litostomatea / Haptoria / Haptorida / Spathidiidae / Arc
Eukaryota / Alveolata / Ciliophora / Intramacronucleata / Litostomatea / Haptoria / Haptorida / Spathidiidae / Bal
Eukaryota / Alveolata / Ciliophora / Intramacronucleata / Litostomatea / Haptoria / Haptorida / Spathidiidae / Bal
Eukaryota / Alveolata / Ciliophora / Intramacronucleata / Litostomatea / Haptoria / Haptorida / Spathidiidae / Bal
Eukaryota / Alveolata / Ciliophora / Intramacronucleata / Litostomatea / Haptoria / Haptorida / Spathidiidae / Bal
Eukaryota / Alveolata / Ciliophora / Intramacronucleata / Litostomatea / Haptoria / Haptorida / Spathidiidae / Spa
Eukaryota / Alveolata / Ciliophora / Intramacronucleata / Litostomatea / Haptoria / Haptorida / Spathidiidae / Spa
Eukaryota / Alveolata / Ciliophora / Intramacronucleata / Litostomatea / Haptoria / Haptorida / Spathidiidae / Spa
Eukaryota / Alveolata / Ciliophora / Intramacronucleata / Litostomatea / Haptoria / Haptorida / Spathidiidae / Teu
Eukaryota / Alveolata / Ciliophora / Intramacronucleata / Litostomatea / Haptoria / Haptorida / Tracheliidae / Trac
Eukaryota / Alveolata / Ciliophora / Intramacronucleata / Litostomatea / Haptoria / Haptorida / Trachelophyllidae
Eukaryota / Alveolata / Ciliophora / Intramacronucleata / Litostomatea / Haptoria / Haptorida / Trachelophyllidae
Eukaryota / Alveolata / Ciliophora / Intramacronucleata / Litostomatea / Haptoria / Pleurostomatida / Amphilepti
Eukaryota / Alveolata / Ciliophora / Intramacronucleata / Litostomatea / Haptoria / Pleurostomatida / Litonotidae
Eukaryota / Alveolata / Ciliophora / Intramacronucleata / Litostomatea / Haptoria / Pleurostomatida / Litonotidae
Eukaryota / Alveolata / Ciliophora / Intramacronucleata / Litostomatea / Haptoria / Pleurostomatida / Litonotidae
Eukaryota / Alveolata / Ciliophora / Intramacronucleata / Litostomatea / Haptoria / Pleurostomatida / Litonotidae

Eukaryota / Alveolata / Ciliophora / Intramacronucleata / Litostomatea / Trichostomatia / Amylovoracidae / Bitric
Eukaryota / Alveolata / Ciliophora / Intramacronucleata / Litostomatea / Trichostomatia / Entodiniomorphida / Bl
Eukaryota / Alveolata / Ciliophora / Intramacronucleata / Litostomatea / Trichostomatia / Entodiniomorphida / Bu
Eukaryota / Alveolata / Ciliophora / Intramacronucleata / Litostomatea / Trichostomatia / Entodiniomorphida / Bu
Eukaryota / Alveolata / Ciliophora / Intramacronucleata / Litostomatea / Trichostomatia / Entodiniomorphida / Bu
Eukaryota / Alveolata / Ciliophora / Intramacronucleata / Litostomatea / Trichostomatia / Entodiniomorphida / Bu
Eukaryota / Alveolata / Ciliophora / Intramacronucleata / Litostomatea / Trichostomatia / Entodiniomorphida / Bu
Eukaryota / Alveolata / Ciliophora / Intramacronucleata / Litostomatea / Trichostomatia / Entodiniomorphida / Bu
Eukaryota / Alveolata / Ciliophora / Intramacronucleata / Litostomatea / Trichostomatia / Entodiniomorphida / Bu
Eukaryota / Alveolata / Ciliophora / Intramacronucleata / Litostomatea / Trichostomatia / Entodiniomorphida / Po
Eukaryota / Alveolata / Ciliophora / Intramacronucleata / Litostomatea / Trichostomatia / Vestibuliferida / Isotrich
Eukaryota / Alveolata / Ciliophora / Intramacronucleata / Litostomatea / Trichostomatia / Vestibuliferida / Paraiso
Eukaryota / Alveolata / Ciliophora / Intramacronucleata / Nassophorea / Microthoracida / Microthoracidae / Lept
Eukaryota / Alveolata / Ciliophora / Intramacronucleata / Nassophorea / Microthoracida / Microthoracidae / Lept
Eukaryota / Alveolata / Ciliophora / Intramacronucleata / Nassophorea / Microthoracida / Microthoracidae / Lept
Eukaryota / Alveolata / Ciliophora / Intramacronucleata / Nassophorea / Microthoracida / Microthoracidae / Lept
Eukaryota / Alveolata / Ciliophora / Intramacronucleata / Nassophorea / Microthoracida / Microthoracidae / Lept
Eukaryota / Alveolata / Ciliophora / Intramacronucleata / Nassophorea / Microthoracida / Microthoracidae / Lept
Eukaryota / Alveolata / Ciliophora / Intramacronucleata / Nassophorea / Microthoracida / Microthoracidae / Lept
Eukaryota / Alveolata / Ciliophora / Intramacronucleata / Nassophorea / Microthoracida / Microthoracidae / Lept
Eukaryota / Alveolata / Ciliophora / Intramacronucleata / Nassophorea / Microthoracida / Microthoracidae / Lept
Eukaryota / Alveolata / Ciliophora / Intramacronucleata / Nassophorea / Microthoracida / Microthoracidae / Lept
Eukaryota / Alveolata / Ciliophora / Intramacronucleata / Nassophorea / Microthoracida / Microthoracidae / Lept
Eukaryota / Alveolata / Ciliophora / Intramacronucleata / Nassophorea / Microthoracida / Microthoracidae / Lept
Eukaryota / Alveolata / Ciliophora / Intramacronucleata / Nassophorea / Microthoracida / Microthoracidae / Lept
Eukaryota / Alveolata / Ciliophora / Intramacronucleata / Nassophorea / Microthoracida / Microthoracidae / Lept
Eukaryota / Alveolata / Ciliophora / Intramacronucleata / Nassophorea / Microthoracida / Microthoracidae / Lept
Eukaryota / Alveolata / Ciliophora / Intramacronucleata / Nassophorea / Nassulida / Colpodidiidae / Colpodidium
Eukaryota / Alveolata / Ciliophora / Intramacronucleata / Nassophorea / Nassulida / Furgasoniidae / Parafurgason
Eukaryota / Alveolata / Ciliophora / Intramacronucleata / Nassophorea / Nassulida / Furgasoniidae / Parafurgason
Eukaryota / Alveolata / Ciliophora / Intramacronucleata / Nassophorea / Nassulida / Nassulidae / Nassula
Eukaryota / Alveolata / Ciliophora / Intramacronucleata / Nassophorea / Nassulida / Nassulidae / Obertrumia
Eukaryota / Alveolata / Ciliophora / Intramacronucleata / Nassophorea / Nassulida / Nassulidae / Obertrumia
Eukaryota / Alveolata / Ciliophora / Intramacronucleata / Nassophorea / Nassulida / Nassulidae / Obertrumia
Eukaryota / Alveolata / Ciliophora / Intramacronucleata / Nassophorea / Nassulida / Nassulidae / Obertrumia
Eukaryota / Alveolata / Ciliophora / Intramacronucleata / Nassophorea / Nassulida / Nassulidae / Obertrumia
Eukaryota / Alveolata / Ciliophora / Intramacronucleata / Nassophorea / Nassulida / Nassulidae / Obertrumia
Eukaryota / Alveolata / Ciliophora / Intramacronucleata / Nassophorea / Synhymeniida / Orthodonellidae / Ortho
Eukaryota / Alveolata / Ciliophora / Intramacronucleata / Oligohymenophorea / Apostomatida / Colliniidae / Colli
Eukaryota / Alveolata / Ciliophora / Intramacronucleata / Oligohymenophorea / Apostomatida / Colliniidae / Colli
Eukaryota / Alveolata / Ciliophora / Intramacronucleata / Oligohymenophorea / Apostomatida / Colliniidae / Colli
Eukaryota / Alveolata / Ciliophora / Intramacronucleata / Oligohymenophorea / Apostomatida / Colliniidae / Colli
Eukaryota / Alveolata / Ciliophora / Intramacronucleata / Oligohymenophorea / Apostomatida / Foettingeriidae /
Eukaryota / Alveolata / Ciliophora / Intramacronucleata / Oligohymenophorea / Apostomatida / Foettingeriidae /
Eukaryota / Alveolata / Ciliophora / Intramacronucleata / Oligohymenophorea / Apostomatida / Foettingeriidae /
Eukaryota / Alveolata / Ciliophora / Intramacronucleata / Oligohymenophorea / Apostomatida / Foettingeriidae /
Eukaryota / Alveolata / Ciliophora / Intramacronucleata / Oligohymenophorea / Apostomatida / Foettingeriidae /
Eukaryota / Alveolata / Ciliophora / Intramacronucleata / Oligohymenophorea / Apostomatida / Foettingeriidae /

Eukaryota / Alveolata / Ciliophora / Intramacronucleata / Oligohymenophorea / Apostomatida / Foettingeriidae /
Eukaryota / Alveolata / Ciliophora / Intramacronucleata / Oligohymenophorea / Apostomatida / Foettingeriidae /
Eukaryota / Alveolata / Ciliophora / Intramacronucleata / Oligohymenophorea / Apostomatida / Foettingeriidae /
Eukaryota / Alveolata / Ciliophora / Intramacronucleata / Oligohymenophorea / Apostomatida / Foettingeriidae /
Eukaryota / Alveolata / Ciliophora / Intramacronucleata / Oligohymenophorea / Apostomatida / Foettingeriidae /
Eukaryota / Alveolata / Ciliophora / Intramacronucleata / Oligohymenophorea / Apostomatida / Foettingeriidae /
Eukaryota / Alveolata / Ciliophora / Intramacronucleata / Oligohymenophorea / Apostomatida / Foettingeriidae /
Eukaryota / Alveolata / Ciliophora / Intramacronucleata / Oligohymenophorea / Apostomatida / Foettingeriidae /
Eukaryota / Alveolata / Ciliophora / Intramacronucleata / Oligohymenophorea / Apostomatida / Foettingeriidae /
Eukaryota / Alveolata / Ciliophora / Intramacronucleata / Oligohymenophorea / Apostomatida / Foettingeriidae /
Eukaryota / Alveolata / Ciliophora / Intramacronucleata / Oligohymenophorea / Apostomatida / Foettingeriidae /
Eukaryota / Alveolata / Ciliophora / Intramacronucleata / Oligohymenophorea / Apostomatida / Foettingeriidae /
Eukaryota / Alveolata / Ciliophora / Intramacronucleata / Oligohymenophorea / Apostomatida / Foettingeriidae /
Eukaryota / Alveolata / Ciliophora / Intramacronucleata / Oligohymenophorea / Apostomatida / Foettingeriidae /
Eukaryota / Alveolata / Ciliophora / Intramacronucleata / Oligohymenophorea / Apostomatida / Foettingeriidae /
Eukaryota / Alveolata / Ciliophora / Intramacronucleata / Oligohymenophorea / Apostomatida / Foettingeriidae /
Eukaryota / Alveolata / Ciliophora / Intramacronucleata / Oligohymenophorea / Apostomatida / Foettingeriidae /
Eukaryota / Alveolata / Ciliophora / Intramacronucleata / Oligohymenophorea / Apostomatida / Foettingeriidae /
Eukaryota / Alveolata / Ciliophora / Intramacronucleata / Oligohymenophorea / Apostomatida / Foettingeriidae /
Eukaryota / Alveolata / Ciliophora / Intramacronucleata / Oligohymenophorea / Apostomatida / Foettingeriidae /
Eukaryota / Alveolata / Ciliophora / Intramacronucleata / Oligohymenophorea / Apostomatida / Foettingeriidae /
Eukaryota / Alveolata / Ciliophora / Intramacronucleata / Oligohymenophorea / Apostomatida / Foettingeriidae /
Eukaryota / Alveolata / Ciliophora / Intramacronucleata / Oligohymenophorea / Apostomatida / Foettingeriidae /
Eukaryota / Alveolata / Ciliophora / Intramacronucleata / Oligohymenophorea / Apostomatida / Foettingeriidae /
Eukaryota / Alveolata / Ciliophora / Intramacronucleata / Oligohymenophorea / Apostomatida / Foettingeriidae /
Eukaryota / Alveolata / Ciliophora / Intramacronucleata / Oligohymenophorea / Apostomatida / Foettingeriidae /
Eukaryota / Alveolata / Ciliophora / Intramacronucleata / Oligohymenophorea / Apostomatida / Foettingeriidae /
Eukaryota / Alveolata / Ciliophora / Intramacronucleata / Oligohymenophorea / Apostomatida / Foettingeriidae /
Eukaryota / Alveolata / Ciliophora / Intramacronucleata / Oligohymenophorea / Apostomatida / Foettingeriidae /
Eukaryota / Alveolata / Ciliophora / Intramacronucleata / Oligohymenophorea / Apostomatida / Foettingeriidae /
Eukaryota / Alveolata / Ciliophora / Intramacronucleata / Oligohymenophorea / Apostomatida / Foettingeriidae /
Eukaryota / Alveolata / Ciliophora / Intramacronucleata / Oligohymenophorea / Apostomatida / Foettingeriidae /
Eukaryota / Alveolata / Ciliophora / Intramacronucleata / Oligohymenophorea / Apostomatida / Foettingeriidae /
Eukaryota / Alveolata / Ciliophora / Intramacronucleata / Oligohymenophorea / Apostomatida / Foettingeriidae /
Eukaryota / Alveolata / Ciliophora / Intramacronucleata / Oligohymenophorea / Apostomatida / Foettingeriidae /
Eukaryota / Alveolata / Ciliophora / Intramacronucleata / Oligohymenophorea / Apostomatida / Pseudocolliniidae
Eukaryota / Alveolata / Ciliophora / Intramacronucleata / Oligohymenophorea / Apostomatida / Pseudocolliniidae
Eukaryota / Alveolata / Ciliophora / Intramacronucleata / Oligohymenophorea / Apostomatida / Pseudocolliniidae
Eukaryota / Alveolata / Ciliophora / Intramacronucleata / Oligohymenophorea / Apostomatida / Pseudocolliniidae
Eukaryota / Alveolata / Ciliophora / Intramacronucleata / Oligohymenophorea / Apostomatida / Pseudocolliniidae
Eukaryota / Alveolata / Ciliophora / Intramacronucleata / Oligohymenophorea / Apostomatida / Pseudocolliniidae
Eukaryota / Alveolata / Ciliophora / Intramacronucleata / Oligohymenophorea / Apostomatida / Pseudocolliniidae
Eukaryota / Alveolata / Ciliophora / Intramacronucleata / Oligohymenophorea / Apostomatida / Pseudocolliniidae
Eukaryota / Alveolata / Ciliophora / Intramacronucleata / Oligohymenophorea / Apostomatida / Pseudocolliniidae
Eukaryota / Alveolata / Ciliophora / Intramacronucleata / Oligohymenophorea / Apostomatida / Pseudocolliniidae
Eukaryota / Alveolata / Ciliophora / Intramacronucleata / Oligohymenophorea / Apostomatida / Pseudocolliniidae
Eukaryota / Alveolata / Ciliophora / Intramacronucleata / Oligohymenophorea / Apostomatida / Pseudocolliniidae
Eukaryota / Alveolata / Ciliophora / Intramacronucleata / Oligohymenophorea / Apostomatida / Pseudocolliniidae

Eukaryota / Alveolata / Ciliophora / Intramacronucleata / Oligohymenophorea / Apostomatida / Pseudocolliniidae
Eukaryota / Alveolata / Ciliophora / Intramacronucleata / Oligohymenophorea / Apostomatida / Pseudocolliniidae
Eukaryota / Alveolata / Ciliophora / Intramacronucleata / Oligohymenophorea / Apostomatida / Pseudocolliniidae
Eukaryota / Alveolata / Ciliophora / Intramacronucleata / Oligohymenophorea / Apostomatida / Pseudocolliniidae
Eukaryota / Alveolata / Ciliophora / Intramacronucleata / Oligohymenophorea / Apostomatida / Pseudocolliniidae
Eukaryota / Alveolata / Ciliophora / Intramacronucleata / Oligohymenophorea / Apostomatida / Pseudocolliniidae
Eukaryota / Alveolata / Ciliophora / Intramacronucleata / Oligohymenophorea / Apostomatida / Pseudocolliniidae
Eukaryota / Alveolata / Ciliophora / Intramacronucleata / Oligohymenophorea / Apostomatida / Pseudocolliniidae
Eukaryota / Alveolata / Ciliophora / Intramacronucleata / Oligohymenophorea / Apostomatida / Pseudocolliniidae
Eukaryota / Alveolata / Ciliophora / Intramacronucleata / Oligohymenophorea / Apostomatida / Pseudocolliniidae
Eukaryota / Alveolata / Ciliophora / Intramacronucleata / Oligohymenophorea / Apostomatida / Pseudocolliniidae
Eukaryota / Alveolata / Ciliophora / Intramacronucleata / Oligohymenophorea / Apostomatida / Pseudocolliniidae
Eukaryota / Alveolata / Ciliophora / Intramacronucleata / Oligohymenophorea / Apostomatida / Pseudocolliniidae
Eukaryota / Alveolata / Ciliophora / Intramacronucleata / Oligohymenophorea / Apostomatida / Pseudocolliniidae
Eukaryota / Alveolata / Ciliophora / Intramacronucleata / Oligohymenophorea / Apostomatida / Pseudocolliniidae
Eukaryota / Alveolata / Ciliophora / Intramacronucleata / Oligohymenophorea / Apostomatida / Pseudocolliniidae
Eukaryota / Alveolata / Ciliophora / Intramacronucleata / Oligohymenophorea / Apostomatida / Pseudocolliniidae
Eukaryota / Alveolata / Ciliophora / Intramacronucleata / Oligohymenophorea / Astomatida / Anoplophryidae / N
Eukaryota / Alveolata / Ciliophora / Intramacronucleata / Oligohymenophorea / Astomatida / Anoplophryidae / N
Eukaryota / Alveolata / Ciliophora / Intramacronucleata / Oligohymenophorea / Astomatida / Radiophryidae / Me
Eukaryota / Alveolata / Ciliophora / Intramacronucleata / Oligohymenophorea / Astomatida / Radiophryidae / Me
Eukaryota / Alveolata / Ciliophora / Intramacronucleata / Oligohymenophorea / Hymenostomatida / Tetrahymeni
Eukaryota / Alveolata / Ciliophora / Intramacronucleata / Oligohymenophorea / Peniculida / Parameciidae / Parar
Eukaryota / Alveolata / Ciliophora / Intramacronucleata / Oligohymenophorea / Peniculida / Stokesiidae / Stokesi
Eukaryota / Alveolata / Ciliophora / Intramacronucleata / Oligohymenophorea / Peniculida / Stokesiidae / Stokesi
Eukaryota / Alveolata / Ciliophora / Intramacronucleata / Oligohymenophorea / Peniculida / Stokesiidae / Stokesi
Eukaryota / Alveolata / Ciliophora / Intramacronucleata / Oligohymenophorea / Peniculida / Stokesiidae / Stokesi
Eukaryota / Alveolata / Ciliophora / Intramacronucleata / Oligohymenophorea / Peniculida / Stokesiidae / Stokesi
Eukaryota / Alveolata / Ciliophora / Intramacronucleata / Oligohymenophorea / Peniculida / Stokesiidae / Stokesi
Eukaryota / Alveolata / Ciliophora / Intramacronucleata / Oligohymenophorea / Peniculida / Stokesiidae / Stokesi
Eukaryota / Alveolata / Ciliophora / Intramacronucleata / Oligohymenophorea / Peniculida / Stokesiidae / Stokesi
Eukaryota / Alveolata / Ciliophora / Intramacronucleata / Oligohymenophorea / Peniculida / Stokesiidae / Stokesi
Eukaryota / Alveolata / Ciliophora / Intramacronucleata / Oligohymenophorea / Peniculida / Urocentridae / Uroce
Eukaryota / Alveolata / Ciliophora / Intramacronucleata / Oligohymenophorea / Peniculida / Urocentridae / Uroce
Eukaryota / Alveolata / Ciliophora / Intramacronucleata / Oligohymenophorea / Peniculida / Urocentridae / Uroce
Eukaryota / Alveolata / Ciliophora / Intramacronucleata / Oligohymenophorea / Peniculida / Urocentridae / Uroce
Eukaryota / Alveolata / Ciliophora / Intramacronucleata / Oligohymenophorea / Peritrichia / Opisthonectidae / Te
Eukaryota / Alveolata / Ciliophora / Intramacronucleata / Oligohymenophorea / Peritrichia / Trichodinidae / Trich
Eukaryota / Alveolata / Ciliophora / Intramacronucleata / Oligohymenophorea / Peritrichia / Trichodinidae / Trich
Eukaryota / Alveolata / Ciliophora / Intramacronucleata / Oligohymenophorea / Peritrichia / Trichodinidae / Trich
Eukaryota / Alveolata / Ciliophora / Intramacronucleata / Oligohymenophorea / Peritrichia / Trichodinidae / Trich
Eukaryota / Alveolata / Ciliophora / Intramacronucleata / Oligohymenophorea / Peritrichia / Trichodinidae / Trich
Eukaryota / Alveolata / Ciliophora / Intramacronucleata / Oligohymenophorea / Peritrichia / Trichodinidae / Trich
Eukaryota / Alveolata / Ciliophora / Intramacronucleata / Oligohymenophorea / Peritrichia / Trichodinidae / Trich
Eukaryota / Alveolata / Ciliophora / Intramacronucleata / Oligohymenophorea / Peritrichia / Trichodinidae / Trich
Eukaryota / Alveolata / Ciliophora / Intramacronucleata / Oligohymenophorea / Peritrichia / Trichodinidae / Trich
Eukaryota / Alveolata / Ciliophora / Intramacronucleata / Oligohymenophorea / Peritrichia / Trichodinidae / Trich
Eukaryota / Alveolata / Ciliophora / Intramacronucleata / Oligohymenophorea / Peritrichia / Trichodinidae / Trich

Eukaryota / Alveolata / Ciliophora / Intramacronucleata / Oligohymenophorea / Peritrichia / Trichodinidae / Tricho
Eukaryota / Alveolata / Ciliophora / Intramacronucleata / Oligohymenophorea / Peritrichia / Trichodinidae / Tricho
Eukaryota / Alveolata / Ciliophora / Intramacronucleata / Oligohymenophorea / Peritrichia / Vorticellidae / Apoca
Eukaryota / Alveolata / Ciliophora / Intramacronucleata / Oligohymenophorea / Peritrichia / Vorticellidae / Pseudo
Eukaryota / Alveolata / Ciliophora / Intramacronucleata / Oligohymenophorea / Peritrichia / Vorticellidae / Vortice
Eukaryota / Alveolata / Ciliophora / Intramacronucleata / Oligohymenophorea / Peritrichia / Vorticellidae / Vortice
Eukaryota / Alveolata / Ciliophora / Intramacronucleata / Oligohymenophorea / Peritrichia / Zoothamniidae / Zoo
Eukaryota / Alveolata / Ciliophora / Intramacronucleata / Oligohymenophorea / Peritrichia / Zoothamniidae / Zoo
Eukaryota / Alveolata / Ciliophora / Intramacronucleata / Oligohymenophorea / Peritrichia / Zoothamniidae / Zoo
Eukaryota / Alveolata / Ciliophora / Intramacronucleata / Oligohymenophorea / Peritrichia / Zoothamniidae / Zoo
Eukaryota / Alveolata / Ciliophora / Intramacronucleata / Oligohymenophorea / Peritrichia / Zoothamniidae / Zoo
Eukaryota / Alveolata / Ciliophora / Intramacronucleata / Oligohymenophorea / Scuticociliatia / Philasterida / Cine
Eukaryota / Alveolata / Ciliophora / Intramacronucleata / Oligohymenophorea / Scuticociliatia / Philasterida / Cine
Eukaryota / Alveolata / Ciliophora / Intramacronucleata / Oligohymenophorea / Scuticociliatia / Philasterida / Cine
Eukaryota / Alveolata / Ciliophora / Intramacronucleata / Oligohymenophorea / Scuticociliatia / Philasterida / Cine
Eukaryota / Alveolata / Ciliophora / Intramacronucleata / Oligohymenophorea / Scuticociliatia / Philasterida / Cine
Eukaryota / Alveolata / Ciliophora / Intramacronucleata / Oligohymenophorea / Scuticociliatia / Philasterida / Cine
Eukaryota / Alveolata / Ciliophora / Intramacronucleata / Oligohymenophorea / Scuticociliatia / Philasterida / Cine
Eukaryota / Alveolata / Ciliophora / Intramacronucleata / Oligohymenophorea / Scuticociliatia / Philasterida / Cine
Eukaryota / Alveolata / Ciliophora / Intramacronucleata / Oligohymenophorea / Scuticociliatia / Philasterida / Cine
Eukaryota / Alveolata / Ciliophora / Intramacronucleata / Oligohymenophorea / Scuticociliatia / Philasterida / Cine
Eukaryota / Alveolata / Ciliophora / Intramacronucleata / Oligohymenophorea / Scuticociliatia / Philasterida / Cine
Eukaryota / Alveolata / Ciliophora / Intramacronucleata / Oligohymenophorea / Scuticociliatia / Philasterida / Cine
Eukaryota / Alveolata / Ciliophora / Intramacronucleata / Oligohymenophorea / Scuticociliatia / Philasterida / Cine
Eukaryota / Alveolata / Ciliophora / Intramacronucleata / Oligohymenophorea / Scuticociliatia / Philasterida / Cine
Eukaryota / Alveolata / Ciliophora / Intramacronucleata / Oligohymenophorea / Scuticociliatia / Philasterida / Cine
Eukaryota / Alveolata / Ciliophora / Intramacronucleata / Oligohymenophorea / Scuticociliatia / Philasterida / Cine
Eukaryota / Alveolata / Ciliophora / Intramacronucleata / Oligohymenophorea / Scuticociliatia / Philasterida / Cine
Eukaryota / Alveolata / Ciliophora / Intramacronucleata / Oligohymenophorea / Scuticociliatia / Philasterida / Cine
Eukaryota / Alveolata / Ciliophora / Intramacronucleata / Oligohymenophorea / Scuticociliatia / Philasterida / Cine
Eukaryota / Alveolata / Ciliophora / Intramacronucleata / Oligohymenophorea / Scuticociliatia / Philasterida / Cine
Eukaryota / Alveolata / Ciliophora / Intramacronucleata / Oligohymenophorea / Scuticociliatia / Philasterida / Cine
Eukaryota / Alveolata / Ciliophora / Intramacronucleata / Oligohymenophorea / Scuticociliatia / Philasterida / Cine
Eukaryota / Alveolata / Ciliophora / Intramacronucleata / Oligohymenophorea / Scuticociliatia / Philasterida / Cine
Eukaryota / Alveolata / Ciliophora / Intramacronucleata / Oligohymenophorea / Scuticociliatia / Philasterida / Cine
Eukaryota / Alveolata / Ciliophora / Intramacronucleata / Oligohymenophorea / Scuticociliatia / Philasterida / Cine
Eukaryota / Alveolata / Ciliophora / Intramacronucleata / Oligohymenophorea / Scuticociliatia / Philasterida / Cine
Eukaryota / Alveolata / Ciliophora / Intramacronucleata / Oligohymenophorea / Scuticociliatia / Philasterida / Cine
Eukaryota / Alveolata / Ciliophora / Intramacronucleata / Oligohymenophorea / Scuticociliatia / Philasterida / Cine
Eukaryota / Alveolata / Ciliophora / Intramacronucleata / Oligohymenophorea / Scuticociliatia / Philasterida / Cine
Eukaryota / Alveolata / Ciliophora / Intramacronucleata / Oligohymenophorea / Scuticociliatia / Philasterida / Cine
Eukaryota / Alveolata / Ciliophora / Intramacronucleata / Oligohymenophorea / Scuticociliatia / Philasterida / Cine
Eukaryota / Alveolata / Ciliophora / Intramacronucleata / Oligohymenophorea / Scuticociliatia / Philasterida / Cine
Eukaryota / Alveolata / Ciliophora / Intramacronucleata / Oligohymenophorea / Scuticociliatia / Philasterida / Cine
Eukaryota / Alveolata / Ciliophora / Intramacronucleata / Oligohymenophorea / Scuticociliatia / Philasterida / Cine
Eukaryota / Alveolata / Ciliophora / Intramacronucleata / Oligohymenophorea / Scuticociliatia / Philasterida / Cine
Eukaryota / Alveolata / Ciliophora / Intramacronucleata / Oligohymenophorea / Scuticociliatia / Philasterida / Cine
Eukaryota / Alveolata / Ciliophora / Intramacronucleata / Oligohymenophorea / Scuticociliatia / Philasterida / Cine

Eukaryota / Alveolata / Ciliophora / Intramacronucleata / Oligohymenophorea / Scuticociliatia / Philasterida / Cine
Eukaryota / Alveolata / Ciliophora / Intramacronucleata / Oligohymenophorea / Scuticociliatia / Philasterida / Cine
Eukaryota / Alveolata / Ciliophora / Intramacronucleata / Oligohymenophorea / Scuticociliatia / Philasterida / Cine
Eukaryota / Alveolata / Ciliophora / Intramacronucleata / Oligohymenophorea / Scuticociliatia / Philasterida / Cine
Eukaryota / Alveolata / Ciliophora / Intramacronucleata / Oligohymenophorea / Scuticociliatia / Philasterida / Cine
Eukaryota / Alveolata / Ciliophora / Intramacronucleata / Oligohymenophorea / Scuticociliatia / Philasterida / Cine
Eukaryota / Alveolata / Ciliophora / Intramacronucleata / Oligohymenophorea / Scuticociliatia / Philasterida / Cine
Eukaryota / Alveolata / Ciliophora / Intramacronucleata / Oligohymenophorea / Scuticociliatia / Philasterida / Cine
Eukaryota / Alveolata / Ciliophora / Intramacronucleata / Oligohymenophorea / Scuticociliatia / Philasterida / Cine
Eukaryota / Alveolata / Ciliophora / Intramacronucleata / Oligohymenophorea / Scuticociliatia / Philasterida / Cine
Eukaryota / Alveolata / Ciliophora / Intramacronucleata / Oligohymenophorea / Scuticociliatia / Philasterida / Cine
Eukaryota / Alveolata / Ciliophora / Intramacronucleata / Oligohymenophorea / Scuticociliatia / Philasterida / Cine
Eukaryota / Alveolata / Ciliophora / Intramacronucleata / Oligohymenophorea / Scuticociliatia / Philasterida / Cine
Eukaryota / Alveolata / Ciliophora / Intramacronucleata / Oligohymenophorea / Scuticociliatia / Philasterida / Cine
Eukaryota / Alveolata / Ciliophora / Intramacronucleata / Oligohymenophorea / Scuticociliatia / Philasterida / Cine
Eukaryota / Alveolata / Ciliophora / Intramacronucleata / Oligohymenophorea / Scuticociliatia / Philasterida / Cine
Eukaryota / Alveolata / Ciliophora / Intramacronucleata / Oligohymenophorea / Scuticociliatia / Philasterida / Cine
Eukaryota / Alveolata / Ciliophora / Intramacronucleata / Oligohymenophorea / Scuticociliatia / Philasterida / Cine
Eukaryota / Alveolata / Ciliophora / Intramacronucleata / Oligohymenophorea / Scuticociliatia / Philasterida / Cine
Eukaryota / Alveolata / Ciliophora / Intramacronucleata / Oligohymenophorea / Scuticociliatia / Philasterida / Cine
Eukaryota / Alveolata / Ciliophora / Intramacronucleata / Oligohymenophorea / Scuticociliatia / Philasterida / Cine
Eukaryota / Alveolata / Ciliophora / Intramacronucleata / Oligohymenophorea / Scuticociliatia / Philasterida / Cine
Eukaryota / Alveolata / Ciliophora / Intramacronucleata / Oligohymenophorea / Scuticociliatia / Philasterida / Cine
Eukaryota / Alveolata / Ciliophora / Intramacronucleata / Oligohymenophorea / Scuticociliatia / Philasterida / Cine
Eukaryota / Alveolata / Ciliophora / Intramacronucleata / Oligohymenophorea / Scuticociliatia / Philasterida / Cine
Eukaryota / Alveolata / Ciliophora / Intramacronucleata / Oligohymenophorea / Scuticociliatia / Philasterida / Cine
Eukaryota / Alveolata / Ciliophora / Intramacronucleata / Oligohymenophorea / Scuticociliatia / Philasterida / Cine
Eukaryota / Alveolata / Ciliophora / Intramacronucleata / Oligohymenophorea / Scuticociliatia / Philasterida / Cine
Eukaryota / Alveolata / Ciliophora / Intramacronucleata / Oligohymenophorea / Scuticociliatia / Philasterida / Cine
Eukaryota / Alveolata / Ciliophora / Intramacronucleata / Oligohymenophorea / Scuticociliatia / Philasterida / Cine
Eukaryota / Alveolata / Ciliophora / Intramacronucleata / Oligohymenophorea / Scuticociliatia / Philasterida / Cine
Eukaryota / Alveolata / Ciliophora / Intramacronucleata / Oligohymenophorea / Scuticociliatia / Philasterida / Cine
Eukaryota / Alveolata / Ciliophora / Intramacronucleata / Oligohymenophorea / Scuticociliatia / Philasterida / Cine
Eukaryota / Alveolata / Ciliophora / Intramacronucleata / Oligohymenophorea / Scuticociliatia / Philasterida / Cine
Eukaryota / Alveolata / Ciliophora / Intramacronucleata / Oligohymenophorea / Scuticociliatia / Philasterida / Cine
Eukaryota / Alveolata / Ciliophora / Intramacronucleata / Oligohymenophorea / Scuticociliatia / Philasterida / Cine
Eukaryota / Alveolata / Ciliophora / Intramacronucleata / Oligohymenophorea / Scuticociliatia / Philasterida / Cine
Eukaryota / Alveolata / Ciliophora / Intramacronucleata / Oligohymenophorea / Scuticociliatia / Philasterida / Cine
Eukaryota / Alveolata / Ciliophora / Intramacronucleata / Oligohymenophorea / Scuticociliatia / Philasterida / Cine
Eukaryota / Alveolata / Ciliophora / Intramacronucleata / Oligohymenophorea / Scuticociliatia / Philasterida / Cine
Eukaryota / Alveolata / Ciliophora / Intramacronucleata / Oligohymenophorea / Scuticociliatia / Philasterida / Cine
Eukaryota / Alveolata / Ciliophora / Intramacronucleata / Oligohymenophorea / Scuticociliatia / Philasterida / Cine
Eukaryota / Alveolata / Ciliophora / Intramacronucleata / Oligohymenophorea / Scuticociliatia / Philasterida / Cine
Eukaryota / Alveolata / Ciliophora / Intramacronucleata / Oligohymenophorea / Scuticociliatia / Philasterida / Cine
Eukaryota / Alveolata / Ciliophora / Intramacronucleata / Oligohymenophorea / Scuticociliatia / Philasterida / Cine
Eukaryota / Alveolata / Ciliophora / Intramacronucleata / Oligohymenophorea / Scuticociliatia / Philasterida / Cine
Eukaryota / Alveolata / Ciliophora / Intramacronucleata / Oligohymenophorea / Scuticociliatia / Philasterida / Cine
Eukaryota / Alveolata / Ciliophora / Intramacronucleata / Oligohymenophorea / Scuticociliatia / Philasterida / Cine

Eukaryota / Alveolata / Ciliophora / Intramacronucleata / Oligohymenophorea / Scuticociliatia / Philasterida / Cine
Eukaryota / Alveolata / Ciliophora / Intramacronucleata / Oligohymenophorea / Scuticociliatia / Philasterida / Cine
Eukaryota / Alveolata / Ciliophora / Intramacronucleata / Oligohymenophorea / Scuticociliatia / Philasterida / Cine
Eukaryota / Alveolata / Ciliophora / Intramacronucleata / Oligohymenophorea / Scuticociliatia / Philasterida / Cine
Eukaryota / Alveolata / Ciliophora / Intramacronucleata / Oligohymenophorea / Scuticociliatia / Philasterida / Cine
Eukaryota / Alveolata / Ciliophora / Intramacronucleata / Oligohymenophorea / Scuticociliatia / Philasterida / Cine
Eukaryota / Alveolata / Ciliophora / Intramacronucleata / Oligohymenophorea / Scuticociliatia / Philasterida / Cine
Eukaryota / Alveolata / Ciliophora / Intramacronucleata / Oligohymenophorea / Scuticociliatia / Philasterida / Cine
Eukaryota / Alveolata / Ciliophora / Intramacronucleata / Oligohymenophorea / Scuticociliatia / Philasterida / Cine
Eukaryota / Alveolata / Ciliophora / Intramacronucleata / Oligohymenophorea / Scuticociliatia / Philasterida / Cine
Eukaryota / Alveolata / Ciliophora / Intramacronucleata / Oligohymenophorea / Scuticociliatia / Philasterida / Cine
Eukaryota / Alveolata / Ciliophora / Intramacronucleata / Oligohymenophorea / Scuticociliatia / Philasterida / Cine
Eukaryota / Alveolata / Ciliophora / Intramacronucleata / Oligohymenophorea / Scuticociliatia / Philasterida / Cine
Eukaryota / Alveolata / Ciliophora / Intramacronucleata / Oligohymenophorea / Scuticociliatia / Philasterida / Cine
Eukaryota / Alveolata / Ciliophora / Intramacronucleata / Oligohymenophorea / Scuticociliatia / Philasterida / Cine
Eukaryota / Alveolata / Ciliophora / Intramacronucleata / Oligohymenophorea / Scuticociliatia / Philasterida / Cine
Eukaryota / Alveolata / Ciliophora / Intramacronucleata / Oligohymenophorea / Scuticociliatia / Philasterida / Cine
Eukaryota / Alveolata / Ciliophora / Intramacronucleata / Oligohymenophorea / Scuticociliatia / Philasterida / Cine
Eukaryota / Alveolata / Ciliophora / Intramacronucleata / Oligohymenophorea / Scuticociliatia / Philasterida / Cine
Eukaryota / Alveolata / Ciliophora / Intramacronucleata / Oligohymenophorea / Scuticociliatia / Philasterida / Cine
Eukaryota / Alveolata / Ciliophora / Intramacronucleata / Oligohymenophorea / Scuticociliatia / Philasterida / Cine
Eukaryota / Alveolata / Ciliophora / Intramacronucleata / Oligohymenophorea / Scuticociliatia / Philasterida / Cine
Eukaryota / Alveolata / Ciliophora / Intramacronucleata / Oligohymenophorea / Scuticociliatia / Philasterida / Cine
Eukaryota / Alveolata / Ciliophora / Intramacronucleata / Oligohymenophorea / Scuticociliatia / Philasterida / Cine
Eukaryota / Alveolata / Ciliophora / Intramacronucleata / Oligohymenophorea / Scuticociliatia / Philasterida / Cine
Eukaryota / Alveolata / Ciliophora / Intramacronucleata / Oligohymenophorea / Scuticociliatia / Philasterida / Cine
Eukaryota / Alveolata / Ciliophora / Intramacronucleata / Oligohymenophorea / Scuticociliatia / Philasterida / Cine
Eukaryota / Alveolata / Ciliophora / Intramacronucleata / Oligohymenophorea / Scuticociliatia / Philasterida / Cine
Eukaryota / Alveolata / Ciliophora / Intramacronucleata / Oligohymenophorea / Scuticociliatia / Philasterida / Cine
Eukaryota / Alveolata / Ciliophora / Intramacronucleata / Oligohymenophorea / Scuticociliatia / Philasterida / Cine
Eukaryota / Alveolata / Ciliophora / Intramacronucleata / Oligohymenophorea / Scuticociliatia / Philasterida / Cine
Eukaryota / Alveolata / Ciliophora / Intramacronucleata / Oligohymenophorea / Scuticociliatia / Philasterida / Cine
Eukaryota / Alveolata / Ciliophora / Intramacronucleata / Oligohymenophorea / Scuticociliatia / Philasterida / Cine
Eukaryota / Alveolata / Ciliophora / Intramacronucleata / Oligohymenophorea / Scuticociliatia / Philasterida / Cine
Eukaryota / Alveolata / Ciliophora / Intramacronucleata / Oligohymenophorea / Scuticociliatia / Philasterida / Cine
Eukaryota / Alveolata / Ciliophora / Intramacronucleata / Oligohymenophorea / Scuticociliatia / Philasterida / Cine
Eukaryota / Alveolata / Ciliophora / Intramacronucleata / Oligohymenophorea / Scuticociliatia / Philasterida / Cine
Eukaryota / Alveolata / Ciliophora / Intramacronucleata / Oligohymenophorea / Scuticociliatia / Philasterida / Cine
Eukaryota / Alveolata / Ciliophora / Intramacronucleata / Oligohymenophorea / Scuticociliatia / Philasterida / Cine
Eukaryota / Alveolata / Ciliophora / Intramacronucleata / Oligohymenophorea / Scuticociliatia / Philasterida / Cine
Eukaryota / Alveolata / Ciliophora / Intramacronucleata / Oligohymenophorea / Scuticociliatia / Philasterida / Cine
Eukaryota / Alveolata / Ciliophora / Intramacronucleata / Oligohymenophorea / Scuticociliatia / Philasterida / Cine
Eukaryota / Alveolata / Ciliophora / Intramacronucleata / Oligohymenophorea / Scuticociliatia / Philasterida / Cine
Eukaryota / Alveolata / Ciliophora / Intramacronucleata / Oligohymenophorea / Scuticociliatia / Philasterida / Cine
Eukaryota / Alveolata / Ciliophora / Intramacronucleata / Oligohymenophorea / Scuticociliatia / Philasterida / Cine
Eukaryota / Alveolata / Ciliophora / Intramacronucleata / Oligohymenophorea / Scuticociliatia / Philasterida / Cine
Eukaryota / Alveolata / Ciliophora / Intramacronucleata / Oligohymenophorea / Scuticociliatia / Philasterida / Cine
Eukaryota / Alveolata / Ciliophora / Intramacronucleata / Oligohymenophorea / Scuticociliatia / Philasterida / Coh

Eukaryota / Alveolata / Ciliophora / Intramacronucleata / Oligohymenophorea / Scuticociliatia / Philasterida / Lox
Eukaryota / Alveolata / Ciliophora / Intramacronucleata / Oligohymenophorea / Scuticociliatia / Philasterida / Lox
Eukaryota / Alveolata / Ciliophora / Intramacronucleata / Oligohymenophorea / Scuticociliatia / Philasterida / Lox
Eukaryota / Alveolata / Ciliophora / Intramacronucleata / Oligohymenophorea / Scuticociliatia / Philasterida / Lox
Eukaryota / Alveolata / Ciliophora / Intramacronucleata / Oligohymenophorea / Scuticociliatia / Philasterida / Lox
Eukaryota / Alveolata / Ciliophora / Intramacronucleata / Oligohymenophorea / Scuticociliatia / Philasterida / Lox
Eukaryota / Alveolata / Ciliophora / Intramacronucleata / Oligohymenophorea / Scuticociliatia / Philasterida / Lox
Eukaryota / Alveolata / Ciliophora / Intramacronucleata / Oligohymenophorea / Scuticociliatia / Philasterida / Lox
Eukaryota / Alveolata / Ciliophora / Intramacronucleata / Oligohymenophorea / Scuticociliatia / Philasterida / Lox
Eukaryota / Alveolata / Ciliophora / Intramacronucleata / Oligohymenophorea / Scuticociliatia / Philasterida / Lox
Eukaryota / Alveolata / Ciliophora / Intramacronucleata / Oligohymenophorea / Scuticociliatia / Philasterida / Lox
Eukaryota / Alveolata / Ciliophora / Intramacronucleata / Oligohymenophorea / Scuticociliatia / Philasterida / Lox
Eukaryota / Alveolata / Ciliophora / Intramacronucleata / Oligohymenophorea / Scuticociliatia / Philasterida / Lox
Eukaryota / Alveolata / Ciliophora / Intramacronucleata / Oligohymenophorea / Scuticociliatia / Philasterida / Lox
Eukaryota / Alveolata / Ciliophora / Intramacronucleata / Oligohymenophorea / Scuticociliatia / Philasterida / Lox
Eukaryota / Alveolata / Ciliophora / Intramacronucleata / Oligohymenophorea / Scuticociliatia / Philasterida / Lox
Eukaryota / Alveolata / Ciliophora / Intramacronucleata / Oligohymenophorea / Scuticociliatia / Philasterida / Lox
Eukaryota / Alveolata / Ciliophora / Intramacronucleata / Oligohymenophorea / Scuticociliatia / Philasterida / Lox
Eukaryota / Alveolata / Ciliophora / Intramacronucleata / Oligohymenophorea / Scuticociliatia / Philasterida / Lox
Eukaryota / Alveolata / Ciliophora / Intramacronucleata / Oligohymenophorea / Scuticociliatia / Philasterida / Lox
Eukaryota / Alveolata / Ciliophora / Intramacronucleata / Oligohymenophorea / Scuticociliatia / Philasterida / Lox
Eukaryota / Alveolata / Ciliophora / Intramacronucleata / Oligohymenophorea / Scuticociliatia / Philasterida / Lox
Eukaryota / Alveolata / Ciliophora / Intramacronucleata / Oligohymenophorea / Scuticociliatia / Philasterida / Lox
Eukaryota / Alveolata / Ciliophora / Intramacronucleata / Oligohymenophorea / Scuticociliatia / Philasterida / Lox
Eukaryota / Alveolata / Ciliophora / Intramacronucleata / Oligohymenophorea / Scuticociliatia / Philasterida / Lox
Eukaryota / Alveolata / Ciliophora / Intramacronucleata / Oligohymenophorea / Scuticociliatia / Philasterida / Lox
Eukaryota / Alveolata / Ciliophora / Intramacronucleata / Oligohymenophorea / Scuticociliatia / Philasterida / Orc
Eukaryota / Alveolata / Ciliophora / Intramacronucleata / Oligohymenophorea / Scuticociliatia / Philasterida / Orc
Eukaryota / Alveolata / Ciliophora / Intramacronucleata / Oligohymenophorea / Scuticociliatia / Philasterida / Orc
Eukaryota / Alveolata / Ciliophora / Intramacronucleata / Oligohymenophorea / Scuticociliatia / Philasterida / Orc
Eukaryota / Alveolata / Ciliophora / Intramacronucleata / Oligohymenophorea / Scuticociliatia / Philasterida / Orc
Eukaryota / Alveolata / Ciliophora / Intramacronucleata / Oligohymenophorea / Scuticociliatia / Philasterida / Orc
Eukaryota / Alveolata / Ciliophora / Intramacronucleata / Oligohymenophorea / Scuticociliatia / Philasterida / Orc
Eukaryota / Alveolata / Ciliophora / Intramacronucleata / Oligohymenophorea / Scuticociliatia / Philasterida / Orc
Eukaryota / Alveolata / Ciliophora / Intramacronucleata / Oligohymenophorea / Scuticociliatia / Philasterida / Orc
Eukaryota / Alveolata / Ciliophora / Intramacronucleata / Oligohymenophorea / Scuticociliatia / Philasterida / Orc
Eukaryota / Alveolata / Ciliophora / Intramacronucleata / Oligohymenophorea / Scuticociliatia / Philasterida / Orc
Eukaryota / Alveolata / Ciliophora / Intramacronucleata / Oligohymenophorea / Scuticociliatia / Philasterida / Orc
Eukaryota / Alveolata / Ciliophora / Intramacronucleata / Oligohymenophorea / Scuticociliatia / Philasterida / Orc
Eukaryota / Alveolata / Ciliophora / Intramacronucleata / Oligohymenophorea / Scuticociliatia / Philasterida / Orc
Eukaryota / Alveolata / Ciliophora / Intramacronucleata / Oligohymenophorea / Scuticociliatia / Philasterida / Par
Eukaryota / Alveolata / Ciliophora / Intramacronucleata / Oligohymenophorea / Scuticociliatia / Philasterida / Par
Eukaryota / Alveolata / Ciliophora / Intramacronucleata / Oligohymenophorea / Scuticociliatia / Philasterida / Phil
Eukaryota / Alveolata / Ciliophora / Intramacronucleata / Oligohymenophorea / Scuticociliatia / Philasterida / Phil
Eukaryota / Alveolata / Ciliophora / Intramacronucleata / Oligohymenophorea / Scuticociliatia / Philasterida / Phil
Eukaryota / Alveolata / Ciliophora / Intramacronucleata / Oligohymenophorea / Scuticociliatia / Philasterida / Phil
Eukaryota / Alveolata / Ciliophora / Intramacronucleata / Oligohymenophorea / Scuticociliatia / Philasterida / Phil
Eukaryota / Alveolata / Ciliophora / Intramacronucleata / Oligohymenophorea / Scuticociliatia / Philasterida / Phil

Eukaryota / Alveolata / Ciliophora / Intramacronucleata / Oligohymenophorea / Scuticociliatia / Philasterida / Phil
Eukaryota / Alveolata / Ciliophora / Intramacronucleata / Oligohymenophorea / Scuticociliatia / Philasterida / Phil
Eukaryota / Alveolata / Ciliophora / Intramacronucleata / Oligohymenophorea / Scuticociliatia / Philasterida / Pse
Eukaryota / Alveolata / Ciliophora / Intramacronucleata / Oligohymenophorea / Scuticociliatia / Philasterida / Pse
Eukaryota / Alveolata / Ciliophora / Intramacronucleata / Oligohymenophorea / Scuticociliatia / Philasterida / Pse
Eukaryota / Alveolata / Ciliophora / Intramacronucleata / Oligohymenophorea / Scuticociliatia / Philasterida / Pse
Eukaryota / Alveolata / Ciliophora / Intramacronucleata / Oligohymenophorea / Scuticociliatia / Philasterida / Pse
Eukaryota / Alveolata / Ciliophora / Intramacronucleata / Oligohymenophorea / Scuticociliatia / Philasterida / Pse
Eukaryota / Alveolata / Ciliophora / Intramacronucleata / Oligohymenophorea / Scuticociliatia / Philasterida / Pse
Eukaryota / Alveolata / Ciliophora / Intramacronucleata / Oligohymenophorea / Scuticociliatia / Philasterida / Uro
Eukaryota / Alveolata / Ciliophora / Intramacronucleata / Oligohymenophorea / Scuticociliatia / Philasterida / Uro
Eukaryota / Alveolata / Ciliophora / Intramacronucleata / Oligohymenophorea / Scuticociliatia / Philasterida / Uro
Eukaryota / Alveolata / Ciliophora / Intramacronucleata / Oligohymenophorea / Scuticociliatia / Philasterida / Uro
Eukaryota / Alveolata / Ciliophora / Intramacronucleata / Oligohymenophorea / Scuticociliatia / Philasterida / Uro
Eukaryota / Alveolata / Ciliophora / Intramacronucleata / Oligohymenophorea / Scuticociliatia / Philasterida / Uro
Eukaryota / Alveolata / Ciliophora / Intramacronucleata / Oligohymenophorea / Scuticociliatia / Philasterida / Uro
Eukaryota / Alveolata / Ciliophora / Intramacronucleata / Oligohymenophorea / Scuticociliatia / Philasterida / Uro
Eukaryota / Alveolata / Ciliophora / Intramacronucleata / Oligohymenophorea / Scuticociliatia / Pleuronematida /
Eukaryota / Alveolata / Ciliophora / Intramacronucleata / Oligohymenophorea / Scuticociliatia / Pleuronematida /
Eukaryota / Alveolata / Ciliophora / Intramacronucleata / Oligohymenophorea / Scuticociliatia / Schizocaryidae / S
Eukaryota / Alveolata / Ciliophora / Intramacronucleata / Oligohymenophorea / Scuticociliatia / Thigmotrichida /
Eukaryota / Alveolata / Ciliophora / Intramacronucleata / Phyllopharyngea / Cyrtophoria / Chlamydodontida / Chi
Eukaryota / Alveolata / Ciliophora / Intramacronucleata / Phyllopharyngea / Cyrtophoria / Chlamydodontida / Chi
Eukaryota / Alveolata / Ciliophora / Intramacronucleata / Phyllopharyngea / Cyrtophoria / Chlamydodontida / Chl
Eukaryota / Alveolata / Ciliophora / Intramacronucleata / Phyllopharyngea / Cyrtophoria / Chlamydodontida / Chl
Eukaryota / Alveolata / Ciliophora / Intramacronucleata / Phyllopharyngea / Cyrtophoria / Chlamydodontida / Chl
Eukaryota / Alveolata / Ciliophora / Intramacronucleata / Phyllopharyngea / Cyrtophoria / Chlamydodontida / Lyn
Eukaryota / Alveolata / Ciliophora / Intramacronucleata / Phyllopharyngea / Cyrtophoria / Chlamydodontida / Lyn
Eukaryota / Alveolata / Ciliophora / Intramacronucleata / Phyllopharyngea / Cyrtophoria / Chlamydodontida / Lyn
Eukaryota / Alveolata / Ciliophora / Intramacronucleata / Phyllopharyngea / Cyrtophoria / Chlamydodontida / Lyn
Eukaryota / Alveolata / Ciliophora / Intramacronucleata / Phyllopharyngea / Cyrtophoria / Chlamydodontida / Lyn
Eukaryota / Alveolata / Ciliophora / Intramacronucleata / Phyllopharyngea / Cyrtophoria / Dysteriida / Dysteriidae
Eukaryota / Alveolata / Ciliophora / Intramacronucleata / Phyllopharyngea / Cyrtophoria / Dysteriida / Dysteriidae
Eukaryota / Alveolata / Ciliophora / Intramacronucleata / Phyllopharyngea / Cyrtophoria / Dysteriida / Dysteriidae
Eukaryota / Alveolata / Ciliophora / Intramacronucleata / Phyllopharyngea / Cyrtophoria / Dysteriida / Dysteriidae
Eukaryota / Alveolata / Ciliophora / Intramacronucleata / Phyllopharyngea / Cyrtophoria / Dysteriida / Dysteriidae
Eukaryota / Alveolata / Ciliophora / Intramacronucleata / Phyllopharyngea / Cyrtophoria / Dysteriida / Dysteriidae
Eukaryota / Alveolata / Ciliophora / Intramacronucleata / Phyllopharyngea / Cyrtophoria / Dysteriida / Dysteriidae
Eukaryota / Alveolata / Ciliophora / Intramacronucleata / Phyllopharyngea / Cyrtophoria / Dysteriida / Dysteriidae
Eukaryota / Alveolata / Ciliophora / Intramacronucleata / Phyllopharyngea / Cyrtophoria / Dysteriida / Dysteriidae
Eukaryota / Alveolata / Ciliophora / Intramacronucleata / Phyllopharyngea / Cyrtophoria / Dysteriida / Hartmannu
Eukaryota / Alveolata / Ciliophora / Intramacronucleata / Phyllopharyngea / Cyrtophoria / Dysteriida / Hartmannu
Eukaryota / Alveolata / Ciliophora / Intramacronucleata / Phyllopharyngea / Cyrtophoria / Dysteriida / Hartmannu
Eukaryota / Alveolata / Ciliophora / Intramacronucleata / Phyllopharyngea / Cyrtophoria / Dysteriida / Plesiotricho
Eukaryota / Alveolata / Ciliophora / Intramacronucleata / Phyllopharyngea / Cyrtophoria / Dysteriida / Plesiotricho
Eukaryota / Alveolata / Ciliophora / Intramacronucleata / Phyllopharyngea / Cyrtophoria / Dysteriida / Plesiotricho
Eukaryota / Alveolata / Ciliophora / Intramacronucleata / Phyllopharyngea / Cyrtophoria / Dysteriida / Plesiotricho
Eukaryota / Alveolata / Ciliophora / Intramacronucleata / Phyllopharyngea / Cyrtophoria / Dysteriida / Plesiotricho

Eukaryota / Alveolata / Ciliophora / Intramacronucleata / Phyllopharyngea / Cyrtophoria / Dysteriida / Plesiotricho
Eukaryota / Alveolata / Ciliophora / Intramacronucleata / Phyllopharyngea / Cyrtophoria / Dysteriida / Plesiotricho
Eukaryota / Alveolata / Ciliophora / Intramacronucleata / Phyllopharyngea / Cyrtophoria / Dysteriida / Plesiotricho
Eukaryota / Alveolata / Ciliophora / Intramacronucleata / Phyllopharyngea / Cyrtophoria / Dysteriida / Plesiotricho
Eukaryota / Alveolata / Ciliophora / Intramacronucleata / Phyllopharyngea / Cyrtophoria / Dysteriida / Plesiotricho
Eukaryota / Alveolata / Ciliophora / Intramacronucleata / Phyllopharyngea / Cyrtophoria / Dysteriida / Plesiotricho
Eukaryota / Alveolata / Ciliophora / Intramacronucleata / Phyllopharyngea / Cyrtophoria / Dysteriida / Plesiotricho
Eukaryota / Alveolata / Ciliophora / Intramacronucleata / Phyllopharyngea / Cyrtophoria / Dysteriida / Plesiotricho
Eukaryota / Alveolata / Ciliophora / Intramacronucleata / Phyllopharyngea / Cyrtophoria / Dysteriida / Plesiotricho
Eukaryota / Alveolata / Ciliophora / Intramacronucleata / Phyllopharyngea / Cyrtophoria / Dysteriida / Plesiotricho
Eukaryota / Alveolata / Ciliophora / Intramacronucleata / Phyllopharyngea / Cyrtophoria / Dysteriida / Plesiotricho
Eukaryota / Alveolata / Ciliophora / Intramacronucleata / Phyllopharyngea / Cyrtophoria / Dysteriida / Plesiotricho
Eukaryota / Alveolata / Ciliophora / Intramacronucleata / Phyllopharyngea / Cyrtophoria / Dysteriida / Plesiotricho
Eukaryota / Alveolata / Ciliophora / Intramacronucleata / Phyllopharyngea / Cyrtophoria / Dysteriida / Plesiotricho
Eukaryota / Alveolata / Ciliophora / Intramacronucleata / Phyllopharyngea / Cyrtophoria / Dysteriida / Plesiotricho
Eukaryota / Alveolata / Ciliophora / Intramacronucleata / Phyllopharyngea / Cyrtophoria / Dysteriida / Plesiotricho
Eukaryota / Alveolata / Ciliophora / Intramacronucleata / Phyllopharyngea / Cyrtophoria / Dysteriida / Plesiotricho
Eukaryota / Alveolata / Ciliophora / Intramacronucleata / Phyllopharyngea / Cyrtophoria / Dysteriida / Plesiotricho
Eukaryota / Alveolata / Ciliophora / Intramacronucleata / Phyllopharyngea / Cyrtophoria / Dysteriida / Plesiotricho
Eukaryota / Alveolata / Ciliophora / Intramacronucleata / Phyllopharyngea / Cyrtophoria / Dysteriida / Plesiotricho
Eukaryota / Alveolata / Ciliophora / Intramacronucleata / Phyllopharyngea / Cyrtophoria / Dysteriida / Plesiotricho
Eukaryota / Alveolata / Ciliophora / Intramacronucleata / Phyllopharyngea / Cyrtophoria / Dysteriida / Plesiotricho
Eukaryota / Alveolata / Ciliophora / Intramacronucleata / Phyllopharyngea / Cyrtophoria / Dysteriida / Plesiotricho
Eukaryota / Alveolata / Ciliophora / Intramacronucleata / Phyllopharyngea / Cyrtophoria / Dysteriida / Plesiotricho
Eukaryota / Alveolata / Ciliophora / Intramacronucleata / Phyllopharyngea / Cyrtophoria / Dysteriida / Plesiotricho
Eukaryota / Alveolata / Ciliophora / Intramacronucleata / Phyllopharyngea / Cyrtophoria / Dysteriida / Plesiotricho
Eukaryota / Alveolata / Ciliophora / Intramacronucleata / Phyllopharyngea / Cyrtophoria / Dysteriida / Plesiotricho
Eukaryota / Alveolata / Ciliophora / Intramacronucleata / Phyllopharyngea / Cyrtophoria / Dysteriida / Plesiotricho
Eukaryota / Alveolata / Ciliophora / Intramacronucleata / Phyllopharyngea / Cyrtophoria / Dysteriida / Plesiotricho
Eukaryota / Alveolata / Ciliophora / Intramacronucleata / Phyllopharyngea / Cyrtophoria / Dysteriida / Plesiotricho
Eukaryota / Alveolata / Ciliophora / Intramacronucleata / Phyllopharyngea / Cyrtophoria / Dysteriida / Plesiotricho
Eukaryota / Alveolata / Ciliophora / Intramacronucleata / Phyllopharyngea / Cyrtophoria / Dysteriida / Plesiotricho
Eukaryota / Alveolata / Ciliophora / Intramacronucleata / Phyllopharyngea / Cyrtophoria / Dysteriida / Plesiotricho
Eukaryota / Alveolata / Ciliophora / Intramacronucleata / Phyllopharyngea / Cyrtophoria / Dysteriida / Plesiotricho
Eukaryota / Alveolata / Ciliophora / Intramacronucleata / Phyllopharyngea / Cyrtophoria / Dysteriida / Plesiotricho
Eukaryota / Alveolata / Ciliophora / Intramacronucleata / Phyllopharyngea / Cyrtophoria / Dysteriida / Plesiotricho
Eukaryota / Alveolata / Ciliophora / Intramacronucleata / Phyllopharyngea / Cyrtophoria / Dysteriida / Plesiotricho
Eukaryota / Alveolata / Ciliophora / Intramacronucleata / Phyllopharyngea / Cyrtophoria / Dysteriida / Plesiotricho
Eukaryota / Alveolata / Ciliophora / Intramacronucleata / Phyllopharyngea / Cyrtophoria / Dysteriida / Plesiotricho
Eukaryota / Alveolata / Ciliophora / Intramacronucleata / Phyllopharyngea / Cyrtophoria / Dysteriida / Plesiotricho
Eukaryota / Alveolata / Ciliophora / Intramacronucleata / Phyllopharyngea / Cyrtophoria / Dysteriida / Plesiotricho
Eukaryota / Alveolata / Ciliophora / Intramacronucleata / Phyllopharyngea / Cyrtophoria / Dysteriida / Plesiotricho
Eukaryota / Alveolata / Ciliophora / Intramacronucleata / Phyllopharyngea / Cyrtophoria / Dysteriida / Plesiotricho
Eukaryota / Alveolata / Ciliophora / Intramacronucleata / Phyllopharyngea / Cyrtophoria / Dysteriida / Plesiotricho
Eukaryota / Alveolata / Ciliophora / Intramacronucleata / Phyllopharyngea / Cyrtophoria / Dysteriida / Plesiotricho
Eukaryota / Alveolata / Ciliophora / Intramacronucleata / Phyllopharyngea / Cyrtophoria / Dysteriida / Plesiotricho
Eukaryota / Alveolata / Ciliophora / Intramacronucleata / Phyllopharyngea / Cyrtophoria / Dysteriida / Plesiotricho
Eukaryota / Alveolata / Ciliophora / Intramacronucleata / Phyllopharyngea / Cyrtophoria / Dysteriida / Plesiotricho

Eukaryota / Alveolata / Ciliophora / Intramacronucleata / Phyllopharyngea / Cyrtophoria / Dysteriida / Plesiotrich
Eukaryota / Alveolata / Ciliophora / Intramacronucleata / Phyllopharyngea / Cyrtophoria / Dysteriida / Plesiotrich
Eukaryota / Alveolata / Ciliophora / Intramacronucleata / Phyllopharyngea / Cyrtophoria / Dysteriida / Plesiotrich
Eukaryota / Alveolata / Ciliophora / Intramacronucleata / Phyllopharyngea / Cyrtophoria / Dysteriida / Plesiotrich
Eukaryota / Alveolata / Ciliophora / Intramacronucleata / Phyllopharyngea / Cyrtophorida / Chilodonella
Eukaryota / Alveolata / Ciliophora / Intramacronucleata / Phyllopharyngea / Cyrtophorida / Chilodonella
Eukaryota / Alveolata / Ciliophora / Intramacronucleata / Phyllopharyngea / Cyrtophorida / Chilodonella
Eukaryota / Alveolata / Ciliophora / Intramacronucleata / Phyllopharyngea / Cyrtophorida / Trithigmostoma
Eukaryota / Alveolata / Ciliophora / Intramacronucleata / Phyllopharyngea / Cyrtophorida / Trithigmostoma
Eukaryota / Alveolata / Ciliophora / Intramacronucleata / Phyllopharyngea / Suctoria / Endogenida / Acinetidae /
Eukaryota / Alveolata / Ciliophora / Intramacronucleata / Phyllopharyngea / Suctoria / Endogenida / Acinetidae /
Eukaryota / Alveolata / Ciliophora / Intramacronucleata / Phyllopharyngea / Suctoria / Endogenida / Acinetidae /
Eukaryota / Alveolata / Ciliophora / Intramacronucleata / Phyllopharyngea / Suctoria / Endogenida / Acinetidae /
Eukaryota / Alveolata / Ciliophora / Intramacronucleata / Phyllopharyngea / Suctoria / Endogenida / Acinetidae /
Eukaryota / Alveolata / Ciliophora / Intramacronucleata / Phyllopharyngea / Suctoria / Endogenida / Tokophryida
Eukaryota / Alveolata / Ciliophora / Intramacronucleata / Phyllopharyngea / Suctoria / Exogenida / Ephelotidae /
Eukaryota / Alveolata / Ciliophora / Intramacronucleata / Phyllopharyngea / Suctoria / Exogenida / Ephelotidae /
Eukaryota / Alveolata / Ciliophora / Intramacronucleata / Phyllopharyngea / Suctoria / Exogenida / Ephelotidae /
Eukaryota / Alveolata / Ciliophora / Intramacronucleata / Phyllopharyngea / Suctoria / Exogenida / Ephelotidae /
Eukaryota / Alveolata / Ciliophora / Intramacronucleata / Phyllopharyngea / Suctoria / Exogenida / Ephelotidae /
Eukaryota / Alveolata / Ciliophora / Intramacronucleata / Phyllopharyngea / Suctoria / Exogenida / Ephelotidae /
Eukaryota / Alveolata / Ciliophora / Intramacronucleata / Phyllopharyngea / Suctoria / Exogenida / Ephelotidae /
Eukaryota / Alveolata / Ciliophora / Intramacronucleata / Phyllopharyngea / Suctoria / Exogenida / Ephelotidae /
Eukaryota / Alveolata / Ciliophora / Intramacronucleata / Phyllopharyngea / Suctoria / Exogenida / Ephelotidae /
Eukaryota / Alveolata / Ciliophora / Intramacronucleata / Phyllopharyngea / Suctoria / Exogenida / Ephelotidae /
Eukaryota / Alveolata / Ciliophora / Intramacronucleata / Phyllopharyngea / Suctoria / Exogenida / Ephelotidae /
Eukaryota / Alveolata / Ciliophora / Intramacronucleata / Phyllopharyngea / Suctoria / Exogenida / Ephelotidae /
Eukaryota / Alveolata / Ciliophora / Intramacronucleata / Phyllopharyngea / Suctoria / Exogenida / Ephelotidae /
Eukaryota / Alveolata / Ciliophora / Intramacronucleata / Plagiopylea / Odontostomatida / Epalxellidae / Epalxella
Eukaryota / Alveolata / Ciliophora / Intramacronucleata / Prostomatea / Prorodontida / Colepidae / Apocoleps
Eukaryota / Alveolata / Ciliophora / Intramacronucleata / Prostomatea / Prorodontida / Colepidae / Apocoleps
Eukaryota / Alveolata / Ciliophora / Intramacronucleata / Prostomatea / Prorodontida / Colepidae / Coleps
Eukaryota / Alveolata / Ciliophora / Intramacronucleata / Prostomatea / Prorodontida / Colepidae / Coleps
Eukaryota / Alveolata / Ciliophora / Intramacronucleata / Prostomatea / Prorodontida / Colepidae / Coleps
Eukaryota / Alveolata / Ciliophora / Intramacronucleata / Prostomatea / Prorodontida / Colepidae / Coleps
Eukaryota / Alveolata / Ciliophora / Intramacronucleata / Prostomatea / Prorodontida / Colepidae / Nolandia
Eukaryota / Alveolata / Ciliophora / Intramacronucleata / Prostomatea / Prorodontida / Colepidae / Tiarina
Eukaryota / Alveolata / Ciliophora / Intramacronucleata / Prostomatea / Prorodontida / Colepidae / Tiarina
Eukaryota / Alveolata / Ciliophora / Intramacronucleata / Prostomatea / Prorodontida / Colepidae / Tiarina
Eukaryota / Alveolata / Ciliophora / Intramacronucleata / Prostomatea / Prorodontida / Colepidae / Tiarina
Eukaryota / Alveolata / Ciliophora / Intramacronucleata / Prostomatea / Prorodontida / Colepidae / Tiarina
Eukaryota / Alveolata / Ciliophora / Intramacronucleata / Prostomatea / Prorodontida / Colepidae / Tiarina
Eukaryota / Alveolata / Ciliophora / Intramacronucleata / Prostomatea / Prorodontida / Holophryidae / Cryptocar
Eukaryota / Alveolata / Ciliophora / Intramacronucleata / Prostomatea / Prorodontida / Holophryidae / Cryptocar
Eukaryota / Alveolata / Ciliophora / Intramacronucleata / Prostomatea / Prorodontida / Holophryidae / Cryptocar
Eukaryota / Alveolata / Ciliophora / Intramacronucleata / Prostomatea / Prorodontida / Holophryidae / Cryptocar
Eukaryota / Alveolata / Ciliophora / Intramacronucleata / Prostomatea / Prorodontida / Holophryidae / Cryptocar
Eukaryota / Alveolata / Ciliophora / Intramacronucleata / Prostomatea / Prorodontida / Holophryidae / Cryptocar

Eukaryota / Alveolata / Ciliophora / Intramacronucleata / Prostomatea / Prorodontida / Holophryidae / Cryptocar
Eukaryota / Alveolata / Ciliophora / Intramacronucleata / Prostomatea / Prorodontida / Holophryidae / Cryptocar
Eukaryota / Alveolata / Ciliophora / Intramacronucleata / Prostomatea / Prorodontida / Holophryidae / Cryptocar
Eukaryota / Alveolata / Ciliophora / Intramacronucleata / Prostomatea / Prorodontida / Holophryidae / Cryptocar
Eukaryota / Alveolata / Ciliophora / Intramacronucleata / Prostomatea / Prorodontida / Holophryidae / Cryptocar
Eukaryota / Alveolata / Ciliophora / Intramacronucleata / Prostomatea / Prorodontida / Holophryidae / Pelagothri
Eukaryota / Alveolata / Ciliophora / Intramacronucleata / Prostomatea / Prorodontida / Prorodontidae / Prorodor
Eukaryota / Alveolata / Ciliophora / Intramacronucleata / Prostomatea / Prorodontida / Prorodontidae / Prorodor
Eukaryota / Alveolata / Ciliophora / Intramacronucleata / Prostomatea / Prorodontida / Prorodontidae / Prorodor
Eukaryota / Alveolata / Ciliophora / Intramacronucleata / Spirotrichea / Choreotrichia / Choreotrichida / Lynnellid
Eukaryota / Alveolata / Ciliophora / Intramacronucleata / Spirotrichea / Choreotrichia / Choreotrichida / Lynnellid
Eukaryota / Alveolata / Ciliophora / Intramacronucleata / Spirotrichea / Choreotrichia / Choreotrichida / Lynnellid
Eukaryota / Alveolata / Ciliophora / Intramacronucleata / Spirotrichea / Choreotrichia / Choreotrichida / Strobilidi
Eukaryota / Alveolata / Ciliophora / Intramacronucleata / Spirotrichea / Choreotrichia / Choreotrichida / Strobilidi
Eukaryota / Alveolata / Ciliophora / Intramacronucleata / Spirotrichea / Choreotrichia / Choreotrichida / Strobilidi
Eukaryota / Alveolata / Ciliophora / Intramacronucleata / Spirotrichea / Choreotrichia / Choreotrichida / Strobilidi
Eukaryota / Alveolata / Ciliophora / Intramacronucleata / Spirotrichea / Choreotrichia / Choreotrichida / Strobilidi
Eukaryota / Alveolata / Ciliophora / Intramacronucleata / Spirotrichea / Choreotrichia / Choreotrichida / Strobilidi
Eukaryota / Alveolata / Ciliophora / Intramacronucleata / Spirotrichea / Choreotrichia / Choreotrichida / Strobilidi
Eukaryota / Alveolata / Ciliophora / Intramacronucleata / Spirotrichea / Choreotrichia / Choreotrichida / Strobilidi
Eukaryota / Alveolata / Ciliophora / Intramacronucleata / Spirotrichea / Choreotrichia / Choreotrichida / Strobilidi
Eukaryota / Alveolata / Ciliophora / Intramacronucleata / Spirotrichea / Choreotrichia / Choreotrichida / Strobilidi
Eukaryota / Alveolata / Ciliophora / Intramacronucleata / Spirotrichea / Choreotrichia / Choreotrichida / Strobilidi
Eukaryota / Alveolata / Ciliophora / Intramacronucleata / Spirotrichea / Choreotrichia / Choreotrichida / Strobilidi
Eukaryota / Alveolata / Ciliophora / Intramacronucleata / Spirotrichea / Choreotrichia / Choreotrichida / Strobilidi
Eukaryota / Alveolata / Ciliophora / Intramacronucleata / Spirotrichea / Choreotrichia / Choreotrichida / Strobilidi
Eukaryota / Alveolata / Ciliophora / Intramacronucleata / Spirotrichea / Choreotrichia / Choreotrichida / Strobilidi
Eukaryota / Alveolata / Ciliophora / Intramacronucleata / Spirotrichea / Choreotrichia / Choreotrichida / Strobilidi
Eukaryota / Alveolata / Ciliophora / Intramacronucleata / Spirotrichea / Choreotrichia / Choreotrichida / Strobilidi
Eukaryota / Alveolata / Ciliophora / Intramacronucleata / Spirotrichea / Choreotrichia / Choreotrichida / Strobilidi
Eukaryota / Alveolata / Ciliophora / Intramacronucleata / Spirotrichea / Choreotrichia / Choreotrichida / Strobilidi
Eukaryota / Alveolata / Ciliophora / Intramacronucleata / Spirotrichea / Choreotrichia / Choreotrichida / Strobilidi
Eukaryota / Alveolata / Ciliophora / Intramacronucleata / Spirotrichea / Choreotrichia / Choreotrichida / Strobilidi
Eukaryota / Alveolata / Ciliophora / Intramacronucleata / Spirotrichea / Choreotrichia / Choreotrichida / Strobilidi
Eukaryota / Alveolata / Ciliophora / Intramacronucleata / Spirotrichea / Choreotrichia / Choreotrichida / Strobilidi
Eukaryota / Alveolata / Ciliophora / Intramacronucleata / Spirotrichea / Choreotrichia / Choreotrichida / Strobilidi
Eukaryota / Alveolata / Ciliophora / Intramacronucleata / Spirotrichea / Choreotrichia / Choreotrichida / Strobilidi
Eukaryota / Alveolata / Ciliophora / Intramacronucleata / Spirotrichea / Choreotrichia / Choreotrichida / Strobilidi
Eukaryota / Alveolata / Ciliophora / Intramacronucleata / Spirotrichea / Choreotrichia / Choreotrichida / Strobilidi
Eukaryota / Alveolata / Ciliophora / Intramacronucleata / Spirotrichea / Choreotrichia / Choreotrichida / Strobilidi
Eukaryota / Alveolata / Ciliophora / Intramacronucleata / Spirotrichea / Choreotrichia / Choreotrichida / Strobilidi
Eukaryota / Alveolata / Ciliophora / Intramacronucleata / Spirotrichea / Choreotrichia / Choreotrichida / Strobilidi
Eukaryota / Alveolata / Ciliophora / Intramacronucleata / Spirotrichea / Choreotrichia / Choreotrichida / Strobilidi
Eukaryota / Alveolata / Ciliophora / Intramacronucleata / Spirotrichea / Choreotrichia / Choreotrichida / Strobilidi
Eukaryota / Alveolata / Ciliophora / Intramacronucleata / Spirotrichea / Choreotrichia / Choreotrichida / Strobilidi
Eukaryota / Alveolata / Ciliophora / Intramacronucleata / Spirotrichea / Choreotrichia / Choreotrichida / Strobilidi
Eukaryota / Alveolata / Ciliophora / Intramacronucleata / Spirotrichea / Choreotrichia / Choreotrichida / Strobilidi
Eukaryota / Alveolata / Ciliophora / Intramacronucleata / Spirotrichea / Choreotrichia / Choreotrichida / Strobilidi

Eukaryota / Alveolata / Ciliophora / Intramacronucleata / Spirotrichea / Choreotrichia / Choreotrichida / Strobilidi
Eukaryota / Alveolata / Ciliophora / Intramacronucleata / Spirotrichea / Choreotrichia / Choreotrichida / Strobilidi
Eukaryota / Alveolata / Ciliophora / Intramacronucleata / Spirotrichea / Choreotrichia / Choreotrichida / Strobilidi
Eukaryota / Alveolata / Ciliophora / Intramacronucleata / Spirotrichea / Choreotrichia / Choreotrichida / Strobilidi
Eukaryota / Alveolata / Ciliophora / Intramacronucleata / Spirotrichea / Choreotrichia / Choreotrichida / Strobilidi
Eukaryota / Alveolata / Ciliophora / Intramacronucleata / Spirotrichea / Choreotrichia / Choreotrichida / Strobilidi
Eukaryota / Alveolata / Ciliophora / Intramacronucleata / Spirotrichea / Choreotrichia / Choreotrichida / Strobilidi
Eukaryota / Alveolata / Ciliophora / Intramacronucleata / Spirotrichea / Choreotrichia / Choreotrichida / Strobilidi
Eukaryota / Alveolata / Ciliophora / Intramacronucleata / Spirotrichea / Choreotrichia / Choreotrichida / Strobilidi
Eukaryota / Alveolata / Ciliophora / Intramacronucleata / Spirotrichea / Choreotrichia / Choreotrichida / Strobilidi
Eukaryota / Alveolata / Ciliophora / Intramacronucleata / Spirotrichea / Choreotrichia / Choreotrichida / Strobilidi
Eukaryota / Alveolata / Ciliophora / Intramacronucleata / Spirotrichea / Choreotrichia / Choreotrichida / Strobilidi
Eukaryota / Alveolata / Ciliophora / Intramacronucleata / Spirotrichea / Choreotrichia / Choreotrichida / Strobilidi
Eukaryota / Alveolata / Ciliophora / Intramacronucleata / Spirotrichea / Choreotrichia / Choreotrichida / Strobilidi
Eukaryota / Alveolata / Ciliophora / Intramacronucleata / Spirotrichea / Choreotrichia / Choreotrichida / Strobilidi
Eukaryota / Alveolata / Ciliophora / Intramacronucleata / Spirotrichea / Choreotrichia / Choreotrichida / Strobilidi
Eukaryota / Alveolata / Ciliophora / Intramacronucleata / Spirotrichea / Choreotrichia / Choreotrichida / Strobilidi
Eukaryota / Alveolata / Ciliophora / Intramacronucleata / Spirotrichea / Choreotrichia / Choreotrichida / Strobilidi
Eukaryota / Alveolata / Ciliophora / Intramacronucleata / Spirotrichea / Choreotrichia / Choreotrichida / Strobilidi
Eukaryota / Alveolata / Ciliophora / Intramacronucleata / Spirotrichea / Choreotrichia / Choreotrichida / Strobilidi
Eukaryota / Alveolata / Ciliophora / Intramacronucleata / Spirotrichea / Choreotrichia / Choreotrichida / Strobilidi
Eukaryota / Alveolata / Ciliophora / Intramacronucleata / Spirotrichea / Choreotrichia / Choreotrichida / Strobilidi
Eukaryota / Alveolata / Ciliophora / Intramacronucleata / Spirotrichea / Choreotrichia / Choreotrichida / Strobilidi
Eukaryota / Alveolata / Ciliophora / Intramacronucleata / Spirotrichea / Choreotrichia / Choreotrichida / Strobilidi
Eukaryota / Alveolata / Ciliophora / Intramacronucleata / Spirotrichea / Choreotrichia / Choreotrichida / Strobilidi
Eukaryota / Alveolata / Ciliophora / Intramacronucleata / Spirotrichea / Choreotrichia / Choreotrichida / Strobilidi
Eukaryota / Alveolata / Ciliophora / Intramacronucleata / Spirotrichea / Choreotrichia / Choreotrichida / Strobilidi
Eukaryota / Alveolata / Ciliophora / Intramacronucleata / Spirotrichea / Choreotrichia / Choreotrichida / Strobilidi
Eukaryota / Alveolata / Ciliophora / Intramacronucleata / Spirotrichea / Choreotrichia / Choreotrichida / Strobilidi
Eukaryota / Alveolata / Ciliophora / Intramacronucleata / Spirotrichea / Choreotrichia / Choreotrichida / Strobilidi
Eukaryota / Alveolata / Ciliophora / Intramacronucleata / Spirotrichea / Choreotrichia / Choreotrichida / Strobilidi
Eukaryota / Alveolata / Ciliophora / Intramacronucleata / Spirotrichea / Choreotrichia / Choreotrichida / Strobilidi
Eukaryota / Alveolata / Ciliophora / Intramacronucleata / Spirotrichea / Choreotrichia / Choreotrichida / Strobilidi
Eukaryota / Alveolata / Ciliophora / Intramacronucleata / Spirotrichea / Choreotrichia / Choreotrichida / Strobilidi
Eukaryota / Alveolata / Ciliophora / Intramacronucleata / Spirotrichea / Choreotrichia / Choreotrichida / Strobilidi
Eukaryota / Alveolata / Ciliophora / Intramacronucleata / Spirotrichea / Choreotrichia / Choreotrichida / Strobilidi
Eukaryota / Alveolata / Ciliophora / Intramacronucleata / Spirotrichea / Choreotrichia / Choreotrichida / Strobilidi
Eukaryota / Alveolata / Ciliophora / Intramacronucleata / Spirotrichea / Choreotrichia / Choreotrichida / Strobilidi
Eukaryota / Alveolata / Ciliophora / Intramacronucleata / Spirotrichea / Choreotrichia / Choreotrichida / Strobilidi
Eukaryota / Alveolata / Ciliophora / Intramacronucleata / Spirotrichea / Choreotrichia / Choreotrichida / Strobilidi
Eukaryota / Alveolata / Ciliophora / Intramacronucleata / Spirotrichea / Choreotrichia / Choreotrichida / Strobilidi
Eukaryota / Alveolata / Ciliophora / Intramacronucleata / Spirotrichea / Choreotrichia / Choreotrichida / Strobilidi
Eukaryota / Alveolata / Ciliophora / Intramacronucleata / Spirotrichea / Choreotrichia / Choreotrichida / Strobilidi
Eukaryota / Alveolata / Ciliophora / Intramacronucleata / Spirotrichea / Choreotrichia / Choreotrichida / Strobilidi
Eukaryota / Alveolata / Ciliophora / Intramacronucleata / Spirotrichea / Choreotrichia / Choreotrichida / Strobilidi
Eukaryota / Alveolata / Ciliophora / Intramacronucleata / Spirotrichea / Choreotrichia / Choreotrichida / Strobilidi
Eukaryota / Alveolata / Ciliophora / Intramacronucleata / Spirotrichea / Choreotrichia / Choreotrichida / Strobilidi
Eukaryota / Alveolata / Ciliophora / Intramacronucleata / Spirotrichea / Choreotrichia / Choreotrichida / Strobilidi

Eukaryota / Alveolata / Ciliophora / Intramacronucleata / Spirotrichea / Choreotrichia / Choreotrichida / Strobilidi
Eukaryota / Alveolata / Ciliophora / Intramacronucleata / Spirotrichea / Choreotrichia / Choreotrichida / Strobilidi
Eukaryota / Alveolata / Ciliophora / Intramacronucleata / Spirotrichea / Choreotrichia / Choreotrichida / Strobilidi
Eukaryota / Alveolata / Ciliophora / Intramacronucleata / Spirotrichea / Choreotrichia / Choreotrichida / Strobilidi
Eukaryota / Alveolata / Ciliophora / Intramacronucleata / Spirotrichea / Choreotrichia / Choreotrichida / Strobilidi
Eukaryota / Alveolata / Ciliophora / Intramacronucleata / Spirotrichea / Choreotrichia / Choreotrichida / Strobilidi
Eukaryota / Alveolata / Ciliophora / Intramacronucleata / Spirotrichea / Choreotrichia / Choreotrichida / Strobilidi
Eukaryota / Alveolata / Ciliophora / Intramacronucleata / Spirotrichea / Choreotrichia / Choreotrichida / Strobilidi
Eukaryota / Alveolata / Ciliophora / Intramacronucleata / Spirotrichea / Choreotrichia / Choreotrichida / Strobilidi
Eukaryota / Alveolata / Ciliophora / Intramacronucleata / Spirotrichea / Choreotrichia / Choreotrichida / Strobilidi
Eukaryota / Alveolata / Ciliophora / Intramacronucleata / Spirotrichea / Choreotrichia / Choreotrichida / Strobilidi
Eukaryota / Alveolata / Ciliophora / Intramacronucleata / Spirotrichea / Choreotrichia / Choreotrichida / Strobilidi
Eukaryota / Alveolata / Ciliophora / Intramacronucleata / Spirotrichea / Choreotrichia / Choreotrichida / Strobilidi
Eukaryota / Alveolata / Ciliophora / Intramacronucleata / Spirotrichea / Choreotrichia / Choreotrichida / Strobilidi
Eukaryota / Alveolata / Ciliophora / Intramacronucleata / Spirotrichea / Choreotrichia / Choreotrichida / Strobilidi
Eukaryota / Alveolata / Ciliophora / Intramacronucleata / Spirotrichea / Choreotrichia / Choreotrichida / Strobilidi
Eukaryota / Alveolata / Ciliophora / Intramacronucleata / Spirotrichea / Choreotrichia / Choreotrichida / Strobilidi
Eukaryota / Alveolata / Ciliophora / Intramacronucleata / Spirotrichea / Choreotrichia / Choreotrichida / Strobilidi
Eukaryota / Alveolata / Ciliophora / Intramacronucleata / Spirotrichea / Choreotrichia / Choreotrichida / Strombid
Eukaryota / Alveolata / Ciliophora / Intramacronucleata / Spirotrichea / Choreotrichia / Choreotrichida / Strombid
Eukaryota / Alveolata / Ciliophora / Intramacronucleata / Spirotrichea / Choreotrichia / Choreotrichida / Strombid
Eukaryota / Alveolata / Ciliophora / Intramacronucleata / Spirotrichea / Choreotrichia / Choreotrichida / Strombid
Eukaryota / Alveolata / Ciliophora / Intramacronucleata / Spirotrichea / Choreotrichia / Choreotrichida / Strombid
Eukaryota / Alveolata / Ciliophora / Intramacronucleata / Spirotrichea / Choreotrichia / Choreotrichida / Strombid
Eukaryota / Alveolata / Ciliophora / Intramacronucleata / Spirotrichea / Choreotrichia / Choreotrichida / Strombid
Eukaryota / Alveolata / Ciliophora / Intramacronucleata / Spirotrichea / Choreotrichia / Choreotrichida / Strombid
Eukaryota / Alveolata / Ciliophora / Intramacronucleata / Spirotrichea / Choreotrichia / Choreotrichida / Strombid
Eukaryota / Alveolata / Ciliophora / Intramacronucleata / Spirotrichea / Choreotrichia / Choreotrichida / Strombid
Eukaryota / Alveolata / Ciliophora / Intramacronucleata / Spirotrichea / Choreotrichia / Choreotrichida / Strombid
Eukaryota / Alveolata / Ciliophora / Intramacronucleata / Spirotrichea / Choreotrichia / Tintinnida / Codonellidae
Eukaryota / Alveolata / Ciliophora / Intramacronucleata / Spirotrichea / Choreotrichia / Tintinnida / Codonellidae
Eukaryota / Alveolata / Ciliophora / Intramacronucleata / Spirotrichea / Choreotrichia / Tintinnida / Codonellidae
Eukaryota / Alveolata / Ciliophora / Intramacronucleata / Spirotrichea / Choreotrichia / Tintinnida / Codonellidae
Eukaryota / Alveolata / Ciliophora / Intramacronucleata / Spirotrichea / Choreotrichia / Tintinnida / Codonellidae
Eukaryota / Alveolata / Ciliophora / Intramacronucleata / Spirotrichea / Choreotrichia / Tintinnida / Codonellidae
Eukaryota / Alveolata / Ciliophora / Intramacronucleata / Spirotrichea / Choreotrichia / Tintinnida / Codonellidae
Eukaryota / Alveolata / Ciliophora / Intramacronucleata / Spirotrichea / Choreotrichia / Tintinnida / Codonellidae
Eukaryota / Alveolata / Ciliophora / Intramacronucleata / Spirotrichea / Choreotrichia / Tintinnida / Codonellidae
Eukaryota / Alveolata / Ciliophora / Intramacronucleata / Spirotrichea / Choreotrichia / Tintinnida / Codonellidae
Eukaryota / Alveolata / Ciliophora / Intramacronucleata / Spirotrichea / Choreotrichia / Tintinnida / Codonellidae
Eukaryota / Alveolata / Ciliophora / Intramacronucleata / Spirotrichea / Choreotrichia / Tintinnida / Codonellidae
Eukaryota / Alveolata / Ciliophora / Intramacronucleata / Spirotrichea / Choreotrichia / Tintinnida / Codonellidae
Eukaryota / Alveolata / Ciliophora / Intramacronucleata / Spirotrichea / Choreotrichia / Tintinnida / Codonellidae
Eukaryota / Alveolata / Ciliophora / Intramacronucleata / Spirotrichea / Choreotrichia / Tintinnida / Codonellidae
Eukaryota / Alveolata / Ciliophora / Intramacronucleata / Spirotrichea / Choreotrichia / Tintinnida / Codonellidae
Eukaryota / Alveolata / Ciliophora / Intramacronucleata / Spirotrichea / Choreotrichia / Tintinnida / Codonellidae
Eukaryota / Alveolata / Ciliophora / Intramacronucleata / Spirotrichea / Choreotrichia / Tintinnida / Codonellidae
Eukaryota / Alveolata / Ciliophora / Intramacronucleata / Spirotrichea / Choreotrichia / Tintinnida / Codonellidae

Eukaryota / Alveolata / Ciliophora / Intramacronucleata / Spirotrichea / Choreotrichia / Tintinnida / Codonellopsic
Eukaryota / Alveolata / Ciliophora / Intramacronucleata / Spirotrichea / Choreotrichia / Tintinnida / Codonellopsic
Eukaryota / Alveolata / Ciliophora / Intramacronucleata / Spirotrichea / Choreotrichia / Tintinnida / Codonellopsic
Eukaryota / Alveolata / Ciliophora / Intramacronucleata / Spirotrichea / Choreotrichia / Tintinnida / Codonellopsic
Eukaryota / Alveolata / Ciliophora / Intramacronucleata / Spirotrichea / Choreotrichia / Tintinnida / Codonellopsic
Eukaryota / Alveolata / Ciliophora / Intramacronucleata / Spirotrichea / Choreotrichia / Tintinnida / Codonellopsic
Eukaryota / Alveolata / Ciliophora / Intramacronucleata / Spirotrichea / Choreotrichia / Tintinnida / Codonellopsic
Eukaryota / Alveolata / Ciliophora / Intramacronucleata / Spirotrichea / Choreotrichia / Tintinnida / Codonellopsic
Eukaryota / Alveolata / Ciliophora / Intramacronucleata / Spirotrichea / Choreotrichia / Tintinnida / Codonellopsic
Eukaryota / Alveolata / Ciliophora / Intramacronucleata / Spirotrichea / Choreotrichia / Tintinnida / Codonellopsic
Eukaryota / Alveolata / Ciliophora / Intramacronucleata / Spirotrichea / Choreotrichia / Tintinnida / Codonellopsic
Eukaryota / Alveolata / Ciliophora / Intramacronucleata / Spirotrichea / Choreotrichia / Tintinnida / Codonellopsic
Eukaryota / Alveolata / Ciliophora / Intramacronucleata / Spirotrichea / Choreotrichia / Tintinnida / Codonellopsic
Eukaryota / Alveolata / Ciliophora / Intramacronucleata / Spirotrichea / Choreotrichia / Tintinnida / Cyttarocylidid
Eukaryota / Alveolata / Ciliophora / Intramacronucleata / Spirotrichea / Choreotrichia / Tintinnida / Epiplocylidida
Eukaryota / Alveolata / Ciliophora / Intramacronucleata / Spirotrichea / Choreotrichia / Tintinnida / Metacylididae
Eukaryota / Alveolata / Ciliophora / Intramacronucleata / Spirotrichea / Choreotrichia / Tintinnida / Metacylididae
Eukaryota / Alveolata / Ciliophora / Intramacronucleata / Spirotrichea / Choreotrichia / Tintinnida / Metacylididae
Eukaryota / Alveolata / Ciliophora / Intramacronucleata / Spirotrichea / Choreotrichia / Tintinnida / Metacylididae
Eukaryota / Alveolata / Ciliophora / Intramacronucleata / Spirotrichea / Choreotrichia / Tintinnida / Metacylididae
Eukaryota / Alveolata / Ciliophora / Intramacronucleata / Spirotrichea / Choreotrichia / Tintinnida / Metacylididae
Eukaryota / Alveolata / Ciliophora / Intramacronucleata / Spirotrichea / Choreotrichia / Tintinnida / Metacylididae
Eukaryota / Alveolata / Ciliophora / Intramacronucleata / Spirotrichea / Choreotrichia / Tintinnida / Metacylididae
Eukaryota / Alveolata / Ciliophora / Intramacronucleata / Spirotrichea / Choreotrichia / Tintinnida / Metacylididae
Eukaryota / Alveolata / Ciliophora / Intramacronucleata / Spirotrichea / Choreotrichia / Tintinnida / Metacylididae
Eukaryota / Alveolata / Ciliophora / Intramacronucleata / Spirotrichea / Choreotrichia / Tintinnida / Metacylididae
Eukaryota / Alveolata / Ciliophora / Intramacronucleata / Spirotrichea / Choreotrichia / Tintinnida / Metacylididae
Eukaryota / Alveolata / Ciliophora / Intramacronucleata / Spirotrichea / Choreotrichia / Tintinnida / Ptychocylidida
Eukaryota / Alveolata / Ciliophora / Intramacronucleata / Spirotrichea / Choreotrichia / Tintinnida / Ptychocylidida
Eukaryota / Alveolata / Ciliophora / Intramacronucleata / Spirotrichea / Choreotrichia / Tintinnida / Ptychocylidida
Eukaryota / Alveolata / Ciliophora / Intramacronucleata / Spirotrichea / Choreotrichia / Tintinnida / Ptychocylidida
Eukaryota / Alveolata / Ciliophora / Intramacronucleata / Spirotrichea / Choreotrichia / Tintinnida / Ptychocylidida
Eukaryota / Alveolata / Ciliophora / Intramacronucleata / Spirotrichea / Choreotrichia / Tintinnida / Ptychocylidida
Eukaryota / Alveolata / Ciliophora / Intramacronucleata / Spirotrichea / Choreotrichia / Tintinnida / Ptychocylidida
Eukaryota / Alveolata / Ciliophora / Intramacronucleata / Spirotrichea / Choreotrichia / Tintinnida / Ptychocylidida
Eukaryota / Alveolata / Ciliophora / Intramacronucleata / Spirotrichea / Choreotrichia / Tintinnida / Ptychocylidida
Eukaryota / Alveolata / Ciliophora / Intramacronucleata / Spirotrichea / Choreotrichia / Tintinnida / Ptychocylidida
Eukaryota / Alveolata / Ciliophora / Intramacronucleata / Spirotrichea / Choreotrichia / Tintinnida / Ptychocylidida
Eukaryota / Alveolata / Ciliophora / Intramacronucleata / Spirotrichea / Choreotrichia / Tintinnida / Ptychocylidida
Eukaryota / Alveolata / Ciliophora / Intramacronucleata / Spirotrichea / Choreotrichia / Tintinnida / Ptychocylidida
Eukaryota / Alveolata / Ciliophora / Intramacronucleata / Spirotrichea / Choreotrichia / Tintinnida / Ptychocylidida
Eukaryota / Alveolata / Ciliophora / Intramacronucleata / Spirotrichea / Choreotrichia / Tintinnida / Ptychocylidida
Eukaryota / Alveolata / Ciliophora / Intramacronucleata / Spirotrichea / Choreotrichia / Tintinnida / Ptychocylidida
Eukaryota / Alveolata / Ciliophora / Intramacronucleata / Spirotrichea / Choreotrichia / Tintinnida / Rhabdonellida
Eukaryota / Alveolata / Ciliophora / Intramacronucleata / Spirotrichea / Choreotrichia / Tintinnida / Rhabdonellida
Eukaryota / Alveolata / Ciliophora / Intramacronucleata / Spirotrichea / Choreotrichia / Tintinnida / Rhabdonellida
Eukaryota / Alveolata / Ciliophora / Intramacronucleata / Spirotrichea / Choreotrichia / Tintinnida / Rhabdonellida
Eukaryota / Alveolata / Ciliophora / Intramacronucleata / Spirotrichea / Choreotrichia / Tintinnida / Rhabdonellida

Eukaryota / Alveolata / Ciliophora / Intramacronucleata / Spirotrichea / Choreotrichia / Tintinnida / Rhabdonellida
Eukaryota / Alveolata / Ciliophora / Intramacronucleata / Spirotrichea / Choreotrichia / Tintinnida / Rhabdonellida
Eukaryota / Alveolata / Ciliophora / Intramacronucleata / Spirotrichea / Choreotrichia / Tintinnida / Rhabdonellida
Eukaryota / Alveolata / Ciliophora / Intramacronucleata / Spirotrichea / Choreotrichia / Tintinnida / Rhabdonellida
Eukaryota / Alveolata / Ciliophora / Intramacronucleata / Spirotrichea / Choreotrichia / Tintinnida / Rhabdonellida
Eukaryota / Alveolata / Ciliophora / Intramacronucleata / Spirotrichea / Choreotrichia / Tintinnida / Rhabdonellida
Eukaryota / Alveolata / Ciliophora / Intramacronucleata / Spirotrichea / Choreotrichia / Tintinnida / Rhabdonellida
Eukaryota / Alveolata / Ciliophora / Intramacronucleata / Spirotrichea / Choreotrichia / Tintinnida / Rhabdonellida
Eukaryota / Alveolata / Ciliophora / Intramacronucleata / Spirotrichea / Choreotrichia / Tintinnida / Rhabdonellida
Eukaryota / Alveolata / Ciliophora / Intramacronucleata / Spirotrichea / Choreotrichia / Tintinnida / Tintinnidae / A
Eukaryota / Alveolata / Ciliophora / Intramacronucleata / Spirotrichea / Choreotrichia / Tintinnida / Tintinnidae / A
Eukaryota / Alveolata / Ciliophora / Intramacronucleata / Spirotrichea / Choreotrichia / Tintinnida / Tintinnidae / A
Eukaryota / Alveolata / Ciliophora / Intramacronucleata / Spirotrichea / Choreotrichia / Tintinnida / Tintinnidae / A
Eukaryota / Alveolata / Ciliophora / Intramacronucleata / Spirotrichea / Choreotrichia / Tintinnida / Tintinnidae / A
Eukaryota / Alveolata / Ciliophora / Intramacronucleata / Spirotrichea / Choreotrichia / Tintinnida / Tintinnidae / A
Eukaryota / Alveolata / Ciliophora / Intramacronucleata / Spirotrichea / Choreotrichia / Tintinnida / Tintinnidae / A
Eukaryota / Alveolata / Ciliophora / Intramacronucleata / Spirotrichea / Choreotrichia / Tintinnida / Tintinnidae / A
Eukaryota / Alveolata / Ciliophora / Intramacronucleata / Spirotrichea / Choreotrichia / Tintinnida / Tintinnidae / A
Eukaryota / Alveolata / Ciliophora / Intramacronucleata / Spirotrichea / Choreotrichia / Tintinnida / Tintinnidae / A
Eukaryota / Alveolata / Ciliophora / Intramacronucleata / Spirotrichea / Choreotrichia / Tintinnida / Tintinnidae / A
Eukaryota / Alveolata / Ciliophora / Intramacronucleata / Spirotrichea / Choreotrichia / Tintinnida / Tintinnidae / A
Eukaryota / Alveolata / Ciliophora / Intramacronucleata / Spirotrichea / Choreotrichia / Tintinnida / Tintinnidae / A
Eukaryota / Alveolata / Ciliophora / Intramacronucleata / Spirotrichea / Choreotrichia / Tintinnida / Tintinnidae / A
Eukaryota / Alveolata / Ciliophora / Intramacronucleata / Spirotrichea / Choreotrichia / Tintinnida / Tintinnidae / A
Eukaryota / Alveolata / Ciliophora / Intramacronucleata / Spirotrichea / Choreotrichia / Tintinnida / Tintinnidae / A
Eukaryota / Alveolata / Ciliophora / Intramacronucleata / Spirotrichea / Choreotrichia / Tintinnida / Tintinnidae / A
Eukaryota / Alveolata / Ciliophora / Intramacronucleata / Spirotrichea / Choreotrichia / Tintinnida / Tintinnidae / A
Eukaryota / Alveolata / Ciliophora / Intramacronucleata / Spirotrichea / Choreotrichia / Tintinnida / Tintinnidae / A
Eukaryota / Alveolata / Ciliophora / Intramacronucleata / Spirotrichea / Choreotrichia / Tintinnida / Tintinnidae / B
Eukaryota / Alveolata / Ciliophora / Intramacronucleata / Spirotrichea / Choreotrichia / Tintinnida / Tintinnidae / B
Eukaryota / Alveolata / Ciliophora / Intramacronucleata / Spirotrichea / Choreotrichia / Tintinnida / Tintinnidae / B
Eukaryota / Alveolata / Ciliophora / Intramacronucleata / Spirotrichea / Choreotrichia / Tintinnida / Tintinnidae / B
Eukaryota / Alveolata / Ciliophora / Intramacronucleata / Spirotrichea / Choreotrichia / Tintinnida / Tintinnidae / B
Eukaryota / Alveolata / Ciliophora / Intramacronucleata / Spirotrichea / Choreotrichia / Tintinnida / Tintinnidae / B
Eukaryota / Alveolata / Ciliophora / Intramacronucleata / Spirotrichea / Choreotrichia / Tintinnida / Tintinnidae / B
Eukaryota / Alveolata / Ciliophora / Intramacronucleata / Spirotrichea / Choreotrichia / Tintinnida / Tintinnidae / B
Eukaryota / Alveolata / Ciliophora / Intramacronucleata / Spirotrichea / Choreotrichia / Tintinnida / Tintinnidae / B
Eukaryota / Alveolata / Ciliophora / Intramacronucleata / Spirotrichea / Choreotrichia / Tintinnida / Tintinnidae / B
Eukaryota / Alveolata / Ciliophora / Intramacronucleata / Spirotrichea / Choreotrichia / Tintinnida / Tintinnidae / S
Eukaryota / Alveolata / Ciliophora / Intramacronucleata / Spirotrichea / Choreotrichia / Tintinnida / Tintinnidae / S
Eukaryota / Alveolata / Ciliophora / Intramacronucleata / Spirotrichea / Choreotrichia / Tintinnida / Tintinnidae / S
Eukaryota / Alveolata / Ciliophora / Intramacronucleata / Spirotrichea / Choreotrichia / Tintinnida / Tintinnidae / S
Eukaryota / Alveolata / Ciliophora / Intramacronucleata / Spirotrichea / Choreotrichia / Tintinnida / Tintinnidae / S
Eukaryota / Alveolata / Ciliophora / Intramacronucleata / Spirotrichea / Choreotrichia / Tintinnida / Tintinnidae / S
Eukaryota / Alveolata / Ciliophora / Intramacronucleata / Spirotrichea / Choreotrichia / Tintinnida / Tintinnidae / S
Eukaryota / Alveolata / Ciliophora / Intramacronucleata / Spirotrichea / Choreotrichia / Tintinnida / Tintinnidae / S
Eukaryota / Alveolata / Ciliophora / Intramacronucleata / Spirotrichea / Choreotrichia / Tintinnida / Tintinnidae / S
Eukaryota / Alveolata / Ciliophora / Intramacronucleata / Spirotrichea / Choreotrichia / Tintinnida / Tintinnidae / S

Eukaryota / Alveolata / Ciliophora / Intramacronucleata / Spirotrichea / Choreotrichia / Tintinnida / Tintinnidae / S
Eukaryota / Alveolata / Ciliophora / Intramacronucleata / Spirotrichea / Choreotrichia / Tintinnida / Tintinnidae / S
Eukaryota / Alveolata / Ciliophora / Intramacronucleata / Spirotrichea / Choreotrichia / Tintinnida / Tintinnidae / S
Eukaryota / Alveolata / Ciliophora / Intramacronucleata / Spirotrichea / Choreotrichia / Tintinnida / Tintinnidae / S
Eukaryota / Alveolata / Ciliophora / Intramacronucleata / Spirotrichea / Choreotrichia / Tintinnida / Tintinnidae / S
Eukaryota / Alveolata / Ciliophora / Intramacronucleata / Spirotrichea / Choreotrichia / Tintinnida / Tintinnidae / S
Eukaryota / Alveolata / Ciliophora / Intramacronucleata / Spirotrichea / Choreotrichia / Tintinnida / Tintinnidae / S
Eukaryota / Alveolata / Ciliophora / Intramacronucleata / Spirotrichea / Choreotrichia / Tintinnida / Tintinnidae / S
Eukaryota / Alveolata / Ciliophora / Intramacronucleata / Spirotrichea / Choreotrichia / Tintinnida / Tintinnidae / S
Eukaryota / Alveolata / Ciliophora / Intramacronucleata / Spirotrichea / Choreotrichia / Tintinnida / Tintinnidae / S
Eukaryota / Alveolata / Ciliophora / Intramacronucleata / Spirotrichea / Choreotrichia / Tintinnida / Tintinnidae / S
Eukaryota / Alveolata / Ciliophora / Intramacronucleata / Spirotrichea / Choreotrichia / Tintinnida / Tintinnidae / T
Eukaryota / Alveolata / Ciliophora / Intramacronucleata / Spirotrichea / Choreotrichia / Tintinnida / Tintinnidae / T
Eukaryota / Alveolata / Ciliophora / Intramacronucleata / Spirotrichea / Choreotrichia / Tintinnida / Undellidae / U
Eukaryota / Alveolata / Ciliophora / Intramacronucleata / Spirotrichea / Choreotrichia / Tintinnida / Xystonellidae
Eukaryota / Alveolata / Ciliophora / Intramacronucleata / Spirotrichea / Choreotrichia / Tintinnida / Xystonellidae
Eukaryota / Alveolata / Ciliophora / Intramacronucleata / Spirotrichea / Choreotrichia / Tintinnida / Xystonellidae
Eukaryota / Alveolata / Ciliophora / Intramacronucleata / Spirotrichea / Choreotrichia / Tintinnida / Xystonellidae
Eukaryota / Alveolata / Ciliophora / Intramacronucleata / Spirotrichea / Choreotrichia / Tintinnida / Xystonellidae
Eukaryota / Alveolata / Ciliophora / Intramacronucleata / Spirotrichea / Choreotrichia / Tintinnida / Xystonellidae
Eukaryota / Alveolata / Ciliophora / Intramacronucleata / Spirotrichea / Choreotrichia / Tintinnida / Xystonellidae
Eukaryota / Alveolata / Ciliophora / Intramacronucleata / Spirotrichea / Choreotrichia / Tintinnida / Xystonellidae
Eukaryota / Alveolata / Ciliophora / Intramacronucleata / Spirotrichea / Choreotrichia / Tintinnida / Xystonellidae
Eukaryota / Alveolata / Ciliophora / Intramacronucleata / Spirotrichea / Choreotrichia / Tintinnida / Xystonellidae
Eukaryota / Alveolata / Ciliophora / Intramacronucleata / Spirotrichea / Choreotrichia / Tintinnida / Xystonellidae
Eukaryota / Alveolata / Ciliophora / Intramacronucleata / Spirotrichea / Choreotrichia / Tintinnida / Xystonellidae
Eukaryota / Alveolata / Ciliophora / Intramacronucleata / Spirotrichea / Choreotrichia / Tintinnida / Xystonellidae
Eukaryota / Alveolata / Ciliophora / Intramacronucleata / Spirotrichea / Choreotrichia / Tintinnida / Xystonellidae
Eukaryota / Alveolata / Ciliophora / Intramacronucleata / Spirotrichea / Choreotrichia / Tintinnida / Xystonellidae
Eukaryota / Alveolata / Ciliophora / Intramacronucleata / Spirotrichea / Choreotrichia / Tintinnida / Xystonellidae
Eukaryota / Alveolata / Ciliophora / Intramacronucleata / Spirotrichea / Hypotrichia / Euplotida / Euplotidae / Eup
Eukaryota / Alveolata / Ciliophora / Intramacronucleata / Spirotrichea / Hypotrichia / Euplotida / Euplotidae / Eup
Eukaryota / Alveolata / Ciliophora / Intramacronucleata / Spirotrichea / Hypotrichia / Euplotida / Euplotidae / Eup
Eukaryota / Alveolata / Ciliophora / Intramacronucleata / Spirotrichea / Hypotrichia / Euplotida / Euplotidae / Eup
Eukaryota / Alveolata / Ciliophora / Intramacronucleata / Spirotrichea / Hypotrichia / Euplotida / Euplotidae / Eup
Eukaryota / Alveolata / Ciliophora / Intramacronucleata / Spirotrichea / Hypotrichia / Euplotida / Euplotidae / Eup
Eukaryota / Alveolata / Ciliophora / Intramacronucleata / Spirotrichea / Hypotrichia / Euplotida / Euplotidae / Eup
Eukaryota / Alveolata / Ciliophora / Intramacronucleata / Spirotrichea / Hypotrichia / Euplotida / Euplotidae / Eup
Eukaryota / Alveolata / Ciliophora / Intramacronucleata / Spirotrichea / Hypotrichia / Euplotida / Uronychiidae / D
Eukaryota / Alveolata / Ciliophora / Intramacronucleata / Spirotrichea / Hypotrichia / Euplotida / Uronychiidae / D
Eukaryota / Alveolata / Ciliophora / Intramacronucleata / Spirotrichea / Hypotrichia / Euplotida / Uronychiidae / D
Eukaryota / Alveolata / Ciliophora / Intramacronucleata / Spirotrichea / Hypotrichia / Euplotida / Uronychiidae / D
Eukaryota / Alveolata / Ciliophora / Intramacronucleata / Spirotrichea / Hypotrichia / unclassified hypotrichs
Eukaryota / Alveolata / Ciliophora / Intramacronucleata / Spirotrichea / Hypotrichia / unclassified hypotrichs
Eukaryota / Alveolata / Ciliophora / Intramacronucleata / Spirotrichea / Hypotrichia / unclassified hypotrichs
Eukaryota / Alveolata / Ciliophora / Intramacronucleata / Spirotrichea / Hypotrichia / unclassified hypotrichs
Eukaryota / Alveolata / Ciliophora / Intramacronucleata / Spirotrichea / Hypotrichia / unclassified hypotrichs
Eukaryota / Alveolata / Ciliophora / Intramacronucleata / Spirotrichea / Hypotrichia / unclassified hypotrichs

Eukaryota / Alveolata / Ciliophora / Intramacronucleata / Spirotrichea / Hypotrichia / unclassified hypotrichs
Eukaryota / Alveolata / Ciliophora / Intramacronucleata / Spirotrichea / Oligotrichia / Strombidiidae / Apostrombi
Eukaryota / Alveolata / Ciliophora / Intramacronucleata / Spirotrichea / Oligotrichia / Strombidiidae / Laboea
Eukaryota / Alveolata / Ciliophora / Intramacronucleata / Spirotrichea / Oligotrichia / Strombidiidae / Laboea
Eukaryota / Alveolata / Ciliophora / Intramacronucleata / Spirotrichea / Oligotrichia / Strombidiidae / Laboea
Eukaryota / Alveolata / Ciliophora / Intramacronucleata / Spirotrichea / Oligotrichia / Strombidiidae / Laboea
Eukaryota / Alveolata / Ciliophora / Intramacronucleata / Spirotrichea / Oligotrichia / Strombidiidae / Laboea
Eukaryota / Alveolata / Ciliophora / Intramacronucleata / Spirotrichea / Oligotrichia / Strombidiidae / Laboea
Eukaryota / Alveolata / Ciliophora / Intramacronucleata / Spirotrichea / Oligotrichia / Strombidiidae / Laboea
Eukaryota / Alveolata / Ciliophora / Intramacronucleata / Spirotrichea / Oligotrichia / Strombidiidae / Laboea
Eukaryota / Alveolata / Ciliophora / Intramacronucleata / Spirotrichea / Oligotrichia / Strombidiidae / Laboea
Eukaryota / Alveolata / Ciliophora / Intramacronucleata / Spirotrichea / Oligotrichia / Strombidiidae / Laboea
Eukaryota / Alveolata / Ciliophora / Intramacronucleata / Spirotrichea / Oligotrichia / Strombidiidae / Laboea
Eukaryota / Alveolata / Ciliophora / Intramacronucleata / Spirotrichea / Oligotrichia / Strombidiidae / Laboea
Eukaryota / Alveolata / Ciliophora / Intramacronucleata / Spirotrichea / Oligotrichia / Strombidiidae / Laboea
Eukaryota / Alveolata / Ciliophora / Intramacronucleata / Spirotrichea / Oligotrichia / Strombidiidae / Laboea
Eukaryota / Alveolata / Ciliophora / Intramacronucleata / Spirotrichea / Oligotrichia / Strombidiidae / Laboea
Eukaryota / Alveolata / Ciliophora / Intramacronucleata / Spirotrichea / Oligotrichia / Strombidiidae / Laboea
Eukaryota / Alveolata / Ciliophora / Intramacronucleata / Spirotrichea / Oligotrichia / Strombidiidae / Laboea
Eukaryota / Alveolata / Ciliophora / Intramacronucleata / Spirotrichea / Oligotrichia / Strombidiidae / Laboea
Eukaryota / Alveolata / Ciliophora / Intramacronucleata / Spirotrichea / Oligotrichia / Strombidiidae / Laboea
Eukaryota / Alveolata / Ciliophora / Intramacronucleata / Spirotrichea / Oligotrichia / Strombidiidae / Laboea
Eukaryota / Alveolata / Ciliophora / Intramacronucleata / Spirotrichea / Oligotrichia / Strombidiidae / Laboea
Eukaryota / Alveolata / Ciliophora / Intramacronucleata / Spirotrichea / Oligotrichia / Strombidiidae / Laboea
Eukaryota / Alveolata / Ciliophora / Intramacronucleata / Spirotrichea / Oligotrichia / Strombidiidae / Laboea
Eukaryota / Alveolata / Ciliophora / Intramacronucleata / Spirotrichea / Oligotrichia / Strombidiidae / Laboea
Eukaryota / Alveolata / Ciliophora / Intramacronucleata / Spirotrichea / Oligotrichia / Strombidiidae / Laboea
Eukaryota / Alveolata / Ciliophora / Intramacronucleata / Spirotrichea / Oligotrichia / Strombidiidae / Laboea
Eukaryota / Alveolata / Ciliophora / Intramacronucleata / Spirotrichea / Oligotrichia / Strombidiidae / Laboea
Eukaryota / Alveolata / Ciliophora / Intramacronucleata / Spirotrichea / Oligotrichia / Strombidiidae / Laboea
Eukaryota / Alveolata / Ciliophora / Intramacronucleata / Spirotrichea / Oligotrichia / Strombidiidae / Laboea
Eukaryota / Alveolata / Ciliophora / Intramacronucleata / Spirotrichea / Oligotrichia / Strombidiidae / Laboea
Eukaryota / Alveolata / Ciliophora / Intramacronucleata / Spirotrichea / Oligotrichia / Strombidiidae / Laboea
Eukaryota / Alveolata / Ciliophora / Intramacronucleata / Spirotrichea / Oligotrichia / Strombidiidae / Laboea
Eukaryota / Alveolata / Ciliophora / Intramacronucleata / Spirotrichea / Oligotrichia / Strombidiidae / Laboea
Eukaryota / Alveolata / Ciliophora / Intramacronucleata / Spirotrichea / Oligotrichia / Strombidiidae / Laboea
Eukaryota / Alveolata / Ciliophora / Intramacronucleata / Spirotrichea / Oligotrichia / Strombidiidae / Laboea
Eukaryota / Alveolata / Ciliophora / Intramacronucleata / Spirotrichea / Oligotrichia / Strombidiidae / Laboea
Eukaryota / Alveolata / Ciliophora / Intramacronucleata / Spirotrichea / Oligotrichia / Strombidiidae / Laboea
Eukaryota / Alveolata / Ciliophora / Intramacronucleata / Spirotrichea / Oligotrichia / Strombidiidae / Laboea
Eukaryota / Alveolata / Ciliophora / Intramacronucleata / Spirotrichea / Oligotrichia / Strombidiidae / Laboea
Eukaryota / Alveolata / Ciliophora / Intramacronucleata / Spirotrichea / Oligotrichia / Strombidiidae / Laboea
Eukaryota / Alveolata / Ciliophora / Intramacronucleata / Spirotrichea / Oligotrichia / Strombidiidae / Laboea
Eukaryota / Alveolata / Ciliophora / Intramacronucleata / Spirotrichea / Oligotrichia / Strombidiidae / Laboea
Eukaryota / Alveolata / Ciliophora / Intramacronucleata / Spirotrichea / Oligotrichia / Strombidiidae / Laboea
Eukaryota / Alveolata / Ciliophora / Intramacronucleata / Spirotrichea / Oligotrichia / Strombidiidae / Laboea
Eukaryota / Alveolata / Ciliophora / Intramacronucleata / Spirotrichea / Oligotrichia / Strombidiidae / Laboea
Eukaryota / Alveolata / Ciliophora / Intramacronucleata / Spirotrichea / Oligotrichia / Strombidiidae / Laboea

Eukaryota / Alveolata / Ciliophora / Intramacronucleata / Spirotrichea / Oligotrichia / Strombidiidae / Laboea
Eukaryota / Alveolata / Ciliophora / Intramacronucleata / Spirotrichea / Oligotrichia / Strombidiidae / Laboea
Eukaryota / Alveolata / Ciliophora / Intramacronucleata / Spirotrichea / Oligotrichia / Strombidiidae / Laboea
Eukaryota / Alveolata / Ciliophora / Intramacronucleata / Spirotrichea / Oligotrichia / Strombidiidae / Laboea
Eukaryota / Alveolata / Ciliophora / Intramacronucleata / Spirotrichea / Oligotrichia / Strombidiidae / Laboea
Eukaryota / Alveolata / Ciliophora / Intramacronucleata / Spirotrichea / Oligotrichia / Strombidiidae / Laboea
Eukaryota / Alveolata / Ciliophora / Intramacronucleata / Spirotrichea / Oligotrichia / Strombidiidae / Laboea
Eukaryota / Alveolata / Ciliophora / Intramacronucleata / Spirotrichea / Oligotrichia / Strombidiidae / Laboea
Eukaryota / Alveolata / Ciliophora / Intramacronucleata / Spirotrichea / Oligotrichia / Strombidiidae / Laboea
Eukaryota / Alveolata / Ciliophora / Intramacronucleata / Spirotrichea / Oligotrichia / Strombidiidae / Laboea
Eukaryota / Alveolata / Ciliophora / Intramacronucleata / Spirotrichea / Oligotrichia / Strombidiidae / Laboea
Eukaryota / Alveolata / Ciliophora / Intramacronucleata / Spirotrichea / Oligotrichia / Strombidiidae / Laboea
Eukaryota / Alveolata / Ciliophora / Intramacronucleata / Spirotrichea / Oligotrichia / Strombidiidae / Laboea
Eukaryota / Alveolata / Ciliophora / Intramacronucleata / Spirotrichea / Oligotrichia / Strombidiidae / Laboea
Eukaryota / Alveolata / Ciliophora / Intramacronucleata / Spirotrichea / Oligotrichia / Strombidiidae / Laboea
Eukaryota / Alveolata / Ciliophora / Intramacronucleata / Spirotrichea / Oligotrichia / Strombidiidae / Laboea
Eukaryota / Alveolata / Ciliophora / Intramacronucleata / Spirotrichea / Oligotrichia / Strombidiidae / Laboea
Eukaryota / Alveolata / Ciliophora / Intramacronucleata / Spirotrichea / Oligotrichia / Strombidiidae / Laboea
Eukaryota / Alveolata / Ciliophora / Intramacronucleata / Spirotrichea / Oligotrichia / Strombidiidae / Laboea
Eukaryota / Alveolata / Ciliophora / Intramacronucleata / Spirotrichea / Oligotrichia / Strombidiidae / Laboea
Eukaryota / Alveolata / Ciliophora / Intramacronucleata / Spirotrichea / Oligotrichia / Strombidiidae / Laboea
Eukaryota / Alveolata / Ciliophora / Intramacronucleata / Spirotrichea / Oligotrichia / Strombidiidae / Laboea
Eukaryota / Alveolata / Ciliophora / Intramacronucleata / Spirotrichea / Oligotrichia / Strombidiidae / Laboea
Eukaryota / Alveolata / Ciliophora / Intramacronucleata / Spirotrichea / Oligotrichia / Strombidiidae / Laboea
Eukaryota / Alveolata / Ciliophora / Intramacronucleata / Spirotrichea / Oligotrichia / Strombidiidae / Laboea
Eukaryota / Alveolata / Ciliophora / Intramacronucleata / Spirotrichea / Oligotrichia / Strombidiidae / Laboea
Eukaryota / Alveolata / Ciliophora / Intramacronucleata / Spirotrichea / Oligotrichia / Strombidiidae / Laboea
Eukaryota / Alveolata / Ciliophora / Intramacronucleata / Spirotrichea / Oligotrichia / Strombidiidae / Laboea
Eukaryota / Alveolata / Ciliophora / Intramacronucleata / Spirotrichea / Oligotrichia / Strombidiidae / Laboea
Eukaryota / Alveolata / Ciliophora / Intramacronucleata / Spirotrichea / Oligotrichia / Strombidiidae / Laboea
Eukaryota / Alveolata / Ciliophora / Intramacronucleata / Spirotrichea / Oligotrichia / Strombidiidae / Laboea
Eukaryota / Alveolata / Ciliophora / Intramacronucleata / Spirotrichea / Oligotrichia / Strombidiidae / Laboea
Eukaryota / Alveolata / Ciliophora / Intramacronucleata / Spirotrichea / Oligotrichia / Strombidiidae / Laboea
Eukaryota / Alveolata / Ciliophora / Intramacronucleata / Spirotrichea / Oligotrichia / Strombidiidae / Laboea
Eukaryota / Alveolata / Ciliophora / Intramacronucleata / Spirotrichea / Oligotrichia / Strombidiidae / Laboea
Eukaryota / Alveolata / Ciliophora / Intramacronucleata / Spirotrichea / Oligotrichia / Strombidiidae / Laboea
Eukaryota / Alveolata / Ciliophora / Intramacronucleata / Spirotrichea / Oligotrichia / Strombidiidae / Laboea
Eukaryota / Alveolata / Ciliophora / Intramacronucleata / Spirotrichea / Oligotrichia / Strombidiidae / Laboea
Eukaryota / Alveolata / Ciliophora / Intramacronucleata / Spirotrichea / Oligotrichia / Strombidiidae / Laboea
Eukaryota / Alveolata / Ciliophora / Intramacronucleata / Spirotrichea / Oligotrichia / Strombidiidae / Laboea
Eukaryota / Alveolata / Ciliophora / Intramacronucleata / Spirotrichea / Oligotrichia / Strombidiidae / Laboea
Eukaryota / Alveolata / Ciliophora / Intramacronucleata / Spirotrichea / Oligotrichia / Strombidiidae / Laboea
Eukaryota / Alveolata / Ciliophora / Intramacronucleata / Spirotrichea / Oligotrichia / Strombidiidae / Laboea
Eukaryota / Alveolata / Ciliophora / Intramacronucleata / Spirotrichea / Oligotrichia / Strombidiidae / Laboea
Eukaryota / Alveolata / Ciliophora / Intramacronucleata / Spirotrichea / Oligotrichia / Strombidiidae / Novistromb
Eukaryota / Alveolata / Ciliophora / Intramacronucleata / Spirotrichea / Oligotrichia / Strombidiidae / Novistromb
Eukaryota / Alveolata / Ciliophora / Intramacronucleata / Spirotrichea / Oligotrichia / Strombidiidae / Novistromb
Eukaryota / Alveolata / Ciliophora / Intramacronucleata / Spirotrichea / Oligotrichia / Strombidiidae / Novistromb

Eukaryota / Alveolata / Ciliophora / Intramacronucleata / Spirotrichea / Oligotrichia / Strombidiidae / Novistromb
Eukaryota / Alveolata / Ciliophora / Intramacronucleata / Spirotrichea / Oligotrichia / Strombidiidae / Novistromb
Eukaryota / Alveolata / Ciliophora / Intramacronucleata / Spirotrichea / Oligotrichia / Strombidiidae / Novistromb
Eukaryota / Alveolata / Ciliophora / Intramacronucleata / Spirotrichea / Oligotrichia / Strombidiidae / Omegastror
Eukaryota / Alveolata / Ciliophora / Intramacronucleata / Spirotrichea / Oligotrichia / Strombidiidae / Omegastror
Eukaryota / Alveolata / Ciliophora / Intramacronucleata / Spirotrichea / Oligotrichia / Strombidiidae / Omegastror
Eukaryota / Alveolata / Ciliophora / Intramacronucleata / Spirotrichea / Oligotrichia / Strombidiidae / Omegastror
Eukaryota / Alveolata / Ciliophora / Intramacronucleata / Spirotrichea / Oligotrichia / Strombidiidae / Parallelostro
Eukaryota / Alveolata / Ciliophora / Intramacronucleata / Spirotrichea / Oligotrichia / Strombidiidae / Strombidiur
Eukaryota / Alveolata / Ciliophora / Intramacronucleata / Spirotrichea / Oligotrichia / Strombidiidae / Strombidiur
Eukaryota / Alveolata / Ciliophora / Intramacronucleata / Spirotrichea / Oligotrichia / Strombidiidae / Strombidiur
Eukaryota / Alveolata / Ciliophora / Intramacronucleata / Spirotrichea / Oligotrichia / Strombidiidae / Strombidiur
Eukaryota / Alveolata / Ciliophora / Intramacronucleata / Spirotrichea / Oligotrichia / Strombidiidae / Strombidiur
Eukaryota / Alveolata / Ciliophora / Intramacronucleata / Spirotrichea / Oligotrichia / Strombidiidae / Strombidiur
Eukaryota / Alveolata / Ciliophora / Intramacronucleata / Spirotrichea / Oligotrichia / Strombidiidae / Strombidiur
Eukaryota / Alveolata / Ciliophora / Intramacronucleata / Spirotrichea / Oligotrichia / Strombidiidae / Strombidiur
Eukaryota / Alveolata / Ciliophora / Intramacronucleata / Spirotrichea / Oligotrichia / Strombidiidae / Strombidiur
Eukaryota / Alveolata / Ciliophora / Intramacronucleata / Spirotrichea / Oligotrichia / Strombidiidae / Strombidiur
Eukaryota / Alveolata / Ciliophora / Intramacronucleata / Spirotrichea / Oligotrichia / Strombidiidae / Strombidiur
Eukaryota / Alveolata / Ciliophora / Intramacronucleata / Spirotrichea / Oligotrichia / Strombidiidae / Strombidiur
Eukaryota / Alveolata / Ciliophora / Intramacronucleata / Spirotrichea / Oligotrichia / Strombidiidae / Strombidiur
Eukaryota / Alveolata / Ciliophora / Intramacronucleata / Spirotrichea / Oligotrichia / Strombidiidae / Strombidiur
Eukaryota / Alveolata / Ciliophora / Intramacronucleata / Spirotrichea / Oligotrichia / Strombidiidae / Strombidiur
Eukaryota / Alveolata / Ciliophora / Intramacronucleata / Spirotrichea / Oligotrichia / Strombidiidae / Strombidiur
Eukaryota / Alveolata / Ciliophora / Intramacronucleata / Spirotrichea / Oligotrichia / Strombidiidae / Strombidiur
Eukaryota / Alveolata / Ciliophora / Intramacronucleata / Spirotrichea / Oligotrichia / Strombidiidae / Strombidiur
Eukaryota / Alveolata / Ciliophora / Intramacronucleata / Spirotrichea / Oligotrichia / Strombidiidae / Strombidiur
Eukaryota / Alveolata / Ciliophora / Intramacronucleata / Spirotrichea / Oligotrichia / Strombidiidae / Strombidiur
Eukaryota / Alveolata / Ciliophora / Intramacronucleata / Spirotrichea / Oligotrichia / Strombidiidae / Strombidiur
Eukaryota / Alveolata / Ciliophora / Intramacronucleata / Spirotrichea / Oligotrichia / Strombidiidae / Strombidiur
Eukaryota / Alveolata / Ciliophora / Intramacronucleata / Spirotrichea / Oligotrichia / Strombidiidae / Strombidiur
Eukaryota / Alveolata / Ciliophora / Intramacronucleata / Spirotrichea / Oligotrichia / Strombidiidae / Strombidiur
Eukaryota / Alveolata / Ciliophora / Intramacronucleata / Spirotrichea / Oligotrichia / Strombidiidae / Strombidiur
Eukaryota / Alveolata / Ciliophora / Intramacronucleata / Spirotrichea / Oligotrichia / Strombidiidae / Strombidiur
Eukaryota / Alveolata / Ciliophora / Intramacronucleata / Spirotrichea / Oligotrichia / Strombidiidae / Strombidiur
Eukaryota / Alveolata / Ciliophora / Intramacronucleata / Spirotrichea / Oligotrichia / Strombidiidae / Strombidiur
Eukaryota / Alveolata / Ciliophora / Intramacronucleata / Spirotrichea / Oligotrichia / Strombidiidae / Strombidiur
Eukaryota / Alveolata / Ciliophora / Intramacronucleata / Spirotrichea / Oligotrichia / Strombidiidae / Strombidiur
Eukaryota / Alveolata / Ciliophora / Intramacronucleata / Spirotrichea / Oligotrichia / Strombidiidae / Strombidiur
Eukaryota / Alveolata / Ciliophora / Intramacronucleata / Spirotrichea / Oligotrichia / Strombidiidae / Strombidiur
Eukaryota / Alveolata / Ciliophora / Intramacronucleata / Spirotrichea / Oligotrichia / Strombidiidae / Strombidiur
Eukaryota / Alveolata / Ciliophora / Intramacronucleata / Spirotrichea / Oligotrichia / Strombidiidae / Strombidiur
Eukaryota / Alveolata / Ciliophora / Intramacronucleata / Spirotrichea / Oligotrichia / Strombidiidae / Strombidiur
Eukaryota / Alveolata / Ciliophora / Intramacronucleata / Spirotrichea / Oligotrichia / Strombidiidae / Strombidiur
Eukaryota / Alveolata / Ciliophora / Intramacronucleata / Spirotrichea / Oligotrichia / Strombidiidae / Strombidiur
Eukaryota / Alveolata / Ciliophora / Intramacronucleata / Spirotrichea / Oligotrichia / Strombidiidae / Strombidiur
Eukaryota / Alveolata / Ciliophora / Intramacronucleata / Spirotrichea / Oligotrichia / Strombidiidae / Strombidiur
Eukaryota / Alveolata / Ciliophora / Intramacronucleata / Spirotrichea / Oligotrichia / Strombidiidae / Strombidiur
Eukaryota / Alveolata / Ciliophora / Intramacronucleata / Spirotrichea / Oligotrichia / Strombidiidae / Strombidiur

Eukaryota / Alveolata / Ciliophora / Intramacronucleata / Spirotrichea / Oligotrichia / Strombidiidae / Strombidiur
Eukaryota / Alveolata / Ciliophora / Intramacronucleata / Spirotrichea / Oligotrichia / Strombidiidae / Strombidiur
Eukaryota / Alveolata / Ciliophora / Intramacronucleata / Spirotrichea / Oligotrichia / Strombidiidae / Strombidiur
Eukaryota / Alveolata / Ciliophora / Intramacronucleata / Spirotrichea / Oligotrichia / Strombidiidae / Strombidiur
Eukaryota / Alveolata / Ciliophora / Intramacronucleata / Spirotrichea / Oligotrichia / Strombidiidae / Strombidiur
Eukaryota / Alveolata / Ciliophora / Intramacronucleata / Spirotrichea / Oligotrichia / Strombidiidae / Strombidiur
Eukaryota / Alveolata / Ciliophora / Intramacronucleata / Spirotrichea / Oligotrichia / Strombidiidae / Strombidiur
Eukaryota / Alveolata / Ciliophora / Intramacronucleata / Spirotrichea / Oligotrichia / Strombidiidae / Strombidiur
Eukaryota / Alveolata / Ciliophora / Intramacronucleata / Spirotrichea / Oligotrichia / Strombidiidae / Strombidiur
Eukaryota / Alveolata / Ciliophora / Intramacronucleata / Spirotrichea / Oligotrichia / Strombidiidae / Strombidiur
Eukaryota / Alveolata / Ciliophora / Intramacronucleata / Spirotrichea / Oligotrichia / Strombidiidae / Strombidiur
Eukaryota / Alveolata / Ciliophora / Intramacronucleata / Spirotrichea / Oligotrichia / Strombidiidae / Strombidiur
Eukaryota / Alveolata / Ciliophora / Intramacronucleata / Spirotrichea / Oligotrichia / Strombidiidae / Strombidiur
Eukaryota / Alveolata / Ciliophora / Intramacronucleata / Spirotrichea / Oligotrichia / Strombidiidae / Strombidiur
Eukaryota / Alveolata / Ciliophora / Intramacronucleata / Spirotrichea / Oligotrichia / Strombidiidae / Strombidiur
Eukaryota / Alveolata / Ciliophora / Intramacronucleata / Spirotrichea / Oligotrichia / Strombidiidae / Strombidiur
Eukaryota / Alveolata / Ciliophora / Intramacronucleata / Spirotrichea / Oligotrichia / Strombidiidae / Strombidiur
Eukaryota / Alveolata / Ciliophora / Intramacronucleata / Spirotrichea / Oligotrichia / Strombidiidae / Strombidiur
Eukaryota / Alveolata / Ciliophora / Intramacronucleata / Spirotrichea / Oligotrichia / Strombidiidae / Strombidiur
Eukaryota / Alveolata / Ciliophora / Intramacronucleata / Spirotrichea / Oligotrichia / Strombidiidae / Strombidiur
Eukaryota / Alveolata / Ciliophora / Intramacronucleata / Spirotrichea / Oligotrichia / Strombidiidae / Strombidiur
Eukaryota / Alveolata / Ciliophora / Intramacronucleata / Spirotrichea / Oligotrichia / Strombidiidae / Strombidiur
Eukaryota / Alveolata / Ciliophora / Intramacronucleata / Spirotrichea / Oligotrichia / Strombidiidae / Strombidiur
Eukaryota / Alveolata / Ciliophora / Intramacronucleata / Spirotrichea / Oligotrichia / Strombidiidae / Strombidiur
Eukaryota / Alveolata / Ciliophora / Intramacronucleata / Spirotrichea / Oligotrichia / Strombidiidae / Strombidiur
Eukaryota / Alveolata / Ciliophora / Intramacronucleata / Spirotrichea / Oligotrichia / Strombidiidae / Strombidiur
Eukaryota / Alveolata / Ciliophora / Intramacronucleata / Spirotrichea / Oligotrichia / Strombidiidae / Strombidiur
Eukaryota / Alveolata / Ciliophora / Intramacronucleata / Spirotrichea / Oligotrichia / Strombidiidae / Strombidiur
Eukaryota / Alveolata / Ciliophora / Intramacronucleata / Spirotrichea / Oligotrichia / Strombidiidae / Strombidiur
Eukaryota / Alveolata / Ciliophora / Intramacronucleata / Spirotrichea / Oligotrichia / Strombidiidae / Strombidiur
Eukaryota / Alveolata / Ciliophora / Intramacronucleata / Spirotrichea / Oligotrichia / Strombidiidae / Strombidiur
Eukaryota / Alveolata / Ciliophora / Intramacronucleata / Spirotrichea / Oligotrichia / Strombidiidae / Strombidiur
Eukaryota / Alveolata / Ciliophora / Intramacronucleata / Spirotrichea / Oligotrichia / Strombidiidae / Strombidiur
Eukaryota / Alveolata / Ciliophora / Intramacronucleata / Spirotrichea / Oligotrichia / Strombidiidae / Strombidiur
Eukaryota / Alveolata / Ciliophora / Intramacronucleata / Spirotrichea / Oligotrichia / Strombidiidae / Strombidiur
Eukaryota / Alveolata / Ciliophora / Intramacronucleata / Spirotrichea / Oligotrichia / Strombidiidae / Strombidiur
Eukaryota / Alveolata / Ciliophora / Intramacronucleata / Spirotrichea / Oligotrichia / Strombidiidae / Strombidiur
Eukaryota / Alveolata / Ciliophora / Intramacronucleata / Spirotrichea / Oligotrichia / Strombidiidae / Strombidiur
Eukaryota / Alveolata / Ciliophora / Intramacronucleata / Spirotrichea / Oligotrichia / Strombidiidae / Strombidiur
Eukaryota / Alveolata / Ciliophora / Intramacronucleata / Spirotrichea / Oligotrichia / Strombidiidae / Strombidiur
Eukaryota / Alveolata / Ciliophora / Intramacronucleata / Spirotrichea / Oligotrichia / Strombidiidae / Strombidiur
Eukaryota / Alveolata / Ciliophora / Intramacronucleata / Spirotrichea / Oligotrichia / Strombidiidae / Strombidiur
Eukaryota / Alveolata / Ciliophora / Intramacronucleata / Spirotrichea / Oligotrichia / Strombidiidae / Strombidiur
Eukaryota / Alveolata / Ciliophora / Intramacronucleata / Spirotrichea / Oligotrichia / Strombidiidae / Strombidiur
Eukaryota / Alveolata / Ciliophora / Intramacronucleata / Spirotrichea / Oligotrichia / Strombidiidae / Strombidiur
Eukaryota / Alveolata / Ciliophora / Intramacronucleata / Spirotrichea / Oligotrichia / Strombidiidae / Strombidiur
Eukaryota / Alveolata / Ciliophora / Intramacronucleata / Spirotrichea / Oligotrichia / Strombidiidae / Strombidiur
Eukaryota / Alveolata / Ciliophora / Intramacronucleata / Spirotrichea / Oligotrichia / Strombidiidae / Strombidiur

Eukaryota / Alveolata / Ciliophora / Intramacronucleata / Spirotrichea / Oligotrichia / Strombidiidae / Varistrombi
Eukaryota / Alveolata / Ciliophora / Intramacronucleata / Spirotrichea / Oligotrichia / Tontoniidae / Pseudotonton
Eukaryota / Alveolata / Ciliophora / Intramacronucleata / Spirotrichea / Oligotrichia / Tontoniidae / Pseudotonton
Eukaryota / Alveolata / Ciliophora / Intramacronucleata / Spirotrichea / Oligotrichia / Tontoniidae / Pseudotonton
Eukaryota / Alveolata / Ciliophora / Intramacronucleata / Spirotrichea / Oligotrichia / Tontoniidae / Pseudotonton
Eukaryota / Alveolata / Ciliophora / Intramacronucleata / Spirotrichea / Oligotrichia / Tontoniidae / Pseudotonton
Eukaryota / Alveolata / Ciliophora / Intramacronucleata / Spirotrichea / Oligotrichia / Tontoniidae / Pseudotonton
Eukaryota / Alveolata / Ciliophora / Intramacronucleata / Spirotrichea / Oligotrichia / Tontoniidae / Pseudotonton
Eukaryota / Alveolata / Ciliophora / Intramacronucleata / Spirotrichea / Oligotrichia / Tontoniidae / Pseudotonton
Eukaryota / Alveolata / Ciliophora / Intramacronucleata / Spirotrichea / Oligotrichia / Tontoniidae / Pseudotonton
Eukaryota / Alveolata / Ciliophora / Intramacronucleata / Spirotrichea / Oligotrichia / Tontoniidae / Pseudotonton
Eukaryota / Alveolata / Ciliophora / Intramacronucleata / Spirotrichea / Oligotrichia / Tontoniidae / Pseudotonton
Eukaryota / Alveolata / Ciliophora / Intramacronucleata / Spirotrichea / Oligotrichia / Tontoniidae / Pseudotonton
Eukaryota / Alveolata / Ciliophora / Intramacronucleata / Spirotrichea / Oligotrichia / Tontoniidae / Pseudotonton
Eukaryota / Alveolata / Ciliophora / Intramacronucleata / Spirotrichea / Oligotrichia / Tontoniidae / Pseudotonton
Eukaryota / Alveolata / Ciliophora / Intramacronucleata / Spirotrichea / Oligotrichia / Tontoniidae / Pseudotonton
Eukaryota / Alveolata / Ciliophora / Intramacronucleata / Spirotrichea / Oligotrichia / Tontoniidae / Pseudotonton
Eukaryota / Alveolata / Ciliophora / Intramacronucleata / Spirotrichea / Oligotrichia / Tontoniidae / Pseudotonton
Eukaryota / Alveolata / Ciliophora / Intramacronucleata / Spirotrichea / Oligotrichia / Tontoniidae / Pseudotonton
Eukaryota / Alveolata / Ciliophora / Intramacronucleata / Spirotrichea / Oligotrichia / Tontoniidae / Pseudotonton
Eukaryota / Alveolata / Ciliophora / Intramacronucleata / Spirotrichea / Oligotrichia / Tontoniidae / Pseudotonton
Eukaryota / Alveolata / Ciliophora / Intramacronucleata / Spirotrichea / Oligotrichia / Tontoniidae / Pseudotonton
Eukaryota / Alveolata / Ciliophora / Intramacronucleata / Spirotrichea / Oligotrichia / Tontoniidae / Pseudotonton
Eukaryota / Alveolata / Ciliophora / Intramacronucleata / Spirotrichea / Oligotrichia / Tontoniidae / Pseudotonton
Eukaryota / Alveolata / Ciliophora / Intramacronucleata / Spirotrichea / Oligotrichia / Tontoniidae / Pseudotonton
Eukaryota / Alveolata / Ciliophora / Intramacronucleata / Spirotrichea / Oligotrichia / Tontoniidae / Pseudotonton
Eukaryota / Alveolata / Ciliophora / Intramacronucleata / Spirotrichea / Oligotrichia / Tontoniidae / Pseudotonton
Eukaryota / Alveolata / Ciliophora / Intramacronucleata / Spirotrichea / Oligotrichia / Tontoniidae / Pseudotonton
Eukaryota / Alveolata / Ciliophora / Intramacronucleata / Spirotrichea / Oligotrichia / Tontoniidae / Pseudotonton
Eukaryota / Alveolata / Ciliophora / Intramacronucleata / Spirotrichea / Oligotrichia / Tontoniidae / Pseudotonton
Eukaryota / Alveolata / Ciliophora / Intramacronucleata / Spirotrichea / Oligotrichia / Tontoniidae / Pseudotonton
Eukaryota / Alveolata / Ciliophora / Intramacronucleata / Spirotrichea / Oligotrichia / Tontoniidae / Pseudotonton
Eukaryota / Alveolata / Ciliophora / Intramacronucleata / Spirotrichea / Oligotrichia / Tontoniidae / Pseudotonton
Eukaryota / Alveolata / Ciliophora / Intramacronucleata / Spirotrichea / Oligotrichia / Tontoniidae / Pseudotonton
Eukaryota / Alveolata / Ciliophora / Intramacronucleata / Spirotrichea / Oligotrichia / Tontoniidae / Pseudotonton
Eukaryota / Alveolata / Ciliophora / Intramacronucleata / Spirotrichea / Oligotrichia / Tontoniidae / Pseudotonton
Eukaryota / Alveolata / Ciliophora / Intramacronucleata / Spirotrichea / Oligotrichia / Tontoniidae / Spirotontonia
Eukaryota / Alveolata / Ciliophora / Intramacronucleata / Spirotrichea / Oligotrichia / Tontoniidae / Spirotontonia
Eukaryota / Alveolata / Ciliophora / Intramacronucleata / Spirotrichea / Oligotrichia / Tontoniidae / Spirotontonia
Eukaryota / Alveolata / Ciliophora / Intramacronucleata / Spirotrichea / Oligotrichia / Tontoniidae / Spirotontonia
Eukaryota / Alveolata / Ciliophora / Intramacronucleata / Spirotrichea / Oligotrichia / Tontoniidae / Spirotontonia
Eukaryota / Alveolata / Ciliophora / Intramacronucleata / Spirotrichea / Oligotrichia / Tontoniidae / Spirotontonia
Eukaryota / Alveolata / Ciliophora / Intramacronucleata / Spirotrichea / Oligotrichia / Tontoniidae / Spirotontonia
Eukaryota / Alveolata / Ciliophora / Intramacronucleata / Spirotrichea / Oligotrichia / Tontoniidae / Spirotontonia
Eukaryota / Alveolata / Ciliophora / Intramacronucleata / Spirotrichea / Oligotrichia / Tontoniidae / Spirotontonia
Eukaryota / Alveolata / Ciliophora / Intramacronucleata / Spirotrichea / Oligotrichia / Tontoniidae / Spirotontonia
Eukaryota / Alveolata / Ciliophora / Intramacronucleata / Spirotrichea / Oligotrichia / Tontoniidae / Spirotontonia
Eukaryota / Alveolata / Ciliophora / Intramacronucleata / Spirotrichea / Oligotrichia / Tontoniidae / Spirotontonia

Eukaryota / Alveolata / Ciliophora / Intramacronucleata / Spirotrichea / Oligotrichia / Tontoniidae / Spirotontonia
Eukaryota / Alveolata / Ciliophora / Intramacronucleata / Spirotrichea / Oligotrichia / Tontoniidae / Spirotontonia
Eukaryota / Alveolata / Ciliophora / Intramacronucleata / Spirotrichea / Oligotrichia / Tontoniidae / Spirotontonia
Eukaryota / Alveolata / Ciliophora / Intramacronucleata / Spirotrichea / Oligotrichia / Tontoniidae / Spirotontonia
Eukaryota / Alveolata / Ciliophora / Intramacronucleata / Spirotrichea / Oligotrichia / Tontoniidae / Spirotontonia
Eukaryota / Alveolata / Ciliophora / Intramacronucleata / Spirotrichea / Oligotrichia / Tontoniidae / Spirotontonia
Eukaryota / Alveolata / Ciliophora / Intramacronucleata / Spirotrichea / Oligotrichia / Tontoniidae / Spirotontonia
Eukaryota / Alveolata / Ciliophora / Intramacronucleata / Spirotrichea / Oligotrichia / Tontoniidae / Spirotontonia
Eukaryota / Alveolata / Ciliophora / Intramacronucleata / Spirotrichea / Oligotrichia / Tontoniidae / Spirotontonia
Eukaryota / Alveolata / Ciliophora / Intramacronucleata / Spirotrichea / Oligotrichia / Tontoniidae / Spirotontonia
Eukaryota / Alveolata / Ciliophora / Intramacronucleata / Spirotrichea / Oligotrichia / Tontoniidae / Spirotontonia
Eukaryota / Alveolata / Ciliophora / Intramacronucleata / Spirotrichea / Oligotrichia / Tontoniidae / Spirotontonia
Eukaryota / Alveolata / Ciliophora / Intramacronucleata / Spirotrichea / Oligotrichia / Tontoniidae / Spirotontonia
Eukaryota / Alveolata / Ciliophora / Intramacronucleata / Spirotrichea / Oligotrichia / Tontoniidae / Spirotontonia
Eukaryota / Alveolata / Ciliophora / Intramacronucleata / Spirotrichea / Oligotrichia / Tontoniidae / Spirotontonia
Eukaryota / Alveolata / Ciliophora / Intramacronucleata / Spirotrichea / Oligotrichia / Tontoniidae / Spirotontonia
Eukaryota / Alveolata / Ciliophora / Intramacronucleata / Spirotrichea / Oligotrichia / Tontoniidae / Spirotontonia
Eukaryota / Alveolata / Ciliophora / Intramacronucleata / Spirotrichea / Protocruziidia / Protocruzia
Eukaryota / Alveolata / Ciliophora / Intramacronucleata / Spirotrichea / Protocruziidia / Protocruzia
Eukaryota / Alveolata / Ciliophora / Intramacronucleata / Spirotrichea / Protocruziidia / Protocruzia
Eukaryota / Alveolata / Ciliophora / Intramacronucleata / Spirotrichea / Stichotrichia / Plagiotomida / Plagiotoma
Eukaryota / Alveolata / Ciliophora / Intramacronucleata / Spirotrichea / Stichotrichia / Pseudoamphisiellida / Pseu
Eukaryota / Alveolata / Ciliophora / Intramacronucleata / Spirotrichea / Stichotrichia / Pseudoamphisiellida / Pseu
Eukaryota / Alveolata / Ciliophora / Intramacronucleata / Spirotrichea / Stichotrichia / Pseudoamphisiellida / Pseu
Eukaryota / Alveolata / Ciliophora / Intramacronucleata / Spirotrichea / Stichotrichia / Pseudoamphisiellida / Pseu
Eukaryota / Alveolata / Ciliophora / Intramacronucleata / Spirotrichea / Stichotrichia / Sporadotrichida / Halteriida
Eukaryota / Alveolata / Ciliophora / Intramacronucleata / Spirotrichea / Stichotrichia / Sporadotrichida / Oxytrichi
Eukaryota / Alveolata / Ciliophora / Intramacronucleata / Spirotrichea / Stichotrichia / Sporadotrichida / Oxytrichi
Eukaryota / Alveolata / Ciliophora / Intramacronucleata / Spirotrichea / Stichotrichia / Sporadotrichida / Oxytrichi
Eukaryota / Alveolata / Ciliophora / Intramacronucleata / Spirotrichea / Stichotrichia / Sporadotrichida / Oxytrichi
Eukaryota / Alveolata / Ciliophora / Intramacronucleata / Spirotrichea / Stichotrichia / Sporadotrichida / Oxytrichi
Eukaryota / Alveolata / Ciliophora / Intramacronucleata / Spirotrichea / Stichotrichia / Sporadotrichida / Oxytrichi
Eukaryota / Alveolata / Ciliophora / Intramacronucleata / Spirotrichea / Stichotrichia / Sporadotrichida / Oxytrichi
Eukaryota / Alveolata / Ciliophora / Intramacronucleata / Spirotrichea / Stichotrichia / Sporadotrichida / Oxytrichi
Eukaryota / Alveolata / Ciliophora / Intramacronucleata / Spirotrichea / Stichotrichia / Sporadotrichida / Oxytrichi
Eukaryota / Alveolata / Ciliophora / Intramacronucleata / Spirotrichea / Stichotrichia / Sporadotrichida / Oxytrichi
Eukaryota / Alveolata / Ciliophora / Intramacronucleata / Spirotrichea / Stichotrichia / Sporadotrichida / Oxytrichi
Eukaryota / Alveolata / Ciliophora / Intramacronucleata / Spirotrichea / Stichotrichia / Sporadotrichida / Oxytrichi
Eukaryota / Alveolata / Ciliophora / Intramacronucleata / Spirotrichea / Stichotrichia / Sporadotrichida / Oxytrichi
Eukaryota / Alveolata / Ciliophora / Intramacronucleata / Spirotrichea / Stichotrichia / Sporadotrichida / Oxytrichi
Eukaryota / Alveolata / Ciliophora / Intramacronucleata / Spirotrichea / Stichotrichia / Sporadotrichida / Oxytrichi
Eukaryota / Alveolata / Ciliophora / Intramacronucleata / Spirotrichea / Stichotrichia / Sporadotrichida / Oxytrichi
Eukaryota / Alveolata / Ciliophora / Intramacronucleata / Spirotrichea / Stichotrichia / Sporadotrichida / Oxytrichi
Eukaryota / Alveolata / Ciliophora / Intramacronucleata / Spirotrichea / Stichotrichia / Sporadotrichida / Oxytrichi
Eukaryota / Alveolata / Ciliophora / Intramacronucleata / Spirotrichea / Stichotrichia / Sporadotrichida / Oxytrichi
Eukaryota / Alveolata / Ciliophora / Intramacronucleata / Spirotrichea / Stichotrichia / Sporadotrichida / Oxytrichi
Eukaryota / Alveolata / Ciliophora / Intramacronucleata / Spirotrichea / Stichotrichia / Sporadotrichida / Oxytrichi
Eukaryota / Alveolata / Ciliophora / Intramacronucleata / Spirotrichea / Stichotrichia / Stichotrichida / Amphisielli

Eukaryota / Alveolata / Ciliophora / Intramacronucleata / Spirotrichea / Stichotrichia / Stichotrichida / Amphisielli
Eukaryota / Alveolata / Ciliophora / Intramacronucleata / Spirotrichea / Stichotrichia / Stichotrichida / Amphisielli
Eukaryota / Alveolata / Ciliophora / Intramacronucleata / Spirotrichea / Stichotrichia / Stichotrichida / Amphisielli
Eukaryota / Alveolata / Ciliophora / Intramacronucleata / Spirotrichea / Stichotrichia / Stichotrichida / Amphisielli
Eukaryota / Alveolata / Ciliophora / Intramacronucleata / Spirotrichea / Stichotrichia / Stichotrichida / Amphisielli
Eukaryota / Alveolata / Ciliophora / Intramacronucleata / Spirotrichea / Stichotrichia / Stichotrichida / Amphisielli
Eukaryota / Alveolata / Ciliophora / Intramacronucleata / Spirotrichea / Stichotrichia / Stichotrichida / Amphisielli
Eukaryota / Alveolata / Ciliophora / Intramacronucleata / Spirotrichea / Stichotrichia / Stichotrichida / Apogastros
Eukaryota / Alveolata / Ciliophora / Intramacronucleata / Spirotrichea / Stichotrichia / Stichotrichida / Epiclintidae
Eukaryota / Alveolata / Ciliophora / Intramacronucleata / Spirotrichea / Stichotrichia / Stichotrichida / Schmidinge
Eukaryota / Alveolata / Ciliophora / Intramacronucleata / Spirotrichea / Stichotrichia / unclassified Stichotrichia /
Eukaryota / Alveolata / Ciliophora / Intramacronucleata / Spirotrichea / Stichotrichia / unclassified Stichotrichia /
Eukaryota / Alveolata / Ciliophora / Intramacronucleata / Spirotrichea / Stichotrichia / unclassified Stichotrichia /
Eukaryota / Alveolata / Ciliophora / Intramacronucleata / Spirotrichea / Stichotrichia / unclassified Stichotrichia /
Eukaryota / Alveolata / Ciliophora / Intramacronucleata / Spirotrichea / Stichotrichia / unclassified Stichotrichia /
Eukaryota / Alveolata / Ciliophora / Intramacronucleata / Spirotrichea / Stichotrichia / unclassified Stichotrichia /
Eukaryota / Alveolata / Ciliophora / Intramacronucleata / Spirotrichea / Stichotrichia / unclassified Stichotrichia /
Eukaryota / Alveolata / Ciliophora / Intramacronucleata / Spirotrichea / Stichotrichia / unclassified Stichotrichia /
Eukaryota / Alveolata / Ciliophora / Intramacronucleata / Spirotrichea / Stichotrichia / unclassified Stichotrichia /
Eukaryota / Alveolata / Ciliophora / Intramacronucleata / Spirotrichea / Stichotrichia / unclassified Stichotrichia /
Eukaryota / Alveolata / Ciliophora / Intramacronucleata / Spirotrichea / Stichotrichia / unclassified Stichotrichia /
Eukaryota / Alveolata / Ciliophora / Intramacronucleata / Spirotrichea / Stichotrichia / unclassified Stichotrichia /
Eukaryota / Alveolata / Ciliophora / Intramacronucleata / Spirotrichea / Stichotrichia / unclassified Stichotrichia /
Eukaryota / Alveolata / Ciliophora / Intramacronucleata / Spirotrichea / Stichotrichia / unclassified Stichotrichia /
Eukaryota / Alveolata / Ciliophora / Intramacronucleata / Spirotrichea / Stichotrichia / unclassified Stichotrichia /
Eukaryota / Alveolata / Ciliophora / Intramacronucleata / Spirotrichea / Stichotrichia / unclassified Stichotrichia /
Eukaryota / Alveolata / Ciliophora / Intramacronucleata / Spirotrichea / Stichotrichia / unclassified Stichotrichia /
Eukaryota / Alveolata / Ciliophora / Intramacronucleata / Spirotrichea / Stichotrichia / unclassified Stichotrichia /
Eukaryota / Alveolata / Ciliophora / Intramacronucleata / Spirotrichea / Stichotrichia / Urostylida / Holostichidae /
Eukaryota / Alveolata / Ciliophora / Intramacronucleata / Spirotrichea / Stichotrichia / Urostylida / Holostichidae /
Eukaryota / Alveolata / Ciliophora / Intramacronucleata / Spirotrichea / Stichotrichia / Urostylida / Holostichidae /
Eukaryota / Alveolata / Ciliophora / Intramacronucleata / Spirotrichea / Stichotrichia / Urostylida / Holostichidae /
Eukaryota / Alveolata / Ciliophora / Intramacronucleata / Spirotrichea / Stichotrichia / Urostylida / Holostichidae /
Eukaryota / Alveolata / Ciliophora / Intramacronucleata / Spirotrichea / Stichotrichia / Urostylida / Holostichidae /
Eukaryota / Alveolata / Ciliophora / Intramacronucleata / Spirotrichea / Stichotrichia / Urostylida / Holostichidae /
Eukaryota / Alveolata / Ciliophora / Intramacronucleata / Spirotrichea / Stichotrichia / Urostylida / Pseudokerono
Eukaryota / Alveolata / Ciliophora / Intramacronucleata / Spirotrichea / Stichotrichia / Urostylida / Pseudokerono
Eukaryota / Alveolata / Ciliophora / Intramacronucleata / Spirotrichea / Stichotrichia / Urostylida / Pseudokerono
Eukaryota / Alveolata / Ciliophora / Intramacronucleata / Spirotrichea / Stichotrichia / Urostylida / Urostylidae / U
Eukaryota / Alveolata / Ciliophora / Intramacronucleata / Spirotrichea / Stichotrichia / Urostylida / Urostylidae / U
Eukaryota / Alveolata / Ciliophora / Intramacronucleata / Spirotrichea / Stichotrichia / Urostylida / Urostylidae / U
Eukaryota / Alveolata / Ciliophora / Intramacronucleata / Spirotrichea / Stichotrichia / Urostylida / Urostylidae / U
Eukaryota / Alveolata / Ciliophora / Intramacronucleata / Spirotrichea / Stichotrichia / Urostylida / Urostylidae / U
Eukaryota / Alveolata / Ciliophora / Intramacronucleata / Spirotrichea / Stichotrichia / Urostylida / Urostylidae / U
Eukaryota / Alveolata / Ciliophora / Intramacronucleata / Spirotrichea / Stichotrichia / Urostylida / Urostylidae / U
Eukaryota / Alveolata / Ciliophora / Intramacronucleata / Spirotrichea / Stichotrichia / Urostylida / Urostylidae / U
Eukaryota / Alveolata / Ciliophora / Intramacronucleata / Spirotrichea / Stichotrichia / Urostylida / Urostylidae / U
Eukaryota / Alveolata / Ciliophora / Intramacronucleata / Spirotrichea / Stichotrichia / Urostylida / Urostylidae / U

Eukaryota / Alveolata / Ciliophora / Intramacronucleata / Spirotrichea / unclassified Spirotrichea / Rigidotrichidae
Eukaryota / Alveolata / Ciliophora / Postciliodesmatophora / Heterotrichea / Heterotrichida / Chattonidiidae / Cha
Eukaryota / Alveolata / Ciliophora / Postciliodesmatophora / Heterotrichea / Heterotrichida / Chattonidiidae / Cha
Eukaryota / Alveolata / Ciliophora / Postciliodesmatophora / Heterotrichea / Heterotrichida / Climacostomidae / F
Eukaryota / Alveolata / Ciliophora / Postciliodesmatophora / Heterotrichea / Heterotrichida / Climacostomidae / F
Eukaryota / Alveolata / Ciliophora / Postciliodesmatophora / Heterotrichea / Heterotrichida / Folliculinidae / Follic
Eukaryota / Alveolata / Ciliophora / Postciliodesmatophora / Heterotrichea / Heterotrichida / Peritromidae / Perit
Eukaryota / Alveolata / Ciliophora / Postciliodesmatophora / Heterotrichea / Heterotrichida / Peritromidae / Perit
Eukaryota / Alveolata / Ciliophora / Postciliodesmatophora / Heterotrichea / Heterotrichida / Peritromidae / Perit
Eukaryota / Alveolata / Ciliophora / Postciliodesmatophora / Heterotrichea / Heterotrichida / Peritromidae / Perit
Eukaryota / Alveolata / Ciliophora / Postciliodesmatophora / Heterotrichea / Heterotrichida / Phacodinium
Eukaryota / Alveolata / Ciliophora / Postciliodesmatophora / Heterotrichea / Licnophorida / Licnophoridae / Licno
Eukaryota / Alveolata / Ciliophora / Postciliodesmatophora / Karyorelictea / Loxodida / Loxodidae / Loxodes
Eukaryota / Alveolata / Ciliophora / Postciliodesmatophora / Karyorelictea / Loxodida / Loxodidae / Loxodes
Eukaryota / Alveolata / Ciliophora / Postciliodesmatophora / Karyorelictea / Loxodida / Loxodidae / Remanella
Eukaryota / Alveolata / Ciliophora / Postciliodesmatophora / Karyorelictea / Loxodida / Loxodidae / Remanella
Eukaryota / Alveolata / Ciliophora / Postciliodesmatophora / Karyorelictea / Loxodida / Loxodidae / Remanella
Eukaryota / Alveolata / Ciliophora / Postciliodesmatophora / Karyorelictea / Loxodida / Loxodidae / Remanella
Eukaryota / Alveolata / Ciliophora / Postciliodesmatophora / Karyorelictea / Loxodida / Loxodidae / Remanella
Eukaryota / Alveolata / Ciliophora / Postciliodesmatophora / Karyorelictea / Loxodida / Loxodidae / Remanella
Eukaryota / Alveolata / Ciliophora / Postciliodesmatophora / Karyorelictea / Loxodida / Loxodidae / Remanella
Eukaryota / Alveolata / Ciliophora / Postciliodesmatophora / Karyorelictea / Loxodida / Loxodidae / Remanella
Eukaryota / Alveolata / Ciliophora / Postciliodesmatophora / Karyorelictea / Loxodida / Loxodidae / Remanella
Eukaryota / Alveolata / Ciliophora / Postciliodesmatophora / Karyorelictea / Loxodida / Loxodidae / Remanella
Eukaryota / Alveolata / Ciliophora / Postciliodesmatophora / Karyorelictea / Loxodida / Loxodidae / Remanella
Eukaryota / Alveolata / Ciliophora / Postciliodesmatophora / Karyorelictea / Loxodida / Loxodidae / Remanella
Eukaryota / Alveolata / Ciliophora / Postciliodesmatophora / Karyorelictea / Protostomatida / Trachelocercidae / [illegible]

Blast % similarity	Blast Hit Genbank
83.78%	KF607087
83.78%	KC139715
84.68%	KC139715
81.98%	KC139717
81.08%	KC139715
82.88%	KC139717
81.08%	KC139715
88.8%	AF060455
88.8%	AF060455
97.56%	KF569684
87.7%	KF569684
86.06%	KF569684
90.16%	KF569684
84.92%	JF747213
87.12%	U82204
85.6%	JF747212
88.7%	AF060453
90.16%	JF747216
100.0%	HM215507
88.52%	M97908
82.11%	DQ388599
90.24%	M97908
88.52%	M97908
89.34%	M97908
85.24%	M97908
95.93%	M97908
98.37%	DQ388599
90.98%	M97908
99.18%	JF747216
89.34%	JF747215
89.34%	HM140394
96.74%	HM140394
88.52%	JF747217
86.88%	JF747218
89.34%	JF747218
93.49%	JF747217
99.19%	GQ259748
81.14%	GQ259748
91.86%	GQ259748
83.6%	GQ259748
87.7%	EU264562
87.7%	GQ259748
86.88%	EU264562
85.24%	GQ259748
83.6%	GQ259748
86.06%	GQ259748
86.06%	EU264562

86.88%	GQ259748
84.42%	GQ259748
80.32%	GQ259748
85.24%	GQ259748
90.16%	GQ259748
83.6%	GQ259748
84.42%	GQ259748
84.42%	GQ259748
95.12%	EU264562
81.14%	GQ259748
85.24%	GQ259748
83.6%	EU264562
84.42%	GQ259748
86.06%	GQ259748
85.24%	EU264562
85.24%	GQ259748
84.42%	EU264562
84.42%	GQ259748
86.17%	EU264562
85.24%	EU264562
86.06%	EU264562
92.68%	GQ259748
98.37%	GQ259748
95.12%	GQ259748
83.6%	GQ259748
85.24%	GQ259748
86.06%	GQ259748
84.42%	GQ259748
97.56%	GQ259748
86.88%	JQ723962
88.52%	JQ723962
87.7%	JQ723962
90.16%	EU264564
95.93%	AF060454
81.3%	JQ356868
98.37%	AF060452
100.0%	AF060452
87.7%	AF300288
99.18%	AF300286
92.15%	JN412739
100.0%	JN412737
95.04%	JN084213
86.0%	JN084213
97.0%	JN084213
87.0%	JN412739
85.57%	JN412738
94.0%	JN412740
96.0%	JN412737

84.46%	JN412738
92.0%	JN412737
98.0%	JN412740
96.38%	JN412737
84.15%	JN412739
100.0%	HQ227994
95.09%	HQ227994
93.06%	HQ227994
94.0%	HQ227994
96.03%	HQ227994
100.0%	HQ227920
93.0%	HQ227994
98.0%	HQ227994
82.56%	JF263448
95.93%	FJ876971
88.61%	FJ876971
87.7%	FJ876971
98.37%	FJ876971
95.93%	FJ876971
99.06%	U57771
97.19%	U57771
80.0%	DQ487196
81.73%	JF263447
83.65%	JF263447
83.49%	JF263447
87.7%	FJ875140
87.8%	FJ875140
88.52%	FJ875140
89.34%	FJ875140
85.24%	FJ875140
80.18%	DQ411860
95.28%	DQ411860
84.61%	JF263444
84.61%	JF263444
83.8%	JF263444
84.61%	JF263444
98.11%	Z22931
96.0%	JF263451
94.0%	Z22931
81.13%	DQ411863
91.5%	HM581673
82.24%	U80313
83.48%	U80313
93.45%	AY102173
91.58%	GQ351698
97.19%	L26448
80.73%	EF123708
97.19%	KC493570

80.9%	AF298821
80.55%	AB794977
80.23%	AB555711
80.73%	AB555712
80.37%	AB555711
83.33%	AB555712
86.2%	AB555711
80.55%	AB555712
82.4%	AB795029
84.25%	AB555710
81.98%	U57769
80.55%	AB794982
86.06%	HQ668466
88.52%	HQ668466
83.47%	EU286811
83.47%	EU286811
85.95%	EU286811
84.29%	HQ668466
87.8%	HQ668466
83.47%	HQ668466
84.29%	HQ668466
82.78%	HQ668466
81.81%	HQ668466
83.47%	EU286811
83.47%	HQ668466
81.66%	HQ668466
81.3%	HQ668466
91.26%	EU264560
83.87%	KC832955
85.18%	KC832955
94.3%	KC832949
89.34%	X65149
92.68%	X65149
89.34%	X65149
87.8%	X65149
88.52%	X65149
97.56%	X65149
97.34%	DQ232761
93.22%	HQ591488
100.0%	HQ591488
90.83%	HQ591488
91.52%	HQ591473
94.87%	EU503535
93.22%	EU503534
88.42%	EU503534
88.79%	EU503534
93.22%	EU503534
92.37%	EU503534

88.88%	EU503534
87.28%	EU503535
90.51%	EU503534
88.33%	EU503534
92.37%	EU503534
93.16%	EU503534
95.72%	EU503535
94.01%	EU503535
94.06%	EU503535
91.52%	EU503534
92.37%	EU503535
92.3%	EU503535
90.67%	EU503535
92.24%	EU503534
91.52%	EU503535
87.06%	EU503534
91.52%	EU503534
92.37%	EU503534
93.1%	EU503534
87.06%	EU503538
93.16%	EU503538
86.55%	EU503536
85.34%	EU503538
99.14%	EU503539
88.88%	EU503539
91.45%	EU503539
89.16%	EU503539
98.29%	EU503539
89.91%	EU503539
96.58%	EU503539
84.61%	EU503539
96.58%	EU503539
87.17%	EU503539
96.58%	EU503539
98.29%	EU503539
98.3%	KF516511
91.52%	KF516511
90.67%	KF516511
91.52%	KF516511
98.3%	KF516511
90.67%	KF516511
89.83%	KF516511
90.67%	KF516511
92.37%	KF516511
90.67%	KF516511
88.42%	KF516511
88.98%	KF516511
93.22%	KF516511

91.52%	KF516511
87.6%	KF516511
92.37%	KF516511
94.06%	KF516511
90.67%	KF516511
95.76%	KF516511
91.52%	KF516511
95.76%	KF516511
97.45%	KF516511
91.52%	KF516511
96.61%	KF516511
92.37%	KF516511
88.98%	KF516511
88.98%	KF516511
88.33%	KF516511
89.83%	KF516511
99.15%	KF516511
81.96%	HQ446276
81.81%	HQ446276
85.0%	HQ446277
85.0%	HQ446277
100.0%	X56171
86.17%	HE819879
90.0%	HM030738
90.9%	HM030738
88.33%	HM030738
90.0%	HM030738
86.66%	HM030738
88.33%	HM030738
88.33%	HM030738
87.6%	HM030738
87.7%	HM030738
88.33%	AF255357
88.33%	AF255357
86.77%	AF255357
86.88%	AF255357
100.0%	EF417835
80.9%	EF599288
81.65%	AY788099
80.9%	EF569682
80.0%	EF599288
82.88%	EF599288
80.9%	EF569682
82.56%	JQ663868
82.72%	EF599288
99.08%	AY788099
81.65%	JQ663869
82.4%	JQ663869

84.4%	JQ663869
80.73%	JQ663869
94.33%	GU187058
94.49%	DQ662847
100.0%	KF524423
100.0%	KF524426
100.0%	DQ868351
89.81%	DQ868350
98.14%	DQ662855
97.22%	AF429897
94.44%	DQ868352
88.42%	FJ870103
85.95%	FJ870103
87.6%	FJ870103
87.6%	FJ870103
86.77%	FJ870103
89.25%	FJ870103
90.83%	FJ870103
85.95%	FJ870103
89.25%	FJ870103
89.25%	FJ870103
90.9%	FJ870103
85.83%	FJ870103
89.25%	FJ870103
85.0%	FJ870103
84.16%	FJ870103
86.66%	FJ870103
88.33%	FJ870103
89.25%	FJ870103
85.12%	FJ870103
86.06%	FJ870103
85.83%	FJ870103
95.04%	FJ870103
90.16%	FJ870103
89.25%	FJ870103
85.12%	FJ870103
90.16%	FJ870103
89.25%	FJ870103
90.08%	FJ870103
87.6%	FJ870103
85.95%	FJ870103
89.25%	FJ870103
87.6%	FJ870103
87.7%	FJ870103
85.83%	FJ870103
88.42%	FJ870103
80.83%	FJ870103
84.16%	FJ870103

89.25%	FJ870103
88.42%	FJ870103
89.16%	FJ870103
87.6%	FJ870103
90.08%	FJ870103
86.77%	FJ870103
90.0%	FJ870103
87.6%	FJ870103
87.7%	FJ870103
85.12%	FJ870103
85.12%	FJ870103
89.16%	FJ870103
88.42%	FJ870103
86.66%	FJ870103
89.25%	FJ870103
80.83%	FJ870103
85.12%	FJ870103
86.88%	FJ870103
84.29%	FJ870103
90.08%	FJ870103
85.12%	FJ870103
88.52%	FJ870103
90.0%	FJ870103
90.0%	FJ870103
87.6%	FJ870103
86.06%	FJ870103
86.77%	FJ870103
89.34%	FJ870103
86.77%	FJ870103
85.83%	FJ870103
85.95%	FJ870103
88.42%	FJ870103
88.42%	FJ870103
88.42%	FJ870103
89.25%	FJ870103
85.24%	FJ870103
85.12%	FJ870103
87.6%	FJ870103
87.6%	FJ870103
87.6%	FJ870103
88.42%	FJ870103
87.5%	FJ870103
89.25%	FJ870103
85.12%	FJ870103
88.42%	FJ870103
86.77%	FJ870103
84.16%	FJ870103
85.12%	FJ870103

86.77%	FJ870103
88.42%	FJ870103
86.66%	FJ870103
87.6%	FJ870103
85.95%	FJ870103
85.95%	FJ870103
84.29%	FJ870103
88.42%	FJ870103
85.12%	FJ870103
88.42%	FJ870103
86.77%	FJ870103
87.6%	FJ870103
88.42%	FJ870103
84.42%	FJ870103
88.52%	FJ870103
89.34%	FJ870103
87.6%	FJ870103
87.6%	FJ870103
89.25%	FJ870103
90.98%	FJ870103
90.9%	FJ870103
85.95%	FJ870103
85.95%	FJ870103
80.99%	FJ870103
88.42%	FJ870103
88.42%	FJ870103
85.95%	FJ870103
85.12%	FJ870103
85.83%	FJ870103
86.88%	KF301567
89.43%	KF301567
88.52%	KF301567
86.88%	KF301567
87.7%	KF301567
88.52%	KF301567
90.0%	JX310020
90.83%	JX310020
92.43%	JX310020
83.33%	JX310020
91.59%	JX310020
91.59%	JX310020
90.75%	JX310020
84.29%	FJ868186
85.12%	FJ868186
82.92%	FJ868188
81.81%	FJ868186
87.5%	FJ868186
95.53%	HM236335

87.7%	AY881632
89.43%	AY881632
93.49%	AY881632
90.24%	AY881632
87.7%	AY881632
88.52%	AY881632
86.88%	KF878932
89.34%	KF878932
86.88%	KF878932
90.16%	KF878932
88.52%	KF878932
89.34%	KF878932
90.16%	KF878932
80.48%	KF878931
88.52%	KF878932
93.54%	JQ723963
88.52%	JQ723963
89.43%	KF878932
86.06%	FJ876969
84.42%	FJ876969
90.16%	GQ292767
83.73%	EU744176
86.88%	EU744176
80.32%	FJ876969
81.14%	GQ292767
81.96%	EU744176
86.44%	U51554
93.33%	U51554
88.33%	U51554
88.23%	U51554
85.12%	U51554
85.0%	U51554
100.0%	AY103189
81.03%	JN885085
96.55%	AY103189
94.82%	AY103189
98.24%	HM236336
93.91%	HM236336
80.0%	AY103191
87.28%	FJ858379
94.91%	GQ214552
94.06%	GQ214552
85.12%	AY212805
85.0%	AY212805
86.77%	AY212805
87.93%	AY541690
90.67%	JN885091
87.28%	HM768743

98.27%	HM236338
95.68%	FJ648350
99.13%	AY835669
93.85%	FJ899594
99.05%	AY835669
93.1%	AY835669
100.0%	AY833087
93.1%	AY835669
95.32%	AY835669
85.59%	EF158848
80.7%	JN885083
100.0%	GQ465466
98.27%	Z22881
87.93%	AY103190
100.0%	AY103190
87.82%	EF486866
100.0%	EF486866
81.35%	JX310012
90.24%	Z29517 Z29539
93.38%	AF527756
83.03%	HQ445964
97.39%	JN867021
98.26%	JN867021
88.98%	FJ998031
88.88%	FJ998031
88.03%	FJ998031
84.61%	FJ998033
88.03%	FJ998034
91.45%	FJ998034
89.74%	FJ998034
91.37%	FJ998036
87.17%	FJ870068
97.41%	DQ057347
85.47%	FJ868205
86.95%	AY378112
86.2%	JN867020
87.93%	JN867020
83.62%	JN867020
84.48%	JN867020
82.9%	JN867020
86.44%	FJ868204
85.59%	FJ868204
84.61%	FJ870071
88.23%	FJ870070
88.13%	FJ870070
89.16%	FJ870070
92.37%	FJ870070
93.33%	FJ870070

89.91%	FJ870070
91.59%	FJ870070
93.27%	FJ870070
88.23%	FJ870070
93.27%	FJ870070
88.23%	FJ870070
84.03%	FJ870070
95.79%	FJ870070
90.75%	FJ870070
85.71%	FJ870070
93.27%	FJ870070
94.11%	FJ870070
91.59%	FJ870070
93.27%	FJ870070
88.13%	FJ870070
85.71%	FJ870070
87.39%	FJ870070
93.27%	FJ870070
91.59%	FJ870070
93.27%	FJ870070
88.23%	FJ870070
86.55%	FJ870070
91.59%	FJ870070
90.75%	FJ870070
88.23%	FJ870070
94.11%	FJ870070
91.59%	FJ870070
89.91%	FJ870070
87.39%	FJ870070
92.43%	FJ870070
89.07%	FJ870070
87.5%	FJ870070
91.59%	FJ870070
90.75%	FJ870070
89.91%	FJ870070
93.27%	FJ870070
94.11%	FJ870070
85.71%	FJ870070
90.0%	FJ870070
92.43%	FJ870070
92.43%	FJ870070
93.91%	FJ870070
89.83%	FJ870070
93.27%	FJ870070
91.66%	FJ870070
91.59%	FJ870070
91.59%	FJ870070
89.07%	FJ870070

91.52%	FJ870070
92.43%	FJ870070
89.07%	FJ870070
92.43%	FJ870070
100.0%	AF300282
85.59%	AF300283
91.3%	AF300282
87.39%	FJ998037
86.55%	FJ998037
87.28%	FJ865206
82.14%	FJ865206
98.14%	FJ865207
99.07%	FJ865207
96.29%	FJ865207
86.84%	AY102174
94.69%	AY331804
94.64%	AY331805
98.23%	EU600180
97.34%	DQ834370
98.23%	AY331804
90.51%	AY331804
92.92%	DQ834370
93.8%	AY331804
100.0%	AY331805
94.69%	DQ834370
85.71%	AY331804
88.79%	AY331804
90.43%	AY331804
89.43%	EF014286
85.0%	FJ858213
84.16%	FJ858213
87.8%	AM292313
83.73%	AM292313
85.36%	AM292312
82.92%	DQ487194
95.04%	FJ858215
99.16%	FJ858217
84.42%	FJ858217
83.33%	FJ858217
86.77%	FJ858217
94.11%	FJ858217
95.83%	FJ858217
87.6%	AM412525
94.21%	AM412525
98.34%	AM412525
94.21%	AM412525
89.43%	HM215513
91.05%	HM215513

94.26%	AM412525
94.21%	DQ270014
83.47%	AM412525
94.21%	AM412525
80.16%	AM412525
86.06%	AB486009
86.88%	X71140
88.52%	X71140
94.3%	X71140
100.0%	FJ876965
96.69%	FJ876965
95.04%	FJ876965
98.34%	AY541683
95.04%	AY541683
88.61%	FJ876963
90.08%	JQ781699
93.49%	FJ876963
100.0%	JQ781699
90.98%	JQ781699
95.86%	JQ781699
90.9%	AY541683
91.73%	JQ781699
91.05%	FJ876963
90.9%	JQ781699
90.98%	JQ781699
95.12%	FJ876963
90.98%	AY541683
93.38%	JQ781699
90.24%	JQ781699
92.68%	FJ876963
89.43%	FJ876963
86.99%	FJ876963
97.52%	AY541683
89.43%	FJ876963
97.52%	AY541683
93.49%	FJ876963
87.8%	FJ876963
92.56%	JQ781699
92.56%	AY541683
87.8%	FJ876963
92.56%	JQ781699
89.25%	AY541683
90.08%	JQ781699
94.21%	AY541683
88.42%	AY541683
95.04%	AY541683
95.04%	AY541683
90.08%	AY541683

88.61%	FJ876963
90.24%	DQ986131
89.43%	FJ876964
87.8%	FJ876964
95.12%	DQ986131
82.11%	FJ876964
87.8%	FJ876964
94.3%	DQ986131
93.49%	DQ986131
88.7%	FJ876964
94.3%	DQ986131
89.43%	FJ876964
89.43%	FJ876964
95.93%	FJ876964
94.3%	DQ986131
92.68%	DQ986131
92.68%	DQ986131
86.17%	FJ876964
91.86%	DQ986131
88.61%	FJ876964
92.0%	DQ986131
92.68%	DQ986131
91.05%	DQ986131
95.96%	DQ986131
88.61%	FJ876964
90.24%	DQ986131
89.43%	FJ876964
94.3%	DQ986131
92.68%	FJ876964
91.93%	DQ986131
89.43%	FJ876964
89.51%	DQ986131
90.16%	AY143573
89.25%	AY143573
90.9%	AY143573
93.38%	AY143573
94.26%	AY143573
90.08%	AY143573
91.8%	AY143573
91.8%	AY143573
90.9%	AY143573
91.73%	AY143573
93.38%	AY143573
90.98%	AY143573
91.73%	AY143573
85.83%	AY143573
92.56%	AY143573
91.8%	AY143573

85.83%	AY143573
85.12%	AY143573
86.44%	AY143573
88.42%	AY143573
84.29%	AY143573
89.16%	AY143573
88.42%	AY143573
84.29%	AY143573
88.42%	AY143573
90.08%	AY143573
86.77%	AY143573
90.9%	AY143573
88.42%	AY143573
85.83%	AY143573
88.42%	AY143573
87.7%	AY143573
88.88%	AY143573
89.25%	AY143573
88.7%	DQ393786
91.86%	DQ393786
85.36%	DQ393786
85.36%	DQ393786
97.56%	AM412524
97.56%	AM412524
91.86%	AM412524
91.86%	AM412524
92.68%	AM412524
93.49%	AM412524
95.93%	AM412524
99.17%	JQ408172
100.0%	JQ408166
98.34%	JQ408166
99.17%	JQ408166
99.17%	JQ408166
97.52%	JQ408166
98.34%	JQ408166
96.69%	JQ408166
98.34%	JQ408166
98.34%	JQ408166
99.17%	JQ408166
82.92%	JX178854
95.04%	JX178900
86.17%	JX178900
98.34%	JQ408162
100.0%	JX178900
93.02%	AB640683
94.21%	EU399542
85.12%	JX178862

98.36%	JQ924057
97.52%	JQ924057
85.12%	JQ924057
95.86%	JQ924057
99.18%	JQ924057
98.34%	JQ924057
95.04%	JQ924057
95.53%	JQ924057
98.34%	JQ924057
96.69%	JQ924057
97.52%	JQ924057
97.52%	JQ924057
99.17%	EU399538
100.0%	JQ408169
98.18%	JX101854
96.69%	JX000469
91.93%	JX000467
89.25%	JX000467
94.21%	JX000467
95.86%	AY143568
95.86%	AY143568
86.77%	AY143568
95.04%	AY143568
95.04%	AY143568
99.17%	JQ392572
95.04%	JQ392572
93.38%	JQ392572
99.18%	JQ924052
88.42%	JQ924052
88.42%	JQ924052
85.95%	JQ924052
95.04%	JQ924052
87.6%	JQ924052
95.08%	JQ924052
88.42%	JQ924052
88.42%	JQ924052
93.44%	JQ924052
90.16%	JQ924052
97.52%	JQ924052
91.73%	JQ924052
88.61%	JQ924052
96.69%	JQ924052
96.69%	JQ924052
100.0%	JQ408175
97.52%	AY143566
89.25%	JQ408175
89.25%	JQ408175
83.47%	JQ408175

95.08%	JQ408175
99.17%	AY143566
99.17%	AY143566
99.17%	AY143566
95.53%	AY143566
93.33%	AY143566
86.77%	AY143566
92.68%	JQ837816
92.68%	JQ837816
98.37%	JQ924059
91.05%	JQ924059
97.52%	EU399530
88.7%	JQ924059
99.18%	JQ924059
98.37%	JQ924059
91.4%	JQ408156
99.17%	JQ408176
99.17%	JQ408176
80.0%	JQ408156
96.52%	JQ408176
98.34%	JQ408176
100.0%	JQ408176
99.17%	JQ408176
96.69%	JQ408176
99.17%	JQ408176
99.17%	JQ408176
98.33%	JQ408156
93.33%	JQ408156
96.77%	JQ408159
96.74%	JQ408159
98.37%	JN871721
96.74%	JQ408159
95.93%	JQ408159
90.24%	JQ408159
94.35%	JQ408159
95.12%	JQ408159
92.68%	JQ408159
92.68%	JQ408159
95.96%	JQ408155
100.0%	JQ408155
98.37%	EU399536
95.12%	JQ408155
97.56%	JQ408155
91.93%	EU399536
94.3%	EU399536
98.37%	JQ408155
98.37%	EU399536
95.12%	EU399536

91.05%	JQ408155
94.3%	JQ408155
92.68%	JQ408155
94.3%	JQ408155
99.15%	JQ408155
90.98%	JQ408155
98.37%	JQ408155
98.37%	JQ408155
93.49%	JQ408155
95.12%	JQ408155
96.74%	JQ408155
100.0%	AY143563
90.32%	AY143563
94.21%	JQ408171
90.9%	JX178773
87.6%	JX178773
88.42%	JX178773
94.26%	JX178773
95.08%	JX178773
82.64%	JX178773
89.25%	JX178773
100.0%	EU399535
88.0%	EU399535
80.8%	JQ837819
88.88%	EU399535
95.08%	JQ408160
100.0%	JQ408160
95.04%	JQ408160
86.06%	JQ408160
95.04%	JQ408160
81.65%	FJ346568
100.0%	HM154534
100.0%	AJ305251
82.3%	FR873721
80.35%	HM140406
100.0%	EF094972
85.04%	FJ346568
97.34%	JF903800
82.75%	JN172996
82.75%	JF701983
84.48%	JF701983
81.19%	JN172996
89.34%	AF508778
89.25%	AF508777
86.77%	AF508778
87.6%	AF508777
90.9%	AF508777
100.0%	KC287214

94.95%	KC287214
85.48%	JX025560
84.67%	AY302563
96.74%	AY302563
83.73%	AY302563
83.73%	AY302563
82.92%	AY302563
83.06%	AY302563
82.11%	AY302563
86.61%	AY302563
85.36%	AY302563
83.6%	AY302563
85.36%	AY302563
83.73%	AY302563
85.36%	AY302563
82.67%	AY302563
84.12%	AY302563
84.55%	AY302563
83.73%	AY302563
82.11%	AY302563
84.55%	AY302563
82.25%	AY302563
86.17%	AY302563
83.73%	AY302563
86.17%	AY302563
84.55%	AY302563
82.92%	AY302563
84.55%	AY302563
83.2%	AY302563
83.73%	AY302563
82.25%	AY302563
83.87%	AY302563
83.73%	AY302563
83.06%	AY302563
86.99%	AY302563
84.0%	AY302563
83.73%	AY302563
82.11%	AY302563
83.73%	AY302563
82.11%	AY302563
83.87%	AY302563
81.3%	AY302563
85.36%	AY302563
82.11%	AY302563
81.74%	AY302563
82.92%	AY302563
84.67%	AY302563
82.92%	AY302563

85.48%	AY302563
82.11%	AY302563
83.73%	AY302563
84.55%	AY302563
84.92%	AY302563
84.55%	AY302563
82.92%	AY302563
85.36%	AY302563
83.73%	AY302563
80.64%	AY302563
83.87%	AY302563
81.74%	AY302563
83.87%	AY302563
84.55%	AY302563
83.73%	AY302563
82.11%	AY302563
84.25%	AY302563
83.73%	AY302563
81.3%	AY302563
83.73%	AY302563
84.8%	AY302563
83.73%	AY302563
83.06%	AY302563
82.11%	AY302563
80.48%	AY302563
86.99%	AY302563
81.3%	AY302563
86.17%	AY302563
86.17%	AY302563
93.54%	AY302563
82.25%	AY302563
82.92%	AY302563
82.11%	AY302563
98.37%	AY302563
83.73%	AY302563
83.2%	AY302563
81.6%	AY302563
82.11%	AY302563
82.92%	AY302563
84.55%	AY302563
84.55%	AY302563
80.48%	AY302563
85.36%	AY302563
83.73%	AY302563
99.19%	FJ422990
90.24%	FJ876958
80.48%	FJ422988
90.4%	FJ422988

95.96%	FJ422990
87.3%	FJ422988
86.88%	FJ377547
98.38%	EF486862
95.96%	EF486862
95.96%	EF486862
95.96%	EF486862
88.42%	FJ422991
98.38%	FJ543107
98.38%	JF791016
92.74%	FJ543107
94.35%	JF791016
93.49%	JX178850
92.68%	JX178826
95.96%	JF791016
96.77%	JF791016
95.16%	JF791016
92.74%	JX178826
95.16%	JF791016
96.77%	JF791016
98.37%	JX178850
98.38%	JF791016
96.77%	JF791016
98.37%	JX178850
96.74%	JX178850
97.58%	AY541684
91.93%	JX178850
97.58%	JF791016
95.96%	JF791016
94.35%	FJ543107
99.19%	JF791016
95.96%	JF791016
98.38%	JF791016
99.19%	JF791016
86.06%	DQ662848
91.05%	DQ662848
96.82%	JF791016
98.38%	JF791016
95.96%	JF791016
98.38%	JF791016
93.54%	FJ543107
83.73%	JX178826
94.35%	JF791016
93.54%	JX178850
96.77%	JF791016
89.51%	AY143564
99.19%	JF791016
87.2%	U97112

84.55%	JX178850
98.38%	JF791016
95.96%	JF791016
88.61%	JX178850
93.49%	JX178850
96.77%	JF791016
91.05%	JF791016
97.58%	JF791016
95.16%	FJ543107
94.35%	FJ543107
97.58%	JF791016
97.58%	JF791016
96.77%	FJ543107
98.38%	JF791016
95.12%	JF791016
97.58%	JF791016
95.93%	DQ662848
90.32%	FJ543107
97.58%	JF791016
98.38%	JF791016
94.4%	JF791016
91.05%	DQ662848
91.86%	JX178850
98.38%	JF791016
94.3%	JX178850
88.7%	FJ543107
86.99%	JX178850
94.3%	U97112
87.09%	JF791016
97.58%	JF791016
96.77%	JF791016
98.4%	JF791016
97.58%	JF791016
87.09%	JF791016
95.16%	JF791016
91.93%	JF791016
88.61%	JX178850
91.12%	FJ543107
95.96%	FJ543107
94.35%	FJ543107
96.77%	JF791016
97.58%	JF791016
97.58%	JF791016
98.36%	JF791016
96.77%	JF791016
96.77%	JF791016
94.3%	FJ543107
97.56%	JF791016

86.17%	DQ811090
94.65%	FJ422993
96.0%	JX178820
91.93%	JX178806
84.67%	JX178806
86.4%	JX178820
87.4%	FJ422993
83.2%	JX178802
90.4%	JX178820
98.38%	JX178820
92.74%	FJ422993
97.58%	FJ422993
93.54%	JX178820
95.16%	JX178820
95.96%	JX178820
89.51%	JX178788
99.19%	JX178820
96.0%	FJ422993
96.85%	JX178820
99.19%	FJ422993
98.38%	JX178820
99.19%	JX178820
98.38%	JX178820
95.16%	FJ422993
89.43%	JX178820
98.38%	FJ422993
95.96%	JX178820
99.19%	JX178820
97.58%	JX178820
98.38%	FJ422993
95.16%	JX178820
86.29%	FJ422993
95.16%	JX178820
99.19%	JX178820
99.19%	JX178820
91.12%	JX178800
95.96%	FJ422994
90.4%	FJ422994
94.3%	FJ422994
95.12%	FJ422994
87.2%	FJ422994
83.06%	FJ422994
87.9%	FJ422994
89.43%	FJ422994
87.8%	FJ422994
86.99%	FJ715634
94.3%	FJ422994
93.49%	FJ422994

95.12%	FJ422994
83.73%	FJ715634
94.3%	FJ422994
86.17%	FJ715634
88.7%	FJ422994
95.12%	FJ422994
90.32%	FJ422994
91.05%	FJ422994
86.99%	FJ422994
94.3%	FJ422994
95.12%	FJ422994
80.48%	FJ422994
94.3%	FJ422994
94.3%	FJ422994
95.79%	FJ422994
89.51%	FJ422994
86.99%	FJ715634
94.91%	AY217727
98.3%	X65153
89.74%	AY217727
90.08%	AY547545
83.87%	DQ503583
87.2%	DQ503583
83.06%	DQ503583
89.6%	DQ503583
98.37%	EU399529
89.43%	JX946277
91.86%	JX946277
88.61%	DQ022065
92.56%	AF164125
89.43%	AM412772
86.77%	AF508764
91.8%	FN429123
87.6%	JX139117
86.77%	JX139117
86.99%	FJ870099
86.99%	EF194083
86.17%	FJ870099
86.99%	FJ870099
86.17%	EF194083
96.74%	EF194081
90.9%	FM209294
88.42%	AF508768
90.98%	AF508772
85.12%	FM209295
90.9%	X03947 M14600
90.08%	JQ723980
90.24%	GU170843

86.29%	AM412774
85.03%	X53485
90.08%	X53485
89.25%	X53485
89.25%	X53485
89.25%	X53485
91.73%	AJ310485
88.42%	FJ870089
91.59%	FJ008721
82.5%	KC991098
90.24%	AB449362
91.05%	AB449362
90.24%	AB449362
90.24%	AB449362
96.74%	HQ699895
91.05%	AB449362
89.43%	AB449362
92.68%	AB449362
90.24%	AB449362
91.93%	AB449362
90.24%	AB449362
84.67%	AB449362
85.95%	AB449365
88.61%	AB449362
84.55%	AB449362
95.12%	HQ699895
88.61%	AB449362
88.61%	AB449362
87.7%	FJ156105
96.58%	DQ059583
96.58%	DQ059583
96.74%	HM623916
88.61%	EU220227
95.93%	EU220227
100.0%	HM623917
86.88%	DQ359728
80.16%	FJ775723
82.64%	DQ227798
86.77%	JQ723985
96.74%	AY294646
90.9%	JQ723985
90.08%	JQ723985
90.16%	JQ723985
90.08%	JQ723985
90.08%	JQ723985
92.56%	JQ723985
91.73%	JQ723985
86.17%	JQ723985

87.9%	DQ490236
83.8%	AM295495
81.48%	AM295495
81.65%	DQ168806
81.81%	DQ168806
99.13%	EU583992
96.42%	EU583993
92.85%	AJ537427
91.96%	AJ537427
95.53%	EU583993
82.3%	AJ277877
97.27%	AF527758
82.52%	U24248
85.29%	U24248
82.07%	JX015375
81.73%	JX015378
83.65%	JX015378
82.52%	JQ768407
82.52%	JQ768407
81.73%	JX015378
83.8%	JX015375
82.07%	JX015373
83.01%	JX015375
82.69%	JX015378
80.18%	JX015375
83.8%	JQ768407
82.69%	FJ463746
